# Fragmentation of Vancoresmycin Reveals a Strong Dependence on Global Molecular Architecture for Antibacterial Activity

**DOI:** 10.1002/cmdc.70409

**Published:** 2026-07-30

**Authors:** Max Schönenbroicher, Florian Heppner, Maximilian Seul, Simon Morgenschweis, Aia Ali Abdelrahman, Eva Fahr, Niklas Holstein, Anna Müller, Dirk Menche

**Affiliations:** ^1^ Kekulé‐Institute for Organic Chemistry and Biochemistry University of Bonn Bonn Germany; ^2^ Institute of Pharmaceutical Microbiology University Hospital Bonn University of Bonn Bonn Germany; ^3^ German Center for Infection Research (DZIF), Partner Site Bonn‐Cologne Bonn Germany

**Keywords:** amycomycin, antibiotics, tetramic acids, vancoresmycin

## Abstract

Vancoresmycin is a structurally complex tetramic‐acid‐containing natural product with potent activity against Gram‐positive bacteria, yet its structure–activity relationships remain poorly defined. In particular, it is unclear whether discrete substructures contribute independently to antibacterial activity or whether biological function depends on the integrity of the full molecular framework. Here, we report the synthesis and biological evaluation of a series of structurally defined fragments derived from vancoresmycin and its aglycon amycomycin, including the tetramic acid core, eastern and western polyketide segments, and the mycosamine residue. Despite preserving key structural motifs, the fragments did not exhibit considerable antibacterial activity. Only the western fragment displayed weak activity accompanied by indications of membrane‐associated stress. These results demonstrate that antibacterial activity in vancoresmycin cannot be attributed to isolated substructures but instead depends on the integrated molecular architecture. These results provide a framework for future design strategies that preserve global structural properties rather than focusing on discrete motifs.

## Graphical abstract

Fragmentation of the natural product vancoresmycin reveals an essential role of global architecture for biological potency.

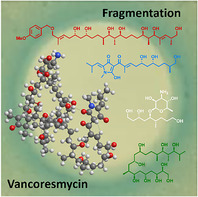

## 1 Introduction

Natural products continue to represent a valuable source of structurally diverse antibacterial agents, particularly in the context of combating increasing resistance of multidrug‐resistant Gram‐positive pathogens, including methicillin‐resistant *Staphylococcus aureus* (MRSA) and vancomycin‐resistant enterococci (VRE). However, the translation of structurally complex natural products into simplified, tractable scaffolds remains a central challenge in medicinal chemistry, as it is often difficult to establish whether biological activity arises from discrete structural motifs or from the collective properties of the full molecular architecture. Tetramic acid‐containing natural products exemplify this ambiguity. While the 2,4‐pyrrolidinedione motif is frequently associated with antibacterial activity, its precise functional role remains incompletely understood. Suggested roles include β‐dicarbonyl metal chelation, tautomeric flexibility and participation in amphiphilic molecular organization [1, 3].

**SCHEME 1:**
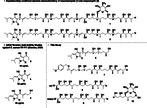
(A) Vancoresmycin (**1**) and amycomycin (**2**) with their recently confirmed stereochemical structure [8, 9]. (B) Screening of various simplified tetramic acids revealed acids **3**, **4,** and **5** with low antibacterial properties (MICs against *S. aureus* SG511) [10]. (C) In this study, the synthesis and biological evaluation of advanced tetramic acid **6**, western and eastern fragments **7**–**9,** and mycosamine **10** are examined.

Vancoresmycin (**1**) and its aglycon amycomycin are particularly compelling representatives of this class. Isolated from *Amycolatopsis* sp. ST 101170, vancoresmycin comprises a tetramic acid moiety linked to a highly oxygenated polyketide backbone and a mycosamine residue [4]. The compound exhibits potent activity against Gram‐positive bacteria, including MRSA and VRE (MIC ≈ 0.125–2 µg/mL), while lacking activity against Gram‐negative strains. Interestingly, the aglycon amycomycin (**2**) retains antibacterial activity (MIC ≈ 1–8 µg/mL), indicating that glycosylation is not strictly required for function [5]. Mechanistic studies suggest a concentration‐dependent depolarization of the bacterial cytoplasmic membrane without classical pore formation [6]. Previous synthetic efforts on vancoresmycin (**1**) have primarily focused on synthetic access to advanced intermediates and confirming stereochemical assignments, while systematic evaluation of structure–activity relationships (SARs) remained limited [7, 9]. Notably, simplified tetramic acid derivatives **3**–**5** (Scheme 1) have been reported to retain low antibacterial activity against *S. aureus*, suggesting that partial structural elements may be sufficient [10]. However, these studies do not directly address whether fragments derived from the native vancoresmycin scaffold retain activity.

In this context, the present study evaluated the independent contribution of individual structural elements of vancoresmycin to antibacterial activity. To this end, we prepared a series of fragments representing the tetramic acid core (**6**), western (**7**), and eastern (**8**, **9**) polyketide substructures and the mycosamine moiety (**10**). By comparing their biological activity, we aimed to determine whether antibacterial function can be retained upon structural simplification or whether it depends on the integrity of the full molecular framework.

## 2 Results and Discussion

The synthetic work focused on the preparation of structurally defined fragments that preserve all key features of the parent scaffold while enabling independent evaluation of their biological activity. The target set included the tetramic acid core **6**, the eastern and western polyketide substructures (**7**–**9**), and the mycosamine fragment **10**. More elaborate tetramic acid moiety **6** was selected as a more extended version of previously studied simplified fragments **3**–**5**, while Western and Eastern subunits **7** and **8** were chosen to evaluate the biological influence of either part of these polyketide antibiotics, also to see whether the full structure could be truncated. Finally, the mycosamine moiety was targeted to find out the influence of this structural element, as this is also part of various further potent metabolites.

**SCHEME 2:**
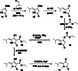
Optimized synthetic sequence to tetramic acid **17**.

The synthesis of tetramic acid **6** commenced from commercially available pyrrolidinone **11**, which served as a versatile precursor for the functionalized tetramic acid core (Scheme 2). Initial studies focused on a base‐mediated olefination with isobutyraldehyde and NaOH, delivering the corresponding olefin **12** in excellent yield and diastereoselectivity [11]. Subsequent methylation (MeI/NaH) and TFA‐mediated cleavage of the methoxy ether provided intermediate **17** as an inseparable mixture with limited diastereoselectivity (d.r. = 3:1), restricting the applicability of this approach and prompting revision of the protecting group strategy. A focused evaluation of alternative protecting groups revealed incompatibility of TBS, benzyl, or acetate with installation or the subsequent methylation step. In contrast, ethyl chloroformate protection of substrate **13** furnished the corresponding carbamate **14** in 84% yield [12]. Subsequent application of Yoda’s rearrangement conditions (CaCl_2_, NEt_3_, DMAP) afforded the 3‐alkoxycarbonyl tetramic acid **15** in 96% yield [13]. This transformation, previously validated for the synthesis of 3‐acyl tetramic acids, proved equally powerful in the present context. Selective *N*‐methylation using MeI and NaHMDS proceeded smoothly, affording *N*‐methyl 3‐alkoxycarbonyl tetramic acid **16**, after which thermal hydrolysis in ethyl acetate/water (80 °C) enabled clean deprotection to tetramic acid **17**. Notably, this revised sequence furnished product **17** with excellent diastereoselectivity (d.r. > 20:1), representing a substantial improvement in terms of more reliable high selectivity compared to the initial approach.

**SCHEME 3:**
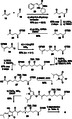
Synthesis of tetramic acid **6,** with the key steps being the organocatalyzed Michael addition and aldol addition.

Acid **6** was accessed from methyl vinyl ketone (**18**) and propanal (**19**, Scheme 3). Organocatalyzed Michael addition, employing (*R*)‐phenylalanine‐derived amine **20**, furnished ketoaldehyde **21** in 68% yield with high enantioselectivity (e.r. = 92:8) [14, 15]. Notably, this transformation proved highly sensitive to substrate purity. Rigorous distillation of both coupling partners was essential to ensure consistent performance, as trace impurities led to a significant reduction in yield (26%). Reduction of ketoaldehyde **21** with LiAlH_4_ afforded the corresponding diol, enabling selective protection of the primary alcohol as its TBS ether **22**. Subsequent Parikh–Doering oxidation cleanly gave the desired methyl ketone **23** in 96% yield. With this intermediate in hand, Paterson aldol coupling with aldehyde **24** was examined [8]. Under optimized conditions (1.2 equiv. of (–)‐Ipc_2_BCl, 2.0 equiv. NEt_3_, DCM), β‐hydroxyketone **25** was obtained in 55% yield with moderate diastereoselectivity (d.r. = 3:1) [16]. Subsequent Narasaka‐Prasad *syn*‐reduction yielded the corresponding 1,3‐diol in excellent diastereoselectivity (d.r. > 20:1), which was protected as bis‐TBS ether **26** [17, 18]. Attempts to form a cyclic silyl ether using (*t*‐Bu)_2_SiCl_2_ were hampered by poor reproducibility (31%–77% yield) and were not pursued further [19]. Oxidative PMB removal using DDQ proceeded smoothly and was accompanied by in situ oxidation of the resulting allylic alcohol to the corresponding aldehyde. Subsequent Pinnick oxidation furnished acid **27**, which was coupled to tetramic acid **17** under Steglich conditions (DCC, DMAP) to give the *O*‐acylated intermediate **28** [20]. Finally, rearrangement under Yoda’s conditions (CaCl_2_, NEt_3_, DMAP) enabled efficient *O*‐ to *C*‐transfer, delivering the desired *C*‐acylated tetramic acid **29** in excellent yield [7]. This transformation highlights the utility of mild Lewis acid‐mediated rearrangements for accessing *C*‐acyl tetramic acid architectures that are otherwise challenging to obtain.

With tetramic acid **29** in hand, attention turned to the final global TBS deprotection to furnish the fully deprotected tetramic acid **6**. This transformation, however, emerged as a major bottleneck due to the pronounced acid‐ and base‐sensitivity of the tetramic acid core. Previous experiments on the corresponding acetonide‐protected analog had demonstrated that both Brønsted‐acid‐mediated (HCl, PPTS, HOAc) and Lewis‐acid‐mediated conditions (BiCl_3_, PdCl_2_(MeCN)_2_) led to rapid decomposition. In line with observations by Schobert et al., the hydroxy group at C5 is likely to play a critical role in destabilizing the tetramic acid core [21]. Given these limitations, attention was directed toward fluoride‐mediated protocols, exploiting the intrinsic lability of silyl ethers. However, conventional conditions proved ineffective. Treatment with TBAF, irrespective of stoichiometry, did not afford the desired product (Table 1, entries 1 and 2), while also introducing significant analytical complications due to persistent tetrabutylammonium residues. Catalytic amounts of TBAF in THF/pH 7 likewise failed to improve the outcome (entry 3) [22]. Similarly, alternative fluoride sources (HF·py, NEt_3_·HF) and Lewis acidic conditions (PdCl_2_(MeCN)_2_ in MeCN/H_2_O) did not enable clean deprotection (entries 4–6), highlighting the narrow reactivity window of this substrate [23, 24]. Reconsideration of fluoride‐mediated deprotection focused on mitigating base‐induced side reactions arising from residual hydroxide in hydrated TBAF solutions. In this context, precedent from Myers’ studies on the kedarcidin core provided a useful guideline, identifying nitrophenols as suitable additives [25]. Systematic evaluation revealed 2‐nitrophenol as an optimal hydroxide scavenger, possessing a p*K*_a_ sufficient to suppress elimination pathways while maintaining effective silyl cleavage. Gratifyingly, application of these conditions enabled global TBS deprotection of tetramic acid **29**, affording the desired product **6** in 47% yield, albeit with residual tetrabutylammonium impurities. Further optimization, most notably, the addition of an extra four equivalents of TBAF after 24 h, significantly improved the yield up to 84%. However, isolation and purification of the final product remained challenging. HPLC purification was accompanied by substantial material loss (11% yield), attributed to tautomerism‐induced peak broadening. Attempts to mitigate this behavior through solvent buffering were unsuccessful, consistent with the intrinsic sensitivity of tetramic acid **6** toward both acidic and basic conditions. Despite these constraints, sufficient material of the fully deprotected tetramic acid **6** was obtained in analytically pure form to enable subsequent biological evaluation.

**TABLE 1:** Screened deprotection conditions for TBS protection of compound 29 to yield tetramic acid 6.

Entry	Conditions	Yield
1	TBAF (9.0 eq.), THF, 65 h	—
2	TBAF (15.0 eq.), THF, 24 h	—
3	TBAF (0.72 eq.), THF/pH 7 buffer, 72 h	—
4	HF·py, THF, pyridine, 16 h	—
5	NEt_3_·3HF, DCM, 7 h	—
6	PdCl_2_(MeCN)_2_, MeCN/H_2_O, 48 h	—
7	TBAF (4.6 eq.), *o*‐nitrophenol (4.6 eq.), THF, 168 h	47%
8	TBAF (4.1 + 4.0 eq.), *o*‐nitrophenol (4.7 eq.), THF, 67 h	84%
9	TBAF (8.3 eq.), THF/HOAc, 48 h	—

**SCHEME 4:**
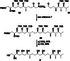
Synthesis of eastern fragment **8** starting from fully protected **32**, which has recently been synthesized in an HWE/β‐boration sequence with phosphonate **30** and aldehyde **31** [9].

The eastern fragment **8** was assembled in a concise and scalable sequence starting from known phosphonate **30** and aldehyde **31** (Scheme 4) [9]. HWE‐coupling and subsequent β‐boration enabled efficient access to the fully protected fragment **32** in excellent yields and diastereoselectivities. Global deprotection was accomplished in a stepwise manner, starting with selective PMB removal using DDQ, followed by acidic cleavage of the TBS groups, to furnish polyol **8** in 25% yield.

**SCHEME 5:**
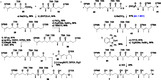
(A) Initially attempted synthesis of surrogate **39** by advanced aldol coupling between ethyl ketone **37** and aldehyde **38**. (B) Successful synthesis of western fragment **7** by HWE coupling of aldehyde **40** and phosphonate **41** [8].

The synthesis of the western fragment **7** proved considerably more challenging and necessitated strategic revision of the initial approach. Early studies focused on HWE olefination between phosphonate **33** and aldehyde **34** (see Scheme 5A). However, aldehyde **34** displayed a pronounced susceptibility toward base‐induced elimination, furnishing the corresponding α,β‐unsaturated aldehyde. A comprehensive screening of reaction conditions identified Ba(OH)_2_ (1.0 equiv.) as uniquely effective in suppressing this decomposition pathway, affording the corresponding enone in 69% yield [26]. Afterwards, the enone was subjected to regioselective 1,4‐reduction with Lipshutz’s "hot Stryker" reagent, providing ketone **35** in 94% yield [27]. Formation of the desired 1,3‐*syn* motif required a multistep sequence involving TES ether cleavage using PdCl_2_, which was accompanied by loss of the primary TBS group. Subsequent Narasaka‐Prasad *syn*‐reduction with excellent diastereoselectivity (d.r. > 20:1) and TBS protection afforded the fully protected fragment **36** [17, 18]. Selective removal of the primary TBS group using HF·pyridine enabled further functionalization via Parikh–Doering oxidation, Grignard addition of EtMgBr, and DMP oxidation, ultimately yielding ethyl ketone **37** in overall good yields. Despite this extensive sequence, the key aldol coupling between ketone **37** and aldehyde **38** proceeded in only 10% yield [28]. Although this transformation had shown promising reactivity in a simplified model system, the low yield and inability to reliably assign the newly formed stereocenter rendered this route unsuitable for further development. In response, the synthetic design was revised to address these limitations. Furthermore, the ethyl ketone moiety was replaced with a primary alcohol to mitigate undesired ketal formation during the final global deprotection step. In a more efficient sequence, aldehyde **40** and phosphonate **41** were smoothly coupled under HWE conditions to furnish enone **42** (Scheme 5B) [8]. Subsequent conjugate reduction, TMS deprotection and Narasaka‐Prasad *syn*‐reduction provided diol **43** with excellent diastereoselectivity (d.r. > 20:1). Final global deprotection of the TBS groups under acidic conditions proceeded cleanly, ultimately delivering the western fragment **7** for further biological evaluation.

**SCHEME 6:**
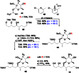
Modified mycosamine donor **44** enabled efficient preparation of mycosamine **10** by facile modifications after glycosylation [9].

We next turned our attention to the synthesis of mycosamine fragment **44** (Scheme 6), a structurally and synthetically challenging deoxyaminosugar for which only limited preparative approaches have been reported [29, 33]. To ensure reliable access, Carreira’s route was adopted and slightly modified, providing TBS‐protected mycosamine donor **44** (see ref. [22]) [9, 33]. Glycosylation with model acceptor **45** under Carreira’s conditions for sterically hindered alcohols proceeded smoothly, affording the desired β‐glycoside TBS‐**48** in 92% yield and good diastereoselectivity (d.r. = 12:1) [34]. Subsequent inversion at C2′ was achieved via a three‐step sequence involving selective benzoate deprotection with NaOMe, oxidation of the liberated alcohol and stereoselective reduction of the resulting ketone, to furnish the C2′‐inverted diastereomer TBS‐**49** in 73% yield with excellent diastereoselectivity (d.r. > 20:1) [35]. However, final global deprotection of the TBS‐protected intermediate with HCl proved problematic, as only the C4′‐mono‐TBS‐protected species could be obtained. Alternative fluoride‐based conditions (NEt_3_·3HF) were similarly ineffective, underscoring the limited compatibility of the TBS protecting group. This prompted a strategic redesign of the glycosyl donor. Specifically, the C4′‐OH group was protected as the more labile TES ether, affording modified donor TES‐**44** [9]. Gratifyingly, glycosylation with TES‐**44** not only proceeded efficiently but also delivered improved diastereoselectivity (d.r. = 18:1), suggesting a beneficial influence of the altered steric environment. Because NaOMe‐mediated benzoate deprotection led to partial TES cleavage, DIBAL‐H was employed, which enabled selective removal of the benzoate moiety without compromising the TES ether [36]. Subsequent C2′‐inversion furnished alcohol TES‐**49** in 86% yield, maintaining excellent diastereoselectivity (d.r. = 18:1). Finally, global deprotection under acidic conditions, followed by Staudinger reaction, provided the fully elaborated mycosamine fragment **10** [37].

With all compounds in hand, we evaluated their antibacterial activity by determining minimum inhibitory concentrations (MICs) against a small panel of test strains in direct comparison with the natural product vancoresmycin (**1**, Table 2), which displays potent activity against Gram‐positive pathogens, including multi‐drug‐resistant strains [6]. Across the series, all compound fragments exhibited markedly reduced antibacterial activity, despite retaining substantial elements of the parent scaffold, including the tetramic acid side chain (**6**), extended polyketide segments (**7–** **9**), and the mycosamine moiety (**10**). Only the western fragment **7** displayed detectable activity against tested staphylococci with potency comparable to that of the simplified tetramic acid moiety **4** [10]. These results indicate that retention of isolated structural motifs alone is insufficient to preserve antibacterial activity, highlighting the importance of the overall molecular architecture. This discrepancy may relate to differences in global physicochemical properties, such as lipophilicity, amphiphilicity, or aggregation behavior, which were not examined.

**TABLE 2:** MIC values for synthesized compounds against a set of Gram‐positive test strains.

Organism		MIC, µg mL^−1^					
		Compound					
		1	6	7	8	9	10
*S. aureus* HG001		0.25	n.t.	16	>64	n.t.	n.t.
*S. aureus* USA 300 (MRSA)		0.25	n.t.	16	>64	n.t.	n.t.
*S. aureus* Mu50 (VISA)		0.5	n.t.	16	>64	n.t.	n.t.
*S. aureus* SG511		0.063	>64	32	>64	>64	>64
*E. faecalis* JH 2–2		0.25	n.t.	>64	>64	n.t.	n.t.
*E. faecalis* V583 (VRE)		0.25	n.t.	>64	>64	n.t.	n.t.
*B. subtilis* 168		0.5	>64	>64	>64	>64	>64

**FIGURE 1:**
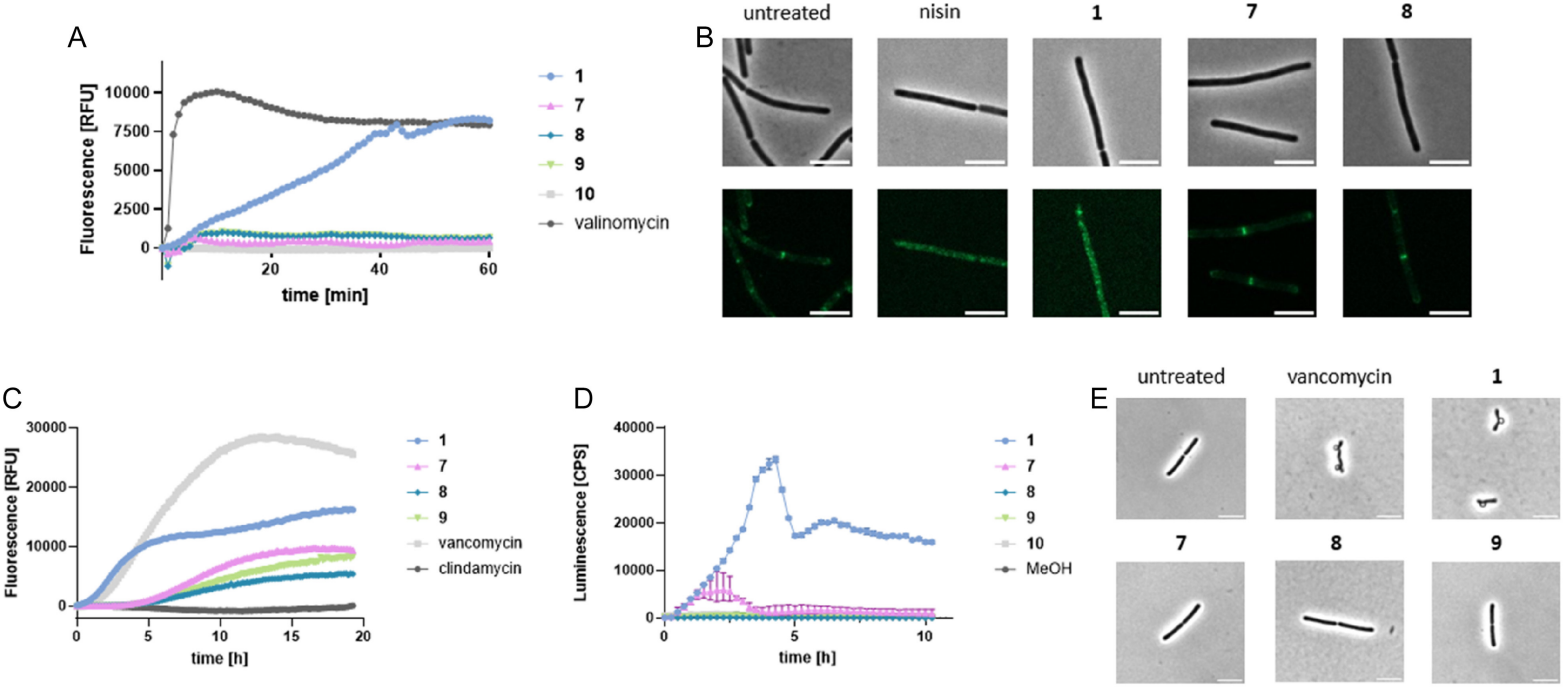
Vancoresmycin fragments partially retain cell wall inhibitory activity but do not affect membrane potential. (A) While the ionophore valinomycin (5 µM, gray circles) caused rapid membrane depolarization, **1** (0.5 µg mL^−1^, blue circles) gradually affected the cytoplasmic membrane. No effect was observed for any of the fragments (64 µg mL^−1^). Membrane potential was monitored using the voltage‐sensitive dye DiSC_3_(5) and is reported as relative fluorescent units (RFU). (B) Fluorescence microscopy revealed that **1** affects membrane potential in *B. subtilis*, resulting in delocalization of the membrane‐potential‐dependent spatial regulator MinD in early exponential phase. **1** induced the formation of distinct fluorescent foci comparable to the pore‐forming control nisin, whereas none of the fragments affected MinD cellular localization. Phase contrast (upper panels) and fluorescence microscopy (lower panels) images of *B. subtilis* cells expressing GFP‐MinD treated with 2× MIC of **1**, 5× MIC of nisin, 256 µg mL^−1^ **8**, or **9**, as well as untreated control cells, are shown. Scale bar = 5 µm. (C) **1** and all tested fragments moderately induce cell wall stress in *B. subtilis* compared to the glycopeptide antibiotic vancomycin, a known peptidoglycan biosynthesis inhibitor. Measured fluorescence is expressed as RFU, and maximum induction curves are shown. (D) Treatment with **1** (blue circles) strongly induced *P*_lial_ as observed by expression of the *Photorhabdus luminescens lux* operon in *B. subtilis* *P*_liaI_‐lux, while only **7** (rose triangles) retained this activity, although at a much lower level. Measured luminescence is expressed as counts per second (CPS), and maximum induction curves are shown. (E) **1** and the glycopeptide vancomycin caused pronounced cell‐shape deformations and induced blebbing in *B. subtilis*, characteristic of cell wall inhibitors. Interference with cell wall biosynthesis results in a weakened cell wall and facilitates protrusions of the cytoplasmic membrane. Phase contrast microscopy images of *B. subtilis* 168 treated with 1× MIC of **1**, **7**, **8**, **9**, or vancomycin (control antibiotic) are shown in comparison to untreated control cells. Scale bar = 5 µm. All experiments were performed in three biological replicates with technical replicates and are shown as representative images or mean ± SD.

Because vancoresmycin selectively targets the cytoplasmic membrane and induces concentration‐dependent depolarization [6], we compared the effects of compounds **1** and **7–10** in a DiSC_3_(5) depolarization assay. The hydrophobic, potentiometric probe accumulates in energized cells, where it undergoes self‐quenching [38]. Upon membrane perturbation or depolarization, dye release results in an increase in fluorescence over time. Vancoresmycin (**1**) induced depolarization in *S. aureus*, albeit more slowly than the ionophore valinomycin, whereas none of the synthesized fragment compounds affected the cytoplasmic membrane under the same conditions (Figure 1A). To exclude species‐specific effects, we investigated membrane potential‐dependent localization of GFP‐labeled MinD in *Bacillus subtilis* (*B. subtilis*). The spatial regulator MinD promotes cell division at midcell and prevents divisome formation close to the cell poles and delocalizes but delocalizes upon membrane potential dissipation. Delocalization was observed following treatment with vancoresmycin, comparable to the pore‐forming control nisin, whereas compounds **7** and **8** did not affect the cellular localization of GFP‐MinD (Figure 1B).

Reporter gene assays in *B. subtilis* were employed to further probe the biological profile of the fragments. Compounds were tested in microtiter plate assays using strains expressing β‐galactosidase in response to cell wall biosynthesis inhibition (*P*_ypuA_) or luciferase upon interference with the lipid II cycle (*P*_liaI_). Monitoring over time revealed moderate induction of *P*_ypuA_ by fragments **7**–**9** and the natural product **1** compared to the control antibiotics glycopeptide vancomycin, known to inhibit peptidoglycan biosynthesis, and protein biosynthesis inhibitor clindamycin, used as a negative control (Figure 1C). Vancoresmycin (**1**) significantly induced LiaRS‐mediated stress (Figure 1D), indicative of lipid II cycle‐interfering antibiotics (LIA), as observed before [6]. Interestingly, the western fragment **7** retained this activity, albeit at a much lower level. In addition, formation of membrane blebs was observed in *B. subtilis* for **1**, further substantiating its cell wall‐inhibitory mechanism of action (Figure 1E), whereas no such effect was observed for the synthesized fragments.

**FIGURE 2:**
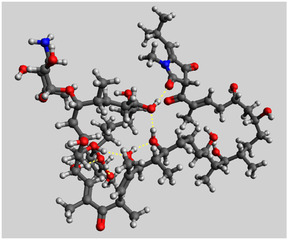
Optimized geometry of the lowest‐energy conformer of vancoresmycin (**1**) on the r^2^scan‐3c level of theory reveals essential H‐bond interaction (yellow dashed lines) between the tetramic acid and the western part of the polyketide backbone [41, 43].

Taken together, the biological data demonstrate that fragmentation of the vancoresmycin scaffold results in a marked reduction or complete loss of antibacterial activity. The molecular basis underlying this effect remains unclear. In particular, it cannot be distinguished whether the inactivity arises from disruption of specific structural interactions or from more general factors such as insufficient membrane interaction, altered amphiphilicity, or conformational variations, all of which may affect direct interaction with the bacterial target. To further elucidate this observation, the 3D structure of vancoresmycin was modeled by first exploring the conformational space using CREST [39], followed by reoptimization of the resulting ensemble with CENSO [40] at the r^2^SCAN‐3c level of theory [41, 43]. The lowest energy conformer is shown in Figure 2. A key intramolecular H─bond interaction between the tetramic acid and the western half of the polyketide backbone appears to lock vancoresmycin (**1**) into a defined conformation. Notably, this key interaction was observed in all calculated conformers. This result suggests that this conformation may be important for its biological activity, consistent with the lack of activity of shorter fragments of vancoresmycin, in which such interactions between the two halves of the molecule are missing. Comparable dependencies on global molecular structure have been reported for other complex natural products, such as abyssomicin C [44].

## 3 Conclusion

In conclusion, a series of structurally defined fragments with all key structural elements of vancoresmycin and amycomycin were synthesized and evaluated with respect to antibacterial activity. Neither the tetramic acid core nor advanced polyketide substructures or the mycosamine unit retained measurable activity when evaluated in isolation. Only the western fragment exhibited weak, cell wall‐associated effects, highlighting the limited functional autonomy of individual substructures and the importance of the biologically relevant conformation. Consistently, 3D modeling indicates that native vancoresmycin adopts a conformation stabilized by a key intramolecular hydrogen bond, which is absent in the fragments and may help maintain a defined structure required for target interaction. Together, these results suggest that vancoresmycin operates as an integrated molecular entity, in which antibacterial activity emerges from the collective interplay of its structural components rather than from isolated motifs. Importantly, they also challenge the assumption that vancoresmycin can be systematically simplified while retaining potent activity, indicating that future (successful) design strategies should preserve key aspects of the overall molecular architecture, particularly amphiphilicity and conformational organization. Further biophysical or structure–activity studies will be required to clarify how these structural features relate to antimicrobial potency.

## Funding

This study was supported by Deutsche Forschungsgemeinschaft (GRK 2873–494832089), Jürgen Manchot Stiftung (stipend to S.M.), Fonds der Chemischen Industrie (stipends to F.H. and A.M.).

## Conflicts of Interest

The authors declare no conflicts of interest.

## Supporting information

Supplementary Material

## Data Availability

The data that support the findings of this study are available in the supplementary material of this article.
