# Supplementary material for: Fragmentation of Vancoresmycin Reveals a Strong Dependence on Global Molecular Architecture for Antibacterial Activity
Source: ChemMedChem. 2026 Jul 30;21(15):e70409. doi: 10.1002/cmdc.70409 (PMC13423584; doi:10.1002/cmdc.70409)
Supplement: Supplementary file 1 — Supplementary Material [file CMDC-21-e70409-s001.pdf]

Supporting Information

Fragmentation of Vancoresmycin Reveals a Strong Dependence on Global Molecular Architecture for Antibacterial Activity

Max Schönenbroicher,[a] Florian Heppner,[a] Maximilian Seul,[a] Simon Morgenschweis,[a] Aia Ali Abdelrahman,[b] Eva Fahr,[b] Niklas Holstein,[b,c] Anna Müller,[b] Dirk Menche*[a]

[a] Kekulé-Institute for Organic Chemistry and Biochemistry
University of Bonn
Gerhard-Domagk-Str. 1, 53121 Bonn, Germany
E-mail: dirk.menche@uni-bonn.de

[b] Institute of Pharmaceutical Microbiology
University of Bonn
Meckenheimer Allee 168, 53115 Bonn, Germany

[c] Niklas Holstein
German Center for Infection Research (DZIF)
Partner site Bonn-Cologne,
Bonn, Germany.

Table of Contents

1. **Material and Methods** ... 3
2. **Synthetic Procedures** ... 5
   - **Synthesis of Tetramic Acid 6** ... 5
   - **Synthesis of Eastern Fragment 8** ... 27
   - **Synthesis of Western Fragment 7** ... 29
   - **Synthesis of Mycosamine 10** ... 79
3. **Biological Evaluation** ... 90
4. **NMR spectra** ... 92
5. **References** ... 230

1. Material and Methods

All reagents were purchased from commercial suppliers (SIGMA ALDRICH, TCI, FISHER SCIENTIFIC, ACROS, ALFA AESAR, ABCR, CARBOLUTION, BLDPHARM) with the highest purity level available and used without further purification unless stated otherwise. Anhydrous solvents (e.g. THF, Et_2O, CH_2Cl_2, DMF, toluene, DCM, MeCN) were obtained from a solvent drying system MB SPS-800 (MBRAUN) and stored over molecular sieves (4 Å) or were purchased from FISHER SCIENTIFIC. Solvents for flash column chromatography were purified by distillation over a Vigreux column at standard pressure (DCM, cyclohexane) or over a packed 1 m column (ethyl acetate).

Reactions, in which dry solvents were used, were performed under an argon atmosphere in flame-dried glassware, which had been flushed with argon unless stated otherwise. The reagents were handled using standard Schlenk techniques. For heating over room temperature, heat-on attachments (HEIDOLPH) were used controlling the temperature by a temperature modulator. For cooling, the following baths were used: acetone/dry ice (–78 °C), acetonitrile/dry ice (–40 °C), water/ice (0 °C). Temperatures between –78 °C and 0 °C were obtained with the immersion cooler HUBER TC 100E-F-NR.

For TLC, silica gel 60_{F254} pre-coated polyester sheets (0.2 mm silica gel, MACHEREY-NAGEL) were used, whereas silica gel 60 RP-18 $F_{254}S$ pre-coated aluminum sheets (MERCK) were used for reversed phase TLC. Detection was achieved with short-wave UV-light (254 nm & 366 nm) and staining with a solution of CAM (1.0 g $Ce(SO_4)_2$, 2.5 g $(NH_4)_6Mo_7O_{24}$, 8.0 mL conc. H_2SO_4 in 100 mL water) and subsequent heating.

For flash column chromatography, silica gel (pore size 60 Å, 35 – 70 µmol) purchased from MERCK or SIGMA ALDRICH was used. For reversed phase column chromatography C18-reversed phase silica gel (pore size 90 Å) from SIGMA ALDRICH or CARL ROTH was used. Compounds were eluted using the stated mixtures under a positive pressure of air. Solvents used for column chromatography were distilled prior to use.

Semi-preparative and analytical HPLC-measurements were performed on systems of the company KNAUER WISSENSCHAFTLICHE GERÄTE GMBH. The solvents for HPLC were purchased in HPLC grade. The chromatograms were recorded by UV-detection.

All NMR-spectra were recorded at 298 K at the analytical department of the Chemical Institutes of the University of Bonn. The following spectrometers of BRUKER were used:

AV I 300, AV I 400, AV I 500, AV III HD 500, AV III HD 700 Cryo. The spectra were measured in deuterated solvents from DEUTERO or CARL ROTH. Due to the repeated occurrence of acidic side-reactions in $CDCl_3$, CD_2Cl_2 was predominantly employed. Although these side reactions could be suppressed by pre-drying of $CDCl_3$ over 5 Å molecular sieves, CD_2Cl_2 was chosen as the primary solvent to minimize decomposition, especially for elaborated fragments. Chemical shifts (δ) are reported in ppm relative to tetramethylsilane (δ = 0.00 ppm) and were calibrated to the residual signal of undeuterated solvents. Data for ^{1}H-NMR are reported as follows: chemical shift (multiplicity, number of hydrogens, coupling constant in Hz, annotation). The following abbreviations were used: s = singlet, d = doublet, t = triplet, q = quartet, dd = doublet of doublet, dddd = doublet of doublet of doublet of doublet, quint = quintet, td = triplet of doublet, tq = triplet of quartet, qd = quartet of doublet, m = multiplet, br = broad. Structural assignments were made with additional information from gNOESY, gCOSY, gHSQC, and gHMBC experiments. For the smaller compounds, the assignments are straightforward based on the molecular structures. For all remaining compounds, peak assignment was made by analogy to the corresponding key compounds.

All mass-spectra were recorded on THERMO FISHER SCIENTIFIC Orbitrap XL, BRUKER DALTONIK micrOTOF-Q or BRUKER DALTONIK Apex IV FT-ICR at the analytical department of the Chemical Institutes of the University of Bonn. Optical rotation values were recorded on ANTON PAAR MCP 150 at 20 °C with a 10 mm cuvette using a LED (589 nm) as light source.

2. Synthetic Procedures

Synthesis of Tetramic Acid 6

Synthesis of compound 12

(*Z*)-4-Methoxy-5-(2-methylpropylidene)-1,5-dihydro-2*H*-pyrrol-2-one

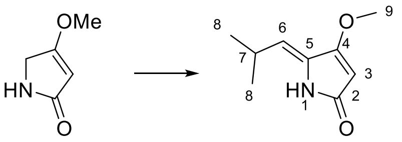

11 **12**
$C_9H_{13}NO_2$
167.21 g/mol

4-Methoxy-3-pyrrolin-2-one (**11**, 2.64 g, 23.4 mmol, 1.0 eq.) was dissolved in NaOH (1.0 M in H_2O, 26 mL) and freshly distilled isobutyraldehyde (2.13 mL, 24.1 mmol, 1.03 eq.) was added. The initial homogeneous reaction mixture was stirred at 60 °C for 7 h. After cooling to room temperature, the white precipitate was filtered off and washed with ice-cold water. It was dissolved in DCM, dried over Na_2SO_4 and the solvent was removed under reduced pressure, yielding product **12** as a colorless amorphous solid (3.90 g, 23.3 mmol, 99%, *Z*/*E* = 11:1).

***Z*-Isomer**

R*f*: 0.15 (cyclohexane/ethyl acetate 1:1); **^{1}H-NMR** (500 MHz, CD_2Cl_2, 298 K): δ [ppm] = 8.06 (s, 1H, NH), 5.26 (d, 1H, $^3J_{6,7}$ = 9.9 Hz, H-6), 5.08 (d, 1H, $^5J_{3,6}$ = 1.8 Hz, H-3), 3.82 (s, 3H, H-9), 2.59 (dhept, 1H, $^3J_{6,7}$ = 9.9 Hz, $^3J_{7,8}$ = 6.7 Hz, H-7), 1.08 (d, 6H, $^3J_{7,8}$ = 6.7 Hz, H-8); **^{13}C-NMR** (126 MHz, CD_2Cl_2, 298 K): δ [ppm] = 171.8 (C-2), 167.0 (C-4), 131.7 (C-5), 117.1 (C-6), 93.0 (C-3), 58.5 (C-9), 27.5 (C-7), 22.8 (2C, C-8); **HRMS (ESI+)** *m*/*z*: $[M+H]^+$ calcd. for $C_9H_{13}NO_2H^+$ 168.1019, found 168.1020.

***E*-Isomer**

R*f*: 0.10 (cyclohexane/ethyl acetate 1:1); **^{1}H-NMR** (500 MHz, CD_2Cl_2, 298 K): δ [ppm] = 7.64 (s, 1H, NH), 5.21 (dd, 1H, $^3J_{6,7}$ = 10.2 Hz, $^5J_{3,6}$ = 1.4 Hz, H-6), 5.16 (d, 1H, $^5J_{3,6}$ = 1.4 Hz, H-3), 3.99 (s, 3H, H-9), 3.27 (dhept, 1H, $^3J_{6,7}$ = 10.2 Hz, $^3J_{7,8}$ = 6.6 Hz, H-7), 1.03 (d, 6H, $^3J_{7,8}$ = 6.6 Hz, H-8); **^{13}C-NMR** (126 MHz, CD_2Cl_2, 298 K): δ [ppm] = 170.7 (C-2), 167.8 (C-4), 131.0 (C-5), 123.4 (C-6), 95.5 (C-3), 58.5 (C-9), 26.4 (C-7), 23.6 (2C, C-8); **HRMS (ESI+)** *m*/*z*: $[M+H]^+$ calcd. for $C_9H_{13}NO_2H^+$ 168.1019, found 168.1020.

Synthesis of compound 13

(*Z*)-5-(2-Methylpropylidene)pyrrolidine-2,4-dione

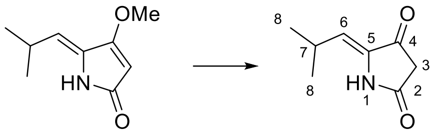

12

13
$C_8H_{11}NO_2$
153.18 g/mol

Compound **12** (112 mg, 670 µmol, 1.0 eq.) was dissolved in concentrated HCl (3.0 mL) and stirred for 7 h at room temperature. Afterwards, water (20 mL) and DCM (20 mL) were added at 0 °C and the phases were separated. The aqueous phase was extracted with DCM (3 × 20 mL), the combined organic phases were dried over $MgSO_4$ and all volatiles were removed under reduced pressure. The product **13** was obtained as a colorless amorphous solid (102 mg, 670 µmol, quant, *Z*/*E* = 18:1).

R_f: 0.28 (cyclohexane/ethyl acetate 1:1); **^{1}H-NMR** (500 MHz, CD_2Cl_2, 298 K): δ [ppm] = 8.29 (s, 1H, NH), 5.50 (d, 1H, $^3J_{6,7}$ = 9.9 Hz, H-6), 3.05 (s, 2H, H-3), 2.43 (dhept, 1H, $^3J_{6,7}$ = 9.9 Hz, $^3J_{7,8}$ = 6.7 Hz, H-7), 1.09 (d, 6H, $^3J_{7,8}$ = 6.7 Hz, H-8); **^{13}C-NMR** (126 MHz, CD_2Cl_2, 298 K): δ [ppm] = 193.7 (C-4), 170.1 (C-2), 134.2 (C-5), 117.7 (C-6), 40.9 (C-3), 27.3 (C-7), 22.2 (2C, C-8); **HRMS (APCI)** *m*/*z*: $[M+H]^+$ calcd. for $C_8H_{11}NO_2H^+$ 154.0863, found 154.0861.

Synthesis of compound 14

(*Z*)-Ethyl (2-(2-methylpropylidene)-5-oxo-2,5-dihydro-1*H*-pyrrol-3-yl) carbonate

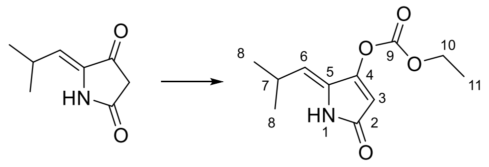

13 **14**

$C_{11}H_{15}NO_4$

225.24 g/mol

NEt_3 (2.56 mL, 18.5 mmol, 1.4 eq.) and ethyl chloroformate (1.44 mL, 15.2 mmol, 1.15 eq.) were added dropwise to compound **13** (2.02 g, 13.2 mmol, 1.0 eq.) in dry DCM (25 mL) at 0 °C. After stirring for 80 min at 0 °C, the yellow solution was diluted with water (15 mL) and $NaHCO_3$-solution (aq., sat., 15 mL) and the phases were separated. The aqueous phase was extracted with DCM (4 × 50 mL), the combined organic layers were dried over Na_2SO_4 and all volatiles were removed under reduced pressure. The crude product was purified by flash column chromatography (SiO_2, cyclohexane/ethyl acetate 6:4), yielding product **14** as an orange amorphous solid (2.50 mg, 11.1 mmol, 84%).

R*f*: 0.35 (cyclohexane/ethyl acetate 1:1); **^{1}H-NMR** (500 MHz, CD_2Cl_2, 298 K): δ [ppm] = 7.82 (s, 1H, NH), 6.03 (dd, 1H, $^4J_{3,NH}$ = 1.9 Hz, $^5J_{3,6}$ = 0.6 Hz, H-3), 5.37 (dd, 1H, $^3J_{6,7}$ = 10.0 Hz, $^5J_{3,6}$ = 0.6 Hz, H-6), 4.34 (q, 2H, $^3J_{10,11}$ = 7.1 Hz, H-10), 2.59 (dhept, 1H, $^3J_{6,7}$ = 10.0 Hz, $^3J_{7,8}$ = 6.7 Hz, H-7), 1.37 (t, 3H, $^3J_{10,11}$ = 7.1 Hz, H-11), 1.10 (d, 6H, $^3J_{7,8}$ = 6.7 Hz, H-8); **^{13}C-NMR** (126 MHz, CD_2Cl_2, 298 K): δ [ppm] = 170.2 (C-2), 155.6 (C-4), 151.1 (C-9), 130.9 (C-5), 119.1 (C-6), 105.0 (C-3), 66.2 (C-10), 27.8 (C-7), 22.7 (2C, C-8), 14.3 (C-11); **HRMS (ESI+)** *m*/*z*: $[M+H]^+$ calcd. for $C_{11}H_{15}NO_4H^+$ 226.1074, found 226.1077.

Synthesis of compound 15

Ethyl (*Z*)-4-hydroxy-5-(2-methylpropylidene)-2-oxo-2,5-dihydro-1*H*-pyrrole-3-carboxylate

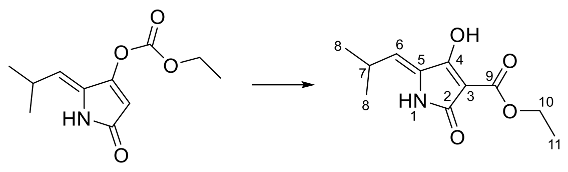

14 **15**

$C_{11}H_{15}NO_4$

225.24 g/mol

NEt_3 (620 µL, 4.47 mmol, 1.4 eq.), $CaCl_2$ (540 mg, 4.87 mmol, 1.5 eq.) and DMAP (125 mg, 1.02 mmol, 0.32 eq.) were added to the yellow solution of compound **14** (725 mg, 3.22 mmol, 1.0 eq.) in dry DCM (25 mL). After stirring for 17 h at room temperature, HCl (0.5 M in H_2O, 40 mL) was added to the orange suspension and the phases were separated. The aqueous phase was extracted with DCM (10 × 40 mL) and the combined organic layers were washed with water (350 mL). Drying over Na_2SO_4 and concentration *in vacuo* yielded product **15** as ochre-colored amorphous solid (697 mg, 3.09 mmol, 96%, *Z*/*E* > 20:1).

R*f*: 0.12 (cyclohexane/ethyl acetate 1:1); **^{1}H-NMR** (700 MHz, CD_2Cl_2, 298 K): δ [ppm] = 10.97 (br s, 1H, H-OH), 7.16 (s, 1H, NH), 5.66 (d, 1H, $^3J_{6,7}$ = 9.9 Hz, H-6), 4.37 (q, 2H, $^3J_{10,11}$ = 7.1 Hz, H-10), 2.58 (dhept, 1H, $^3J_{6,7}$ = 9.9 Hz, $^3J_{7,8}$ = 6.7 Hz, H-7), 1.37 (t, 3H, $^3J_{10,11}$ = 7.1 Hz, H-11), 1.12 (d, 6H, $^3J_{7,8}$ = 6.7 Hz, H-8); **^{13}C-NMR** (176 MHz, CD_2Cl_2, 298 K): δ [ppm] = 174.5 (C-4), 167.8 (C-9), 165.4 (C-2), 129.1 (C-5), 122.3 (C-6), 97.3 (C-3), 61.7 (C-10), 27.9 (C-7), 22.4 (2C, C-8), 14.4 (C-11); **HRMS (ESI+)** *m*/*z*: $[M+H]^+$ calcd. for $C_{11}H_{15}NO_4H^+$ 226.1074, found 226.1074.

Synthesis of compound 17

Ethyl (*Z*)-4-hydroxy-1-methyl-5-(2-methylpropylidene)-2-oxo-2,5-dihydro-1*H*-pyrrole-3-carboxylate

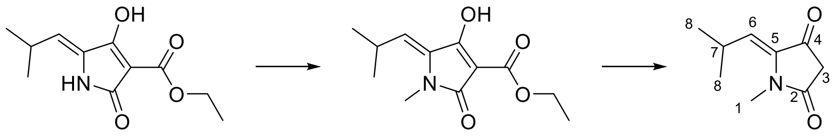

15 **16** **17**

$C_9H_{13}NO_2$
167.21 g/mol

NaHMDS (1.0 M in THF, 8.00 mL, 8.00 mmol, 2.6 eq.) was added dropwise in 5 min to an orange suspension of compound **15** (695 mg, 3.09 mmol, 1.0 eq.) in dry THF (45 mL) at –78 °C. After stirring for 60 min at –78 °C, MeI (2.00 mL, 32.1 mmol, 10.4 eq.) was added to the clear, dark red solution. After warming to –40 °C, stirring continued for 14 h. H_2O (25 mL) and citric acid (0.5 M in H_2O, 20 mL) were added and the phases were separated. The aqueous phase was extracted with EtOAc (6 × 80 mL) and the combined organic layers were dried over Na_2SO_4. After concentration *in vacuo*, product **16** was obtained as an orange oil and redissolved in EtOAc (20 mL). H_2O (0.2 mL) was added and the reaction mixture was refluxed for 3 h. After cooling to room temperature, water (20 mL) was added and the phases separated. The aqueous phase was extracted with EtOAc (3 × 30 mL) and the combined organic layers were dried over Na_2SO_4. After concentration *in vacuo* and flash column chromatography (SiO_2, cyclohexane/ethyl acetate 5:2 → 4:6) yielded product **17** as a yellow amorphous solid (264 mg, 1.58 mmol, 51% over 2 steps).

R_f: 0.36 (cyclohexane/ethyl acetate 1:1); **^{1}H-NMR** (700 MHz, CD_2Cl_2, 298 K): δ [ppm] = 5.48 (d, 1H, $^3J_{6,7}$ = 10.9 Hz, H-6), 3.34 (s, 3H, H-1), 3.01 (dhept, 1H, $^3J_{6,7}$ = 10.9 Hz, $^3J_{7,8}$ = 6.6 Hz, H-7), 2.98 (s, 2H, H-3), 1.10 (d, 6H, $^3J_{7,8}$ = 6.6 Hz, H-8); **^{13}C-NMR** (176 MHz, CD_2Cl_2, 298 K): δ [ppm] = 193.9 (C-4), 169.1 (C-2), 135.2 (C-5), 118.8 (C-6), 39.5 (C-3), 28.7 (C-1), 25.4 (C-7), 23.9 (2C, C-8); **HRMS (ESI+)** *m/z*: $[M+H]^+$ calcd. for $C_9H_{13}NO_2H^+$ 168.1019, found 168.1020.

Synthesis of compound 21

(*S*)-2-Methyl-5-oxohexanal

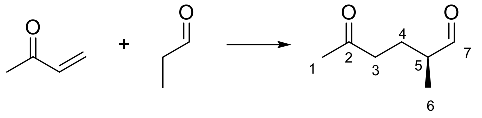

18 **19** **21**

$C_7H_{12}O_2$
128.17 g/mol

Freshly distilled methyl vinyl ketone (**18**, 17.9 mL, 212 mmol, 3.0 eq.) and propionaldehyde (**19**, 5.10 mL, 70.7 mmol, 1.0 eq.) were added to organocatalyst **20**[1] (3.45 g, 14.1 mmol, 0.2 eq.) and ethyl 3,4-dihydroxybenzoate (2.57 g, 14.1 mmol, 0.2 eq.). The reaction mixture was stirred for 16 h at room temperature and afterwards, directly purified by flash column chromatography (SiO_2, pentane/diethyl ether 8:2 → 7:3 → 6:4). Product **21** was obtained as a colorless oil (6.13 g, 47.8 mmol, 68%, *e.r.* = 92:8).

R*f*: 0.15 (*n*-pentane/diethyl ether 7:3); **$[\alpha]_D^{20}$** = +36.1° (c = 1.22, CH_2Cl_2); **^{1}H-NMR** (500 MHz, CD_2Cl_2, 298 K): 9.58 (d, 1H, $^3J_{5,7}$ = 1.7 Hz, H-7), 2.47 (t, 2H, $^3J_{3,4}$ = 7.5 Hz, H-3), 2.33 (qd, 1H, $^3J_{5,6}$ = 7.0 Hz, $^3J_{5,7}$ = 1.7 Hz, H-5), 2.10 (s, 3H, H-1), 1.97 – 1.88 (m, 1H, H-4a), 1.65 – 1.56 (m, 1H, H-4b), 1.08 (d, 3H, $^3J_{5,6}$ = 7.0 Hz, H-6); **^{13}C-NMR** (126 MHz, CD_2Cl_2, 298 K): 208.0 (C-2), 204.8 (C-7), 45.9 (C-5), 40.8 (C-3), 30.1 (C-1), 24.4 (C-4), 13.6 (C-6); **HRMS (ESI+)** *m*/*z*: [M+H]$^+$ calcd. for $C_7H_{13}O_2^+$ 129.0911, found 129.0911.

Synthesis of compound 22

(5*S*)-6-((*tert*-Butyldimethylsilyl)oxy)-5-methylhexan-2-ol

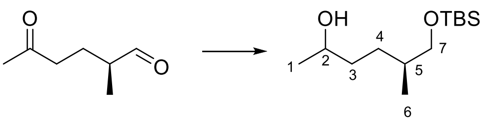

21 **22**

$C_{13}H_{30}O_2Si$
246.47 g/mol

$LiAlH_4$ (4.0 M in Et_2O, 6.26 mL, 25.1 mmol, 2.0 eq.) was added to dry Et_2O (60 mL) at 0 °C. Afterwards, ketoaldehyde **21** (1.61 g, 12.6 mmol, 1.0 eq.) in Et_2O (20 mL) was added dropwise and the reaction mixture was allowed to warm up to room temperature. After stirring for 25 min, the mixture was carefully quenched with water (60 mL) and Rochelle-salt-solution (aq., sat., 30 mL) was added. The phases were separated and the aqueous phase was extracted with Et_2O (3 × 100 mL). The combined organic layers were dried over Na_2SO_4 and the solvent was removed under reduced pressure. To the crude diol (max. 12.6 mmol), dissolved in dry DCM (40 mL), were added at 0 °C imidazole (1.28 g, 18.8 mmol, 1.5 eq.) and TBSCl (1.80 g, 11.9 mmol, 0.95 eq.), dissolved in dry DCM (7 mL). The reaction mixture was stirred for 45 min at room temperature. Then, NH_4Cl-solution (aq., sat., 40 mL) was added and the different layers were separated. The aqueous phase was extracted with DCM (3 × 40 mL) and Et_2O (3 × 40 mL), the combined organic phases were dried over $MgSO_4$ and the solvents were removed under reduced pressure. Product **22** was obtained after flash column chromatography (SiO_2, *n*-pentane:Et_2O 100:0 → 70:30) as a colorless oil (1.95 g, 7.91 mmol, 63%).

R_f: 0.15 (*n*-pentane/diethyl ether 7:3); **^{1}H-NMR** (700 MHz, CD_2Cl_2, 298 K): δ [ppm] = 3.75 – 3.70 (m, 1H, H-2), 3.45 (ddd, 1H, $^2J_{7a,7b}$ = 9.8 Hz, $^3J_{6,7a}$ = 6.0 Hz, $^4J_{4,7a}$ = 1.7 Hz, H-7a), 3.46 – 3.37 (m, 1H, H-7b), 1.59 – 1.53 (m, 1H, H-5), 1.50 – 1.37 (m, 4H, H-3, H-4), 1.15 (2 x d, 3H, $^3J_{1,2}$ = 6.2 Hz, H-1), 0.89 (s, 9H, TBS), 0.88 (2 x d, 3H, $^3J_{5,6}$ = 6.7 Hz, H-6), 0.04 (s, 3H, TBS), 0.04 (s, 3H, TBS); **^{13}C-NMR** (176 MHz, CD_2Cl_2, 298 K): δ [ppm] = 68.7/68.6 (C-2), 68.6/68.5 (C-7), 37.2/37.2 (C-3), 36.3/36.2 (C-5), 29.6 (C-4), 26.1 (3C, TBS), 23.7/23.6 (C-1), 18.6 (TBS), 16.9 (C-6), -5.3 (2C, TBS); **HRMS** (**APCI**) *m*/*z*: $[M+H]^+$ calcd. for $C_{13}H_{30}O_2SiH^+$ 247.2088, found 247.2083.

Synthesis of compound 23

(*S*)-6-((*tert*-Butyldimethylsilyl)oxy)-5-methylhexan-2-one

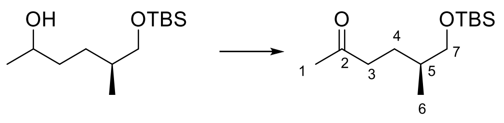

22 **23**

$C_{13}H_{28}O_2Si$
244.45 g/mol

DIPEA (6.00 mL, 35.3 mmol, 4.0 eq.) and DMSO (6.50 mL, 91.5 mmol, 10.5 eq.) were added to py·SO_3 (4.39 g, 27.6 mmol, 3.2 eq.) in dry DCM (70 mL). After cooling to 0 °C, alcohol **22** (2.15 g, 8.72 mmol, 1.0 eq.) in dry DCM (30 mL) was added dropwise and the reaction mixture was stirred for 90 min at 0 °C. After addition of NH_4Cl-solution (aq., sat., 100 mL), the phases were separated. The aqueous phase was extracted with DCM (3 × 100 mL), the combined organic layers were dried over $MgSO_4$ and the solvent was removed under reduced pressure. The crude product was purified by flash column chromatography (SiO_2, cyclohexane/ethyl acetate 9:1 → 7:3) and product **23** was obtained as a colorless oil (2.05 g, 8.39 mmol, 96%).

R*f*: 0.55 (cyclohexane/ethyl acetate 2:1); **$[\alpha]_D^{20}$** = -4.4° (c = 1.13, CH_2Cl_2); **^{1}H-NMR** (500 MHz, CD_2Cl_2, 298 K): *δ* [ppm] = 3.45 – 3.39 (m, 2H, H-7), 2.49 – 2.37 (m, 2H, H-3), 2.10 (s, 3H, H-1), 1.67 (dddd, 1H, $^2J_{4a,4b}$ = 13.5 Hz, $^3J_{3a,4a}$ = 9.4 Hz, $^3J_{4a,5}$ = 6.2 Hz, $^3J_{3b,4a}$ = 5.6 Hz, H-4a), 1.59 – 1.52 (m, 1H, H-5), 1.34 (dddd, 1H, $^2J_{4a,4b}$ = 13.5 Hz, $^3J_{3a,4b}$ = 9.4 Hz, $^3J_{4b,5}$ = 7.7 Hz, $^3J_{3b,4b}$ = 6.0 Hz, H-4b), 0.89 (s, 9H, H-9), 0.86 (d, 3H, $^3J_{5,6}$ = 6.8 Hz, H-6), 0.04 (s, 6H, H-8); **^{13}C-NMR** (126 MHz, CD_2Cl_2, 298 K): *δ* [ppm] = 209.2 (C-2), 68.4 (C-7), 41.7 (C-3), 35.7 (C-5), 29.9 (C-1), 27.7 (C-4), 26.1 (3C, C-9), 18.6 (C-10), 16.7 (C-6), -5.3 (2C, C-8); **HRMS (ESI+)** *m/z*: $[M+H]^+$ calcd. for $C_{13}H_{28}O_2SiH^+$ 245.1931, found 245.1930.

Synthesis of compound 25

(2*S*,7*S*,*E*)-1-((*tert*-Butyldimethylsilyl)oxy)-7-hydroxy-11-((4-methoxybenzyl)oxy)-2,10-dimethylundec-9-en-5-one

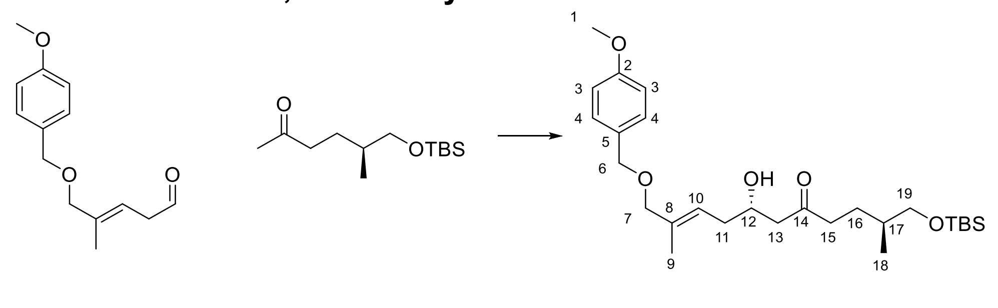

24 **23** **25**

$C_{27}H_{46}O_5Si$
478.75 g/mol

Aldehyde **24**[2] (427 mg, 1.82 mmol, 1.1 eq.) and ketone **23** (405 mg, 1.66 mmol, 1.0 eq.) were both dried overnight in dry DCM (10.0 mL and 4.0 mL) over 3 Å MS. Triethylamine (dried over 3 Å MS, 460 µL, 3.31 mmol, 2.0 eq.) and ketone **23** were

added to (–)-Ipc_2BCl (632 mg, 1.97 mmol, 1.2 eq.) in dry DCM (8.0 mL) at –78 °C. After stirring for 3 h at –78 °C, aldehyde **24** was added dropwise to the white-opaque suspension and stirring at –78 °C continued for 1 h. Afterwards, the reaction suspension was allowed to warm up to –15 °C and stirred for 17 h. MeOH (16 mL), pH 7 buffer (16 mL) and H_2O_2 (35%, 8 mL) were added and the reaction mixture was stirred for another 1.5 h at room temperature. The phases were separated and the aqueous phase was extracted with DCM (3 × 50 mL). The combined organic phases were dried over Na_2SO_4 and the solvents were removed under reduced pressure. The crude product was purified by flash column chromatography (SiO_2, cyclohexane/ethyl acetate 3:1) and product **25** was obtained as a yellow oil (433 mg, 904 µmol, 55%, *d.r.* = 3:1).

R_f: 0.18 (cyclohexane/ethyl acetate 4:1); **$[\alpha]_D^{20}$** = +3.8° (c = 0.79, CH_2Cl_2); **^{1}H-NMR** (700 MHz, CD_2Cl_2, 298 K): δ [ppm] = 7.25 (d, 2H, $^3J_{3,4}$ = 8.7 Hz, H-4), 6.87 (d, 2H, $^3J_{3,4}$ = 8.7 Hz, H-3), 5.47 (tq, 1H, $^3J_{10,11}$ = 7.4 Hz, $^4J_{9,10}$ = 1.4 Hz, H-10), 4.36 (s, 2H, H-6), 4.06 (dtd, 1H, $^3J_{12,13b}$ = 9.2 Hz, $^3J_{11,12}$ = 6.3 Hz, $^3J_{12,13a}$ = 2.8 Hz, H-12), 3.88 (d, 2H, $^4J_{7,9}$ = 0.8 Hz, H-7), 3.79 (s, 3H, H-1), 3.44 – 3.39 (m, 2H, H-19), 2.90 (br s, 1H, OH), 2.61 (dd, 1H, $^3J_{13a,13b}$ = 17.3 Hz, $^3J_{12,13a}$ = 2.8 Hz, H-13a), 2.52 – 2.41 (m, 3H, H-13b, H-15), 2.27 – 2.17 (m, 2H, H-11), 1.70 – 1.65 (m, 1H, H-16a), 1.67 (d, 3H, $^4J_{9,10}$ = 1.4 Hz, H-9), 1.58 – 1.53 (m, 1H, H-17), 1.38 – 1.32 (m, 1H, H-16b), 0.89 (s, 9H, TBS), 0.86 (d, 3H, $^3J_{17,18}$ = 6.7 Hz, H-18), 0.04 (s, 6H, TBS); **^{13}C-NMR** (176 MHz, CD_2Cl_2, 298 K): δ [ppm] = 212.4 (C-14), 159.6 (C-2), 135.6 (C-8), 131.2 (C-5), 129.7 (2C, C-4), 123.3 (C-10), 114.0 (2C, C-3), 76.1 (C-7), 71.7 (C-6), 68.4 (C-19), 68.0 (C-12), 55.6 (C-1), 48.7 (C-13), 41.7 (C-15), 35.7 (C-17), 35.2 (C-11), 27.4 (C-16), 26.1 (3C, TBS), 18.6 (TBS), 16.7 (C-18), 14.3 (C-9), -5.3 (TBS), -5.3 (TBS); **HRMS (ESI+)** *m*/*z*: $[M+H]^+$ calcd. for $C_{27}H_{46}O_5SiH^+$ 479.3187, found 479.3186.

Synthesis of compound S-1

(5*S*,10*S*,*E*)-11-((*tert*-Butyldimethylsilyl)oxy)-1-((4-methoxybenzyl)oxy)-2,10-dimethyl-7-oxoundec-2-en-5-yl (*S*)-3,3,3-trifluoro-2-methoxy-2-phenylpropanoate

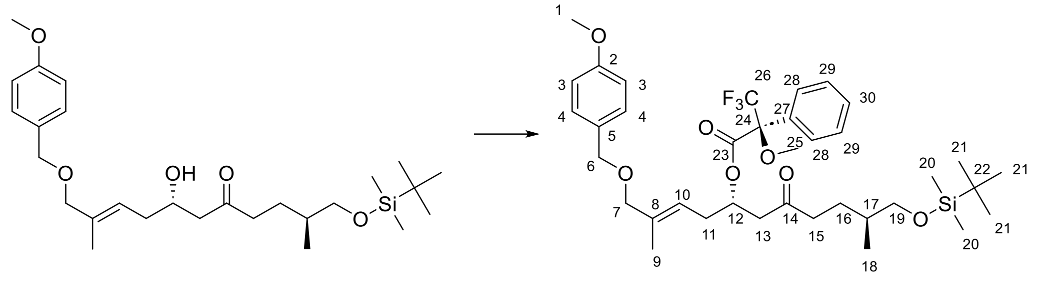

25

S-1
$C_{37}H_{53}F_3O_7Si$
694.90 g/mol

Pyridine (3.50 µL, 43.5 µmol, 3.8 eq.) and (*R*)-MTPA-Cl (5.00 µL, 26.7 µmol, 2.3 eq.) were added to hydroxyketone **25** (5.50 mg, 11.5 µmol, 1.0 eq.) in dry DCM (0.5 mL). After stirring for 5 h at room temperature, the reaction was stopped by the addition of water (0.5 mL). The phases were separated and the aqueous phase was extracted with DCM (3 × 0.5 mL). The combined organic phases were dried over $MgSO_4$ and the solvent was removed under reduced pressure. Purification by flash column chromatography (SiO_2, cyclohexane/ethyl acetate 5:1) gave MOSHER ester **S-1** (8.0 mg, 11.5 µmol, quant.) as a colorless oil, which still contained MOSHER acid.

R*f*: 0.28 (cyclohexane/ethyl acetate 4:1); **[α]$_D^{20}$** = -55.4° (c = 0.44, CH_2Cl_2); **^{1}H-NMR** (700 MHz, CD_2Cl_2, 298 K): *δ* [ppm] = 7.48 – 7.44 (m, 2H, H-28), 7.41 – 7.38 (m, 3H, H-29, H-30), 7.23 (d, 2H, $^3J_{3,4}$ = 8.7 Hz, H-4), 6.87 (d, 2H, $^3J_{3,4}$ = 8.7 Hz, H-3), 5.55 (dtd, 1H, $^3J_{12,13a}$ = 8.5 Hz, $^3J_{11,12}$ = 6.1 Hz, $^3J_{12,13b}$ = 4.3 Hz, H-12), 5.29 (ddt, 1H, $^3J_{10,11a}$ = 7.3 Hz, $^3J_{10,11b}$ = 6.0 Hz, $^4J_{9,10}$ = 1.4 Hz, H-10), 4.33 (s, 2H, H-6), 3.80 (s, 2H, H-7), 3.79 (s, 3H, H-1), 3.47 (d, 3H, H-25), 3.41 (dd, 2H, $^3J_{17,19}$ = 6.7 Hz, $^4J_{16,19}$ = 1.9 Hz, H-19), 2.83 (dd, 1H, $^2J_{13a,13b}$ = 17.4 Hz, $^3J_{12,13a}$ = 8.5 Hz, H-13a), 2.65 (dd, 1H, $^2J_{13a,13b}$ = 17.4 Hz, $^3J_{12,13b}$ = 4.3 Hz, H-13b), 2.53 – 2.42 (m, 2H, H-11a, H-15a), 2.39 – 2.30 (m, 2H, H-11b, H-15b), 1.69 – 1.63 (m, 1H, H-16a), 1.61 (d, 3H, $^4J_{9,10}$ = 1.4 Hz, H-9), 1.60 – 1.47 (m, 1H, H-17), 1.35 – 1.30 (m, 1H, H-16b), 0.89 (s, 9H, H-21), 0.84 (d, 3H, $^3J_{17,18}$ = 6.7 Hz, H-18), 0.03 (s, 6H, H-20); **^{13}C-NMR** (176 MHz, CD_2Cl_2, 298 K): *δ* [ppm] = 207.6 (C-14), 166.2 (C-23), 159.6 (C-2), 137.1 (C-8), 132.8 (C-27), 131.1 (C-5), 130.0 (C-30), 129.2 (2C, C-4), 128.7 (2C, C-29), 127.3 (2C, C-28),

123.2 (q, 1C, $^{1}J_{C,F}$ = 289 Hz, C-26), 120.7 (C-10), 114.0 (2C, C-3), 85.4 (q, 1C, $^{2}J_{C,F}$ = 28.2 Hz, C-24), 75.8 (C-7), 72.7 (C-12), 71.8 (C-6), 68.3 (C-19), 56.3 (q, $^{4}J_{C,F}$ = 1.7 Hz, C-25), 55.6 (C-1), 45.8 (C-13), 41.5 (C-15), 35.6 (C-17), 32.1 (C-11), 27.3 (C-16), 26.1 (3C, C-21), 18.6 (C-22), 16.6 (C-18), 14.2 (C-9), -5.3 (C-20), -5.3 (C-20); **HRMS (ESI+)** *m/z*: $[M+NH_4]^+$ calcd. for $C_{37}H_{53}F_3O_7SiNH_4^+$ 712.3851, found 712.3859.

Synthesis of compound S-2

(5*S*,10*S*,*E*)-11-((*tert*-Butyldimethylsilyl)oxy)-1-((4-methoxybenzyl)oxy)-2,10-dimethyl-7-oxoundec-2-en-5-yl (*R*)-3,3,3-trifluoro-2-methoxy-2-phenylpropanoate

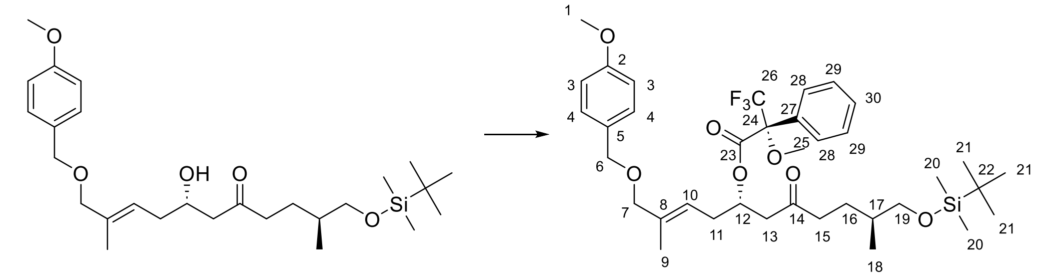

25 **S-2**

$C_{37}H_{53}F_3O_7Si$

694.90 g/mol

Pyridine (3.50 µL, 43.5 µmol, 3.8 eq.) and (*S*)-MTPA-Cl (5.00 µL, 26.7 µmol, 2.3 eq.) were added to hydroxyketone **25** (5.50 mg, 11.5 µmol, 1.0 eq.) in dry DCM (0.5 mL). After stirring for 5.5 h at room temperature, the reaction was stopped by the addition of water (0.5 mL). The phases were separated and the aqueous phase was extracted with DCM (3 × 0.5 mL). The combined organic phases were dried over $MgSO_4$ and the solvent was removed under reduced pressure. Purification by flash column chromatography (SiO_2, cyclohexane/ethyl acetate 5:1) gave MOSHER ester **S-2** (8.00 mg, 11.5 µmol, quant.) as colorless oil, which still contained MOSHER acid.

R_f: 0.26 (cyclohexane/ethyl acetate 4:1); **$[\alpha]_D^{20}$** = +41.0° (c = 1.00, CH_2Cl_2); **^{1}H-NMR** (700 MHz, CD_2Cl_2, 298 K): *δ* [ppm] = 7.48 – 7.44 (m, 2H, H-28), 7.41 – 7.38 (m, 3H, H-29, H-30), 7.23 (d, 2H, $^{3}J_{3,4}$ = 8.7 Hz, H-4), 6.86 (d, 2H, $^{3}J_{3,4}$ = 8.7 Hz, H-3), 5.58 (dtd, 1H, $^{3}J_{12,13a}$ = 8.1 Hz, $^{3}J_{11,12}$ = 6.2 Hz, $^{3}J_{12,13b}$ = 4.5 Hz, H-12), 5.45 – 5.42 (m, 1H, H-10), 4.36 (d, 1H, $^{2}J_{6a,6b}$ = 11.4 Hz, H-6a), 4.34 (d, 1H, $^{2}J_{6a,6b}$ = 11.4 Hz, H-6b), 3.86 (s, 2H, H-7), 3.79 (s, 3H, H-1), 3.49 (s, 3H, H-25), 3.38 (dd, 2H, $^{3}J_{17,19}$ = 6.0 Hz,

$^{4}J_{16,19}$ = 1.8 Hz, H-19), 2.78 (dd, 1H, $^{2}J_{13a,13b}$ = 17.2 Hz, $^{3}J_{12,13a}$ = 8.1 Hz, H-13a), 2.59 (dd, 1H, $^{2}J_{13a,13b}$ = 17.2 Hz, $^{3}J_{12,13b}$ = 4.5 Hz, H-13b), 2.52 – 2.35 (m, 3H, H-11, H-15a), 2.26 (ddd, 1H, $^{2}J_{15a,15b}$ = 16.9 Hz, $^{3}J_{15b,16}$ = 9.9 Hz, $^{3}J_{15b,16}$ = 5.7 Hz, H-15b), 1.66 (d, 3H, $^{4}J_{9,10}$ = 1.4 Hz, H-9), 1.60 – 1.56 (m, 1H, H-16a), 1.54 – 1.47 (m, 1H, H-17), 1.28 – 1.23 (m, 1H, H-16b), 0.89 (s, 9H, H-21), 0.82 (d, 3H, $^{3}J_{17,18}$ = 6.8 Hz, H-18), 0.03 (s, 6H, H-20); **^{13}C-NMR** (176 MHz, CD_2Cl_2, 298 K): δ [ppm] = 207.3 (C-14), 166.0 (C-23), 159.6 (C-2), 137.2 (C-8), 132.7 (C-27), 131.1 (C-5), 130.0 (C-30), 129.6 (2C, C-4), 128.7 (2C, C-29), 127.8 (2C, C-28), 123.2 (q, 1C, $^{1}J_{C,F}$ = 289 Hz, C-26), 120.9 (C-10), 114.0 (2C, C-3), 85.4 (q, 1C, $^{2}J_{C,F}$ = 28.3 Hz, C-24), 75.7 (C-7), 72.9 (C-12), 71.9 (C-6), 68.3 (C-19), 56.3 (d, $^{4}J_{C,F}$ = 1.8 Hz, C-25), 55.6 (C-1), 45.8, (C-13), 41.5 (C-15), 35.6 (C-17), 32.4 (C-11), 27.3 (C-16), 26.1 (3C, C-21), 18.6 (C-22), 16.6 (C-18), 14.3 (C-9), -5.3 (C-20), -5.3 (C-20); **HRMS (ESI+)** *m/z*: $[M+NH_4]^+$ calcd. for $C_{37}H_{53}F_3O_7SiNH_4^+$ 712.3851, found 712.386.

Table 1: Relevant ^{1}H-NMR signals for the MOSHER ester analysis of esters **S-1** and **S-2**.

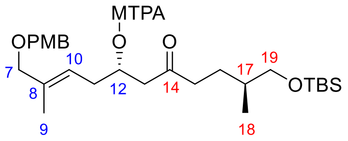

H	δ^S [ppm]	δ^R [ppm]	$\Delta\delta^{SR}$ [ppm]
19	3.41	3.38	+0.03
18	0.84	0.82	+0.02
17	1.53	1.50	+0.03
16b	1.32	1.25	+0.07
16a	1.66	1.58	+0.08
15b	2.38	2.26	+0.12
15a	2.45	2.40	+0.05
13b	2.65	2.59	+0.06
13a	2.83	2.78	+0.05
12	5.55	5.58	-0.03
11b	2.37	2.46	-0.09
11a	2.44	2.50	-0.06
10	5.29	5.43	-0.14
9	1.61	1.66	-0.05
7	3.80	3.86	-0.06

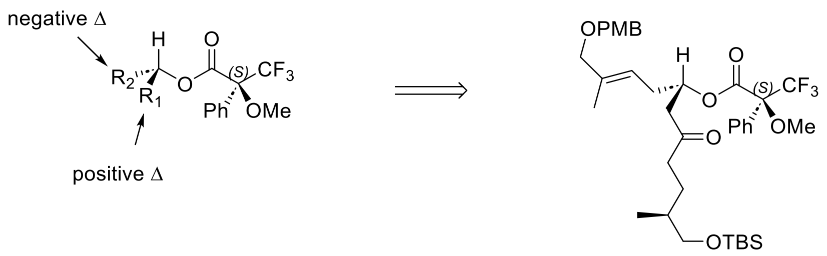

Scheme 1: MOSHER ester analysis confirmed the desired configuration.

For multiplets, the centers of the signals were selected to calculate the differences. The enantiomeric excess was determined by analysis of the ^{1}H NMR spectra of the MOSHER esters **S-1** and **S-2** to be *d.e.* = 50% and *d.r.* = 3:1.

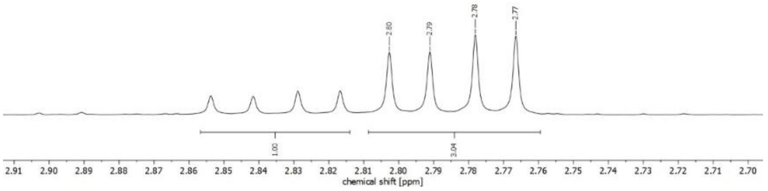

Figure 1: Excerpt of the ^{1}H-NMR spectra of compound **S-2.**

Synthesis of compound S-3

(5*S*,7*R*,10*S*,*E*)-11-((*tert*-Butyldimethylsilyl)oxy)-1-((4-methoxybenzyl)oxy)-2,10-dimethylundec-2-ene-5,7-diol

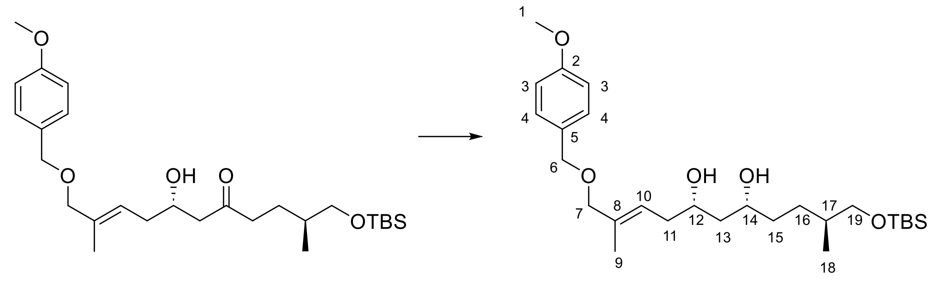

25 **S-3**

$C_{27}H_{48}O_5Si$

480.76g/mol

Et_2BOMe (1.0 M in THF, 290 µL, 290 µmol, 1.2 eq.) was added to a solution of aldol product **25** (115 mg, 240 µmol, 1.0 eq.) in THF/MeOH (4:1, 1.1 mL) at –78 °C. After 15 min, $NaBH_4$ (10.0 mg, 264 µmol, 1.1 eq.) was added in one portion and stirring continued for 4 h at –78 °C. Afterwards, NaOH-solution (3.0 M in H_2O, 2.5 mL) and H_2O_2 (35% in H_2O, 3.5 mL) were added and the biphasic mixture was stirred for additional 4 h at room temperature. Then, Et_2O (5 mL) and water (5 mL) were added and the phases were separated. The aqueous phase was extracted with Et_2O (3 × 20 mL). The combined organic phases were washed with water (50 mL), dried over Na_2SO_4 and the solvent was removed under reduced pressure. The crude product was purified by flash column chromatography (SiO_2, cyclohexane/ethyl acetate 6:1 → 2:1), yielding product **S-3** as a yellow oil (91.9 mg, 191 µmol, 80%).

R*f*: 0.26 (cyclohexane/ethyl acetate 2:1); **$[\alpha]_D^{20}$** = -4.1° (c = 0.74, CH_2Cl_2); **^{1}H-NMR** (700 MHz, CD_2Cl_2, 298 K): *δ* [ppm] = 7.25 (d, 2H, $^3J_{3,4}$ = 8.7 Hz, H-4), 6.87 (d, 2H, $^3J_{3,4}$ = 8.7 Hz, H-3), 5.47 (tq, 1H, $^3J_{10,11}$ = 7.4 Hz, $^4J_{9,10}$ = 1.4 Hz, H-10), 4.36 (s, 2H, H-6), 3.88 (d, 2H, $^4J_{7,9}$ = 1.3 Hz, H-7), 3.88 – 3.85 (m, 1H, H-12), 3.81 – 3.78 (m, 1H, H-14), 3.79 (s, 3H, H-1), 3.45 – 3.38 (m, 2H, H-19), 2.88 (br s, 2H, H-OH), 2.27 – 2.17 (m, 2H, H-11), 1.67 (s, 3H, H-9), 1.64 – 1.60 (m, 1H, H-13a), 1.60 – 1.55 (m, 1H, H-17), 1.51 – 1.39 (m, 4H, H-13b, H-15, H-16a), 1.12 – 1.08 (m, 1H, H-16b), 0.89 (s, 9H, TBS), 0.87 (d, 3H, $^3J_{17,18}$ = 6.7 Hz, H-18), 0.04 (s, 6H, TBS); **^{13}C-NMR** (176 MHz, CD_2Cl_2, 298 K): *δ* [ppm] = 159.6 (C-2), 135.8 (C-8), 131.2 (C-5), 129.7 (2C, C-4), 123.4 (C-10), 114.0 (2C, C-3), 76.2 (C-7), 73.8 (C-14), 73.1 (C-12), 71.7 (C-6), 68.5 (C-19), 55.6 (C-1), 43.0 (C-13), 36.9 (C-11), 36.2 (C-17), 36.0 (C-15), 29.2 (C-16), 26.1 (3C,

TBS), 18.6 (TBS), 17.0 (C-18), 14.3 (C-9), -5.3 (TBS), -5.3 (TBS); **HRMS (ESI+)** *m*/*z*: $[M+H]^+$ calcd. for $C_{27}H_{48}O_5SiH^+$ 481.3344, found 481.3344.

Synthesis of compound S-4

***tert*-Butyl((*S*)-4-((4*R*,6*S*)-6-((*E*)-4-((4-methoxybenzyl)oxy)-3-methylbut-2-en-1-yl)-2,2-dimethyl-1,3-dioxan-4-yl)-2-methylbutoxy)dimethylsilane**

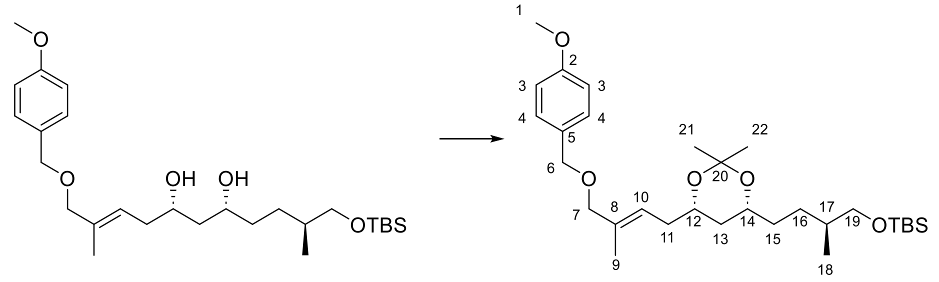

S-3

S-4
$C_{30}H_{52}O_5Si$
520.83 g/mol

Dimethoxy propane (2.0 mL) and CSA (16.4 mM in DCM, 14.0 µL, 230 nmol, 1.0 mol%) were added to diol **S-3** (11.0 mg, 22.9 µmol, 1.0 eq.). After stirring for 2 h at room temperature, the reaction was stopped by the addition of NEt_3 (15.0 µL) and DCM (10 mL) and the solvents were removed under reduced pressure. The crude product was purified by flash column chromatography (SiO_2, cyclohexane/ethyl acetate 9:1), affording product **S-4** as a yellow oil (9.60 mg, 18.4 µmol, 81%).

R_f: 0.51 (cyclohexane/ethyl acetate 4:1); **$[\alpha]_D^{20}$** = -1.0° (c = 1.00, CH_2Cl_2); **^{1}H-NMR** (700 MHz, CD_2Cl_2, 298 K): δ [ppm] = 7.25 (d, 2H, $^3J_{3,4}$ = 8.7 Hz, H-4), 6.86 (d, 2H, $^3J_{3,4}$ = 8.7 Hz, H-3), 5.44 (tq, 1H, $^3J_{10,11}$ = 7.2 Hz, $^4J_{9,10}$ = 1.4 Hz, H-10), 4.35 (s, 2H, H-6), 3.87 (d, 2H, $^4J_{7,9}$ = 1.2 Hz, H-7), 3.87 – 3.83 (m, 1H, H-12), 3.79 (s, 3H, H-1), 3.75 (dtd, 1H, $^3J_{13,14}$ = 11.4 Hz, $^3J_{14,15}$ = 6.2 Hz, $^3J_{13,14}$ = 2.4 Hz, H-14), 3.44 (dd, 1H, $^2J_{19a,19b}$ = 9.8 Hz, $^3J_{17,19a}$ = 5.9 Hz, H-19a), 3.37 (dd, 1H, $^2J_{19a,19b}$ = 9.8 Hz, $^3J_{17,19b}$ = 6.4 Hz, H-19b), 2.25 – 2.21 (m, 1H, H-11a), 2.18 – 2.14 (m, 1H, H-11b), 1.66 (d, 3H, $^4J_{9,10}$ = 1.4 Hz, H-9), 1.57 – 1.54 (m, 1H, H-17), 1.52 – 1.47 (m, 2H, H-13a, H-16a), 1.43 – 1.40 (m, 2H, H-15), 1.40 (s, 3H, H-21), 1.32 (s, 3H, H-22), 1.14 – 1.01 (m, 2H, H-13b, H-16b), 0.89 (s, 9H, TBS), 0.86 (d, 3H, $^3J_{17,18}$ = 6.6 Hz, H-18), 0.03 (s, 6H, TBS); **^{13}C-NMR** (176 MHz, CD_2Cl_2, 298 K): δ [ppm] = 159.6 (C-2), 134.8 (C-8), 131.3 (C-5), 129.7 (2C, C-4), 123.6 (C-10), 114.0 (2C, C-3), 98.7 (C-20), 76.2 (C-7),

71.4 (C-6), 69.7 (C-14), 69.4 (C-12), 68.6 (C-19), 55.6 (C-1), 37.1 (C-13), 36.2 (C-17), 35.3 (C-11), 34.4 (C-15), 30.4 (C-22), 28.8 (C-16), 26.1 (3C, TBS), 20.0 (C-21), 18.6 (TBS), 16.8 (C-18), 14.3 (C-9), -5.3 (TBS), -5.3 (TBS); **HRMS (ESI+)** *m*/*z*: $[M+NH_4]^+$ calcd. for $C_{30}H_{52}O_5SiNH_4^+$ 538.3922, found 538.3921.

Synthesis of compound 26

(5*S*,7*R*,10*S*)-7-((*tert*-Butyldimethylsilyl)oxy)-5-((*E*)-4-((4-methoxybenzyl)oxy)-3-methylbut-2-en-1-yl)-2,2,3,3,10,13,13,14,14-nonamethyl-4,12-dioxa-3,13-disilapentadecane

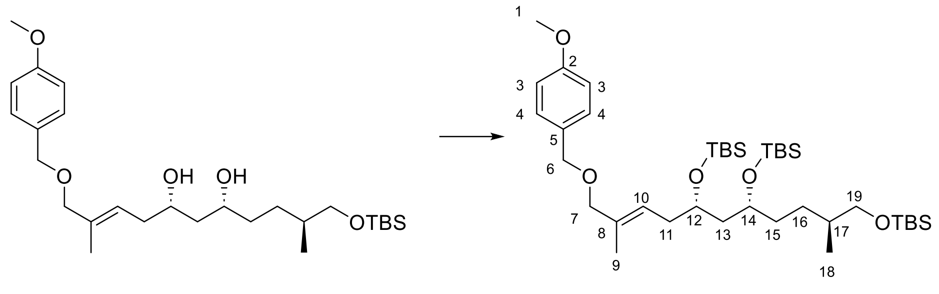

S-3

26
$C_{39}H_{76}O_5Si_3$
709.29 g/mol

2,6-Lutidine (40.0 µL, 345 µmol, 3.0 eq.) and TBSOTf (69.0 µL, 301 µmol, 2.6 eq.) were added dropwise to a solution of diol **S-3** (55.3 mg, 115 µmol, 1.0 eq.) in dry DCM (2.5 mL) at 0 °C. After stirring for 2 h at 0 °C, water (2 mL) was added and the phases were separated. The aqueous phase was extracted with DCM (4 × 2 mL). The combined organic layers were dried over Na_2SO_4 and the solvent was removed under reduced pressure. The crude product was purified by flash column chromatography (SiO_2, cyclohexane/ethyl acetate 19:1) and product **26** was obtained as a colorless oil (77.6 mg, 109 µmol, 95%).

R*f*: 0.54 (cyclohexane/ethyl acetate 9:1); **$[\alpha]_D^{20}$** = +6.4° (c = 0.78, CH_2Cl_2); **^{1}H-NMR** (500 MHz, CD_2Cl_2, 298 K): δ [ppm] = 7.24 (d, 2H, $^3J_{3,4}$ = 8.7 Hz, H-4), 6.86 (d, 2H, $^3J_{3,4}$ = 8.7 Hz, H-3), 5.50 – 5.47 (m, 1H, H-10), 4.34 (s, 2H, H-6), 3.87 (s, 2H, H-7), 3.86 – 3.81 (m, 1H, H-12), 3.79 (s, 3H, H-1), 3.79 – 3.73 (m, 1H, H-14), 3.43 (ddd, 1H, $^2J_{19a,19b}$ = 9.8 Hz, $^3J_{17,19a}$ = 5.8 Hz, $^3J_{16,19a}$ = 1.4 Hz, H-19a), 3.36 (ddd, 1H, $^2J_{19a,19b}$ = 9.8 Hz, $^3J_{17,19b}$ = 6.3 Hz, $^3J_{16,19a}$ = 1.1 Hz, H-19b), 2.29 – 2.18 (m, 2H, H-11), 1.66 (s, 3H, H-9), 1.66 – 1.60 (m, 1H, H-13a), 1.58 – 1.54 (m, 1H, H-13b), 1.52 – 1.39

(m, 3H, H-15a, H-16a, H-17), 1.36 – 1.29 (m, 1H, H-15b), 1.06 – 0.99 (m, 1H, H-16b), 0.89 (s, 9H, TBS), 0.89 (s, 9H, TBS), 0.88 (s, 9H, TBS), 0.86 (d, 3H, $^3J_{17,18}$ = 6.6 Hz, H-18), 0.05 (s, 6H, TBS), 0.04 (s, 6H, TBS), 0.03 (s, 6H, TBS); **^{13}C-NMR** (126 MHz, CD_2Cl_2, 298 K): *δ* [ppm] = 159.5 (C-2), 134.2 (C-8), 131.4 (C-5), 129.6 (2C, C-4), 124.5 (C-10), 114.0 (2C, C-3), 76.4 (C-7), 71.3 (C-6), 70.3 (C-14), 69.9 (C-12), 68.7 (C-19), 55.6 (C-1), 45.2 (C-13), 36.5 (C-17), 36.1 (C-11), 35.0 (C-15), 29.0 (C-16), 26.1 (3C, TBS), 26.1 (3C, TBS), 26.1 (3C, TBS), 18.6 (TBS), 18.3 (TBS), 18.3 (TBS), 17.0 (C-18), 14.4 (C-9), -4.1 (2C, TBS), -4.3 (TBS), -4.4 (TBS), -5.3 (TBS), -5.3 (TBS); **HRMS (APCI)** *m*/*z*: [M+H]$^+$ calcd. for $C_{39}H_{76}O_5Si_3H^+$ 709.5073, found 709.5078.

Synthesis of compound S-5

(5*S*,7*R*,10*S*,*E*)-5,7,11-Tris((*tert*-butyldimethylsilyl)oxy)-2,10-dimethylundec-2-enal

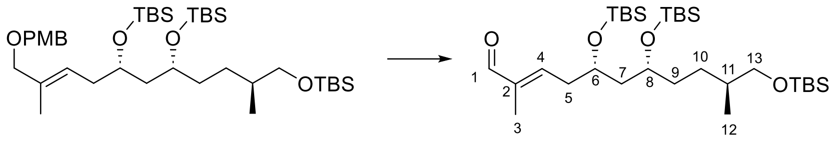

26

S-5
$C_{31}H_{66}O_4Si_3$
587.12 g/mol

DDQ (343 mg, 1.51 mmol, 3.0 eq.) was added to PMB-ether **26** (359 mg, 506 µmol, 1.0 eq.) in DCM/pH 7 buffer (10:1, 11.0 mL). After stirring for 2.5 h and 3.5 h at room temperature, further aliquots of DDQ (120 mg, 529 µmol, 1.0 eq. and 190 mg, 837 µmol, 1.7 eq.) were added. After a total stirring time of 5 h, the reaction mixture was diluted with water (50 mL) and DCM (40 mL) and the phases were separated. The aqueous phase was extracted with DCM (3 × 50 mL), the combined organic layers were dried over $MgSO_4$ and the solvent was removed under reduced pressure. The crude product was purified by flash column chromatography (SiO_2, cyclohexane/ethyl acetate 9:1) and product **S-5** was obtained as a yellow oil (259 mg, 440 µmol, 87%).

R*f*: 0.53 (cyclohexane/ethyl acetate 9:1); **$[\alpha]_D^{20}$** = +1.8° (c = 0.55, CH_2Cl_2); **^{1}H-NMR** (500 MHz, CD_2Cl_2, 298 K): *δ* [ppm] = 9.40 (s, 1H, H-1), 6.60 (ddq, 1H, $^3J_{4,5b}$ = 8.0 Hz, $^3J_{4,5a}$ = 6.7 Hz, $^4J_{3,4}$ = 1.4 Hz, H-4), 4.05 – 4.00 (m, 1H, H-6), 3.77 – 3.72 (m, 1H, H-8), 3.43 (ddd, 1H, $^2J_{13a,13b}$ = 9.8 Hz, $^3J_{11,13a}$ = 5.9 Hz, $^4J_{10,13a}$ = 1.8 Hz, H-13a), 3.38 (ddd,

1H, $^{2}J_{13a,13b}$ = 9.8 Hz, $^{3}J_{11,13b}$ = 5.9 Hz, H-13b), 2.60 – 2.55 (m, 1H, H-5a), 2.49 (dddt, 1H, $^{2}J_{5a,5b}$ = 15.3 Hz, $^{3}J_{4,5b}$ = 8.0 Hz, $^{3}J_{5b,6}$ = 5.7 Hz, $^{4}J_{5b,7}$ = 0.9 Hz, H-5b), 1.73 (d, 3H, $^{4}J_{3,4}$ = 1.4 Hz, H-3), 1.73 – 1.68 (m, 1H, H-7a), 1.59 – 1.54 (m, 2H, H-7b, H-9a), 1.51 – 1.44 (m, 2H, H-10a, H-11), 1.42 – 1.35 (m, 1H, H-9b), 1.08 – 1.01 (m, 1H, H-10b), 0.89 (s, 9H, TBS), 0.89 (s, 9H, TBS), 0.89 (s, 9H, TBS), 0.87 (d, 3H, $^{3}J_{11,12}$ = 6.7 Hz, H-12), 0.07 (s, 3H, TBS), 0.06 (s, 3H, TBS), 0.06 (s, 3H, TBS), 0.05 (s, 3H, TBS), 0.03 (s, 6H, TBS); **^{13}C-NMR** (126 MHz, CD_2Cl_2, 298 K): *δ* [ppm] = 195.2 (C-1), 151.3 (C-4), 140.8 (C-2), 70.2 (C-8), 68.9 (C-6), 68.6 (C-13), 45.2 (C-7), 37.0 (C-5), 36.5 (C-11), 35.2 (C-9), 28.8 (C-10), 26.1 (3C, TBS), 26.1 (3C, TBS), 26.0 (3C, TBS), 18.6 (TBS), 18.3 (TBS), 18.3 (TBS), 16.9 (C-12), 9.7 (C-3), -4.1 (TBS), -4.3 (3C, TBS), -5.3 (2C, TBS); **HRMS (ESI+)** *m*/*z*: $[M+H]^+$ calcd. for $C_{31}H_{66}O_4Si_3H^+$ 587.4342, found 587.4341.

Synthesis of compound 27

(5*S*,7*R*,10*S*,*E*)-5,7,11-Tris((*tert*-butyldimethylsilyl)oxy)-2,10-dimethylundec-2-enoic acid

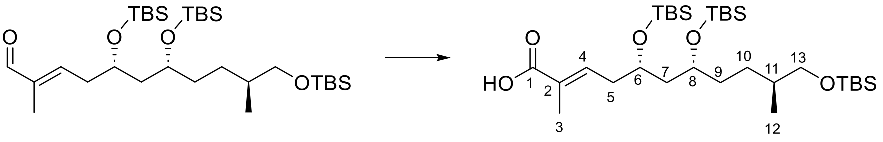

S-5

27
$C_{31}H_{66}O_5Si_3$
603.12 g/mol

2-Methyl-2-butene (181 µL, 1.71 mmol, 8.0 eq.) and a solution of $NaOCl_2$ (80%, 217 mg, 1.92 mmol, 9.0 eq.) and $NaH_2PO_4 \cdot 2H_2O$ (256 mg, 1.64 µmol, 7.7 eq.) in water (1.0 mL) were added to aldehyde **S-5** (125 mg, 213 µmol, 1.0 eq.) in *t*-BuOH (10 mL). After stirring for 2 h at room temperature, NH_4Cl-solution (aq., sat., 20 mL) and DCM (20 mL) were added and the phases were separated. The aqueous phase was extracted with DCM (3 × 25 mL), the combined organic layers were dried over Na_2SO_4 and the solvents were removed under reduced pressure. The crude product was purified by flash column chromatography (SiO_2, cyclohexane/ethyl acetate 9:1), yielding product **27** as a pale-yellow oil (127 mg, 210 µmol, 99%).

R_f: 0.28 (cyclohexane/ethyl acetate 9:1); **$[\alpha]_D^{20}$** = -1.6° (c = 0.63, CH_2Cl_2); **^{1}H-NMR** (700 MHz, CD_2Cl_2, 298 K): δ [ppm] = 6.97 (ddq, 1H, $^3J_{4,5}$ = 8.7 Hz, $^3J_{4,5}$ = 7.1 Hz, $^4J_{3,4}$ = 1.4 Hz, H-4), 3.98 – 3.94 (m, 1H, H-6), 3.76 – 3.72 (m, 1H, H-8), 3.44 (dd, 1H, $^2J_{13a,13b}$ = 9.7 Hz, $^3J_{11,13a}$ = 5.7 Hz, H-13a), 3.38 (ddd, 1H, $^2J_{13a,13b}$ = 9.7 Hz, $^3J_{11,13b}$ = 6.2 Hz, $^3J_{10,13b}$ = 1.1 Hz, H-13b), 2.45 – 2.41 (m, 1H, H-5a), 2.36 – 2.32 (m, 1H, H-5b), 1.84 (d, 3H, $^3J_{3,4}$ = 1.4 Hz, H-3), 1.69 (ddd, 1H, $^2J_{7a,7b}$ = 13.5 Hz, $^3J_{6,7a}$ = 7.1 Hz, $^3J_{7a,8}$ = 6.0 Hz, H-7a), 1.58 – 1.45 (m, 4H, H-7b, H-9a, H-10a, H-11), 1.40 – 1.35 (m, 1H, H-9b), 1.09 – 1.02 (m, 1H, H-10b), 0.89 (s, 9H, TBS), 0.89 (s, 9H, TBS), 0.88 (s, 9H, TBS), 0.87 (d, 3H, $^3J_{11,12}$ = 6.7 Hz, H-12), 0.07 (s, 3H, TBS), 0.06 (s, 3H, TBS), 0.06 (s, 3H, TBS), 0.05 (s, 3H, TBS), 0.03 (s, 6H, TBS); **^{13}C-NMR** (176 MHz, CD_2Cl_2, 298 K): δ [ppm] = 172.0 (C-1), 142.0 (C-4), 128.4 (C-2), 70.2 (C-8), 69.1 (C-6), 68.6 (C-13), 45.4 (C-7), 37.2 (C-5), 36.5 (C-11), 35.2 (C-9), 28.9 (C-10), 26.1 (3C, TBS), 26.1 (3C, TBS), 26.0 (3C, TBS), 18.6 (TBS), 18.3 (TBS), 18.3 (TBS), 16.9 (C-12), 12.6 (C-3), -4.1 (TBS), -4.3 (TBS), -4.4 (TBS), -4.4 (TBS), -5.3 (TBS), -5.3 (TBS); **HRMS (ESI+)** *m*/*z*: $[M+H]^+$ calcd. for $C_{31}H_{66}O_5Si_3H^+$ 603.4291, found 603.4300.

Synthesis of compound 28

(*Z*)-1-Methyl-2-(2-methylpropylidene)-5-oxo-2,5-dihydro-1*H*-pyrrol-3-yl (5*S*,7*R*,10*S*,*E*)-5,7,11-tris((*tert*-butyldimethylsilyl)oxy)-2,10-dimethylundec-2-enoate

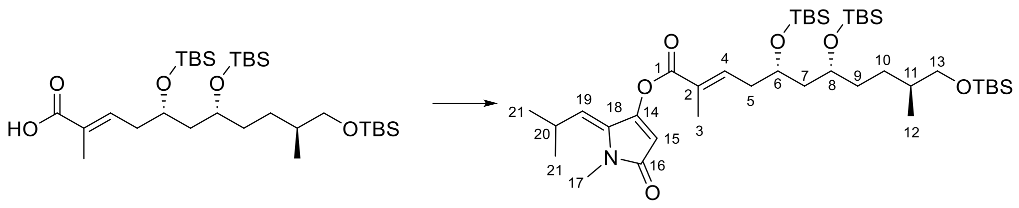

27 **28**

$C_{40}H_{77}NO_6Si_3$
752.31 g/mol

DMAP (10.2 mg, 83.5 µmol, 0.22 eq.) and DCC (1.0 M in DCM, 440 µL, 440 µmol, 1.2 eq.) were added to carboxylic acid **27** (266 mg, 441 µmol, 1.2 eq.) in DCM (1.5 mL) at 0 °C. After stirring for 15 min at 0 °C, tetramic acid **17** (61.4 mg, 367 µmol, 1.0 eq.) was added and stirring at room temperature continued for 15 h. Afterwards, the reaction mixture was filtered over Celite® and the solvent was removed under reduced pressure. The crude product was purified by flash column chromatography (SiO_2, cyclohexane/ethyl acetate 9:1 → 7:1), yielding product **28** as a yellow solid (236 mg, 314 µmol, 86%).

R_f: 0.33 (cyclohexane/ethyl acetate 9:1); **$[\alpha]_D^{20}$** = -1.3° (c = 0.79, CH_2Cl_2); **^{1}H-NMR** (500 MHz, CD_2Cl_2, 298 K): *δ* [ppm] = 7.07 (tq, 1H, $^3J_{4,5}$ = 7.3 Hz, $^4J_{3,4}$ = 1.4 Hz, H-4), 5.98 (d, 1H, $^5J_{15,19}$ = 0.7 Hz, H-15), 5.27 (dd, 1H, $^3J_{19,20}$ = 10.7 Hz, $^5J_{15,19}$ = 0.7 Hz, H-19), 4.02 (p, 1H, $^3J_{5,6}$ = 5.9 Hz, $^3J_{6,7}$ = 5.9 Hz, H-6), 3.77 – 3.72 (m, 1H, H-8), 3.43 (ddd, 1H, $^2J_{13a,13b}$ = 9.8 Hz, $^3J_{11,13a}$ = 5.9 Hz, $^4J_{10,13a}$ = 2.0 Hz, H-13a), 3.38 (ddd, 1H, $^2J_{13a,13b}$ = 9.8 Hz, $^3J_{11,13b}$ = 6.0 Hz, H-13b), 3.29 (s, 3H, H-17), 3.15 (dhept, 1H, $^3J_{19,20}$ = 10.7 Hz, $^3J_{20,21}$ = 6.6 Hz, H-20), 2.52 – 2.47 (m, 1H, H-5a), 2.44 – 2.48 (m, 1H, H-5b), 1.93 (d, 3H, $^3J_{3,4}$ = 1.3 Hz, H-3), 1.72 (ddd, 1H, $^2J_{7a,7b}$ = 13.4 Hz, $^3J_{7a,8}$ = 7.4 Hz, $^3J_{6,7a}$ = 5.9 Hz, H-7a), 1.60 – 1.53 (m, 2H, H-7b, H-9a), 1.51 – 1.45 (m, 2H, H-10a, H-11), 1.43 – 1.36 (m, 1H, H-9b), 1.12 (d, 6H, $^3J_{20,21}$ = 6.6 Hz, H-21), 1.08 – 1.01 (m, 1H, H-10), 0.90 – 0.87 (m, 30H, H-12, TBS), 0.08 (s, 3H, TBS), 0.07 (s, 3H, TBS), 0.06 (s, 3H, TBS), 0.06 (s, 3H, TBS), 0.03 (s, 6H, TBS); **^{13}C-NMR** (126 MHz, CD_2Cl_2, 298 K): *δ* [ppm] = 169.8 (C-16), 163.6 (C-1), 155.3 (C-14), 143.5 (C-4), 132.9 (C-18), 128.2 (C-2), 119.1 (C-19), 104.4 (C-15), 70.2 (C-8), 69.0 (C-6), 68.6 (C-13), 45.4 (C-7), 37.2 (C-5), 36.5 (C-11), 35.3 (C-9), 28.8 (C-10), 27.9 (C-17), 26.1 (6C, TBS), 26.1 (3C,

TBS), 26.0 (C-20), 23.9 (2C, C-21), 18.6 (TBS), 18.3 (2C, TBS), 16.9 (C-12), 13.0 (C-3), -4.0 (TBS), -4.2 (TBS), -4.2 (TBS), -4.3 (TBS), -5.3 (2C, TBS); **HRMS (ESI+)** *m/z*: $[M+H]^+$ calcd. for $C_{40}H_{77}NO_6Si_3H^+$ 752.5131, found 752.5136.

Synthesis of compound 29

(*Z*)-4-Hydroxy-1-methyl-5-(2-methylpropylidene)-3-((5*S*,7*R*,10*S*,*E*)-5,7,11-tris((*tert*-butyldimethylsilyl)oxy)-2,10-dimethylundec-2-enoyl)-1,5-dihydro-2*H*-pyrrol-2-one

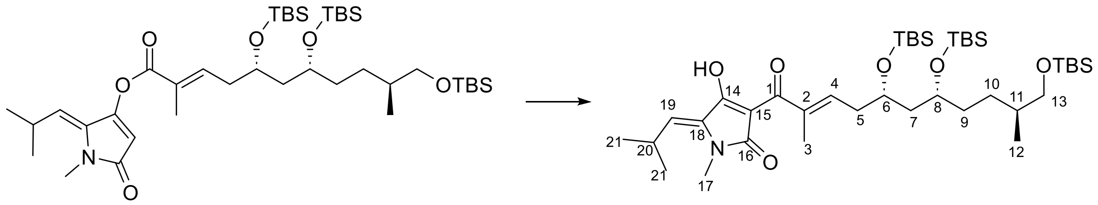

28 **29**

$C_{40}H_{77}NO_6Si_3$
752.31 g/mol

NEt_3 (15.0 mg, 108 µmol, 1.3 eq.), $CaCl_2$ (16.1 mg, 145 µmol, 1.8 eq) and DMAP (5.30 mg, 43.3 µmol, 0.53 eq.) were added to ester **28** (61.2 mg, 81.4 µmol, 1.0 eq.) in DCM (3.0 mL). After stirring for 70 h at room temperature, citric acid (0.5 M in H_2O, 15 mL), H_2O (15 mL) and DCM (30 mL) were added and the phases were separated. The aqueous phase was extracted with DCM (3 x 40 mL), the combined organic layers were washed with water (200 mL), dried over Na_2SO_4 and concentrated *in vacuo*. The resulting brown/yellow solid was dissolved in DCM (20 mL) and washed with HCl (0.5 M in H_2O, 20 mL). The aqueous phase was extracted with DCM (3 x 20 mL), dried over Na_2SO_4 and concentrated *in vacuo*. Product **29** was obtained as a yellow oil (59.7 mg, 79.4 µmol, 98%).

R*f*: 0.38 (cyclohexane/ethyl acetate 4:1); **$[\alpha]_D^{20}$** = +2.8° (c = 0.36, CH_2Cl_2); **^{1}H-NMR** (400 MHz, CD_2Cl_2, 298 K): δ [ppm] = 7.12 (tq, 1H, $^3J_{4,5}$ = 7.5 Hz, $^4J_{3,4}$ = 1.3 Hz, H-4), 7.06 (tq, 1H, $^3J_{4',5}$ = 7.5 Hz, $^4J_{3,4'}$ = 1.3 Hz, H-4'), 5.60 (dd, 1H, $^3J_{19,20}$ = 10.8 Hz, H-19), 3.98 (p, 1H, $^3J_{5,6}$ = 6.1 Hz, $^3J_{6,7}$ = 6.1 Hz, H-6), 3.77 (p, 1H, $^3J_{7,8}$ = 6.0 Hz, $^3J_{8,9}$ = 6.0 Hz, H-8), 3.44 (ddd, 1H, $^2J_{13a,13b}$ = 9.7 Hz, $^3J_{11,13a}$ = 5.7 Hz, $^4J_{10,13a}$ = 2.4 Hz, H-13a), 3.39 – 3.34 (m, 1H, H-13b), 3.35 (s, 3H, H-17), 3.28 (s, 3H, H-17'), 3.14 – 2.98 (m, 1H, H-20), 2.53 – 2.41 (m, 2H, H-5), 1.93 (d, 3H, $^4J_{3,4}$ = 1.3 Hz, H-3), 1.72 – 1.65 (m, 2H, H-7), 1.58 – 1.43 (m, 3H, H-9a, H-10a, H-11), 1.40 – 1.33 (m, 1H, H-9b), 1.13

(d, 6H, $^3J_{20,21}$ = 6.5 Hz, H-21), 1.11 (d, 6H, $^3J_{20,21}$ = 6.5 Hz, H-21'), 1.07 – 1.00 (m, 1H, H-10b), 0.89 – 0.86 (m, 30H, 3 x TBS, H-12), 0.06 (s, 3H, TBS), 0.06 (s, 6H, TBS), 0.05 (s, 3H, TBS), 0.03 (s, 3H, TBS), 0.03 (s, 3H, TBS); **^{13}C-NMR** (176 MHz, CD_2Cl_2, 298 K): *δ* [ppm] = 175.4 (C-14), 169.4 (C-1), 166.2 (C-16), 141.7 (C-4), 133.1 (C-18), 130.7 (C-2), 120.0 (C-19), 102.7 (C-15), 70.3 (C-8), 69.2 (C-6), 68.7 (C-13), 43.5 (C-7), 37.6 (C-5), 36.5 (C-11), 35.0 (C-9), 29.0 (C-10), 28.1 (C-1), 26.1 (3C, TBS), 26.1 (3C, TBS), 26.1 (3C, TBS), 26.0 (C-20), 23.8 (2C, C-4), 18.6 (TBS), 18.4 (TBS), 18.3 (TBS), 17.0 (C-12), 13.1 (C-3), -4.2 (TBS), -4.3 (2C, TBS), -4.4 (TBS), -5.3 (TBS), -5.3 (TBS); **HRMS (ESI+)** *m*/*z*: $[M+H]^+$ calcd. for $C_{40}H_{77}NO_6Si_3H^+$ 752.5131, found 752.5131.

Synthesis of compound 6

(*Z*)-4-Hydroxy-1-methyl-5-(2-methylpropylidene)-3-((5*S*,7*R*,10*S*,*E*)-5,7,11-trihydroxy-2,10-dimethylundec-2-enoyl)-1,5-dihydro-2*H*-pyrrol-2-one

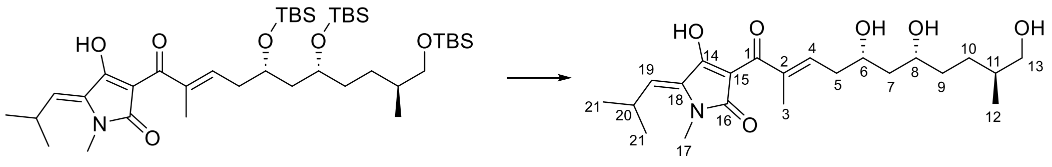

29 **6**

$C_{22}H_{35}NO_6$

409.52 g/mol

2-Nitrophenol (56.0 mg, 403 µmol, 4.7 eq.) and TBAF (1.0 M in THF, 350 µL, 350 µmol, 4.1 eq.) were added to compound **29** (64.7 mg, 86.0 µmol, 1.0 eq.) in THF (4.0 mL). After stirring for 21 h at room temperature, TBAF (1.0 M in THF, 350 µL, 350 µmol, 4.1 eq.) and THF (2.0 mL) were added and stirring continued for 67 h. The yellow suspension was directly purified by flash column chromatography (DCM/MeOH 10:1), yielding product **6** as a colorless amorphous solid (29.6 mg, 72.3 µmol, 84%).

R_f: 0.16 (ethyl acetate/MeOH 9:1); **$[\alpha]_D^{20}$** = -3.3° (c = 0.60, MeOD); **^{1}H-NMR** (700 MHz, MeOD, 298 K): *δ* [ppm] = 5.83 (t, 1H, $^5J_{15,19}$ = 0.6 Hz, H-15), 5.56 – 5.34 (2 x br s, 1H, H-19), 3.94 – 3.89 (m, 1H, H-6), 3.79 – 3.74 (m, 1H, H-8), 3.46 – 3.42 (m, 1H, H-13a), 3.36 – 3.31 (m, 1H, H-13b), 3.24 (s, 3H, H-17), 3.09 (dhept, 1H, $^3J_{19,20}$ = 10.7 Hz, $^3J_{20,21}$ = 6.6 Hz, H-20), 2.36 – 2.26 (m, 2H, H-5), 1.84 (s, 3H, H-3), 1.70 – 1.61 (m, 2H, H-7), 1.60 – 1.39 (m, 4H, H-9, H-10, H-11), 1.35 – 1.22 (m, 1H, H-10b), 1.10 (dd, 6H, $^3J_{20,21}$ = 6.6 Hz, $^4J_{19,21}$ = 1.4 Hz, H-21), 0.93 (2 x d, 3H, $^3J_{11,12}$ = 6.6 Hz, H-12);

^{13}C-NMR (176 MHz, MeOD, 298 K): *δ* [ppm] = 195.0 (C-1), 183.6 (C-14), 174.3 (C-16), 141.2 (C-4), 136.3 (C-18), 130.9 (C-2), 116.5 (C-19), 100.4 (C-15), 71.8 (C-8), 71.2 (C-6), 68.3 (C-13), 44.4 (C-7), 37.5 (C-5), 37.1 (C-11), 35.8 (C-9), 30.1 (C-10), 27.8 (C-17), 26.5 (C-20), 24.4 (2C, C-21), 17.2 (C-12), 13.9 (C-3); **HRMS (ESI+)** *m*/*z*: $[M+H]^+$ calcd. for $C_{22}H_{35}NO_6H^+$ 410.2537, found 410.2539.

HPLC (analytical): t_R = 2.086 min; on System GANYMED+ with KNAUER Eurospher II 100-3 C18P; 3 µm; 2.0 mm x 100 mm (N21052206), isocratic MeCN/NH_4Ac (20 mM, pH 6.8) = 20:80, flow rate: 0.5 mL/min, total running time: 6.0 min, 25 °C, detection at 275 nm.

HPLC (preparative): t_R = 11.037 min; on System TETHYS + with KNAUER Eurospher II 100-5 C18P; 5 µm; 250 x 16 mm + Vorsäule (CJ158 + CJ145); isocratic MeCN/ NH_4Ac (20 mM, pH 6.8) = 20:80, flow rate: 16.0 mL/min, total running time: 30 min, detection at 275 nm.

Synthesis of Eastern Fragment 8

Synthesis of compound S-6

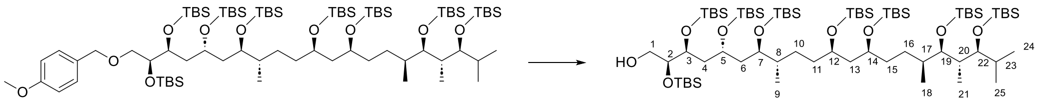

32

S-6

$C_{73}H_{164}O_9Si_8$

1410.79 g/mol

DDQ (50.6 mg, 223 µmol, 1.7 eq.) was added to a solution of PMB ether **32**[3] (206 mg, 135 µmol, 1.0 eq.) in DCM (10 mL) and pH 7 buffer (1.0 mL). After stirring for 5.5 h at room temperature, $NaHCO_3$-solution (aq., sat., 6 mL), $Na_2S_2O_3$-solution (aq., sat., 6 mL) and water (10 mL) were added and the phases were separated. The aqueous phase was extracted with DCM (3 x 30 mL), the combined organic phases were washed with water (80 mL), dried over $MgSO_4$ and the solvent was removed under reduced pressure. The crude product was purified by flash column chromatography (SiO_2, cyclohexane/ethyl acetate 40:1), yielding product **S-6** as a colorless oil (173 mg, 122 µmol, 91%).

R*f*: 0.19 (cyclohexane/ethyl acetate 30:1); **$[\alpha]_D^{20}$** = -13.0° (c = 1.00, $CHCl_3$); **^{1}H-NMR** (700 MHz, CD_2Cl_2, 298 K): δ [ppm] = 3.87 (ddd, 1H, $^3J_{3,4}$ = 9.8 Hz, $^3J_{2,3}$ = 4.2 Hz, $^3J_{3,4}$ = 1.5 Hz, H-3), 3.79 – 3.74 (m, 3H, H-2, H-5, H-12), 3.74 – 3.65 (m, 5H, H-1a, H-7, H-14, H-19, H-22), 3.54 (ddd, 1H, $^2J_{1a,1b}$ = 11.0 Hz, $^3J_{1b,2}$ = 6.4 Hz, $^3J_{1b,OH}$ = 6.1 Hz, H-1b), 1.95 (t, 1H, $^3J_{1,OH}$ = 6.1 Hz, H-OH), 1.88 (ddd, 1H, $^3J_{19,20}$ = 7.3 Hz, $^3J_{20,21}$ = 7.3 Hz, $^3J_{20,22}$ = 4.7 Hz, H-20), 1.86 – 1.79 (m, 2H, H-4a, H-23), 1.69 – 1.63 (m, 3H, H-6a, H-13a, H-17), 1.62 – 1.58 (m, 1H, H-11a), 1.56 – 1.51 (m, 2H, H-4b, H-6b), 1.50 – 1.44 (m, 4H, H-8, H-10a, H-13b, H-15a), 1.44 – 1.38 (m, 2H, H-15b, H-16a), 1.30 – 1.23 (m, 2H, H-11b, H-16b), 1.10 – 1.04 (m, 1H, H-10b), 0.96 (t, 9H, $^3J_{TES,TES}$ = 8.0 Hz, TES), 0.93 (d, 3H, $^3J_{24,25}$ = 6.9 Hz, H-24), 0.92 – 0.88 (m, 72H, H-9, H-21, H-25, 7 x TBS), 0.87 (d, 3H, $^3J_{17,18}$ = 6.9 Hz, H-18), 0.61 (q, 6H, $^3J_{TES,TES}$ = 8.0 Hz, TES), 0.13 – 0.04 (m, 42H, 7 x TBS); **^{13}C-NMR** (176 MHz, CD_2Cl_2, 298 K): δ [ppm] = 77.9 (C-19), 77.6 (C-22), 74.7 (C-2), 74.5 (C-7), 72.6 (C-3), 70.4 (C-12), 70.2 (C-14), 69.6 (C-5), 63.3 (C-1), 45.5 (C-13), 45.0 (C-20), 42.7 (C-6), 39.6 (C-4), 39.3 (C-8), 37.5 (C-17), 36.4 (C-15), 35.8 (C-11), 31.2 (C-23), 30.3 (C-16), 27.5 (C-10), 26.5 (3C, TBS), 26.4 (3C, TBS), 26.3 (3C, TBS), 26.2 (3C, TBS), 26.2 (3C, TBS), 26.1 (3C, TBS), 26.1 (3C, TBS), 21.5 (C-24), 18.8 (TBS), 18.7 (TBS), 18.6 (C-25), 18.4 (TBS), 18.4 (TBS), 18.3 (TBS), 18.3 (TBS), 18.3 (TBS), 15.5 (C-9), 14.4 (C-18), 12.2 (C-21), 7.3 (3C, TES), 5.8 (3C, TES), -2.9 (TBS), -3.2 (TBS), -3.3 (2C, TBS), -3.5 (TBS), -3.7 (TBS), -3.9 (TBS), -4.0 (TBS), -4.0 (2C, TBS), -4.2 (TBS), -4.2 (TBS), -4.2 (TBS), -4.5 (TBS); **HRMS (ESI+)** *m/z*: $[M + H]^+$ calcd. for $C_{73}H_{164}O_9Si_8H^+$ 1410.0602, found 1410.0604.

Synthesis of compound 8

(2*S*,3*S*,5*R*,7*R*,8*S*,11*R*,13*S*,16*S*,17*R*,18*S*,19*S*)-8,16,18,20-Tetramethylhenicosane-1,2,3,5,7,11,13,17,19-nonaol

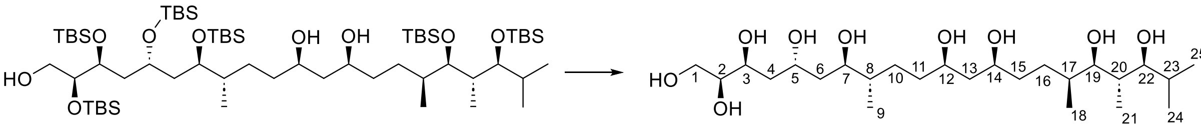

S-6

4

$C_{25}H_{52}O_9$

496.68 g/mol

To compound **S-6** (48.0 mg, 34.0 µmol, 1.0 eq.) in dry MeOH (4.0 mL) was added HCl (1.0 M in H_2O, 410 µL, 410 µmol, 12 eq.). The solution was stirred at room temperature for 72 h before $NaHCO_3$ (36.0 mg, 429 µmol, 13 eq.) was added. After further stirring for 5 min, the solvent was removed *in vacuo* and the crude product was purified by flash column chromatography (POLYGOPREP® 60-50 C_{18} SiO_2, H_2O/MeCN 9:1 → 7:1), yielding an amorphous colorless solid (4.30 mg, 8.66 µmol, 25%).

R_f: 0.35 (DCM/MeOH 4:1); **$[\alpha]_D^{20}$** = +6.5° (c = 0.46, MeOH); **^{1}H-NMR** (700 MHz, MeOD, 298 K): *δ* [ppm] = 4.08 (tt, 1H, $^3J_{5,6}$ = 9.6 Hz, $^3J_{5,4}$ = 2.9 Hz, H-5), 3.88 (dt, 1H, $^3J_{3,4}$ = 10.2 Hz, $^3J_{3,2}$ = 3.1 Hz, H-3), 3.78 – 3.71 (m, 3H, H-7, H-12, H-14), 3.65 (dd, 1H, $^2J_{1a,1b}$ = 11.2 Hz, $^3J_{1a,2}$ = 4.8 Hz, H-1a), 3.59 – 3.56 (m, 1H, H-1b), 3.56 – 3.53 (m, 1H, H-19), 3.49 (ddd, 1H, $^3J_{2,1b}$ = 6.9 Hz, $^3J_{2,1a}$ = 4.8 Hz, $^3J_{2,3}$ = 3.1 Hz, H-2), 3.44 – 3.39 (m, 1H, H-22), 1.89 (pq, 1H, $^3J_{23,22}$ = 6.9 Hz, $^3J_{23,24}$ = 6.9 Hz, $^3J_{23,25}$ = 3.0 Hz, H-23), 1.73 – 1.69 (m, 1H, H-20), 1.68 – 1.64 (m, 2H, H-4a, H-17), 1.64 – 1.63 (m, 1H, H-10a), 1.63 – 1.60 (m, 1H, H-13a), 1.61 – 1.59 (m, 1H, H-11a), 1.58 – 1.55 (m, 1H, H-4b), 1.55 – 1.52 (m, 3H, H-13b, H-6a, H-8), 1.51 – 1.47 (m, 1H, H-6b), 1.47 – 1.45 (m, 1H, H-15), 1.45 – 1.43 (m, 2H, H-16), 1.39 – 1.35 (m, 1H, H-11b), 1.16 (tdd, 1H, $^3J_{10b,11a}$ = 11.3 Hz, $^3J_{10b,8}$ = 8.4 Hz, $^3J_{10b,11b}$ = 3.9 Hz, H-10b), 0.98 (d, 3H, $^3J_{24,23}$ = 6.9 Hz, H-24), 0.92 (d, 3H, $^3J_{9,8}$ = 5.2 Hz, H-8), 0.88 (d, 3H, $^3J_{18,17}$ = 6.7 Hz, H-18), 0.86 (d, 3H, $^3J_{25,23}$ = 6.9 Hz, H-25), 0.77 (d, 3H, $^3J_{21,20}$ = 6.8 Hz, H-21); **^{13}C-NMR** (176 MHz, MeOD, 298 K): *δ* [ppm] = 82.2 (C-22), 80.2 (C-19), 76.3 (C-2), 72.9 (C-7), 72.2 (C-12), 71.9 (C-14), 69.7 (C-3), 66.5 (C-5), 64.6 (C-1), 44.8 (C-13), 42.6 (C-4), 42.0 (C-6), 40.7 (C-8), 39.5 (C-20), 36.4 (C-15), 36.4 (C-11, C-17), 31.2 (C-16, C-23), 29.3 (C-10), 20.8 (C-24), 15.6 (C-9), 14.7 (C-25), 13.6 (C-23), 12.7 (C-18); **HRMS (ESI+)** *m/z*: $[M + H]^+$ calcd. for $C_{25}H_{52}O_9H^+$ 497.3688, found: 497.3688.

Synthesis of Western Fragment 7

Synthesis of compound S-8

(*S*)-1-((*tert*-Butyldimethylsilyl)oxy)hex-5-en-3-ol

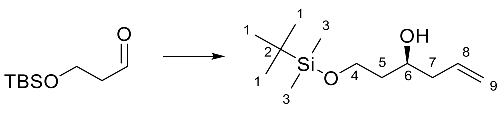

S-7 **S-8**

$C_{12}H_{26}O_2Si$

230.42 g/mol

Aldehyde **S-7** (5.35 g, 28.4 mmol, 1.00 eq.) was dried in dry Et_2O (10.0 mL) over 4 Å molecular sieves overnight. To (—)-Ipc_2BOMe (11.8 g, 37.3 mmol, 1.31 eq.) in dry Et_2O (50 mL) was added allyl-MgBr (1.0 M in Et_2O, 32.0 mL, 32.0 mmol, 1.13 eq.) dropwise in 10 min at 0 °C. The cooling bath was removed and stirring at room temperature continued for 1 h. The white-opaque suspension was cooled to –78 °C and aldehyde **S-7** (+2 x 1.5 mL rinse) was added dropwise within 10 min. After stirring for 1.5 h at –78 °C, NaOH (3.0 M in H_2O, 80 mL), THF (40 mL) and H_2O_2 (35%, 30 mL) were added and stirring at reflux continued for 2 h. Then, the phases were separated and the aqueous phase was extracted with Et_2O (3 x 100 mL). The solvents were removed under reduced pressure until ~100 mL remained. $FeSO_4$ (20.1 g) in water (100 mL) was added and stirring at room temperature continued for 16 h. Afterwards, the phases were separated and the aqueous phase was extracted with diethyl ether (3 x 100 mL). The combined organic phases were dried over $MgSO_4$ and all solvents were removed under reduced pressure. After flash column chromatography (SiO_2, cyclohexane/ethyl acetate 9:1), product **S-8** (5.51 g, 23.9 mmol, 84%) was obtained as a colorless oil.

R_f: 0.39 (cyclohexane/ethyl acetate 9:1); **$[\alpha]_D^{20}$** = -7.8° (c = 1.15, $CHCl_3$); **^{1}H-NMR** (500 MHz, CD_2Cl_2, 298 K): δ [ppm] = 5.85 (ddt, 1H, $^3J_{8,9E}$ = 17.3 Hz, $^3J_{8,9Z}$ = 10.2 Hz, $^3J_{7,8}$ = 7.2 Hz, H-8), 5.11 – 5.04 (m, 2H, H-9), 3.87 (ddd, 1H, $^2J_{4a,4b}$ = 10.2 Hz, $^3J_{4a,5}$ = 5.5 Hz, $^3J_{4a,5}$ = 4.7 Hz, H-4a), 3.83 (dtdd, 1H, $^3J_{5,6}$ = 8.8 Hz, $^3J_{6,7}$ = 6.2 Hz, $^3J_{5,6}$ = 3.0 Hz, $^3J_{6,OH}$ = 2.7 Hz, H-6), 3.79 (ddd, 1H, $^2J_{4a,4b}$ = 10.2 Hz, $^3J_{4b,5}$ = 8.1 Hz, $^3J_{4b,5}$ = 4.5 Hz, H-4b), 3.00 (d, 1H, $^3J_{6,OH}$ = 2.7 Hz, H-OH), 2.21 (ddt, 2H, $^3J_{7,8}$ = 7.2 Hz, $^3J_{6,7}$ = 6.2 Hz, $^4J_{7,9}$ = 1.3 Hz, H-7), 1.69 – 1.57 (m, 2H, H-5), 0.90 (s, 9H, H-1), 0.08 (s, 6H, H-3); **^{13}C-NMR** (126 MHz, CD_2Cl_2, 298 K): δ [ppm] = 135.8 (C-8), 117.2 (C-9), 71.1 (C-6), 62.7 (C-4), 42.5 (C-7), 38.5 (C-5), 26.0 (3C, C-1), 18.4 (C-2),

-5.4 (C-3), -5.4 (C-3); **HRMS (ESI+)** *m/z*: $[M + H]^+$ calcd. for $C_{12}H_{26}O_2SiH^+$ 231.1775, found 231.1765.

Synthesis of compound S-9

(*S*)-1-((*tert*-Butyldimethylsilyl)oxy)hex-5-en-3-yl (*R*)-3,3,3-trifluoro-2-methoxy-2-phenylpropanoate

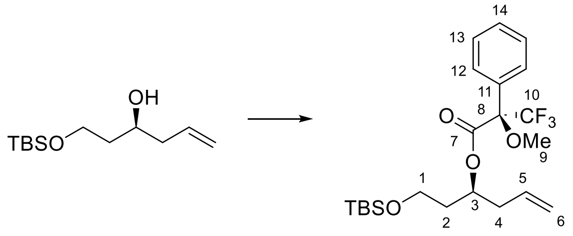

S-8 **S-9**

$C_{22}H_{33}F_3O_4Si$

446.58 g/mol

Pyridine (20.0 µL, 248 µmol, 11.2 eq.) and (*S*)-MTPA-Cl (12.0 µL, 64.1 µmol, 2.9 eq.) were added to alcohol **S-8** (5.10 mg, 22.1 µmol, 1.0 eq.) in dry DCM (0.6 mL). After stirring for 16 h at room temperature, water (1.5 mL) and DCM (1 mL) were added. The phases were separated and the aqueous phase was extracted with DCM (4 x 2 mL). The combined organic phases were dried over $MgSO_4$ and the solvent was removed under reduced pressure. Purification by flash column chromatography (SiO_2, cyclohexane/ethyl acetate 19:1) gave MOSHER ester **S-9** (10.1 mg, 22.1 µmol, quant.) as a colorless oil.

R_f: 0.54 (cyclohexane/ethyl acetate 9:1); **$[\alpha]_D^{20}$** = +50.8° (c = 1.30, $CHCl_3$); **^{1}H-NMR** (500 MHz, CD_2Cl_2, 298 K): δ [ppm] = 7.54 – 7.52 (m, 2H, H-12), 7.43 – 7.38 (m, 3H, H-13, H-14), 5.80 (dddd, 1H, $^3J_{5,6E}$ = 17.0 Hz, $^3J_{5,6Z}$ = 10.3 Hz, $^3J_{4,5}$ = 7.4 Hz, $^3J_{4,5}$ = 6.7 Hz, H-5), 5.34 – 5.29 (m, 1H, H-3), 5.15 – 5.10 (m, 2H, H-6), 3.57 – 3.46 (m, 5H, H-1, H-9), 2.51 – 2.45 (m, 2H, H-4), 1.79 (dt, 2H, $^3J_{2,3}$ = 6.8 Hz, $^3J_{1,2}$ = 6.1 Hz, H-2), 0.88 (s, 9H, TBS), 0.01 (s, 3H, TBS), 0.01 (s, 3H, TBS); **^{13}C-NMR** (126 MHz, CD_2Cl_2, 298 K): δ [ppm] = 166.3 (C-7), 133.7 (C-5), 132.9 (C-11), 130.0 (C-14), 128.7 (2C, C-13), 127.8 (2C, C-12), 123.9 (q, 1C, $^1J_{C,F}$ = 288 Hz, C-10), 118.6 (C-6), 84.9 (q, 1C, $^2J_{C,F}$ = 27.5 Hz, C-8), 74.3 (C-3), 59.2 (C-1), 55.9 (q, $^4J_{C,F}$ = 1.7 Hz, C-12), 38.9 (C-4), 36.7 (C-2), 26.0 (3C, TBS), 18.4 (TBS), -5.4 (2C, TBS); **HRMS (ESI+)** *m/z*: $[M+H]^+$ calcd. for $C_{22}H_{33}F_3O_4SiH^+$ 447.2177, found 447.2173.

Synthesis of compound S-10

(*S*)-1-((*tert*-Butyldimethylsilyl)oxy)hex-5-en-3-yl (*S*)-3,3,3-trifluoro-2-methoxy-2-phenylpropanoate

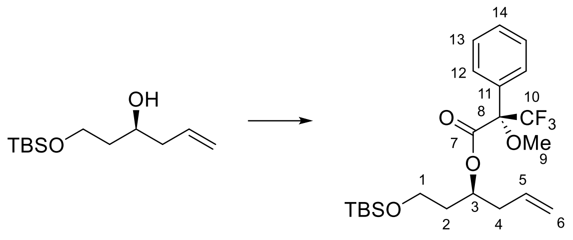

S-8 **S-10**

$C_{22}H_{33}F_3O_4Si$

446.58 g/mol

Pyridine (20.0 µL, 248 µmol, 11.2 eq.) and (*R*)-MTPA-Cl (12.0 µL, 64.1 µmol, 2.9 eq.) were added to alcohol **S-8** (5.10 mg, 22.1 µmol, 1.0 eq.) in dry DCM (0.6 mL). After stirring for 16 h at room temperature, water (1.5 mL) and DCM (1 mL) were added, the phases were separated and the aqueous phase was extracted with DCM (4 x 2 mL). The combined organic phases were dried over $MgSO_4$ and the solvent was removed under reduced pressure. Purification by flash column chromatography (SiO_2, cyclohexane/ethyl acetate 19:1) gave MOSHER ester **S-10** (8.50 mg, 19.0 µmol, 86%) as colorless oil.

R_f: 0.54 (cyclohexane/ethyl acetate 9:1); **$[\alpha]_D^{20}$** = -0.5° (c = 1.90, $CHCl_3$); **^{1}H-NMR** (500 MHz, CD_2Cl_2, 298 K): δ [ppm] = 7.53 – 7.51 (m, 2H, H-12), 7.43 – 7.39 (m, 3H, H-13, H-14), 5.71 – 5.63 (m, 1H, H-5), 5.34 – 5.29 (m, 1H, H-3), 5.05 – 5.01 (m, 2H, H-6), 3.71 – 3.62 (m, 2H, H-1), 3.52 (q, 3H, $^5J_{9,F}$ = 1.2 Hz, H-9), 2.47 – 2.34 (m, 2H, H-4), 1.90 – 1.77 (m, 2H, H-2), 0.89 (s, 9H, TBS), 0.04 (s, 6H, TBS); **^{13}C-NMR** (126 MHz, CD_2Cl_2, 298 K): δ [ppm] = 166.3 (C-7), 133.3 (C-5), 132.8 (C-11), 130.0 (C-14), 128.7 (2C, C-13), 127.9 (2C, C-12), 123.9 (q, 1C, $^1J_{C,F}$ = 288 Hz, C-10), 118.5 (C-6), 85.1 (q, 1C, $^2J_{C,F}$ = 27.5 Hz, C-8), 74.2 (C-3), 59.3 (C-1), 55.8 (q, $^4J_{C,F}$ = 1.7 Hz, C-12), 38.6 (C-4), 36.6 (C-2), 26.0 (3C, TBS), 18.5 (TBS), -5.4 (2C, TBS); **HRMS (ESI+)** *m*/*z*: $[M+H]^+$ calcd. for $C_{22}H_{33}F_3O_4SiH^+$ 447.2177, found 447.2173.

Table 2: Relevant ^{1}H-NMR signals for the MOSHER ester analysis of esters **S-9** and **S-10**.

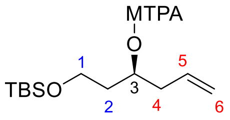

H	δ^{S} [ppm]	δ^{R} [ppm]	$\Delta\delta^{SR}$ [ppm]
6	5.03	5.12	-0.09
5	5.67	5.80	-0.13
4	2.41	2.48	-0.07
2	1.84	1.79	+0.05
1	3.66	3.52	+0.14

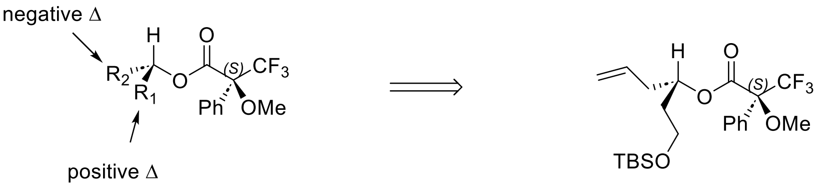

Scheme 2: MOSHER ester analysis confirmed the desired configuration.

For multiplets, the centers of the signals were selected to calculate the differences. The enantiomeric excess was determined by analysis of the ^{1}H-NMR spectra of the MOSHER esters **S-9** and **S-10** to be *e.e.* = 92% and *e.r.* = 26:1.

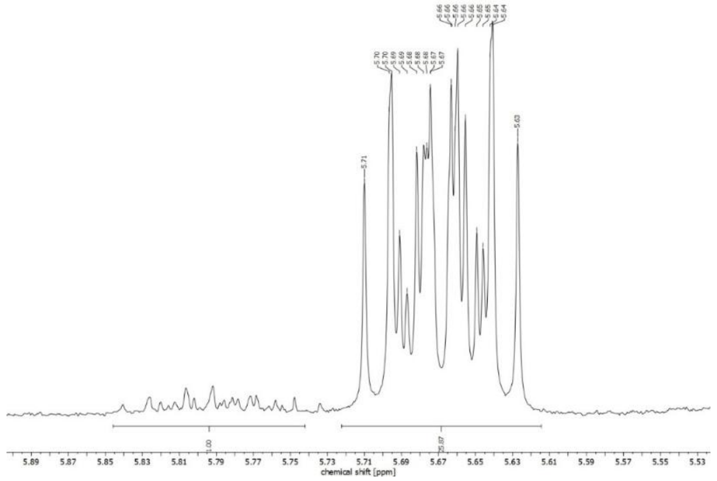

Figure 2: Excerpt of the ^{1}H-NMR spectra of compound **S-10**.

Synthesis of compound S-11

(*S*)-5-Allyl-2,2,3,3,9,9,10,10-octamethyl-4,8-dioxa-3,9-disilaundecane

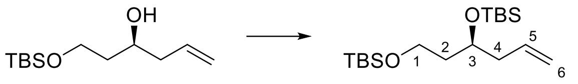

S-8 S-11

$C_{18}H_{40}O_2Si_2$

344.69 g/mol

2,6-Lutidine (940 µL, 8.12 mmol, 2.0 eq.) and TBSOTf (1.40 mL, 6.09 mmol, 1.5 eq.) were added dropwise to alcohol **S-8** (935 mg, 4.06 mmol, 1.0 eq.) in dry DCM (25 mL) at 0 °C. After stirring for 2 h, NH_4Cl-solution (aq., sat., 30 mL) was added. The phases were separated and the aqueous phase was extracted with DCM (3 x 30 mL). The combined organic phases were dried over $MgSO_4$ and all volatiles were removed under reduced pressure. After flash column chromatography (SiO_2, cyclohexane/ethyl acetate 30:1 → 15:1), product **S-11** (1.36 g, 3.96 mmol, 98%) was obtained as a colorless oil.

R_f: 0.69 (cyclohexane/ethyl acetate 9:1); **$[\alpha]_D^{20}$** = +20.6° (c = 1.07, $CHCl_3$); **^{1}H-NMR** (500 MHz, CD_2Cl_2, 298 K): δ [ppm] = 5.83 (ddt, 1H, $^3J_{5,6E}$ = 17.3 Hz, $^3J_{5,6Z}$ = 10.3 Hz, $^3J_{4,5}$ = 7.2 Hz, H-5), 5.06 – 5.01 (m, 2H, H-6), 3.89 (tt, 1H, $^3J_{3,4}$ = 6.8 Hz, $^3J_{2,3}$ = 5.2 Hz, H-3), 3.67 (td, 2H, $^3J_{1,2}$ = 6.5 Hz, $^4J_{1,3}$ = 1.8 Hz, H-1), 2.29 – 2.17 (m, 2H, H-4), 1.68 – 1.57 (m, 2H, H-2), 0.89 (s, 18H, TBS), 0.06 (s, 6H, TBS), 0.04 (s, 6H, TBS); **^{13}C-NMR** (126 MHz, CD_2Cl_2, 298 K): δ [ppm] = 135.8 (C-5), 116.9 (C-6), 69.3 (C-3), 60.2 (C-1), 42.5 (C-4), 40.2 (C-2), 26.1 (s, 3C, TBS), 26.1 (s, 3C, TBS), 18.5 (TBS), 18.4 (TBS), -4.3 (TBS), -4.6 (TBS), -5.2 (2C, TBS); **HRMS (ESI+)** *m/z*: $[M + H]^+$ calcd. for $C_{18}H_{40}O_2Si_2H^+$ 345.2640, found 345.2643.

Synthesis of compound S-12

(*R*)-3,5-Bis((*tert*-butyldimethylsilyl)oxy)pentanal

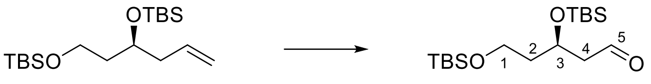

S-11 S-12

$C_{17}H_{38}O_3Si_2$

348.67 g/mol

Ozone was bubbled through a solution of alkene **S-11** (4.40 g, 12.8 mmol, 1.0 eq.) in DCM (75 mL) and methanol (5 mL) at –78 °C until a blue color appeared. Afterwards, argon was bubbled through to expel the excess ozone and PPh_3 (4.20 g, 16.0 mmol, 1.25 eq.) was added. After stirring for 2.5 h at room temperature, the crude product was concentrated onto Celite®. Afterwards, flash column chromatography (SiO_2, cyclohexane/ethyl acetate 19:1 → 9:1) yielded product **S-12** (3.92 g, 11.3 mmol, 88%) as a colorless oil.

R$_f$: 0.38 (cyclohexane/ethyl acetate 19:1); **[α]$_D^{20}$** = +8.8° (c = 1.14, $CHCl_3$); **^{1}H-NMR** (700 MHz, CD_2Cl_2, 298 K): *δ* [ppm] = 9.78 (dd, 1H, $^3J_{4b,5}$ = 3.0 Hz, $^3J_{4a,5}$ = 2.0 Hz, H-5), 4.37 (tdd, 1H, $^3J_{3,4b}$ = 6.3 Hz, $^3J_{2,3}$ = 6.2 Hz, $^3J_{3,4a}$ = 5.1 Hz, H-3), 3.69 (t, 2H, $^3J_{1,2}$ = 6.2 Hz, H-1), 2.59 (ddd, 1H, $^2J_{4a,4b}$ = 15.7 Hz, $^3J_{3,4a}$ = 5.1 Hz, $^3J_{4a,5}$ = 2.0 Hz, H-4a), 2.50 (ddd, 1H, $^2J_{4a,4b}$ = 15.7 Hz, $^3J_{3,4b}$ = 6.3 Hz, $^3J_{4b,5}$ = 3.0 Hz, H-4b), 1.78 (ddt, 1H, $^2J_{2a,2b}$ = 13.9 Hz, $^3J_{1,2a}$ = 6.2 Hz, $^3J_{2a,3}$ = 6.2 Hz, H-2a), 1.70 (dq, 1H, $^2J_{2a,2b}$ = 13.9 Hz, $^3J_{1,2b}$ = 6.2 Hz, $^3J_{2b,3}$ = 6.2 Hz, H-2b), 0.89 (s, 9H, TBS), 0.87 (s, 9H, TBS), 0.09 (s, 3H, TBS), 0.06 (s, 3H, TBS), 0.05 (s, 6H, TBS); **^{13}C-NMR** (176 MHz, CD_2Cl_2, 298 K): *δ* [ppm] = 202.4 (C-5), 66.0 (C-3), 59.6 (C-1), 51.5 (C-4), 41.0 (C-2), 26.1 (3C, TBS), 25.9 (3C, TBS), 18.5 (TBS), 18.3 (TBS), -4.4 (TBS), -4.6 (TBS), -5.3 (TBS), -5.3 (TBS); **HRMS (ESI+)** *m/z*: [M + H]$^+$ calcd. for $C_{17}H_{38}O_3Si_2H^+$ 347.2432, found 347.2437.

Synthesis of compound S-13

Ethyl (*S,E*)-5,7-bis((*tert*-butyldimethylsilyl)oxy)-2-methylhept-2-enoate

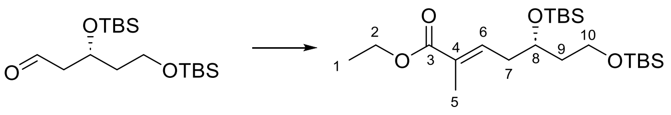

S-12

S-13

$C_{22}H_{46}O_4Si_2$

430.78 g/mol

Triethyl-2-phosphonopropionate (230 µL, 1.07 mmol, 1.4 eq.) was added to $Ba(OH)_2$ (500 mg, 2.92 mmol, 3.9 eq.) in dry THF (6.0 mL). After stirring for 50 min at room temperature, aldehyde **S-12** (260 mg, 750 µmol, 1.0 eq.), dissolved in THF/H_2O (40:1, 2.0 mL), was added. After stirring for 2 h at room temperature, NH_4Cl-solution (aq., sat., 10 mL) was added and the phases were separated. The aqueous phase was extracted with Et_2O (3 × 20 mL), the combined organic layers were dried over $MgSO_4$

and the solvents were removed under reduced pressure. The crude product was purified by flash column chromatography (SiO_2, pentane/diethyl ether 20:1) and the product **S-13** was obtained as a colorless oil (293 mg, 681 µmol, 91%).

R*f*: 0.32 (cyclohexane/ethyl acetate 19:1); **$[\alpha]_D^{20}$** = +7.2° (c = 0.97, $CHCl_3$); **^{1}H-NMR** (500 MHz, CD_2Cl_2, 298 K): *δ* [ppm] = 6.81 (tq, 1H, $^3J_{6,7}$ = 7.4 Hz, $^4J_{5,6}$ = 1.4 Hz, H-6), 4.15 (q, 2H, $^3J_{1,2}$ = 7.1 Hz, H-2), 3.98 (p, 1H, $^3J_{7,8}$ = 5.9 Hz, $^3J_{8,9}$ = 5.9 Hz, H-8), 3.68 (t, 2H, $^3J_{9,10}$ = 6.4 Hz, H-10), 2.41 – 2.30 (m, 2H, H-7), 1.82 (d, 3H, $^4J_{5,6}$ = 1.4 Hz, H-5), 1.69 – 1.62 (m, 2H, H-9), 1.27 (t, 3H, $^3J_{1,2}$ = 7.1 Hz, H-1), 0.89 (s, 9H, TBS), 0.88 (s, 9H, TBS), 0.07 (s, 3H, TBS), 0.06 (s, 3H, TBS), 0.04 (s, 6H, TBS); **^{13}C-NMR** (126 MHz, CD_2Cl_2, 298 K): *δ* [ppm] = 168.2 (C-3), 139.0 (C-6), 129.4 (C-4), 69.0 (C-8), 60.7 (C-2), 60.0 (C-10), 40.9 (C-9), 37.2 (C-7), 26.1 (3C, TBS), 26.0 (3C, TBS), 18.5 (TBS), 18.3 (TBS), 14.5 (C-1), 12.8 (C-5), -4.4 (TBS), -4.5 (TBS), -5.2 (2C, TBS); **HRMS (ESI+)** *m/z*: [M + H]$^+$ calcd. for $C_{22}H_{46}O_4Si_2H^+$ 431.3007, found 431.3006.

Synthesis of compound S-14

(*S*,*E*)-5,7-Bis((*tert*-butyldimethylsilyl)oxy)-2-methylhept-2-en-1-ol

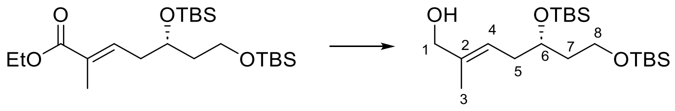

S-13

S-14
$C_{20}H_{44}O_3Si_2$
388.74 g/mol

DIBAL-H (1.0 M in cyclohexane, 1.75 mL, 1.75 mmol, 2.7 eq.) was added to ester **S-13** (275 mg, 639 µmol, 1.0 eq.) in dry THF (8.0 mL) at –78 °C. After stirring for 2 h at -78 °C, Rochelle-salt-solution (aq., sat., 8.0 mL) was added and stirring at room temperature continued until the two phases clearly separated (3 h). The phases were separated and the aqueous phase was extracted with Et_2O (3 x 10 mL). The combined organic layers were dried over $MgSO_4$ and all volatiles were removed under reduced pressure. The crude product was purified by flash column chromatography (SiO_2, cyclohexane/ethyl acetate 9:1 → 7:1), yielding product **S-14** as a colorless oil (228 mg, 586 µmol, 92%).

R*f*: 0.20 (cyclohexane/ethyl acetate 9:1); **[α]$_D$^{20}** = +17.2° (c = 1.16, $CHCl_3$); **^{1}H-NMR** (500 MHz, CD_2Cl_2, 298 K): *δ* [ppm] = 5.45 (tq, 1H, $^3J_{4,5}$ = 7.3 Hz, $^4J_{3,4}$ = 1.4 Hz, H-4), 3.98 (s, 1H, H-1a), 3.97 (s, 1H, H-1b), 3.88 (dtd, 1H, $^3J_{6,7a}$ = 7.0 Hz, $^3J_{6,7b}$ = 5.9 Hz, $^3J_{5,6}$ = 4.9 Hz, H-6), 3.70 – 3.63 (m, 2H, H-8), 2.22 (ddt, 2H, $^3J_{4,5}$ = 7.3 Hz, $^3J_{5,6}$ = 4.9 Hz, $^4J_{5,7}$ = 0.9 Hz, H-5), 1.67 – 1.60 (m, 2H, H-7), 1.65 (d, 3H, $^4J_{3,4}$ = 1.4 Hz, H-3), 1.31 (br s, 1H, H-1), 0.89 (s, 9H, TBS), 0.88 (s, 9H, TBS), 0.06 (s, 3H, TBS), 0.05 (s, 3H, TBS), 0.04 (s, 6H, TBS); **^{13}C-NMR** (126 MHz, CD_2Cl_2, 298 K): *δ* [ppm] = 136.9 (C-2), 122.4 (C-4), 69.6 (C-6), 69.1 (C-1), 60.3 (C-8), 40.4 (C-7), 36.2 (C-5), 26.1 (3C, TBS), 26.0 (3C, TBS), 18.5 (TBS), 18.4 (TBS), 14.0 (C-3), -4.3 (TBS), -4.6 (TBS), -5.2 (2C, TBS); **HRMS (ESI+)** *m/z*: [M + H]$^+$ calcd. for $C_{20}H_{44}O_3Si_2H^+$ 389.2902, found 389.2900.

Synthesis of compound S-15

(*S*,*E*)-5-(4-((4-Methoxybenzyl)oxy)-3-methylbut-2-en-1-yl)-2,2,3,3,9,9,10,10-octamethyl-4,8-dioxa-3,9-disilaundecane

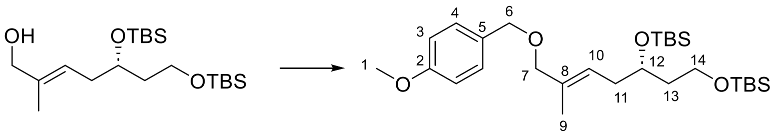

S-14 **S-15**

$C_{28}H_{52}O_4Si_2$

508.89 g/mol

PMBCl (750 µL, 5.56 mmol, 1.5 eq.) and NaH (60% in mineral oil, 185 mg, 4.63 mmol, 1.2 eq.) were added to alcohol **S-14** (1.47 g, 3.78 mmol, 1.0 eq.) in dry DMF (40 mL) at 0 °C. After stirring for 18 h at room temperature, NH_4Cl-solution (aq., sat., 30 mL) was added and stirring continued for 20 min. Water (50 mL) and DCM (100 mL) were added and the phases were separated. The aqueous phase was extracted with DCM (3 x 100 mL), the combined organic layers were dried over $MgSO_4$ and all volatiles were removed under reduced pressure. The crude product was purified by flash column chromatography (SiO_2, cyclohexane/ethyl acetate 20:1), furnishing product **S-15** as a colorless oil (1.68 g, 3.29 mmol, 87%).

R*f*: 0.30 (cyclohexane/ethyl acetate 19:1); **[α]$_D$^{20}** = +13.0° (c = 0.92, $CHCl_3$); **^{1}H-NMR** (500 MHz, CD_2Cl_2, 298 K): *δ* [ppm] = 7.24 (d, 2H, $^3J_{3,4}$ = 8.8 Hz, H-4), 6.86 (d, 2H, $^3J_{3,4}$ = 8.8 Hz, H-3), 5.48 (tq, 1H, $^3J_{10,11}$ = 7.4 Hz, $^4J_{9,10}$ = 1.4 Hz, H-10), 4.34 (s, 2H,

H-6), 3.91 – 3.86 (m, 1H, H-12), 3.87 (s, 2H, H-7), 3.79 (s, 3H, H-1), 3.70 – 3.65 (m, 2H, H-14), 2.24 (ddt, 2H, $^3J_{10,11}$ = 7.4 Hz, $^3J_{11,12}$ = 5.8 Hz, $^4J_{11,13}$ = 0.8 Hz, H-11), 1.68 – 1.59 (m, 2H, H-13), 1.66 (d, 3H, $^4J_{9,10}$ = 1.4 Hz, H-9), 0.89 (s, 9H, TBS), 0.88 (s, 9H, TBS), 0.06 (s, 3H, TBS), 0.06 (s, 3H, TBS), 0.04 (s, 3H, TBS), 0.03 (s, 3H, TBS); **^{13}C-NMR** (126 MHz, CD_2Cl_2, 298 K): *δ* [ppm] = 159.5 (C-2), 134.3 (C-8), 131.4 (C-5), 129.6 (2C, C-4), 124.5 (C-10), 114.0 (2C, C-3), 76.4 (C-7), 71.4 (C-6), 69.5 (C-12), 60.3 (C-14), 55.6 (C-1), 40.4 (C-13), 36.3 (C-11), 26.1 (3C, TBS), 26.1 (3C, TBS), 18.5 (TBS), 18.4 (TBS), 14.3 (C-9), -4.3 (TBS), -4.6 (TBS), -5.2 (2C, TBS); **HRMS (ESI+)** *m/z*: $[M + H]^+$ calcd. for $C_{28}H_{52}O_4Si_2H^+$ 509.3477, found 509.3474.

Synthesis of compound S-16

(*S*,*E*)-7-((4-Methoxybenzyl)oxy)-6-methylhept-5-ene-1,3-diol

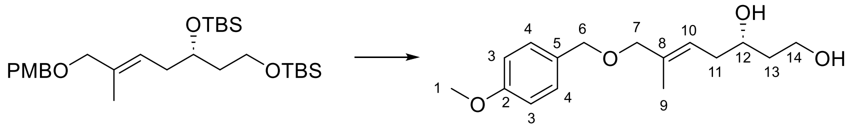

S-15 **S-16**

$C_{16}H_{24}O_4$
280.36 g/mol

TBAF (1.0 M in THF, 5.00 mL, 5.00 mmol, 6.0 eq.) was added to TBS-ether **S-15** (427 mg, 839 µmol, 1.0 eq.) in dry THF (10.0 mL). After stirring for 16 h at room temperature, NH_4Cl-solution (aq., sat., 30 mL) was added and the phases were separated. The aqueous phase was extracted with EtOAc (5 x 30 mL) and the combined organic layers were dried over Na_2SO_4. After removal of all volatiles under reduced pressure, the crude product was purified by flash column chromatography (SiO_2, cyclohexane/ethyl acetate 1:1 → 0:100), yielding the product **S-16** as a colorless oil (232 mg, 826 µmol, 98%).

R_f: 0.22 (ethyl acetate); **$[\alpha]_D^{20}$** = -2.9° (c = 1.02, $CHCl_3$); **^{1}H-NMR** (700 MHz, CD_2Cl_2, 298 K): *δ* [ppm] = 7.25 (d, 2H, $^3J_{3,4}$ = 8.8 Hz, H-4), 6.87 (d, 2H, $^3J_{3,4}$ = 8.8 Hz, H-3), 5.47 (tq, 1H, $^3J_{10,11}$ = 7.5 Hz, $^4J_{9,10}$ = 1.4 Hz, H-10), 4.37 (s, 2H, H-6), 3.89 (s, 2H, H-7), 3.89 – 3.85 (m, 1H, H-12), 3.83 – 3.80 (m, 1H, H-14a), 3.79 (s, 3H, H-1), 3.79 – 3.75 (m, 1H, H-14b), 2.34 (d, 1H, $^3J_{12,OH}$ = 3.3 Hz, H-OH), 2.30 – 2.21 (m, 2H, H-11), 2.21 (t, 1H, $^3J_{14,OH}$ = 5.3 Hz, H-OH), 1.73 – 1.69 (m, 1H, H-13a), 1.68 (d, 3H,

$^{4}J_{9,10}$ = 1.4 Hz, H-9), 1.68 – 1.63 (m, 1H, H-13b); **^{13}C-NMR** (176 MHz, CD_2Cl_2, 298 K): δ [ppm] = 159.6 (C-2), 136.0 (C-8), 131.2 (C-5), 129.7 (2C, C-4), 123.3 (C-10), 114.0 (2C, C-3), 76.1 (C-7), 72.1 (C-12), 71.8 (C-6), 62.0 (C-14), 55.6 (C-1), 38.5 (C-13), 36.6 (C-11), 14.4 (C-9); **HRMS (ESI+)** *m/z*: [M + Na]$^+$ calcd. for $C_{16}H_{24}O_4Na^+$ 303.1567, found 303.1564.

Synthesis of compound S-17

(*S*,*E*)-3,3,9,9-Tetraethyl-5-(4-((4-methoxybenzyl)oxy)-3-methylbut-2-en-1-yl)-4,8-dioxa-3,9-disilaundecane

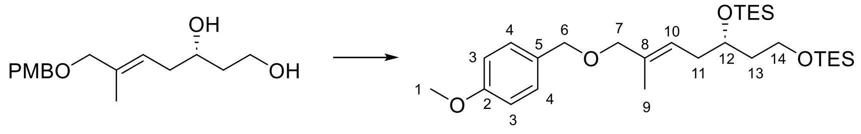

S-16

S-17
$C_{28}H_{52}O_4Si_2$
508.89 g/mol

Imidazole (2.42 g, 35.5 mmol, 6.1 eq.), DMAP (152 mg, 1.24 mmol, 0.21 eq.) and TESCl (4.00 mL, 23.8 mmol, 4.1 eq.) were added to diol **S-16** (1.63 g, 5.81 mmol, 1.0 eq.) in dry THF (70 mL). After stirring for 17 h at room temperature, the white-opaque suspension was diluted with NH_4Cl-solution (aq., sat., 50 mL) and water (20 mL). The phases were separated and the aqueous phase was extracted with Et_2O (3 x 100 mL). The combined organic layers were dried over $MgSO_4$ and all volatiles were removed under reduced pressure. The crude product was purified by flash column chromatography (SiO_2, cyclohexane/ethyl acetate 20:1), yielding product **S-17** as a colorless oil (2.98 g, max. 5.81 mmol, quant.).

R$_f$: 0.47 (cyclohexane/ethyl acetate 9:1); **$[\alpha]_D^{20}$** = +9.2° (c = 0.98, $CHCl_3$); **^{1}H-NMR** (500 MHz, CD_2Cl_2, 298 K): δ [ppm] = 7.25 (d, 2H, $^{3}J_{3,4}$ = 8.6 Hz, H-4), 6.86 (d, 2H, $^{3}J_{3,4}$ = 8.6 Hz, H-3), 5.48 (tq, 1H, $^{3}J_{10,11}$ = 7.2 Hz, $^{4}J_{9,10}$ = 1.4 Hz, H-10), 4.35 (s, 2H, H-6), 3.92 – 3.87 (m, 1H, H-12), 3.87 (s, 2H, H-7), 3.79 (s, 3H, H-1), 3.67 (td, 2H, $^{3}J_{13,14}$ = 6.4 Hz, $^{4}J_{12,14}$ = 1.5 Hz, H-14), 2.24 (dd, 2H, $^{3}J_{10,11}$ = 7.2 Hz, $^{3}J_{11,12}$ = 6.6 Hz, H-11), 1.70 – 1.58 (m, 2H, H-13), 1.66 (d, 3H, $^{4}J_{9,10}$ = 1.4 Hz, H-9), 0.96 (t, 9H, $^{3}J_{TES,TES}$ = 7.9 Hz, TES), 0.95 (t, 9H, $^{3}J_{TES,TES}$ = 7.9 Hz, TES), 0.60 (q, 6H, $^{3}J_{TES,TES}$ = 7.9 Hz, TES), 0.58 (q, 6H, $^{3}J_{TES,TES}$ = 7.9 Hz, TES); **^{13}C-NMR** (126 MHz,

CD_2Cl_2, 298 K): *δ* [ppm] = 159.5 (C-2), 134.4 (C-8), 131.4 (C-5), 129.6 (2C, C-4), 124.4 (C-10), 114.0 (2C, C-3), 76.4 (C-7), 71.4 (C-6), 69.6 (C-12), 60.0 (C-14), 55.6 (C-1), 40.6 (C-13), 36.4 (C-11), 14.3 (C-9), 7.1 (3C, TES), 7.0 (3C, TES), 5.4 (3C, TES), 4.8 (3C, TES); **HRMS (ESI+)** *m/z*: $[M + Na]^+$ calcd. for $C_{28}H_{52}O_4Si_2Na^+$ 531.3296, found 531.3295.

Synthesis of compound S-18

(*S,E*)-7-((4-Methoxybenzyl)oxy)-6-methyl-3-((triethylsilyl)oxy)hept-5-en-1-ol

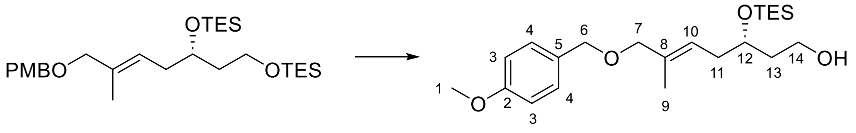

S-17 **S-18**

$C_{22}H_{38}O_4Si$
394.63 g/mol

TBAF (1.0 M in THF, 3.50 mL, 3.50 mmol, 1.02 eq.) was added to TES-ether **S-17** (1.74 g, 3.42 mmol, 1.0 eq.) in THF/HOAc (10:1, 66 mL) at –30 °C. After keeping the temperature between –30 °C and –15 °C for 5 h, $NaHCO_3$-solution (aq., sat., 100 mL) was added and the biphasic mixture was stirred for 30 min at room temperature. The phases were separated and the aqueous phase was extracted with Et_2O (3 x 75 min). The combined organic layers were dried over Na_2SO_4 and all volatiles were removed under reduced pressure. The crude product was purified by flash column chromatography (SiO_2, cyclohexane/ethyl acetate 9:1 → 4:1), yielding product **S-18** as a colorless oil (1.06 g, 2.67 mmol, 78%).

R_f: 0.20 (cyclohexane/ethyl acetate 4:1); **$[\alpha]_D^{20}$** = +20.3° (c = 0.74, $CHCl_3$); **^{1}H-NMR** (500 MHz, CD_2Cl_2, 298 K): *δ* [ppm] = 7.24 (d, 2H, $^3J_{3,4}$ = 8.6 Hz, H-4), 6.86 (d, 2H, $^3J_{3,4}$ = 8.6 Hz, H-3), 5.47 (tq, 1H, $^3J_{10,11}$ = 7.3 Hz, $^4J_{9,10}$ = 1.4 Hz, H-10), 4.35 (s, 2H, H-6), 3.97 (tdd, 1H, $^3J_{12,13b}$ = 7.1 Hz, $^3J_{11,12}$ = 5.6 Hz, $^3J_{12,13a}$ = 4.0 Hz, H-12), 3.87 (s, 2H, H-7), 3.79 (s, 3H, H-1), 3.79 – 3.73 (m, 1H, H-14a), 3.70 – 3.64 (m, 1H, H-14b), 2.32 – 2.28 (m, 2H, H-11), 2.15 (t, 1H, $^3J_{14,OH}$= 5.3 Hz, H-OH), 1.76 (dddd, 1H, $^2J_{13a,13b}$ = 14.2 Hz, $^3J_{13a,14}$ = 7.8 Hz, $^3J_{13a,14}$ = 5.2 Hz, $^3J_{12,13a}$ = 4.0 Hz, H-13a), 1.66 (d, 3H, $^4J_{9,10}$ = 1.4 Hz, H-9), 1.62 (dddd, 1H, $^2J_{13a,13b}$ = 14.2 Hz, $^3J_{12,13b}$ = 7.1 Hz, $^3J_{13b,14}$ = 6.1 Hz, $^3J_{13b,14}$ = 4.7 Hz, H-13b), 0.97 (t, 9H, $^3J_{TES,TES}$ = 7.9 Hz, TES), 0.63 (q,

6H, $^3J_{TES,TES}$ = 7.9 Hz, TES); **^{13}C-NMR** (126 MHz, CD_2Cl_2, 298 K): δ [ppm] = 159.6 (C-2), 134.8 (C-8), 131.3 (C-5), 129.6 (2C, C-4), 123.6 (C-10), 114.0 (2C, C-3), 76.2 (C-7), 71.9 (C-12), 71.6 (C-6), 60.6 (C-14), 55.6 (C-1), 38.6 (C-13), 36.1 (C-11), 14.3 (C-9), 7.0 (3C, TES), 5.3 (3C, TES); **HRMS (ESI+)** *m/z*: [M + Na]$^+$ calcd. for $C_{22}H_{38}O_4SiNa^+$ 417.2432, found 417.2429.

Synthesis of compound S-19

Diethyl ((4*S*,*E*)-2-hydroxy-8-((4-methoxybenzyl)oxy)-7-methyl-4-((triethylsilyl)oxy)oct-6-en-1-yl)phosphonate

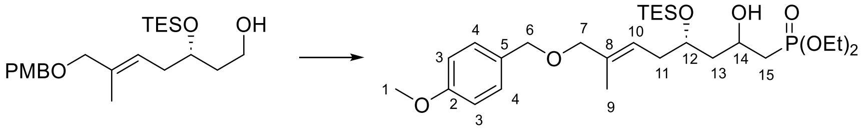

S-18

S-19
$C_{27}H_{49}O_7PSi$
544.74 g/mol

To a suspension of py·SO_3 (498 mg, 3.13 mmol, 3.0 eq.), DIPEA (1.00 mL, 5.88 mmol, 5.7 eq.) and DMSO (730 µL, 10.3 mmol, 10.0 eq.) in dry DCM (15.0 mL) was added alcohol **S-18** (408 mg, 1.03 mmol, 1.0 eq.) in dry DCM (5.0 mL) at 0 °C. After stirring for 75 min at room temperature, $NaHCO_3$-solution (aq., sat., 20 mL) was added and the phases were separated. The aqueous phase was extracted with DCM (3 x 20 mL) and the combined organic phases were washed with $CuSO_4$-solution (aq., sat., 2 x 100 mL), NH_4Cl-solution (aq., sat., 2 x 100 mL) and brine (100 mL). After drying over $MgSO_4$ and evaporation of all volatiles under reduced pressure, the crude aldehyde was added to a premixed solution of diethylmethylphosphonate (450 µL, 3.08 mmol, 3.0 eq.) and *n*-BuLi (2.5 M in THF, 1.10 mL, 2.75 mmol, 2.7 eq), which was previously stirred for 1 h at –78 °C. After stirring for 45 min at –78 °C, water (15 mL) and NH_4Cl-solution (aq., sat., 5 mL) were added and the biphasic mixture was warmed up to room temperature. The phases were separated and the aqueous phase was extracted with EtOAc (4 x 25 mL). The combined organic layers were dried over Na_2SO_4 and all volatiles were removed under reduced pressure. The crude product was purified by flash column chromatography (SiO_2, cyclohexane/ethyl acetate 1:1 → 0:100) and product **S-19** was obtained as a colorless oil (417 mg, 765 µmol, 74%).

R_f: 0.30 (ethyl acetate); **$[\alpha]_D^{20}$** = +9.7° (c = 0.72, $CHCl_3$); **^{1}H-NMR** (500 MHz, CD_2Cl_2, 298 K): δ [ppm] = 7.25 (d, 2H, $^3J_{3,4}$ = 8.6 Hz, H-4), 6.86 (d, 2H, $^3J_{3,4}$ = 8.6 Hz, H-3), 5.48 – 5.42 (m, 1H, H-10), 4.35 (s, 2H, H-6), 4.19 – 3.97 (m, 6H, H-12, H-14, OEt), 3.87 (s, 2H, H-7), 3.79 (s, 3H, H-1), 3.78/3.72 (d, 1H, $^3J_{14,OH}$ = 1.8 Hz, H-OH), 2.34 – 2.24 (m, 2H, H-11), 2.00 – 1.78 (m, 2H, H-15), 1.70 – 1.59 (m, 2H, H-13), 1.66 (d, 3H, $^4J_{9,10}$ = 1.3 Hz, H-9), 1.30 (2 x t, 6H, $^3J_{OEt,OEt}$ = 7.1 Hz, OEt), 0.97 (2 x t, 9H, $^3J_{TES,TES}$ = 8.1 Hz, TES), 0.63 (2 x q, 6H, $^3J_{TES,TES}$ = 7.8 Hz, TES); **^{13}C-NMR** (126 MHz, CD_2Cl_2, 298 K): δ [ppm] = 159.5 (C-2), 134.9/134.8 (C-8), 131.3 (C-5), 129.7/129.6 (2C, C-4), 123.7/123.5 (C-10), 114.0 (2C, C-3), 76.3 (C-7), 71.6/71.6 (C-6), 71.3 (d, $^4J_{C,P}$= 1.6 Hz, C-12), 69.8 (d, $^4J_{C,P}$= 2.3 Hz, C-12), 65.4 (d, $^2J_{C,P}$= 4.3 Hz, C-14), 63.7 (d, $^2J_{C,P}$= 4.6 Hz, C-14), 62.1 (d, $^2J_{C,P}$= 6.2 Hz, OEt), 62.0 (d, $^2J_{C,P}$= 7.4 Hz, OEt), 55.6 (C-1), 45.0 (d, $^3J_{C,P}$= 11.4 Hz, C-13), 44.9 (d, $^3J_{C,P}$= 9.8 Hz, C-13), 36.5/36.1 (C-11), 34.9 (d, $^1J_{C,P}$= 31.6 Hz, C-15), 33.9 (d, $^1J_{C,P}$= 31.9 Hz, C-15), 16.6/16.6 (2C, OEt), 14.4/14.3 (C-9), 7.1/7.0 (3C, TES), 5.4/5.3 (3C, TES); **HRMS (ESI+)** *m/z*: $[M + H]^+$ calcd. for $C_{27}H_{49}O_7PsiH^+$ 545.3058, found 545.3058.

Synthesis of compound 33

Diethyl (*S*,*E*)-(8-((4-methoxybenzyl)oxy)-7-methyl-2-oxo-4-((triethylsilyl)oxy)oct-6-en-1-yl)phosphonate

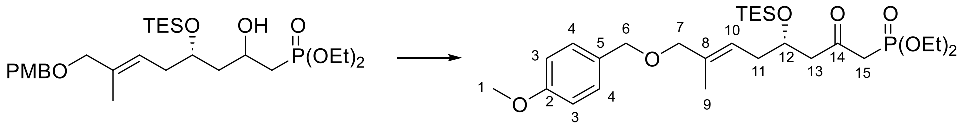

S-19

33

$C_{27}H_{47}O_7PSi$

542.72 g/mol

$NaHCO_3$ (63.5 mg, 756 µmol, 2.0 eq.) and DMP (331 mg, 781 µmol, 2.1 eq.) were added to alcohol **S-19** (205 mg, 376 µmol, 1.0 eq.) in dry DCM (8.0 mL). After stirring for 45 min at room temperature, $Na_2S_2O_3$-solution (aq., sat., 4 mL) was added and stirring continued for 15 min. DCM (10 mL) and water (10 mL) were added and the phases were separated. The aqueous phase was extracted with DCM (3 x 20 mL) and the combined organic phases were dried over Na_2SO_4. After removal of all volatiles under reduced pressure and purification by flash column chromatography (SiO_2,

cyclohexane/ethyl acetate 1:1 → 1:2), product **33** was obtained as a colorless oil (209 mg, 376 μmol, quant.).

R$_f$: 0.42 (ethyl acetate); **[α]$_D^{20}$** = +19.6° (c = 1.02, $CHCl_3$); **^{1}H-NMR** (500 MHz, CD_2Cl_2, 298 K): *δ* [ppm] = 7.25 (d, 2H, $^3J_{3,4}$ = 8.7 Hz, H-4), 6.86 (d, 2H, $^3J_{3,4}$ = 8.7 Hz, H-3), 5.45 (tq, 1H, $^3J_{10,11}$ = 7.3 Hz, $^4J_{9,10}$ = 1.4 Hz, H-10), 4.35 (s, 2H, H-6), 4.21 (dtd, 1H, $^3J_{12,13a}$ = 6.9 Hz, $^3J_{11,12}$ = 6.0 Hz, $^3J_{12,13b}$ = 5.1 Hz, H-12), 4.12 – 4.05 (m, 4H, OEt), 3.87 (s, 2H, H-7), 3.79 (s, 3H, H-1), 3.08 (dd, 1H, $^2J_{P,15a}$ = 20.0 Hz, $^2J_{15a,15b}$ = 13.6 Hz, H-15a), 3.03 (dd, 1H, $^2J_{P,15b}$ = 20.0 Hz, $^2J_{15a,15b}$ = 13.6 Hz, H-15b), 2.75 (dd, 1H, $^2J_{13a,13b}$ = 16.0 Hz, $^3J_{12,13a}$ = 6.9 Hz, H-13a), 2.67 (dd, 1H, $^2J_{13a,13b}$ = 16.0 Hz, $^3J_{12,13b}$ = 5.1 Hz, H-13b), 2.26 (dd, 2H, $^3J_{10,11}$ = 7.3 Hz, $^3J_{11,12}$ = 6.0 Hz, H-11), 1.65 (d, 3H, $^4J_{9,10}$ = 1.4 Hz, H-9), 1.30 (t, 6H, $^3J_{OEt,OEt}$ = 7.1 Hz, OEt), 0.94 (t, 9H, $^3J_{TES,TES}$ = 7.9 Hz, TES), 0.59 (q, 6H, $^3J_{TES,TES}$ = 7.9 Hz, TES); **^{13}C-NMR** (126 MHz, CD_2Cl_2, 298 K): *δ* [ppm] = 201.3 (d, $^2J_{C,P}$ = 6.4 Hz, C-14), 159.6 (C-2), 135.4 (C-8), 131.3 (C-5), 129.6 (2C, C-4), 123.2 (C-10), 114.0 (2C, C-3), 76.2 (C-7), 71.6 (C-6), 69.1 (C-12), 62.8 (d, $^2J_{C,P}$ = 3.5 Hz, OEt), 62.7 (d, $^2J_{C,P}$ = 3.6 Hz, OEt), 55.6 (C-1), 51.3 (C-13), 44.0 (d, $^1J_{C,P}$ = 126.7 Hz, C-15), 36.3 (C-11), 16.6 (OEt), 16.5 (OEt), 14.3 (C-9), 7.0 (3C, TES), 5.3 (3C, TES); **HRMS (APCI)** *m/z*: [M + Na]$^+$ calcd. for $C_{27}H_{47}O_7PSiNa^+$ 565.2721, found 565.2720.

Synthesis of compound S-20

5-((*tert*-Butyldimethylsilyl)oxy)pentanal

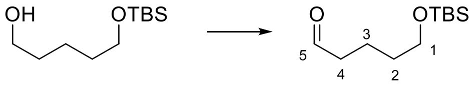

S-19

S-20

$C_{11}H_{24}O_2Si$

216.40 g/mol

DIPEA (1.60 mL, 9.16 mmol, 4.0 eq.), DMSO (1.63 mL, 22.9 mmol, 10.0 eq.) and alcohol **S-19** (500 mg, 2.29 mmol, 1.0 eq.) were added to a cooled suspension of py·SO_3 (1.09 g, 6.87 mmol, 3.0 eq.) in dry DCM (8.0 mL) at 0 °C. After stirring for 1 h at room temperature, $NaHCO_3$-solution was added (aq., sat., 8 mL) and the aqueous layer was extracted with DCM (3 x 5 mL). The combined organic layers were washed with $CuSO_4$-solution (aq., sat., 2 x 15 mL), NH_4Cl-solution (aq., sat., 3 x 15 mL) and

brine (20 mL), dried over $MgSO_4$ and subsequent concentration *in vacuo* yielded aldehyde **S-20** (509 mg, 2.29 mmol, quant.) as a pale-yellow oil which was directly used without further purification.

R_f: 0.38 (cyclohexane/ethyl acetate 9:1); **^{1}H-NMR** (500 MHz, CD_2Cl_2, 298 K): δ [ppm] = 9.73 (t, 1H, $^3J_{4,5}$ = 1.8 Hz, H-5), 3.62 (t, 2H, $^3J_{1,2}$ = 6.2 Hz, H-1), 2.43 (td, 2H, $^3J_{3,4}$ = 7.3 Hz, $^3J_{4,5}$ = 1.8 Hz, H-4), 1.70 – 1.62 (m, 2H, H-2), 1.58 – 1.50 (m, 2H, H-3), 0.89 (s, 9H, TBS), 0.04 (s, 6H, TBS); **^{13}C-NMR** (126 MHz, CD_2Cl_2, 298 K): δ [ppm] = 202.9 (C-5), 63.0 (C-1), 44.0 (C-4), 32.6 (C-2), 26.1 (3C, TBS), 19.0 (C-3), 18.6 (TBS), -5.3 (2C, TBS); **HRMS (APCI)** *m/z*: [M + H]$^+$ calcd. for $C_{11}H_{24}O_2SiH^+$ 217.1618, found 217.1617.

Synthesis of compound S-21

(3*R*,4*S*)-8-((*tert*-Butyldimethylsilyl)oxy)-3-methyloct-1-en-4-ol

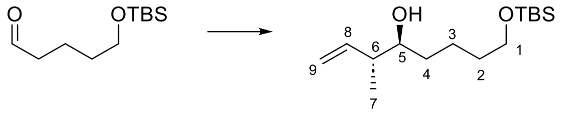

S-20 **S-21**

$C_{15}H_{32}O_2Si$
272.50 g/mol

To a precooled suspension of KO*t*Bu (622 mg, 5.38 mmol, 1.0 eq.) in dry THF (6.6 mL) at –78 °C was added *trans*-butene (1.01 mL, 10.8 mmol, 2.0 eq.) *via* transfer canula before *n*-BuLi (2.5 M in hexane, 2.15 mL, 5.38 mmol, 1.0 eq.) was added within 15 min. The resulting solution was allowed to warm to –50 °C for 20 min at which the color changed from light yellow to orange. Cooling to –78 °C and addition of (+)-Ipc_2BOMe (1.70 g, 5.38 mmol, 1.0 eq.) in dry Et_2O (5.4 mL) within 30 min gave a colorless solution that was stirred for another 30 min. $BF_3{\cdot}Et_2O$ (675 µL, 5.38 mmol, 1.0 eq.) was added dropwise before pre-dried aldehyde **S-20** (3 Å molecular sieves, 1.28 g in 5.9 mL THF, 5.92 mmol, 1.1 eq.) was added over 2 h. Stirring was continued for 3 h until NaOH (aq., 3.0 M, 10 mL), methanol (3.0 mL) and H_2O_2 (35%, 6.0 mL) were added and the biphasic mixture was refluxed for 40 min. The aqueous layer was extracted with Et_2O (3 x 15 mL) and the combined organic layers were washed with $Na_2S_2O_3$-solution (aq., sat., 30 mL), NH_4Cl-solution (aq., sat., 30 mL) and brine (30 mL), dried over $MgSO_4$ and concentrated *in vacuo*. Purification by flash column

chromatography (SiO_2, cyclohexane/ethyl acetate 20:1 → 10:1) yielded homoallyl alcohol **S-21** as a colorless oil (1.05 mg, 3.87 mmol, 72%, *d.r.* > 20:1).

R_f: 0.25 (cyclohexane/ethyl acetate 10:1); **$[\alpha]_D^{20}$** = -2.4° (c = 0.84, CH_2Cl_2); **^{1}H-NMR** (500 MHz, CD_2Cl_2, 298 K): δ [ppm] = 5.83 – 5.72 (m, 1H, H-8), 5.13 – 5.01 (m, 2H, H-9), 3.66 – 3.58 (m, 2H, H-1), 3.42 – 3.33 (m, 1H, H-5), 2.19 (dddt, 1H, $^3J_{6,8}$ = 6.9 Hz, $^3J_{6,7}$ = 6.9 Hz, $^3J_{5,6}$ = 6.8 Hz, $^4J_{6,9}$ = 1.2 Hz, H-6), 1.56 – 1.46 (m, 4H, H-2, H-4), 1.42 – 1.31 (m, 2H, H-3), 1.02 (d, 3H, $^3J_{6,7}$ = 6.9 Hz, H-7), 0.89 (s, 9H, TBS), 0.04 (s, 6H, TBS); **^{13}C-NMR** (126 MHz, CD_2Cl_2 298K): δ [ppm] = 141.0 (C-8), 116.0 (C-9), 75.0 (C-5), 63.5 (C-1), 44.5 (C-6), 34.5 (C-4), 33.3 (C-2), 26.1 (3C, TBS), 22.5 (C-3), 18.6 (TBS), 16.5 (C-7), -5.2 (2C, TBS); **HRMS (ESI+)** *m/z*: $[M + H]^+$ calcd. for $C_{15}H_{32}O_2SiH^+$ 273.2244, found 273.2246.

Synthesis of compound S-22

(3*R*,4*S*)-8-((*tert*-Butyldimethylsilyl)oxy)-3-methyloct-1-en-4-yl (*R*)-3,3,3-trifluoro-2-methoxy-2-phenylpropanoate

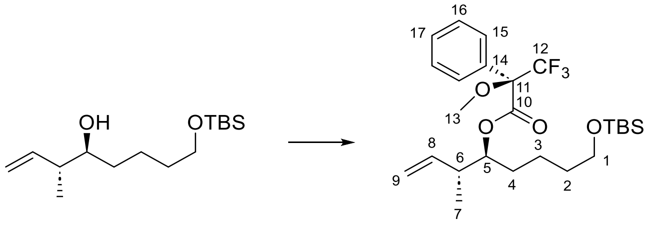

S-21 **S-22**

$C_{25}H_{39}F_3O_4Si$

488.66 g/mol

Pyridine (30.0 µL, 366 µmol, 20.0 eq.) and (*S*)-MTPA-Cl (6.90 µL, 36.7 µmol, 2.0 eq.) were added to a solution of alcohol **S-21** (5.00 mg, 18.4 µmol, 1.0 eq.) in dry DCM (0.5 mL). After stirring at room temperature overnight, water (1.0 mL) and Et_2O (2.0 mL) were added, the phases were separated and the aqueous layer was extracted with Et_2O (3 x 0.5 mL). After drying over $MgSO_4$, concentration *in vacuo* and flash chromatographic purification (SiO_2, cyclohexane/ethyl acetate 2:1), (*R*)-*Mosher* ester **S-22** was obtained as a colorless oil (9.00 mg, 18.4 µmol, quant.).

R_f: 0.54 (cyclohexane/ethyl acetate 10:1); **$[\alpha]_D^{20}$** = -1.1° (c = 0.90, CH_2Cl_2); **^{1}H-NMR** (700 MHz, CD_2Cl_2, 298 K): δ [ppm] = 7.57 – 7.51 (m, 2H, H-16), 7.45 – 7.39 (m, 3H, H-15, H-17), 5.67 (ddd, 1H, $^3J_{8,9E}$ = 17.1 Hz, $^3J_{8,9Z}$ = 10.3 Hz, $^3J_{6,8}$ = 8.0 Hz, H-8), 5.06 (dd, 1H, $^3J_{5,6}$ = 7.7 Hz, $^3J_{4,5}$ = 4.8 Hz, H-9E), 5.04 – 5.00 (m, 2H, H-9Z), 3.58 (t, 2H, $^3J_{1,2}$ = 6.3 Hz, H-1), 3.54 (q, 3H, $^5J_{13,F}$ = 1.3 Hz, H-13), 2.51 – 2.45 (m, 1H, H-6), 1.69 – 1.57 (m, 2H, H-4), 1.54 – 1.45 (m, 2H, H-2), 1.42 – 1.31 (m, 2H, H-3), 0.91 (d, 3H, $^3J_{6,7}$ = 6.9 Hz, H-7), 0.88 (s, 9H, TBS), 0.03 (s, 6H, TBS); **^{13}C-NMR** (176 MHz, CD_2Cl_2, 298 K): δ [ppm] = 166.5 (C-11), 139.0 (C-8), 132.8 (C-14), 130.0 (C-17), 128.7 (2C, C-15), 127.8 (2C, C-16), 123.9 (q, $^1J_{12,F}$ = 288 Hz, C-12), 116.3 (C-9), 84.9 (d, $^2J_{11,F}$ = 27.5 Hz, C-11), 80.7 (C-5), 63.2 (C-1), 55.9 (d, $^4J_{13,F}$ = 1.5 Hz, C-13), 41.2 (C-6), 33.0 (C-4), 31.2 (C-2), 26.1 (3C, TBS), 22.3 (C-3), 18.6 (TBS), 16.1 (C-7), -5.3 (2C, TBS); **HRMS (APCI)** *m/z*: $[M + H]^+$ calcd. for $C_{25}H_{39}O_4F_3SiH^+$ 489.2642, found 489.2638.

Synthesis of compound S-23

(3*R*,4*S*)-8-((*tert*-Butyldimethylsilyl)oxy)-3-methyloct-1-en-4-yl (*S*)-3,3,3-trifluoro-2-methoxy-2-phenylpropanoate

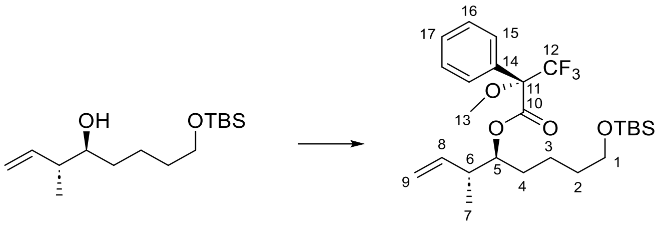

S-21 **S-23**

$C_{25}H_{39}F_3O_4Si$

488.66 g/mol

Pyridine (30.0 µL, 366 µmol, 20.0 eq.) and (*S*)-MTPA-Cl (6.90 µL, 36.7 µmol, 2.0 eq.) were added to a solution of alcohol **S-21** (5.00 mg, 18.4 µmol, 1.0 eq.) in dry DCM (0.5 mL). After stirring at room temperature overnight, water (1.0 mL) and Et_2O (2.0 mL) were added, the phases were separated and the aqueous layer was extracted with Et_2O (3 x 0.5 mL). After drying over $MgSO_4$, concentration *in vacuo* and flash chromatographic purification (SiO_2, cyclohexane/ethyl acetate 2:1), (*S*)-*Mosher* ester **S-23** was obtained as a colorless oil (9.00 mg, 18.4 µmol, quant.).

R_f: 0.54 (cyclohexane/ethyl acetate 10:1); **$[\alpha]_D^{20}$** = +2.2° (c = 0.90, CH_2Cl_2); **^{1}H-NMR** (700 MHz, CD_2Cl_2, 298 K): *δ* [ppm] = 7.55 – 7.53 (m, 2H, H-16), 7.42 – 7.40 (m, 3H, H-15, H-17), 5.75 (ddd, 1H, $^3J_{8,9E}$ = 17.2 Hz, $^3J_{8,9Z}$ = 10.4 Hz, $^3J_{6,8}$ = 7.9 Hz, H-8), 5.10 – 5.04 (m, 3H, H-9), 3.54 – 3.48 (m, 5H, H-1, H-13), 2.57 – 2.50 (m, 1H, H-6), 1.62 – 1.52 (m, 2H, H-4), 1.47 – 1.37 (m, 2H, H-2), 1.26 – 1.17 (m, 2H, H-3), 1.04 (d, 3H, $^3J_{6,7}$ = 6.9 Hz, H-7), 0.88 (s, 9H, TBS), 0.03 (s, 6H, TBS); **^{13}C-NMR** (176 MHz, CD_2Cl_2, 298 K): *δ* [ppm] = 166.6 (C-11), 139.4 (C-8), 132.8 (C-14), 130.0 (C-17), 128.7 (2C, C-15), 127.9 (2C, C-16), 123.9 (q, $^1J_{12,F}$ = 288 Hz, C-12), 116.3 (C-9), 85.0 (d, $^2J_{11,F}$ = 27.5 Hz, C-11), 80.7 (C-5), 63.2 (C-1), 55.9 (d, $^4J_{13,F}$ = 1.5 Hz, C-13), 41.4 (C-6), 32.9 (C-4), 31.0 (C-2), 26.1 (3C, TBS), 21.8 (C-3), 18.6 (TBS), 16.3 (C-7), -5.3 (2C, TBS); **HRMS (APCI)** *m/z*: $[M + H]^+$ calcd. for $C_{25}H_{39}O_4F_3SiH^+$ 489.2642, found 489.2641.

Table 3: Relevant ^{1}H-NMR signals for the MOSHER ester analysis of esters **S-22** and **S-23**.

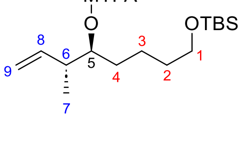

H	δ^S [ppm]	δ^R [ppm]	$\Delta\delta^{SR}$ [ppm]
9	5.08	5.01	+0.07
8	5.75	5.67	+0.08
6	2.53	2.48	+0.05
7	1.04	0.91	+0.13
5	5.06	5.06	0.00
4	1.55	1.62	-0.07
3	1.22	1.37	-0.15
2	1.43	1.51	-0.08
1	3.52	3.58	-0.06

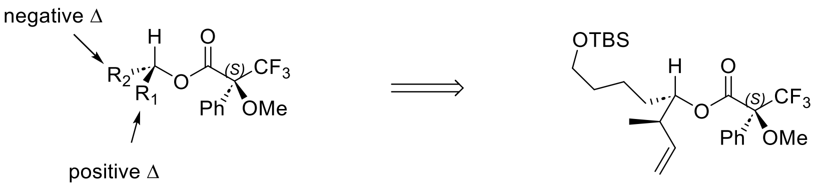

Scheme 3: MOSHER ester analysis confirmed the desired configuration.

For multiplets, the centers of the signals were selected to calculate the differences. The enantiomeric excess was determined by analysis of the ^{1}H NMR spectra of the MOSHER esters **S-22** and **S-23** to be *e.r.* > 20:1.

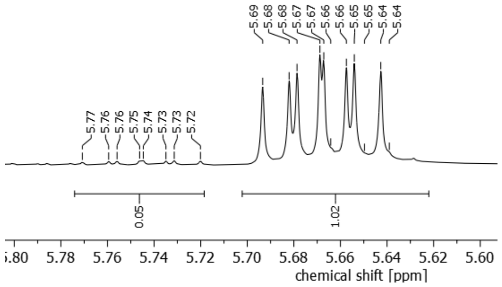

Figure 3: Excerpt of the ^{1}H-NMR spectra of compound **S-22.**

Synthesis of compound S-24

(*S*)-5-((*R*)-But-3-en-2-yl)-2,2,3,3,11,11,12,12-octamethyl-4,10-dioxa-3,11-disilatridecane

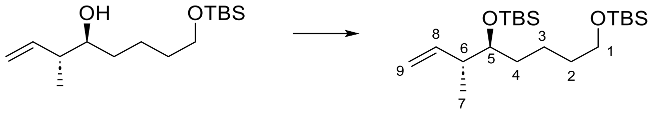

S-21

S-24
$C_{21}H_{46}O_2Si_2$
386.77 g/mol

2,6-Lutidine (1.89 mL, 16.2 mmol, 2.5 eq.) and TBSOTf (2.09 mL, 9.09 mmol, 1.4 eq.) were added to a solution of alcohol **S-21** (1.77 g, 6.50 mmol, 1.0 eq.) in dry DCM/Et_2O (1:1, 33 mL) at 0 °C. After stirring for 40 min at 0 °C, NH_4Cl-solution (aq., sat., 30 mL) was added, the phases were separated and the aqueous layer was extracted with DCM (3 x 20 mL). The combined organic layers were dried over $MgSO_4$ and concentrated *in vacuo*. After flash column chromatography (SiO_2, cyclohexane/ethyl acetate 10:1), silyl ether **S-24** (2.39 g, 6.18 mmol, 95%) was obtained as colorless oil.

R_f: 0.25 (cyclohexane/ethyl acetate 10:1); **$[\alpha]_D^{20}$** = -2.7° (c = 1.12, CH_2Cl_2); **^{1}H-NMR** (400 MHz, CD_2Cl_2, 298 K): δ [ppm] = 5.80 (ddd, 1H, $^3J_{8,9E}$ = 16.7 Hz, $^3J_{8,9Z}$ = 10.9 Hz, $^3J_{6,8}$ = 7.6 Hz, H-8), 5.03 – 4.96 (m, 2H, H-9), 3.61 – 3.54 (m, 3H, H-1, H-5), 2.36 – 2.26 (m, 1H, H-6), 1.52 – 1.42 (m, 2H, H-4), 1.42 – 1.34 (m, 3H, H-2, H-3a), 1.33 – 1.23 (m, 1H, H-3b), 0.99 (d, 3H, $^3J_{6,7}$ = 6.9 Hz, H-7), 0.89 (s, 9H, TBS), 0.89 (s, 9H, TBS), 0.05 (s, 6H, TBS), 0.05 (s, 6H, TBS); **^{13}C-NMR** (126 MHz, CD_2Cl_2, 298 K): δ [ppm] = 141.5 (C-8), 114.4 (C-9), 76.2 (C-5), 63.5 (C-1), 43.5 (C-6), 33.8 (C-4), 33.5 (C-2), 26.1 (3C, TBS), 26.1 (3C, TBS), 22.5 (C-3), 18.6 (TBS), 18.4 (TBS), 15.5 (C-7), -4.2 (TBS), -4.4 (TBS), -5.3 (2C, TBS); **HRMS (APCI)** *m/z*: $[M + H]^+$ calcd. for $C_{21}H_{46}O_2Si_2H^+$ 387.3109, found 387.3111.

Synthesis of compound S-25

(2*S*,3*S*)-3,7-Bis((*tert*-butyldimethylsilyl)oxy)-2-methylheptanal

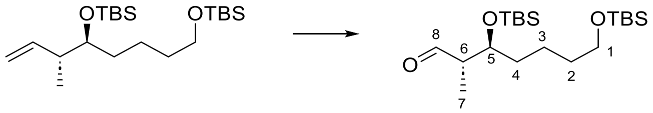

S-24

S-25
$C_{20}H_{44}O_3Si_2$
388.74 g/mol

After cooling a solution of silyl ether **S-24** (241 mg, 623 µmol, 1.0 eq.) in DCM/methanol (10:1, 14.3 mL) to −78 °C, treatment with ozone (ca. 8 min) under vigorous stirring was performed, until a slightly blue color persisted. Subsequently, PPh_3 (245 mg, 934 µmol, 1.5 eq.) was added and the reaction mixture was allowed to warm to room temperature to stir for further 40 min before concentration *in vacuo* (35 °C evaporation temperature, <1 h to avoid elimination of sec. siloxy-function). After flash column chromatography (SiO_2, toluene), aldehyde **S-25** (229 mg, 592 µmol, 95%) was obtained as a colorless oil which was used within 24 h.

R_f: 0.25 (cyclohexane/ethyl acetate 10:1); **$[\alpha]_D^{20}$** = -5.3° (c = 0.44, CH_2Cl_2); **^{1}H-NMR** (700 MHz, CD_2Cl_2, 298 K): δ [ppm] = 9.72 (d, 1H, $^3J_{6,8}$ = 2.2 Hz, H-8), 3.95 (ddd, 1H, $^3J_{4,5}$ = 5.8 Hz, $^3J_{4,5}$ = 5.6 Hz, $^3J_{5,6}$ = 5.0 Hz, H-5), 3.60 (t, 2H, $^3J_{1,2}$ = 6.3 Hz, H-1), 2.50 (qdd, 1H, $^3J_{6,7}$ = 7.0 Hz, $^3J_{5,6}$ = 5.0 Hz, $^3J_{6,8}$ = 2.2 Hz, H-6), 1.61 – 1.55 (m, 1H, H-4a), 1.52 – 1.44 (m, 3H, H-2, H-4b), 1.43 – 1.36 (m, 2H, H-3), 1.05 (d, 3H, $^3J_{6,7}$ = 7.0 Hz, H-7), 0.89 (s, 9H, TBS), 0.88 (s, 9H, TBS), 0.08 (s, 3H, TBS), 0.06 (s, 3H, TBS), 0.04 (s, 6H, TBS); **^{13}C-NMR** (176 MHz, CD_2Cl_2, 298 K): δ [ppm] = 205.1 (C-8), 73.8 (C-5), 63.3 (C-1), 51.6 (C-6), 34.9 (C-4), 33.4 (C-2), 26.1 (3C, TBS), 25.9 (3C, TBS), 21.6 (C-3), 18.6 (TBS), 18.3 (TBS), 10.5 (C-7), -4.1 (TBS), -4.6 (TBS), -5.2 (2C, TBS); **HRMS (APCI)** *m/z*: $[M + H]^+$ calcd. for $C_{20}H_{44}O_3Si_2H^+$ 389.2902, found 389.2907.

Synthesis of compound S-26

(3*S*,4*R*,5*R*,6*S*)-6,10-Bis((*tert*-butyldimethylsilyl)oxy)-3,5-dimethyldec-1-en-4-ol

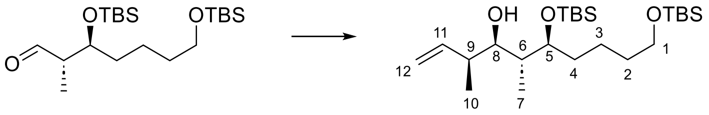

S-25

S-26
$C_{24}H_{52}O_3Si_2$
444.85 g/mol

cis-Butene (550 µL, 5.83 mmol, 5.0 eq.) was added *via* transfer canula to a precooled suspension of KO*t*Bu (161 mg, 1.40 mmol, 1.2 eq.) in dry THF (4.0 mL) at −78°C, followed by addition of *n*-BuLi (2.5 M in hexane, 560 µL, 1.40 mmol, 1.2 eq.) within 15 min. The resulting solution was warmed to –25 °C for 20 min at which the color changed to bright yellow. Re-cooling to –78 °C and addition of (+)-Ipc_2BOMe (460 mg, 1.46 mmol, 1.25 eq.) in dry Et_2O (3.6 mL) within 30 min gave a colorless solution that was stirred for another 30 min. $BF_3{\cdot}Et_2O$ (180 µL, 1.46 mmol, 1.25 eq.), followed by pre-dried aldehyde **S-25** (3 Å molecular sieves, 453 mg in 5.5 mL THF, 1.17 mmol, 1.0 eq.) were added over 90 min. Stirring continued for 120 min until NaOH (aq., 3.0 M, 10 mL), methanol (3.0 mL) and H_2O_2 (35%, 4.0 mL) were added and the biphasic mixture was refluxed for 60 min. The aqueous layer was extracted with Et_2O (3 x 10 mL), the combined organic layers were washed with $Na_2S_2O_3$-solution (aq., sat. 30 mL), NH_4Cl-solution (aq., sat. 30 mL) and brine (30 mL), dried over Na_2SO_4 and concentrated *in vacuo*. Purification by flash column chromatography (SiO_2, cyclohexane/ethyl acetate 40:1 → 20:1) yielded homoallyl alcohol **S-26** as a colorless oil (451 mg, 1.02 mmol, 87%, *d.r.* > 20:1).

R_f: 0.52 (cyclohexane/ethyl acetate 10:1); **$[\alpha]_D^{20}$** = -8.0° (c = 1.00, CH_2Cl_2); **^{1}H-NMR** (700 MHz, CD_2Cl_2, 298 K): *δ* [ppm] = 5.96 – 5.90 (m, 1H, H-11), 5.10 – 5.06 (m, 2H, H-12), 3.93 (ddd, 1H, $^3J_{8,9}$ = 7.5 Hz, $^3J_{6,8}$ = 4.6 Hz, $^3J_{8,OH}$ = 3.0 Hz, H-8), 3.61 (t, 2H, $^3J_{1,2}$ = 6.2 Hz, H-1), 3.40 (dt, 1H, $^3J_{5,6}$ = 9.4 Hz, $^3J_{4,5}$ = 2.8 Hz, H-5), 2.43 – 2.36 (m, 1H, H-9), 2.20 (d, 1H, $^3J_{8,OH}$ = 3.0 Hz, H-OH), 1.78 (dqd, 1H, $^3J_{5,6}$ = 9.4 Hz, $^3J_{6,7}$ = 7.0 Hz, $^3J_{6,8}$ = 4.6 Hz, H-6), 1.54 – 1.39 (m, 5H, H-2, H-3a, H-4), 1.35 – 1.28 (m, 1H, H-3b), 0.96 (d, 3H, $^3J_{9,10}$ = 6.8 Hz, H-10), 0.90 (s, 9H, TBS), 0.89 (s, 9H, TBS), 0.82 (d, 3H, $^3J_{6,7}$ = 7.0 Hz, H-7), 0.08 (s, 6H, TBS), 0.04 (s, 6H, TBS); **^{13}C-NMR** (176 MHz, CD_2Cl_2, 298 K): *δ* [ppm] = 143.3 (C-11), 114.5 (C-12), 76.4 (C-8), 75.1 (C-5), 63.6 (C-1),

41.4 (C-6), 39.7 (C-9), 33.6 (C-4), 32.7 (C-2), 26.1 (3C, TBS), 26.1 (3C, TBS), 22.5 (C-3), 18.6 (TBS), 18.4 (TBS), 11.6 (C-10), 10.8 (C-7), -4.3 (TBS), -4.4 (TBS), -5.2 (2C, TBS); **HRMS (ESI+)** *m/z*: $[M + H]^+$ calcd. for $C_{24}H_{52}O_3Si_2H^+$ 445.3528, found 445.3518.

Synthesis of compound S-27

(5*R*,6*S*,7*S*)-5-((*S*)-But-3-en-2-yl)-7-((*tert*-butyldimethylsilyl)oxy)-2,2,3,3,6,13,13,14,14-nonamethyl-4,12-dioxa-3,13-disilapentadecane

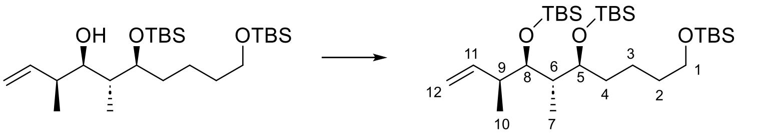

S-26 **S-27**

$C_{30}H_{66}O_3Si_3$
559.11 g/mol

DIPEA (825 µL, 4.86 mmol, 2.0 eq.) and TBSOTf (840 µL, 3.64 mmol, 1.5 eq.) were added to a solution of alcohol **S-26** (1.08 g, 2.43 mmol, 1.0 eq.) in dry DCM (10 mL) at 0°C. After stirring for 50 min at 0 °C, NH_4Cl-solution (aq., sat., 10 mL) was added and the phases were separated. The aqueous layer was extracted with DCM (3 x 5 mL), the combined organic layers were dried over $MgSO_4$ and concentrated *in vacuo*. Purification by flash column chromatography (SiO_2, cyclohexane/ethyl acetate 10:1) yielded silyl ether **S-27** (1.33 g, 2.38 mmol, 98%) as a colorless oil.

R_f: 0.75 (cyclohexane/ethyl acetate 10:1); **$[\alpha]_D^{20}$** = -13.0° (c = 1.00, CH_2Cl_2); **^{1}H-NMR** (700 MHz, CD_2Cl_2, 298 K): δ [ppm] = 5.91 (ddd, 1H, $^3J_{11,12E}$ = 17.4 Hz, $^3J_{11,12Z}$ = 10.4 Hz, $^3J_{9,11}$ = 7.0 Hz, H-11), 5.04 – 4.96 (m, 2H, H-12), 3.92 (dd, 1H, $^3J_{8,9}$ = 5.5 Hz, $^3J_{6,8}$ = 5.0 Hz, H-8), 3.64 – 3.55 (m, 3H, H-1, H-5), 2.40 (dqdt, 1H, $^3J_{9,11}$ = 7.0 Hz, $^3J_{9,10}$ = 6.9 Hz, $^3J_{8,9}$ = 5.5 Hz, $^4J_{9,12}$ = 1.4 Hz, H-9), 1.84 (qdd, 1H, $^3J_{6,7}$ = 7.1 Hz, $^3J_{5,6}$ = 7.1 Hz, $^3J_{6,8}$ = 5.0 Hz, H-6), 1.54 – 1.40 (m, 3H, H-2a, H-4), 1.40 – 1.32 (m, 2H, H-2b, H-3a), 1.32 – 1.20 (m, 1H, H-3b), 0.99 (d, 3H, $^3J_{9,10}$ = 6.9 Hz, H-10), 0.92 – 0.86 (m, 27H, TBS), 0.83 (d, 3H, $^3J_{6,7}$ = 7.1 Hz, H-7), 0.08 – 0.01 (m, 18H, TBS); **^{13}C-NMR** (176 MHz, CD_2Cl_2, 298 K): δ [ppm] = 143.5 (C-11), 113.7 (C-12), 77.6 (C-8), 72.8 (C-5), 63.6 (C-1), 44.1 (C-6), 41.7 (C-9), 33.9 (C-4), 32.0 (C-2), 26.4 (3C, TBS), 26.2 (3C, TBS), 26.1 (3C, TBS), 22.4 (C-3), 18.7 (TBS), 18.6 (TBS), 18.4 (TBS), 14.4 (C-10),

10.5 (C-7), -3.4 (TBS), -3.7 (TBS), -4.1 (TBS), -4.1 (TBS), -5.2 (TBS), -5.2 (TBS); **HRMS (APCI)** *m/z*: $[M + H]^+$ calcd. for $C_{30}H_{66}O_3Si_3H^+$ 559.4393, found 559.4389.

Synthesis of compound S-28

4-((4*S*,5*S*,6*R*)-6-((*S*)-But-3-en-2-yl)-2,2,5-trimethyl-1,3-dioxan-4-yl)butan-1-ol

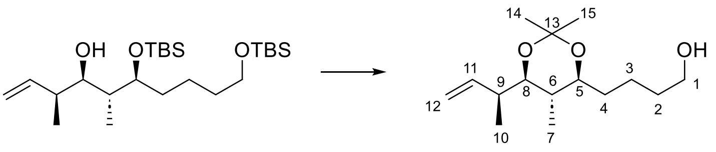

S-26 **S-28**
$C_{15}H_{28}O_3$
256.39 g/mol

A solution of homoallyl alcohol **S-26** (10.0 mg, 22.5 µmol, 1.0 eq.) in MeOH/MeCN (1:1, 1.0 mL) was treated with HCl (1.0 M in H_2O, 110 µL, 110 µmol, 5.0 eq.) and stirred at ambient temperature for 100 min. Afterwards, the mixture was diluted with brine (0.5 mL), water (0.5 mL) and acetonitrile (1.0 mL), followed by extraction of the aqueous phase with ethyl acetate (5 x 1.0 mL), drying over Na_2SO_4 and concentration *in vacuo*. The resulting crude product was dissolved in dry DCM (0.5 mL) and 2,2-dimethoxypropane (0.05 mL) and a catalytic amount of CSA (<1.0 mg) was added. After stirring overnight, acetic acid (2.0 M in H_2O, 2 mL) and methanol (1 mL) were added and stirring continued for additional 5 h. Extraction of the aqueous layer with DCM (3 x 1 mL), drying of the combined organic phases over $MgSO_4$ and concentration *in vacuo*, followed by flash column chromatographic purification (SiO_2, cyclohexane/ethyl acetate 4:1), yielded acetonide **S-28** (4.00 mg, 15.8 µmol, 70%) as a colorless oil.

R_f: 0.38 (cyclohexane/ethyl acetate 1:1); **$[\alpha]_D^{20}$** = -16.7° (c = 0.78, CH_2Cl_2); **^{1}H-NMR** (700 MHz, CD_2Cl_2, 298 K): δ [ppm] = 5.91 (ddd, 1H, $^3J_{11,12E}$ = 17.4 Hz, $^3J_{11,12Z}$ = 10.4 Hz, $^3J_{9,11}$ = 7.1 Hz, H-11), 5.01 (ddd, 1H, $^3J_{11,12E}$ = 17.4 Hz, $^2J_{12Z,12E}$ = 1.6 Hz, $^4J_{9,12E}$ = 1.6 Hz, H-12E), 4.94 (ddd, 1H, $^3J_{11,12Z}$ = 10.4 Hz, $^2J_{12Z,12E}$ = 1.6 Hz, $^4J_{9,12Z}$ = 1.1 Hz, H-12Z), 3.60 (t, 2H, $^3J_{1,2}$ = 6.3 Hz, H-1), 3.49 (dd, 1H, $^3J_{6,8}$ = 10.2 Hz, $^3J_{8,9}$ = 2.5 Hz, H-8), 3.44 (ddd, 1H, $^3J_{5,6}$ = 10.2 Hz, $^3J_{4a,5}$ = 6.2 Hz, $^3J_{4b,5}$ = 4.4 Hz, H-5), 2.40 (dqdt, 1H, $^3J_{9,11}$ = 7.0 Hz, $^3J_{9,10}$ = 6.9 Hz, $^3J_{8,9}$ = 2.5 Hz, $^4J_{9,12}$ = 1.1 Hz, H-9), 1.68 – 1.61 (m, 1H, H-6), 1.59 – 1.47 (m, 2H, H-4), 1.44 – 1.32 (m, 7H, H-2, H-3, H-14), 1.30

(s, 3H, H-15), 0.96 (d, 3H, $^3J_{9,10}$ = 6.9 Hz, H-10), 0.77 (d, 3H, $^3J_{6,7}$ = 6.6 Hz, H-7); **^{13}C-NMR** (176 MHz, CD_2Cl_2, 298 K): δ [ppm] = 143.7 (C-11), 113.1 (C-12), 98.1 (C-13), 77.5 (C-8), 74.5 (C-5), 63.1 (C-1), 38.6 (C-6), 35.8 (C-9), 33.2 (C-4), 33.2 (C-2), 30.2 (C-14), 21.8 (C-3), 19.7 (C-15), 12.4 (C-10), 12.1 (C-7); **HRMS (ESI+)** *m/z*: $[M + H]^+$ calcd. for $C_{15}H_{28}O_3H^+$ 257.2111, found 257.2107.

The relative configuration of the 1,3-diol and the methyl group were confirmed by NMR-analysis of the coupling constants of acetonide **S-28** to be 1,2-*anti*/1,3-*anti*.

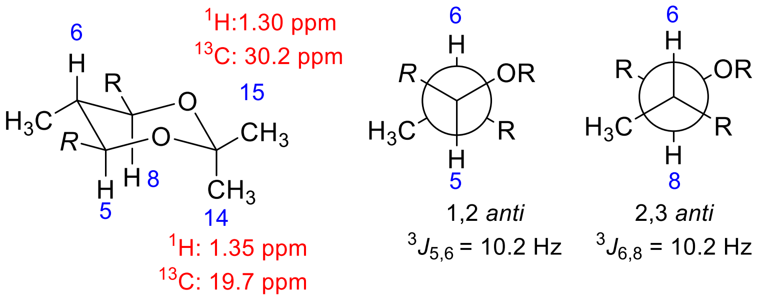

Figure 4: Analysis of the coupling constants of acetonide **S-28** confirmed the *1,2*-anti/*1,3*-anti-configuration.

Synthesis of compound S-29

(*S*)-2,2,3,3,11,11,12,12-Octamethyl-5-((*R*)-1-((4*R*,5*S*)-2,2,5-trimethyl-1,3-dioxan-4-yl)ethyl)-4,10-dioxa-3,11-disilatridecane

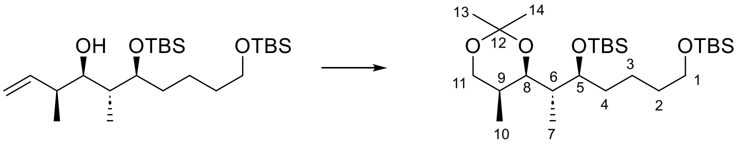

S-26

S-29
$C_{26}H_{56}O_4Si_2$
488.90 g/mol

A solution of homoallyl alcohol **S-26** (14.4 mg, 32.4 µmol, 1.0 eq.) in DCM/MeOH (15:1, 1.6 mL) was cooled to –78 °C and ozone was bubbled through (ca. 2 min) under vigorous stirring. The resulting blue solution was flushed with argon until the blue color mainly vanished and subsequently, $NaBH_4$ (6.10 mg, 161 µmol, 5.0 eq.) was added. After 10 min stirring at –78 °C, the reaction mixture was allowed to warm to 0 °C and to stir for additional 3 h. Then, NH_4Cl-solution (aq., sat., 2 mL) was added and the phases were separated. Extraction of the aqueous layer with DCM (3 x 1 mL), drying of the combined organic layers over Na_2SO_4 and concentration *in vacuo* gave the crude product, which was purified by flash column chromatography (SiO_2, cyclohexane/ethyl acetate 4:1) to yield a colorless oil. This product was dissolved in dry DCM (0.5 mL) and 2,2-dimethoxypropane (0.5 mL) and a catalytic amount of CSA (<1.0 mg) was added. After stirring overnight, water (1.0 mL) was added and the phases were separated. Extraction of the aqueous layer with DCM (3 x 1 mL), drying of the combined organic phase over $MgSO_4$ and concentration *in vacuo* followed by column chromatographic purification (SiO_2, cyclohexane/ethyl acetate 10:1) yielded acetonide **S-29** (10.2 mg, 22.5 µmol, 70%) as a colorless oil.

R_f: 0.50 (cyclohexane/ethyl acetate 10:1); **$[\alpha]_D^{20}$** = -23.4° (c = 0.94, CH_2Cl_2); **^{1}H-NMR** (700 MHz, CD_2Cl_2, 298 K): δ [ppm] = 4.05 (dd, 1H, $^2J_{11a,11b}$ = 11.5 Hz, $^3J_{9,11a}$ = 2.8 Hz, H-11a), 3.96 (ddd, 1H, $^3J_{4a,5}$ = 8.9 Hz, $^3J_{4b,5}$ = 3.5 Hz, $^3J_{5,6}$ = 3.5 Hz, H-5), 3.64 (dd, 1H, $^3J_{6,8}$ = 10.4 Hz, $^3J_{8,9}$ = 2.3 Hz, H-8), 3.60 (t, 2H, $^3J_{1,2}$ = 6.3 Hz, H-1), 3.50 (dd, 1H, $^2J_{11a,11b}$ = 11.5 Hz, $^3J_{9,11b}$ = 1.7 Hz, H-11b), 1.77 (dqd, 1H, $^3J_{6,8}$ = 10.4 Hz, $^3J_{6,7}$ = 7.0 Hz, $^3J_{5,6}$ = 3.5 Hz, H-6), 1.54 – 1.48 (m, 2H, H-4a, H-9), 1.47 – 1.41 (m, 2H, H-2a, H-4b), 1.37 – 1.21 (m, 9H, H-2b, H-3, H-13, H-14), 1.02 (d, 3H, $^3J_{9,10}$ = 6.9 Hz, H-10), 0.91 – 0.85 (m, 18H, TBS), 0.74 (d, 3H, $^3J_{6,7}$ = 7.0 Hz, H-7), 0.05 – 0.03 (m, 12H, TBS);

¹³C-NMR (176 MHz, CD_2Cl_2, 298 K): *δ* [ppm] = 98.5 (C-12), 73.2 (C-8), 71.8 (C-5), 67.6 (C-11), 63.7 (C-1), 40.7 (C-6), 33.5 (C-9), 30.9 (C-4), 30.2 (C-14), 29.9 (C-2), 26.1 (3C, TBS), 26.1 (3C, TBS), 23.4 (C-3), 19.0 (C-13), 18.6 (TBS), 18.4 (TBS), 10.4 (C-10), 8.4 (C-7), -4.3 (TBS), -4.4 (TBS), -5.2 (TBS), -5.2 (TBS); **HRMS (ESI+)** *m/z*: $[M + H]^+$ calcd. for $C_{26}H_{56}O_4Si_2H^+$ 489.3790, found 489.3788.

The relative configuration of the 1,3-diol and the methyl group were confirmed by NMR-analysis of the coupling constants of acetonide **S-29** to be *syn*.

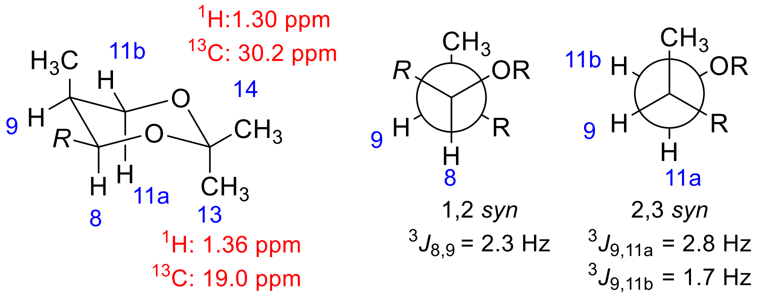

Figure 5: Analysis of the coupling constants of acetonide **S-29** confirmed the *syn* -configuration.

Synthesis of compound 34

(2*R*,3*S*,4*S*,5*S*)-3,5,9-Tris((*tert*-butyldimethylsilyl)oxy)-2,4-dimethylnonanal

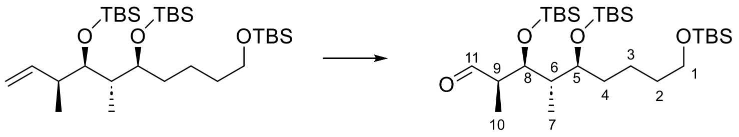

S-27

34
$C_{29}H_{64}O_4Si_3$
561.08 g/mol

A solution of silyl ether **S-27** (70.0 mg, 125 µmol, 1.0 eq.) in DCM/MeOH (10:1, 2.5 mL) was cooled to –78 °C and ozone was bubbled through (ca. 5 min) under vigorous stirring. The resulting blue solution was flushed with argon until the blue color mainly vanished and subsequently, PPh_3 (42.7 mg, 163 µmol, 1.3 eq.) was added. After 15 min stirring at –78 °C, the reaction mixture was warmed to room temperature to stir for further 90 min. After concentration *in vacuo* (35 °C evaporation temperature, <1 h to avoid elimination of sec. siloxy-group), flash column chromatographic purification

(SiO_2, toluene) of the residue yielded aldehyde **34** (62.0 mg, 110 µmol, 88%) as a colorless oil which was used within 24 h.

Rf: 0.36 (toluene); **[α]D20** = -15.0° (c = 1.00, CH_2Cl_2); **1H-NMR** (700 MHz, CD_2Cl_2, 298 K): δ [ppm] = 9.69 (s, 1H, H-11), 4.26 (dd, 1H, $^3J_{6,8}$ = 7.9 Hz, $^3J_{8,9}$ = 2.1 Hz, H-8), 3.86 (ddd, 1H, $^3J_{4a,5}$ = 7.8 Hz, $^3J_{4b,5}$ = 4.5 Hz, $^3J_{5,6}$ = 4.5 Hz, H-5), 3.62 (t, 2H, $^3J_{1,2}$ = 6.0 Hz, H-1), 2.52 (qd, 1H, $^3J_{9,10}$ = 7.0 Hz, $^3J_{8,9}$ = 2.1 Hz, H-9), 1.91 (dqd, 1H, $^3J_{6,8}$ = 7.9 Hz, $^3J_{6,7}$ = 7.3 Hz, $^3J_{5,6}$ = 4.5 Hz, H-6), 1.54 – 1.47 (m, 3H, H-2a, H-4), 1.44 – 1.38 (m, 2H, H-2b, H-3a), 1.34 – 1.28 (m, 1H, H-3b), 1.10 (d, 3H, $^3J_{9,10}$ = 7.0 Hz, H-10), 0.90 – 0.88 (m, 18H, TBS), 0.87 (s, 9H, TBS), 0.85 (d, 3H, $^3J_{6,7}$ = 7.3 Hz, H-7), 0.07 – -0.03 (m, 18H, TBS); **13C-NMR** (176 MHz, CD_2Cl_2, 298 K): δ [ppm] = 205.4 (C-11), 72.9 (C-8), 72.0 (C-5), 63.5 (C-1), 50.5 (C-9), 43.9 (C-6), 33.8 (C-4), 32.0 (C-2), 26.2 (3C, TBS), 26.1 (3C, TBS), 26.1 (3C, TBS), 22.5 (C-3), 18.6 (TBS), 18.5 (TBS), 18.3 (TBS), 10.8 (C-10), 7.4 (C-7), -3.9 (TBS), -4.0 (TBS), -4.1 (TBS), -4.2 (TBS), -5.2 (TBS), -5.2 (TBS); **HRMS (ESI+)** *m/z*: $[M + H]^+$ calcd. for $C_{29}H_{64}O_4Si_3Na^+$ 583.4005, found 583.4010.

Synthesis of compound S-30

(5*S*,10*S*,11*R*,12*S*,13*S*,*E*)-11,13-Bis((*tert*-butyldimethylsilyl)oxy)-3,3-diethyl-5-((*E*)-4-((4-methoxybenzyl)oxy)-3-methylbut-2-en-1-yl)-10,12,19,19,20,20-hexamethyl-4,18-dioxa-3,19-disilahenicos-8-en-7-one

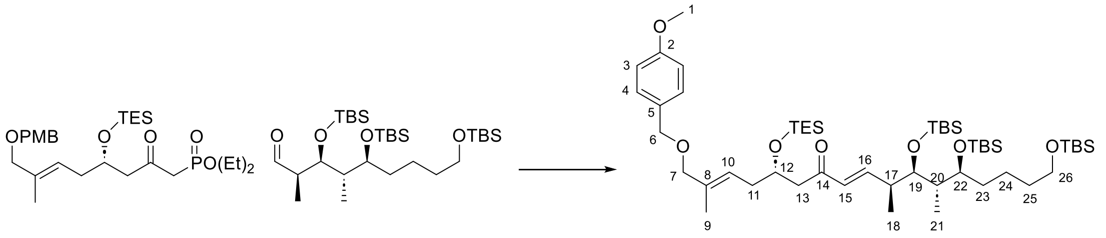

33 **34** **S-30**

$C_{52}H_{100}O_7Si_4$

949.71 g/mol

$Ba(OH)_2$ (91.2 mg, 532 µmol, 1.0 eq.) was added to a solution of phosphonate **34** (303 mg, 559 µmol, 1.06 eq) in dry THF (6.6 mL). After stirring for 1 h at ambient temperature, the resulting suspension was cooled to 0 °C and aldehyde **33** (296 mg, 528 µmol, 1.0 eq), dissolved in THF/water (40:1, 5.15 mL), was added slowly. Stirring continued for 5.5 h at 0 °C and naturally warmed to room temperature while stirring for 15 h. NH_4Cl-solution (aq., sat., 8 mL) and water (8 mL) were added, the phases were

separated, the aqueous layer was extracted with Et_2O (3 x 15 mL) and the organic extracts were dried over Na_2SO_4. Concentration *in vacuo* and purification by flash column chromatography (SiO_2, cyclohexane/ethyl acetate 25:1) yielded enone **S-30** (347 mg, 364 µmol, 69%) as a pale-yellow oil.

R*f*: 0.42 (cyclohexane/ethyl acetate 10:1); **$[\alpha]_D^{20}$** = 0.0° (c = 1.00, CH_2Cl_2); **^{1}H-NMR** (500 MHz, CD_2Cl_2, 298 K): δ [ppm] = 7.24 (d, 2H, $^3J_{3,4}$ = 8.7 Hz, H-4), 6.90 – 6.81 (m, 3H, H-3, H-16), 6.08 (dd, 1H, $^3J_{15,16}$ = 15.9 Hz, $^4J_{15,17}$ = 1.4 Hz, H-15), 5.50 – 5.44 (m, 1H, H-10), 4.34 (s, 2H, H-6), 4.29 (dtd, 1H, $^3J_{12,13a}$ = 6.9 Hz, $^3J_{11,12}$ = 6.5 Hz, $^3J_{12,13b}$ = 5.2 Hz, H-12), 3.89 – 3.82 (m, 3H, H-7, H-22), 3.78 (s, 3H, H-1), 3.69 – 3.66 (m, 1H, H-19), 3.60 (t, 2H, $^3J_{25,26}$ = 6.0 Hz, H-26), 2.72 (dd, 1H, $^2J_{13a,13b}$ = 15.7 Hz, $^3J_{12,13a}$ = 6.9 Hz, H-13a), 2.60 – 2.52 (m, 2H, H-11a, H-17), 2.27 (dd, 2H, $^3J_{10,11}$ = 7.0 Hz, $^3J_{11,12}$ = 6.5 Hz, H-11), 1.85 (dqd, 1H, $^3J_{19,20}$ = 7.3 Hz, $^3J_{20,21}$ = 7.1 Hz, $^3J_{20,22}$ = 4.9 Hz, H-20), 1.65 (d, 3H, $^4J_{9,10}$ = 1.4 Hz, H-9), 1.52 – 1.31 (m, 5H, H-23a, H-24, H-25), 1.30 – 1.21 (m, 1H, H-23b), 1.05 (d, 3H, $^3J_{17,18}$ = 6.8 Hz, H-18), 0.95 – 0.87 (m, 36H, 3xTBS, TES), 0.83 (d, 3H, $^3J_{20,21}$ = 7.1 Hz, H-21), 0.57 (q, 6H, $^3J_{TES,TES}$ = 7.8 Hz, TES), 0.07 – 0.00 (m, 18H, TBS); **^{13}C-NMR** (126 MHz, $CDCl_3$, 298 K): δ [ppm] = 198.8 (C-14), 159.5 (C-2), 151.5 (C-16), 135.1 (C-8), 131.3 (C-5), 130.4 (C-15), 129.6 (2C, C-4), 123.7 (C-10), 114.0 (2C, C-3), 76.7 (C-19), 76.3 (C-7), 72.8 (C-22), 71.5 (C-6), 69.2 (C-12), 63.5 (C-26), 55.6 (C-1), 48.1 (C-13), 44.2 (C-20), 40.7 (C-17), 36.5 (C-11), 33.9 (C-25), 31.9 (C-23), 26.3 (3C, TBS), 26.1 (6C, TBS), 22.4 (C-24), 18.6 (TBS), 18.6 (TBS), 18.4 (TBS), 14.3 (C-9), 13.5 (C-18), 10.6 (C-21), 7.1 (3C, TES), 5.3 (3C, TES), -3.6 (TBS), -3.7 (TBS), -4.1 (TBS), -4.1 (TBS), -5.2 (TBS), -5.2 (TBS); **HRMS (ESI+)** *m*/*z*: $[M+NH_4]^+$ calcd. for $C_{52}H_{100}O_7Si_4NH_4^+$ 966.6884, found 966.6885.

Synthesis of compound 35

(5*S*,10*S*,11*R*,12*S*,13*S*)-11,13-Bis((*tert*-butyldimethylsilyl)oxy)-3,3-diethyl-5-((*E*)-4-((4-methoxybenzyl)oxy)-3-methylbut-2-en-1-yl)-10,12,19,19,20,20-hexamethyl-4,18-dioxa-3,19-disilahenicosan-7-one

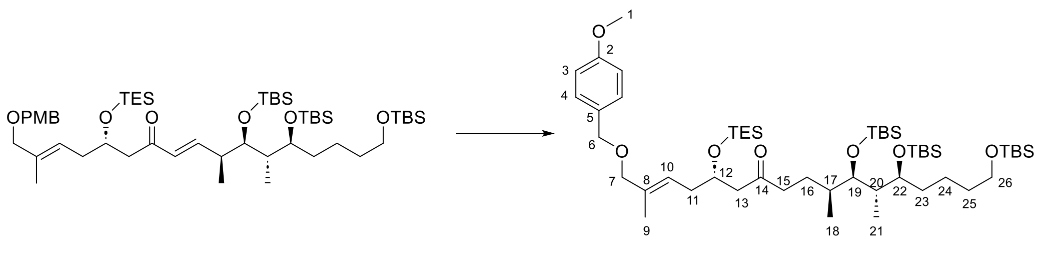

S-30 **35**

$C_{52}H_{102}O_7Si_4$
951.72 g/mol

The 'hot Stryker' stock solution was prepared according to the literature procedure.[4] A solution of enone **S-30** (1.11 g, 1.17 mmol, 1.0 eq.) and *tert*-butyl alcohol (335 µL, 3.51 mmol, 3.0 eq.) in dry toluene (22.0 mL) was degassed by freeze-pump-thaw technique before 'hot Stryker' stock solution (10.0 mM (BDP)CuH, 1.75 mL, 17.5 µmol, 0.01 eq.) was added. After stirring at 45 °C for 24 h, the resulting solution was concentrated *in vacuo* and purified via flash column chromatography (SiO_2, cyclohexane/ethyl acetate 30:1 → 20:1) to obtain ketone **35** (1.04 g, 1.10 mmol, 94%) as a colorless oil.

R*f*: 0.20 (cyclohexane/ethyl acetate 20:1); **$[\alpha]_D^{20}$** = -2.2° (c = 0.93, CH_2Cl_2); **^{1}H-NMR** (500 MHz, CD_2Cl_2, 298 K): δ [ppm] = 7.24 (d, 2H, $^3J_{3,4}$ = 8.7 Hz, H-4), 6.86 (d, 2H, $^3J_{3,4}$ = 8.7 Hz, H-3), 5.45 (tq, 1H, $^3J_{10,11}$ = 7.4 Hz, $^4J_{9,10}$ = 1.4 Hz, H-10), 4.35 (s, 2H, H-6), 4.23 (dtd, 1H, $^3J_{12,13a}$ = 7.2 Hz, $^3J_{11,12}$ = 6.2 Hz, $^3J_{12,13b}$ = 5.1 Hz, H-12), 3.92 – 3.84 (m, 3H, H-7, H-22), 3.79 (s, 3H, H-1), 3.63 – 3.57 (m, 2H, H-26), 3.48 (dd, 1H, $^3J_{19,20}$ = 8.5 Hz, $^3J_{17,19}$ = 1.6 Hz, H-19), 2.55 (dd, 1H, $^2J_{13a,13b}$ = 15.6 Hz, $^3J_{12,13a}$ = 7.1 Hz, H-13a), 2.50 – 2.40 (m, 2H, H-13b, H-15a), 2.38 – 2.30 (m, 1H, H-15b), 2.24 (dd, 2H, $^3J_{10,11}$ = 7.4 Hz, $^3J_{11,12}$ = 6.2 Hz, H-11), 1.88 – 1.79 (m, 1H, H-20), 1.64 (d, 3H, $^4J_{9,10}$ = 1.4 Hz, H-9), 1.62 – 1.55 (m, 2H, H-16a, H-17), 1.51 – 1.44 (m, 4H, H-23a, H-24a, H-25), 1.39 – 1.20 (m, 3H, H-16b, H-23b, H-24b), 0.95 – 0.87 (m, 36H, 3xTBS, TES), 0.83 (d, 3H, $^3J_{17,18}$ = 6.5 Hz, H-18), 0.78 (d, 3H, $^3J_{20,21}$ = 7.1 Hz, H-21), 0.58 (q, 6H, $^3J_{TES,TES}$ = 8.0 Hz, TES), 0.08 – 0.02 (m, 18H, TBS); **^{13}C-NMR** (126 MHz, $CDCl_3$, 298 K): δ [ppm] = 209.5 (C-14), 159.5 (C-2), 135.1 (C-8), 131.3 (C-5), 129.6 (2C, C-4), 123.5 (C-10), 114.0 (2C, C-3), 77.8 (C-19), 76.2 (C-7), 72.9

(C-22), 71.6 (C-6), 69.1 (C-12), 63.6 (C-26), 55.6 (C-1), 50.2 (C-13), 44.1 (C-20), 43.2 (C-15), 36.4 (C-17), 36.4 (C-11), 34.0 (C-25), 31.4 (C-23), 28.9 (C-16), 26.5 (3C, TBS), 26.1 (6C, TBS), 22.9 (C-24), 18.8 (TBS), 18.6 (TBS), 18.4 (TBS), 14.3 (C-9), 13.4 (C-18), 10.6 (C-21), 7.1 (3C, TES), 5.3 (3C, TES), -3.4 (TBS), -3.4 (TBS), -4.1 (2C, TBS), -5.2 (TBS), -5.2 (TBS); **HRMS (ESI+)** *m/z*: $[M+Na]^+$ calcd. for $C_{52}H_{102}O_7Si_4Na^+$ 973.6595, found 973.6599.

Synthesis of compound S-31

(5*S*,10*S*,11*R*,12*S*,13*S*,*E*)-11,13-Bis((*tert*-butyldimethylsilyl)oxy)-5,17-dihydroxy-1-((4-methoxybenzyl)oxy)-2,10,12-trimethylheptadec-2-en-7-one

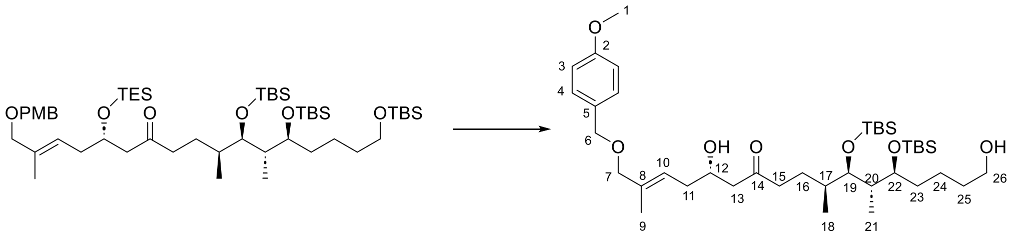

35

S-31

$C_{40}H_{74}O_7Si_2$

723.20 g/mol

A solution of ketone **35** (161 mg, 170 µmol, 1.0 eq.) in dry MeOH (12.0 mL) and dry DCM (0.2 mL) was cooled to 0 °C before $PdCl_2$ (7.50 mg, 42.6 µmol, 0.25 eq.) was added. The resulting mixture was stirred for 4.25 h at 0 °C and was then diluted with DCM (50 mL). Filtration of the mixture over a Celite® pad and concentration *in vacuo* yielded a cloudy resin that was dissolved in Et_2O (10 mL) and filtrated over a silica pad. After concentration *in vacuo* and flash column chromatographic purification (SiO_2, cyclohexane/ethyl acetate 3:1) ketone **S-31** was received as a colorless resin (100 mg, 139 µmol, 82%).

R*f*: 0.15 (cyclohexane/ethyl acetate 7:3); **$[\alpha]_D^{20}$** = 0.0° (c = 0.27, CH_2Cl_2); **^{1}H-NMR** (700 MHz, CD_2Cl_2, 298 K): δ [ppm] = 7.24 (d, 2H, $^3J_{3,4}$ = 8.7 Hz, H-4), 6.86 (d, 2H, $^3J_{3,4}$ = 8.7 Hz, H-3), 5.46 (ddq, 1H, $^3J_{10,11a}$ = 7.3 Hz, $^3J_{10,11b}$ = 6.0 Hz, $^4J_{9,10}$ = 1.4 Hz, H-10), 4.36 (s, 2H, H-6), 4.08 – 4.03 (m, 1H, H-12), 3.91 – 3.86 (m, 3H, H-7, H-22), 3.79 (s, 3H, H-1), 3.59 (t, 2H, $^3J_{25,26}$ = 6.5 Hz, H-26), 3.49 (dd, 1H, $^3J_{19,20}$ = 8.3 Hz, $^3J_{17,19}$ = 1.9 Hz, H-19), 2.62 – 2.56 (m, 1H, H-13a), 2.52 – 2.45 (m, 2H, H-13b, H-15a), 2.38 (ddd, 1H, $^2J_{15a,15b}$ = 17.0 Hz, $^3J_{15b,16}$ = 9.7 Hz, $^3J_{15b,16}$ = 5.7 Hz, H-15b), 2.28 –

2.23 (m, 1H, H-11a), 2.23 – 2.17 (m, 1H, H-11b), 1.84 (dqd, 1H, $^3J_{19,20}$ = 8.3 Hz, $^3J_{20,21}$ = 7.1 Hz, $^3J_{20,22}$ = 4.4 Hz, H-20), 1.67 (d, 3H, $^4J_{9,10}$ = 1.4 Hz, H-9), 1.66 – 1.57 (m, 2H, H-16a, H-17), 1.56 – 1.44 (m, 4H, H-16a, H-23a, H-24a, H-25), 1.38 – 1.32 (m, 2H, H-16b, H-23b), 1.30 – 1.23 (m, 1H, H-24b), 0.91 (s, 9H, TBS), 0.89 (s, 9H, TBS), 0.84 (d, 3H, $^3J_{17,18}$ = 6.8 Hz, H-18), 0.80 (d, 3H, $^3J_{20,21}$ = 7.1 Hz, H-21), 0.08 – 0.03 (m, 12H, TBS); **^{13}C-NMR** (176 MHz, $CDCl_3$, 298 K): δ [ppm] = 212.2 (C-14), 159.6 (C-2), 135.6 (C-8), 131.2 (C-5), 129.7 (2C, C-4), 123.3 (C-10), 114.0 (2C, C-3), 77.7 (C-19), 76.1 (C-7), 72.9 (C-22), 71.8 (C-6), 68.0 (C-12), 63.2 (C-26), 55.6 (C-1), 48.8 (C-13), 44.0 (C-20), 42.5 (C-15), 36.5 (C-17), 35.2 (C-11), 33.8 (C-25), 31.4 (C-23), 28.9 (C-16), 26.4 (3C, TBS), 26.1 (3C, TBS), 22.6 (C-24), 18.8 (TBS), 18.4 (TBS), 14.3 (C-9), 13.5 (C-18), 10.7 (C-21), -3.4 (TBS), -3.5 (TBS), -4.1 (TBS), -4.2 (TBS); **HRMS (ESI+)** *m*/*z*: $[M+H]^+$ calcd. for $C_{40}H_{74}O_7Si_2H^+$ 723.5046, found 723.5049.

Synthesis of compound S-32

(5*S*,6*S*,7*R*,8*S*,11*R*,13*S*,*E*)-5,7-Bis((*tert*-butyldimethylsilyl)oxy)-17-((4-methoxybenzyl)oxy)-6,8,16-trimethylheptadec-15-ene-1,11,13-triol

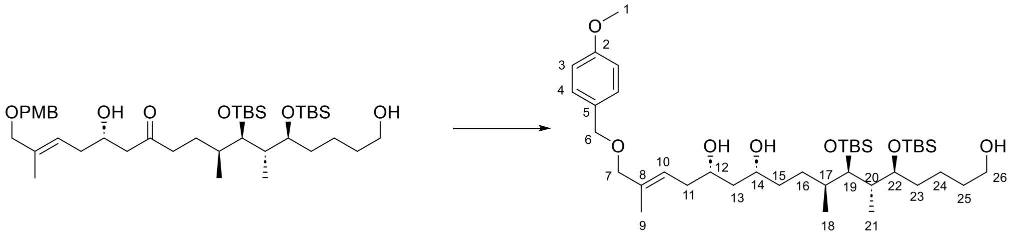

S-31

S-32
$C_{40}H_{76}O_7Si_2$
725.21 g/mol

A solution of ketone **S-31** (615 mg, 850 µmol, 1.0 eq.) in dry THF/MeOH (4:1, 10.0 mL) was cooled to –78 °C before diethyl methoxyborane (1.0 M in THF, 1.74 mL, 1.74 mmol, 2.05 eq.) was added dropwise. After 15 min of stirring, $NaBH_4$ (48.3 mg, 1.28 mmol, 1.5 eq.) was carefully added and stirring continued for 4 h at –78 °C. The resulting mixture was quenched by dropwise addition of HOAc (1.7 mL), diluted with ethyl acetate (25 mL) and stirred for 10 min at room temperature. $NaHCO_3$-solution (aq., sat., 25 mL) was added carefully, resulting in CO_2 evolution and the aqueous layer was extracted with ethyl acetate (2 x 20 mL). Drying of the combined organic layers over Na_2SO_4 and concentration *in vacuo* gave a crude product, which was azeotroped

several times with methanol (6 x 5.0 mL). Flash column chromatographic purification (SiO_2, cyclohexane/ethyl acetate 3:2) of the resulting residue yielded triol **S-32** (595 mg, 808 µmol, 96%) as a colorless resin.

R*f*: 0.29 (cyclohexane/ethyl acetate 1:1); **$[\alpha]_D^{20}$** = -11.6° (c = 0.78, CH_2Cl_2); **^{1}H-NMR** (700 MHz, CD_2Cl_2, 298 K): δ [ppm] = 7.24 (d, 2H, $^3J_{3,4}$ = 8.7 Hz, H-4), 6.86 (d, 2H, $^3J_{3,4}$ = 8.7 Hz, H-3), 5.46 (tq, 1H, $^3J_{10,11}$ = 7.4 Hz, $^4J_{9,10}$ = 1.4 Hz, H-10), 4.37 (s, 2H, H-6), 3.92 – 3.86 (m, 4H, H-7, H-12, H-22), 3.81 – 3.78 (m, 1H, H-14), 3.79 (s, 3H, H-1), 3.59 (t, 2H, $^3J_{25,26}$ = 6.4 Hz, H-26), 3.50 (dd, 1H, $^3J_{19,20}$ = 8.2 Hz, $^3J_{17,19}$ = 2.0 Hz, H-19), 2.85 (d, 1H, $^3J_{12,OH}$ = 2.8 Hz, H-OH), 2.82 (d, 1H, $^3J_{14,OH}$ = 2.6 Hz, H-OH), 2.29 – 2.18 (m, 2H, H-11), 1.84 (dqd, 1H, $^3J_{19,20}$ = 8.2 Hz, $^3J_{20,21}$ = 7.1 Hz, $^3J_{20,22}$ = 4.3 Hz, H-20), 1.67 (d, 3H, $^4J_{9,10}$ = 1.4 Hz, H-9), 1.65 – 1.59 (m, 2H, H-13a, H-17), 1.56 – 1.43 (m, 6H, H-13b, H-15a, H-16a, H-24a, H-25), 1.42 – 1.29 (m, 4H, H-15b, H-16b, H-23), 1.29 – 1.17 (m, 2H, H-24b, H-OH), 0.91 (s, 9H, TBS), 0.89 (s, 9H, TBS), 0.86 (d, 3H, $^3J_{17,18}$ = 6.9 Hz, H-18), 0.82 (d, 3H, $^3J_{20,21}$ = 7.1 Hz, H-21), 0.07 – 0.04 (m, 12H, TBS); **^{13}C-NMR** (176 MHz, $CDCl_3$, 298 K): δ [ppm] = 159.6 (C-2), 136.0 (C-8), 131.2 (C-5), 129.7 (2C, C-4), 123.3 (C-10), 114.0 (2C, C-3), 77.9 (C-19), 76.2 (C-7), 73.7 (C-14), 73.1 (C-22), 73.0 (C-12), 71.8 (C-6), 63.1 (C-26), 55.6 (C-1), 44.0 (C-20), 43.0 (C-13), 37.2 (C-17), 37.2 (C-15), 36.9 (C-11), 33.7 (C-25), 31.3 (C-23), 30.8 (C-16), 26.5 (3C, TBS), 26.1 (3C, TBS), 22.6 (C-24), 18.8 (TBS), 18.4 (TBS), 14.4 (C-9), 13.8 (C-18), 10.7 (C-21), -3.4 (TBS), -3.5 (TBS), -4.1 (TBS), -4.1 (TBS); **HRMS (ESI+)** *m*/*z*: $[M+H]^+$ calcd. for $C_{40}H_{76}O_7Si_2H^+$ 725.5202, found 725.5203.

Synthesis of compound 36

(5*S*,7*R*,10*S*,11*R*,12*S*,13*S*)-7,11,13-Tris((*tert*-butyldimethylsilyl)oxy)-5-((*E*)-4-((4-methoxybenzyl)oxy)-3-methylbut-2-en-1-yl)-2,2,3,3,10,12,19,19,20,20-decamethyl-4,18-dioxa-3,19-disilahenicosane

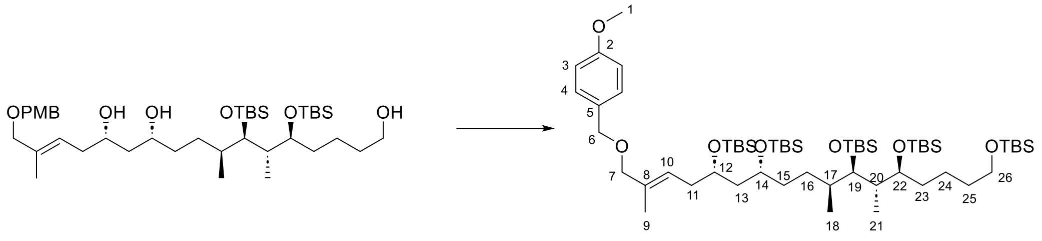

S-32

36
$C_{58}H_{118}O_7Si_5$
1068.00 g/mol

2,6-Lutidine (84.0 µL, 720 µmol, 4.5 eq.) and TBSOTf (150 µL, 640 µmol, 4.0 eq.) were added at 0 °C to a solution of diol **S-32** (116 mg, 160 µmol, 1.0 eq.) in dry DCM (3.0 mL). After stirring for 75 min at room temperature, NH_4Cl-solution (aq., sat., 2 mL) and water (2 mL) were added. The phases were separated and the aqueous phase was extracted with DCM (3 x 3 mL). The combined organic phases were dried over $MgSO_4$ and the solvent was removed under reduced pressure. The crude product was purified by flash column chromatography (SiO_2, cyclohexane/ethyl acetate 50:1) yielding product **36** as a colorless oil (150 mg, 141 µmol, 88%).

R*f*: 0.18 (cyclohexane/ethyl acetate 30:1); **$[\alpha]_D^{20}$** = -6.9° (c = 0.72, CH_2Cl_2); **^{1}H-NMR** (500 MHz, CD_2Cl_2, 298 K): *δ* [ppm] = 7.24 (d, 2H, $^3J_{3,4}$ = 8.7 Hz, H-4), 6.86 (d, 2H, $^3J_{3,4}$ = 8.7 Hz, H-3), 5.52 – 5.47 (m, 1H, H-10), 4.35 (s, 2H, H-6), 3.93 – 3.89 (m, 1H, H-22), 3.88 (s, 2H, H-7), 3.87 – 3.83 (m, 1H, H-12), 3.82 – 3.76 (m, 1H, H-14), 3.79 (s, 3H, H-1), 3.61 (t, 2H, $^3J_{25,26}$ = 6.0 Hz, H-26), 3.49 (dd, 1H, $^3J_{19,20}$ = 8.4 Hz, $^3J_{17,19}$ = 1.9 Hz, H-19), 2.27 (dt, 1H, $^2J_{11a,11b}$ = 13.5 Hz, $^3J_{11a,10/12}$ = 6.1 Hz, H-11a), 2.20 (dt, 1H, $^2J_{11a,11b}$ = 13.5 Hz, $^3J_{11b,10/12}$ = 6.6 Hz, H-11b), 1.85 (dqd, 1H, $^3J_{19,20}$ = 8.4 Hz, $^3J_{20,21}$ = 7.3 Hz, $^3J_{20,22}$ = 4.2 Hz, H-20), 1.71 – 1.64 (m, 1H, H-13a), 1.66 (d, 3H, $^4J_{9,10}$ = 1.4 Hz, H-9), 1.63 – 1.55 (m, 2H, H-15a, H-17), 1.51 – 1.45 (m, 5H, H-13b, H-16a, H-24a, H-25), 1.36 – 1.20 (m, 5H, H-15b, H-16b, H-23, H-24b), 0.93 – 0.88 (m, 45H, 5 x TBS), 0.87 (d, 3H, $^3J_{17,18}$ = 7.0 Hz, H-18), 0.81 (d, 3H, $^3J_{20,21}$ = 7.3 Hz, H-21), 0.07 – 0.04 (m, 30H, 5 x TBS); **^{13}C-NMR** (126 MHz, $CDCl_3$, 298 K): *δ* [ppm] = 159.6 (C-2), 134.2 (C-8), 131.5 (C-5), 129.6 (2C, C-4), 124.6 (C-10), 114.0 (2C, C-3), 78.4 (C-19), 76.5 (C-7), 73.2 (C-22), 71.4 (C-6), 70.3 (C-14), 69.9 (C-12), 63.7 (C-26), 55.6 (C-1), 45.3 (C-13), 44.1 (C-20), 37.9 (C-17), 36.3 (C-15), 36.1 (C-11), 34.0 (C-25), 31.6

(C-23), 30.0 (C-16), 26.5 (3C, TBS), 26.2 (6C, TBS), 26.2 (3C, TBS), 26.1 (3C, TBS), 23.0 (C-24), 18.8 (TBS), 18.6 (TBS), 18.4 (TBS), 18.4 (2C, TBS), 14.4 (C-9), 14.2 (C-18), 10.8 (C-21), -3.3 (TBS), -3.5 (TBS), -3.9 (TBS), -4.0 (TBS), -4.1 (TBS), -4.1 (TBS), -4.2 (TBS), -4.3 (TBS), -5.2 (TBS), -5.2 (TBS); **HRMS (ESI+)** *m*/*z*: $[M+NH_4]^+$ calcd. for $C_{58}H_{118}O_7Si_5NH_4^+$ 1084.8062, found 1084.8069.

Synthesis of compound S-33

(5*S*,6*S*,7*R*,8*S*,11R,13*S*,*E*)-5,7,11,13-Tetrakis((*tert*-butyldimethylsilyl)oxy)-17-((4-methoxybenzyl)oxy)-6,8,16-trimethylheptadec-15-en-1-ol

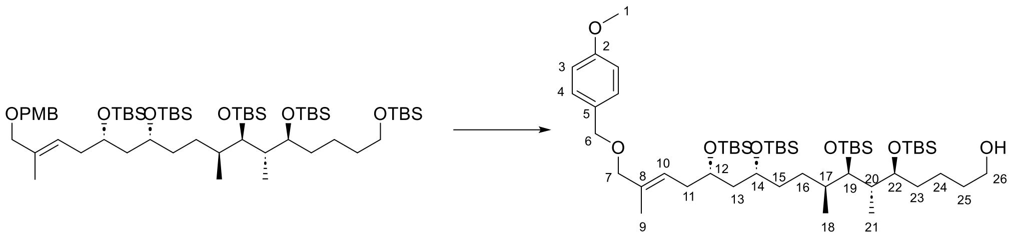

36 **S-33**

$C_{52}H_{104}O_7Si_4$
953.74 g/mol

HF·py (70%, 3.2 mL) was added to dry THF (16.8 mL) and pyridine (10.0 mL) at 0 °C. After stirring for 2 min, the colorless solution was added to TBS-ether **36** (740 mg, 693 µmol, 1.0 eq.) in dry THF (5.5 ml) and stirring at 0 °C continued for 4.5 h. Afterwards, $NaHCO_3$-solution (aq., sat., 30 mL), brine (20 mL) and Et_2O (30 mL) were added and the phases were separated. The aqueous phase was extracted with Et_2O (4 x 20 mL), the combined organic phases were dried over Na_2SO_4 and all solvents were removed under reduced pressure. The crude product was purified by flash column chromatography (SiO_2, cyclohexane/ethyl acetate 10:1) to yield product **S-33** as a colorless oil (535 mg, 561 µmol, 81%).

R*f*: 0.35 (cyclohexane/ethyl acetate 4:1); **$[\alpha]_D^{20}$** = -5.0° (c = 1.00, CH_2Cl_2); **^{1}H-NMR** (500 MHz, CD_2Cl_2, 298 K): *δ* [ppm] = 7.24 (d, 2H, $^3J_{3,4}$ = 8.6 Hz, H-4), 6.86 (d, 2H, $^3J_{3,4}$ = 8.7 Hz, H-3), 5.51 – 5.48 (m, 1H, H-10), 4.36 (d, 1H, $^3J_{6a,6b}$ = 11.6 Hz, H-6a), 4.33 (d, 1H, $^3J_{6a,6b}$ = 11.6 Hz, H-6b), 3.93 – 3.89 (m, 1H, H-22), 3.88 (s, 2H, H-7), 3.88 – 3.83 (m, 1H, H-12), 3.81 – 3.77 (m, 1H, H-14), 3.79 (s, 3H, H-1), 3.59 (td, 2H, $^3J_{25,26}$ = 5.9 Hz, $^3J_{26,OH}$ = 5.9 Hz, H-26), 3.49 (dd, 1H, $^3J_{19,20}$ = 8.3 Hz, $^3J_{17,19}$ = 1.9 Hz, H-19), 2.27 (ddd, 1H, $^2J_{11a,11b}$ = 13.5 Hz, $^3J_{10,11a}$ = 7.1 Hz, $^3J_{11a,12}$ = 5.1 Hz, H-11a), 2.20 (ddd, 1H, $^2J_{11a,11b}$ = 13.5 Hz, $^3J_{10,11b}$ = 7.1 Hz, $^3J_{11b,12}$ = 6.1 Hz, H-11b), 1.85 (dqd, 1H,

$^3J_{19,20}$ = 8.3 Hz, $^3J_{20,21}$ = 7.1 Hz, $^3J_{20,22}$ = 4.2 Hz, H-20), 1.70 – 1.64 (m, 1H, H-13a), 1.66 (d, 3H, $^4J_{9,10}$ = 1.4 Hz, H-9), 1.62 – 1.53 (m, 6H, H-13b, H-15a, H-17, H-24a, H-25), 1.51 – 1.45 (m, 1H, H-16a), 1.38 – 1.33 (m, 2H, H-23), 1.32 – 1.20 (m, 4H, H-15b, H-16b, H-24b, H-OH), 0.91 (s, 9H, TBS), 0.89 (s, 9H, TBS), 0.89 (s, 18H, TBS), 0.87 (d, 3H, $^3J_{17,18}$ = 7.1 Hz, H-18), 0.81 (d, 3H, $^3J_{20,21}$ = 7.1 Hz, H-21), 0.07 – 0.05 (m, 24H, 4 x TBS); **^{13}C-NMR** (126 MHz, $CDCl_3$, 298 K): δ [ppm] = 159.6 (C-2), 134.3 (C-8), 131.4 (C-5), 129.6 (2C, C-4), 124.6 (C-10), 114.0 (2C, C-3), 78.5 (C-19), 76.5 (C-7), 73.1 (C-22), 71.4 (C-6), 70.3 (C-14), 69.9 (C-12), 63.7 (C-26), 55.6 (C-1), 45.3 (C-13), 44.1 (C-20), 38.0 (C-17), 36.3 (C-15), 36.1 (C-11), 33.9 (C-25), 31.4 (C-23), 29.9 (C-16), 26.5 (3C, TBS), 26.2 (3C, TBS), 26.2 (3C, TBS), 26.1 (3C, TBS), 22.7 (C-24), 18.8 (TBS), 18.4 (TBS), 18.4 (2C, TBS), 14.4 (C-9), 14.2 (C-18), 10.8 (C-21), -3.3 (TBS), -3.6 (TBS), -3.9 (TBS), -4.0 (TBS), -4.1 (TBS), -4.1 (TBS), -4.2 (TBS), -4.3 (TBS); **HRMS (ESI+)** *m*/*z*: $[M+K]^+$ calcd. for $C_{52}H_{104}O_7Si_4K^+$ 991.6491, found 991.6504.

Synthesis of compound S-34

(5*S*,6*S*,7*R*,8*S*,11*R*,13*S*,*E*)-5,7,11,13-Tetrakis((*tert*-butyldimethylsilyl)oxy)-17-((4-methoxybenzyl)oxy)-6,8,16-trimethylheptadec-15-enal

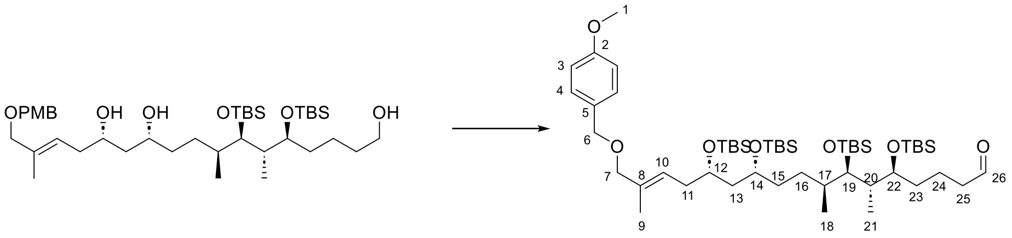

S-33

S-34
$C_{52}H_{102}O_7Si_4$
1068.00 g/mol

DIPEA (70.0 µL, 412 µmol, 4.1 eq.) and DMSO (70.0 µL, 986 mmol, 9.8 eq.) were added to a suspension of py·SO_3 (49.4 mg, 310 µmol, 3.1 eq.) in dry DCM (3.0 mL) at 0 °C. Alcohol **S-33** (96.4 mg, 101 µmol, 1.0 eq.) in dry DCM (2.0 mL) was added and the stirring at room temperature continued for 100 min. Afterwards, $NaHCO_3$-solution (aq., sat., 4 mL) was added, the phases were separated and the aqueous phase was extracted with DCM (5 × 4 mL). The combined organic layers were washed with $CuSO_4$-solution (aq., sat., 2 x 30 mL), NH_4Cl-solution (aq., sat., 2 x 40 mL) and brine (1 x 50 mL) and dried over $MgSO_4$. After removal of all volatiles under reduced pressure, product **S-34** was obtained as pale-yellow oil (94.0 mg, 98.8 µmol, 98%).

R*f*: 0.33 (cyclohexane/ethyl acetate 9:1); **[α]$_D$^{20}** = -7.7° (c = 1.04, CH_2Cl_2); **^{1}H-NMR** (500 MHz, CD_2Cl_2, 298 K): δ [ppm] = 9.72 (t, 1H, $^3J_{25,26}$ = 1.9 Hz, H-26), 7.24 (d, 2H, $^3J_{3,4}$ = 8.7 Hz, H-4), 6.86 (d, 2H, $^3J_{3,4}$ = 8.7 Hz, H-3), 5.51 – 5.47 (m, 1H, H-10), 4.36 (d, 1H, $^2J_{6a,6b}$ = 12.1 Hz, H-6a), 4.33 (d, 1H, $^2J_{6a,6b}$ = 12.1 Hz, H-6b), 3.95 (ddd, 1H, $^3J_{22,23a}$ = 8.4 Hz, $^3J_{22,23b}$ = 4.1 Hz, $^3J_{20,22}$ = 4.1 Hz, H-22), 3.88 (s, 2H, H-7), 3.88 – 3.82 (m, 1H, H-12), 3.81 – 3.76 (m, 1H, H-14), 3.79 (s, 3H, H-1), 3.46 (dd, 1H, $^3J_{19,20}$ = 8.4 Hz, $^3J_{17,19}$ = 1.9 Hz, H-19), 2.39 (dd, 2H, $^3J_{24,25}$ = 7.4 Hz, $^3J_{25,26}$ = 1.9 Hz, H-25), 2.27 (dt, 1H, $^2J_{11a,11b}$ = 13.5 Hz, $^3J_{11a,10/12}$ = 6.2 Hz, H-11a), 2.20 (dt, 1H, $^2J_{11a,11b}$ = 13.5 Hz, $^3J_{11b,10/12}$ = 6.6 Hz, H-11b), 1.86 (ddd, 1H, $^3J_{19,20}$ = 8.4 Hz, $^3J_{20,21}$ = 7.1 Hz, $^3J_{20,22}$ = 4.2 Hz, H-20), 1.83 – 1.77 (m, 1H, H-24a), 1.71 – 1.64 (m, 1H, H-13a), 1.66 (d, 3H, $^4J_{9,10}$ = 1.4 Hz, H-9), 1.64 – 1.52 (m, 4H, H-13b, H-15a, H-17, H-24b), 1.50 – 1.46 (m, 1H, H-16b), 1.39 – 1.32 (m, 2H, H-23), 1.30 – 1.20 (m, 2H, H-15b, H-16b), 0.91 (s, 9H, TBS), 0.90 (s, 9H, TBS), 0.89 (s, 9H, TBS), 0.89 (s, 9H, TBS), 0.88 (d, 3H, $^3J_{17,18}$ = 6.9 Hz, H-18), 0.81 (d, 3H, $^3J_{20,21}$ = 7.1 Hz, H-21), 0.09 – 0.04 (m, 24H, 4 x TBS); **^{13}C-NMR** (126 MHz, $CDCl_3$, 298 K): δ [ppm] = 202.9 (C-26), 159.6 (C-2), 134.3 (C-8), 131.5 (C-5), 129.6 (2C, C-4), 124.6 (C-10), 114.0 (2C, C-3), 78.5 (C-19), 76.5 (C-7), 72.8 (C-22), 71.5 (C-6), 70.3 (C-14), 69.9 (C-12), 55.6 (C-1), 45.3 (C-13), 44.7 (C-25), 44.0 (C-20), 38.1 (C-17), 36.3 (C-15), 36.1 (C-11), 31.1 (C-23), 29.7 (C-16), 26.5 (3C, TBS), 26.2 (3C, TBS), 26.2 (3C, TBS), 26.1 (3C, TBS), 19.4 (C-24), 18.8 (TBS), 18.4 (TBS), 18.4 (2C, TBS), 14.4 (C-9), 14.2 (C-18), 10.7 (C-21), -3.3 (TBS), -3.5 (TBS), -3.9 (TBS), -4.0 (TBS), -4.1 (TBS), -4.1 (TBS), -4.2 (TBS), -4.3 (TBS); **HRMS (ESI+)** *m*/*z*: $[M+Na]^+$ calcd. for $C_{52}H_{102}O_7Si_4Na^+$ 973.6596, found 973.6602.

Synthesis of compound 37

(7*S*,8*S*,9*R*,10*S*,13*R*,15*S*,*E*)-7,9,13,15-Tetrakis((*tert*-butyldimethylsilyl)oxy)-19-((4-methoxybenzyl)oxy)-8,10,18-trimethylnonadec-17-en-3-one

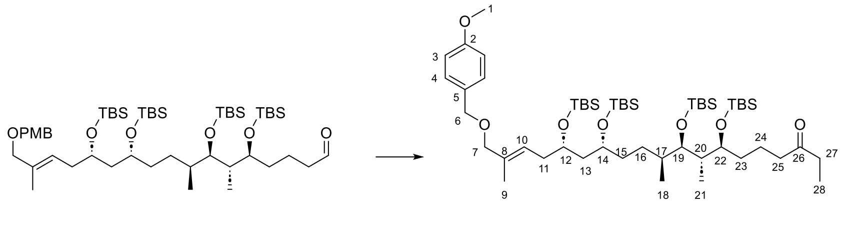

S-34 **37**

$C_{54}H_{106}O_7Si_4$
979.78 g/mol

EtMgBr (3.0 M in Et_2O, 331 µmol, 3.5 eq.) was added dropwise to a cooled solution of aldehyde S-**34** (270 mg, 284 µmol, 1.0 eq.) in dry THF (1.4 mL) at 0 °C. After stirring at 0 °C for 1 h, NH_4Cl-solution (aq., sat., 2 mL) and water (2 mL) were added and the phases were separated. The aqueous phase was extracted with Et_2O (3 x 3 mL) and the combined extracts were dried over Na_2SO_4 and concentrated *in vacuo*. Column chromatographic purification yielded the corresponding secondary alcohol (236 mg, 241 µmol, 85%) as a colorless resin. Afterwards, DMP (102 mg, 229 µmol, 2.1 eq.) was added at 0 °C to a cooled solution of the alcohol (107 mg, 109 µmol, 1.0 eq.) in dry DCM (3.0 mL). The reaction mixture was stirred for 3 h at room temperature, followed by addition of $Na_2S_2O_3$-solution (aq., sat., 2 mL) and $NaHCO_3$-solution (aq., sat., 2 mL). After 10 min of vigorous stirring, the phases were separated, aqueous layer was extracted with DCM (3 x 3.0 mL) and the combined organic extracts were dried over Na_2SO_4 and concentrated *in vacuo*. Purification by flash column chromatography (SiO_2, cyclohexane/ethyl acetate 20:1) yielded ketone **37** (92.2 mg, 94.8 µmol, 87%) as a colorless resin.

R_f: 0.34 (cyclohexane/ethyl acetate 10:1); **$[\alpha]_D^{20}$** = -6.6° (c = 0.60, CH_2Cl_2); **^{1}H-NMR** (700 MHz, CD_2Cl_2, 298 K): δ [ppm] = 7.24 (d, 2H, $^3J_{3,4}$ = 8.7 Hz, H-4), 6.85 (d, 2H, $^3J_{3,4}$ = 8.7 Hz, H-3), 5.48 (t, 1H, $^3J_{10,11}$ = 7.2 Hz, H-10), 4.35 (d, 1H, $^2J_{6a,6b}$ = 11.6 Hz, H-6a), 4.32 (d, 1H, $^2J_{6a,6b}$ = 11.6 Hz, H-6b), 3.94 – 3.88 (m, 1H, H-22), 3.87 (s, 2H, H-7), 3.86 – 3.81 (m, 1H, H-12), 3.80 – 3.75 (m, 1H, H-14), 3.78 (s, 3H, H-1), 3.44 (dd, 1H, $^3J_{19,20}$ = 8.0 Hz, $^3J_{17,19}$ = 1.6 Hz, H-19), 2.41 – 2.34 (m, 4H, H-25, H-27), 2.30 – 2.15 (m, 2H, H-11), 1.87 – 1.80 (m, 1H, H-20), 1.76 – 1.62 (m, 5H, H-9, H-13a, H-24a), 1.62 – 1.41 (m, 6H, H-13b, H-15, H-16a, H-17, H-24b), 1.32 – 1.18 (m, 3H, H-16b,

H-23), 1.00 (t, 3H, $^3J_{27,28}$ = 7.3 Hz, H-28), 0.92 – 0.84 (m, 39H, H-18, 4 x TBS), 0.79 (d, 3H, $^3J_{20,21}$ = 7.1 Hz, H-21), 0.08 – 0.03 (m, 24H, TBS); **^{13}C-NMR** (176 MHz, CD_2Cl_2, 298 K): δ [ppm] = 211.5 (C-26), 159.5 (C-2), 134.2 (C-8), 131.4 (C-5), 129.6 (2C, C-4), 124.6 (C-10), 114.0 (2C, C-3), 78.4 (C-19), 76.5 (C-7), 72.9 (C-22), 71.4 (C-6), 70.2 (C-14), 69.9 (C-12), 55.6 (C-1), 45.3 (C-13), 44.0 (C-20), 43.2 (C-25), 37.9 (C-17), 36.2 (C-15), 36.0 (C-11), 35.9 (C-27), 31.1 (C-23), 29.8 (C-16), 26.5 (3C, TBS), 26.2 (3C, TBS), 26.1 (3C, TBS), 26.1 (3C, TBS), 21.1 (C-24), 18.8 (TBS), 18.4 (TBS), 18.3 (TBS), 18.3 (TBS), 14.4 (C-9), 14.1 (C-18), 10.7 (C-28), 8.0 (C-21), -3.4 (TBS), -3.6 (TBS), -4.0 (TBS), -4.0 (TBS), -4.1 (TBS), -4.2 (TBS), -4.3 (TBS), -4.4 (TBS); **HRMS (ESI+)** *m*/*z*: $[M+Na]^+$ calcd. for $C_{54}H_{106}O_7Si_4Na^+$ 1001.6908, found 1001.6918.

Synthesis of compound 39

(5*R*,6*R*,9*R*,10*R*,15*S*,16*S*,17*R*,18*S*,21*R*,23*S*,*E*)-15,17,21-Tris((*tert*-butyldimethylsilyl)oxy)-3,3,5-triethyl-9-hydroxy-23-((*E*)-4-((4-methoxybenzyl)oxy)-3-methylbut-2-en-1-yl)-6,8,10,16,18,25,25,26,26-nonamethyl-4,24-dioxa-3,25-disilaheptacos-7-en-11-one

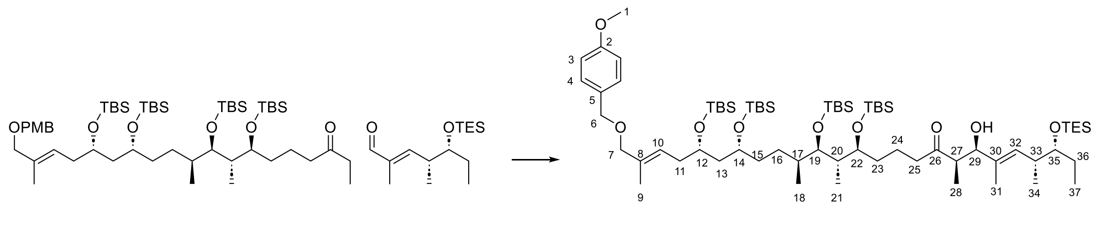

37 **38** **39**

$C_{69}H_{136}O_9Si_5$
1250.26 g/mol

Freshly prepared (+)-Ipc_2BOTf (~1.9 M solution in *n*-hexane, 30.0 µL, 57.0 µmol, 1.3 eq.) was added dry Et_2O (0.25 mL) at −78 °C. DIPEA (15.7 µL, 92.2 µmol, 2.1 eq.) and ketone **37** (dried overnight over 3 Å molecular sieves, 43.0 mg, 44.0 µmol, 1.0 eq.), dissolved in dry Et_2O (0.4 mL), were added dropwise and stirring continued for 3 h. To the resulting suspension, aldehyde **38** (dried overnight over 3 Å molecular sieves, 15.4 mg, 57.1 µmol, 1.3 eq.) in dry Et_2O (0.3 mL) was added dropwise at –78 °C and stirring continued for 1 h at –78 °C, before the reaction flask was sealed and stored in the freezer overnight (–22 °C). Methanol (1.0 mL), pH 7 buffer (2.0 mL) and H_2O_2 (35%, 1.0 mL) were added at 0 °C and the mixture was stirred for 1 h at room temperature. After addition of Et_2O (2 mL) and brine (2 mL) and phase separation, the aqueous layer was extracted with Et_2O (3 x 2 mL) and the extract was washed with $NaHCO_3$-solution (aq., sat., 4 mL) before drying over $MgSO_4$ and concentration *in*

vacuo. Flash column chromatographic purification (SiO_2, cyclohexane/ethyl acetate 40:1 → 10:1) yielded hydroxyketone **39** as a colorless oil (5.80 mg, 4.60 µmol, 10%).

R_f: 0.09 (cyclohexane/ethyl acetate 10:1); **$[\alpha]_D^{20}$** = -2.6° (c = 0.78, CH_2Cl_2); **^{1}H-NMR** (500 MHz, CD_2Cl_2, 298 K): δ [ppm] = 7.24 (d, 2H, $^3J_{3,4}$ = 8.7 Hz, H-4), 6.85 (d, 2H, $^3J_{3,4}$ = 8.7 Hz, H-3), 5.49 (t, 1H, $^3J_{10,11}$ = 7.2 Hz, H-10), 5.35 – 5.29 (m, 1H, H-32), 4.35 (d, 1H, $^2J_{6a,6b}$ = 11.6 Hz, H-6a), 4.32 (d, 1H, $^2J_{6a,6b}$ = 11.6 Hz, H-6b), 4.25 (dd, 1H, $^3J_{27,29}$ = 4.1 Hz, $^3J_{29,OH}$ = 3.3 Hz, H-29), 3.91 (dt, 1H, $^3J_{20,22}$ = 7.7 Hz, $^3J_{22,23}$ = 3.9 Hz, H-22), 3.87 (s, 2H, H-7), 3.87 – 3.81 (m, 1H, H-12), 3.80 – 3.75 (m, 1H, H-14), 3.78 (s, 3H, H-1), 3.48 (td, 1H, $^3J_{35,36}$ = 6.3 Hz, $^3J_{33,35}$ = 3.5 Hz, H-35), 3.44 (dd, 1H, $^3J_{19,20}$ = 8.6 Hz, $^3J_{17,19}$ = 1.8 Hz, H-19), 2.74 (qd, 1H, $^3J_{27,28}$ = 7.1 Hz, $^3J_{27,29}$ = 4.1 Hz, H-27), 2.61 (d, 1H, $^3J_{29,OH}$ = 2.8 Hz, H-OH), 2.55 (dqd, 1H, $^3J_{32,33}$ = 9.7 Hz, $^3J_{33,34}$ = 7.1 Hz, $^3J_{33,35}$ = 3.5 Hz, H-33), 2.47 (t, 2H, $^3J_{24,25}$ = 7.4 Hz, H-25), 2.23 (ddd, 1H, $^2J_{11a,11b}$ = 13.6 Hz, $^3J_{10,11a}$ = 6.3 Hz, $^3J_{11a,12}$ = 5.9 Hz, H-11a), 2.19 (dt, 1H, $^2J_{11a,11b}$ = 13.6 Hz, $^3J_{11b,12}$ = 6.8 Hz, $^3J_{10,11b}$ = 6.4 Hz, H-11b), 1.87 – 1.80 (m, 1H, H-20), 1.77 – 1.64 (m, 2H, H-13a, H-24a), 1.65 (d, 3H, $^4J_{9,10}$ = 1.3 Hz, H-9), 1.62 – 1.58 (m, 1H, H-15a), 1.58 (d, 3H, $^4J_{31,32}$ = 1.3 Hz, H-31), 1.52 – 1.42 (m, 4H, H-13b, H-16a, H-17, H-24b), 1.42 – 1.36 (m, 2H, H-36), 1.31 – 1.19 (m, 4H, H-15b, H-16b, H-23), 1.00 (d, 3H, $^3J_{27,28}$ = 7.1 Hz, H-28), 0.97 (t, 9H, $^3J_{TES,TES}$ = 7.9 Hz, TES), 0.93 (d, 3H, $^3J_{33,34}$ = 7.1 Hz, H-34), 0.90 (s, 9H, TBS), 0.89 (s, 9H, TBS), 0.88 (s, 9H, TBS), 0.88 (s, 9H, TBS), 0.86 (d, 3H, $^3J_{17,18}$ = 6.9 Hz, H-18), 0.83 (t, 3H, $^3J_{36,37}$ = 7.4 Hz, H-37), 0.79 (d, 3H, $^3J_{20,21}$ = 7.1 Hz, H-21), 0.61 (q, 6H, $^3J_{TES,TES}$ = 7.9 Hz, TES), 0.06 (s, 3H, TBS), 0.05 (s, 12H, TBS), 0.05 (s, 9H, TBS); **^{13}C-NMR** (126 MHz, CD_2Cl_2, 298 K): δ [ppm] = 215.4 (C-26), 159.5 (C-2), 134.2 (C-8), 133.3 (C-30), 131.4 (C-5), 129.7 (2C, C-4), 129.6 (C-32), 124.6 (C-10), 114.0 (2C, C-3), 78.4 (C-19), 77.7 (C-35), 76.5 (C-7), 75.6 (C-29), 72.9 (C-22), 71.4 (C-6), 70.2 (C-14), 69.9 (C-12), 55.6 (C-1), 48.5 (C-27), 45.3 (C-13), 44.0 (C-20), 42.8 (C-25), 37.9 (C-17), 37.5 (C-33), 36.2 (C-15), 36.0 (C-11), 31.0 (C-23), 29.8 (C-16), 27.9 (C-36), 26.2 (3C, TBS), 26.1 (3C, TBS), 26.1 (3C, TBS), 26.1 (3C, TBS), 20.7 (C-24), 18.8 (TBS), 18.4 (TBS), 18.3 (TBS), 18.3 (TBS), 17.2 (C-34), 14.4 (C-9), 14.1 (C-18), 13.6 (C-31), 10.7 (C-28), 10.3 (C-37), 9.0 (C-21), 7.2 (3C, TES), 5.6 (3C, TES), -3.4 (TBS), -3.5 (TBS), -4.0 (TBS), -4.0 (TBS), -4.1 (TBS), -4.1 (TBS), -4.3 (TBS), -4.4 (TBS); **HRMS (ESI+)** *m*/*z*: $[M+H]^+$ calcd. For $C_{69}H_{136}O_9Si_5H^+$ 1249.9103, found 1249.9097.

Synthesis of compound 42[2]

(6*R*,7*E*,9*R*,10*R*,12*E*,15*S*,16*S*,17*R*,18*S*,21*R*,23*S*)-15,17,21-Tris((*tert*-butyldimethylsilyl)oxy)-23-((*E*)-4-((4-methoxybenzyl)oxy)-3-methylbut-2-en-1-yl)-2,2,3,3,6,8,10,16,18,25,25,26,26-tridecamethyl-9-((trimethylsilyl)oxy)-4,24-dioxa-3,25-disilaheptacosa-7,12-dien-11-one

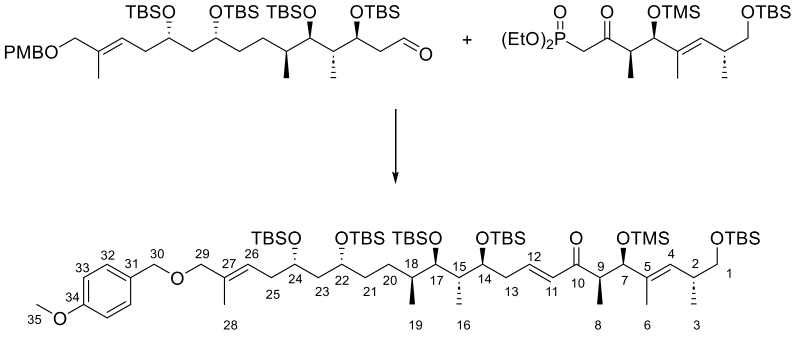

42
$C_{70}H_{138}O_9Si_6$
1292.38 g/mol

To a stirred solution of phosphonate **41**[2] (190 mg, 363 µmol, 1.7 eq.) in dry THF (5.2 mL) was added anhydrous $Ba(OH)_2$ (62.1 mg, 363 µmol, 1.7 eq.) and the suspension was stirred for 75 min at room temperature. Then, a solution of aldehyde **40**[2] (197 mg, 213 µmol, 1.0 eq.) in THF/H_2O (40:1, 5.3 mL) was added and the mixture was stirred for 20 h. Afterwards, NH_4Cl-solution (aq., sat., 5 mL) and water (5 mL) were added and the aqueous phase was extracted with Et_2O (3 × 10 mL). The combined organic phases were dried over Na_2SO_4 and the volatiles were removed under reduced pressure. Purification of the crude product by flash column chromatography (SiO_2, cyclohexane/ethyl acetate 100:0 → 25:1) yielded enone **42** as a colorless syrup (241 mg, 187 µmol, 87%, *d.r.* > 20:1).

R*f*: 0.54 (cyclohexane/ethyl acetate 9:1); **$[\alpha]_D^{20}$** = -8.7° (c = 0.92, $CHCl_3$); **^{1}H-NMR** (700 MHz, CD_2Cl_2, 298 K): *δ* [ppm] = 7.26 – 7.22 (m, 2H, H-32), 6.87 – 6.84 (m, 2H, H-33), 6.81 (dt, 1H, $^3J_{11,12}$ = 15.7 Hz, $^3J_{12,13}$ = 7.3 Hz, H-12), 6.11 (dt, 1H, $^3J_{11,12}$ = 15.7 Hz, $^4J_{11,13}$ = 1.3 Hz, H-11), 5.50 – 5.47 (m, 1H, H-26), 5.08 (dq, 1H, $^3J_{2,4}$ = 9.4 Hz, $^4J_{4,6}$ = 1.2 Hz, H-4), 4.35 (d, 1H, $^2J_{30a,30b}$ = 11.4 Hz, H-30a), 4.33 (d, 1H, $^2J_{30a,30b}$ = 11.3 Hz, H-30b), 4.13 (dd, 1H, $^3J_{7,9}$ = 7.4 Hz, $^4J_{6,7}$ = 0.9 Hz, H-7), 4.07 (dt, 1H, $^3J_{14,15}$ = 8.0 Hz, $^3J_{13,14}$ = 3.8 Hz, H-14), 3.90 – 3.86 (m, 2H, H-29), 3.87 – 3.82 (m, 1H, H-24), 3.81 – 3.75 (m, 4H, H-22, H-35), 3.47 – 3.42 (m, 2H, H-1a, H-17), 3.33 (dd, 1H, $^2J_{1a,1b}$ = 9.7 Hz, $^3J_{1b,2}$ = 7.3 Hz, H-1b), 2.92 (dq, 1H, $^3J_{7,9}$ = 7.4 Hz, $^3J_{8,9}$ = 6.9 Hz, H-9), 2.50 – 2.42 (m, 1H, H-2), 2.30 – 2.23 (m, 3H, H-13, H-25a), 2.23 – 2.17 (m, 1H,

H-25b), 1.91 – 1.84 (m, 1H, H-15), 1.70 – 1.64 (m, 1H, H-23a), 1.66 (d, 3H, $^{4}J_{26,28}$ = 1.3 Hz, H-28), 1.64 – 1.60 (m, 1H, H-21a), 1.58 (d, 3H, $^{4}J_{4,6}$ = 1.3 Hz, H-6), 1.65 – 1.53 (m, 1H, H-16), 1.55 – 1.47 (m, 2H, H-20a, H-23b), 1.26 – 1.19 (m, 2H, H-20b, H-21b), 1.02 (d, 3H, $^{3}J_{8,9}$ = 6.9 Hz, H-8), 0.91 – 0.89 (m, 9H, TBS), 0.89 – 0.87 (m, 39H, H-19, TBS), 0.84 (d, 3H, $^{3}J_{2,3}$ = 6.7 Hz, H-3), 0.84 (d, 3H, $^{3}J_{15,16}$ = 7.1 Hz, H-16), 0.09 – 0.00 (m, 39H, TBS, TMS); **^{13}C-NMR** (176 MHz, CD_2Cl_2, 298 K): δ [ppm] = 201.8 (C-10), 159.5 (C-34), 144.9 (C-12), 135.6 (C-5), 134.2 (C-27), 131.9 (C-11), 131.4 (C-31), 130.8 (C-4), 129.6 (2C, C-32), 124.5 (C-26), 114.0 (2C, C-33), 79.7 (C-7), 78.5 (C-17), 76.4 (C-29), 71.6 (C-14), 71.4 (C-30), 70.2 (C-22), 69.9 (C-24), 68.0 (C-1), 55.6 (C-35), 48.5 (C-9), 45.3 (C-23), 44.4 (C-15), 38.0 (C-18), 36.2 (C-21), 36.0 (C-25), 35.6 (C-2), 35.2 (C-13), 29.8 (C-20), 26.5 (3C, TBS), 26.2 (3C, TBS), 26.1 (3C, TBS), 26.1 (3C, TBS), 26.0 (3C, TBS), 18.7 (TBS), 18.6 (TBS), 18.3 (TBS), 18.3 (TBS), 18.3 (TBS), 17.2 (C-3), 14.4 (C-28), 14.2 (C-19), 12.9 (C-8), 12.3 (C-6), 10.6 (C-16), 0.2 (3C, TMS), -3.3 (TBS), -3.3 (TBS), -4.0 (TBS), -4.1 (TBS), -4.1 (TBS), -4.2 (TBS), -4.2 (TBS), -4.4 (TBS), -5.3 (TBS), -5.3 (TBS); **HRMS (ESI+)** *m*/*z*: $[M+H]^+$ calcd. for $C_{70}H_{138}O_9Si_6H^+$ 1292.9053, found 1292.9065.

Synthesis of compound S-35[2]

(6*R*,9*R*,10*R*,15*S*,16*S*,17*R*,18*S*,21*R*,23*S*,*E*)-15,17,21-Tris((*tert*-butyldimethylsilyl)oxy)-23-((*E*)-4-((4-methoxybenzyl)oxy)-3-methylbut-2-en-1-yl)-2,2,3,3,6,8,10,16,18,25,25,26,26-tridecamethyl-9-((trimethylsilyl)oxy)-4,24-dioxa-3,25-disilaheptacos-7-en-11-one

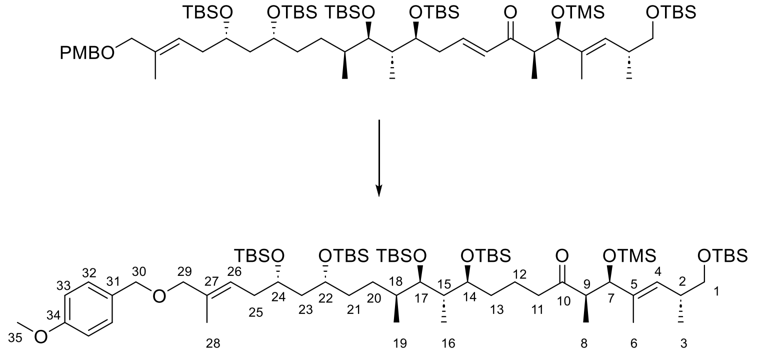

S-35
$C_{70}H_{140}O_9Si_6$
1294.39 g/mol

The 'hot Stryker' stock solution was prepared according to a literature procedure, developed by Lipshutz and co-workers. To a solution of enone **42** (236 mg, 182 µmol, 1.0 eq.) in dry toluene (10 mL) was added dry *t*-BuOH (83.0 µL, 182 µmol, 5.0 eq.) and the mixture was degassed by argon bubbling for 60 min. Then, the "hot Stryker" stock solution (1.30 mL, 13.0 µmol, 7.1 mol%) was added and the reaction was stirred at 45 °C for 22 h. Afterwards, additional "hot Stryker" stock solution (1.30 mL, 13.0 µmol, 7.1 mol%) was added and the mixture was stirred for an additional 26 h at 45 °C. The mixture was filtered over Celite® and washed with ethyl acetate. The volatiles were removed under reduced pressure and the crude product was purified by flash column chromatography (SiO_2, cyclohexane/ethyl acetate 30:0 → 20:1) to yield ketone **S-35** (234 mg, 181 µmol, 99%) as a colorless syrup.

R*f*: 0.55 (cyclohexane/ethyl acetate 9:1); **$[\alpha]_D^{20}$** = -5.8° (c = 0.52, $CHCl_3$); **^{1}H-NMR** (500 MHz, CD_2Cl_2, 298 K): *δ* [ppm] = 7.27 – 7.21 (m, 2H, H-32), 6.88 – 6.83 (m, 2H, H-33), 5.52 – 5.46 (m, 1H, H-26), 5.11 (dq, 1H, $^3J_{2,4}$ = 9.4 Hz, $^4J_{4,6}$ = 1.3 Hz, H-4), 4.38 – 4.31 (m, 2H, H-30), 4.10 (dq, 1H, $^3J_{7,9}$ = 7.4 Hz, $^4J_{6,7}$ = 0.9 Hz, H-7), 3.93 – 3.89 (m, 1H, H-14), 3.89 – 3.87 (m, 2H, H-29), 3.87 – 3.83 (m, 1H, H-24), 3.83 – 3.75 (m, 4H, H-22, H-35), 3.48 – 3.42 (m, 2H, H-1a, H-17), 3.35 (dd, 1H, $^2J_{1a,1b}$ = 9.7 Hz, $^3J_{1b,2}$ = 7.2 Hz, H-1b), 2.68 (dq, 1H, $^3J_{7,9}$ = 7.4 Hz, $^3J_{8,9}$ = 6.9 Hz, H-9), 2.50 (dq, 2H, $^3J_{2,4}$ = 9.2 Hz, $^3J_{2,3}$ = 6.9 Hz, H-2), 2.41 – 2.32 (m, 1H, H-11), 2.27 (dt, 1H, $^2J_{25a,25b}$ = 12.7 Hz, $^3J_{25a,26}$ = 6.1 Hz, H-25a), 2.20 (dt, 1H, $^2J_{25a,25b}$ = 14.1 Hz,

$^{3}J_{25b,26}$ = 6.6 Hz, H-25b), 1.84 (dq, 1H, $^{3}J_{15,17}$ = 11.2 Hz, $^{3}J_{15,16}$ = 7.4 Hz, H-15), 1.72 – 1.67 (m, 2H, H-12a, H-23a), 1.66 (d, 3H, $^{4}J_{26,28}$ = 1.3 Hz, H-28), 1.65 – 1.61 (m, 1H, H-21a), 1.59 (d, 3H, $^{4}J_{4,6}$ = 1.3 Hz, H-6), 1.58 – 1.52 (m, 2H, H-18, H-23b), 1.50 – 1.43 (m, 1H, H-20a), 1.43 – 1.35 (m, 1H, H-12b), 1.33 – 1.22 (m, 4H, H-13, H-20b, H-21b), 1.02 (d, 3H, $^{3}J_{8,9}$ = 6.8 Hz, H-8), 0.90 – 0.88 (m, 48H, H-3, TBS), 0.87 (d, 3H, $^{3}J_{18,19}$ = 6.9 Hz, H-19), 0.79 (d, 3H, $^{3}J_{15,16}$ = 7.1 Hz, H-16), 0.08 – 0.02 (m, 39H, TMS, TBS); **^{13}C-NMR** (126 MHz, CD_2Cl_2, 298 K): *δ* [ppm] = 212.8 (C-10), 159.6 (C-34), 135.8 (C-5), 134.2 (C-27), 131.5 (C-31), 130.9 (C-4), 129.6 (2C, C-32), 124.6 (C-26), 114.0 (2C, C-33), 79.9 (C-7), 78.4 (C-17), 76.5 (C-29), 73.0 (C-14), 71.4 (C-30), 70.3 (C-22), 69.9 (C-24), 68.1 (C-1), 55.6 (C-35), 51.0 (C-9), 45.3 (C-23), 44.2 (C-15), 43.5 (C-11), 38.0 (C-18), 36.3 (C-21), 36.1 (C-25), 35.6 (C-2), 31.3 (C-13), 29.9 (C-20), 26.5 (3C, TBS), 26.2 (3C, TBS), 26.2 (3C, TBS), 26.2 (3C, TBS), 26.1 (3C, TBS), 20.5 (C-12), 18.8 (TBS), 18.7 (TBS), 18.4 (TBS), 18.4 (2C, TBS), 17.4 (C-3), 14.4 (C-28), 14.1 (C-19), 12.8 (C-8), 12.2 (C-6), 10.7 (C-16), 0.2 (3C, TMS), -3.3 (TBS), -3.4 (TBS), -3.9 (TBS), -4.0 (TBS), -4.1 (TBS), -4.1 (TBS), -4.2 (TBS), -4.3 (TBS), -5.2 (TBS), -5.3 (TBS); **HRMS (ESI+)** *m*/*z*: $[M+Na]^+$ calcd. for $C_{70}H_{140}O_9Si_6Na^+$ 1315.9005, found 1315.9009.

Synthesis of compound S-36[2]

(6*R*,9*R*,10*R*,15*S*,16*S*,17*R*,18*S*,21*R*,23*S*,*E*)-15,17,21-Tris((*tert*-butyldimethylsilyl)oxy)-9-hydroxy-23-((*E*)-4-((4-methoxybenzyl)oxy)-3-methylbut-2-en-1-yl)-2,2,3,3,6,8,10,16,18,25,25,26,26-tridecamethyl-4,24-dioxa-3,25-disilaheptacos-7-en-11-one

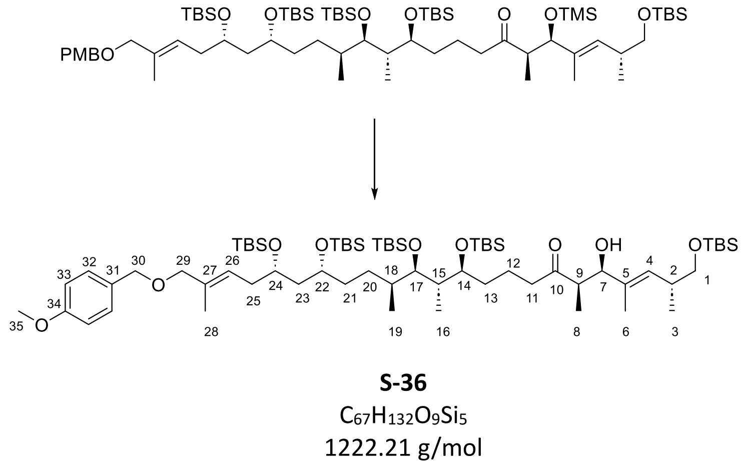

S-36
$C_{67}H_{132}O_9Si_5$
1222.21 g/mol

To an ice-cooled solution of ketone **S-35** (201 mg, 155 µmol, 1.0 eq.) in dry THF (7.5 mL) and MeOH (7.5 mL) was added pyridinium *p*-toluenesulfonate (11.7 mg,

46.5 µmol, 0.30 eq.) and the reaction was allowed to slowly come to room temperature. After the mixture was stirred for 6 h, triethylamine (20 µL) was added and the volatiles were removed under reduced pressure. The crude product was purified by flash column chromatography (SiO_2, cyclohexane/ethyl acetate 15:1 → 4:1), yielding alcohol **S-36** (154 mg, 126 µmol, 81%) as a colorless syrup.

R*f*: 0.12 (cyclohexane/ethyl acetate 10:1); **$[\alpha]_D^{20}$** = -5.2° (c = 0.58, $CHCl_3$); **^{1}H-NMR** (500 MHz, CD_2Cl_2, 298 K): *δ* [ppm] = 7.27 – 7.21 (m, 2H, H-32), 6.89 – 6.82 (m, 2H, H-33), 5.51 – 5.46 (m, 1H, H-26), 5.25 (dq, 1H, $^3J_{2,4}$ = 9.4 Hz, $^4J_{4,6}$ = 1.5 Hz, H-4), 4.36 – 4.33 (m, 2H, H-30), 4.26 (dd, 1H, $^3J_{7,9}$ = 4.0 Hz, $^3J_{7,OH}$ = 3.0 Hz, H-7), 3.92 (ddd, 1H, $^3J_{13,14}$ = 8.6 Hz, $^3J_{14,15}$ = 4.5 Hz, $^3J_{13,14}$ = 4.0 Hz, H-14), 3.89 – 3.86 (m, 2H, H-29), 3.87 – 3.82 (m, 1H, H-24), 3.81 – 3.75 (m, 4H, H-22, H-35), 3.48 – 3.43 (m, 2H, H-1a, H-17), 3.39 (dd, 1H, $^2J_{1a,1b}$ = 9.7 Hz, $^3J_{1b,2}$ = 7.0 Hz, H-1b), 2.71 (qd, 1H, $^3J_{8,9}$ = 7.1 Hz, $^3J_{7,9}$ = 4.0 Hz, H-9), 2.60 – 2.54 (m, 2H, H-2, H-OH), 2.48 (t, 2H, $^3J_{11,12}$ = 7.4 Hz, H-11), 2.27 (dt, 1H, $^2J_{25a,25b}$ = 12.7 Hz, $^3J_{24/26,25a}$ = 6.1 Hz, H-25a), 2.20 (dt, 1H, $^2J_{25a,25b}$ = 13.9 Hz, $^3J_{24/26,25b}$ = 6.5 Hz, H-25b), 1.85 (ddd, 1H, $^3J_{15,17}$ = 7.4 Hz, $^3J_{15,16}$ = 7.0 Hz, $^3J_{14,15}$ = 4.5 Hz, H-15), 1.79 – 1.70 (m, 1H, H-12a), 1.69 – 1.63 (m, 1H, H-23a), 1.66 (d, 3H, $^4J_{26,28}$ = 1.3 Hz, H-28), 1.63 – 1.59 (m, 1H, H-21a), 1.59 (d, 3H, $^4J_{4,6}$ = 1.3 Hz, H-6), 1.57 – 1.41 (m, 4H, H-12b, H-18, H-20a, H-23b), 1.36 – 1.17 (m, 4H, H-13, H-20b, H-21b), 1.01 (d, 3H, $^3J_{8,9}$ = 7.1 Hz, H-8), 0.93 (d, 3H, $^3J_{2,3}$ = 6.7 Hz, H-3), 0.91 – 0.88 (m, 45H, TBS), 0.87 (d, 3H, $^3J_{18,19}$ = 6.9 Hz, H-19), 0.80 (d, 3H, $^3J_{15,16}$ = 7.0 Hz, H-16), 0.08 – 0.02 (m, 30H, TBS); **^{13}C-NMR** (126 MHz, CD_2Cl_2, 298 K): *δ* [ppm] = 215.4 (C-10), 159.6 (C-34), 134.5 (C-5), 134.2 (C-27), 131.4 (C-31), 129.6 (3C, C-4, C-32), 124.6 (C-26), 114.0 (2C, C-33), 78.5 (C-17), 76.5 (C-29), 75.6 (C-7), 72.9 (C-14), 71.4 (C-30), 70.3 (C-22), 69.9 (C-24), 68.4 (C-1), 55.6 (C-35), 48.4 (C-9), 45.3 (C-23), 44.1 (C-15), 42.8 (C-11), 38.0 (C-18), 36.3 (C-21), 36.1 (C-25), 35.7 (C-2), 31.1 (C-13), 29.8 (C-20), 26.5 (3C, TBS), 26.2 (3C, TBS), 26.2 (3C, TBS), 26.1 (6C, TBS), 20.8 (C-12), 18.8 (TBS), 18.6 (TBS), 18.4 (TBS), 18.4 (2C, TBS), 17.4 (C-3), 14.4 (C-28), 14.1 (C-19), 13.8 (C-6), 10.7 (C-16), 10.2 (C-8), -3.3 (TBS), -3.5 (TBS), -3.9 (TBS), -4.0 (TBS), -4.1 (TBS), -4.1 (TBS), -4.2 (TBS), -4.3 (TBS), -5.2 (TBS), -5.3 (TBS); **HRMS (ESI+)** *m/z*: $[M+NH_4]^+$ calcd. for $C_{67}H_{132}O_9Si_5NH_4^+$ 1238.9056, found 1238.9053.

Synthesis of compound 43[2]

(6*R*,9*R*,10*S*,11*R*,15*S*,16*S*,17*R*,18*S*,21*R*,23*S*,*E*)-15,17,21-Tris((*tert*-butyldimethylsilyl)oxy)-23-((*E*)-4-((4-methoxybenzyl)oxy)-3-methylbut-2-en-1-yl)-2,2,3,3,6,8,10,16,18,25,25,26,26-tridecamethyl-4,24-dioxa-3,25-disilaheptacos-7-ene-9,11-diol-2,2,3,3,6,8,10,16,18,25,25,26,26-tridecamethyl-4,24-dioxa-3,25-disilaheptacos-7-en-11-one

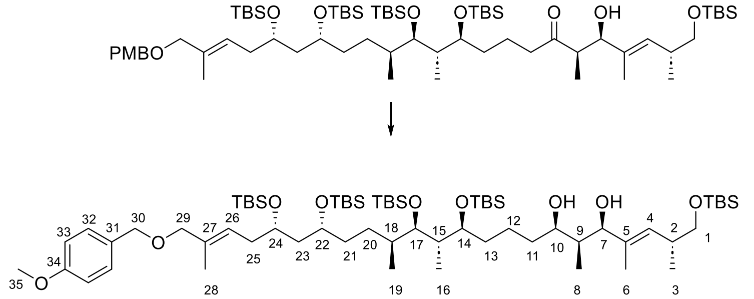

43
$C_{67}H_{134}O_9Si_5$
1224.22 g/mol

β-Hydroxy ketone **S-36** (148 mg, 121 µmol, 1.0 eq.) was dissolved in a mixture of dry THF (2.7 mL) and MeOH (0.8 mL) and the solution was cooled to -78 °C. Then, diethyl methoxyborane (1.0 M in THF, 206 µL, 206 µmol, 1.7 eq.) was added dropwise and the resulting mixture was stirred for 20 min at -78 °C. Sodium borohydride (6.89 mg, 182 µmol, 1.5 eq.) was added and the mixture was stirred for additional 3 h at the indicated temperature. Afterwards, the cooling bath was removed and NaOH solution (1.0 M in H_2O, 4 mL) and H_2O_2 solution (35%, 0.8 mL) were added. The mixture was stirred for 2 h at room temperature. Then, $Na_2S_2O_3$-solution (aq., sat., 4 mL) was added carefully and the mixture was stirred for 10 min at room temperature. The aqueous phase was extracted with Et_2O (3 × 20 mL) and the combined organic phases were dried over Na_2SO_4. Removal of the volatiles *in vacuo* and purification of the crude product by flash column chromatography (SiO_2, cyclohexane/ethyl acetate 10:1 → 5:1) yielded diol **43** (141 mg, 115 µmol, 95%, *d.r.* > 20:1) as a colorless syrup.

R_f: 0.31 (cyclohexane/ethyl acetate 4:1); **$[\alpha]_D^{20}$** = -12.0° (c = 0.50, $CHCl_3$); **^{1}H-NMR** (500 MHz, CD_2Cl_2, 298 K): δ [ppm] = 7.27 – 7.21 (m, 2H, H-32), 6.88 – 6.83 (m, 2H, H-33), 5.51 – 5.47 (m, 1H, H-26), 5.24 (dt, 1H, $^3J_{2,4}$ = 9.4 Hz, $^4J_{4,6}$ = 1.4 Hz, H-4), 4.38 – 4.32 (m, 2H, H-30), 4.16 – 4.13 (m, 1H, H-7), 3.94 – 3.89 (m, 1H, H-14), 3.89 – 3.87 (m, 2H, H-29), 3.88 – 3.82 (m, 1H, H-24), 3.83 – 3.80 (m, 1H, H-10), 3.82 – 3.76 (m, 4H, H-22, H-35), 3.51 – 3.44 (m, 2H, H-1a, H-17), 3.40 (dd, 1H, $^2J_{1a,1b}$ = 9.7 Hz, $^3J_{1b,2}$ = 7.1 Hz, H-1b), 2.59 (dq, 1H, $^3J_{2,4}$ = 9.4 Hz, $^3J_{2,3}$ = 6.7 Hz, H-2), 2.45 (d, 1H, $^3J_{7,OH}$ = 2.6 Hz, H-OH), 2.38 (d, 1H, $^3J_{10,OH}$ = 3.4 Hz, H-OH), 2.27 (ddd, 1H,

$^{2}J_{25a,25b}$ = 13.5 Hz, $^{3}J_{25a,26}$ = 6.3 Hz, $^{3}J_{24,25a}$ = 5.9 Hz, H-25a), 2.20 (ddd, 1H, $^{2}J_{25a,25b}$ = 13.5 Hz, $^{3}J_{24,25b}$ = 6.8 Hz, $^{3}J_{25b,26}$ = 6.4 Hz, H-25b), 1.89 – 1.81 (m, 1H, H-15), 1.72 – 1.63 (m, 2H, H-9, H-23a), 1.66 (d, 3H, $^{3}J_{26,28}$ = 1.3 Hz, H-28), 1.64 – 1.60 (m, 1H, H-11a), 1.60 – 1.56 (m, 1H, H-18), 1.58 (d, 3H, $^{3}J_{4,6}$ = 1.4 Hz, H-6), 1.56 – 1.52 (m, 2H, H-12a, H-23b), 1.52 – 1.43 (m, 2H, H-20a, H-21a), 1.44 – 1.37 (m, 1H, H-21b), 1.40 – 1.31 (m, 2H, H-13), 1.32 – 1.25 (m, 2H, H-11b, H-20b), 1.25 – 1.18 (m, 1H, H-12b), 0.95 (d, 3H, $^{3}J_{2,3}$ = 6.7 Hz, H-3), 0.92 – 0.88 (m, 45H, TBS), 0.87 (d, 3H, $^{3}J_{18,19}$ = 6.9 Hz, H-19), 0.81 (d, 3H, $^{3}J_{15,16}$ = 7.1 Hz, H-16), 0.78 (d, 3H, $^{3}J_{8,9}$ = 7.1 Hz, H-8), 0.08 – 0.03 (m, 30H, TBS); **^{13}C-NMR** (126 MHz, CD_2Cl_2, 298 K): *δ* [ppm] = 159.6 (C-34), 136.3 (C-5), 134.3 (C-27), 131.4 (C-31), 129.6 (2C, C-32), 128.1 (C-4), 124.6 (C-26), 114.0 (2C, C-33), 81.0 (C-7), 78.5 (C-17), 76.5 (C-29), 76.4 (C-10), 73.2 (C-14), 71.4 (C-30), 70.3 (C-22), 70.0 (C-24), 68.4 (C-1), 55.6 (C-35), 45.3 (C-23), 44.0 (C-15), 38.8 (C-9), 38.0 (C-18), 36.3 (C-11), 36.3 (C-21), 36.1 (C-25), 35.6 (C-2), 31.5 (C-13), 30.1 (C-20), 26.5 (3C, TBS), 26.2 (3C, TBS), 26.2 (3C, TBS), 26.1 (3C, TBS), 26.1 (3C, TBS), 23.1 (C-12), 18.8 (TBS), 18.6 (TBS), 18.4 (TBS), 18.4 (2C, TBS), 17.5 (C-3), 14.4 (C-28), 14.2 (C-19), 14.1 (C-6), 10.8 (C-16), 4.8 (C-8), -3.3 (TBS), -3.6 (TBS), -3.9 (TBS), -4.0 (TBS), -4.1 (TBS), -4.1 (TBS), -4.2 (TBS), -4.3 (TBS), -5.2 (TBS), -5.3 (TBS); **HRMS (ESI+)** *m*/*z*: $[M+H]^+$ calcd. for $C_{67}H_{134}O_9Si_5H^+$ 1223.8947, found 1223.8936.

Synthesis of compound 7[2]

(2*R*,3*E*,5*R*,6*S*,7*R*,11*S*,12*S*,13*R*,14*S*,17*R*,19*S*,21*E*)-23-((4-Methoxybenzyl)oxy)-2,4,6,12,14,22-hexamethyltricosa-3,21-diene-1,5,7,11,13,17,19-heptaol

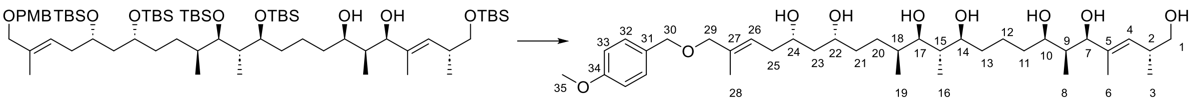

43

7
$C_{37}H_{64}O_9$
652.91 g/mol

To a solution of diol **43** (12.1 mg, 9.91 µmol, 1.0 eq.) in dry MeOH (2 mL) was added HCl (1.0 M in H_2O, 160 µl, 160 µmol, 16.2 eq.). After stirring for 90 h at room temperature, $NaHCO_3$ (15 mg) was added and the suspension was stirred for 10 min. The volatiles were removed under reduced pressure and the crude product was purified by flash column chromatography (SiO_2, CH_2Cl_2/MeOH 6:1). The resulting

product was further purified additionally by HPLC to yield polyol **7** (3.10 mg, 4.75 µmol, 48%) as a colorless oil.

R_f: 0.58 (CH_2Cl_2/MeOH 4:1); **$[\alpha]_D^{20}$** = -9.7° (c = 0.62, MeOH); **^{1}H-NMR** (700 MHz, CD_2Cl_2, 298 K): δ [ppm] = 7.28 – 7.23 (m, 2H, H-32), 6.92 – 6.87 (m, 2H, H-33), 5.54 (tq, 1H, $^3J_{25,26}$ = 7.3 Hz, $^4J_{26,28}$ = 1.3 Hz, H-26), 5.26 (dq, 1H, $^3J_{2,4}$ = 9.4 Hz, $^4J_{4,6}$ = 1.3 Hz, H-4), 4.38 (s, 2H, H-30), 4.00 (dq, 1H, $^3J_{7,9}$ = 7.1 Hz, $^4J_{6,7}$ = 1.0 Hz, H-7), 3.91 (d, 2H, $^4J_{26,29}$ = 1.2 Hz, H-29), 3.86 – 3.82 (m, 1H, H-24), 3.82 – 3.77 (m, 1H, H-14), 3.79 (s, 3H, H-35), 3.76 – 3.72 (m, 1H, H-22), 3.62 (ddd, 1H, $^3J_{10,11}$ = 7.9 Hz, $^3J_{9,10}$ = 5.7 Hz, $^3J_{10,11}$ = 2.4 Hz, H-10), 3.43 – 3.39 (m, 1H, H-1a), 3.39 – 3.35 (m, 2H, H-1b, H-17), 2.62 (dqd, 1H, $^3J_{2,4}$ = 9.4 Hz, $^3J_{2,3}$ = 6.8 Hz, $^3J_{1,2}$ = 6.5 Hz, H-2), 2.31 – 2.22 (m, 2H, H-25), 1.77 – 1.71 (m, 1H, H-15), 1.69 (d, 3H, $^4J_{26,28}$ = 1.3 Hz, H-28), 1.67 – 1.62 (m, 3H, H-23a, H-12a, H-9), 1.61 (d, 3H, $^4J_{4,6}$ = 1.3 Hz, H-6), 1.60 – 1.56 (m, 2H, H-18, H-23b), 1.57 – 1.47 (m, 5H, H-11, H-13a, H-20a, H-21a), 1.46 – 1.40 (m, 1H, H-21b), 1.36 – 1.27 (m, 3H, H-12b, H-13b, H-20b), 0.98 (d, 3H, $^3J_{2,3}$ = 6.8 Hz, H-3), 0.91 (d, 3H, $^3J_{8,9}$ = 6.9 Hz, H-8), 0.84 (d, 3H, $^3J_{18,19}$ = 6.8 Hz, H-19), 0.76 (d, 3H, $^3J_{15,16}$ = 6.9 Hz, H-16); **^{13}C-NMR** (176 MHz, CD_2Cl_2, 298 K): δ [ppm] = 160.8 (C-34), 137.8 (C-5), 135.3 (C-27), 131.6 (C-31), 130.9 (C-4), 130.7 (2C, C-32), 125.7 (C-30), 114.7 (2C, C-33), 81.0 (C-7), 77.7 (C-17), 77.0 (C-29), 74.9 (C-14), 73.9 (C-10), 72.2 (C-30), 71.8 (C-22), 71.5 (C-24), 68.2 (C-1), 55.7 (C-35), 44.4 (C-23), 42.7 (C-15), 40.9 (C-9), 36.8 (C-25), 36.5 (C-11), 36.4 (C-21), 36.3 (C-2), 36.2 (C-18), 33.2 (C-13), 31.3 (C-20), 23.5 (C-12), 17.5 (C-3), 14.4 (C-28), 12.6 (C-19), 12.6 (C-6), 11.7 (C-16), 7.8 (C-8); HRMS (ESI+) *m/z*: $[M+H]^+$ calcd. for $C_{37}H_{64}O_9H^+$ 653.4623, found 653.4622.

HPLC (analytical): t_R = 2.429 min; on System EUROPA+ with MACHEREY-NAGEL Nucleodur 100-3 C18ec; 3 µm; 2.0 mm x 100 mm (N21070272), isocratic MeCN/H_2O = 40:60, flow rate: 0.5 mL/min, total running time: 6.0 min, 25 °C, detection at 225 nm.

HPLC (preparative): t_R = 14.800 min; on System TETHYS + with KNAUER Eurospher II 100-5 C18; 5 µm; 250 x 8 mm + Vorsäule 30 x 8 mm (YF169 + BG270); isocratic MeCN/H_2O = 40:60, flow rate: 4.0 mL/min, total running time: 45min, detection at 225 nm.

Synthesis of Mycosamine 10

Synthesis of compound TBS-48

(2*R*,3*R*,4*R*,5*S*,6*R*)-4-Azido-5-((*tert*-butyldimethylsilyl)oxy)-2-(((7*S*,9*R*,10*S*)-7-((*tert*-butyldimethylsilyl)oxy)-2,2,3,3,10,15,15,16,16-nonamethyl-4,14-dioxa-3,15-disilaheptadecan-9-yl)oxy)-6-methyltetrahydro-2*H*-pyran-3-yl benzoate

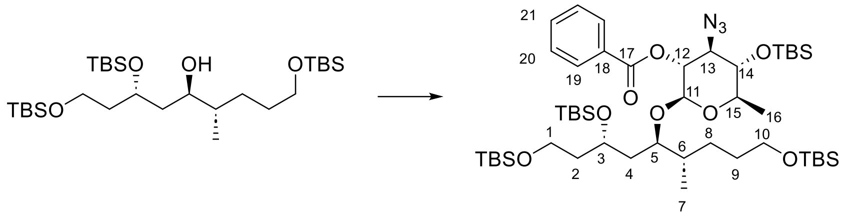

45

TBS-**48**

$C_{47}H_{91}N_3O_8Si_4$

938.60 g/mol

Alcohol **45**[3] (20.0 mg, 36.4 µmol, 1.0 eq.) and sugar donor TBS-**44**[5] (27.7 mg, 50.3 µmol, 1.4 eq.) were dried azeotropically with toluene (2 x 5 mL). Afterwards, they were diluted with dry *n*-hexane (2.0 mL) and 2-chloro-6-methylpyridine (4.00 µL, 36.7 µmol, 1.0 eq.) and 2-chloro-6-methylpyridinium triflate (5.20 mg, 18.7 µmol, 0.5 eq.) were added. After 10 min, a colorless precipitate was formed and stirring continued for 2 h at room temperature. Then, Et_2O (4 mL) and $NaHCO_3$-solution (aq., sat., 4 mL) were added, the phases were separated and the aqueous phase was extracted with Et_2O (5 x 4 mL). The combined organic layers were dried over Na_2SO_4 and all volatiles were removed under reduced pressure. After flash column chromatography (SiO_2, cyclohexane/ethyl acetate 40:1), product TBS-**48** (31.4 mg, 33.5 µmol, 92%, *d.r.* = 12:1) was obtained as a colorless oil.

R_f: 0.41 (cyclohexane/ethyl acetate 19:1); **$[\alpha]_D^{20}$** = +26.0° (c = 1.04, $CHCl_3$); **^{1}H-NMR** (500 MHz, CD_2Cl_2, 298 K): δ [ppm] = 8.06 (dd, 2H, $^3J_{19,20}$ = 8.4 Hz, $^4J_{19,21}$ = 1.3 Hz, H-19), 7.62 – 7.59 (m, 1H, H-21), 7.50 – 7.47 (m, 2H, H-20), 5.04 (dd, 1H, $^3J_{12,13}$ = 10.1 Hz, $^3J_{11,12}$ = 7.9 Hz, H-12), 4.62 (d, 1H, $^3J_{11,12}$ = 7.9 Hz, H-11), 3.64 – 3.59 (m, 2H, H-3, H-5), 3.53 (t, 2H, $^3J_{9,10}$ = 6.6 Hz, H-10), 3.52 (dd, 1H, $^3J_{12,13}$ = 10.1 Hz, $^3J_{13,14}$ = 9.1 Hz, H-13), 3.45 – 3.31 (m, 3H, H-1, H-15), 3.25 (t, 1H, $^3J_{13,14}$ = 9.1 Hz, $^3J_{14,15}$ = 9.0 Hz, H-14), 1.88 – 1.81 (m, 1H, H-6), 1.57 – 1.50 (m, 1H, H-9a), 1.45 – 1.36 (m, 4H, H-2a, H-4, H-9b), 1.34 – 1.20 (m, 2H, H-2b, H-8a), 1.29 (d, 3H, $^3J_{15,16}$ = 6.2 Hz, H-16), 1.08 – 1.01 (m, 1H, H-8b), 0.93 (s, 9H, TBS), 0.87 (s, 9H, TBS), 0.85 (s, 9H, TBS), 0.84 (s, 9H, TBS), 0.82 (d, 3H, $^3J_{6,7}$ = 6.9 Hz, H-7), 0.20 (s, 3H, TBS), 0.14 (s, 3H, TBS), 0.03 (s, 3H, TBS), 0.02 (s, 6H, TBS), -0.02 (s, 3H, TBS),

-0.03 (s, 3H, TBS), -0.10 (s, 3H, TBS); **^{13}C-NMR** (126 MHz, CD_2Cl_2, 298 K): *δ* [ppm] = 165.2 (C-17), 133.8 (C-21), 130.1 (2C, C-19), 130.0 (C-18), 129.0 (2C, C-20), 101.7 (C-11), 82.1 (C-5), 75.4 (C-14), 73.6 (C-15), 73.4 (C-12), 69.3 (C-13), 67.9 (C-3), 63.7 (C-10), 59.3 (C-1), 41.2 (C-2), 38.8 (C-4), 37.6 (C-6), 31.2 (C-9), 28.9 (C-8), 26.1 (3C, TBS), 26.1 (3C, TBS), 26.1 (3C, TBS), 26.0 (3C, TBS), 18.6 (TBS), 18.5 (C-16), 18.4 (TBS), 18.4 (TBS), 18.2 (TBS), 14.6 (C-7), -3.8 (TBS), -3.9 (TBS), -4.1 (TBS), -4.2 (TBS), -5.2 (4C, TBS); **HRMS (ESI+)** *m/z*: $[M + H]^+$ calcd. For $C_{47}H_{91}N_3O_8Si_4H^+$ 938.5956, found 938.5959.

Synthesis of compound S-37

(2*R*,3*R*,4*R*,5*S*,6*R*)-4-Azido-5-((*tert*-butyldimethylsilyl)oxy)-2-(((7*S*,9*R*,10*S*)-7-((*tert*-butyldimethylsilyl)oxy)-2,2,3,3,10,15,15,16,16-nonamethyl-4,14-dioxa-3,15-disilaheptadecan-9-yl)oxy)-6-methyltetrahydro-2*H*-pyran-3-ol

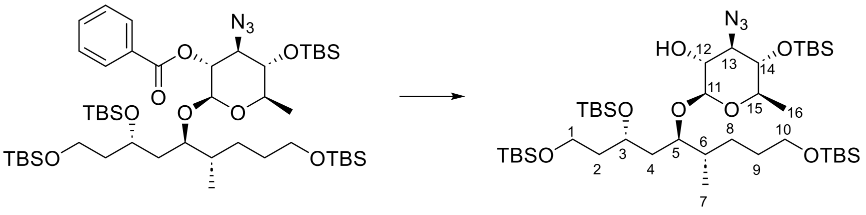

TBS-**48**

S-37
$C_{40}H_{87}N_3O_7Si_4$
834.49 g/mol

NaOMe (5.4 M in MeOH, 80.0 µL, 432 µmol, 12.9 eq.) was added to ester TBS-**48** (31.4 mg, 33.5 µmol, 1.0 eq.) in dry MeOH (3.0 mL). After stirring for 6 h at room temperature, DCM (10 mL) and NH_4Cl-solution (aq., sat., 10 mL) were added. The phases were separated and the aqueous phase was extracted with DCM (3 x 10 mL). The combined organic layers were dried over Na_2SO_4 and all volatiles were removed under reduced pressure. After flash column chromatography (SiO_2, cyclohexane/ethyl acetate 30:1), product **S-37** was obtained as a colorless oil (25.2 mg, 30.2 µmol, 90%). **R_f**: 0.29 (cyclohexane/ethyl acetate 19:1); **$[\alpha]_D^{20}$** = +3.6° (c = 1.11, $CHCl_3$); **^{1}H-NMR** (700 MHz, CD_2Cl_2, 298 K): *δ* [ppm] = 4.28 (d, 1H, $^3J_{11,12}$ = 7.6 Hz, H-11), 4.09 (tdd, 1H, $^3J_{2,3}$ = 6.1 Hz, $^3J_{3,4a}$ = 4.0 Hz, $^3J_{3,4b}$ = 3.4 Hz, H-3), 3.89 (d, 1H, $^3J_{12,OH}$ = 2.5 Hz, H-OH), 3.71 (t, 2H, $^3J_{1,2}$ = 6.1 Hz, H-1), 3.71 – 3.68 (m, 1H, H-5), 3.58 (t, 2H, $^3J_{9,10}$ = 6.5 Hz, H-10), 3.32 – 3.27 (m, 2H, H-12, H-15), 3.22 (dd, 1H, $^3J_{12,13}$ = 9.8 Hz, $^3J_{13,14}$ = 9.1 Hz, H-13), 3.06 (dd, 1H, $^3J_{13,14}$ = 9.1 Hz, $^3J_{14,15}$ = 8.9 Hz,

H-14), 1.92 (dddd, 1H, $^3J_{6,8b}$ = 9.0 Hz, $^3J_{6,7}$ = 6.8 Hz, $^3J_{6,8a}$ = 5.4 Hz, $^3J_{5,6}$ = 3.8 Hz, H-6), 1.80 (ddt, 1H, $^2J_{2a,2b}$ = 13.7 Hz, $^3J_{1,2a}$ = 6.1 Hz, $^3J_{2a,3}$ = 6.1 Hz, H-2a), 1.73 (ddd, 1H, $^2J_{4a,4b}$ = 14.8 Hz, $^3J_{4a,5}$ = 9.8 Hz, $^3J_{3,4a}$ = 4.0 Hz, H-4a), 1.69 (ddt, 1H, $^2J_{2a,2b}$ = 13.7 Hz, $^3J_{1,2b}$ = 6.1 Hz, $^3J_{2b,3}$ = 6.1 Hz, H-2b), 1.60 – 1.56 (m, 1H, H-9a), 1.49 – 1.43 (m, 2H, H-4b, H-9b), 1.32 – 1.27 (m, 1H, H-8a), 1.22 (d, 3H, $^3J_{15,16}$ = 6.2 Hz, H-16), 1.10 (dddd, 1H, $^2J_{8a,8b}$ = 13.7 Hz, $^3J_{8b,9}$ = 10.4 Hz, $^3J_{6,8b}$ = 9.0 Hz, $^3J_{8b,9}$ = 5.1 Hz, H-8b), 0.91 (s, 9H, TBS), 0.91 (s, 9H, TBS), 0.90 (s, 9H, TBS), 0.89 (s, 9H, TBS), 0.86 (d, 3H, $^3J_{6,7}$ = 6.8 Hz, H-7), 0.18 (s, 3H, TBS), 0.11 (s, 3H, TBS), 0.10 (s, 3H, TBS), 0.10 (s, 3H, TBS), 0.08 (s, 3H, TBS), 0.07 (s, 3H, TBS), 0.04 (s, 6H, TBS); **^{13}C-NMR** (176 MHz, CD_2Cl_2, 298 K): δ [ppm] = 105.1 (C-11), 83.9 (C-5), 75.2 (C-14), 74.3 (C-12), 73.6 (C-15), 70.1 (C-13), 68.7 (C-3), 63.6 (C-10), 60.3 (C-1), 40.4 (C-2), 37.5 (C-6), 36.8 (C-4), 31.3 (C-9), 29.6 (C-8), 26.2 (3C, TBS), 26.1 (3C, TBS), 26.1 (3C, TBS), 26.1 (3C, TBS), 18.7 (TBS), 18.7 (TBS), 18.6 (C-16), 18.4 (TBS), 18.4 (TBS), 14.0 (C-7), -4.0 (TBS), -4.1 (TBS), -4.2 (TBS), -4.2 (TBS), -5.1 (TBS), -5.2 (2C, TBS), -5.3 (TBS); **HRMS (ESI+)** *m/z*: $[M + H]^+$ calcd. For $C_{40}H_{87}N_3O_7Si_4H^+$ 834.5694, found 834.5692.

Synthesis of compound TBS-49

(2*R*,3*S*,4*R*,5*S*,6*R*)-4-Azido-5-((*tert*-butyldimethylsilyl)oxy)-2-(((7*S*,9*R*,10*S*)-7-((*tert*-butyldimethylsilyl)oxy)-2,2,3,3,10,15,15,16,16-nonamethyl-4,14-dioxa-3,15-disilaheptadecan-9-yl)oxy)-6-methyltetrahydro-2*H*-pyran-3-ol

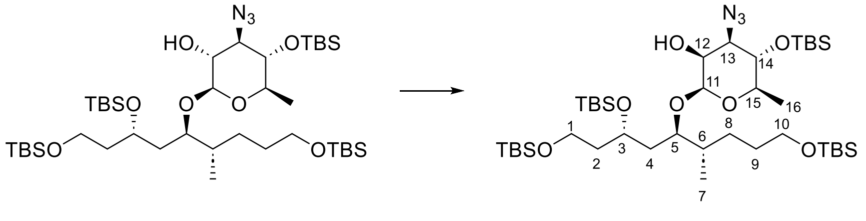

S-37

TBS-**49**
$C_{40}H_{87}N_3O_7Si_4$
834.49 g/mol

DMP (28.8 mg, 67.9 µmol, 3.7 eq.) was added to alcohol **S-37** (15.0 mg, 18.0 µmol, 1.0 eq.) in dry DCM (1.5 mL). After stirring for 2 h at room temperature, another portion of DMP (12.2 mg, 28.8 mg, 1.6 eq.) was added and stirring continued for further 2 h. Afterwards, Et_2O (4 mL) and $NaHCO_3$-solution (aq., sat., 4 mL) were added. The phases were separated and the aqueous phase was extracted with Et_2O (5 x 4 mL). The combined organic layers were dried over Na_2SO_4 and all volatiles were removed

under reduced pressure. The white solid was taken up in dry THF (0.6 mL) and dry MeOH (0.4 mL) and $NaBH_4$ (3.20 mg, 84.6 µmol, 4.7 eq.) was added at 0 °C. After stirring for 2 h at 0 °C, Et_2O (4 mL) and $NaHCO_3$-solution (aq., sat., 4 mL) were added. The phases were separated and the aqueous phase was extracted with Et_2O (5 x 4 mL). The combined organic layers were dried over Na_2SO_4 and all volatiles were removed under reduced pressure. After flash column chromatography (SiO_2, cyclohexane/ethyl acetate 25:1), product TBS-**49** (11.0 mg, 13.2 µmol, 73%, *d.r.* > 20:1) was obtained as colorless oil.

R_f: 0.16 (cyclohexane/ethyl acetate 19:1); **$[\alpha]_D^{20}$** = -7.4° (c = 0.95, $CHCl_3$); **^{1}H-NMR** (700 MHz, CD_2Cl_2, 298 K): δ [ppm] = 4.54 (d, 1H, $^3J_{11,12}$ = 1.1 Hz, H-11), 4.05 (ddd, 1H, $^3J_{12,13}$ = 3.0 Hz, $^3J_{12,OH}$ = 2.6 Hz, $^3J_{11,12}$ = 1.1 Hz, H-12), 3.92 (dddd, 1H, $^3J_{3,4}$ = 8.6 Hz, $^3J_{2,3}$ = 6.5 Hz, $^3J_{2,3}$ = 5.2 Hz, $^3J_{3,4}$ = 3.7 Hz, H-3), 3.81 (ddd, 1H, $^3J_{4,5}$ = 9.1 Hz, $^3J_{5,6}$ = 3.8 Hz, $^3J_{4,5}$ = 2.6 Hz, H-5), 3.67 (t, 2H, $^3J_{1,2}$ = 6.3 Hz, H-1), 3.58 (t, 2H, $^3J_{9,10}$ = 6.5 Hz, H-10), 3.55 (dd, 1H, $^3J_{13,14}$ = 9.5 Hz, $^3J_{14,15}$ = 8.9 Hz, H-14), 3.21 (dq, 1H, $^3J_{14,15}$ = 8.9 Hz, $^3J_{15,16}$ = 6.2 Hz, H-15), 3.08 (ddd, 1H, $^3J_{13,14}$ = 9.5 Hz, $^3J_{12,13}$ = 3.0 Hz, $^4J_{13,OH}$ = 1.0 Hz, H-13), 2.41 (dd, 1H, $^3J_{12,OH}$ = 2.6 Hz, $^4J_{13,OH}$ = 1.0 Hz, H-OH), 1.89 (dddd, 1H, $^3J_{6,8b}$ = 8.9 Hz, $^3J_{6,7}$ = 6.8 Hz, $^3J_{6,8a}$ = 5.4 Hz, $^3J_{5,6}$ = 3.8 Hz, H-6), 1.76 – 1.71 (m, 1H, H-2a), 1.67 – 1.64 (m, 1H, H-2b), 1.62 – 1.56 (m, 2H, H-4a, H-9a), 1.50 – 1.44 (m, 2H, H-4b, H-9b), 1.32 (dddd, 1H, $^2J_{8a,8b}$ = 13.7 Hz, $^3J_{8a,9}$ = 10.8 Hz, $^3J_{8a,9}$ = 5.4 Hz, $^3J_{6,8a}$ = 5.4 Hz, H-8a), 1.27 (d, 3H, $^3J_{15,16}$ = 6.2 Hz, H-16), 1.12 (dddd, 1H, $^2J_{8a,8b}$ = 13.7 Hz, $^3J_{8b,9}$ = 10.4 Hz, $^3J_{6,8b}$ = 8.9 Hz, $^3J_{8b,9}$ = 5.1 Hz, H-8b), 0.91 (s, 9H, TBS), 0.90 (s, 9H, TBS), 0.89 (s, 9H, TBS), 0.89 (s, 9H, TBS), 0.87 (d, 3H, $^3J_{6,7}$ = 6.8 Hz, H-7), 0.20 (s, 3H, TBS), 0.12 (s, 3H, TBS), 0.09 (s, 3H, TBS), 0.07 (s, 3H, TBS), 0.05 (s, 6H, TBS), 0.04 (s, 6H, TBS); **^{13}C-NMR** (176 MHz, CD_2Cl_2, 298 K): δ [ppm] = 100.0 (C-11), 81.0 (C-5), 73.9 (C-15), 72.0 (C-14), 71.8 (C-12), 68.0 (C-3), 67.3 (C-13), 63.6 (C-10), 59.8 (C-1), 41.5 (C-2), 38.2 (C-4), 37.4 (C-6), 31.2 (C-9), 29.3 (C-8), 26.1 (3C, TBS), 26.1 (3C, TBS), 26.1 (3C, TBS), 26.0 (3C, TBS), 18.6 (TBS), 18.6 (C-16), 18.5 (TBS), 18.4 (TBS), 18.4 (TBS), 14.6 (C-7), -3.6 (TBS), -4.0 (TBS), -4.1 (TBS), -4.2 (TBS), -5.2 (TBS), -5.2 (TBS), -5.2 (TBS), -5.2 (TBS); **HRMS (APCI)** *m/z*: $[M + H]^+$ calcd. For $C_{40}H_{87}N_3O_7Si_4H^+$ 834.5694, found 834.5692.

Synthesis of compound S-38

(2*R*,3*S*,4*R*,5*S*,6*R*)-4-Amino-5-((*tert*-butyldimethylsilyl)oxy)-2-(((7*S*,9*R*,10*S*)-7-((*tert*-butyldimethylsilyl)oxy)-2,2,3,3,10,15,15,16,16-nonamethyl-4,14-dioxa-3,15-disilaheptadecan-9-yl)oxy)-6-methyltetrahydro-2*H*-pyran-3-ol

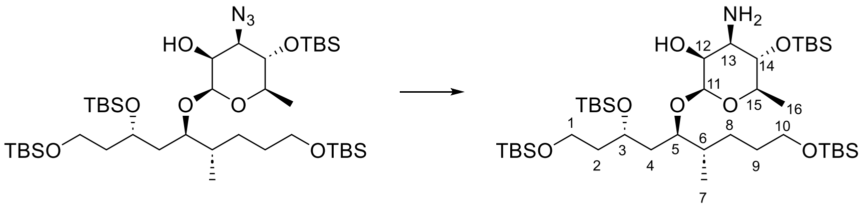

TBS-**49**

S-38
$C_{40}H_{89}NO_7Si_4$
808.49 g/mol

PPh_3 (24.3 mg, 92.6 µmol, 4.8 eq.) was added to azide TBS-**49** (16.2 mg, 19.4 µmol, 1.0 eq.) in THF/H_2O (1:1, 2.0 mL). After stirring for 17 h at room temperature, Et_2O (2 mL) and water (2 mL) were added. The phases were separated and the aqueous phase was extracted with Et_2O (5 x 4 mL). The combined organic layers were dried over Na_2SO_4 and all volatiles were removed under reduced pressure. After flash column chromatography (SiO_2, cyclohexane/ethyl acetate 9:1 → 1:1), product **S-38** was obtained as a colorless oil (13.0 mg, 16.1 µmol, 83%).

R*f*: 0.23 (cyclohexane/ethyl acetate 9:1); **$[\alpha]_D^{20}$** = -7.0° (c = 1.00, $CHCl_3$); **^{1}H-NMR** (700 MHz, CD_2Cl_2, 298 K): δ [ppm] = 4.54 (d, 1H, $^3J_{11,12}$ = 1.1 Hz, H-11), 3.93 – 3.89 (m, 1H, H-3), 3.84 (d, 1H, $^3J_{12,13}$ = 2.9 Hz, H-12), 3.79 (ddd, 1H, $^3J_{4,5}$ = 9.1 Hz, $^3J_{5,6}$ = 3.6 Hz, $^3J_{4,5}$ = 2.6 Hz, H-5), 3.67 (t, 2H, $^3J_{1,2}$ = 6.5 Hz, H-1), 3.58 (t, 2H, $^3J_{9,10}$ = 6.6 Hz, H-10), 3.29 (dd, 1H, $^3J_{13,14}$ = 9.5 Hz, $^3J_{14,15}$ = 8.8 Hz, H-14), 3.17 (dq, 1H, $^3J_{14,15}$ = 8.8 Hz, $^3J_{15,16}$ = 6.2 Hz, H-15), 2.55 (dd, 1H, $^3J_{13,14}$ = 9.5 Hz, $^3J_{12,13}$ = 2.9 Hz, H-13), 1.90 (dddd, 1H, $^3J_{6,8b}$ = 8.8 Hz, $^3J_{6,7}$ = 6.7 Hz, $^3J_{6,8a}$ = 5.5 Hz, $^3J_{5,6}$ = 3.6 Hz, H-6), 1.75 – 1.65 (m, 2H, H-2), 1.60 – 1.55 (m, 2H, H-4a, H-9a), 1.49 – 1.44 (m, 2H, H-4b, H-9b), 1.33 – 1.29 (m, 1H, H-8a), 1.23 (d, 3H, $^3J_{15,16}$ = 6.2 Hz, H-16), 1.12 (dddd, 1H, $^2J_{8a,8b}$ = 13.6 Hz, $^3J_{8b,9}$ = 10.5 Hz, $^3J_{6,8b}$ = 8.8 Hz, $^3J_{8b,9}$ = 5.2 Hz, H-8b), 0.91 (s, 9H, TBS), 0.89 (s, 9H, TBS), 0.89 (s, 9H, TBS), 0.89 (s, 9H, TBS), 0.87 (d, 3H, $^3J_{6,7}$ = 6.7 Hz, H-7), 0.15 (s, 3H, TBS), 0.12 (s, 3H, TBS), 0.08 (s, 3H, TBS), 0.07 (s, 3H, TBS), 0.05 (s, 6H, TBS), 0.04 (s, 6H, TBS); **^{13}C-NMR** (176 MHz, CD_2Cl_2, 298 K): δ [ppm] = 100.9 (C-11), 80.7 (C-5), 76.0 (C-14), 73.8 (C-15), 72.3 (C-12), 68.0 (C-3), 63.7 (C-10), 59.8 (C-1), 58.0 (C-13), 41.6 (C-2), 38.2 (C-4), 37.4 (C-6), 31.2 (C-9), 29.4 (C-8), 26.1 (3C, TBS), 26.1 (3C, TBS), 26.1 (3C, TBS), 26.1 (3C, TBS), 18.8 (C-16), 18.6 (TBS), 18.5 (TBS), 18.5 (TBS), 18.4 (TBS), 14.6 (C-7), -3.1

(TBS), -3.7 (TBS), -3.7 (TBS), -4.1 (TBS), -5.2 (2C, TBS), -5.2 (2C, TBS); **HRMS (ESI+)** *m/z*: $[M + H]^+$ calcd. For $C_{40}H_{89}NO_7Si_4H^+$ 808.5789, found 808.5794.

Synthesis of compound TES-48[3]

(2*R*,3*R*,4*R*,5*S*,6*R*)-4-Azido-2-(((7*S*,9*R*,10*S*)-7-((*tert*-butyldimethylsilyl)oxy)-2,2,3,3,10,15,15,16,16-nonamethyl-4,14-dioxa-3,15-disilaheptadecan-9-yl)oxy)-6-methyl-5-((triethylsilyl)oxy)tetrahydro-2*H*-pyran-3-yl benzoate

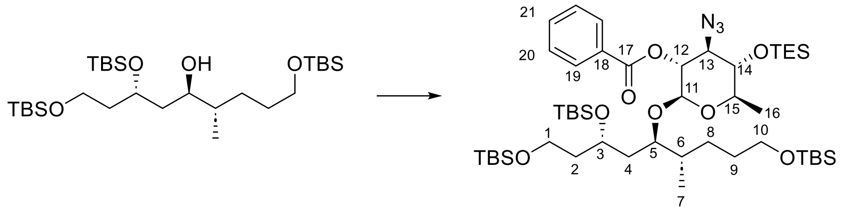

45 TES-**48**
$C_{47}H_{91}N_3O_8Si_4$
938.60 g/mol

Alcohol **45**[3] (35.7 mg, 65.0 µmol, 1.0 eq.) and sugar donor TES-**44**[3] (41.1 mg, 74.5 µmol, 1.15 eq.) were azeotropically dried with toluene and dried for 1 h at high vacuum. After dilution with dry *n*-hexane (2.0 mL), 2-chloro-6-methylpyridine (10.0 µL, 91.7 µmol, 1.41 eq.) and 2-chloro-6-methylpyridinium triflate (9.40 mg, 33.9 µmol, 0.52 eq.) were added. After 10 min, a colorless precipitate was formed. Stirring continued for 90 min at room temperature and then, Et_2O (4 mL) and water (4 mL) were added. The phases were separated and the aqueous phase was extracted with Et_2O (5 x 4 mL). The combined organic layers were dried over Na_2SO_4 and all volatiles were removed under reduced pressure. After flash column chromatography (SiO_2, cyclohexane/ethyl acetate 40:1), product TES-**48** (55.9 mg, 59.6 µmol, 92%, *d.r.* = 18:1) was obtained as a colorless oil.

R_f: 0.64 (cyclohexane/ethyl acetate 9:1); **$[\alpha]_D^{20}$** = +24.0° (c = 1.00, $CHCl_3$); **^{1}H-NMR** (500 MHz, CD_2Cl_2, 298 K): δ [ppm] = 8.06 (dd, 2H, $^3J_{19,20}$ = 8.4 Hz, $^4J_{19,21}$ = 1.3 Hz, H-19), 7.63 – 7.59 (m, 1H, H-21), 7.50 – 7.47 (m, 2H, H-20), 5.05 (dd, 1H, $^3J_{12,13}$ = 10.2 Hz, $^3J_{11,12}$ = 7.9 Hz, H-12), 4.61 (d, 1H, $^3J_{11,12}$ = 7.9 Hz, H-11), 3.64 – 3.60 (m, 2H, H-3, H-5), 3.54 (t, 2H, $^3J_{9,10}$ = 6.6 Hz, H-10), 3.52 (dd, 1H, $^3J_{12,13}$ = 10.2 Hz, $^3J_{13,14}$ = 9.0 Hz, H-13), 3.40 – 3.32 (m, 3H, H-1, H-15), 3.26 (t, 1H, $^3J_{13,14}$ = 9.0 Hz, $^3J_{14,15}$ = 9.0 Hz, H-14), 1.87 – 1.82 (m, 1H, H-6), 1.58 – 1.49 (m, 1H, H-9a), 1.45 – 1.35 (m, 4H, H-2a, H-4, H-9b), 1.33 – 1.20 (m, 2H, H-2b, H-8a), 1.30 (d, 3H, $^3J_{15,16}$ = 6.1 Hz, H-16), 1.09 – 0.98 (m, 1H, H-8b), 1.00 (s, 9H, $^3J_{TES,TES}$ = 7.9 Hz,

TES), 0.87 (s, 9H, TBS), 0.85 (s, 9H, TBS), 0.84 (s, 9H, TBS), 0.82 (d, 3H, $^{3}J_{6,7}$ = 6.8 Hz, H-7), 0.72 – 0.67 (m, 6H, TES), 0.03 (s, 3H, TBS), 0.02 (s, 3H, TBS), 0.02 (s, 3H, TBS), -0.02 (s, 3H, TBS), -0.03 (s, 3H, TBS), -0.09 (s, 3H, TBS); **^{13}C-NMR** (126 MHz, CD_2Cl_2, 298 K): δ [ppm] = 165.2 (C-17), 133.8 (C-21), 130.1 (2C, C-19), 130.0 (C-18), 129.0 (2C, C-20), 101.7 (C-11), 82.1 (C-5), 75.5 (C-14), 73.6 (C-15), 73.5 (C-12), 69.2 (C-13), 67.9 (C-3), 63.6 (C-10), 59.3 (C-1), 41.2 (C-2), 38.8 (C-4), 37.6 (C-6), 31.2 (C-9), 29.0 (C-8), 26.1 (3C, TBS), 26.1 (3C, TBS), 26.1 (3C, TBS), 18.6 (TBS), 18.4 (TBS), 18.2 (TBS), 18.2 (C-16), 14.6 (C-7), 7.0 (3C, TES), 5.6 (3C, TES), -3.8 (TBS), -3.9 (TBS), -5.2 (4C, TBS); **HRMS (ESI+)** *m/z*: [M + K]$^+$ calcd. for $C_{47}H_{91}N_3O_8Si_4K^+$ 976.5515, found 976.5517.

Synthesis of compound S-39[3]

(2*R*,3*R*,4*R*,5*S*,6*R*)-4-Azido-2-(((7*S*,9*R*,10*S*)-7-((*tert*-butyldimethylsilyl)oxy)-2,2,3,3,10,15,15,16,16-nonamethyl-4,14-dioxa-3,15-disilaheptadecan-9-yl)oxy)-6-methyl-5-((triethylsilyl)oxy)tetrahydro-2*H*-pyran-3-ol

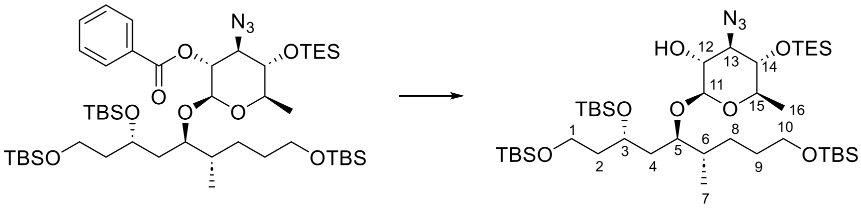

TES-**48**

S-39
$C_{40}H_{87}N_3O_7Si_4$
834.49 g/mol

DIBAL-H (1.0 M in hexanes, 280 µL, 280 µmol, 5.0 eq.) was added at –78 °C to ester TES-**48** (52.9 mg, 56.4 µmol, 1.0 eq.) in dry DCM (4.0 mL). After stirring for 90 min at –78 °C, Rochelle-salt-solution (aq., sat., 4 mL) was added and the biphasic mixture was stirred at room temperature for 2.5 h. The phases were separated and the aqueous phase was extracted with DCM (5 x 4 mL). The combined organic layers were dried over Na_2SO_4 and all volatiles were removed under reduced pressure. After flash column chromatography (SiO_2, cyclohexane/ethyl acetate 35:1), product **S-39** (41.4 mg, 49.6 µmol, 88%) was obtained as a colorless oil.

R*f*: 0.34 (cyclohexane/ethyl acetate 20:1); **$[\alpha]_D^{20}$** = -3.4° (c = 1.16, $CHCl_3$); **^{1}H-NMR** (500 MHz, CD_2Cl_2, 298 K): δ [ppm] = 4.28 (d, 1H, $^{3}J_{11,12}$ = 7.6 Hz, H-11), 4.11 – 4.07 (m, 1H, H-3), 3.85 (dd, 1H, $^{3}J_{12,OH}$ = 2.5 Hz, $^{4}J_{13,OH}$ = 0.8 Hz, H-OH), 3.71 (t, 2H, $^{3}J_{1,2}$ = 6.2 Hz, H-1), 3.71 – 3.68 (m, 1H, H-5), 3.59 (t, 2H, $^{3}J_{9,10}$ = 6.5 Hz, H-10), 3.34 –

3.26 (m, 2H, H-12, H-15), 3.22 (ddd, 1H, $^3J_{12,13}$ = 9.7 Hz, $^3J_{13,14}$ = 9.0 Hz, $^4J_{13,OH}$ = 0.8 Hz, H-13), 3.06 (t, 1H, $^3J_{13,14}$ = 9.0 Hz, $^3J_{14,15}$ = 9.0 Hz, H-14), 1.95 – 1.90 (m, 1H, H-6), 1.81 (dtd, 1H, $^2J_{2a,2b}$ = 13.9 Hz, $^3J_{1,2a}$ = 6.2 Hz,$^3J_{2a,3}$ = 6.0 Hz, H-2a), 1.76 – 1.71 (m, 1H, H-4a), 1.69 (dtd, 1H, $^2J_{2a,2b}$ = 13.9 Hz, $^3J_{1,2b}$ = 6.2 Hz,$^3J_{2b,3}$ = 6.0 Hz, H-2b), 1.62 – 1.55 (m, 1H, H-9a), 1.50 – 1.42 (m, 2H, H-4b, H-9b), 1.33 – 1.26 (m, 1H, H-8a), 1.23 (d, 3H, $^3J_{15,16}$ = 6.1 Hz, H-16), 1.14 – 1.07 (m, 1H, H-8b), 0.98 (t, 9H, $^3J_{TES,TES}$ = 7.9 Hz, TES), 0.91 (s, 9H, TBS), 0.91 (s, 9H, TBS), 0.89 (s, 9H, TBS), 0.86 (d, 3H, $^3J_{6,7}$ = 6.8 Hz, H-7), 0.72 – 0.63 (m, 6H, TES), 0.11 (s, 3H, TBS), 0.10 (s, 3H, TBS), 0.08 (s, 3H, TBS), 0.08 (s, 3H, TBS), 0.04 (s, 3H, TBS), 0.04 (s, 3H, TBS); **^{13}C-NMR** (126 MHz, CD_2Cl_2, 298 K): δ [ppm] = 105.1 (C-11), 83.9 (C-5), 75.3 (C-14), 74.5 (C-12), 73.7 (C-15), 70.1 (C-13), 68.7 (C-3), 63.6 (C-10), 60.3 (C-1), 40.5 (C-2), 37.6 (C-6), 36.9 (C-4), 31.3 (C-9), 29.6 (C-8), 26.2 (3C, TBS), 26.2 (6C, TBS), 18.7 (TBS), 18.6 (TBS), 18.5 (TBS), 18.4 (C-16), 14.0 (C-7), 7.1 (3C, TES), 5.7 (3C, TES) -4.1 (TBS), -4.1 (TBS), -5.1 (TBS), -5.2 (2C, TBS), -5.3 (TBS); **HRMS (ESI+)** *m/z*: $[M + K]^+$ calcd. for $C_{40}H_{87}N_3O_7Si_4K^+$ 872.5253, found 872.5258.

Synthesis of compound TES-49[3]

(2*R*,3*S*,4*R*,5*S*,6*R*)-4-Azido-2-(((7*S*,9*R*,10*S*)-7-((*tert*-butyldimethylsilyl)oxy)-2,2,3,3,10,15,15,16,16-nonamethyl-4,14-dioxa-3,15-disilaheptadecan-9-yl)oxy)-6-methyl-5-((triethylsilyl)oxy)tetrahydro-2*H*-pyran-3-ol

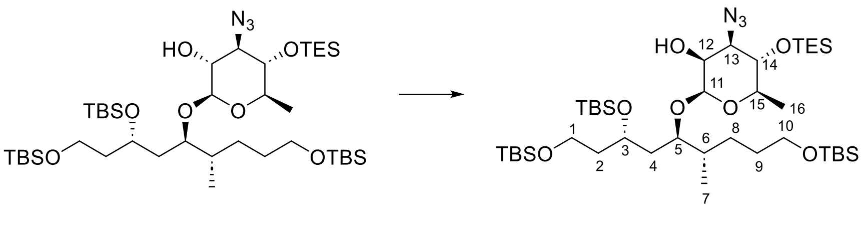

S-39 TES-**49**

$C_{40}H_{87}N_3O_7Si_4$

834.49 g/mol

DMP (68.9 mg, 162 µmol, 3.9 eq.) was added to alcohol **S-39** (35.1 mg, 42.1 µmol, 1.0 eq.) in dry DCM (1.2 mL). After stirring for 4 h at room temperature, Et_2O (4 mL), H_2O (2 mL) and $NaHCO_3$-solution (aq., sat., 2 mL) were added. The phases were separated and the aqueous phase was extracted with Et_2O (5 x 4 mL). The combined organic layers were dried over Na_2SO_4 and all volatiles were removed under reduced pressure. The white solid was taken up in dry THF (0.6 mL) and dry MeOH (0.4 mL) and $NaBH_4$ (6.1 0 mg, 161 µmol, 3.9 eq.) was added at 0 °C. After stirring for 2 h at 0 °C, Et_2O (4 mL) and H_2O (4 mL) were added. The phases were separated and the aqueous phase was extracted with Et_2O (5 x 4 mL). The combined organic layers were dried over Na_2SO_4 and all volatiles were removed under reduced pressure. After flash column chromatography (SiO_2, cyclohexane/ethyl acetate 30:1), product TES-**49** (30.1 mg, 36.1 µmol, 86%, *d.r.* = 18:1) was obtained as a colorless oil.

R*f*: 0.27 (cyclohexane/ethyl acetate 20:1); **$[\alpha]_D^{20}$** = -6.6° (c = 1.22, $CHCl_3$); **^{1}H-NMR** (500 MHz, CD_2Cl_2, 298 K): δ [ppm] = 4.54 (d, 1H, $^3J_{11,12}$ = 1.0 Hz, H-11), 4.05 (ddd, 1H, $^3J_{12,13}$ = 3.0 Hz, $^3J_{12,OH}$ = 2.5 Hz, $^3J_{11,12}$ = 1.0 Hz, H-12), 3.95 – 3.90 (m, 1H, H-3), 3.81 (ddd, 1H, $^3J_{4,5}$ = 9.1 Hz, $^3J_{5,6}$ = 3.1 Hz, $^3J_{4,5}$ = 3.1 Hz, H-5), 3.67 (t, 2H, $^3J_{1,2}$ = 6.3 Hz, H-1), 3.59 (t, 2H, $^3J_{9,10}$ = 6.5 Hz, H-10), 3.57 (dd, 1H, $^3J_{13,14}$ = 9.4 Hz, $^3J_{14,15}$ = 8.9 Hz, H-14), 3.21 (dq, 1H, $^3J_{14,15}$ = 8.9 Hz, $^3J_{15,16}$ = 6.2 Hz, H-15), 3.08 (ddd, 1H, $^3J_{13,14}$ = 9.4 Hz, $^3J_{12,13}$ = 3.0 Hz, $^4J_{13,OH}$ = 1.0 Hz, H-13), 2.39 (dd, 1H, $^3J_{12,OH}$ = 2.5 Hz, $^4J_{13,OH}$ = 1.0 Hz, H-OH), 1.92 – 1.87 (m, 1H, H-6), 1.78 – 1.71 (m, 1H, H-2a), 1.69 – 1.63 (m, 1H, H-2b), 1.63 – 1.55 (m, 2H, H-4a, H-9a), 1.51 – 1.44 (m, 2H, H-4b, H-9b), 1.36 – 1.30 (m, 1H, H-8a), 1.28 (d, 3H, $^3J_{15,16}$ = 6.2 Hz, H-16), 1.17 – 1.09 (m, 1H, H-8b), 0.99 (t, 9H, $^3J_{TES,TES}$ = 7.9 Hz, TES), 0.90 (s, 9H, TBS), 0.90 (s, 9H,

TBS), 0.89 (s, 9H, TBS), 0.88 (d, 3H, $^{3}J_{6,7}$ = 6.9 Hz, H-7), 0.72 – 0.67 (m, 6H, TES), 0.09 (s, 3H, TBS), 0.07 (s, 3H, TBS), 0.06 (s, 6H, TBS), 0.04 (s, 6H, TBS); **^{13}C-NMR** (126 MHz, CD_2Cl_2, 298 K): *δ* [ppm] = 100.1 (C-11), 81.0 (C-5), 73.9 (C-15), 72.2 (C-14), 71.7 (C-12), 68.1 (C-3), 67.3 (C-13), 63.7 (C-10), 59.9 (C-1), 41.6 (C-2), 38.3 (C-4), 37.5 (C-6), 31.3 (C-9), 29.4 (C-8), 26.2 (6C, TBS), 26.1 (3C, TBS), 18.6 (TBS), 18.5 (TBS), 18.4 (TBS), 18.3 (C-16), 14.6 (C-7), 7.1 (3C, TES), 5.7 (3C, TES), -3.6 (TBS), -4.0 (TBS), -5.1 (2C, TBS), -5.2 (TBS), -5.2 (TBS); **HRMS (APCI)** *m/z*: $[M + H]^+$ calcd. for $C_{40}H_{87}N_3O_7Si_4H^+$ 834.5694, found 834.5693.

Synthesis of compound S-40[3]

(3*S*,5*R*,6*S*)-5-(((2*R*,3*S*,4*S*,5*S*,6*R*)-4-Azido-3,5-dihydroxy-6-methyltetrahydro-2*H*-pyran-2-yl)oxy)-6-methylnonane-1,3,9-triol

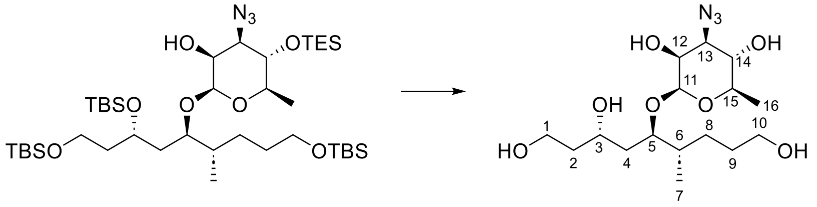

TES-**49**

S-40
$C_{16}H_{31}N_3O_7$
377.44 g/mol

HCl (1.0 M in H_2O, 90.0 µL, 90.0 µmol, 4.8 eq.) was added to alcohol TES-**49** (15.5 mg, 18.6 µmol, 1.0 eq.) in MeOH (1.0 mL). After stirring for 18 h at room temperature, solid $NaHCO_3$ (10.2 mg, 121 µmol, 6.5 eq.) was added and the reaction mixture was concentrated *in vacuo*. After flash column chromatography (SiO_2, DCM/MeOH 10:1 → 5:1), product **S-40** (5.90 mg, 15.6 µmol, 84%) was obtained as a colorless solid.

R*f*: 0.13 (DCM/MeOH 9:1); **$[\alpha]_D^{20}$** = -1.7° (c = 1.18, MeOH); **^{1}H-NMR** (500 MHz, MeOD, 298 K): *δ* [ppm] = 4.59 (d, 1H, $^{3}J_{11,12}$ = 1.0 Hz, H-11), 3.98 (dd, 1H, $^{3}J_{12,13}$ = 3.0 Hz, $^{3}J_{11,12}$ = 1.0 Hz, H-12), 3.88 – 3.84 (m, 2H, H-3, H-5), 3.74 – 3.66 (m, 2H, H-1), 3.55 (t, 2H, $^{3}J_{9,10}$ = 6.5 Hz, H-10), 3.52 (dd, 1H, $^{3}J_{13,14}$ = 10.1 Hz, $^{3}J_{14,15}$ = 9.2 Hz, H-14), 3.28 (dq, 1H, $^{3}J_{14,15}$ = 9.2 Hz, $^{3}J_{15,16}$ = 6.1 Hz, H-15), 3.20 (dd, 1H, $^{3}J_{13,14}$ = 10.1 Hz, $^{3}J_{12,13}$ = 3.0 Hz, H-13), 1.99 – 1.92 (m, 1H, H-6), 1.69 – 1.64 (m, 2H, H-2), 1.64 – 1.58 (m, 1H, H-9a), 1.57 – 1.53 (m, 1H, H-4a), 1.53 – 1.49 (m, 1H, H-9b), 1.45 – 1.38 (m, 2H, H-4b, H-8a), 1.31 (d, 3H, $^{3}J_{15,16}$ = 6.1 Hz, H-16), 1.22 – 1.15 (m, 1H, H-8b), 0.92 (d, 3H, $^{3}J_{6,7}$ = 6.8 Hz, H-7); **^{13}C-NMR** (126 MHz, MeOD, 298 K): *δ* [ppm] = 102.8 (C-11), 81.9 (C-5), 74.5 (C-15), 72.0 (C-12), 71.8 (C-14), 66.9 (C-13),

66.4 (C-3), 63.2 (C-10), 60.1 (C-1), 41.8 (C-2), 38.8 (C-4), 38.3 (C-6), 31.5 (C-9), 30.5 (C-8), 18.0 (C-16), 14.8 (C-7); **(ESI+)** *m/z*: [M + H]$^+$ calcd. for $C_{16}H_{31}N_3O_7H^+$ 378.2235, found 378.2236.

Synthesis of compound 10[3]

(3*S*,5*R*,6*S*)-5-(((2*R*,3*S*,4*S*,5*S*,6*R*)-4-Amino-3,5-dihydroxy-6-methyltetrahydro-2*H*-pyran-2-yl)oxy)-6-methylnonane-1,3,9-triol

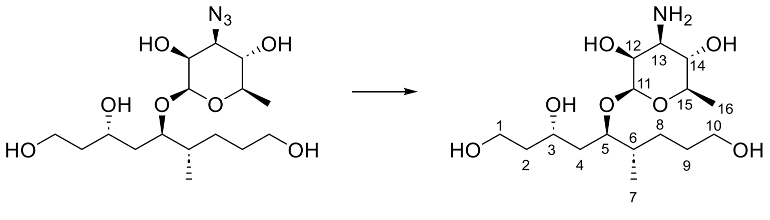

S-40 **10**

$C_{16}H_{33}NO_7$

351.44 g/mol

PPh_3 (12.5 mg, 47.7 µmol, 2.4 eq.) was added to alcohol **S-40** (7.50 mg, 19.9 µmol, 1.0 eq.) in THF/MeOH/H_2O (1:1:1, 1.20 mL). After stirring for 21 h at room temperature, the reaction mixture was concentrated *in vacuo*. After flash column chromatography (SiO_2, DCM/MeOH 2:1 → 1:1), product **10** (3.70 mg, 10.5 µmol, 53%) was obtained as a colorless solid.

R*f*: 0.12 (DCM/MeOH 7:3); **[α]$_D^{20}$** = -2.6° (c = 1.16, $CHCl_3$); **^{1}H-NMR** (500 MHz, MeOD, 298 K): *δ* [ppm] = 4.61 (d, 1H, $^3J_{11,12}$ = 0.9 Hz, H-11), 3.90 – 3.86 (m, 2H, H-3, H-5), 3.84 (dd, 1H, $^3J_{12,13}$ = 3.0 Hz, $^3J_{11,12}$ = 0.9 Hz, H-12), 3.70 (2 x t, 2H, $^3J_{1,2}$ = 6.4 Hz, H-1), 3.55 (t, 2H, $^3J_{9,10}$ = 6.7 Hz, H-10), 3.23 (dq, 1H, $^3J_{14,15}$ = 9.2 Hz, $^3J_{15,16}$ = 6.2 Hz, H-15), 3.11 (dd, 1H, $^3J_{13,14}$ = 9.8 Hz, $^3J_{14,15}$ = 9.2 Hz, H-14), 2.52 (dd, 1H, $^3J_{13,14}$ = 9.8 Hz, $^3J_{12,13}$ = 3.0 Hz, H-13), 2.01 – 1.96 (m, 1H, H-6), 1.67 (q, 2H, $^3J_{1,2}$ = 6.4 Hz, $^3J_{2,3}$ = 6.4 Hz, H-2), 1.64 – 1.59 (m, 1H, H-9a), 1.57 – 1.49 (m, 2H, H-4a, H-9b), 1.44 – 1.37 (m, 2H, H-4b, H-8a), 1.29 (d, 3H, $^3J_{15,16}$ = 6.2 Hz, H-16), 1.23 – 1.15 (m, 1H, H-8b), 0.92 (d, 3H, $^3J_{6,7}$ = 6.8 Hz, H-7); **^{13}C-NMR** (126 MHz, MeOD, 298 K): *δ* [ppm] = 103.5 (C-11), 81.6 (C-5), 74.9 (C-14), 74.7 (C-15), 72.0 (C-12), 66.6 (C-3), 63.2 (C-10), 60.2 (C-1), 57.6 (C-13), 41.8 (C-2), 38.8 (C-4), 38.3 (C-6), 31.5 (C-9), 30.5 (C-8), 18.1 (C-16), 14.8 (C-7); **HRMS (ESI+)** *m/z*: [M + H]$^+$ calcd. for $C_{16}H_{33}NO_7H^+$ 352.2330, found 352.2334.

3. Biological Evaluation

Strains.

**Bacterial strains**		
Staphylococcus aureus	HG001	Herbert *et al.*, 2010[6]
Staphylococcus aureus	USA 300 (CA-MRSA)	NARSA; Seybold *et al.*, 2006[7]
Staphylococcus aureus	Mu50 (VISA)	ATCC; Kuroda *et al.*, 2001[8]
Staphylococcus aureus	SG511	Dietrich *et al.*, 2021[9]
Enterococcus faecalis	JH 2-2	Jacob and Hobbs, 1974[10]
Enterococcus faecalis	V583 (VRE)	ATCC, Sahm *et al.*, 1989[11]
Bacillus subtilis	168	Kunst *et al.*, 1997[12]
Bacillus subtilis	P_{ypuA}-*lacZ*	Harms *et al.*, 2018[13]
Bacillus subtilis	P_{liaI}-*lux*	Radeck *et al.*, 2016[14]

Susceptibility testing. Minimal inhibitory concentrations (MICs) of natural compound **1** and synthesized fragments against the test panel strains were determined by standard broth microdilution according to the CLSI guidelines.[15] Assays were performed in U-bottom polypropylene microtiter plates (Greiner Bio-One, Kremsmuenster, Austria) using cation-adjusted Mueller-Hinton broth (MHB, Oxoid, Thermo Fisher Diagnostics, Wesel, Germany), except for enterococci, for which Brain Heart Infusion broth (BHI, Oxoid, Thermo Fisher Diagnostics, Wesel, Germany) was used. MICs were defined as the lowest concentrations at which no visible growth was observed.

DiSC$_3$(5) release assay. Membrane depolarization was assessed using 3,3-dipropylthiadicarbocyanine iodide (DiSC$_3$(5), Biomol, Hamburg, Germany). *S. aureus* was grown in liquid culture at 37°C and 120 rpm to an OD_{600} of 0.3. DiSC$_3$(5) was added to a final concentration of 1 µM, and the DMSO concentration (Merck KGaA, Darmstadt, Germany) was adjusted to 1 % v/v. 200 µL aliquots were transferred to flat-bottom black polystyrene 96-well plates (Greiner Bio-One, Kremsmuenster, Austria), and fluorescence was recorded using a Tecan Infinite F Plex plate reader (Tecan Group, Männedorf, Switzerland) equipped with a monochromator at Λ_{ex} = 580 nm and Λ_{em} = 635 nm at 1 min intervals until the signal remained stable for 5 min, indicating maximal dye uptake and self-quenching. Compounds were added and fluorescence was monitored for 1 h. Valinomycin (Merck KGaA, Darmstadt, Germany) in the presence of 300 mM potassium chloride (Carl Roth GmbH + Co. KG, Karlsruhe, Germany) served as a positive control.

MinD assay. *B. subtilis* 168 *amyE*::*spc* P_{xyl}-*gfp-minD*[16] was grown in Luria-Bertani (LB) broth supplemented with 0.1% xylose and 0.2% glucose (w/v, Merck KGaA, Darmstadt, Germany) to prevent sporulation. Cultures were incubated at 30 °C and 120 rpm and subcultured to an OD_{600} of 0.3. Imaging was performed 15 minutes after addition of **1** (2x MIC) or fragments **7** and **8** (256 µg mL^{-1}). Nisin (5x MIC) was used as a positive control. Samples were immobilized on microscope slides covered with 1% agarose (w/v, Carl Roth GmbH + Co. KG, Karlsruhe, Germany). Widefield fluorescence microscopy was performed using an AxioObserver Z1

system (Carl Zeiss AG, Oberkochen, Germany) equipped with a Colibri 5/7 LED light source, an Axiocam 820 mono camera, a Plan-Apochromat 100x/1.40 Oil Ph 3 M27 objective, and an Apotome 3 module for optical sectioning (Carl Zeiss AG, Oberkochen, Germany). Images were acquired with an exposure time of 350 ms using excitation and emission filters of 450-488 nm and 501–527 nm, respectively. Data acquisition was managed with Zen Blue 2.0 software (Carl Zeiss AG, Oberkochen, Germany) and analyzed using ImageJ2 v2.14.049 (NIH).[17]

***B. subtilis* reporter gene assays.** *B. subtilis* reporter strains carrying promotor fusions specific to cell wall stress (P_{ypuA}-*lacZ*[13]) or stress induced by lipid II-cycle interfering antibiotics (P_{liaI}-*lux*[14]) were analyzed in a microtiter-based format. Briefly, reporter strains were grown in MHB supplemented with 5 µg mL^{-1} chloramphenicol (Carl Roth GmbH + Co. KG, Karlsruhe, Germany) at 30 °C to an OD_{600} of 0.5. Cells were then transferred to 96-well black wall chimney plates containing serially diluted antibiotics and 4-methylumbelliferyl-β-D-galactopyranoside (50 µM, final concentration, Merck KGaA, Darmstadt, Germany) to monitor β-galactosidase expression, or 96-well white wall chimney plates containing serially diluted antibiotics to measure luminescence from expression of the *Photorhabdus luminescence* luciferase gene cluster. Fluorescence and luminescence were monitored over time at 30 °C using a Tecan Spark 10M microplate reader (Tecan Group, Männedorf, Switzerland).

Bacterial Cell Wall Integrity Assay. Bacterial cell wall integrity was assessed as described before.[18] Briefly, *B. subtilis* 168 was grown in MHB at 30 °C to an OD_{600} of 0.3. Cells were treated with compound **1**, fragments **7**-**9**, or vancomycin at 1× MIC and incubated at 30 °C. After 30 min of treatment, 200 µL culture aliquots were fixed in 1 mL acetic acid/ methanol (1:3, v/v) and immobilized on a thin film of 1% agarose (w/v, Carl Roth GmbH + Co. KG, Karlsruhe, Germany) containing 0.9% NaCl (w/v, Carl Roth GmbH + Co. KG, Karlsruhe, Germany) on microscope slides. Phase-contrast images were acquired using an Axio Observer Z1 microscope (Zeiss) equipped with an HXP 120-V light source and an Axio Cam MR3 camera. Images were recorded with ZEN 2 software (Zeiss) and analyzed and postprocessed using ImageJ v1.45s (NIH).[17]

4. Computational Methods

The conformational space of vancoresmycin was analyzed using CREST version 3.0.2[19], the starting geometry was previously optimized on the GFN2-xTB level of theory.[20] Implicit solvation was applied using the ALPB model for methanol with default settings in CREST.[21] All resulting conformers were further reoptimized using CENSO version 3.0.2.[22] For the screening and prescreening, the default settings were applied. The optimization was performed using the default settings with the r^{2}SCAN-3c functional.[23–25] Implicit solvation was again for by using the CPCM model for methanol.[26] The single point energies, free energies and their respective Boltzmann weights at 298.15 K are presented in Table 4.

Table 4: Results after the optimization step of the CENSO calculation at the r2SCAN-3c level of theory. From initially 34 conformers of the CREST run only 6 conformers remained.

CONF	E (DFT) [Eh]	ΔGsolv [kcal/mol]	GmRRHO [Eh]	Gtot [kcal/mol]	ΔGtot [kcal/mol]	Boltzmann weight at 298.15 K [%]
CONF1	4469.751790	0.000000	1.750467	4468.001322	2.17	1.54
CONF3	4469.752064	0.000000	1.748639	4468.003425	0.85	14.32
CONF5	4469.755021	0.000000	1.750237	4468.004784	0.00	60.44
CONF12	4469.753041	0.000000	1.749200	4468.003842	0.59	22.27
CONF23	4469.752423	0.000000	1.752291	4468.000133	2.92	0.44
CONF25	4469.752439	0.000000	1.751545	-468.000894	2.44	0.98

The remaining structures were analyzed for potential hydrogen-bonding interactions based on interatomic distances using a cutoff radius of 3.0 Å. All conformers with a Boltzmann population greater than 1% exhibited a characteristic hydrogen-bonding network. In particular, a key hydrogen-bonding interaction between the tetramic acid moiety and the opposite half of the molecule appears to lock the molecule into a defined conformation. An overview of the conserved hydrogen-bonding network is presented in Figure 6A, while the resulting conformers are shown in Figure 6B.

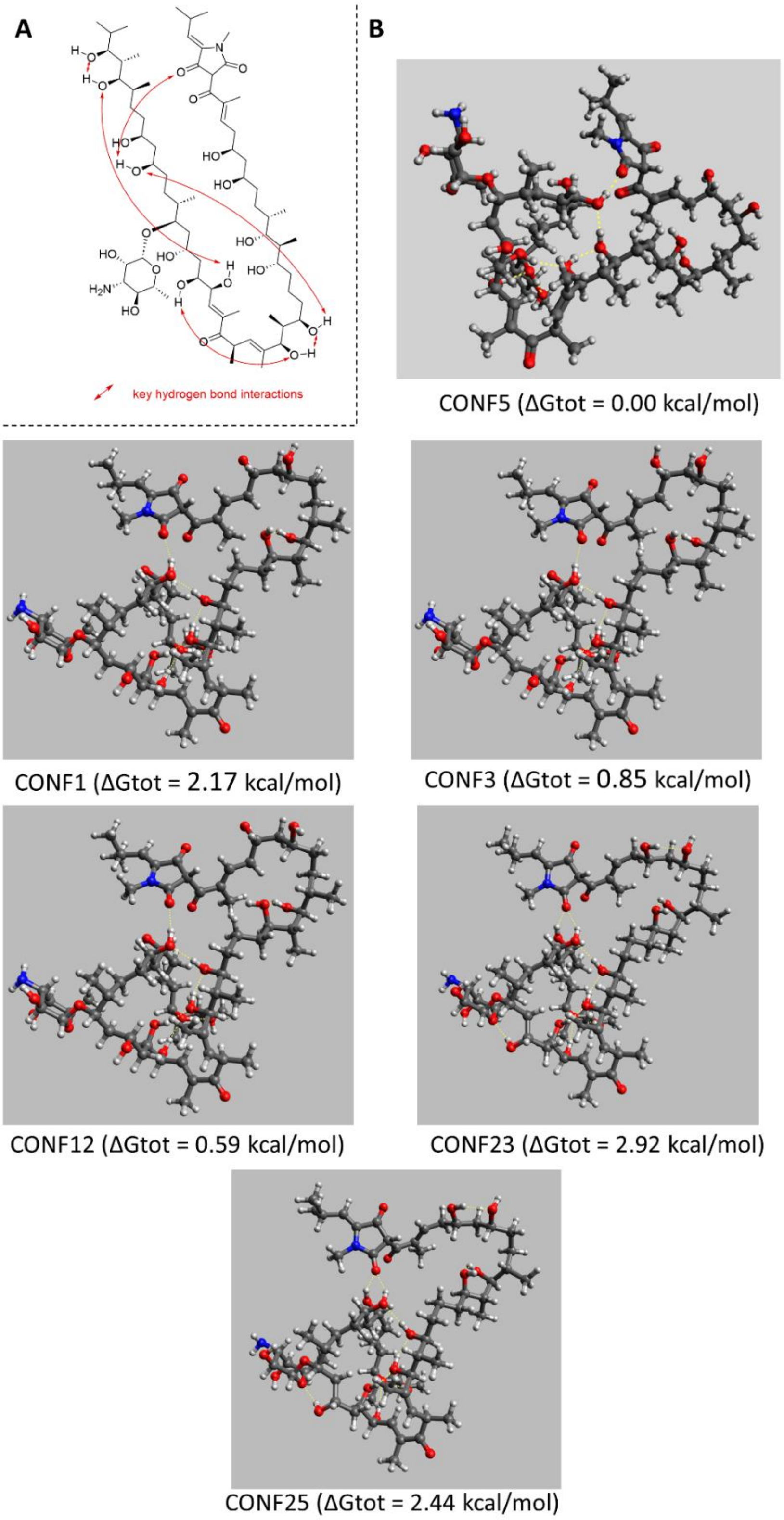

Figure 6: **A** Overview over the hydrogen bond network conserved in all confomers with a Boltzmann weight over 1%. **B** Optimized geometries obtained from the CENSO calculations of vancoresmycin (**1**) after the optimization step on the r^{2}scan-3c level of theory. H-bond interactions (yellow dashed lines) are highlighted based on the distance (cutoff radius 3.0 Å).[23–25]

5. NMR spectra

Tetramic Acid 6

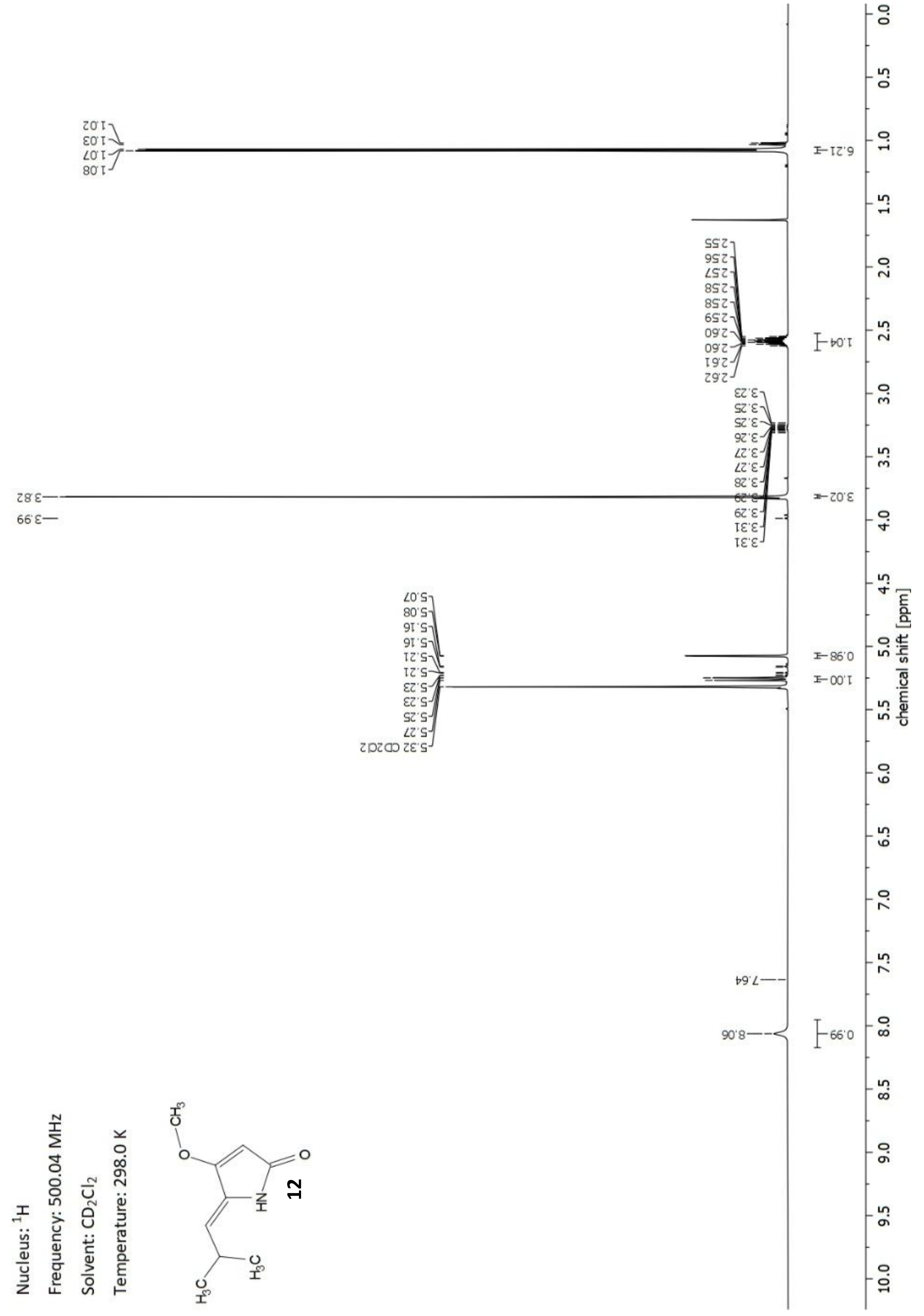

Nucleus: ^{13}C

Frequency: 125.75 MHz

Solvent: CD_2Cl_2

Temperature: 298.0 K

12

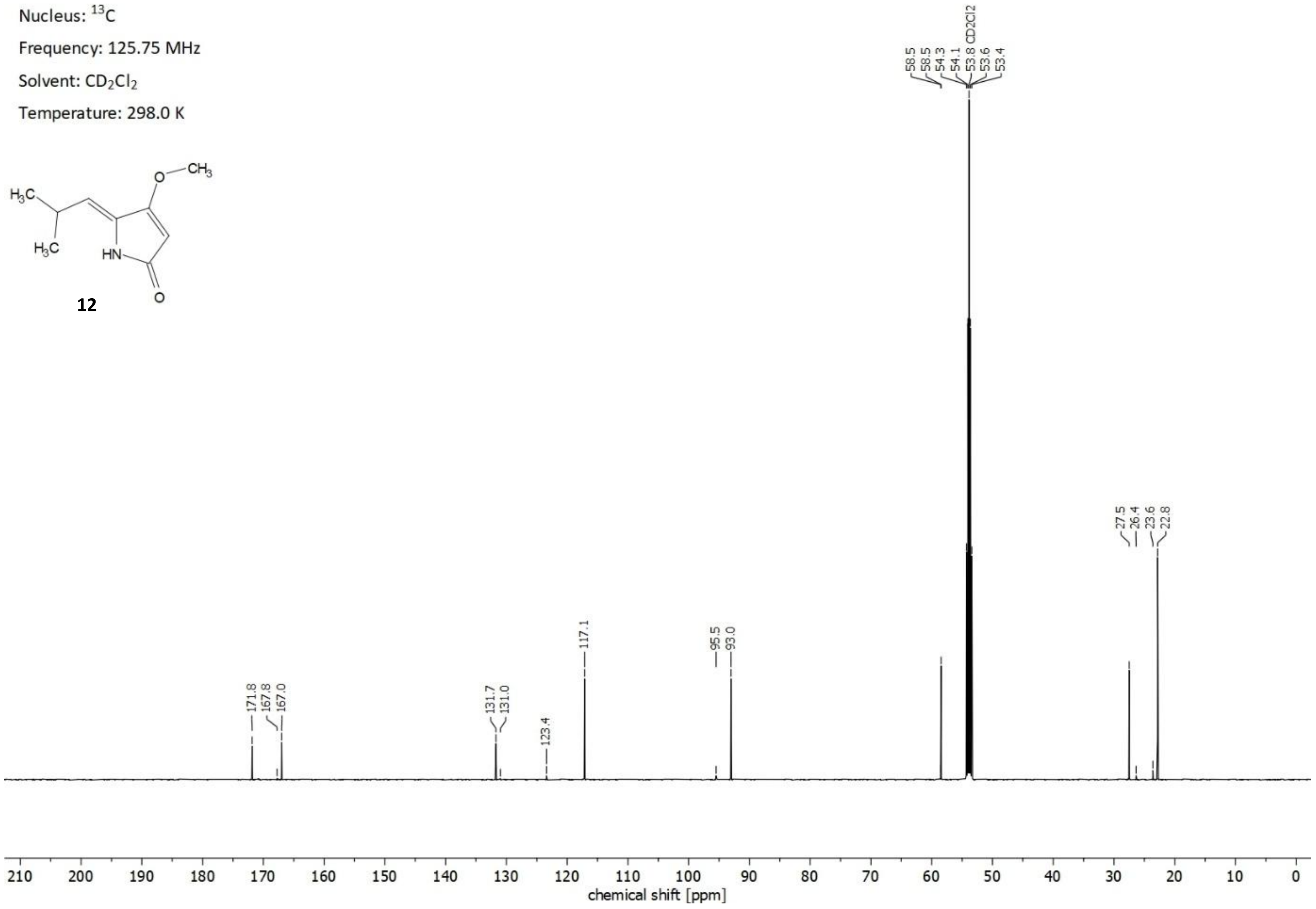

Nucleus: ^{1}H

Frequency: 500.04 MHz

Solvent: CD_2Cl_2

Temperature: 298.0 K

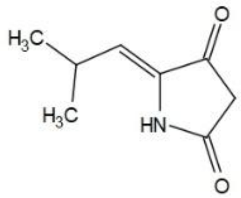

13

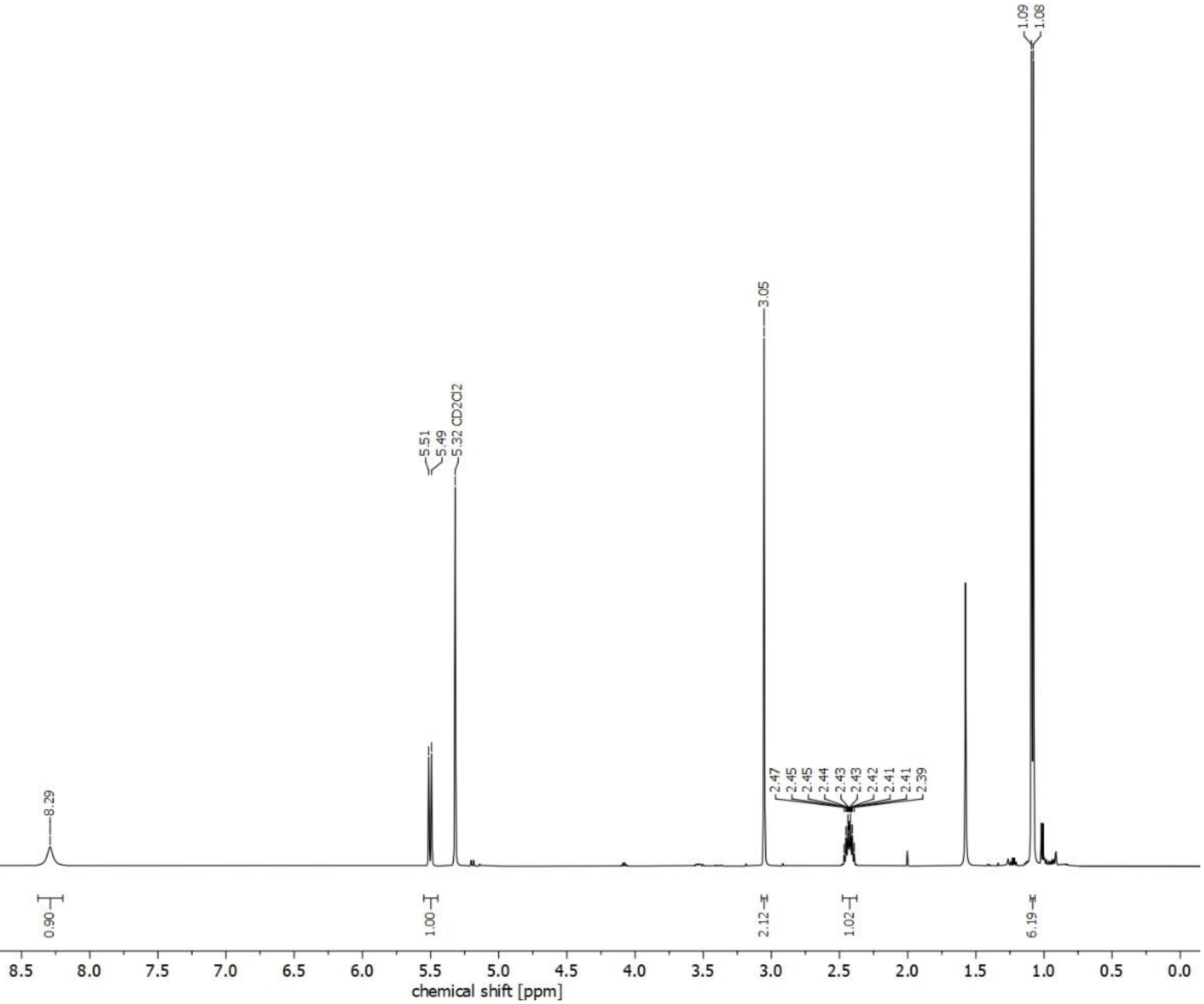

Nucleus: ^{13}C

Frequency: 125.75 MHz

Solvent: CD_2Cl_2

Temperature: 298.0 K

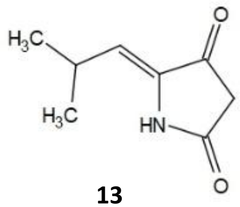

13

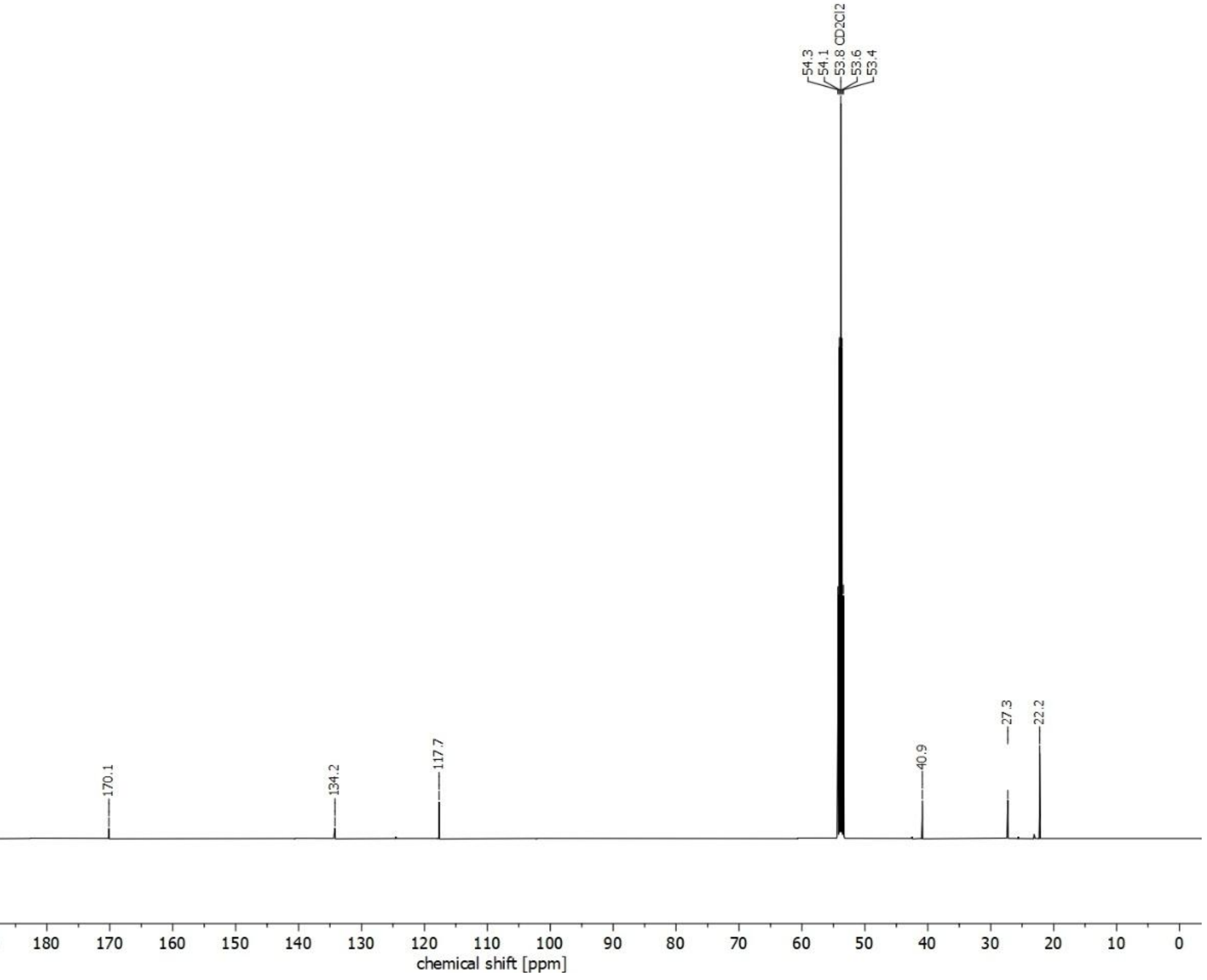

Nucleus: ^{1}H

Frequency: 500.04 MHz

Solvent: CD_2Cl_2

Temperature: 298.0 K

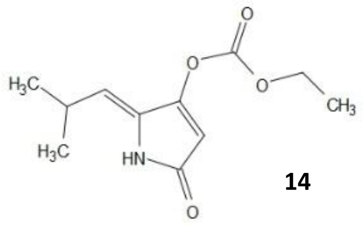

14

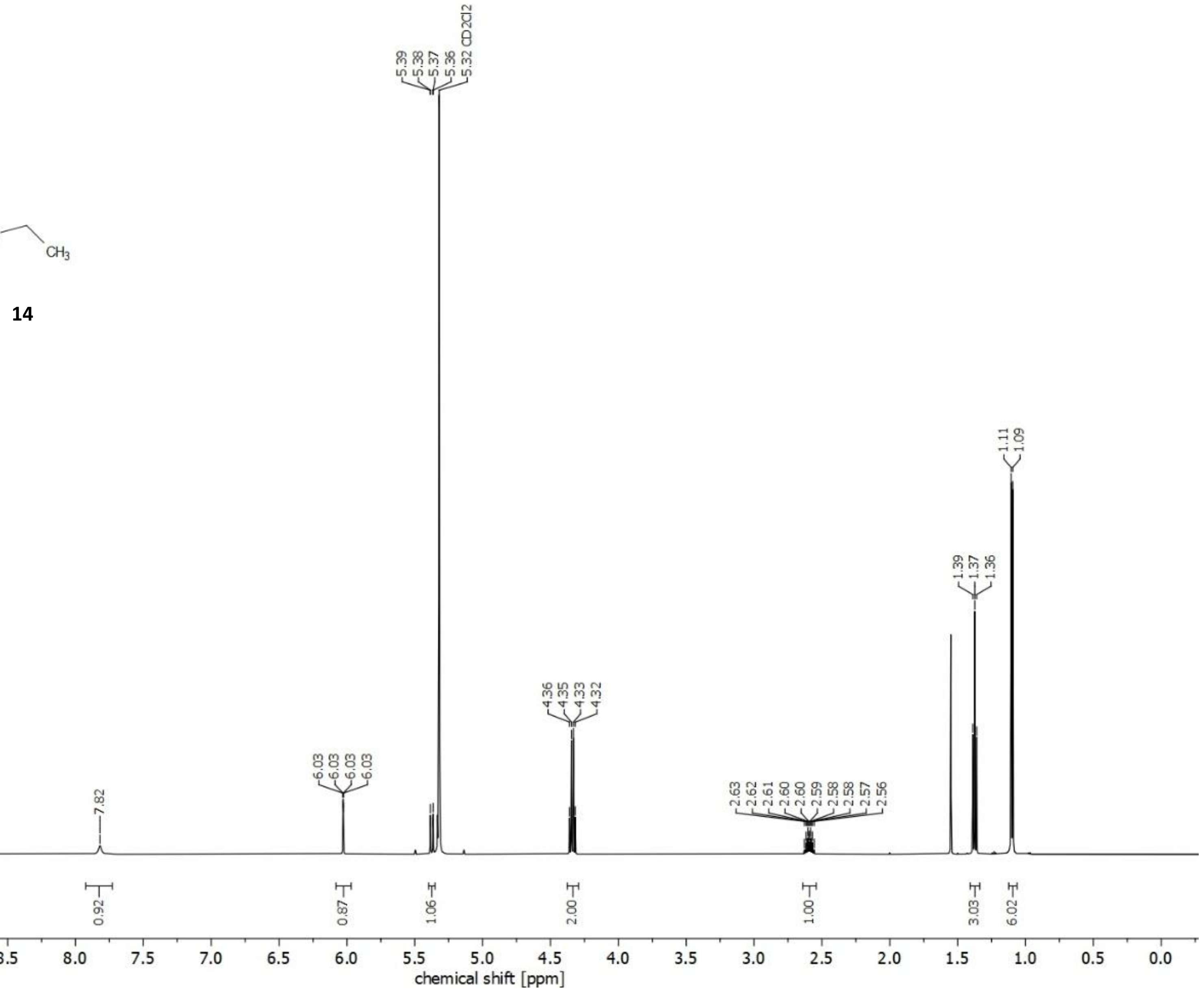

Nucleus: ^{13}C

Frequency: 125.75 MHz

Solvent: CD_2Cl_2

Temperature: 298.0 K

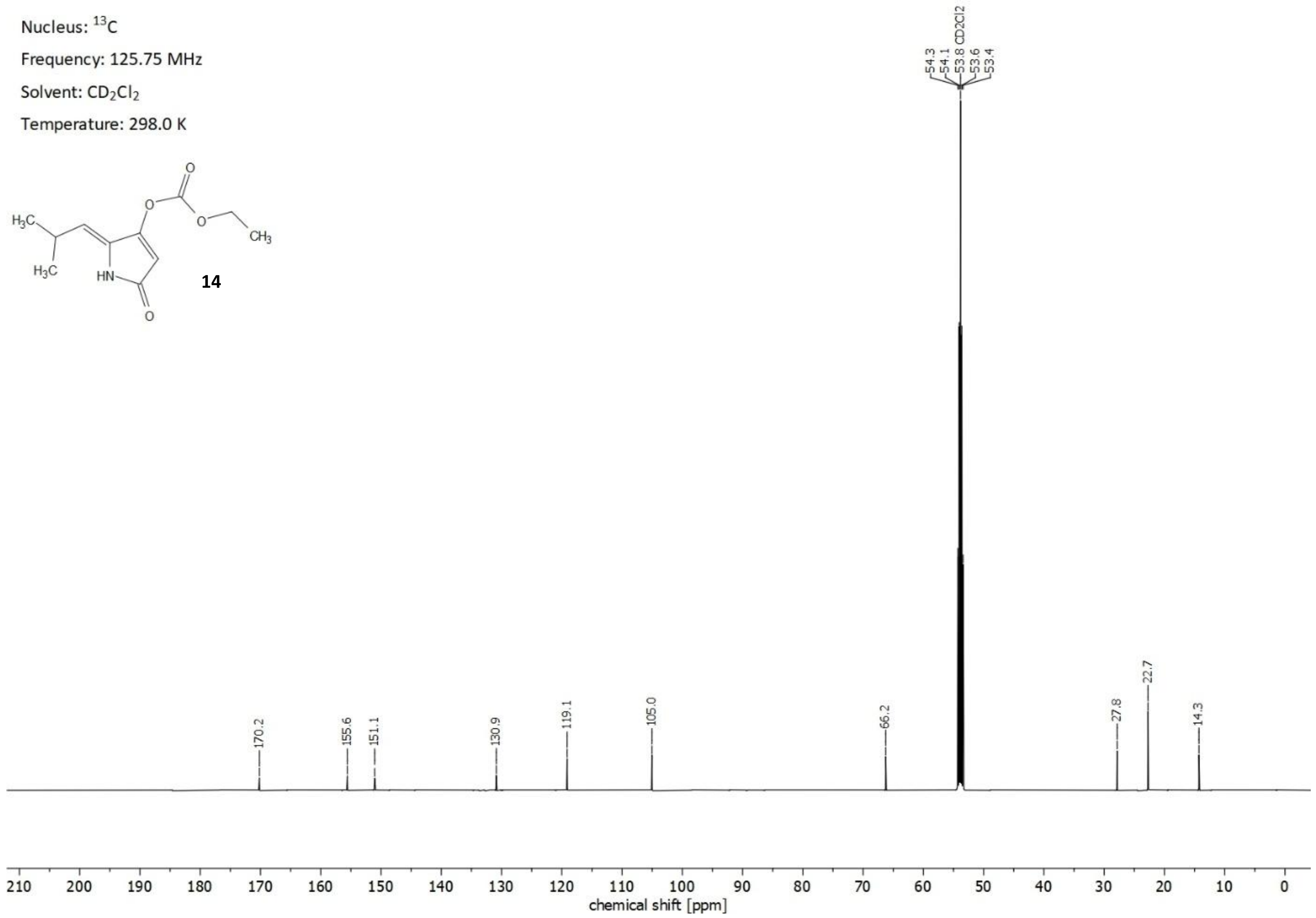

14

Nucleus: ^{1}H

Frequency: 500.04 MHz

Solvent: CD_2Cl_2

Temperature: 298.0 K

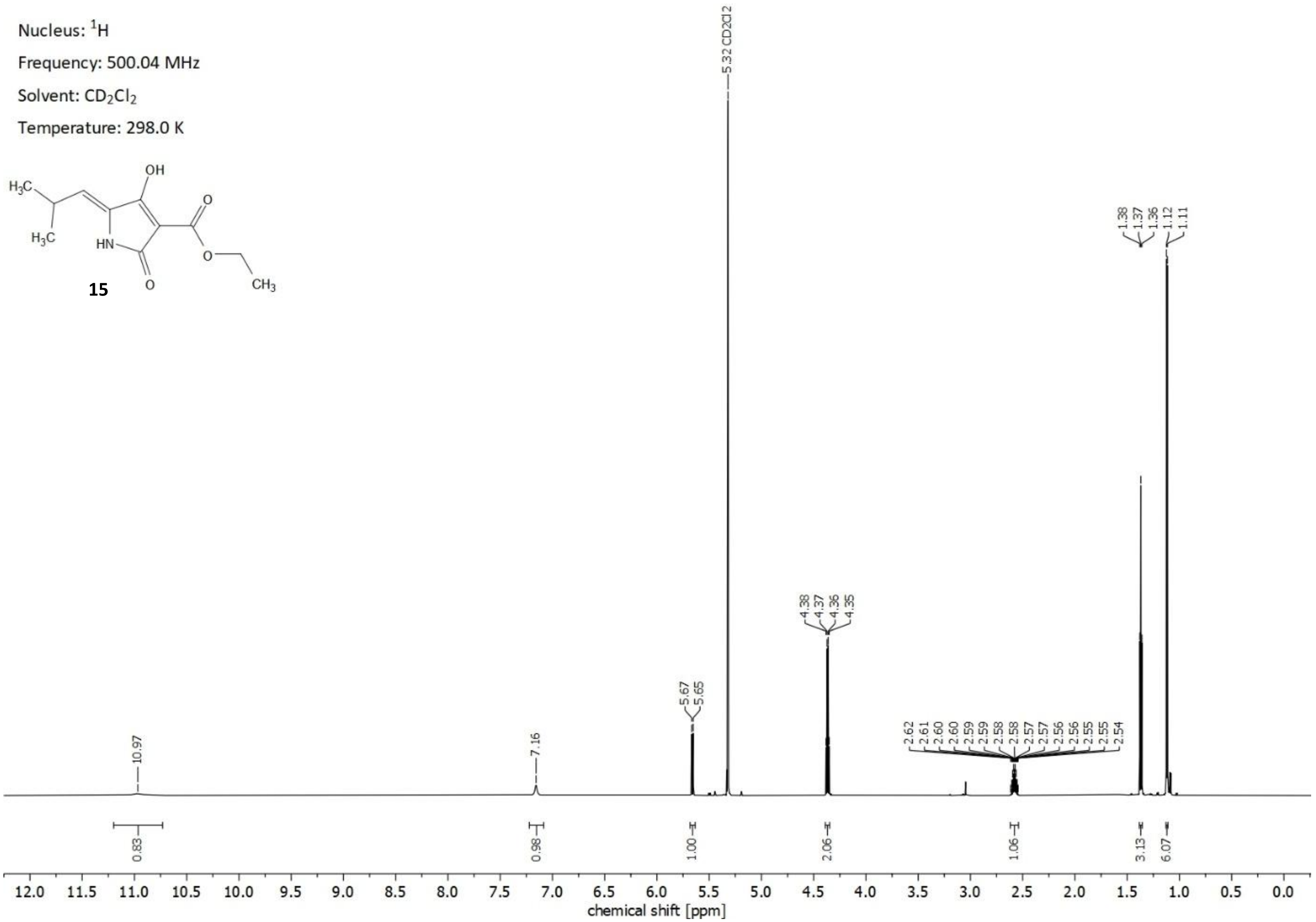

Nucleus: ^{13}C

Frequency: 125.75 MHz

Solvent: CD_2Cl_2

Temperature: 298.0 K

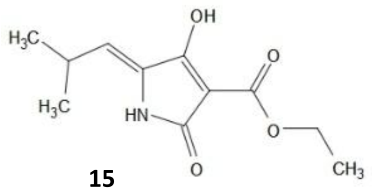

15

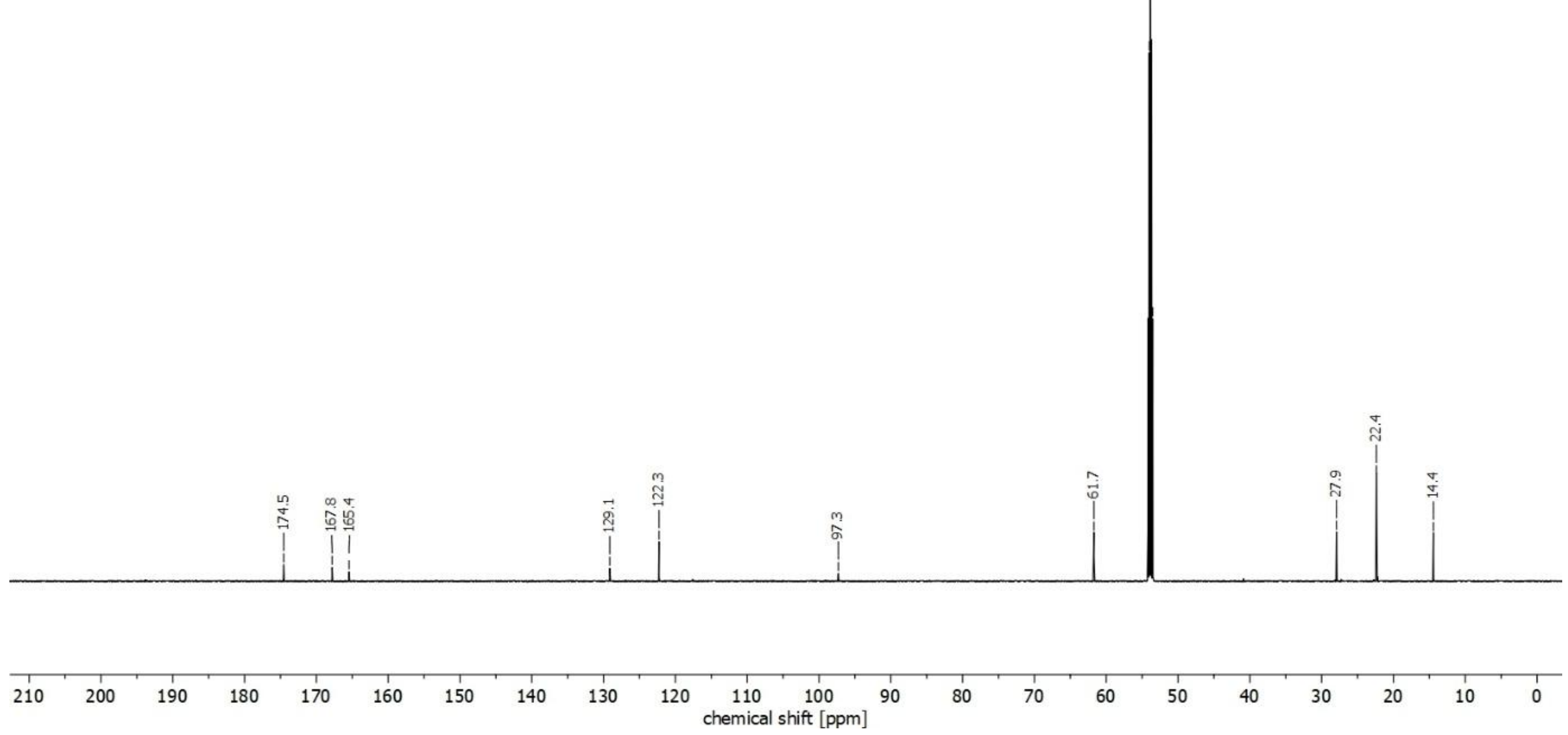

Nucleus: ^{1}H

Frequency: 700.41 MHz

Solvent: CD_2Cl_2

Temperature: 298.0 K

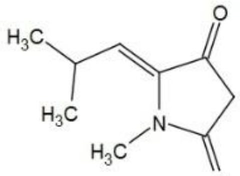

17

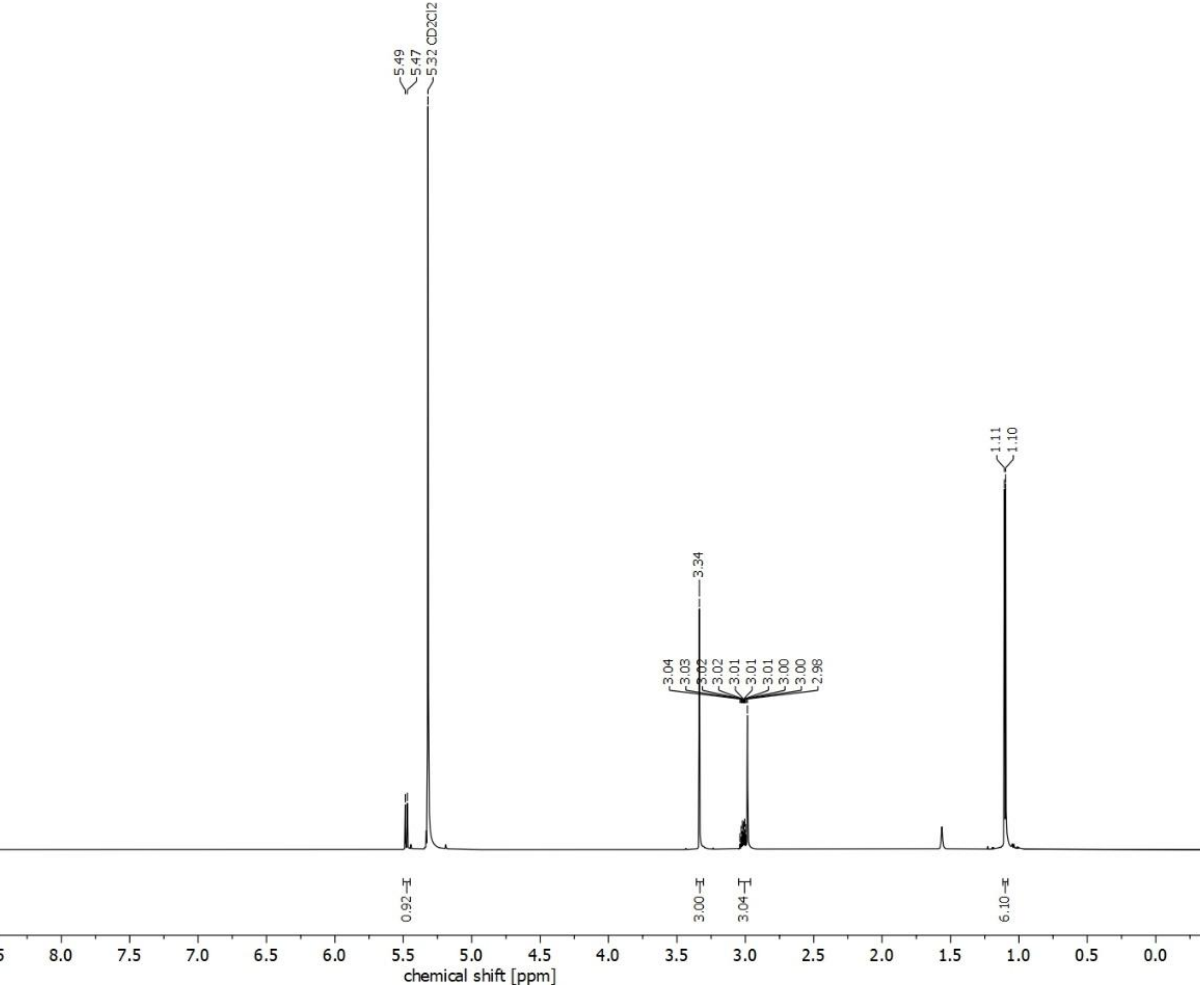

Nucleus: ^{13}C

Frequency: 176.14 MHz

Solvent: CD_2Cl_2

Temperature: 298.0 K

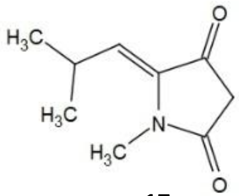

17

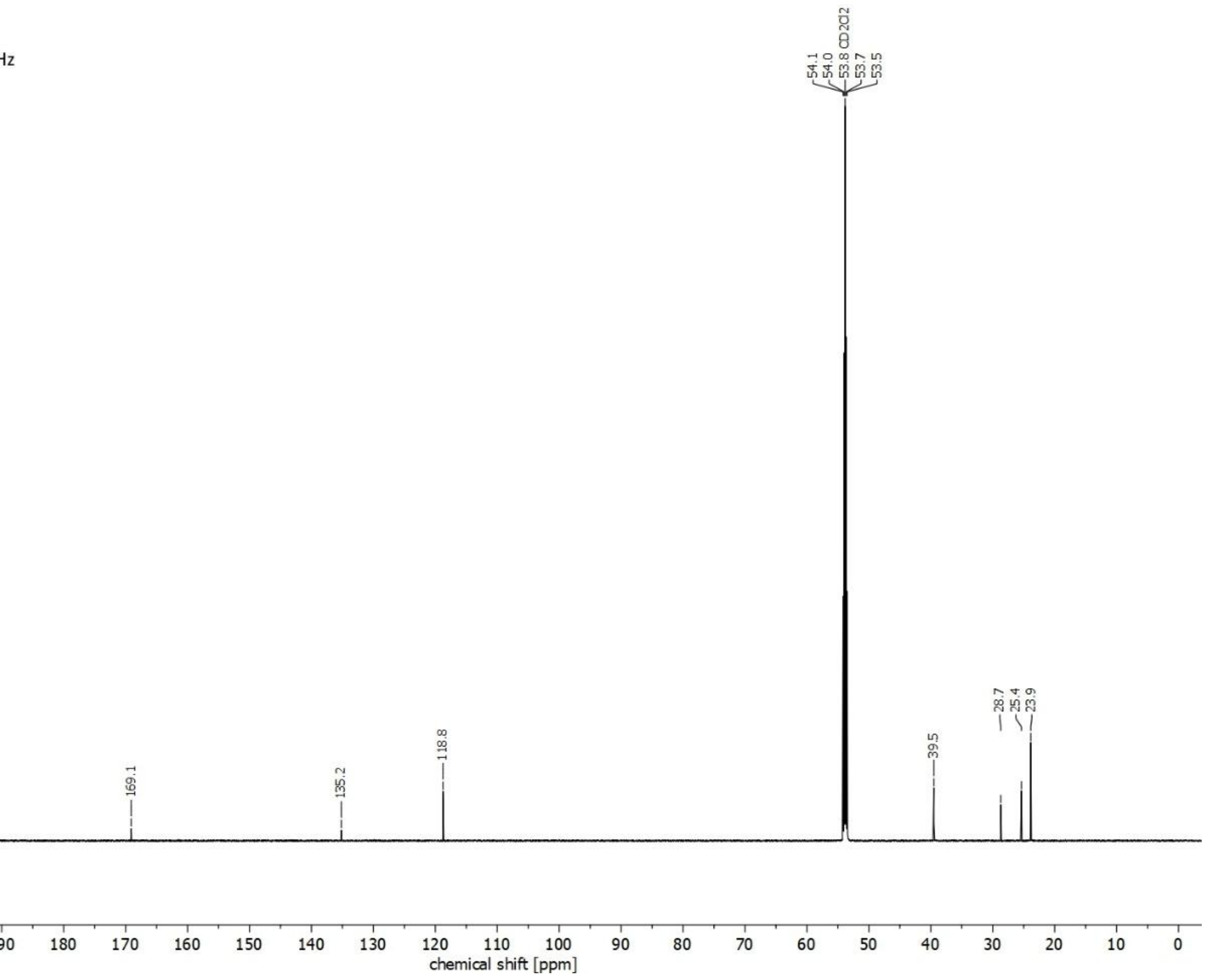

Nucleus: ^{1}H

Frequency: 500.04 MHz

Solvent: CD_2Cl_2

Temperature: 298.0 K

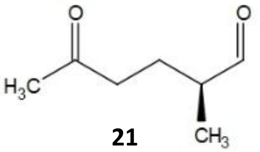

21

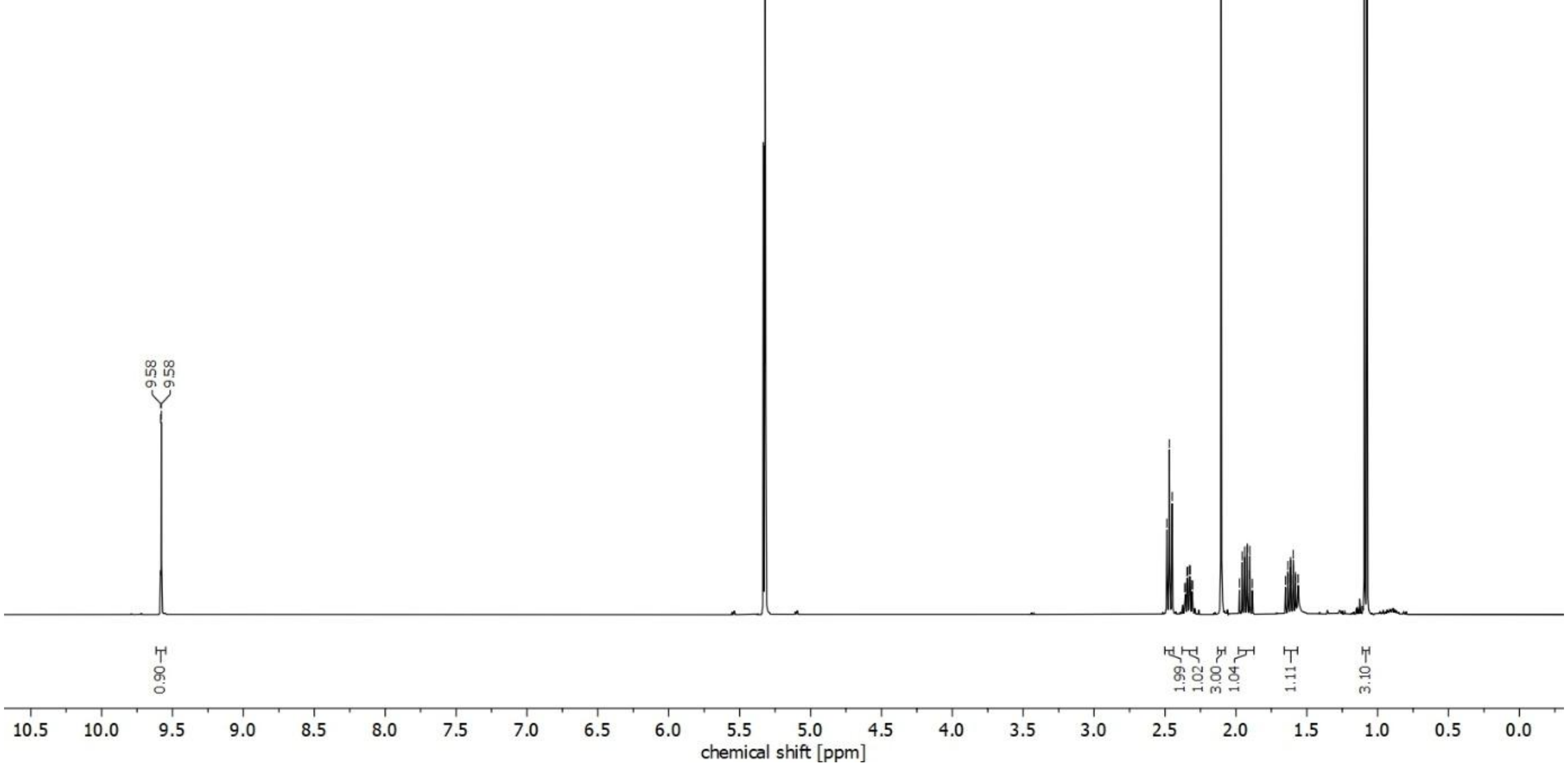

Nucleus: ^{13}C

Frequency: 125.75 MHz

Solvent: CD_2Cl_2

Temperature: 298.0 K

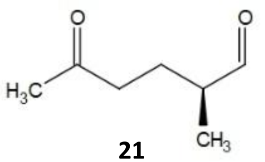

21

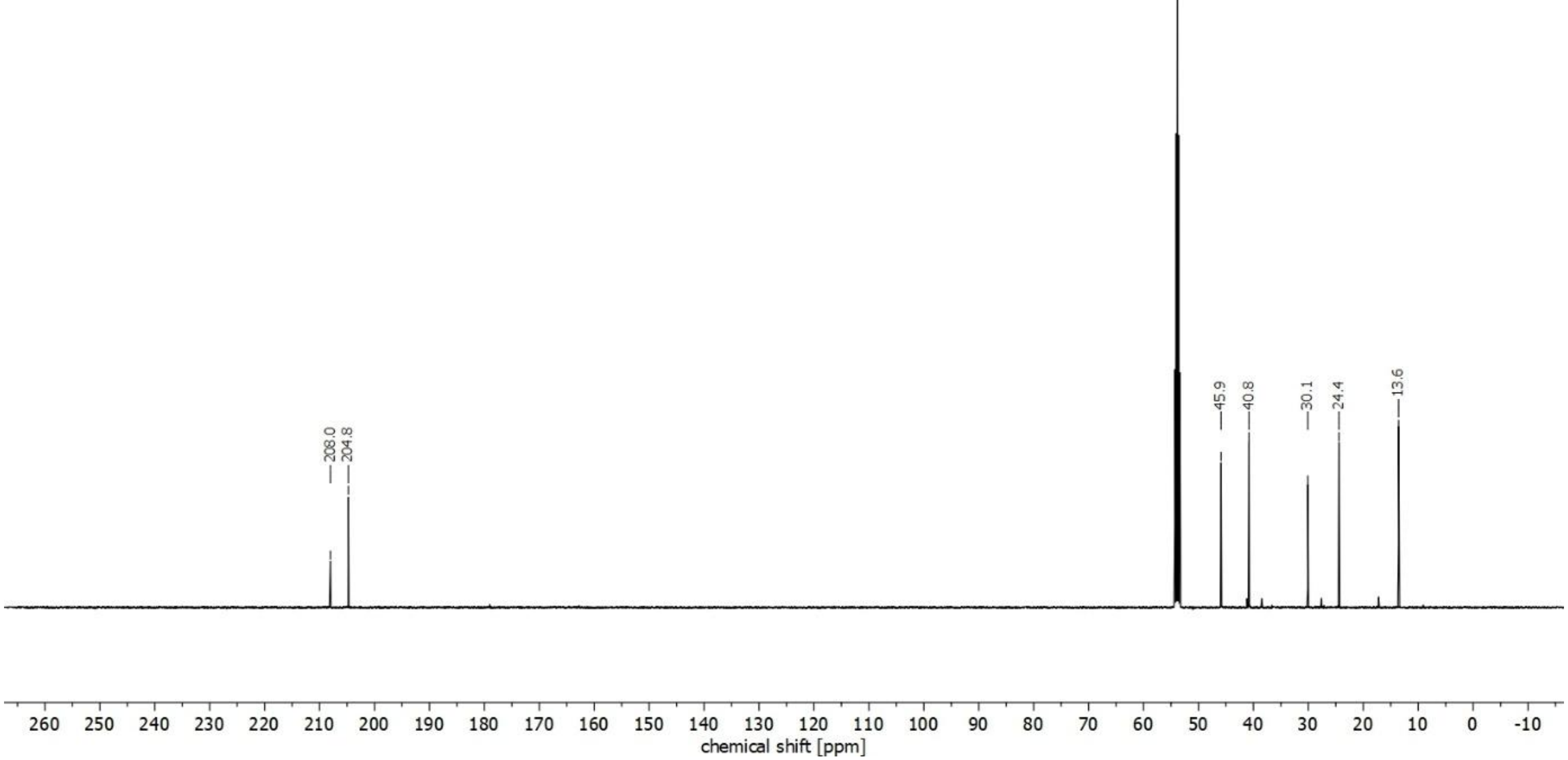

Nucleus: ^{1}H

Frequency: 500.04 MHz

Solvent: CD_2Cl_2

Temperature: 298.0 K

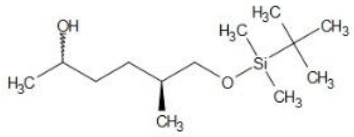

22

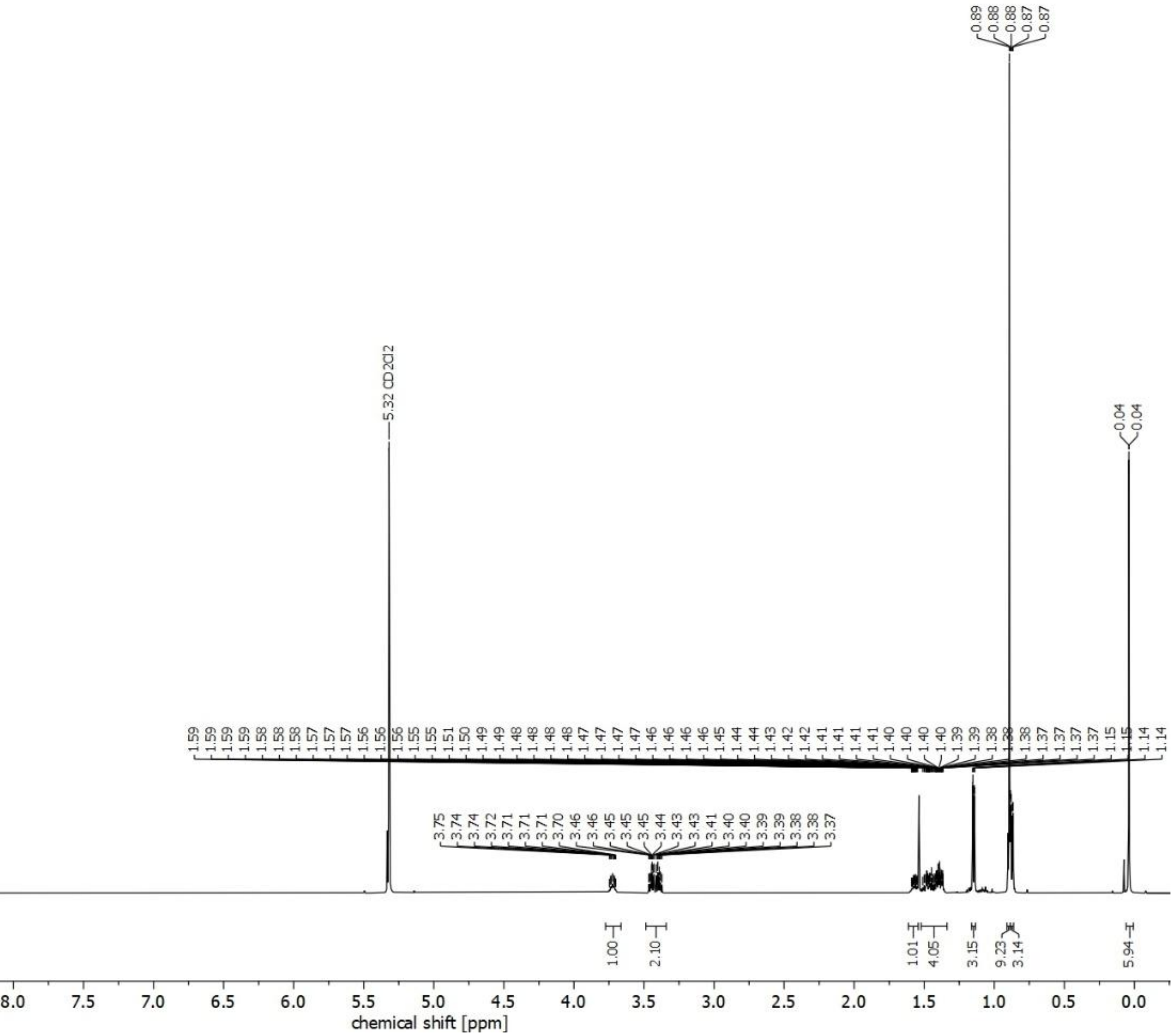

Nucleus: ^{13}C

Frequency: 125.75 MHz

Solvent: CD_2Cl_2

Temperature: 298.0 K

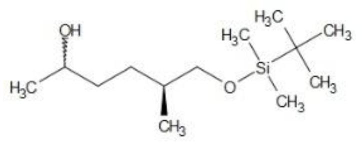

22

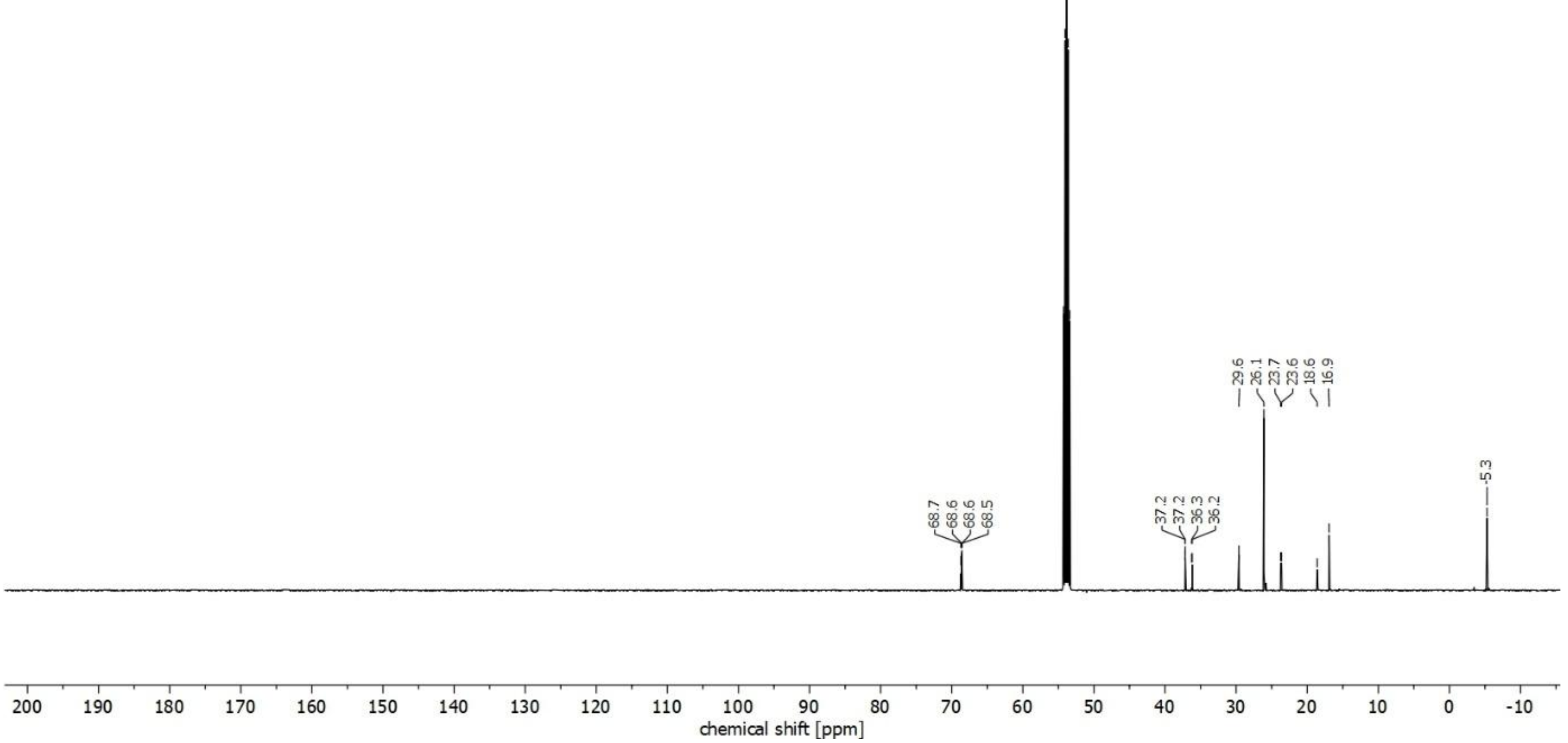

Nucleus: ^{1}H

Frequency: 499.13 MHz

Solvent: CD_2Cl_2

Temperature: 298.0 K

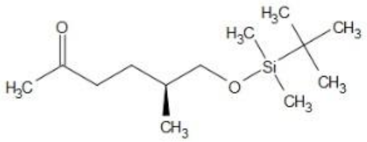

23

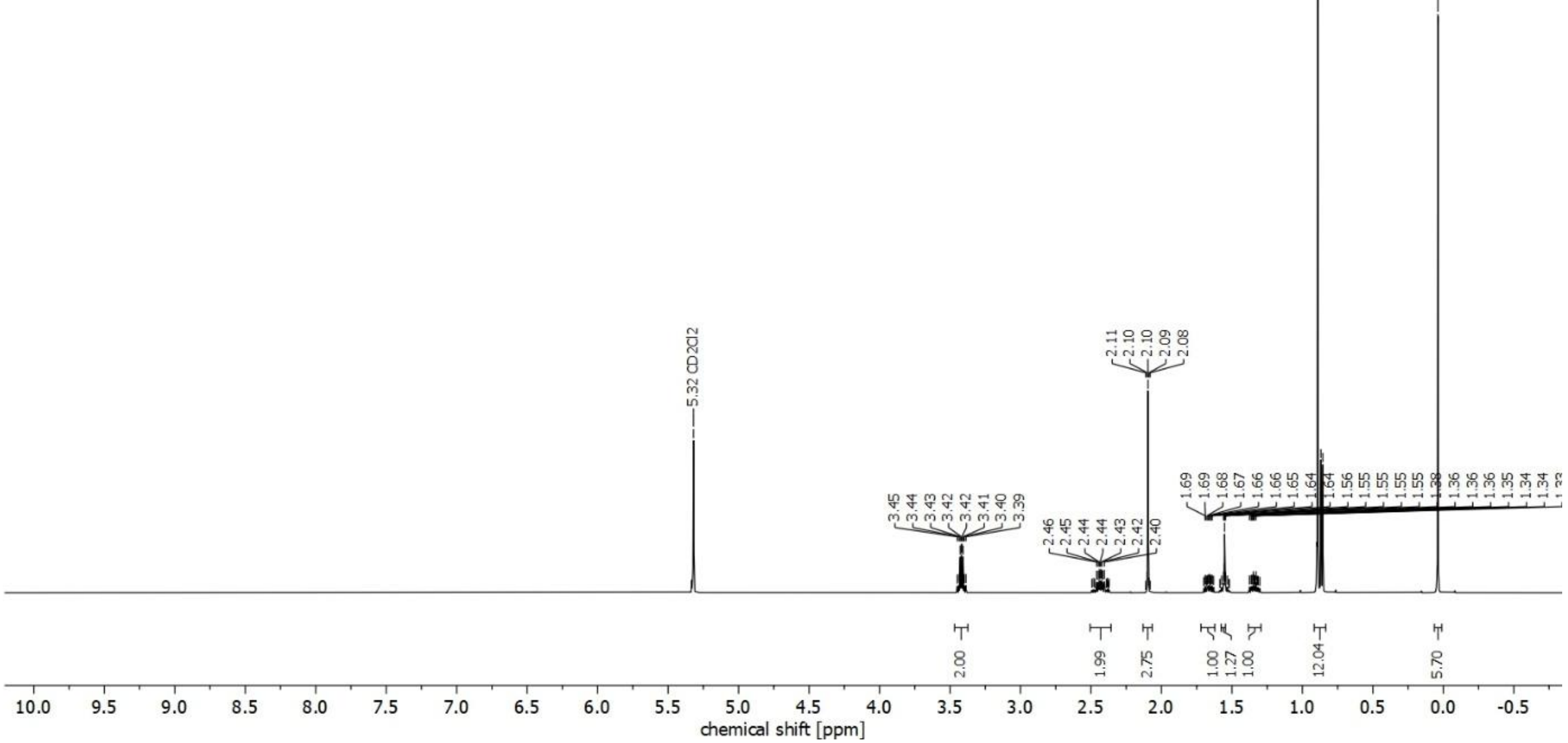

Nucleus: ^{13}C

Frequency: 125.52 MHz

Solvent: CD_2Cl_2

Temperature: 298.0 K

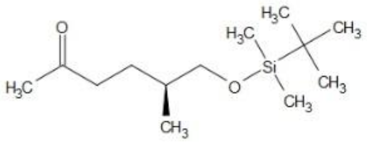

23

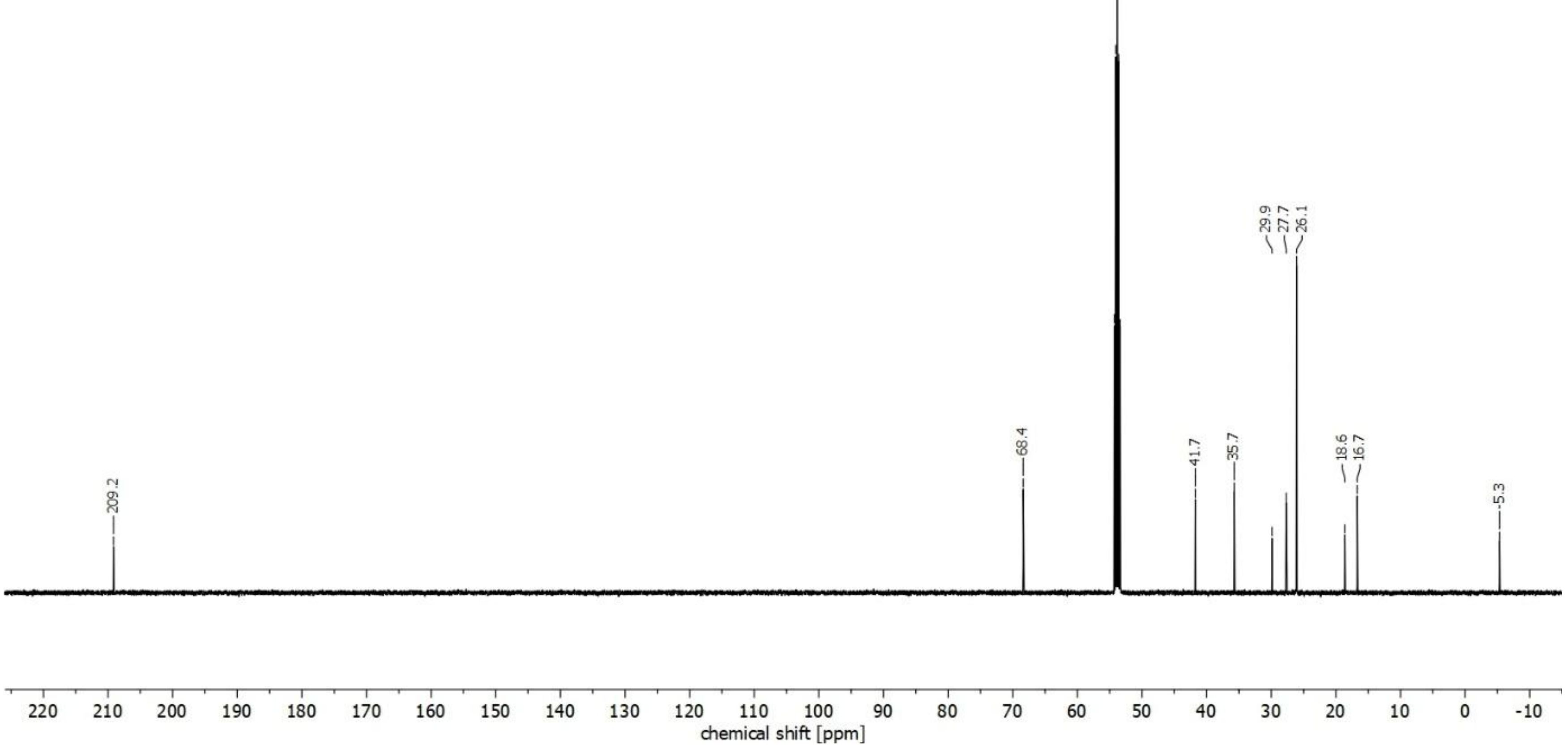

Nucleus: ^{1}H

Frequency: 700.41 MHz

Solvent: CD_2Cl_2

Temperature: 298.0 K

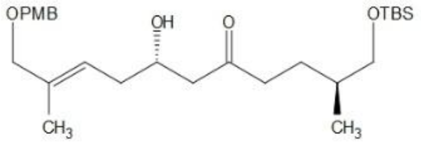

25

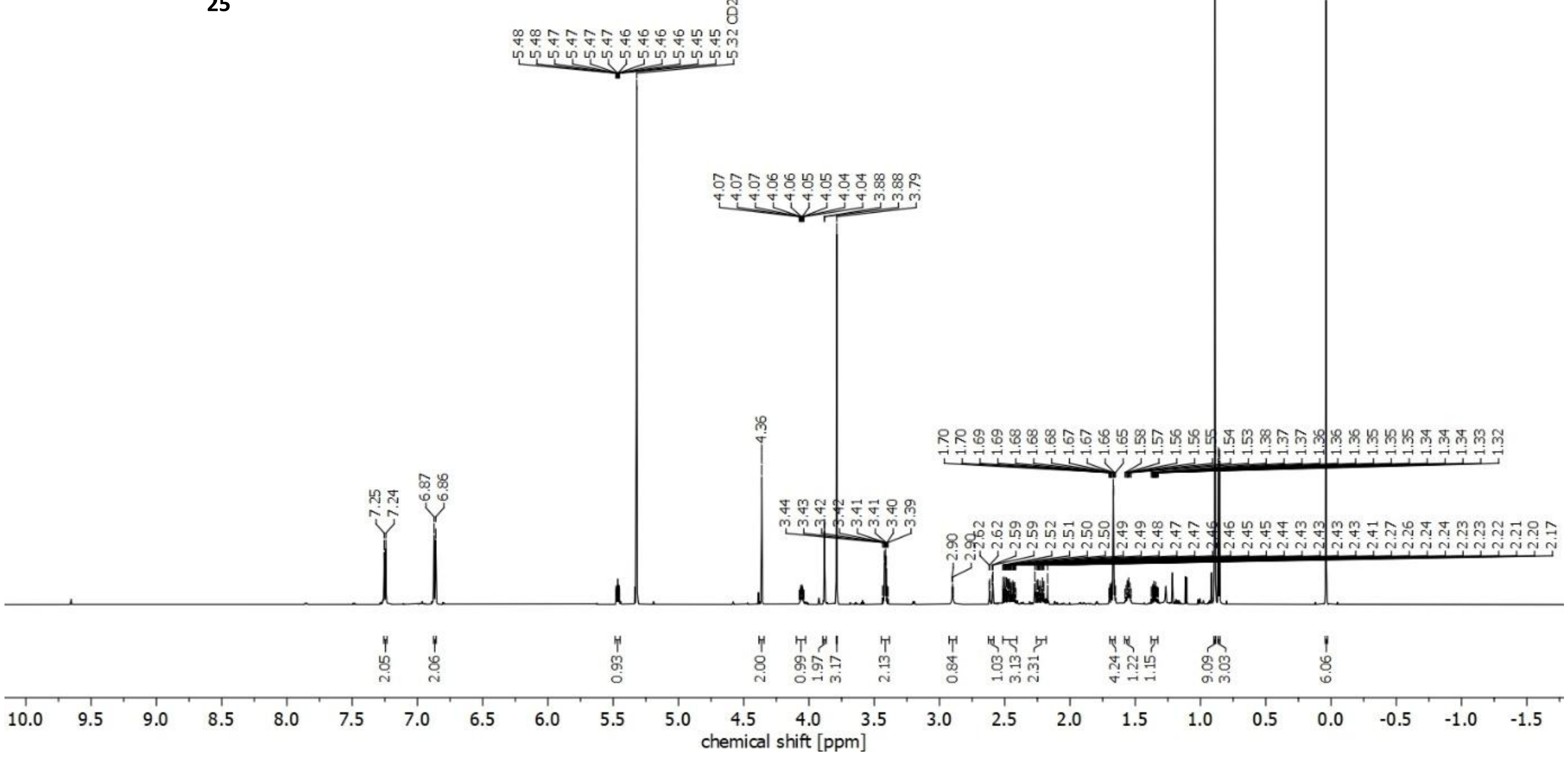

Nucleus: ^{13}C

Frequency: 176.14 MHz

Solvent: CD_2Cl_2

Temperature: 298.0 K

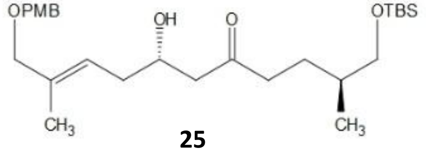

25

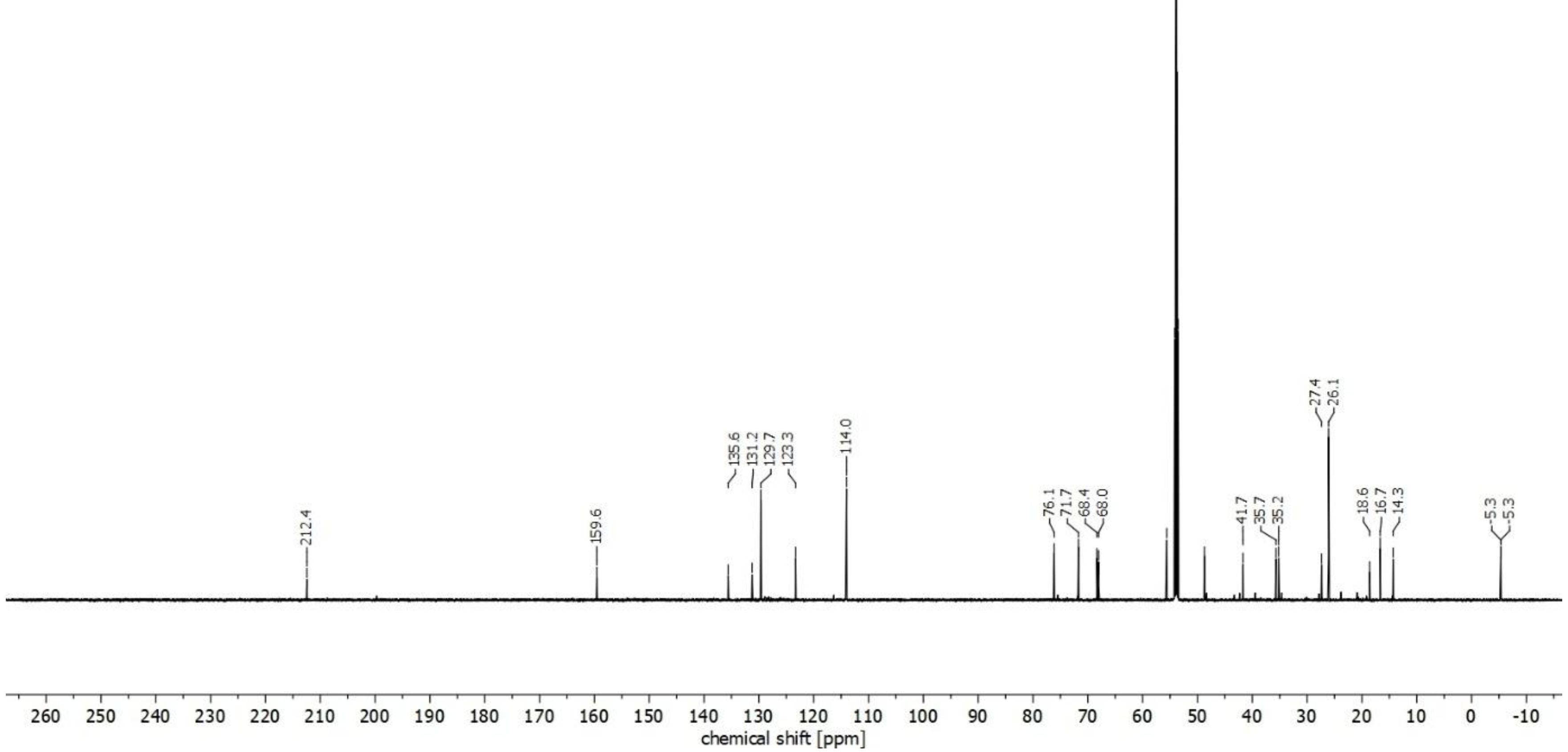

Nucleus: ^{1}H

Frequency: 700.41 MHz

Solvent: CD_2Cl_2

Temperature: 298.0 K

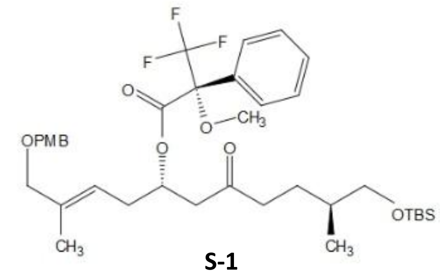

S-1

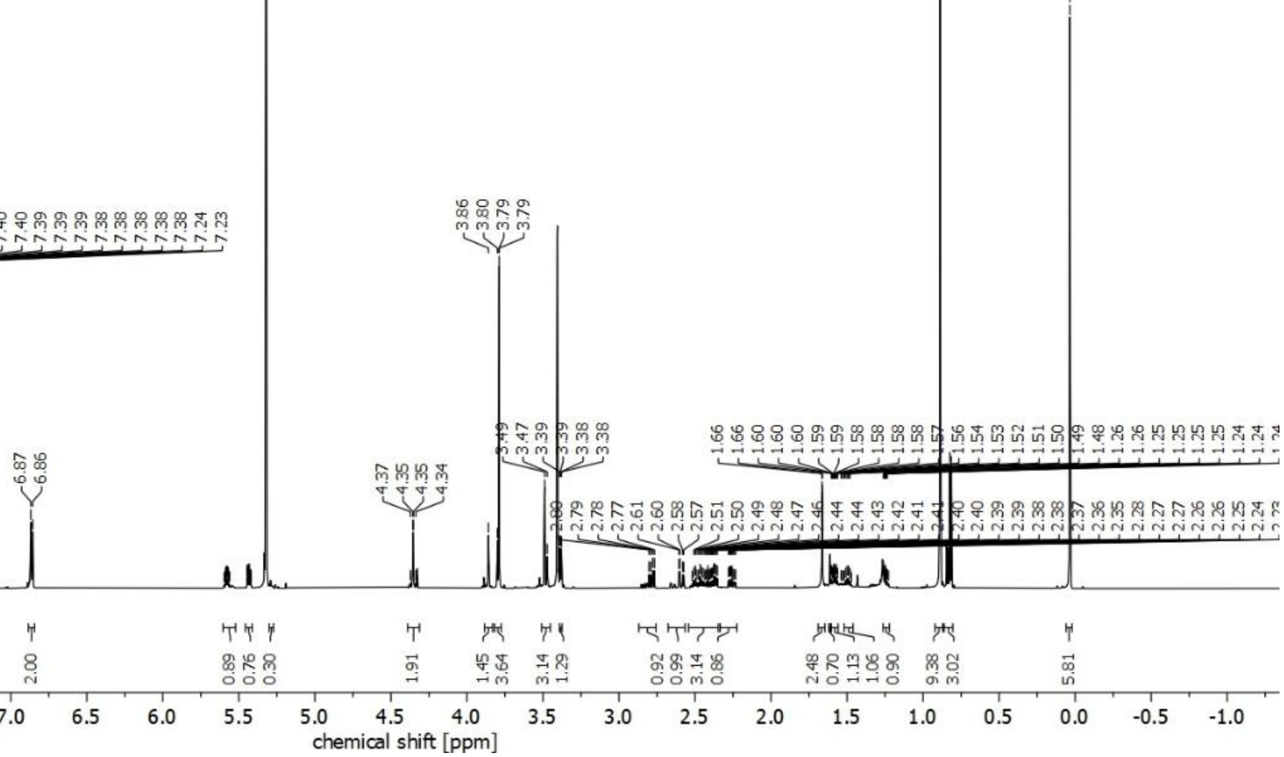

Nucleus: ^{13}C

Frequency: 176.14 MHz

Solvent: CD_2Cl_2

Temperature: 298.0 K

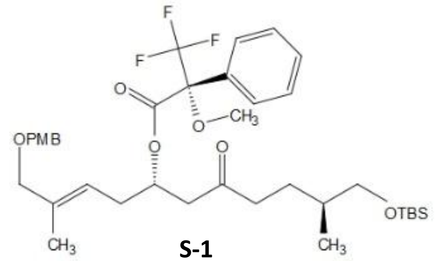

S-1

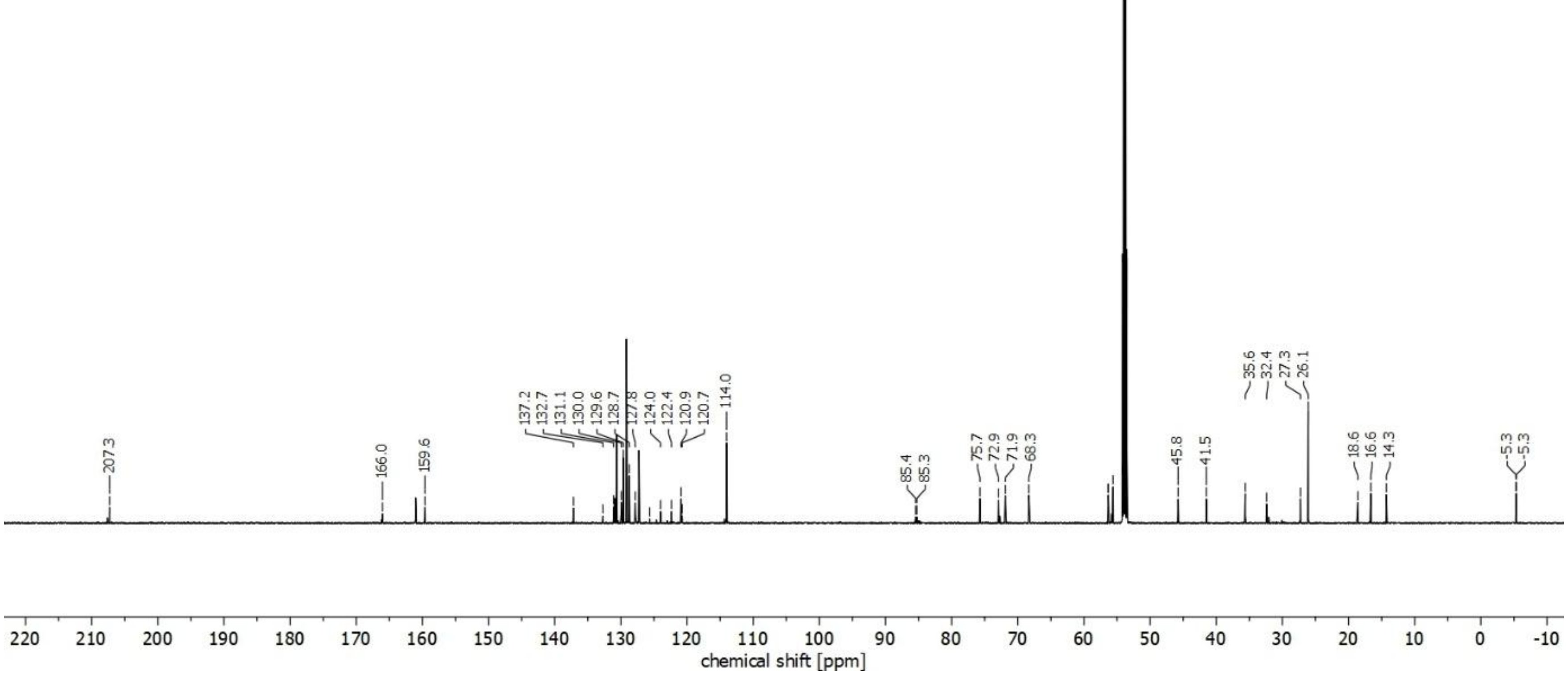

Nucleus: ^{1}H

Frequency: 700.41 MHz

Solvent: CD_2Cl_2

Temperature: 298.0 K

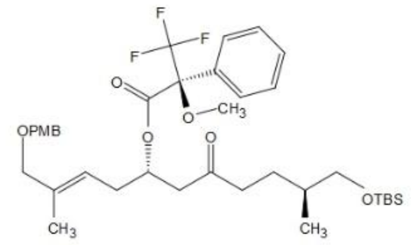

S-2

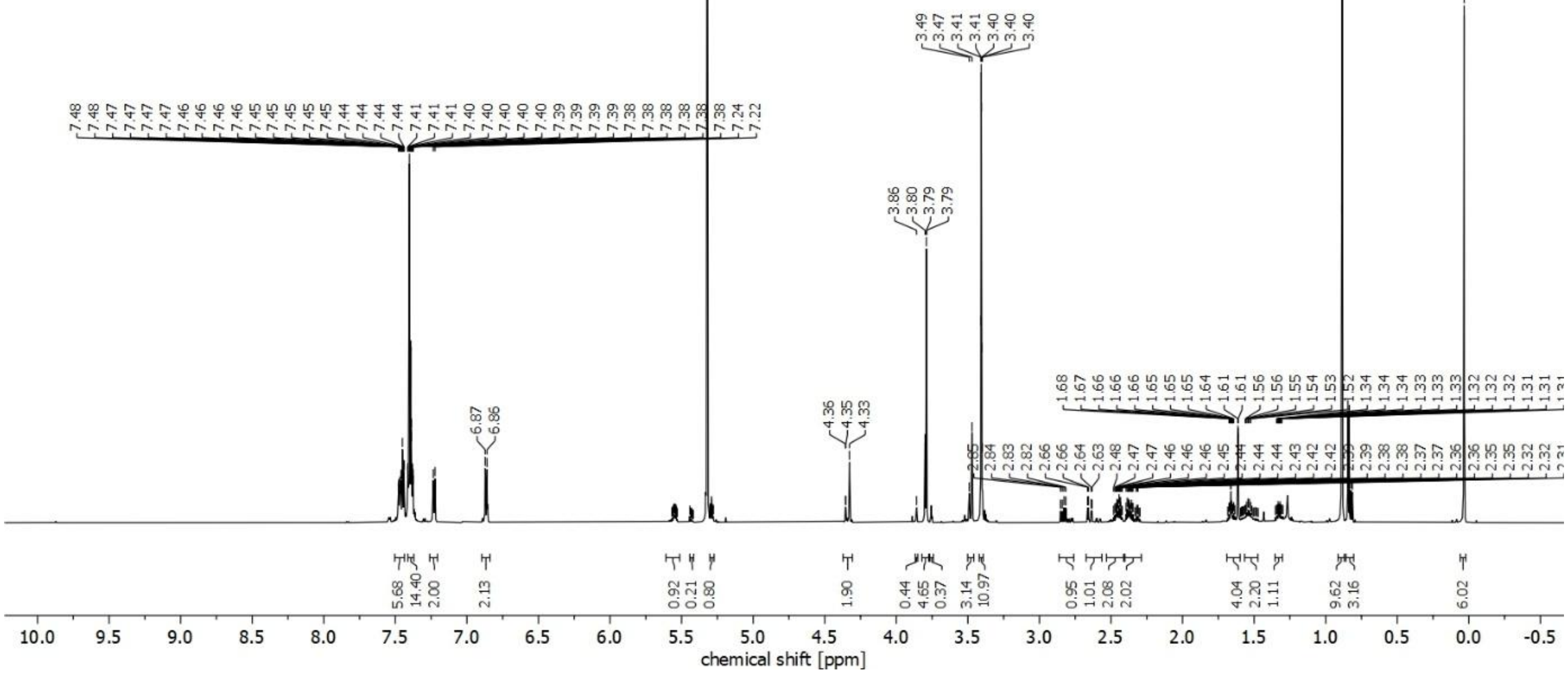

Nucleus: ^{13}C

Frequency: 176.14 MHz

Solvent: CD_2Cl_2

Temperature: 298.0 K

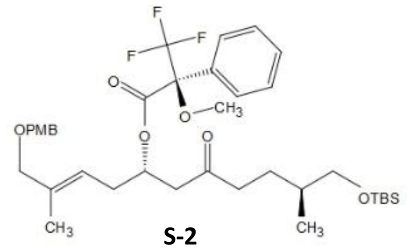

S-2

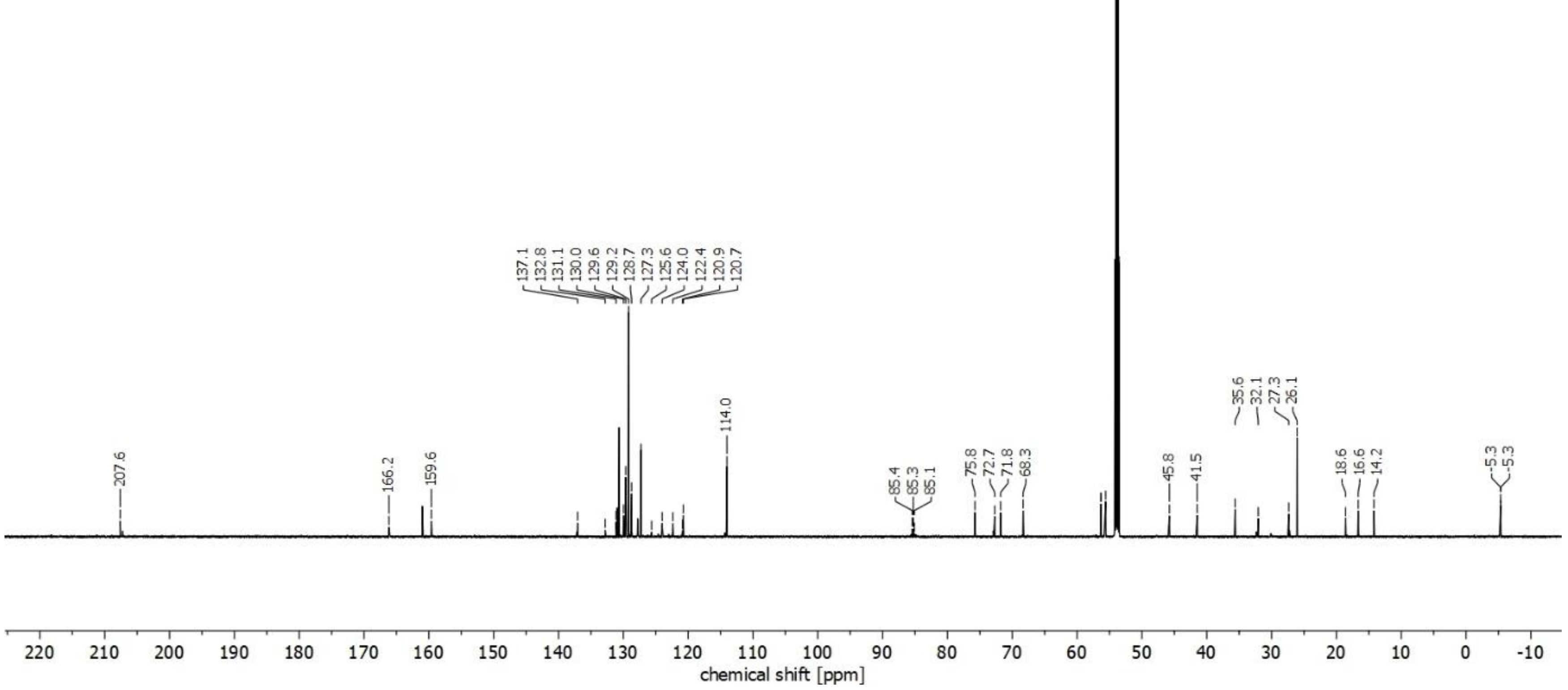

Nucleus: ^{1}H

Frequency: 700.41 MHz

Solvent: CD_2Cl_2

Temperature: 298.0 K

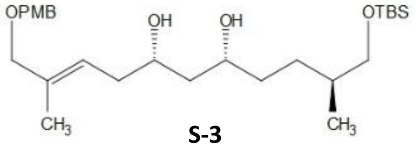

S-3

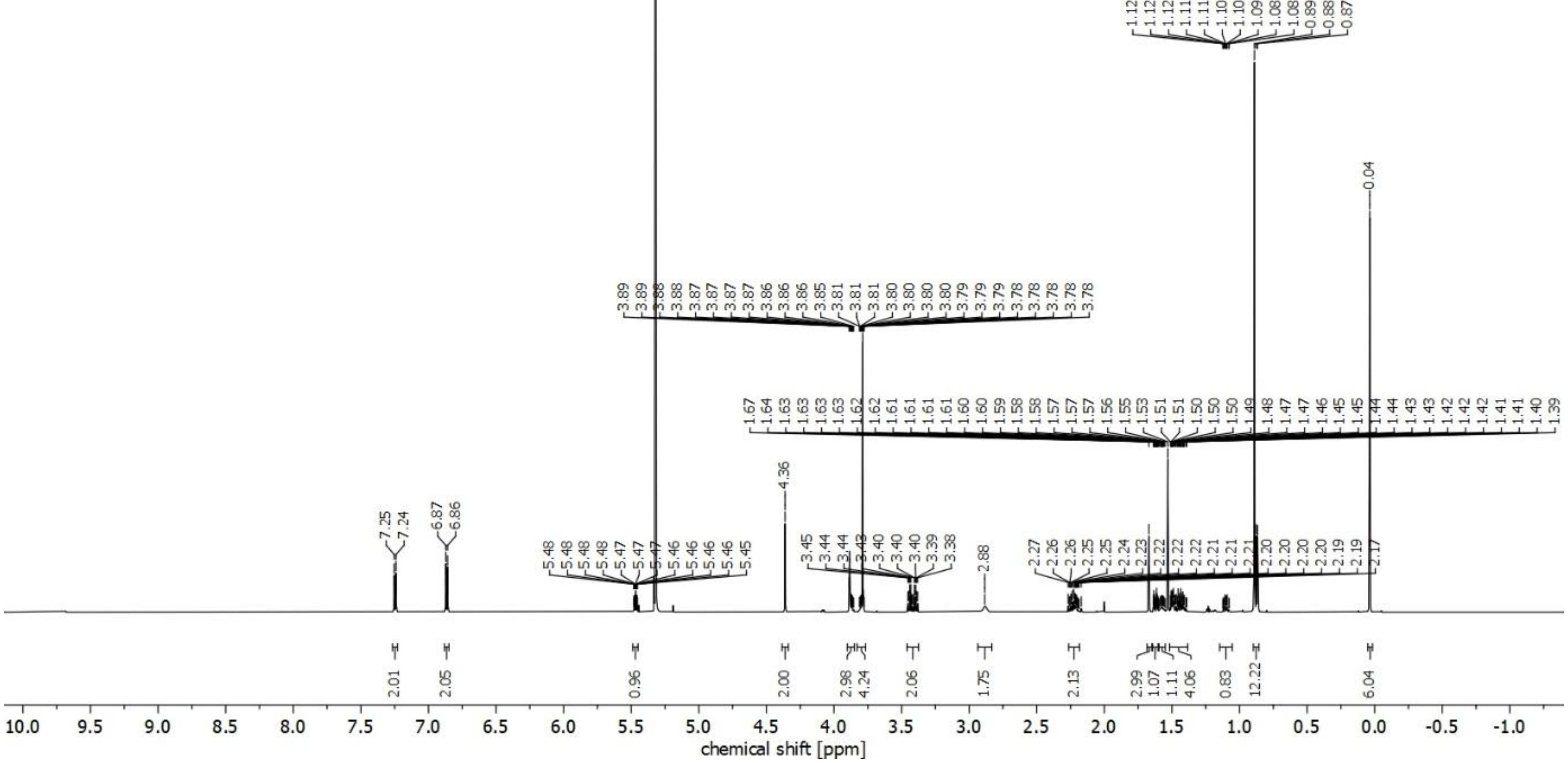

Nucleus: ^{13}C

Frequency: 176.14 MHz

Solvent: CD_2Cl_2

Temperature: 298.0 K

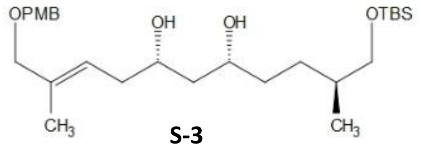

S-3

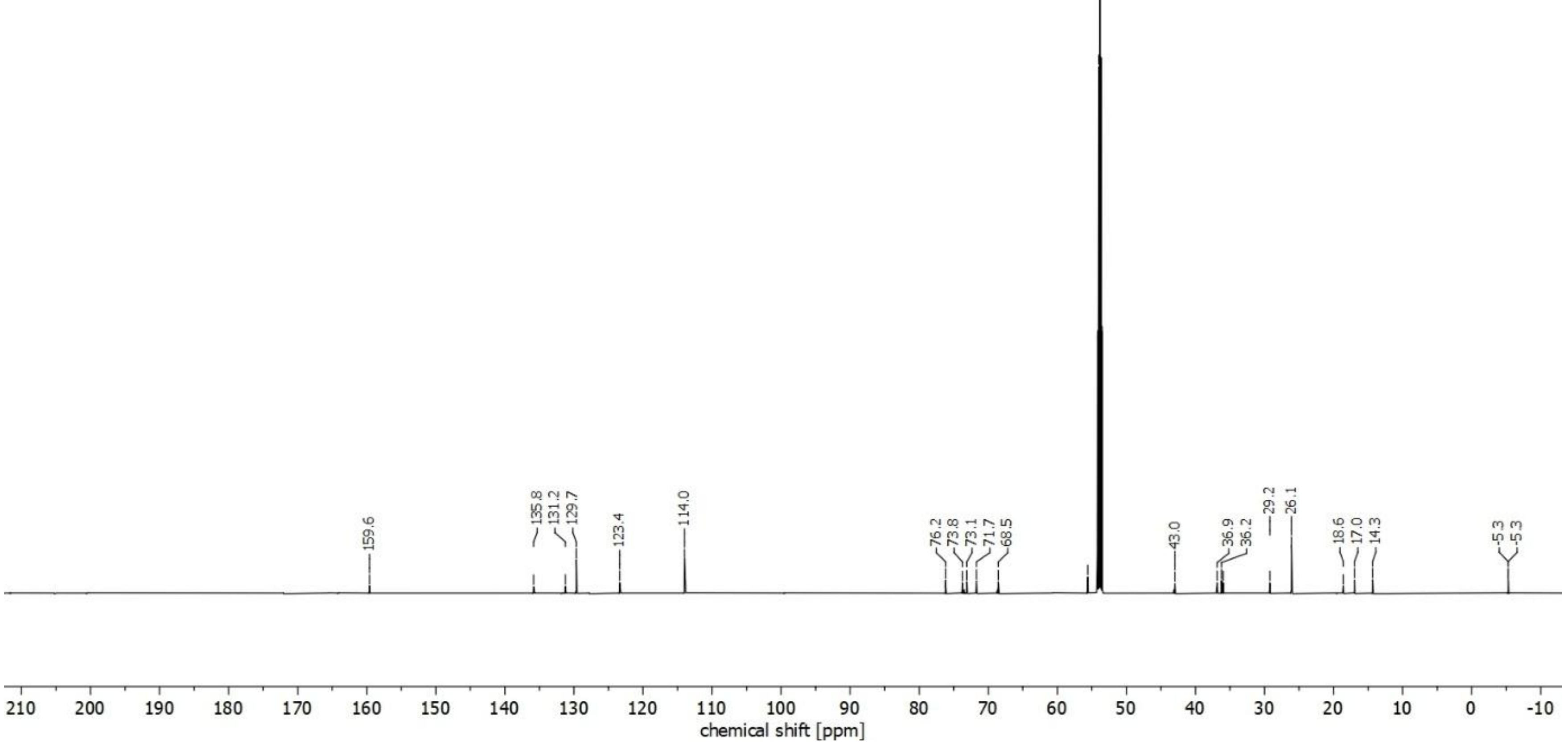

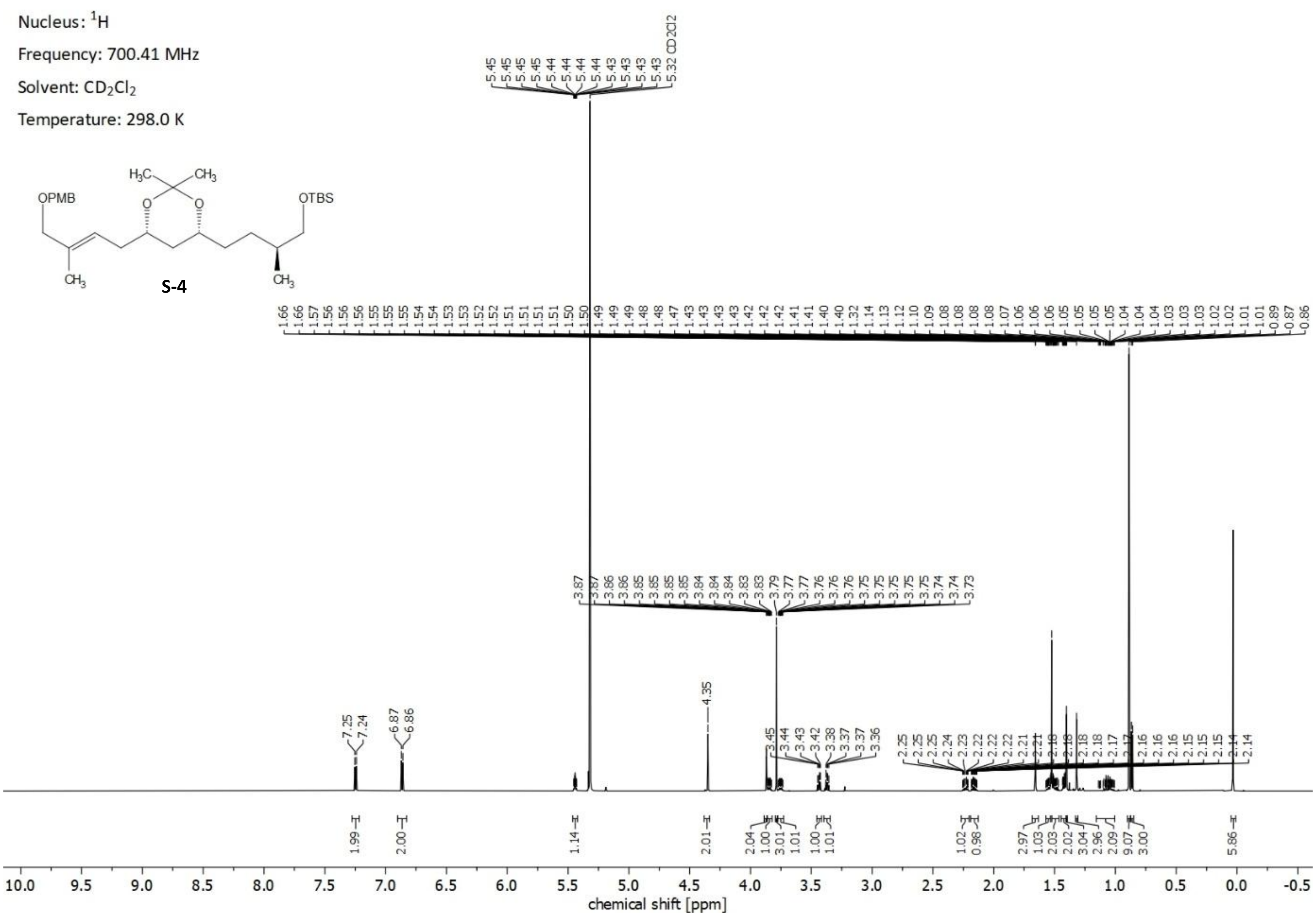
Nucleus: ^{1}H
Frequency: 700.41 MHz
Solvent: CD_2Cl_2
Temperature: 298.0 K
OPMB
OTBS
S-4
chemical shift [ppm]

Nucleus: ^{13}C

Frequency: 176.14 MHz

Solvent: CD_2Cl_2

Temperature: 298.0 K

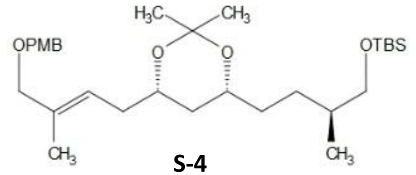

S-4

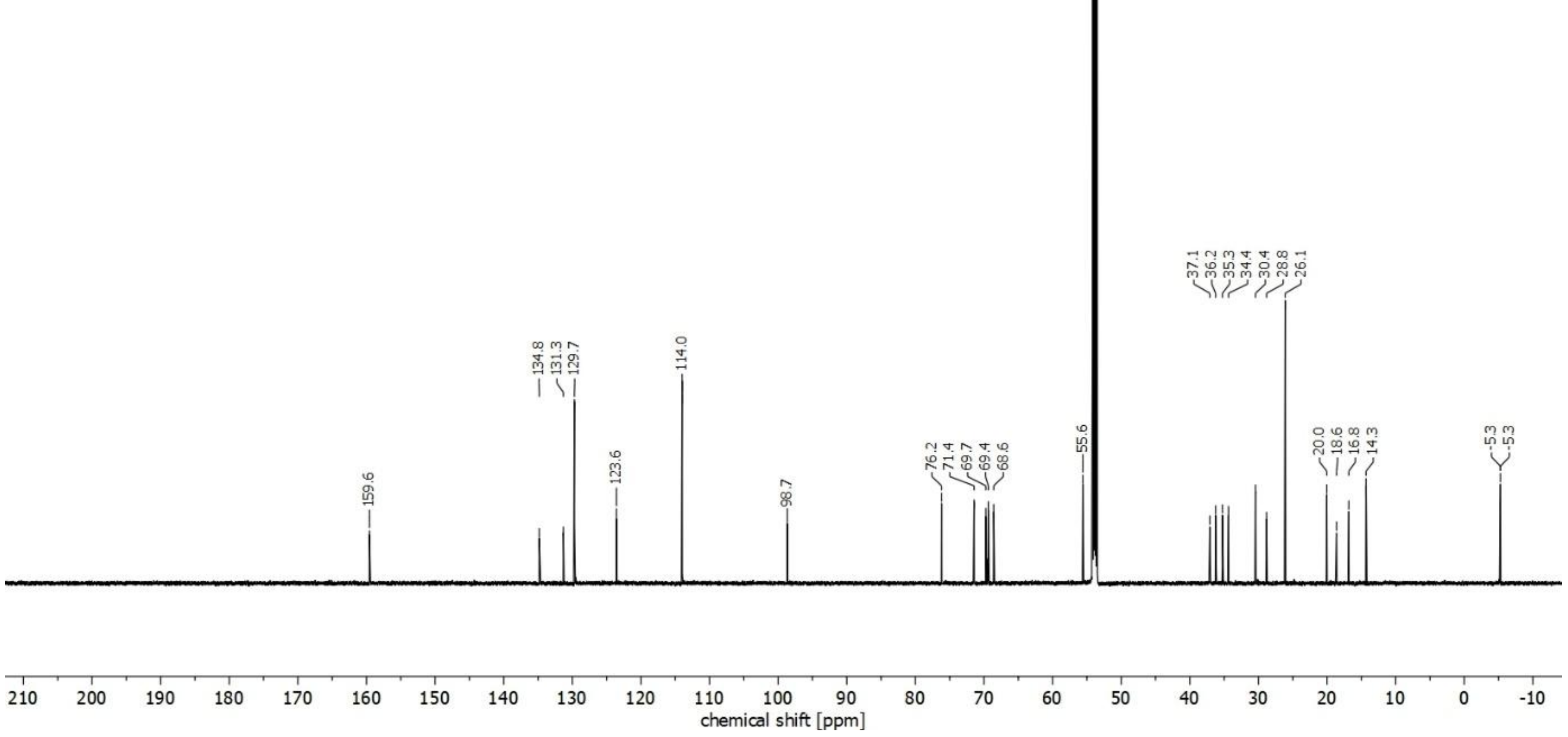

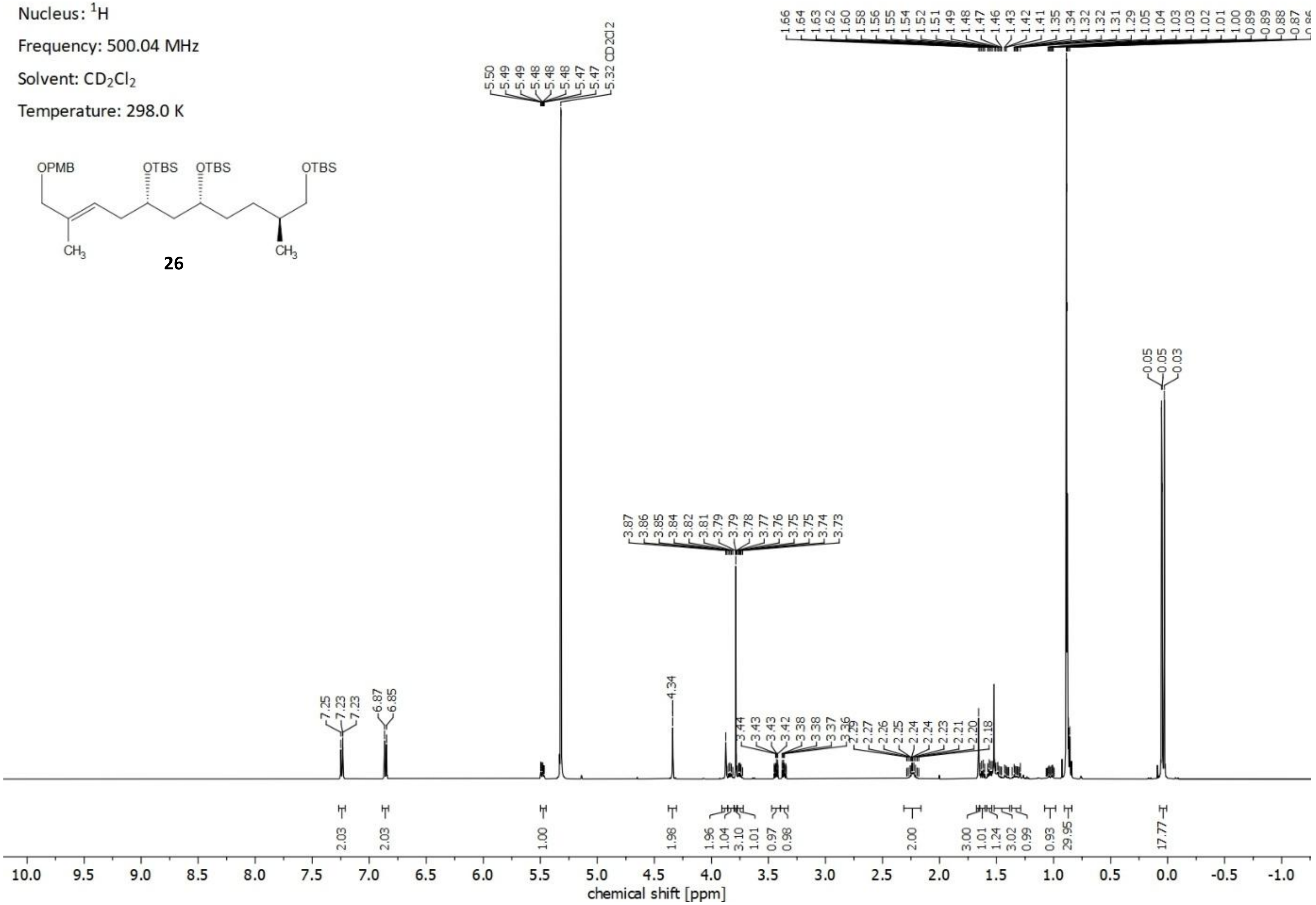
Nucleus: ^{1}H
Frequency: 500.04 MHz
Solvent: CD_2Cl_2
Temperature: 298.0 K
OPMB
OTBS
OTBS
OTBS
CH_3
CH_3
26
5.32 CD2Cl2
chemical shift [ppm]

Nucleus: ^{13}C

Frequency: 125.75 MHz

Solvent: CD_2Cl_2

Temperature: 298.0 K

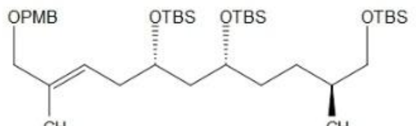

26

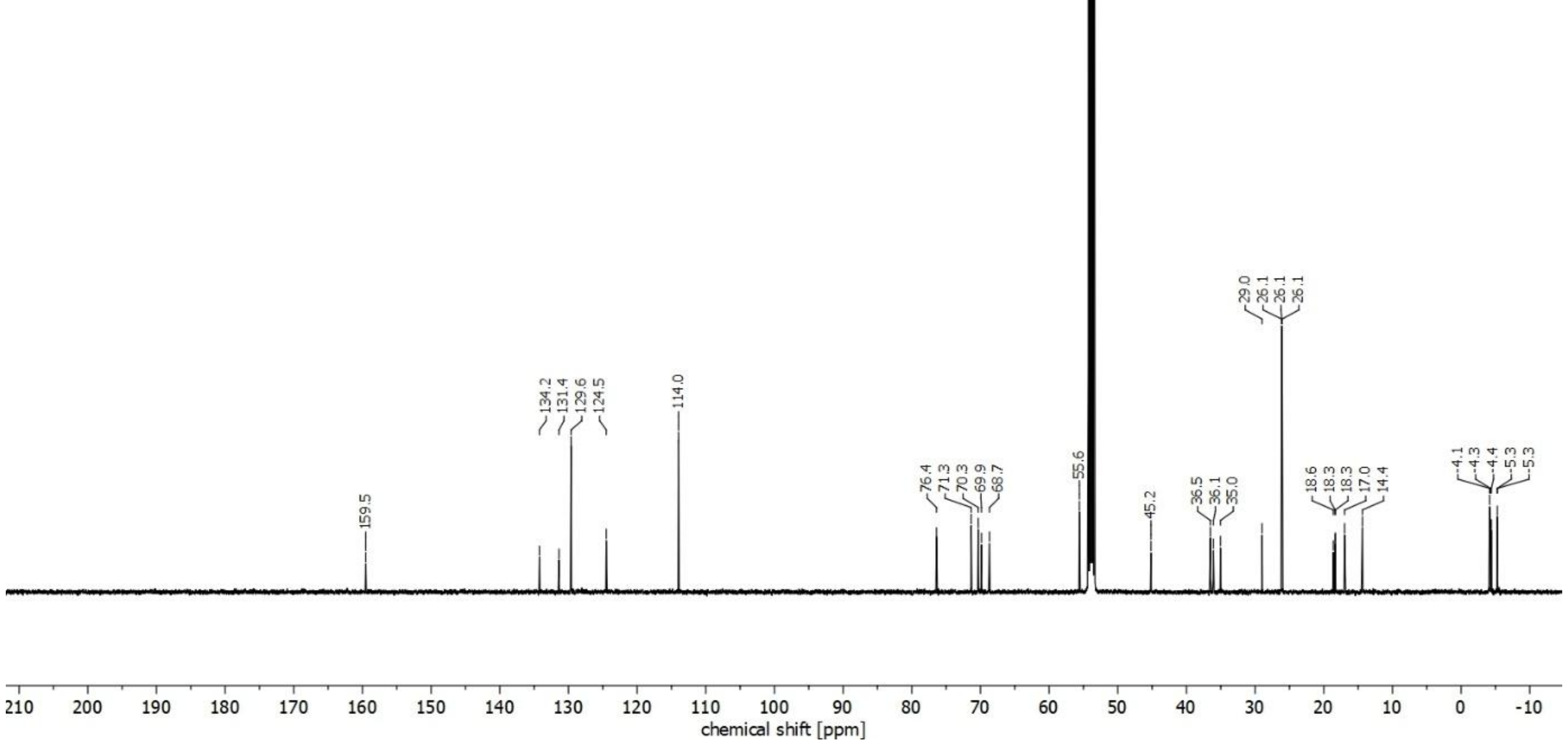

Nucleus: ^{1}H

Frequency: 499.13 MHz

Solvent: CD_2Cl_2

Temperature: 298.0 K

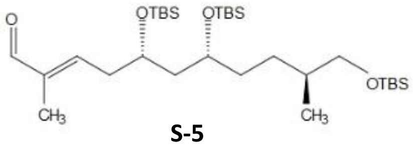

S-5

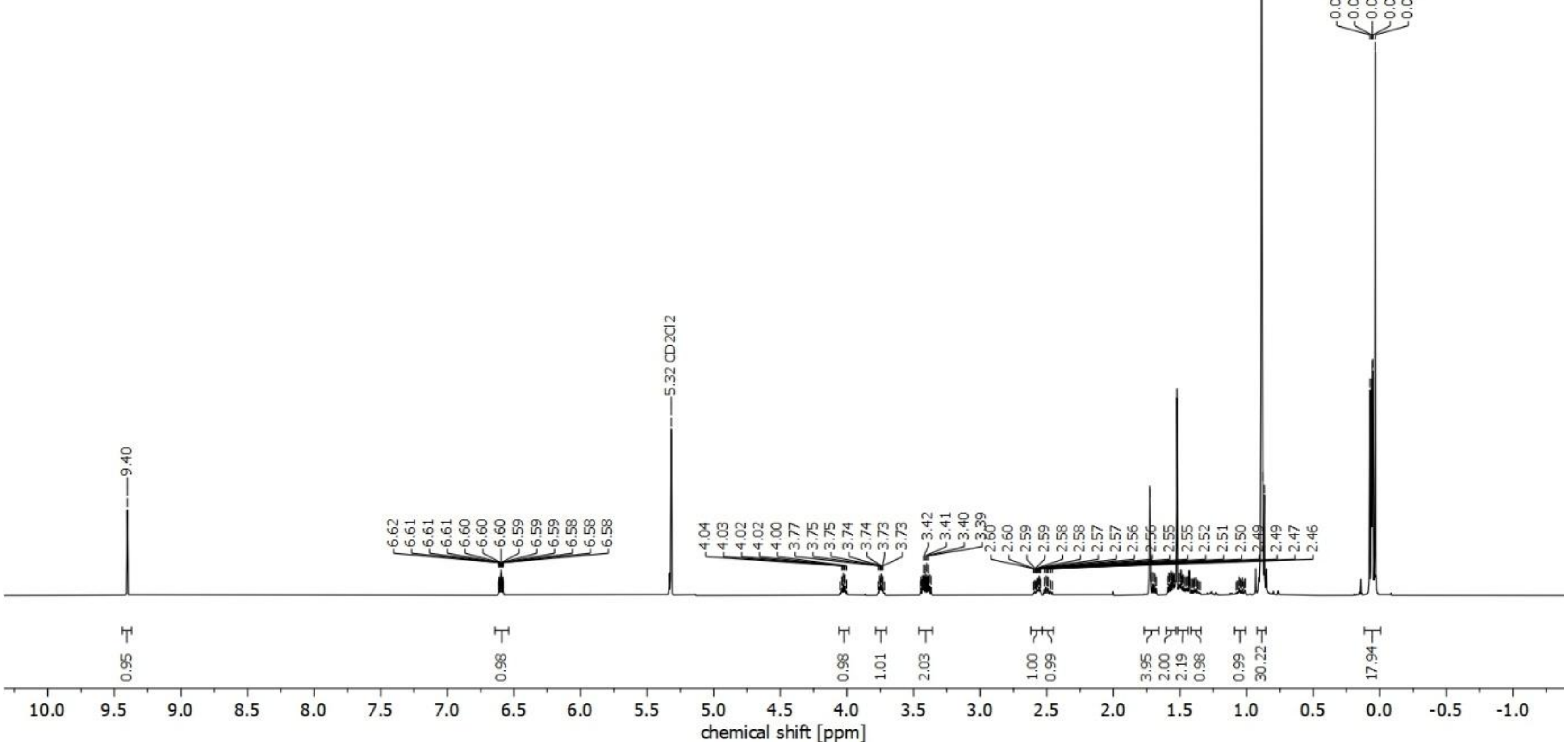

Nucleus: ^{13}C

Frequency: 125.52 MHz

Solvent: CD_2Cl_2

Temperature: 298.0 K

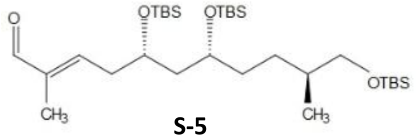

S-5

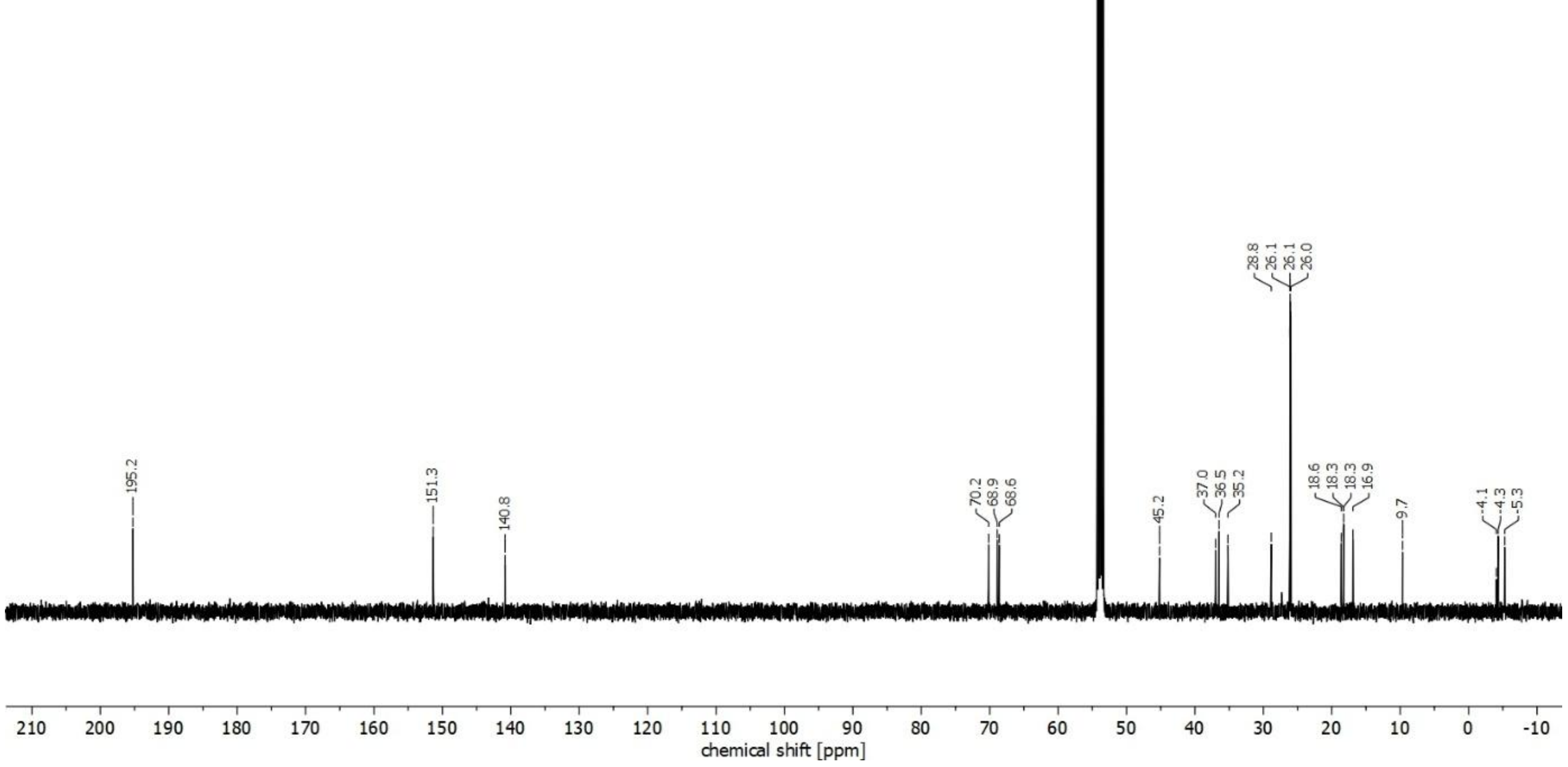

Nucleus: ^{1}H

Frequency: 700.41 MHz

Solvent: CD_2Cl_2

Temperature: 298.0 K

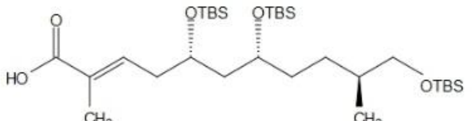

27

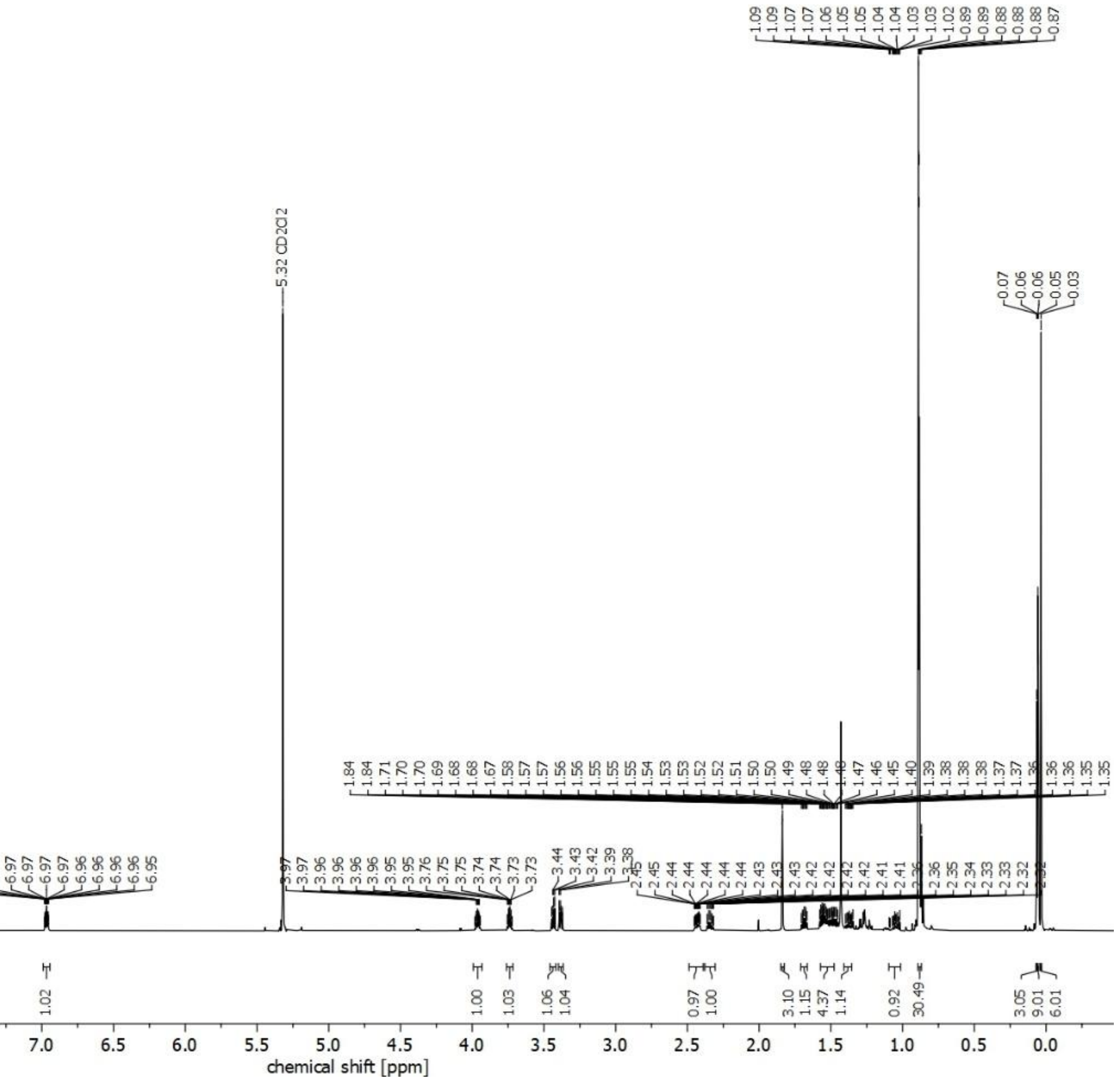

Nucleus: ^{13}C

Frequency: 176.14 MHz

Solvent: CD_2Cl_2

Temperature: 298.0 K

27

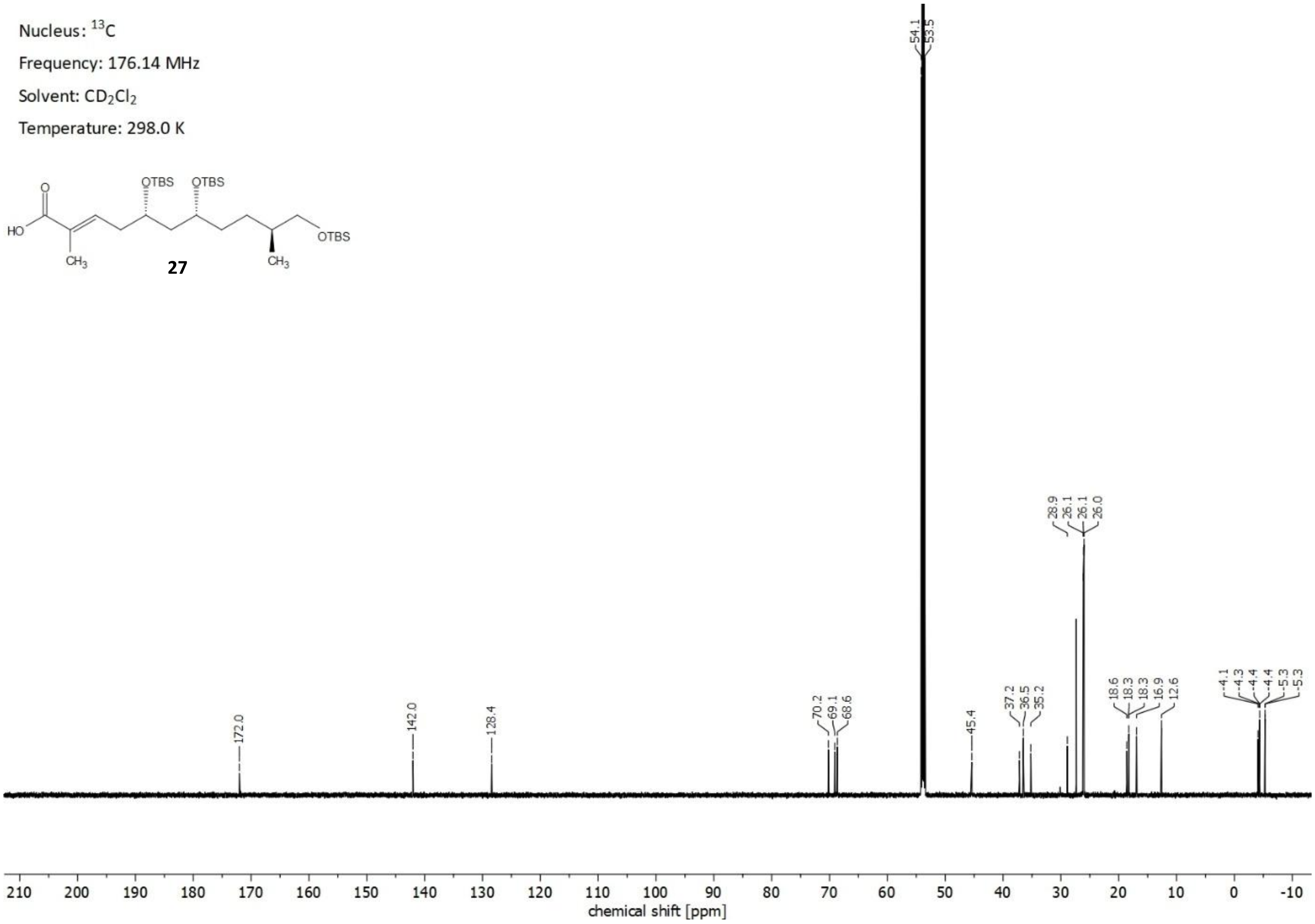

Nucleus: ^{1}H

Frequency: 499.13 MHz

Solvent: CD_2Cl_2

Temperature: 298.0 K

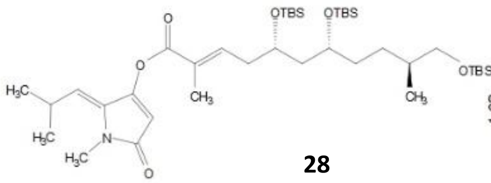

28

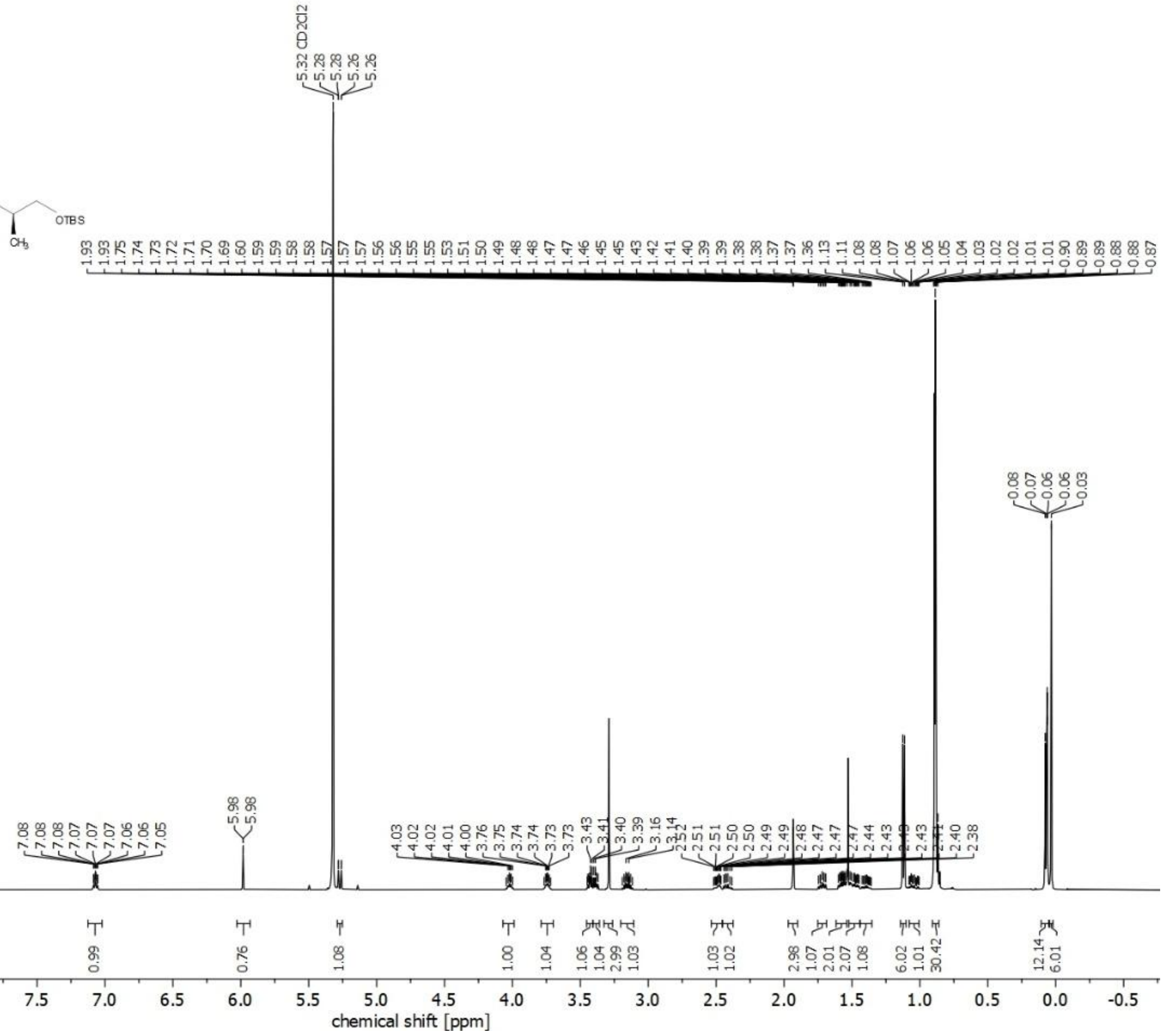

Nucleus: ^{13}C

Frequency: 125.52 MHz

Solvent: CD_2Cl_2

Temperature: 298.0 K

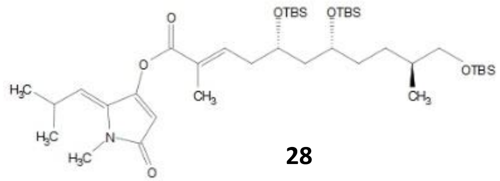

28

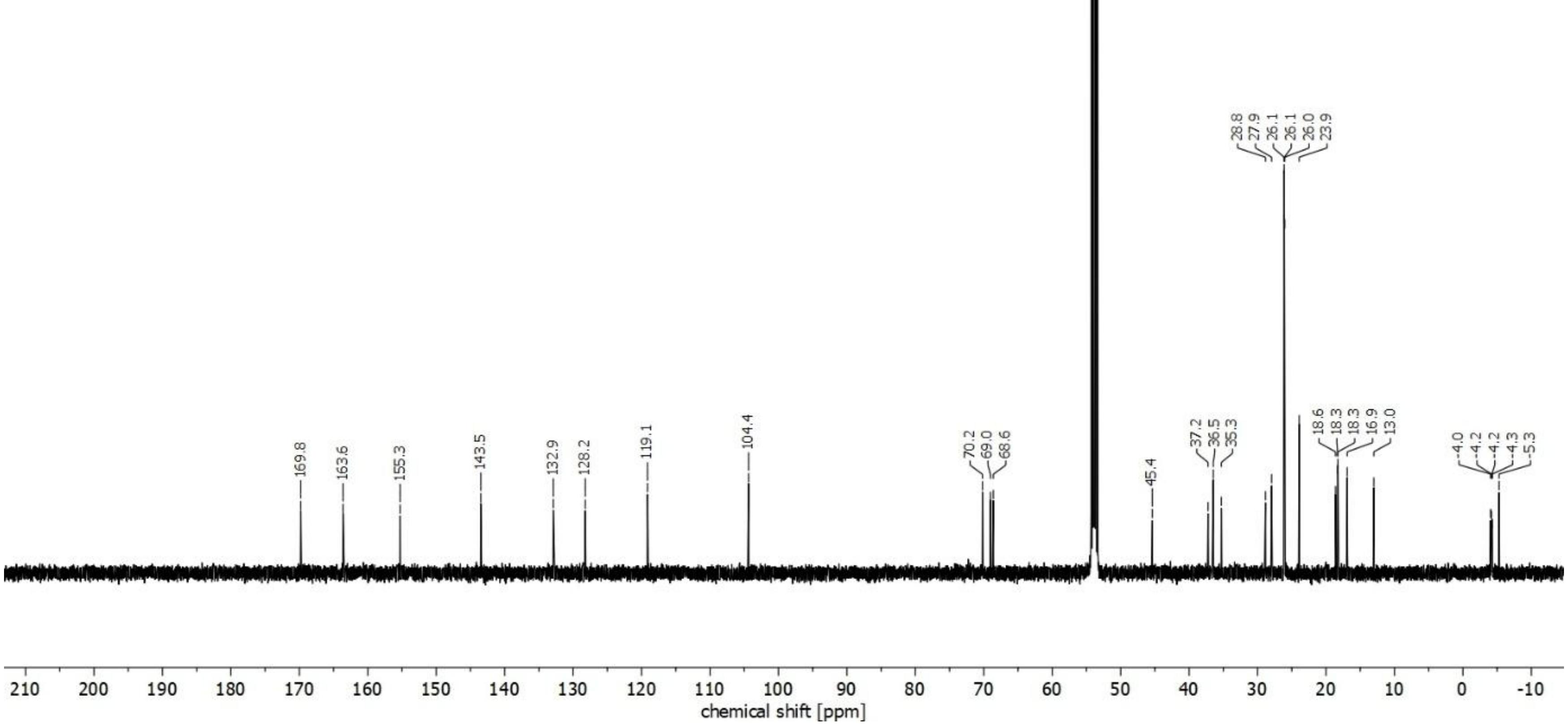

Nucleus: ^{1}H

Frequency: 400.13 MHz

Solvent: CD_2Cl_2

Temperature: 298.0 K

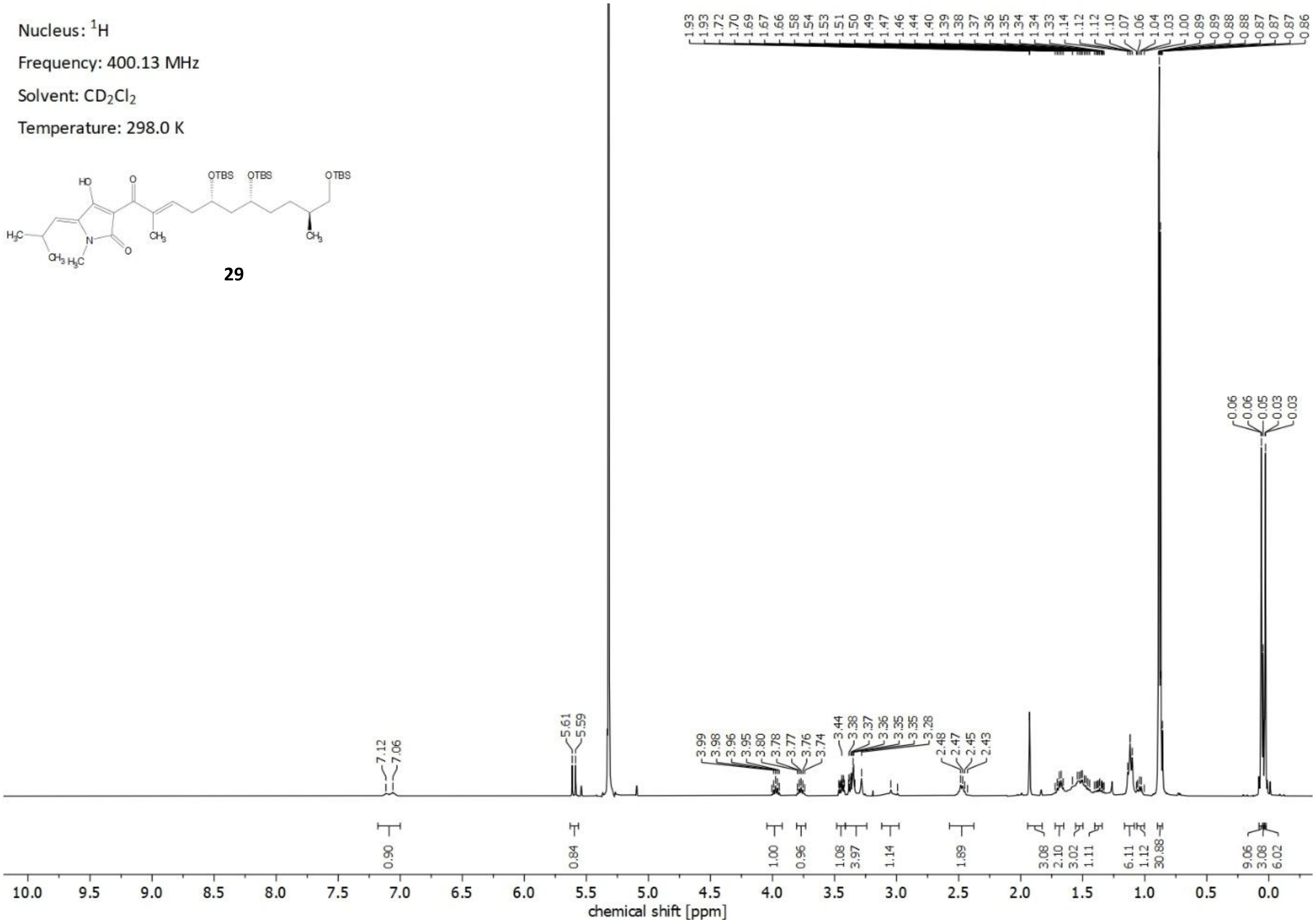

29

Nucleus: ^{13}C

Frequency: 176.14 MHz

Solvent: CD_2Cl_2

Temperature: 298.0 K

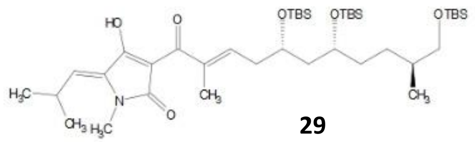

29

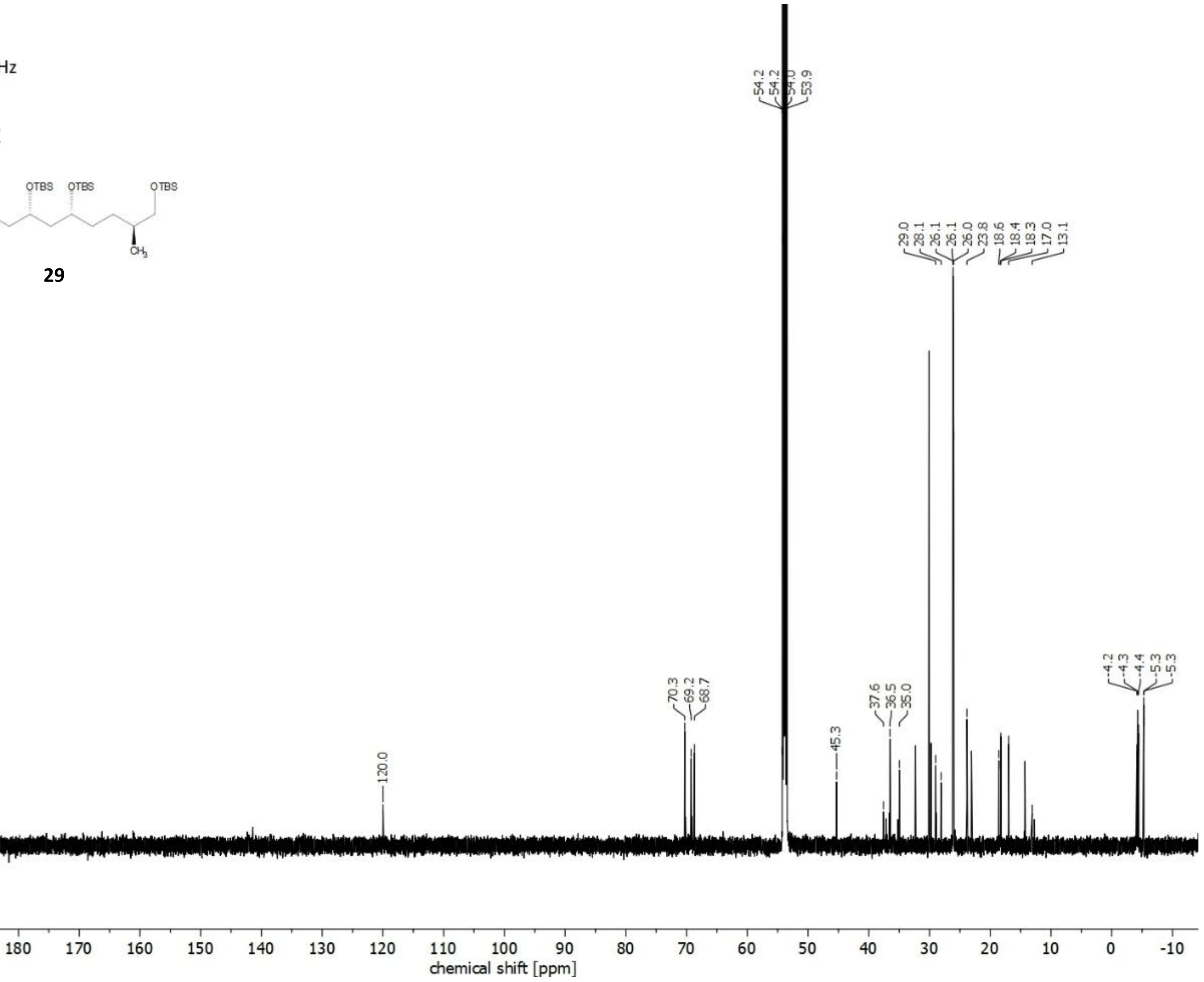

Nucleus: ^{1}H

Frequency: 700.41 MHz

Solvent: MeOD

Temperature: 298.0 K

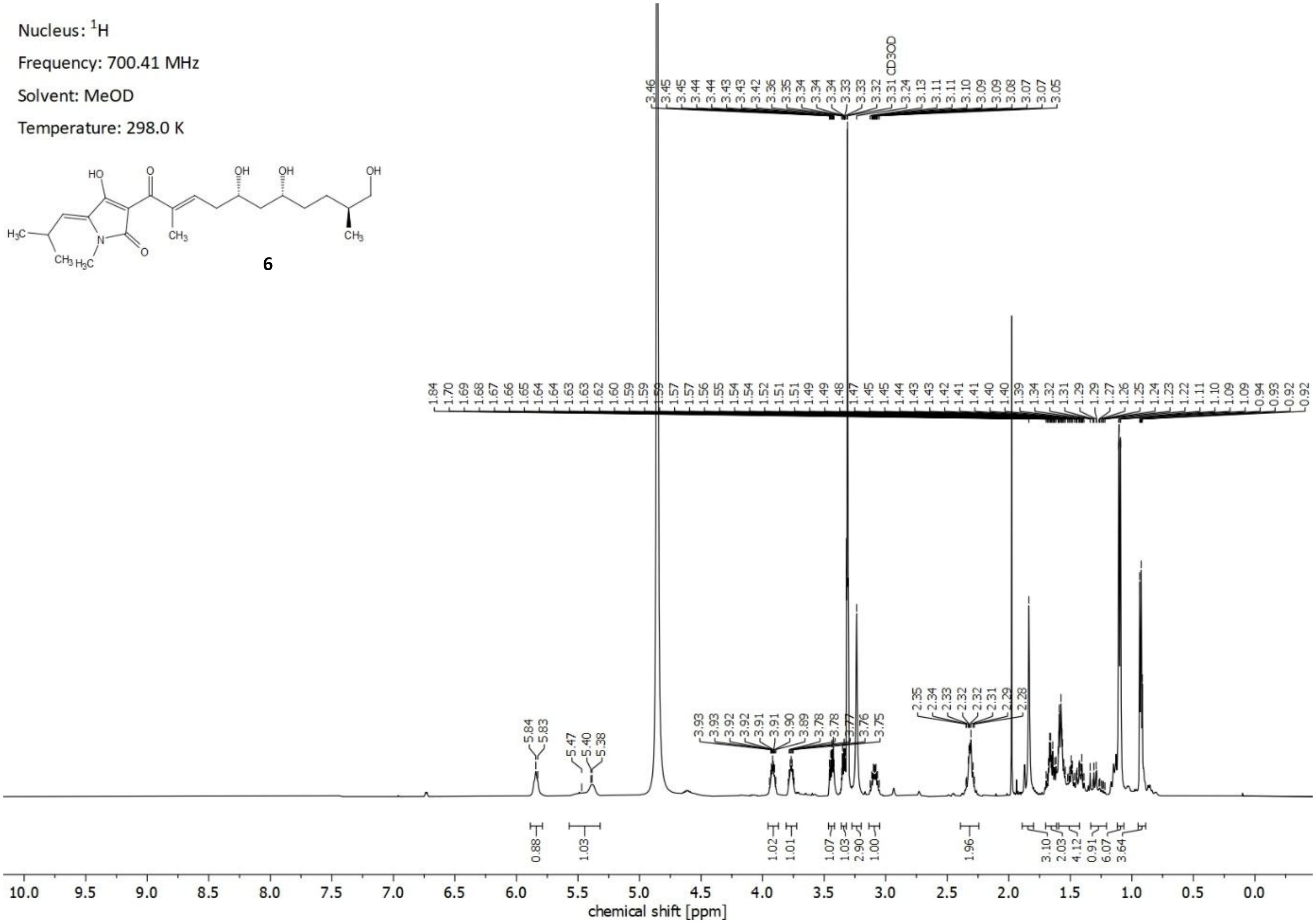

6

Nucleus: ^{13}C

Frequency: 176.14 MHz

Solvent: MeOD

Temperature: 298.0 K

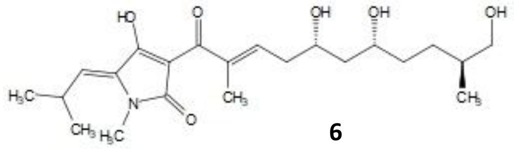

6

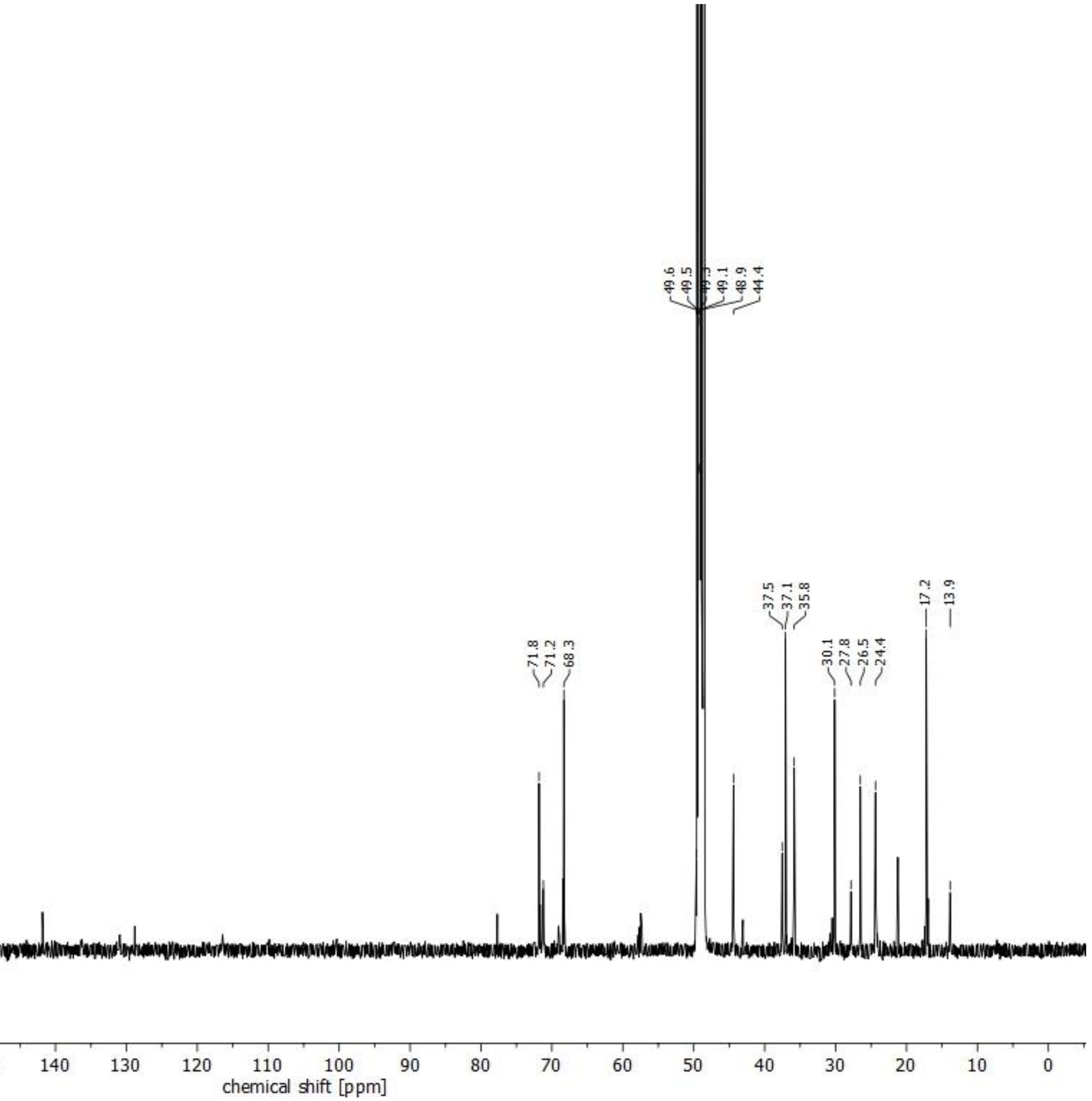

Synthesis of Eastern Fragment 8

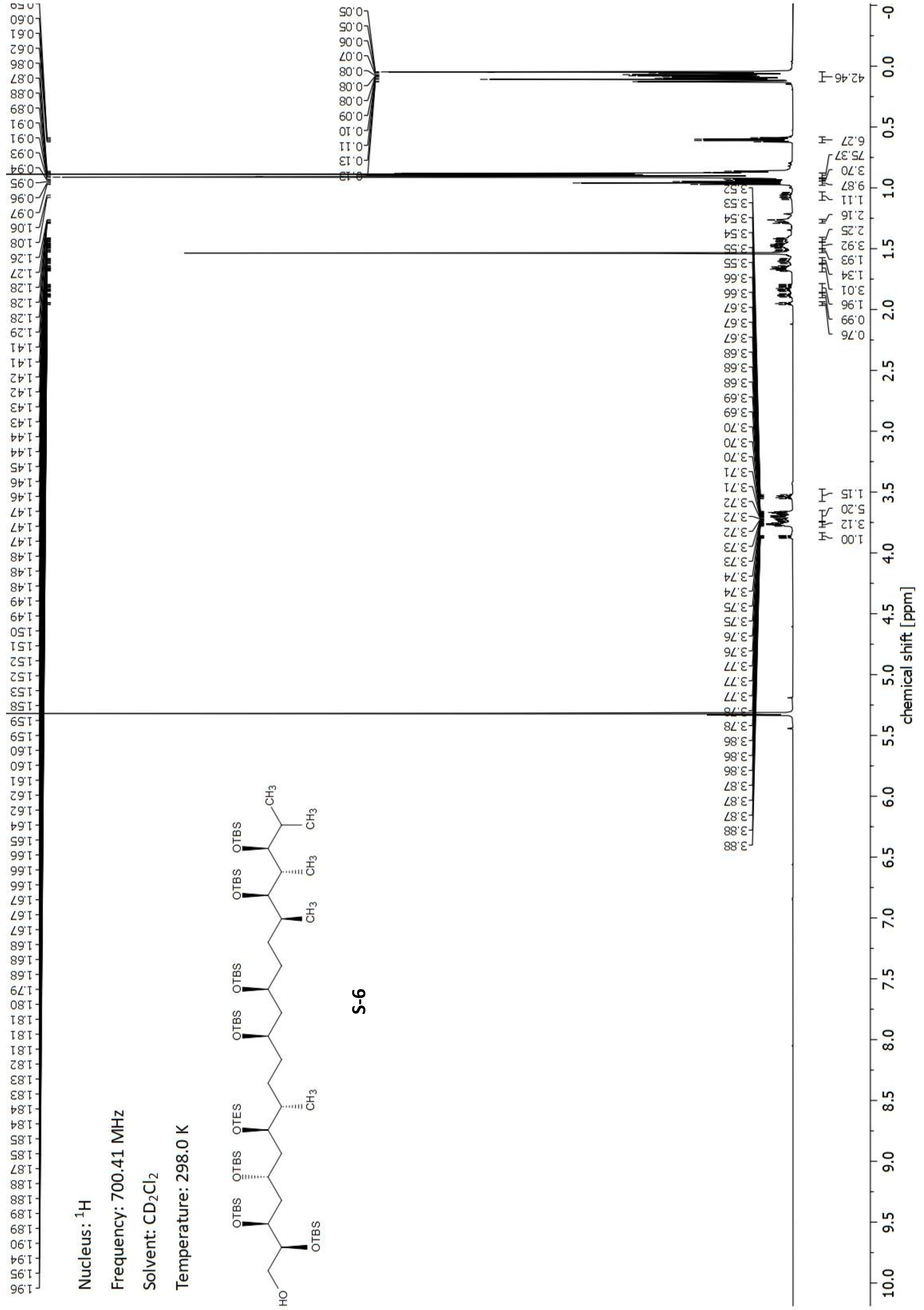

S-6

Nucleus: ^{13}C

Frequency: 176.14 MHz

Solvent: CD_2Cl_2

Temperature: 298.0 K

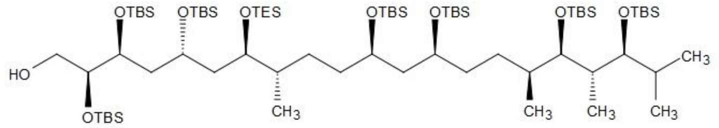

S-6

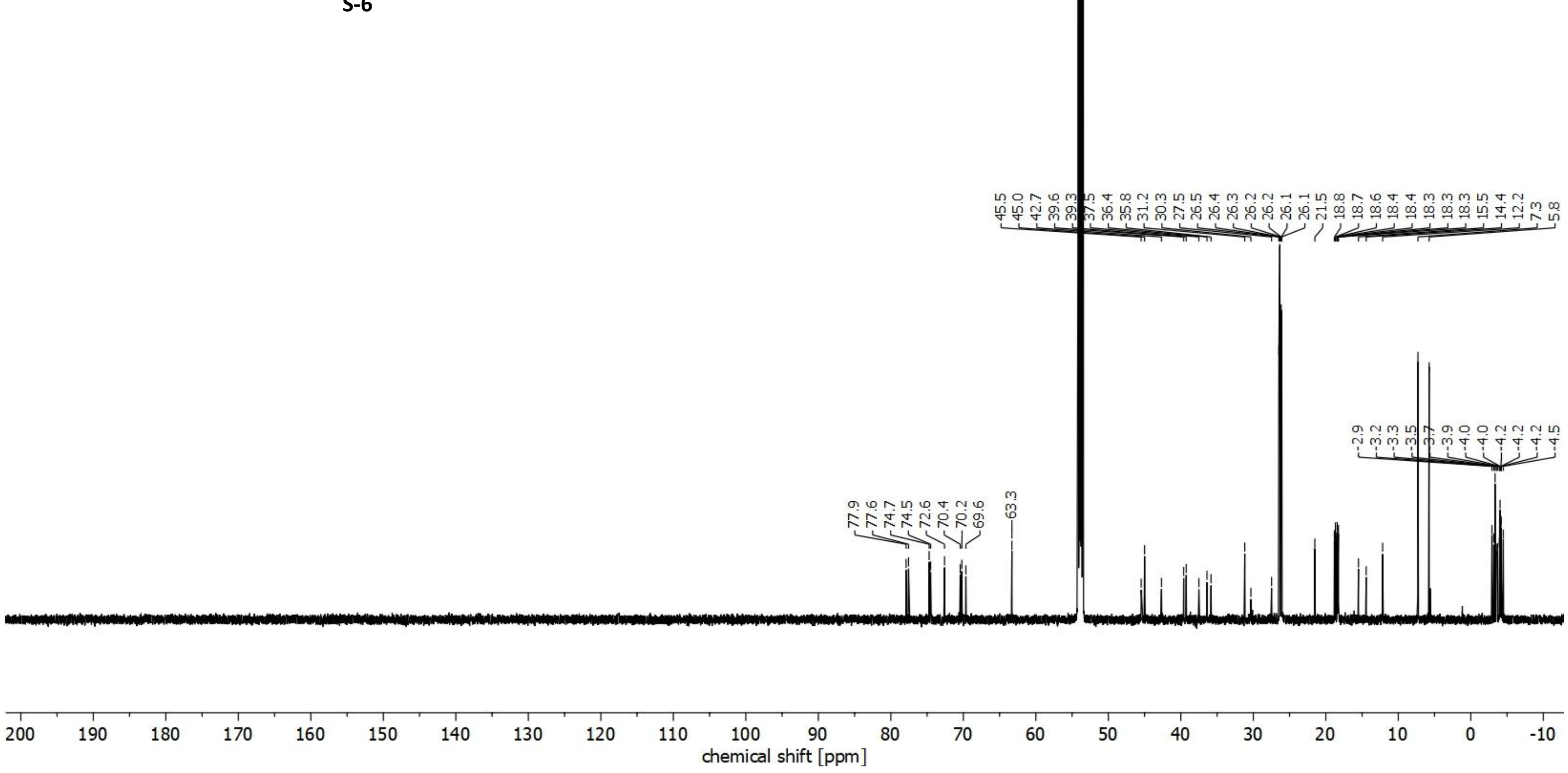

Nucleus: 1H-NMR,
Frequency: 700.41 MHz,
Solvent: MeOD

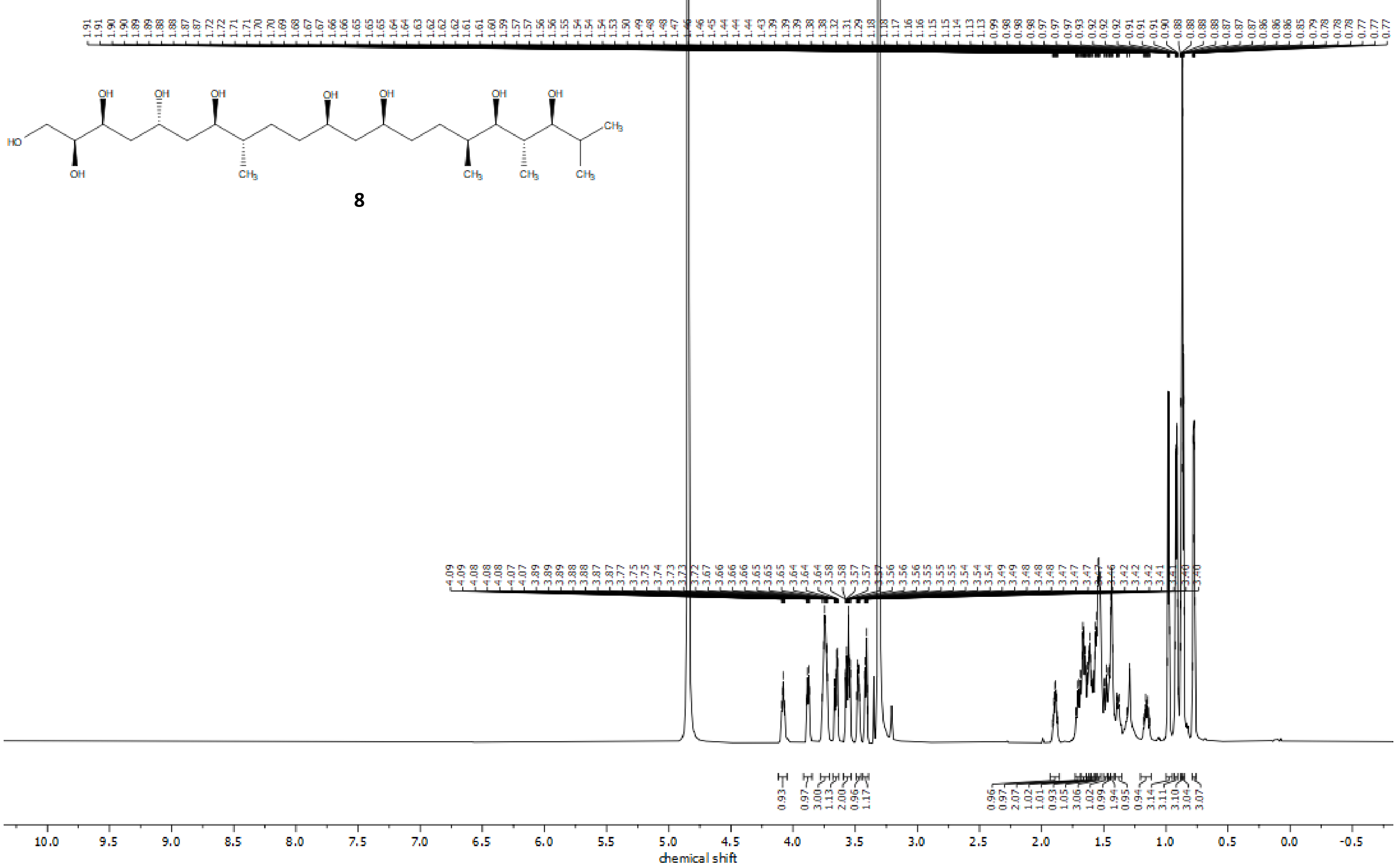

Nucleus: 13C-NMR,
Frequency: 176.14 MHz,
Solvent: MeOD

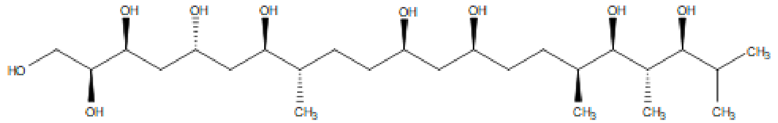

8

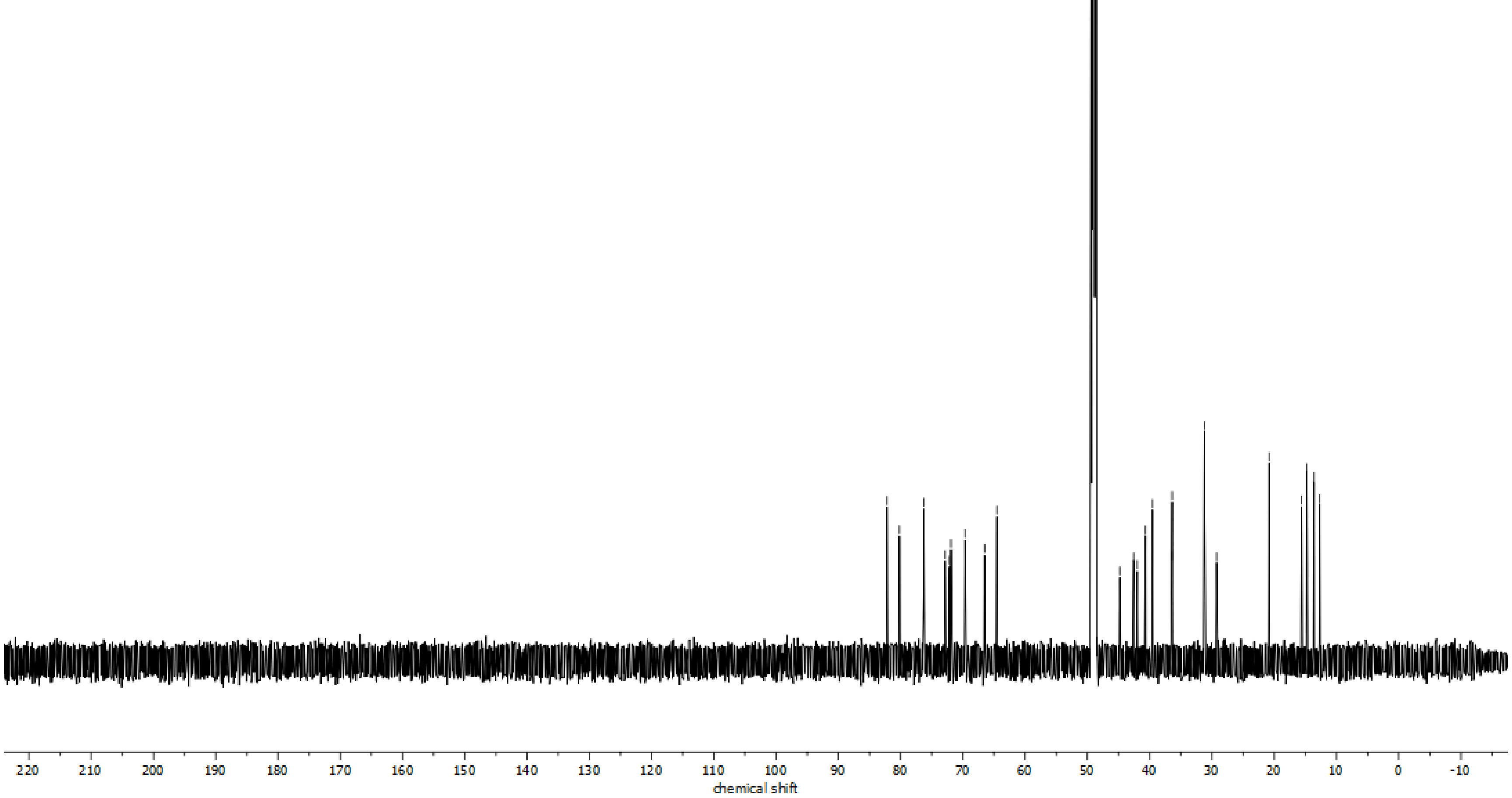

Synthesis of Western Fragment 7

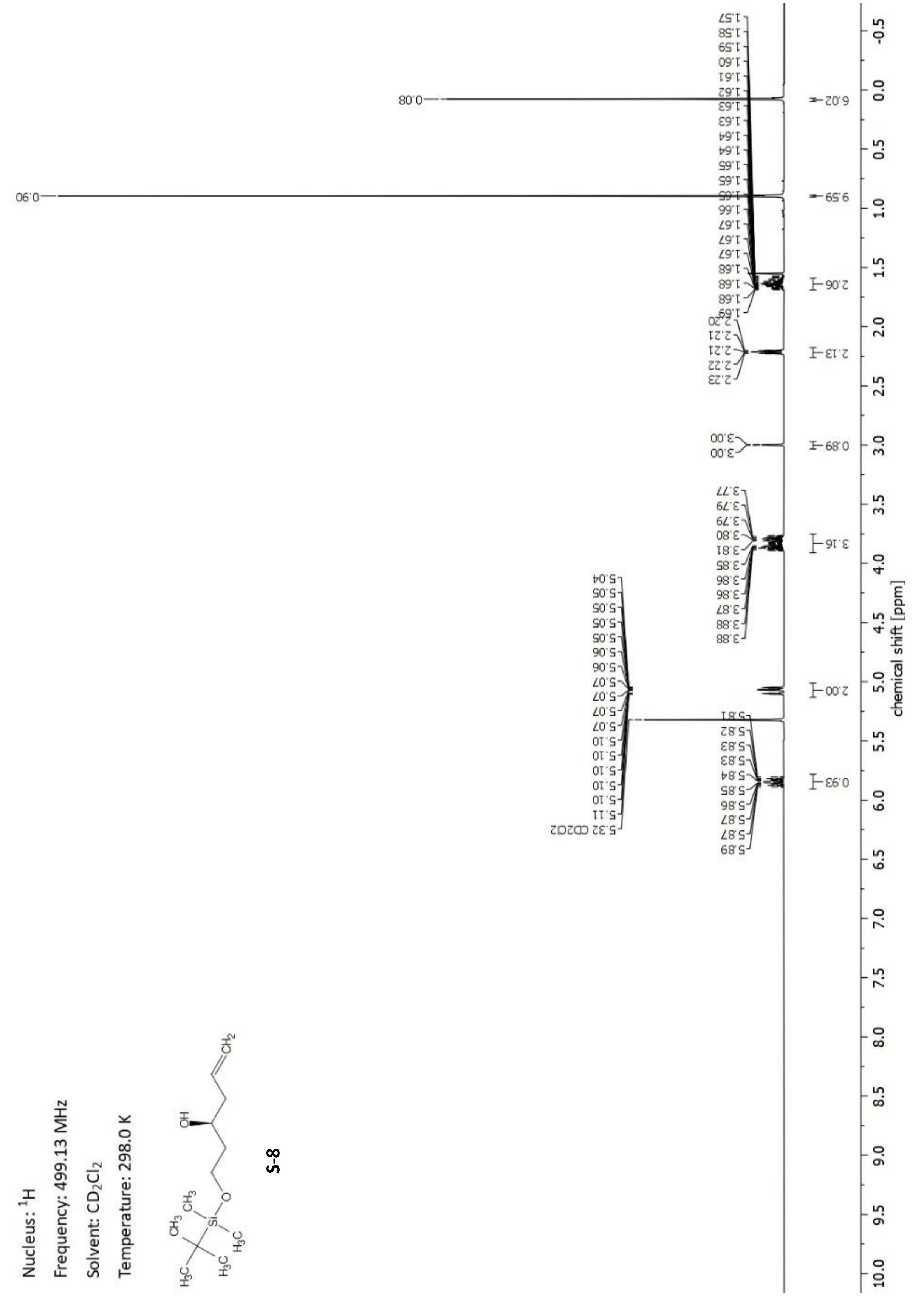

Nucleus: ^{13}C

Frequency: 125.52 MHz

Solvent: CD_2Cl_2

Temperature: 298.0 K

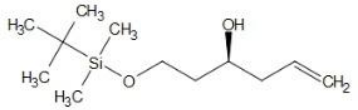

S-8

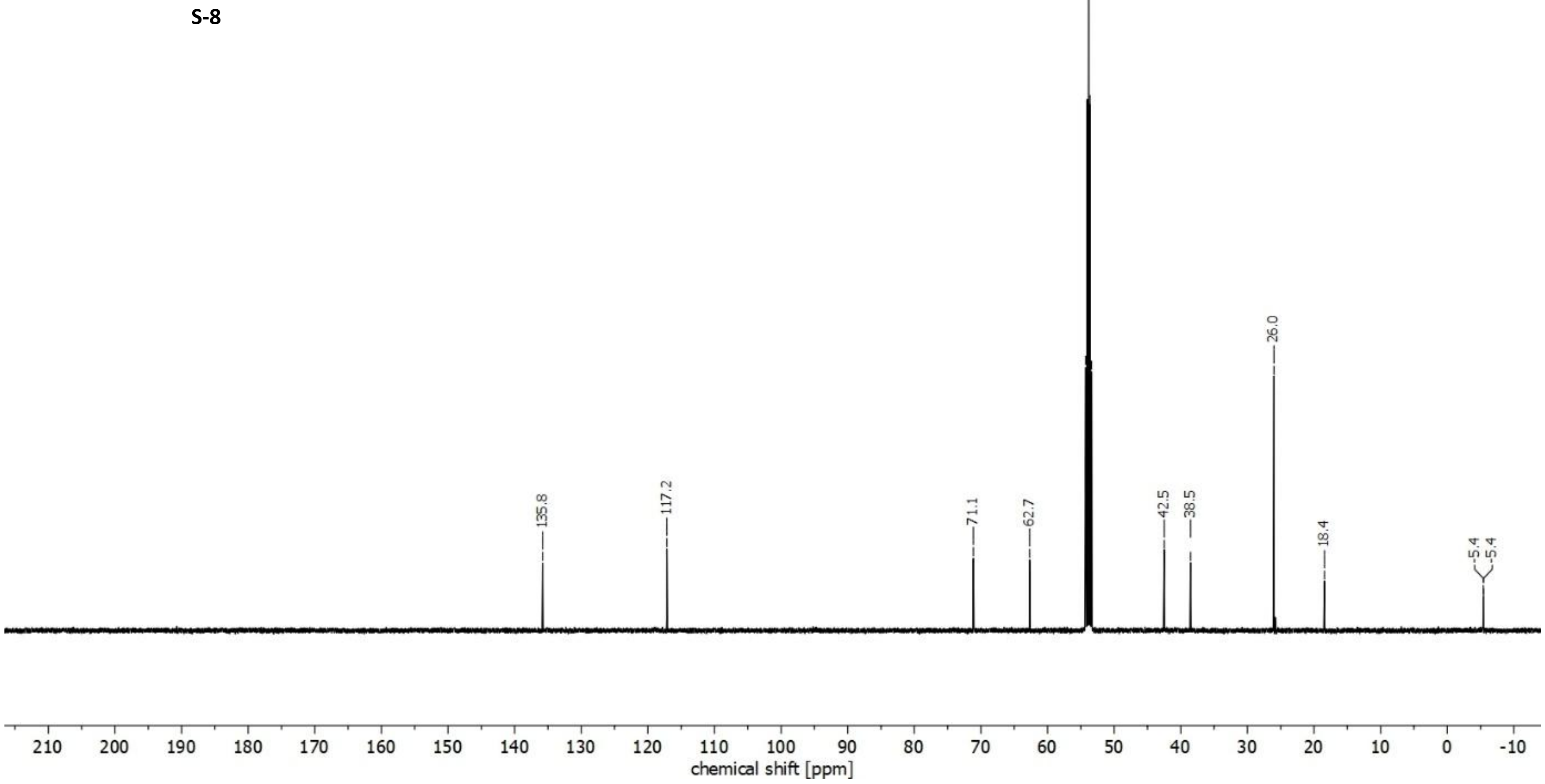

Nucleus: ^{1}H

Frequency: 500.04 MHz

Solvent: CD_2Cl_2

Temperature: 298.0 K

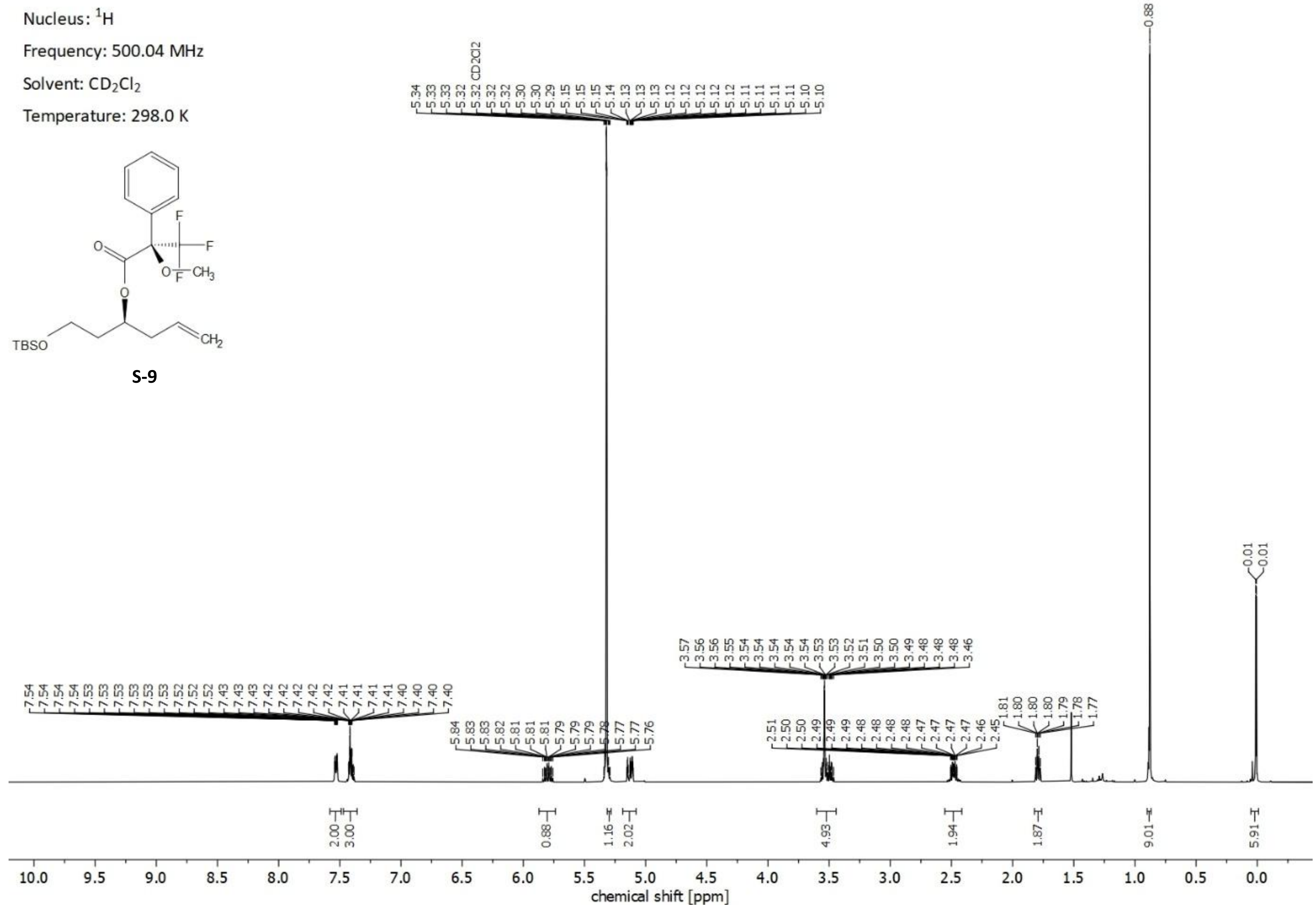

S-9

Nucleus: ^{13}C

Frequency: 125.75 MHz

Solvent: CD_2Cl_2

Temperature: 298.0 K

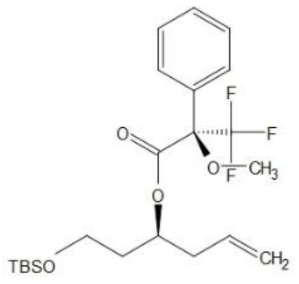

S-9

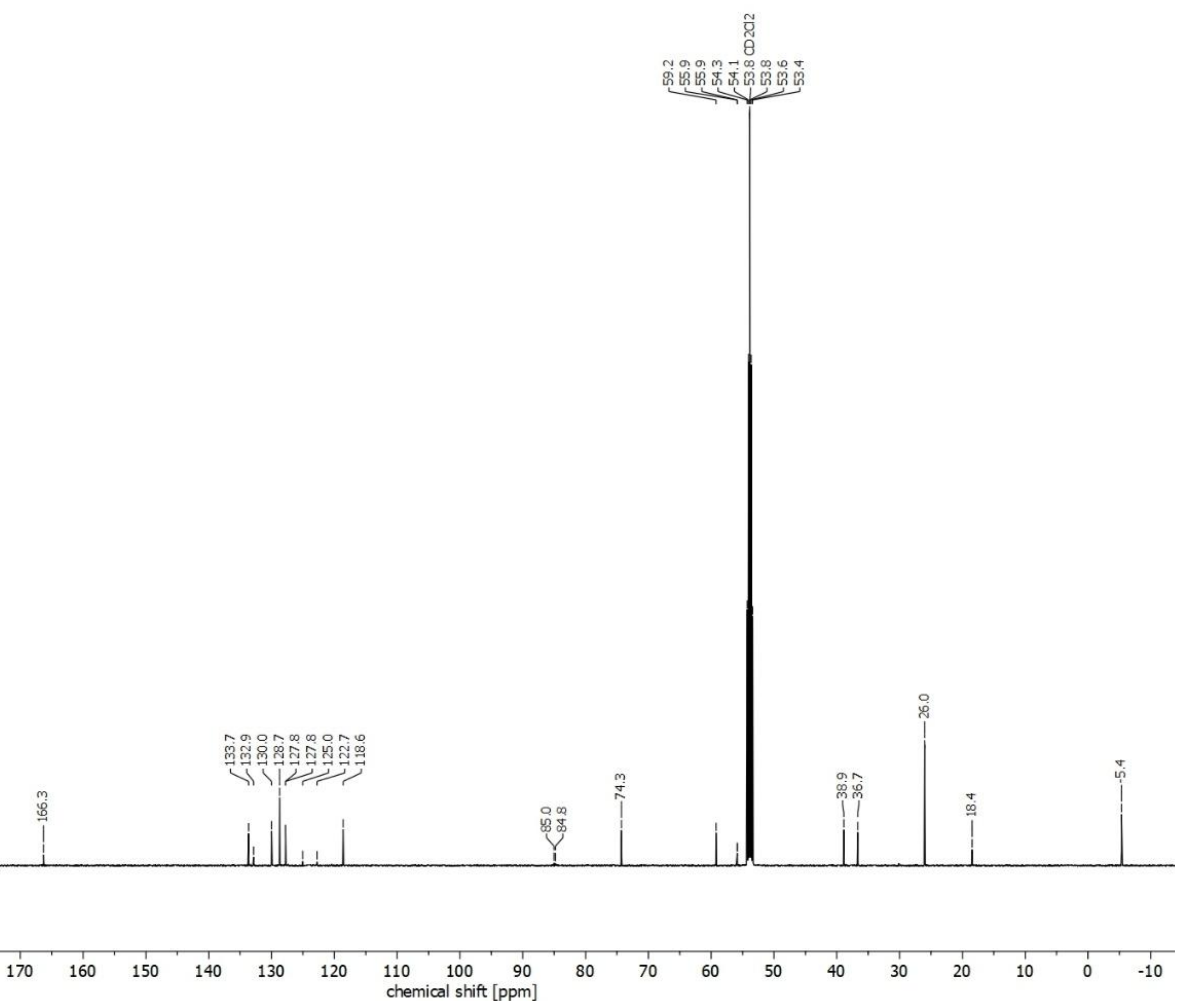

Nucleus: ^{1}H

Frequency: 500.04 MHz

Solvent: CD_2Cl_2

Temperature: 298.0 K

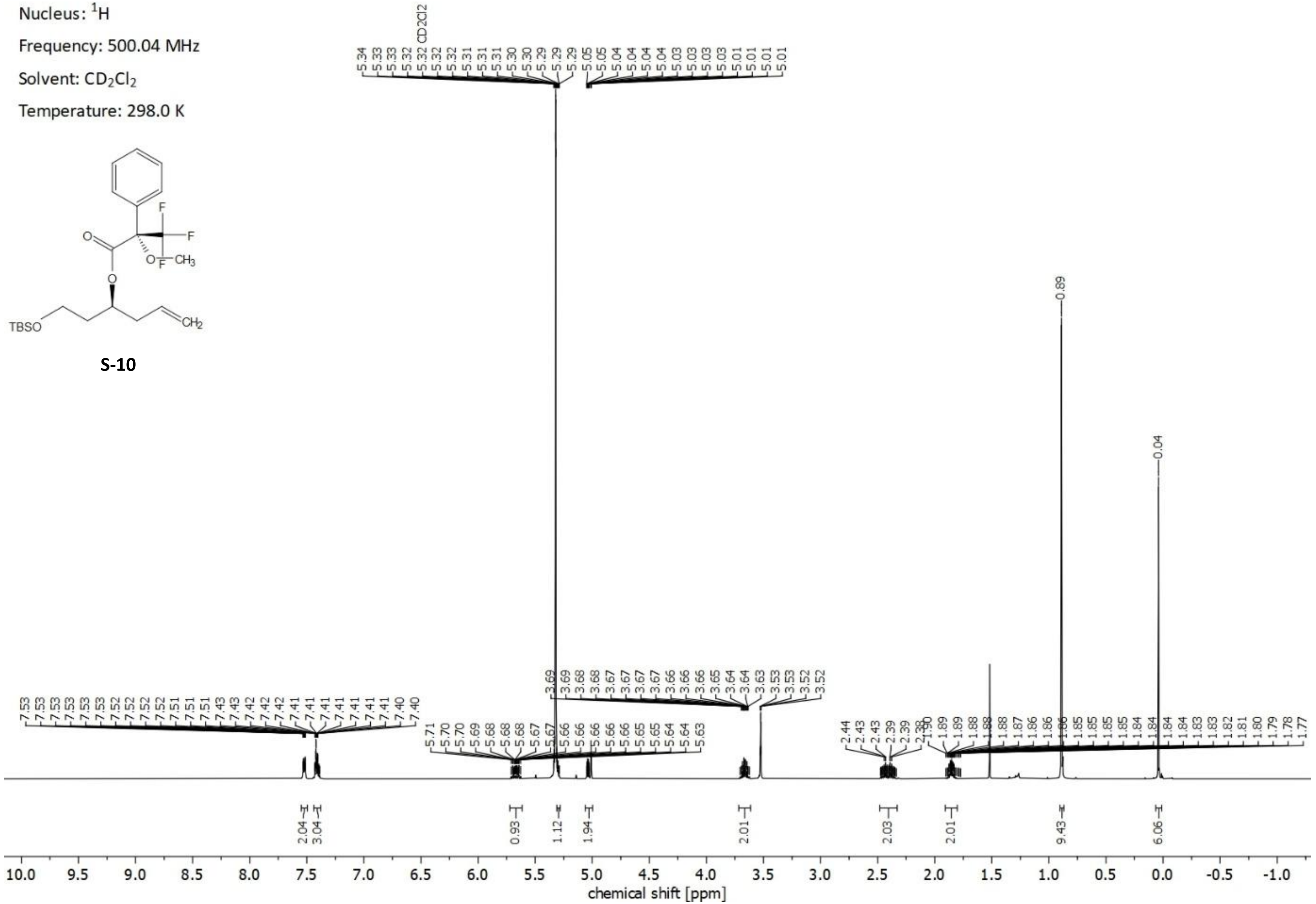

Nucleus: ^{13}C

Frequency: 125.75 MHz

Solvent: CD_2Cl_2

Temperature: 298.0 K

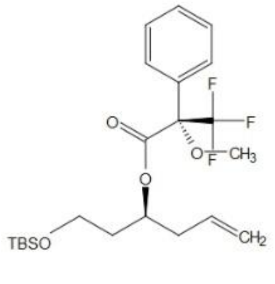

S-10

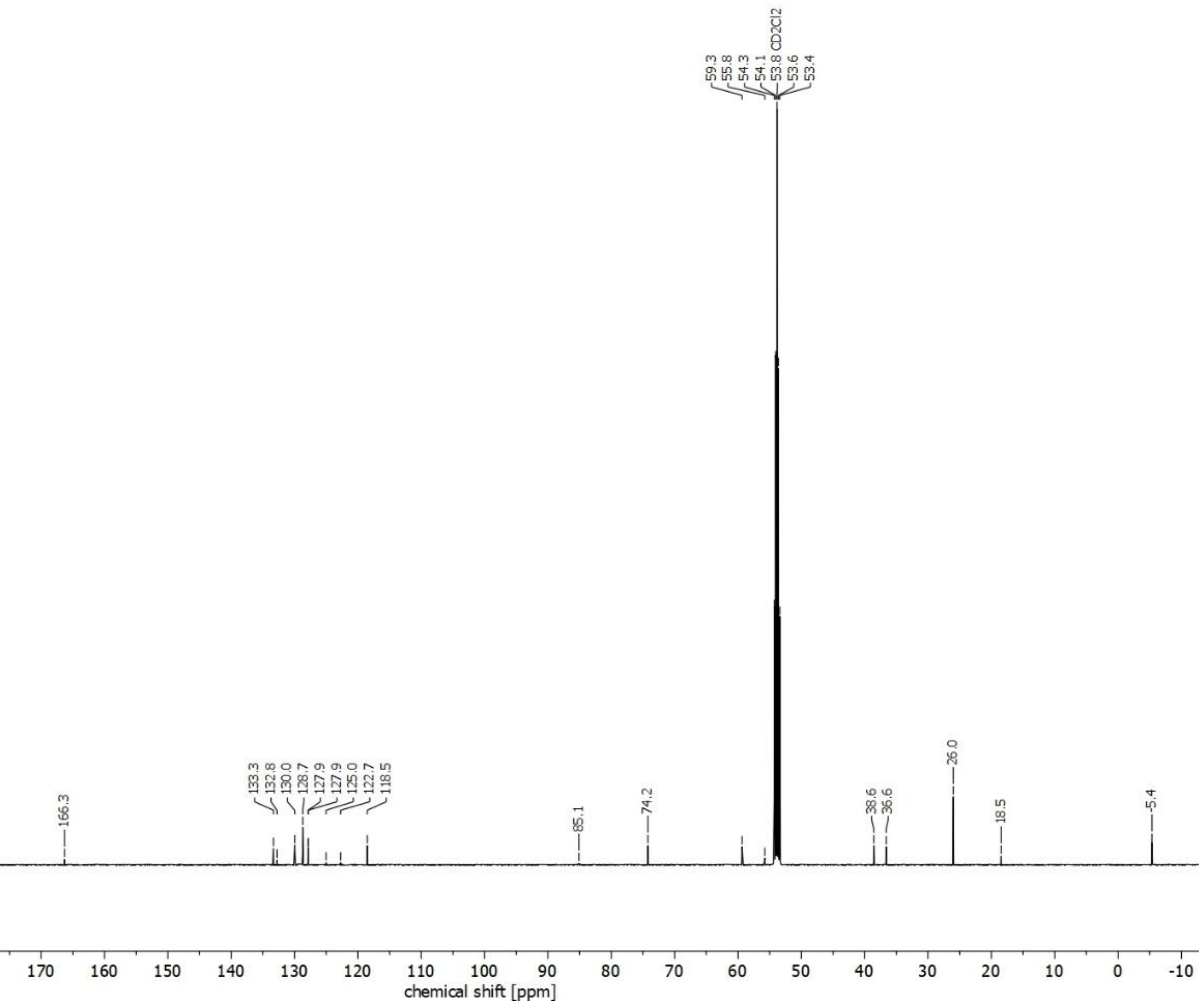

Nucleus: ^{1}H

Frequency: 500.04 MHz

Solvent: CD_2Cl_2

Temperature: 298.0 K

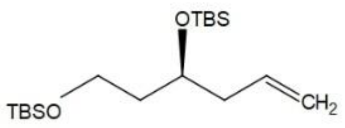

S-11

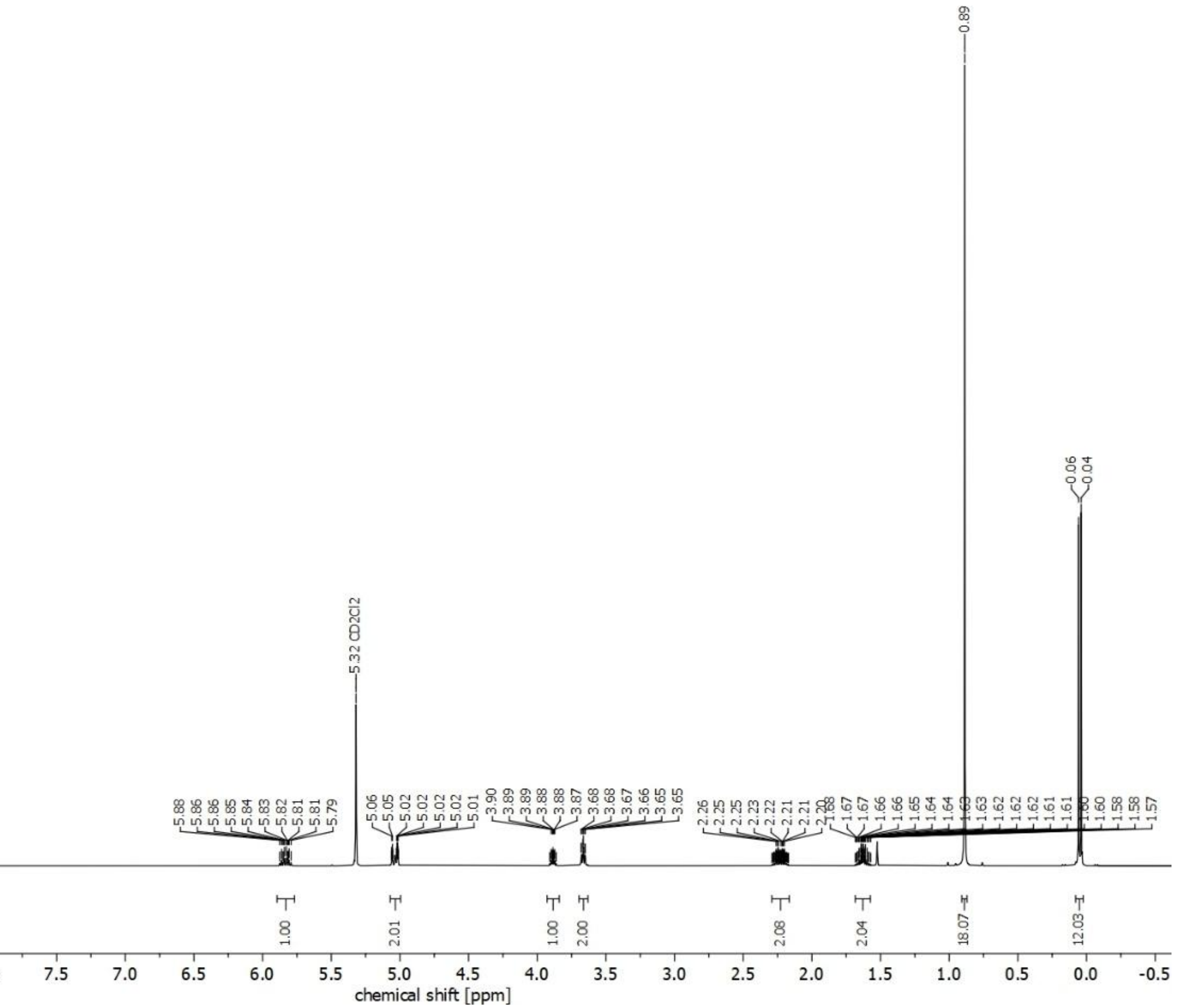

Nucleus: ^{13}C

Frequency: 125.75 MHz

Solvent: CD_2Cl_2

Temperature: 298.0 K

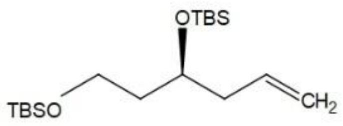

S-11

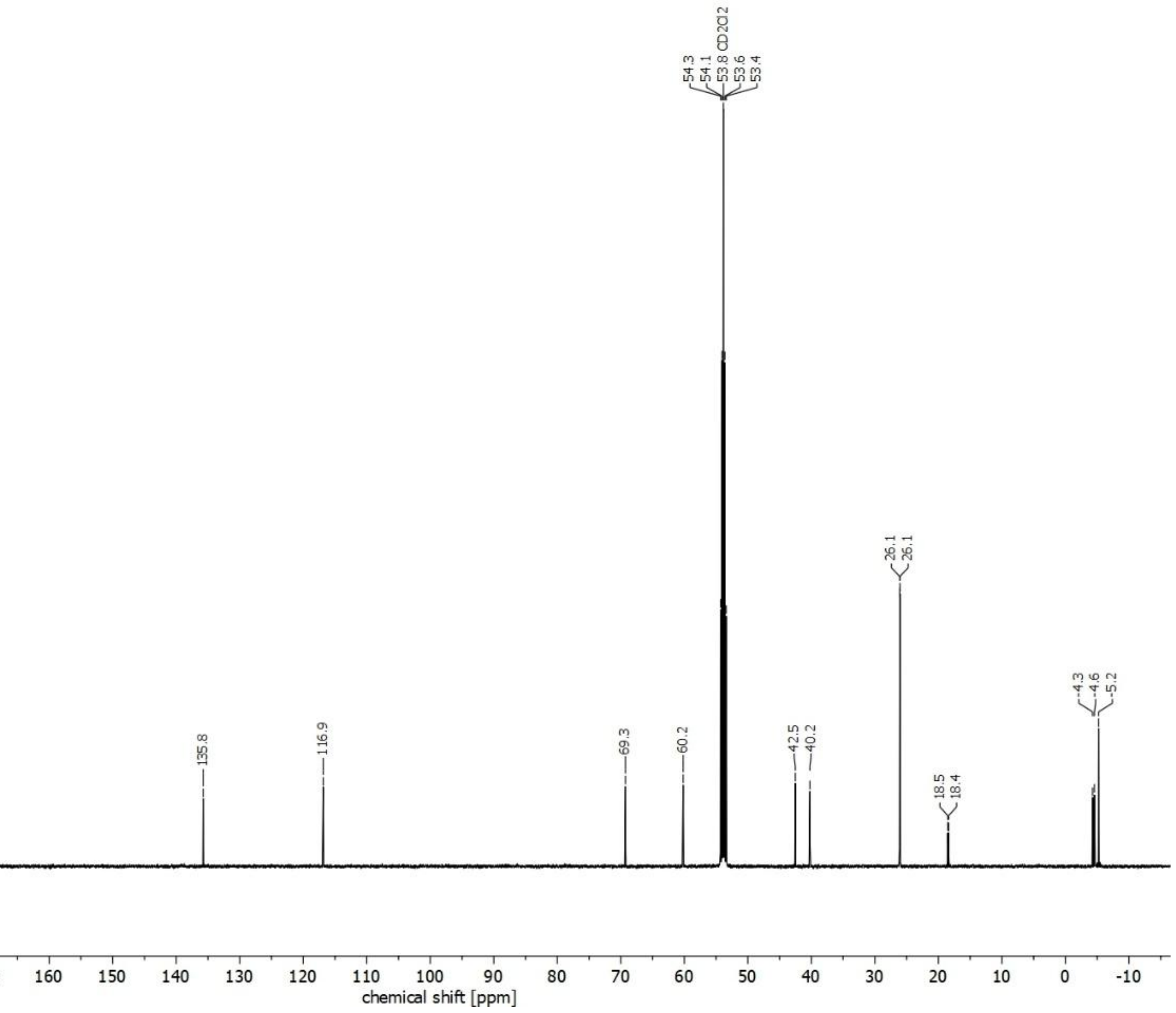

Nucleus: ^{1}H

Frequency: 700.41 MHz

Solvent: CD_2Cl_2

Temperature: 298.0 K

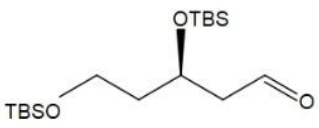

S-12

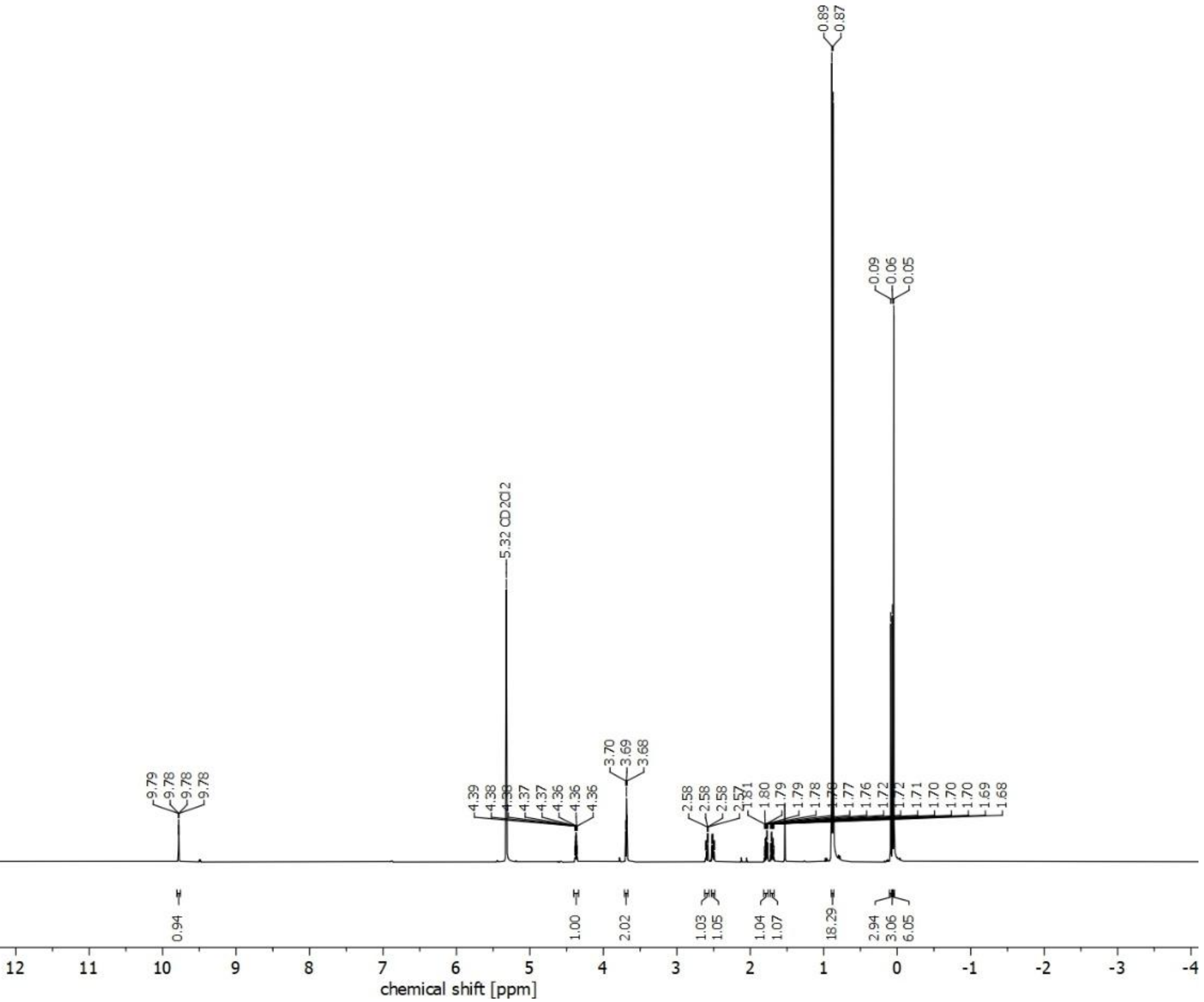

Nucleus: ^{13}C

Frequency: 176.14 MHz

Solvent: CD_2Cl_2

Temperature: 298.0 K

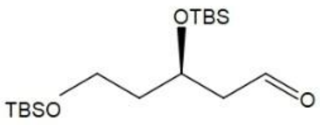

S-12

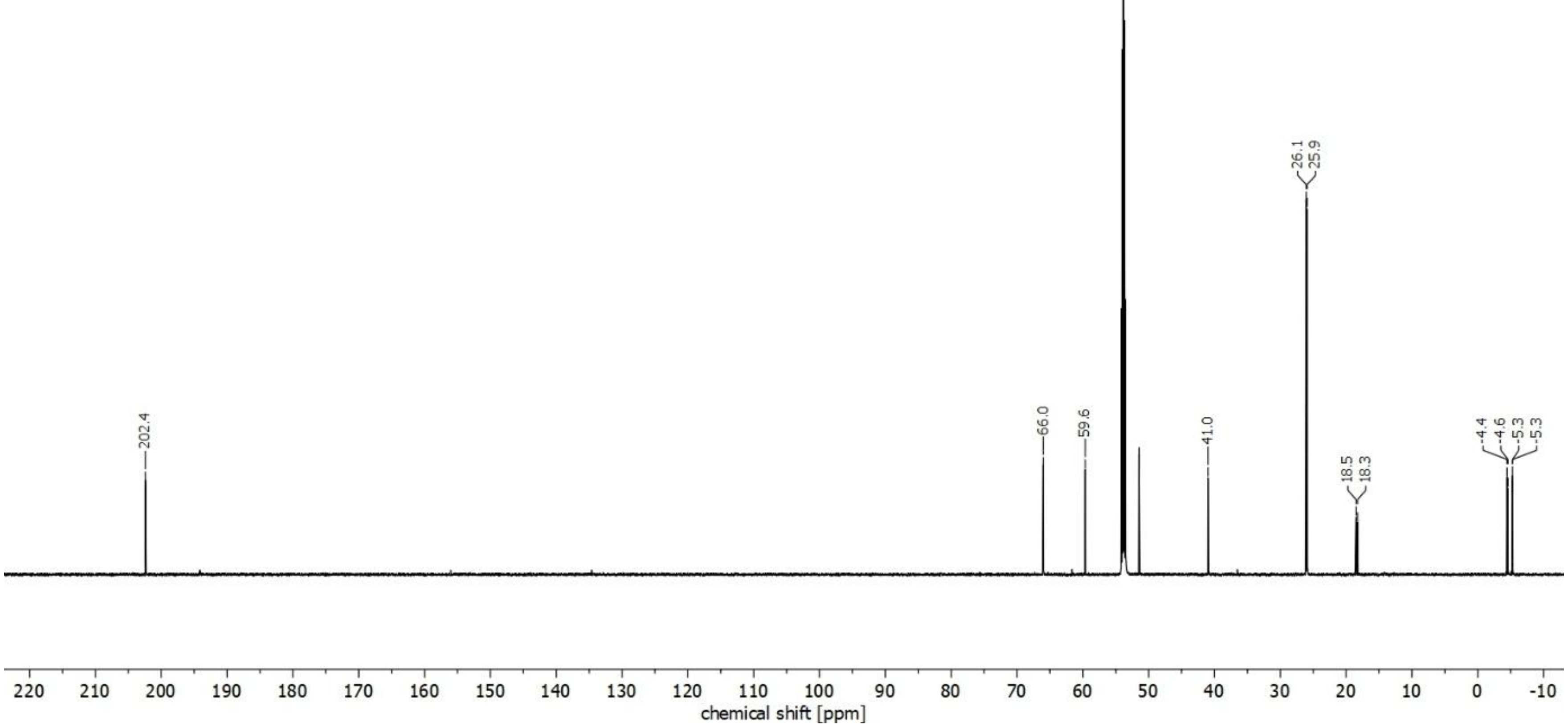

Nucleus: ^{1}H

Frequency: 500.04 MHz

Solvent: CD_2Cl_2

Temperature: 298.0 K

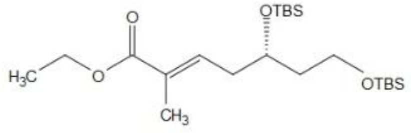

S-13

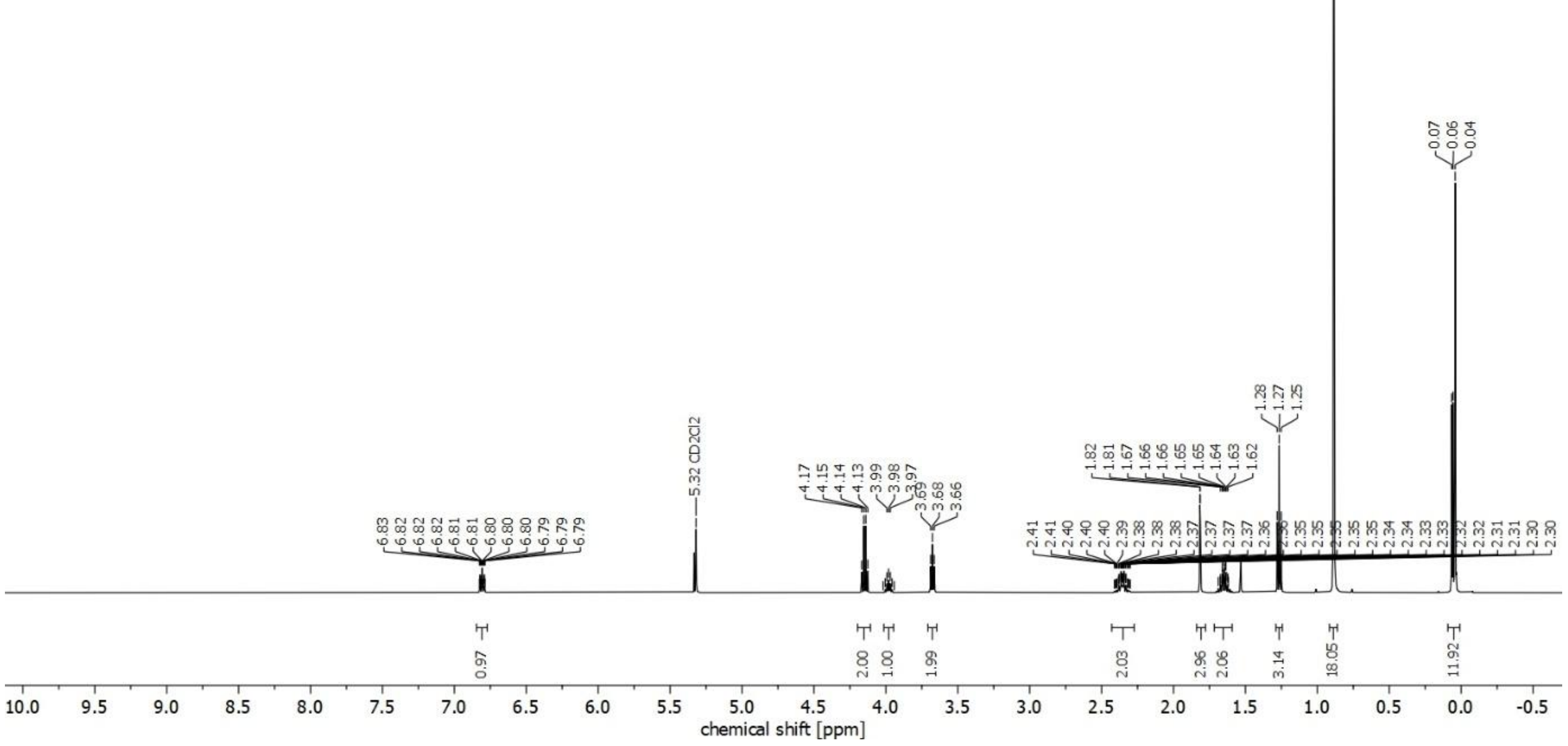

Nucleus: ^{13}C

Frequency: 125.75 MHz

Solvent: CD_2Cl_2

Temperature: 298.0 K

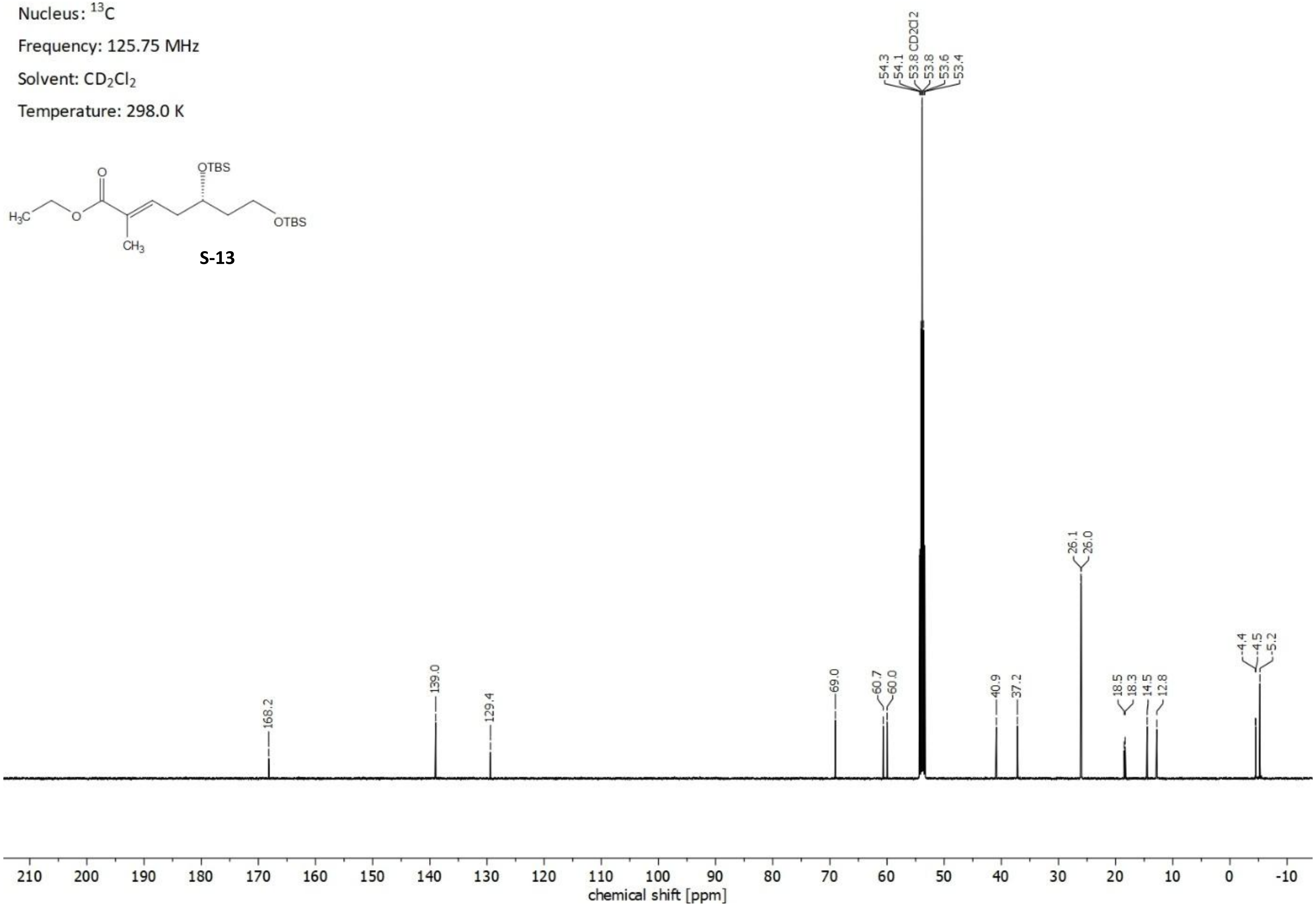

Nucleus: ^{1}H

Frequency: 500.04 MHz

Solvent: CD_2Cl_2

Temperature: 298.0 K

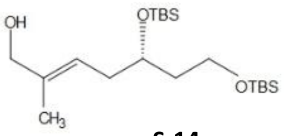

S-14

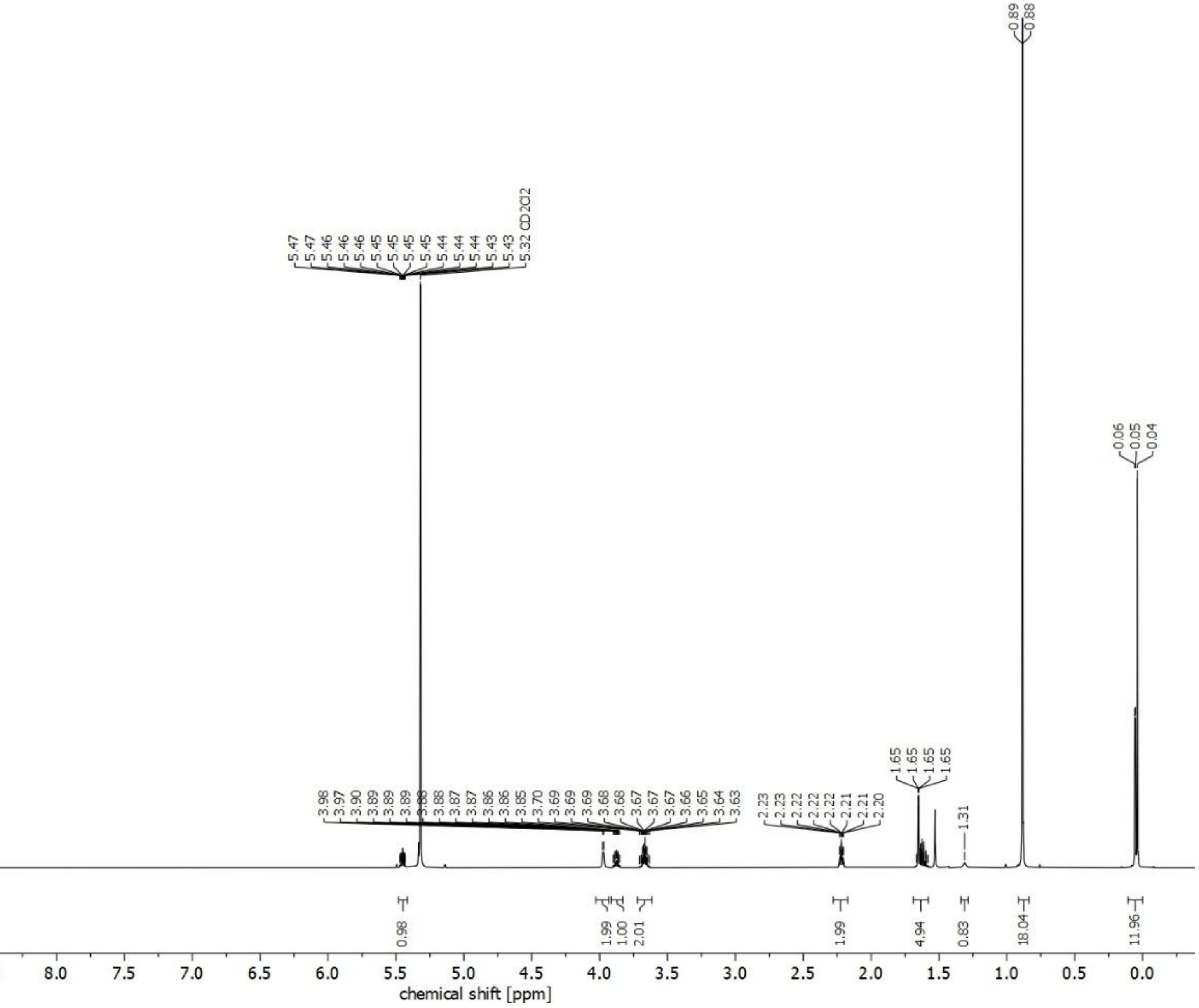

Nucleus: ^{13}C

Frequency: 125.75 MHz

Solvent: CD_2Cl_2

Temperature: 298.0 K

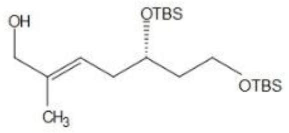

S-14

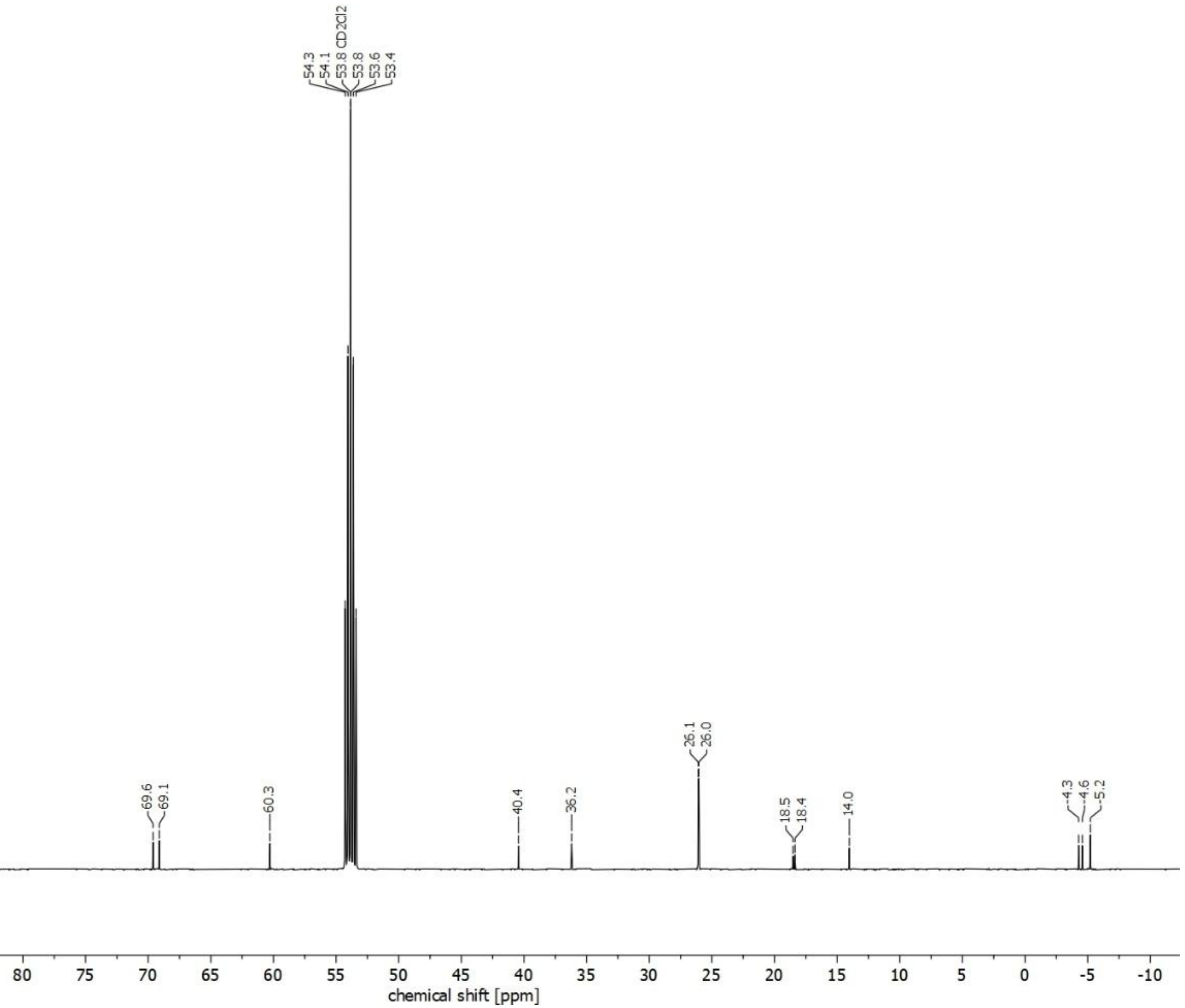

Nucleus: ^{1}H

Frequency: 500.04 MHz

Solvent: CD_2Cl_2

Temperature: 298.0 K

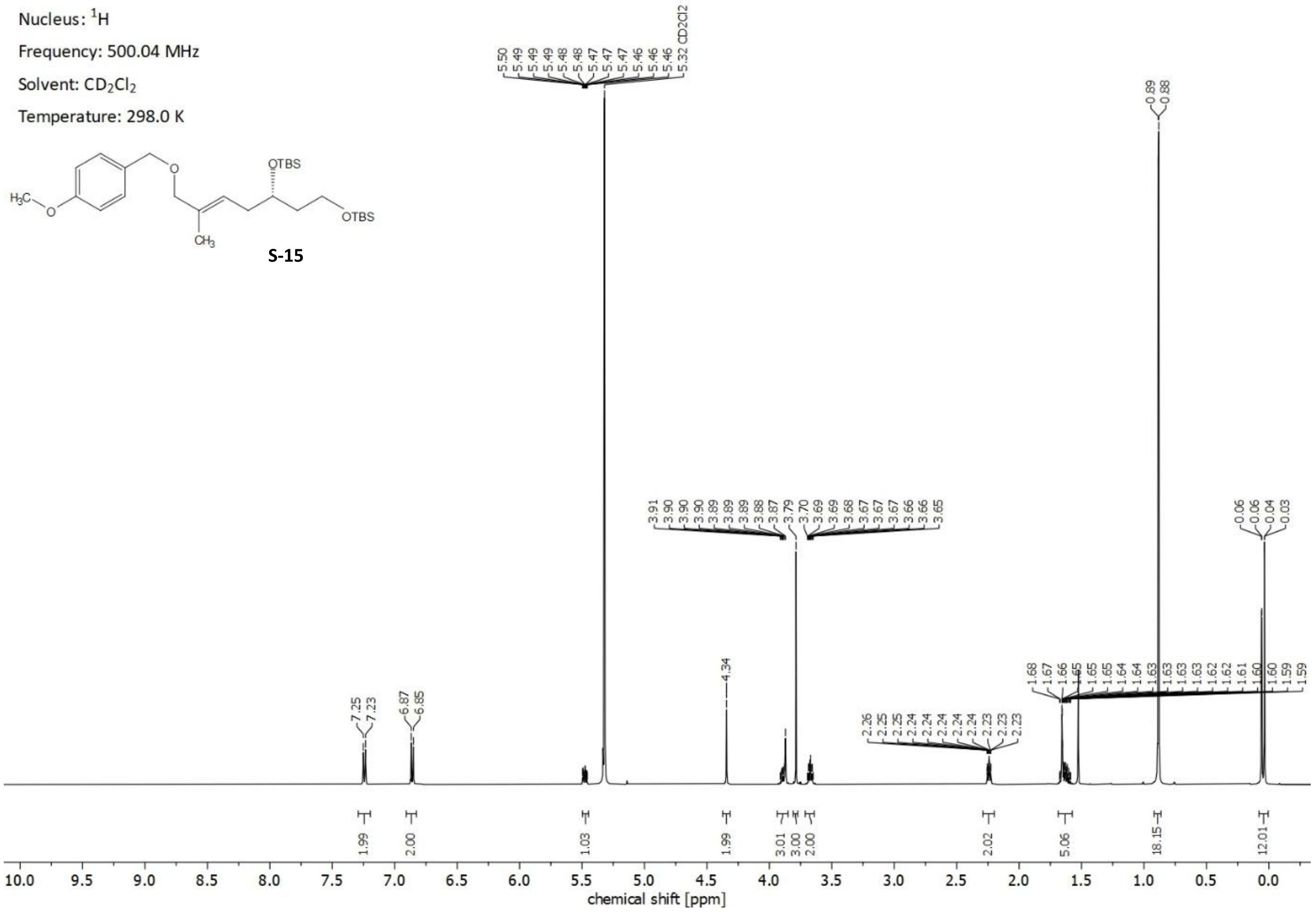

S-15

Nucleus: ^{13}C

Frequency: 125.75 MHz

Solvent: CD_2Cl_2

Temperature: 298.0 K

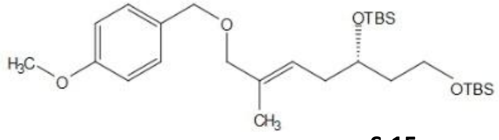

S-15

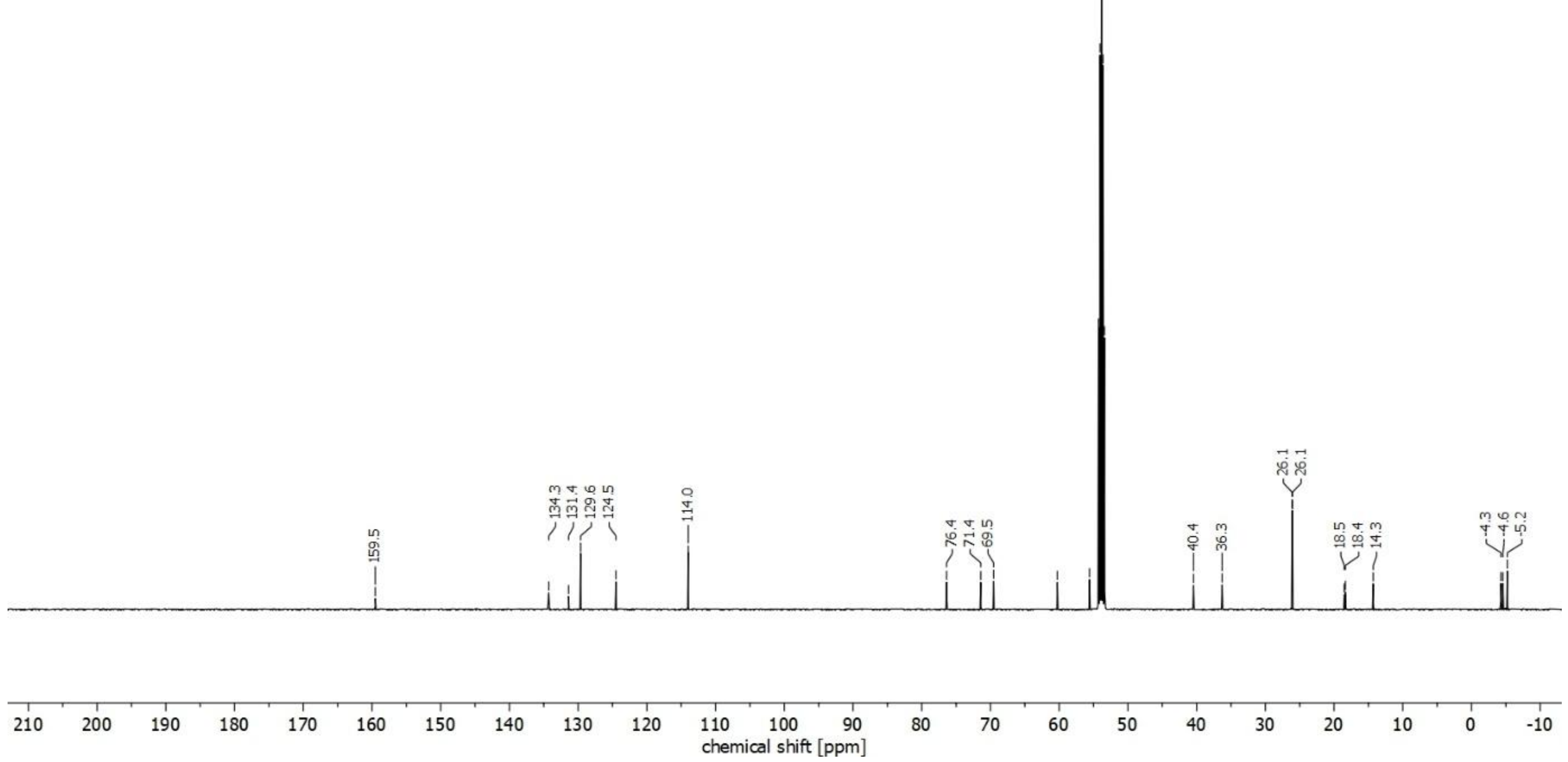

Nucleus: ^{1}H

Frequency: 700.41 MHz

Solvent: CD_2Cl_2

Temperature: 298.0 K

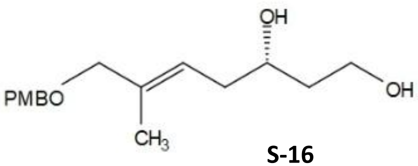

S-16

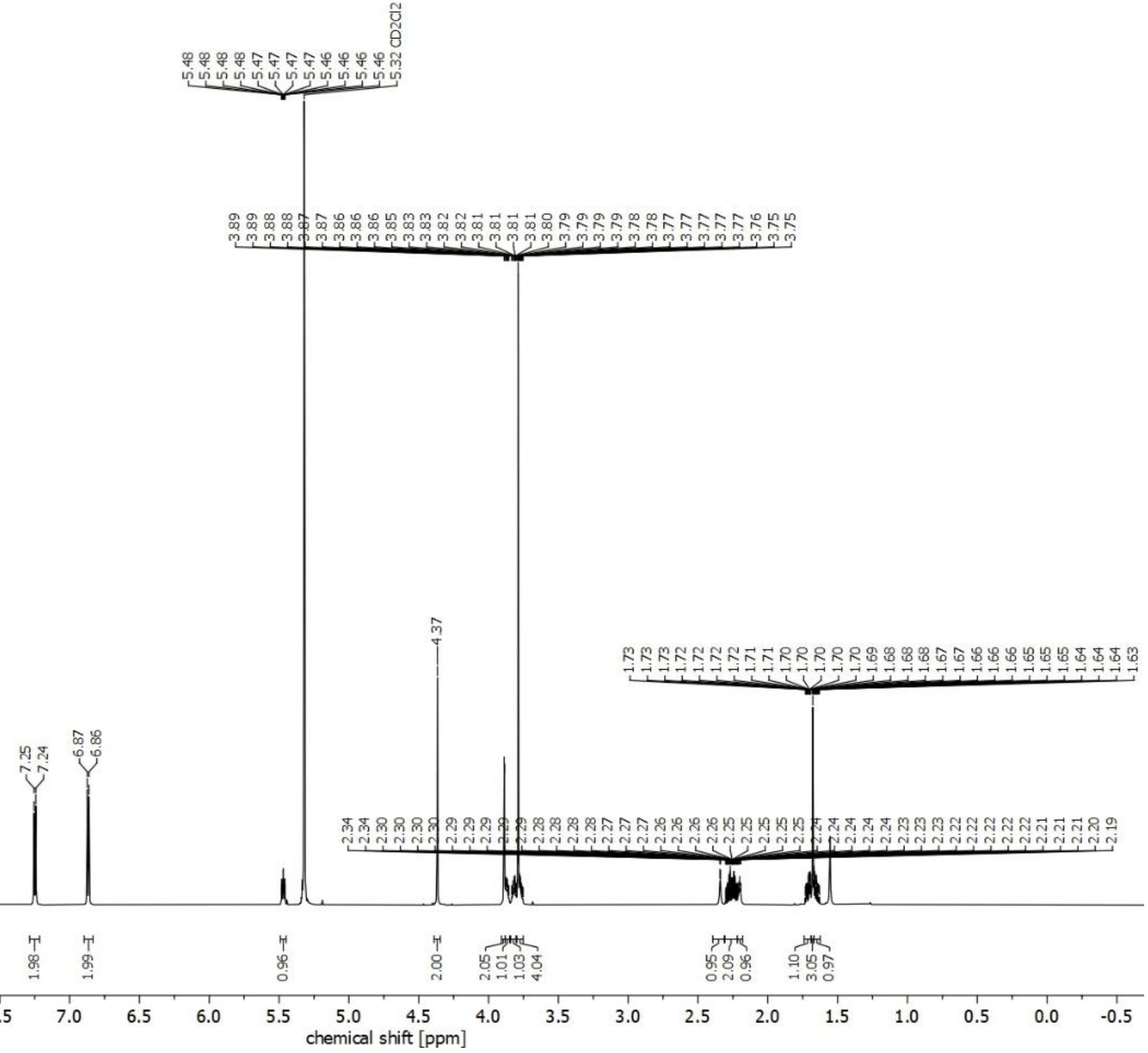

Nucleus: ^{13}C

Frequency: 176.14 MHz

Solvent: CD_2Cl_2

Temperature: 298.0 K

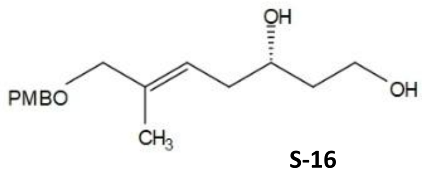

S-16

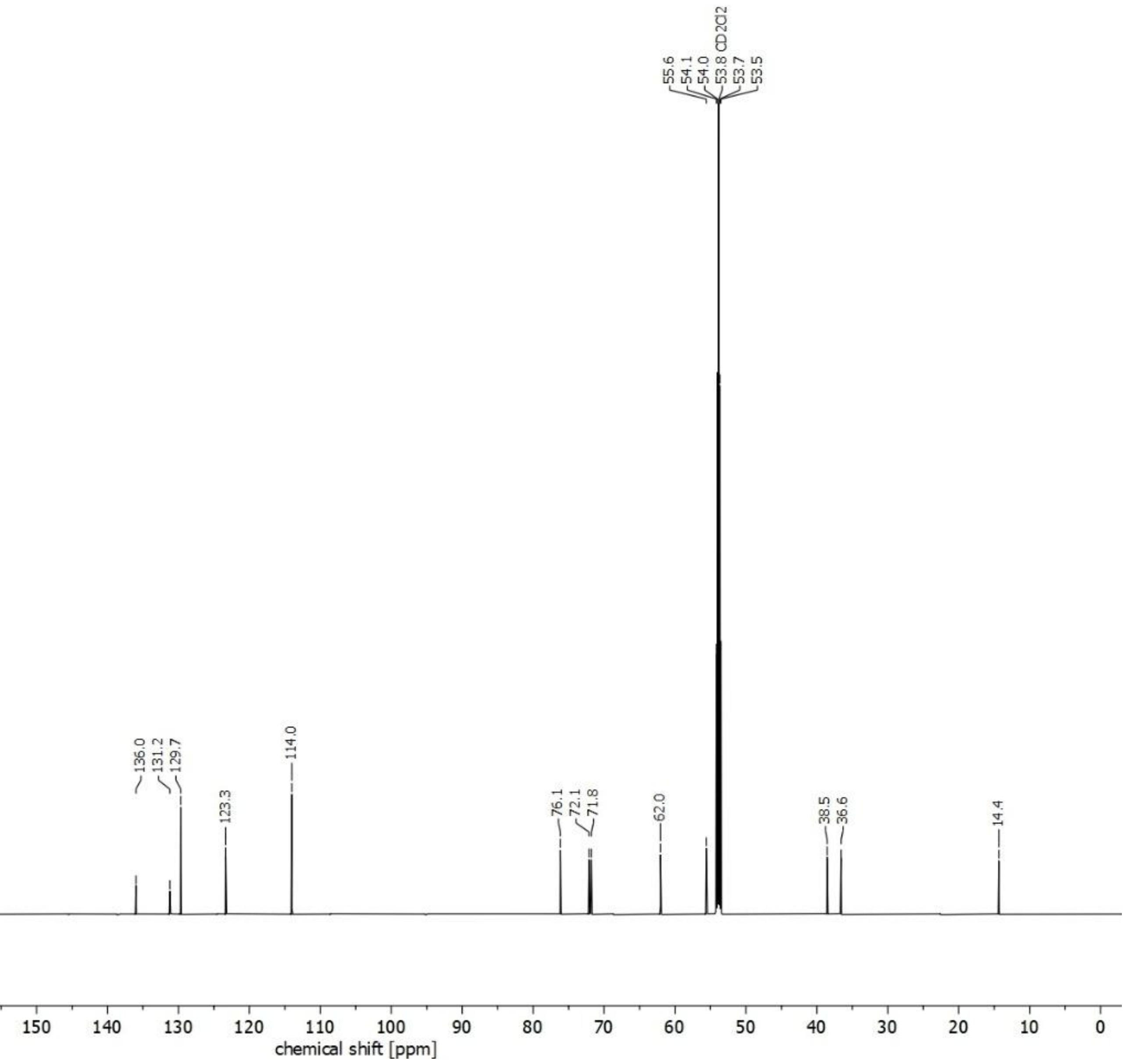

Nucleus: ^{1}H

Frequency: 500.04 MHz

Solvent: CD_2Cl_2

Temperature: 298.0 K

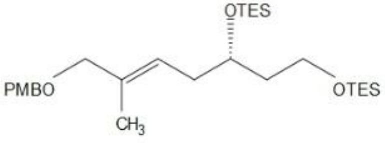

S-17

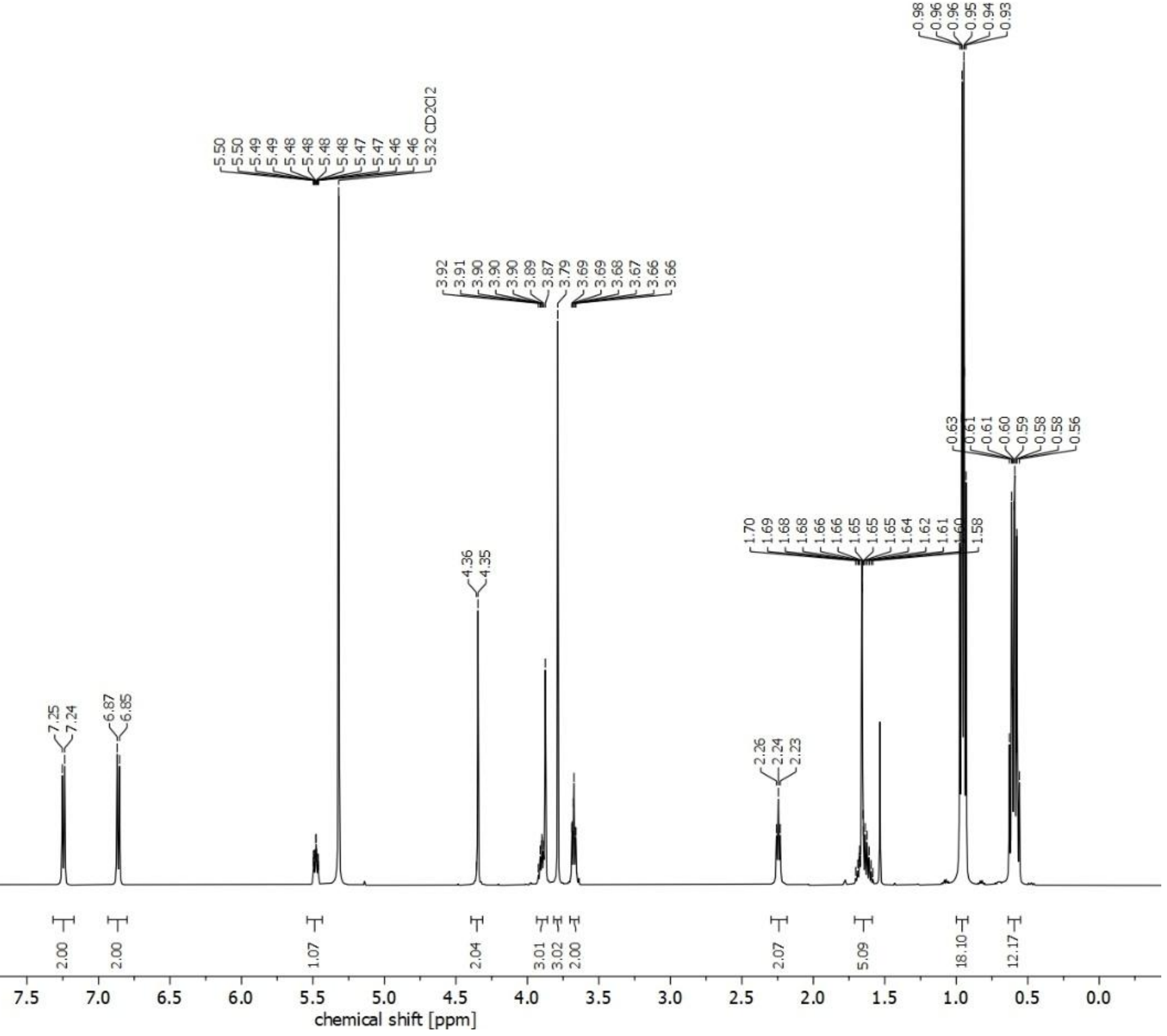

Nucleus: ^{13}C

Frequency: 125.75 MHz

Solvent: CD_2Cl_2

Temperature: 298.0 K

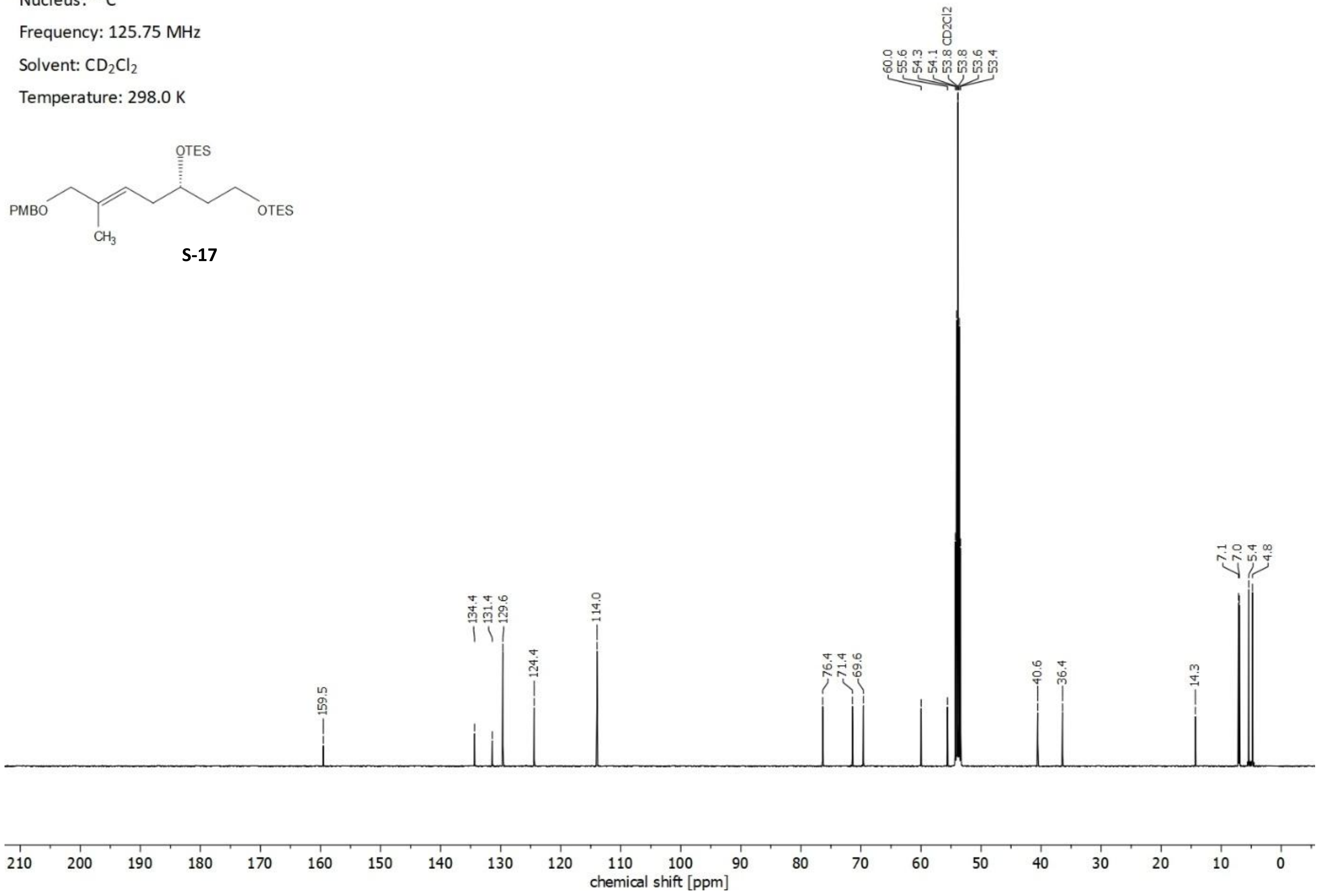

S-17

Nucleus: ^{1}H

Frequency: 500.04 MHz

Solvent: CD_2Cl_2

Temperature: 298.0 K

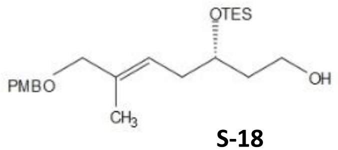

S-18

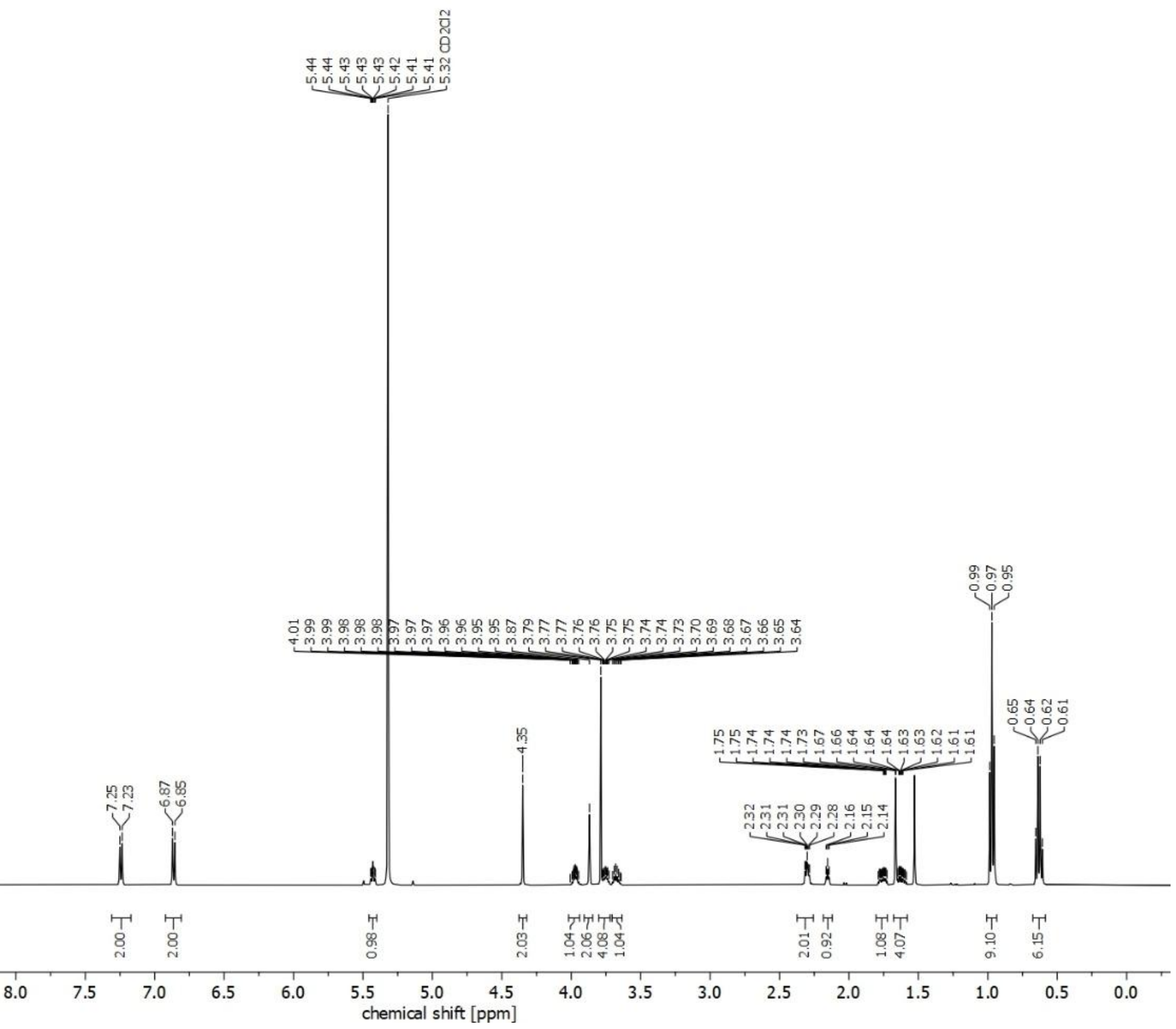

Nucleus: ^{13}C

Frequency: 125.75 MHz

Solvent: CD_2Cl_2

Temperature: 298.0 K

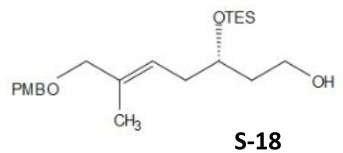

S-18

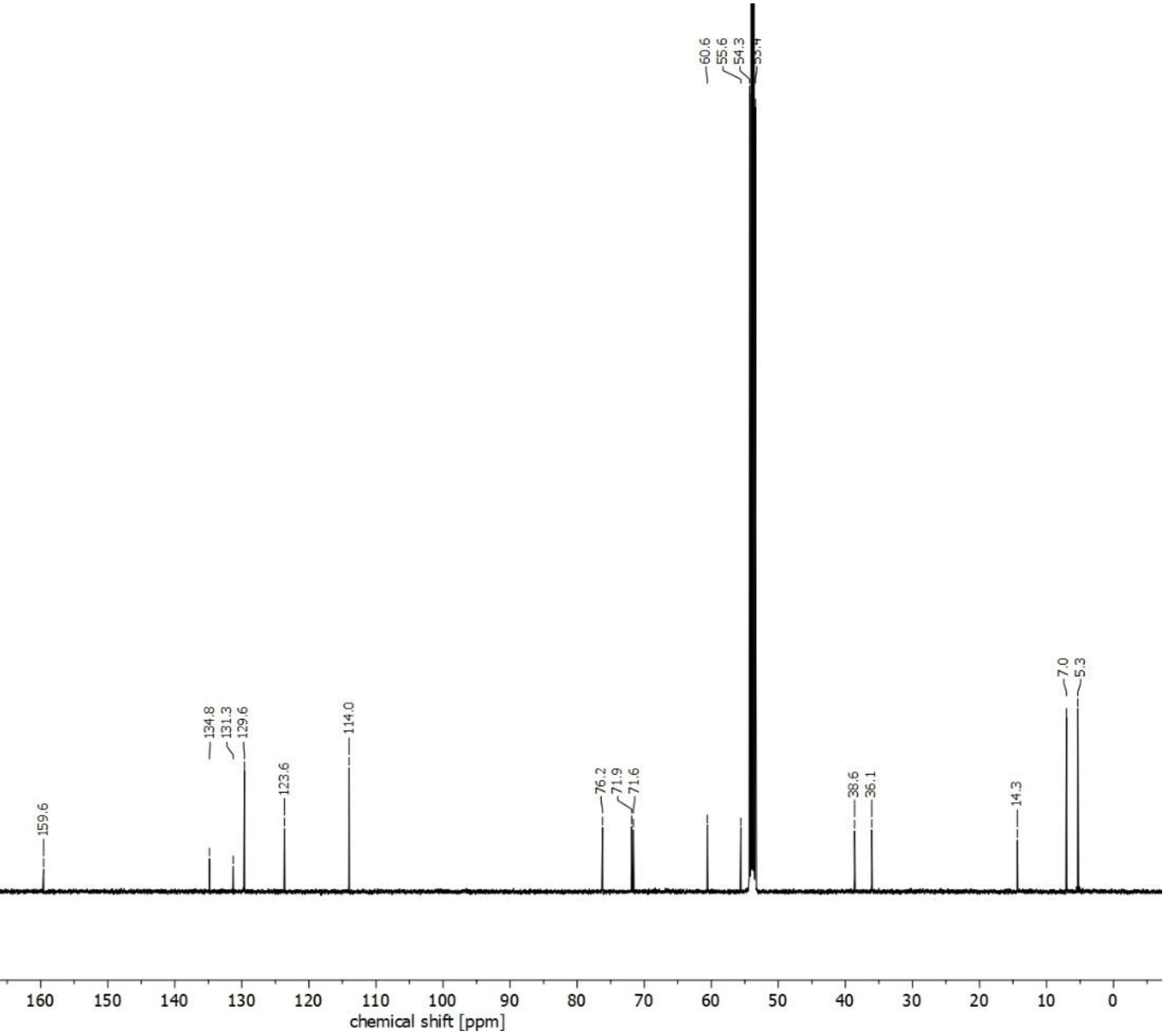

Nucleus: ^{1}H

Frequency: 500.04 MHz

Solvent: CD_2Cl_2

Temperature: 298.0 K

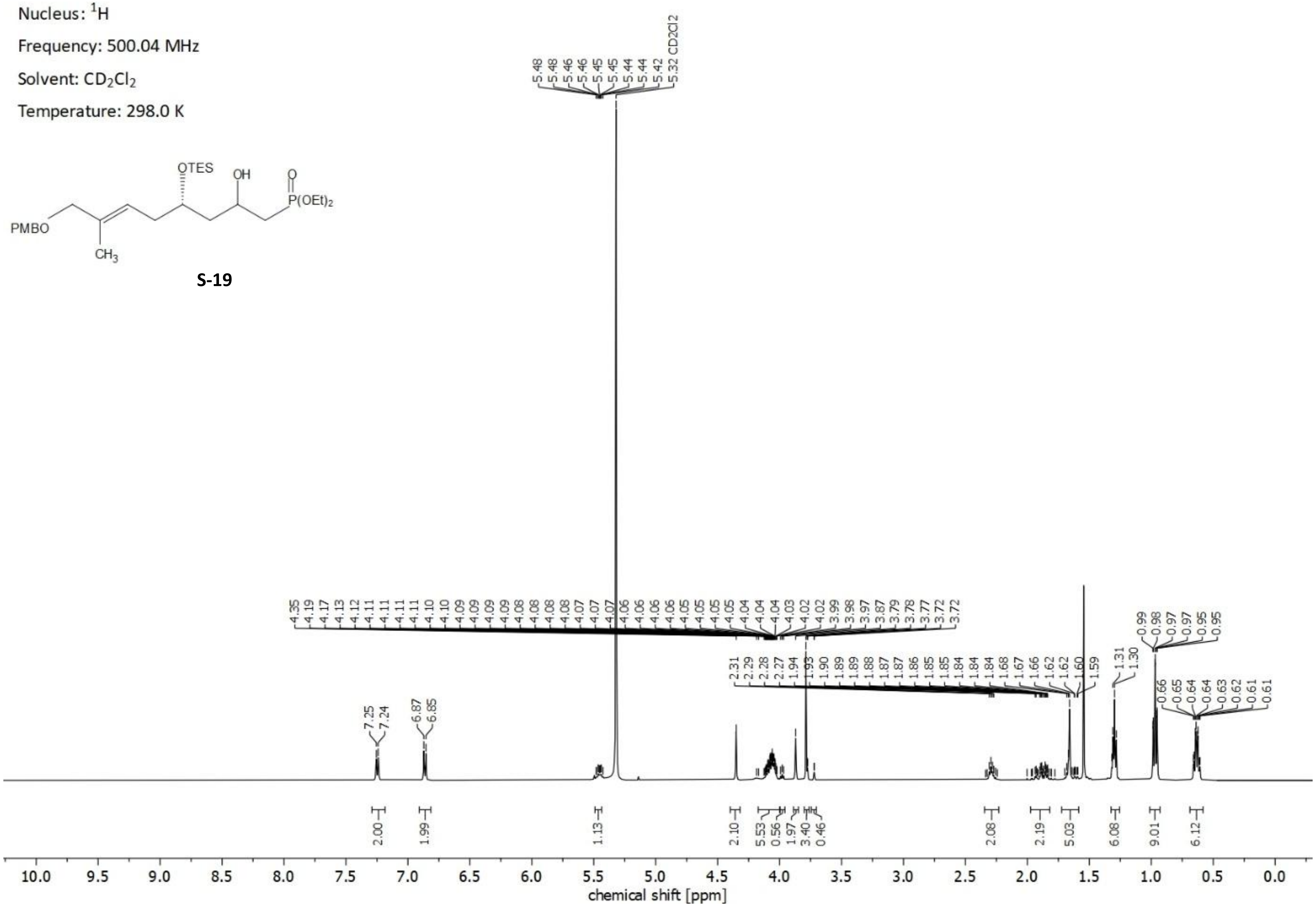

S-19

Nucleus: ^{13}C

Frequency: 125.75 MHz

Solvent: CD_2Cl_2

Temperature: 298.0 K

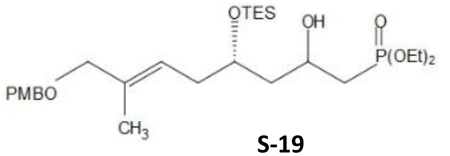

S-19

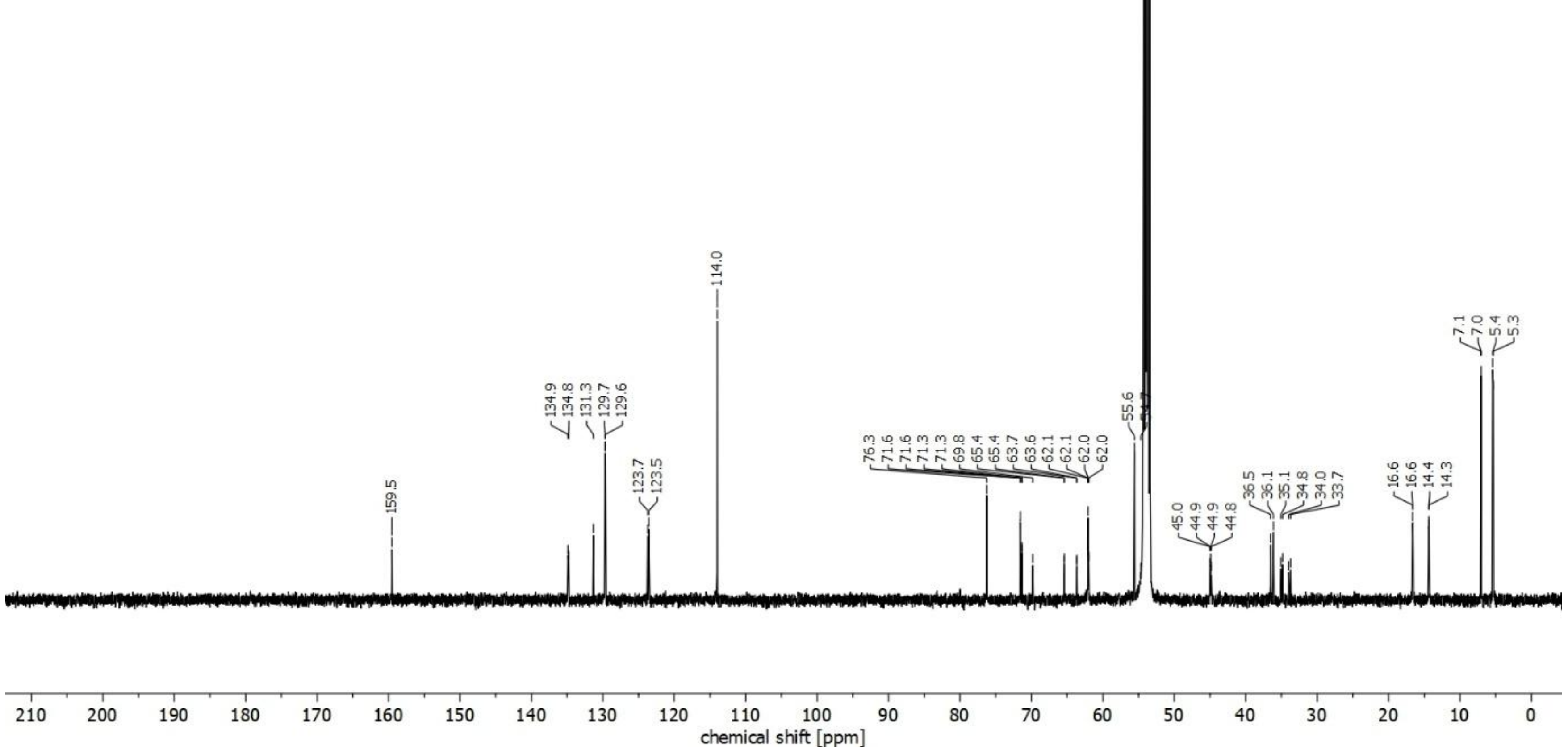

Nucleus: ^{1}H

Frequency: 500.04 MHz

Solvent: CD_2Cl_2

Temperature: 298.0 K

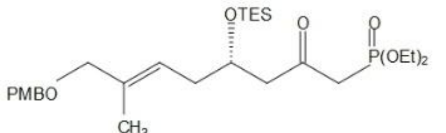

33

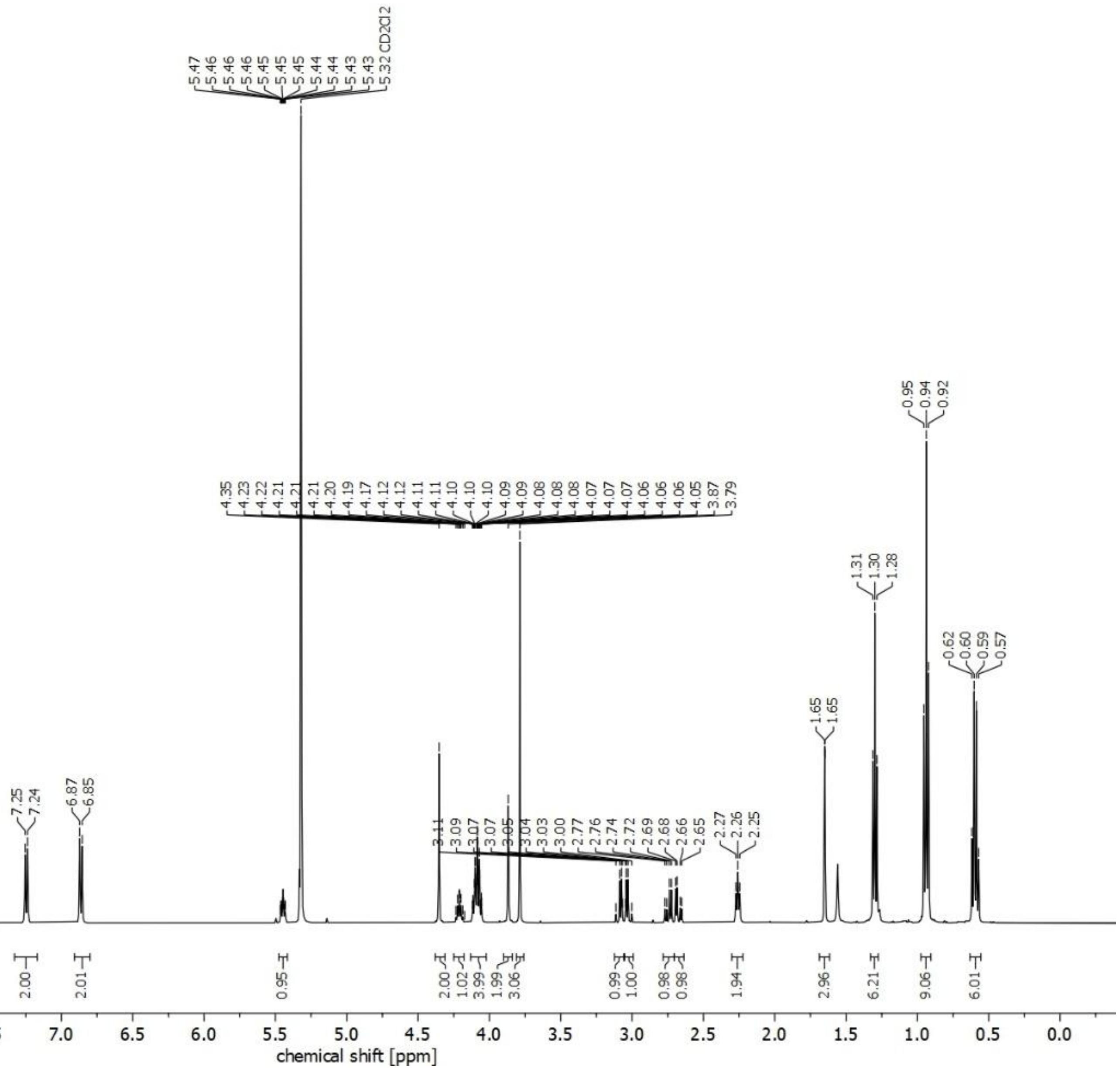

Nucleus: ^{13}C

Frequency: 125.75 MHz

Solvent: CD_2Cl_2

Temperature: 298.0 K

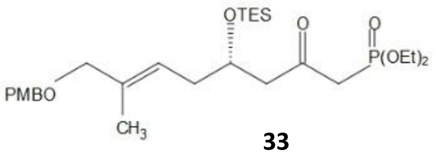

33

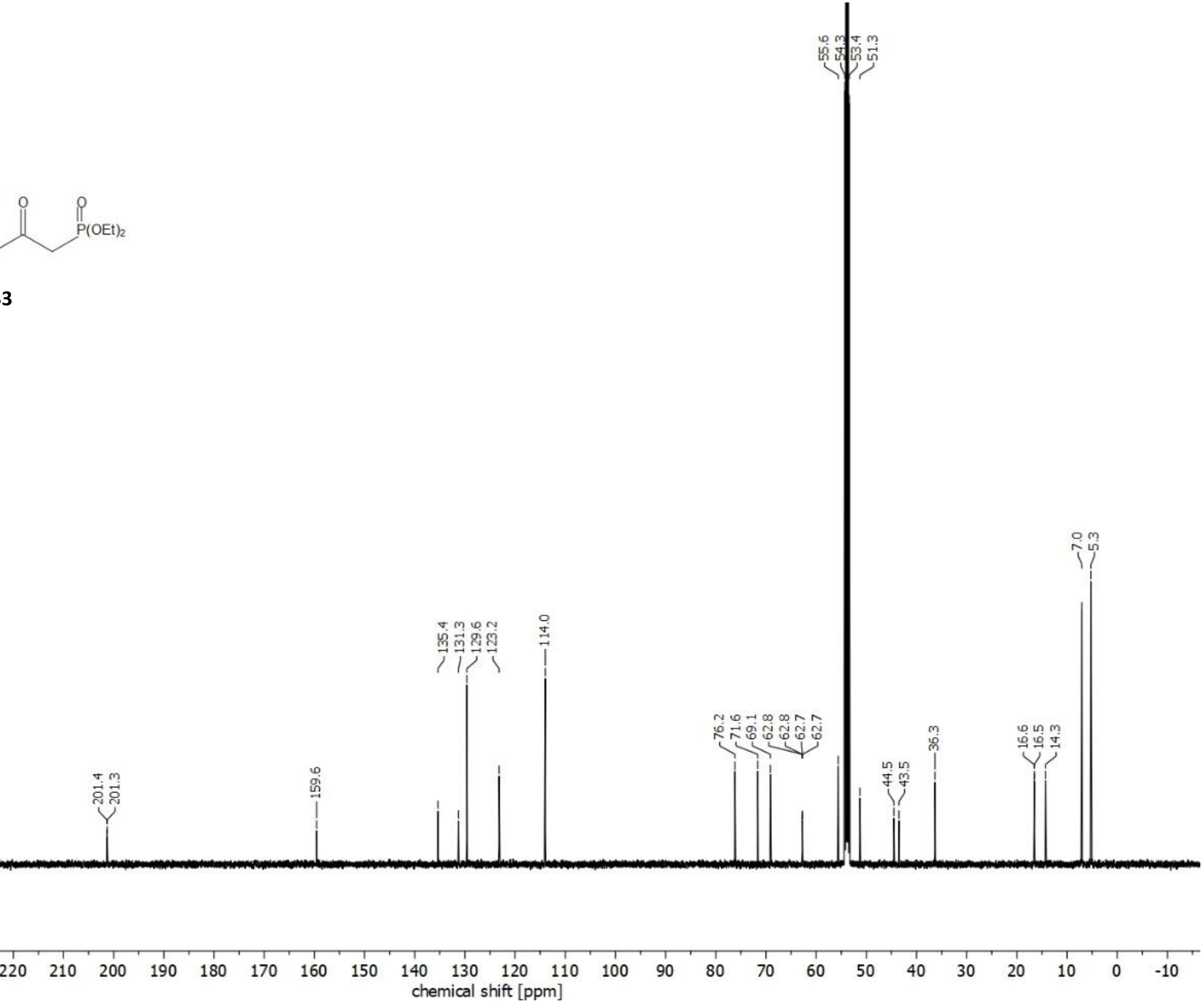

Nucleus: ^{1}H

Frequency: 500.04 MHz

Solvent: CD_2Cl_2

Temperature: 298.0 K

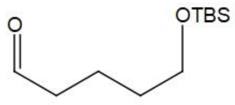

S-20

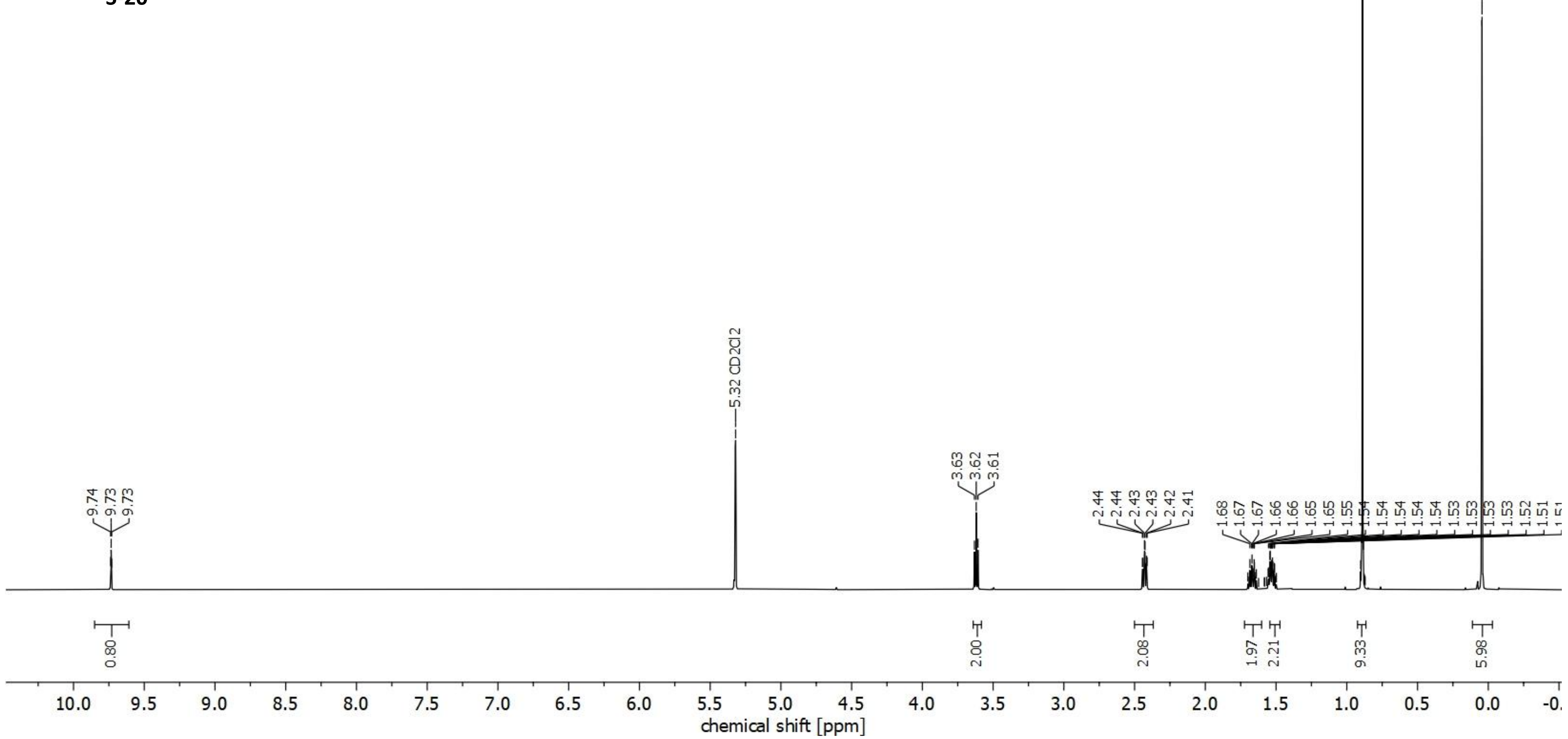

Nucleus: ^{13}C

Frequency: 125.75 MHz

Solvent: CD_2Cl_2

Temperature: 298.0 K

S-20

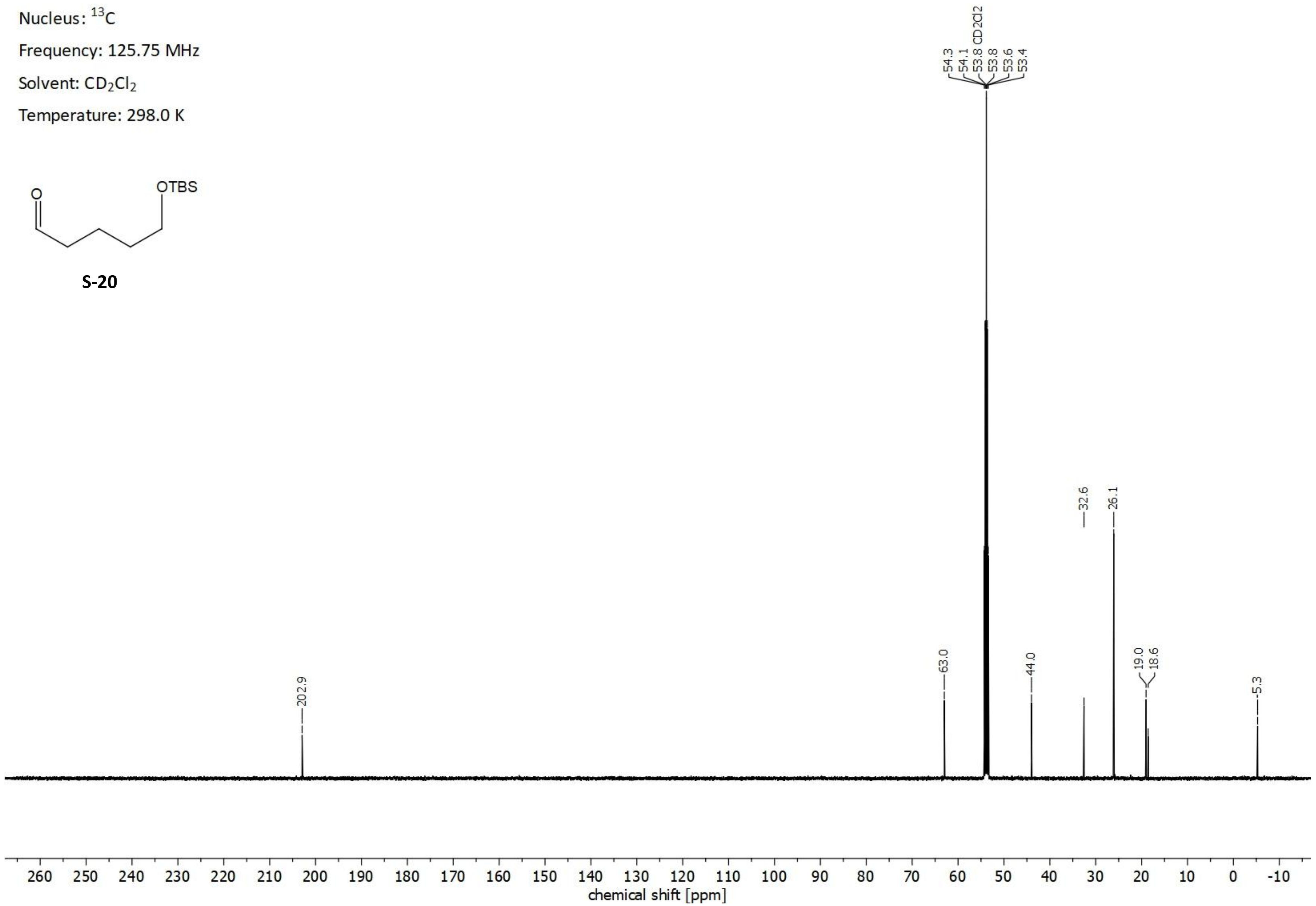

Nucleus: ^{1}H

Frequency: 400.13 MHz

Solvent: CD_2Cl_2

Temperature: 298.0 K

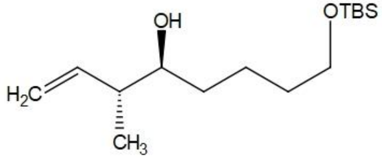

S-21

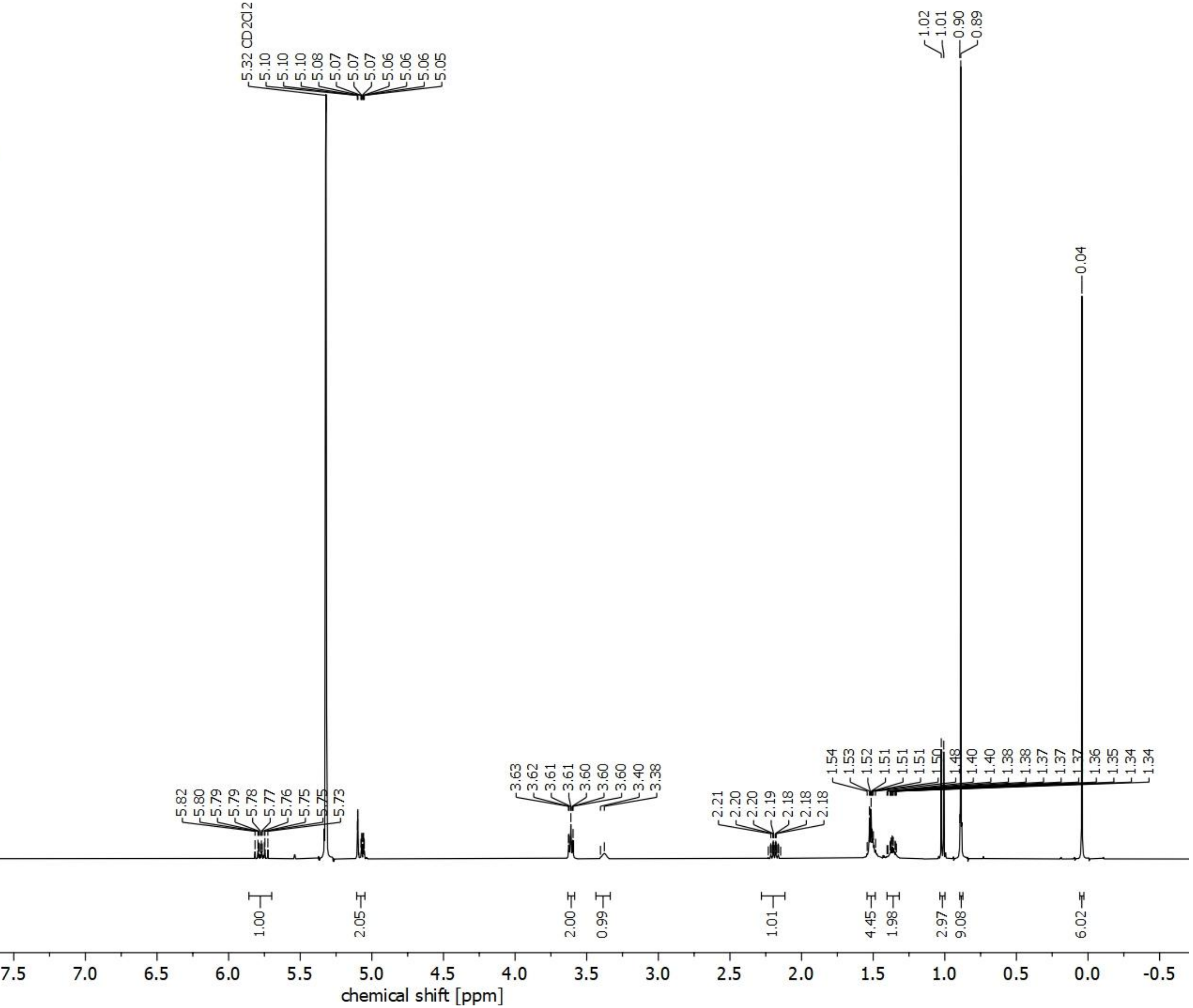

Nucleus: ^{13}C

Frequency: 125.75 MHz

Solvent: CD_2Cl_2

Temperature: 298.0 K

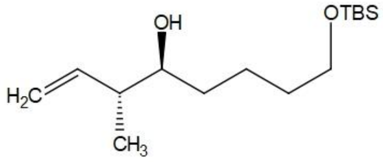

S-21

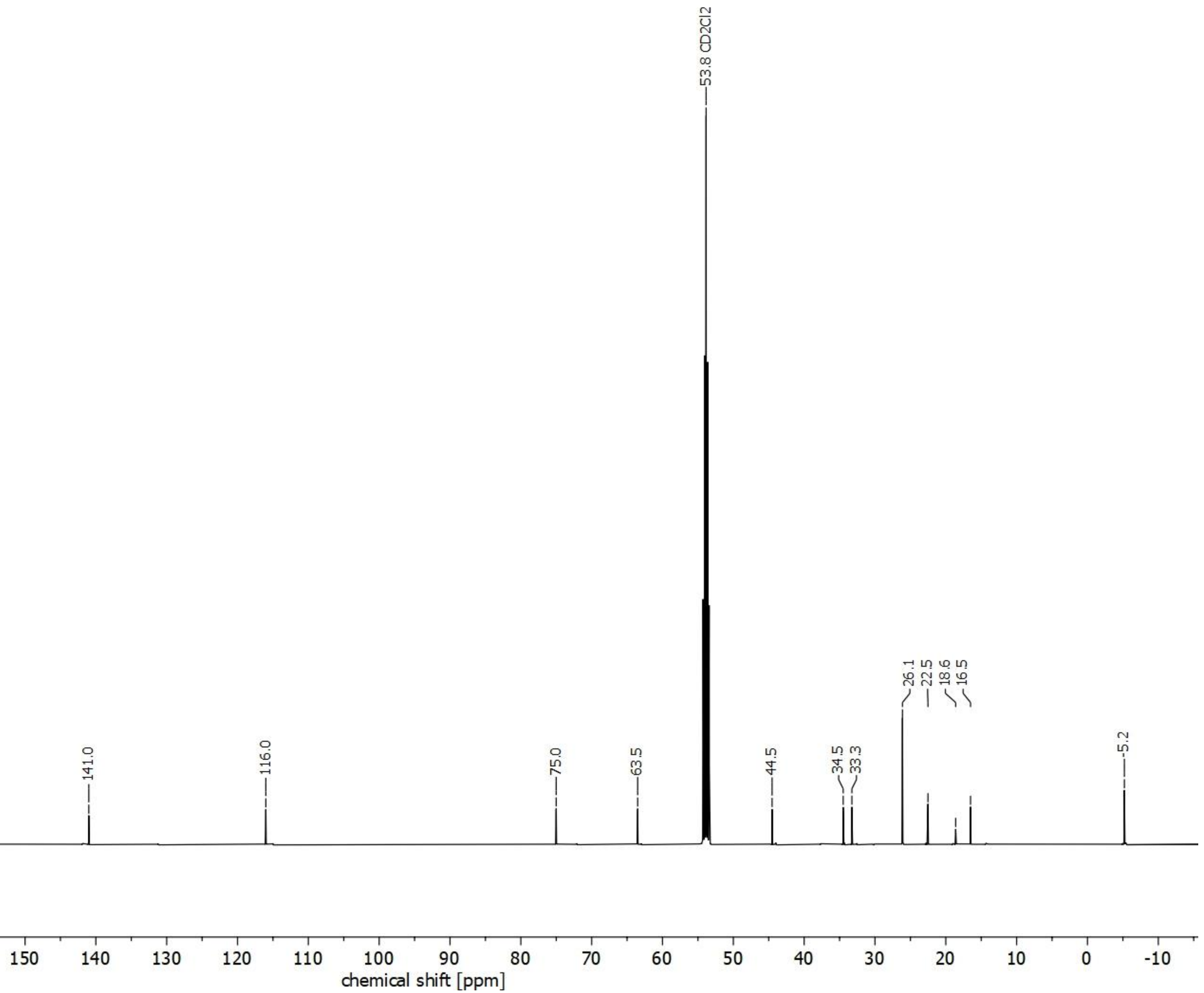

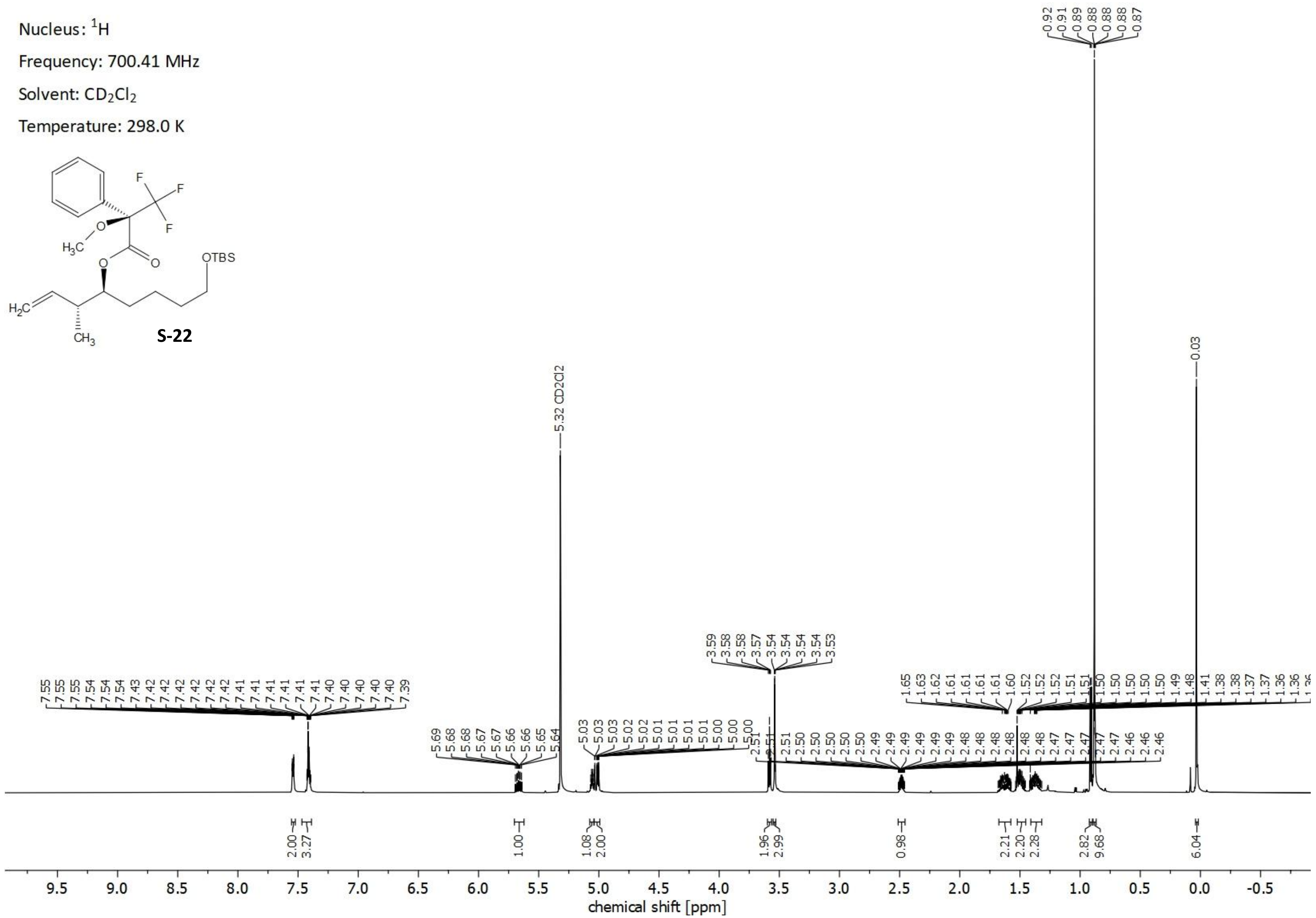
Nucleus: ¹H
Frequency: 700.41 MHz
Solvent: CD2Cl2
Temperature: 298.0 K
OTBS
S-22
5.32 CD2Cl2
0.03
chemical shift [ppm]

Nucleus: ^{13}C

Frequency: 176.14 MHz

Solvent: CD_2Cl_2

Temperature: 298.0 K

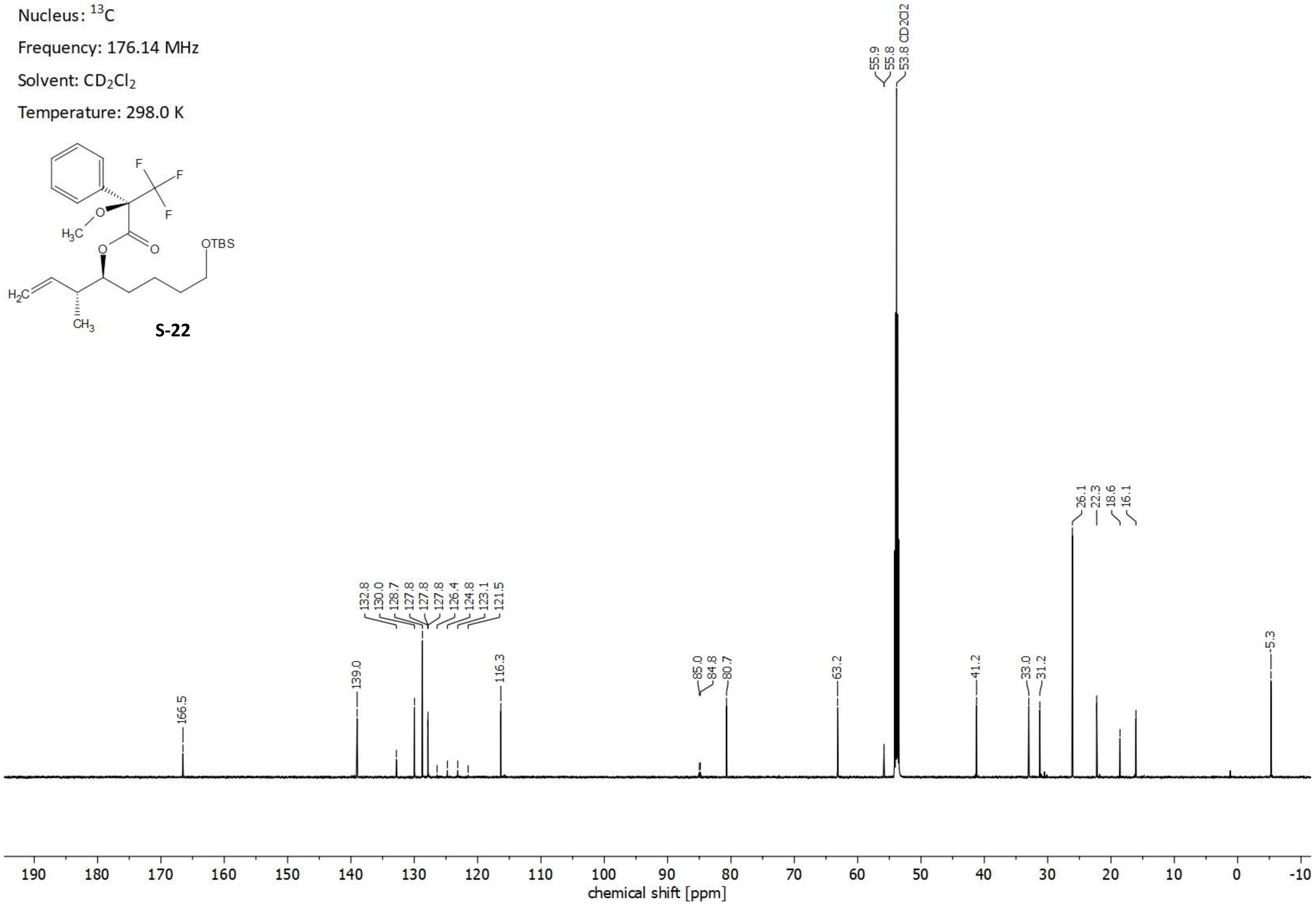

S-22

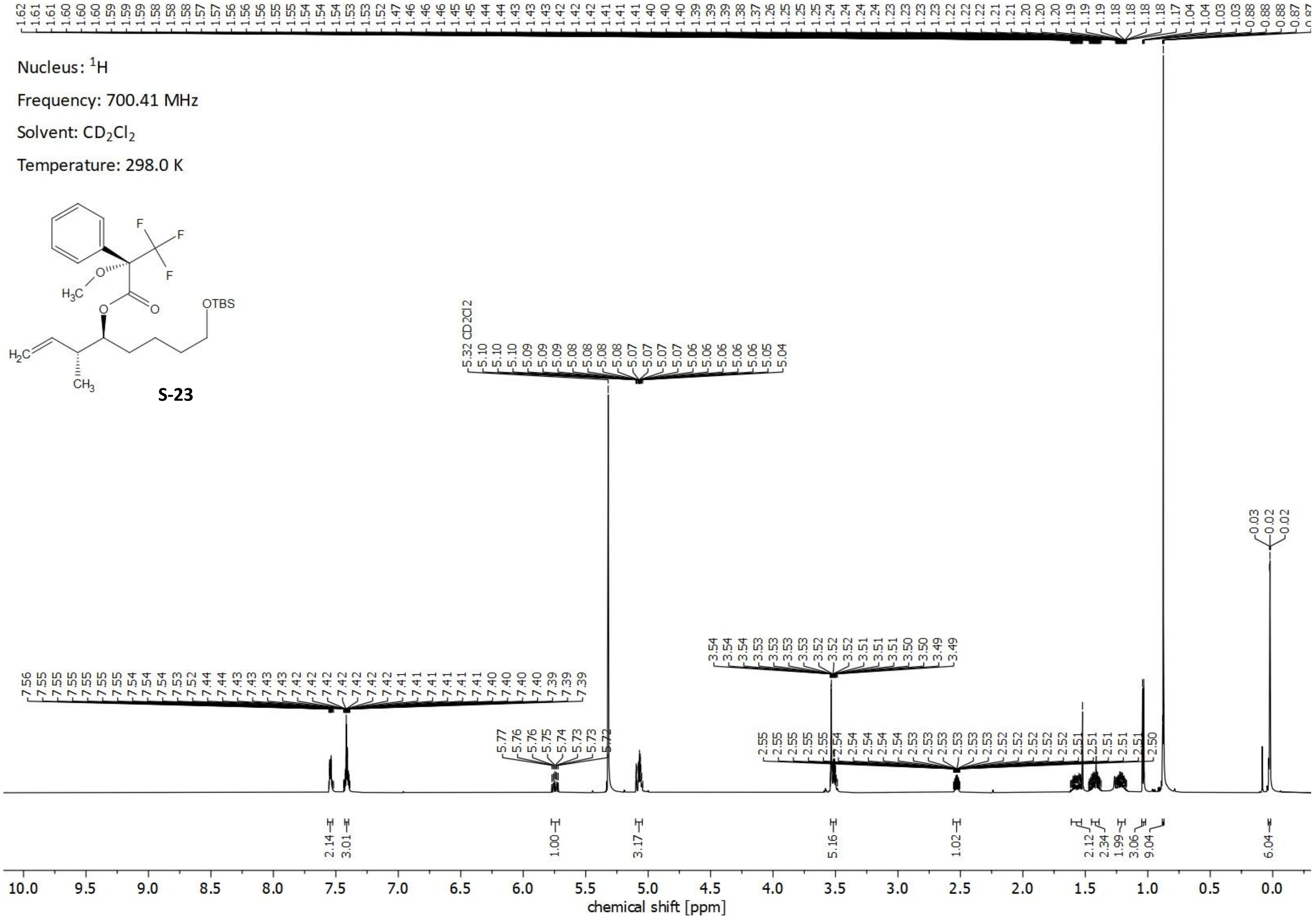
Nucleus: ^{1}H
Frequency: 700.41 MHz
Solvent: CD_2Cl_2
Temperature: 298.0 K
S-23
chemical shift [ppm]

Nucleus: ^{13}C

Frequency: 176.14 MHz

Solvent: CD_2Cl_2

Temperature: 298.0 K

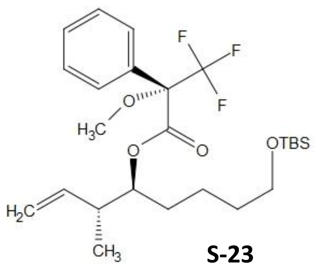

S-23

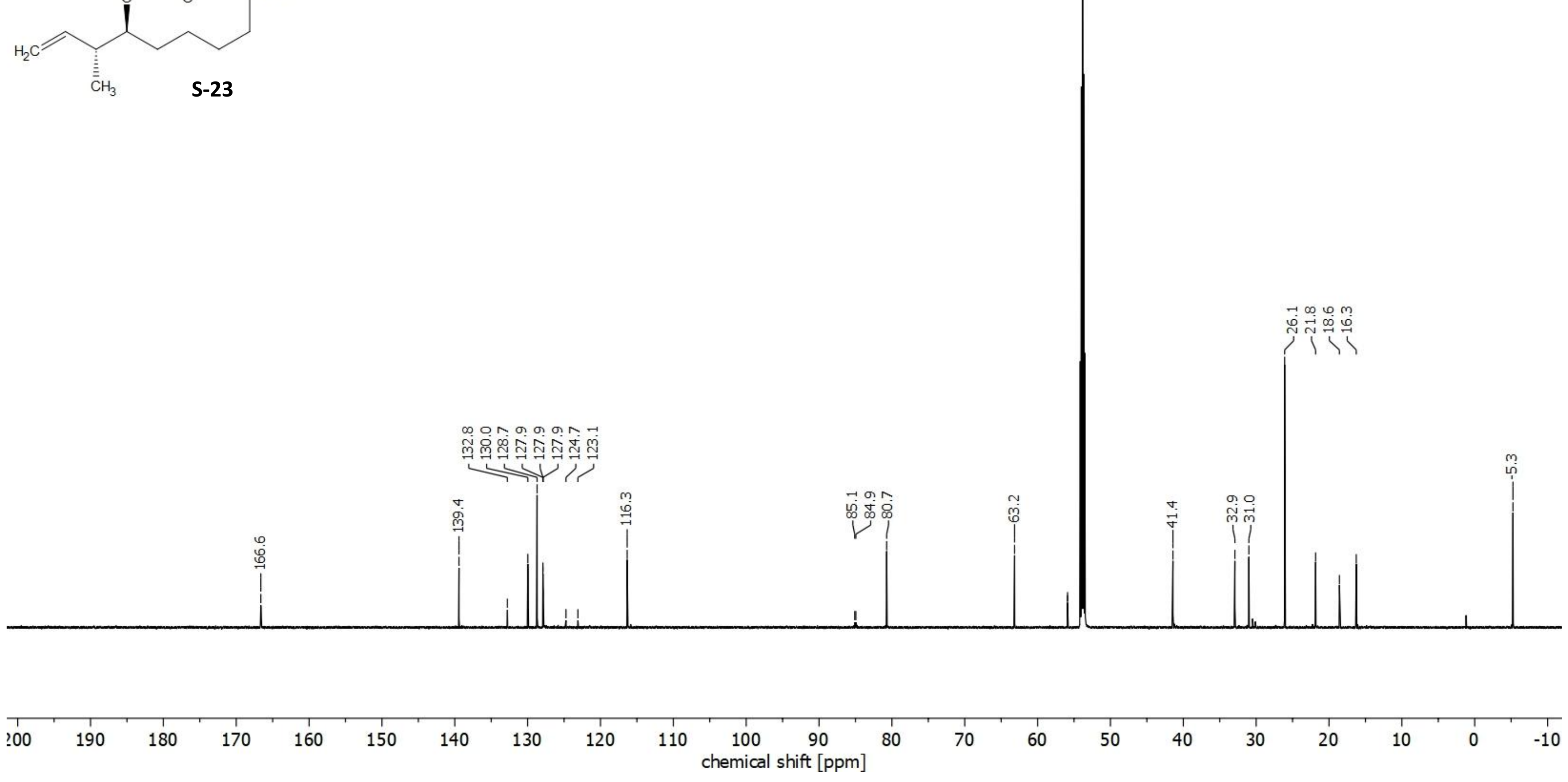

Nucleus: ^{1}H

Frequency: 400.13 MHz

Solvent: CD_2Cl_2

Temperature: 298.0 K

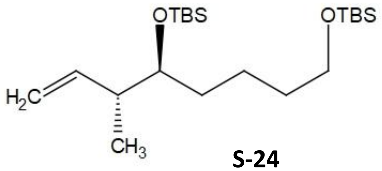

S-24

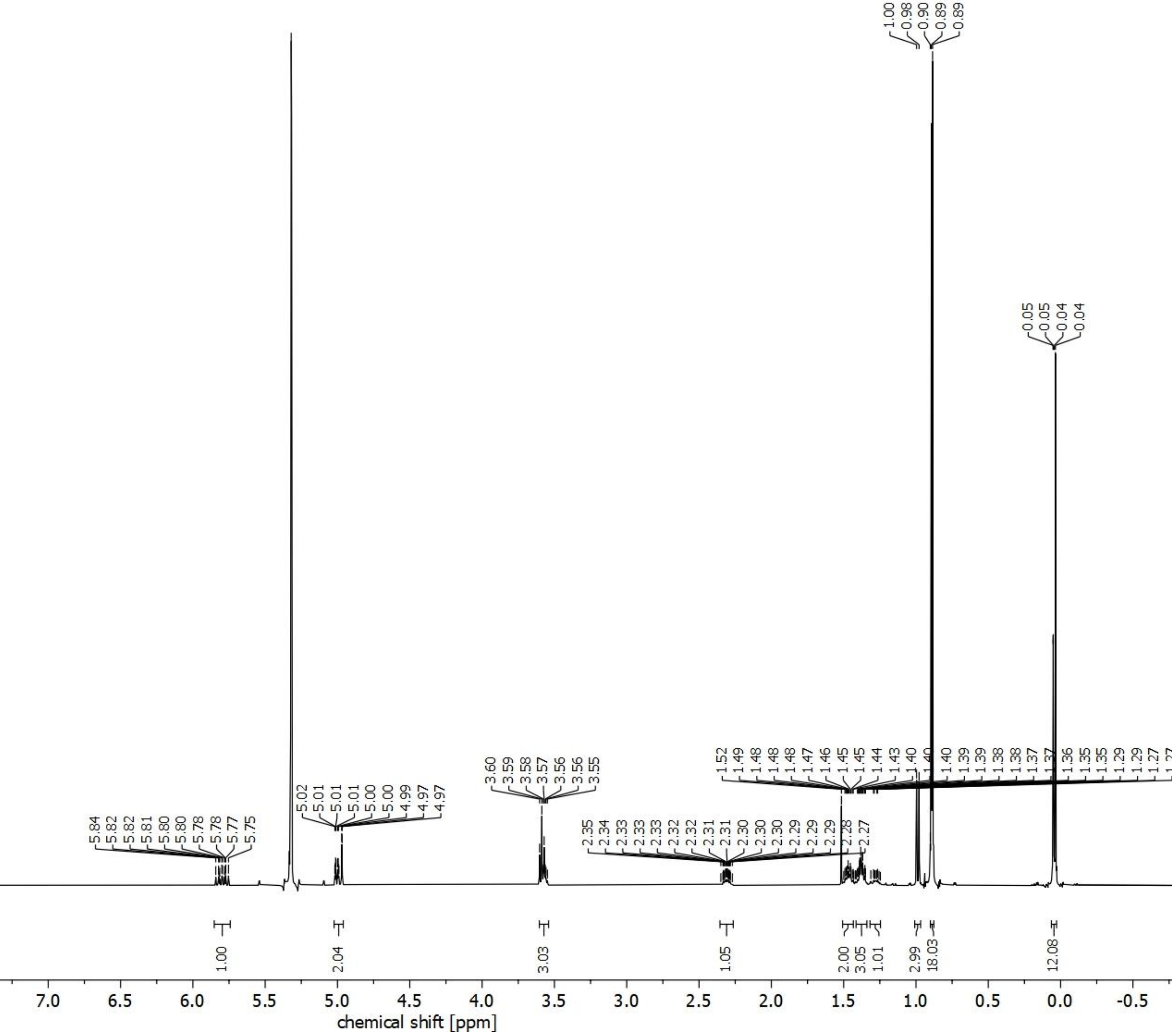

Nucleus: ^{13}C

Frequency: 125.75 MHz

Solvent: CD_2Cl_2

Temperature: 298.0 K

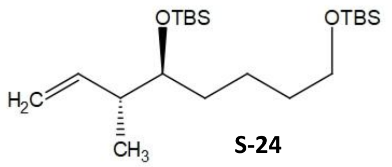

S-24

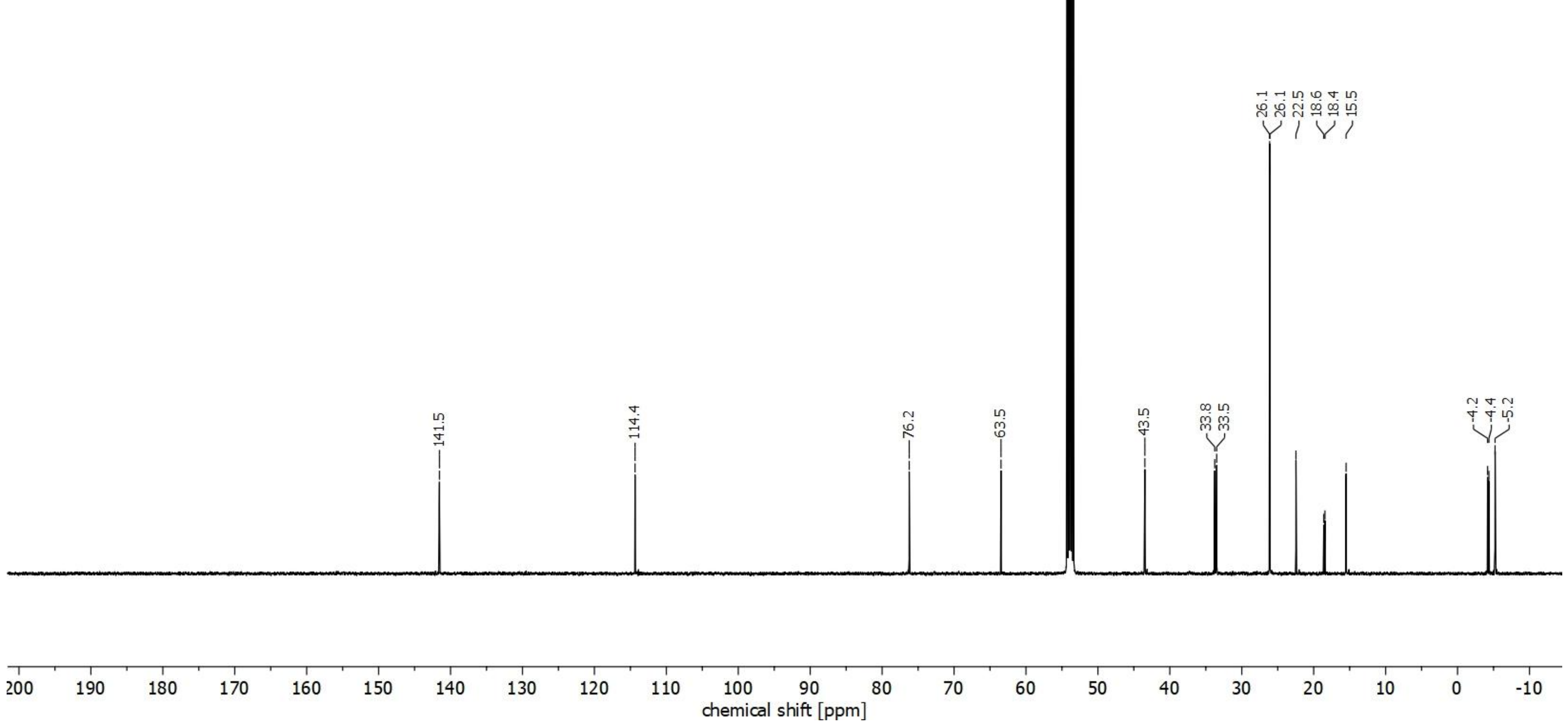

Nucleus: ^1H

Frequency: 700.41 MHz

Solvent: CD_2Cl_2

Temperature: 298.0 K

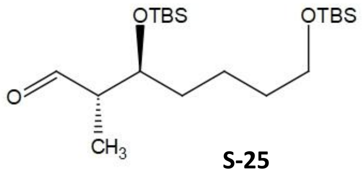

S-25

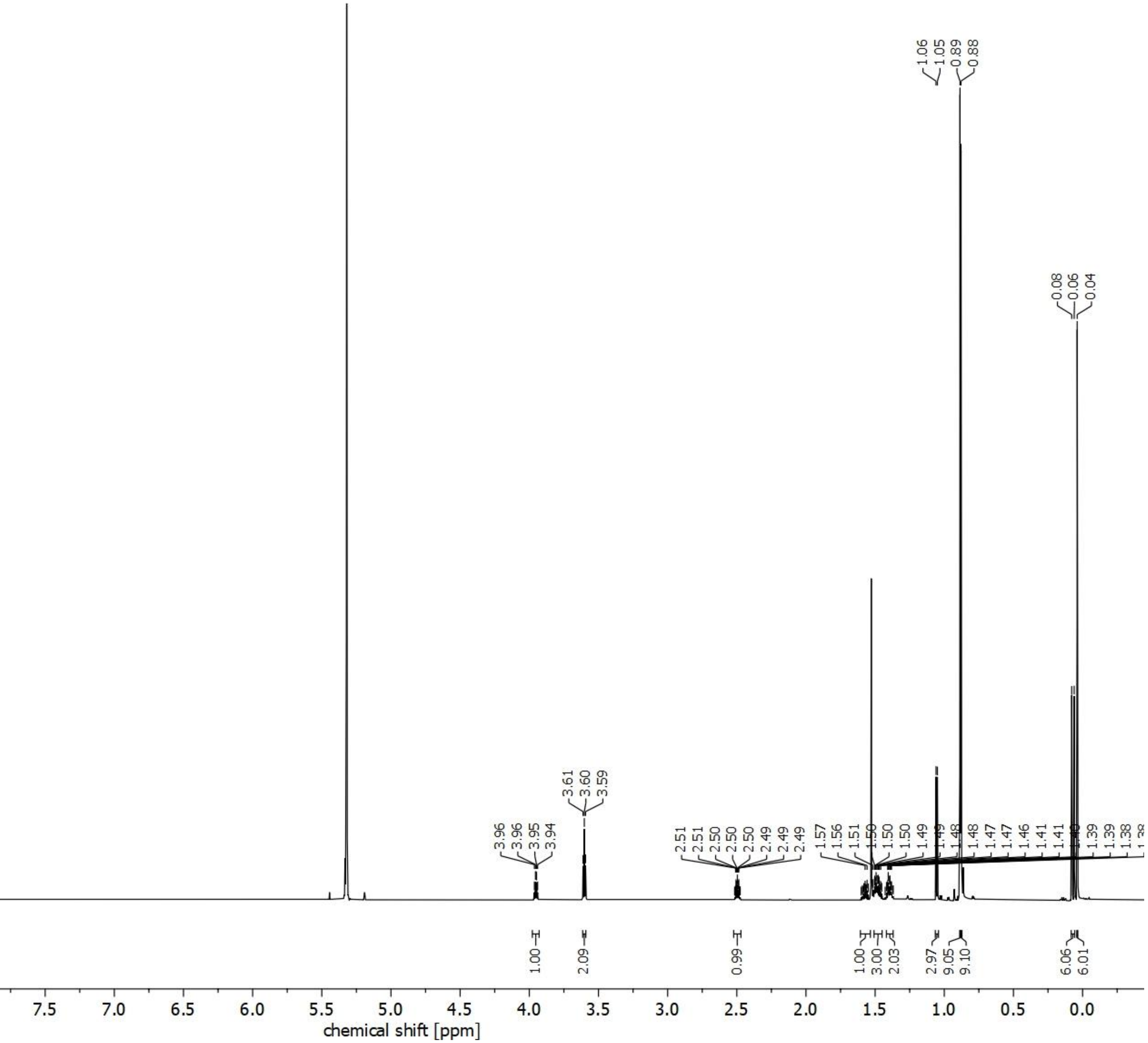

Nucleus: ^{13}C

Frequency: 176.14 MHz

Solvent: CD_2Cl_2

Temperature: 298.0 K

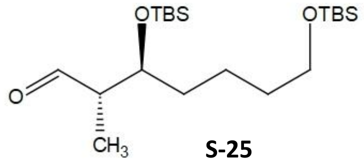

S-25

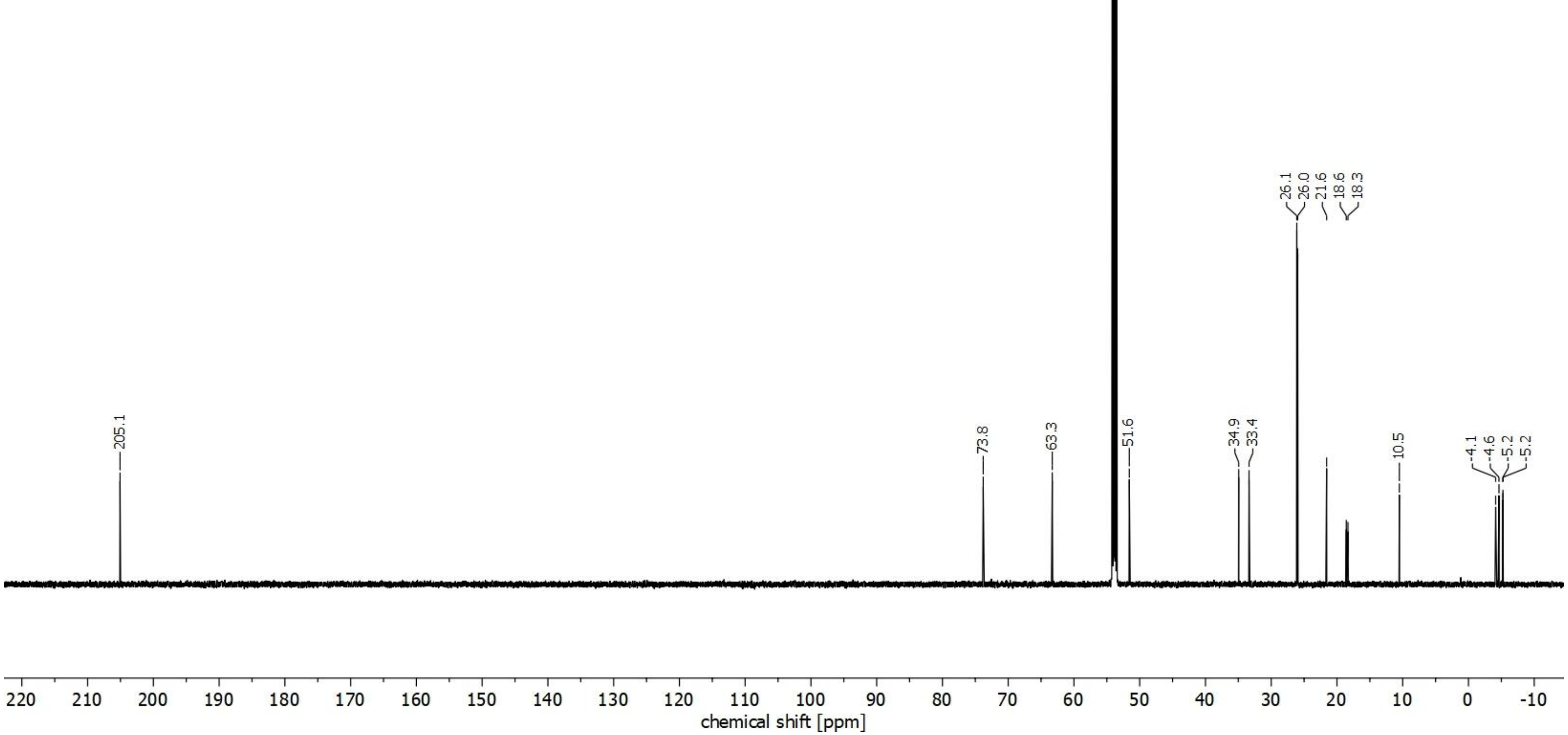

Nucleus: ^{1}H

Frequency: 700.41 MHz

Solvent: CD_2Cl_2

Temperature: 298.0 K

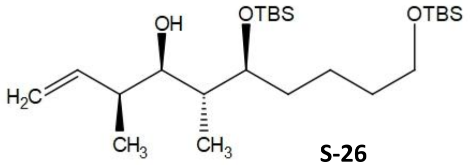

S-26

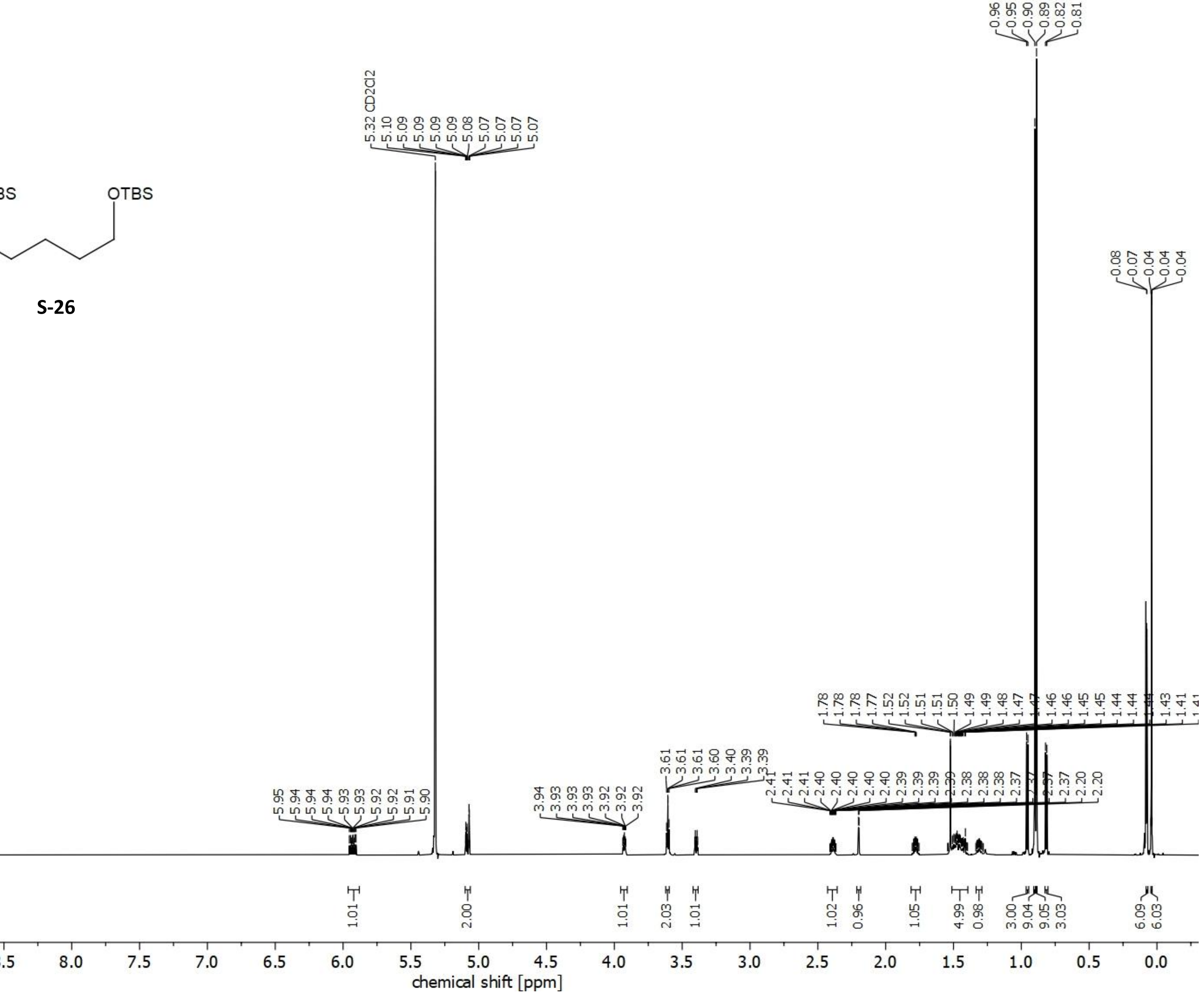

Nucleus: ^{13}C

Frequency: 176.14 MHz

Solvent: CD_2Cl_2

Temperature: 298.0 K

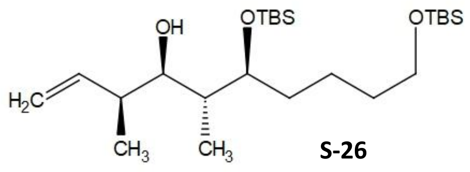

S-26

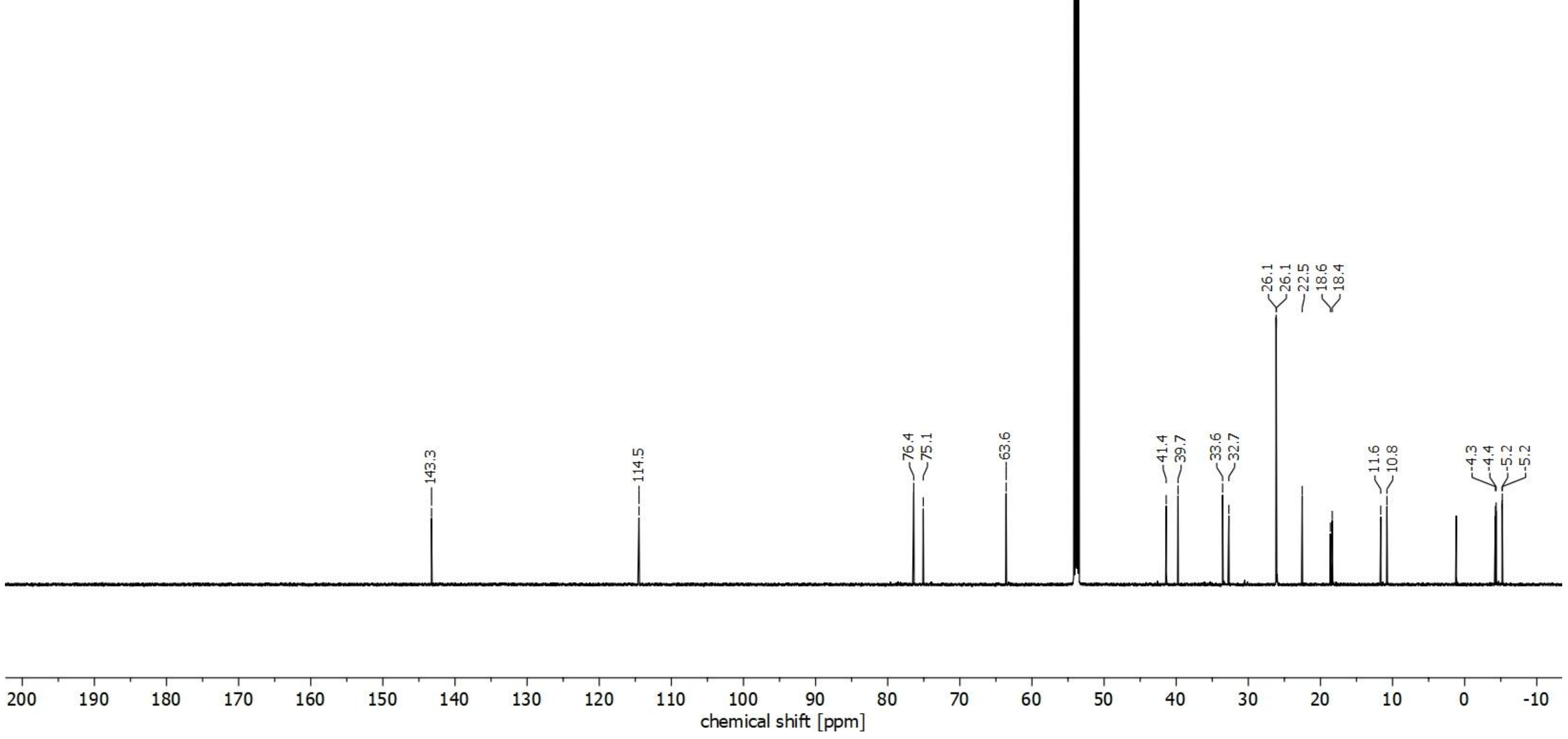

Nucleus: ^{1}H

Frequency: 400.13 MHz

Solvent: CD_2Cl_2

Temperature: 298.0 K

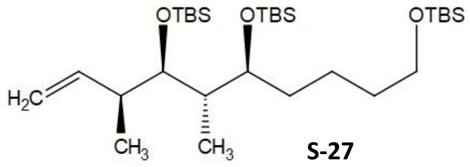

S-27

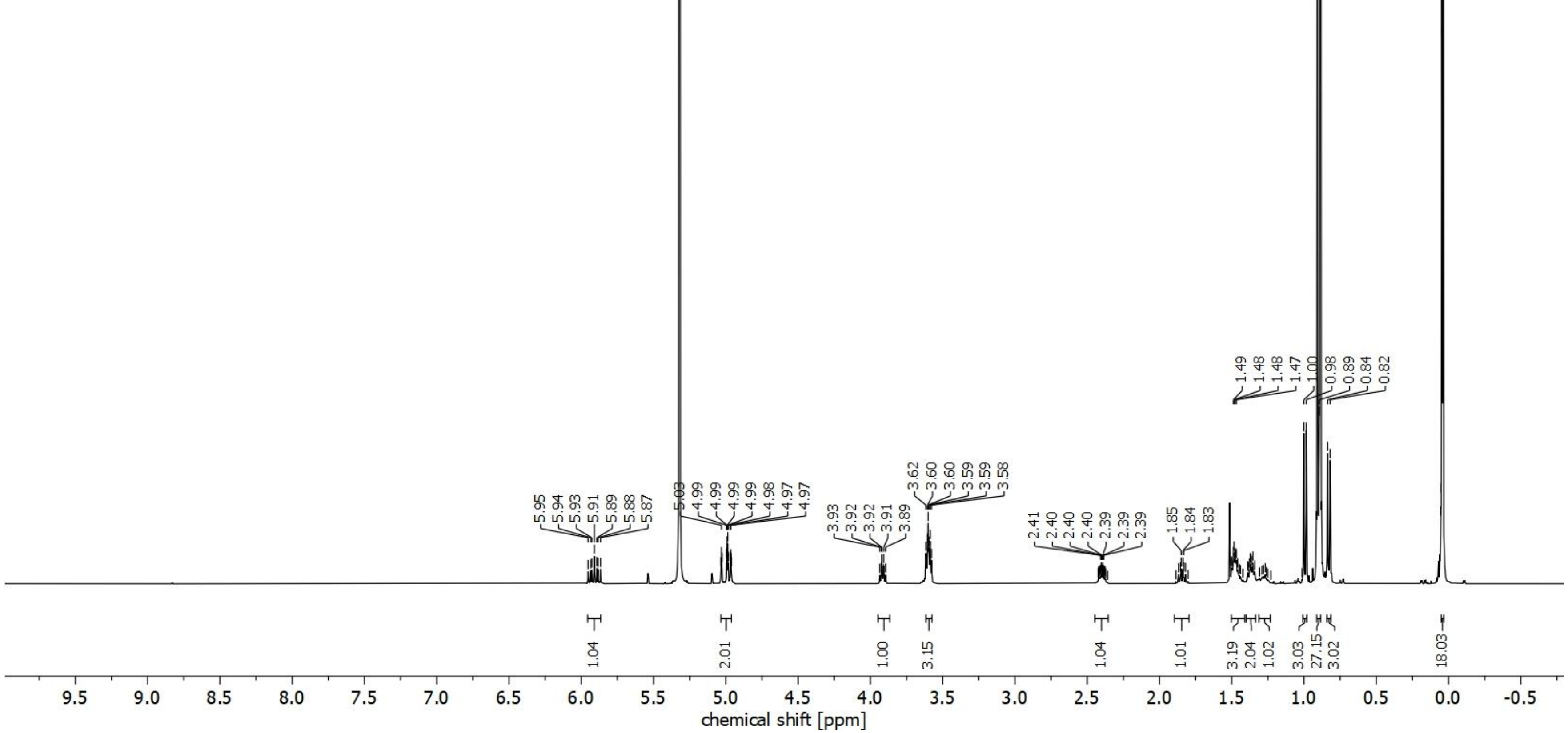

Nucleus: ^{13}C

Frequency: 176.14 MHz

Solvent: CD_2Cl_2

Temperature: 298.0 K

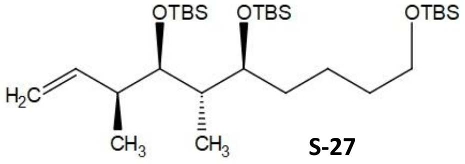

S-27

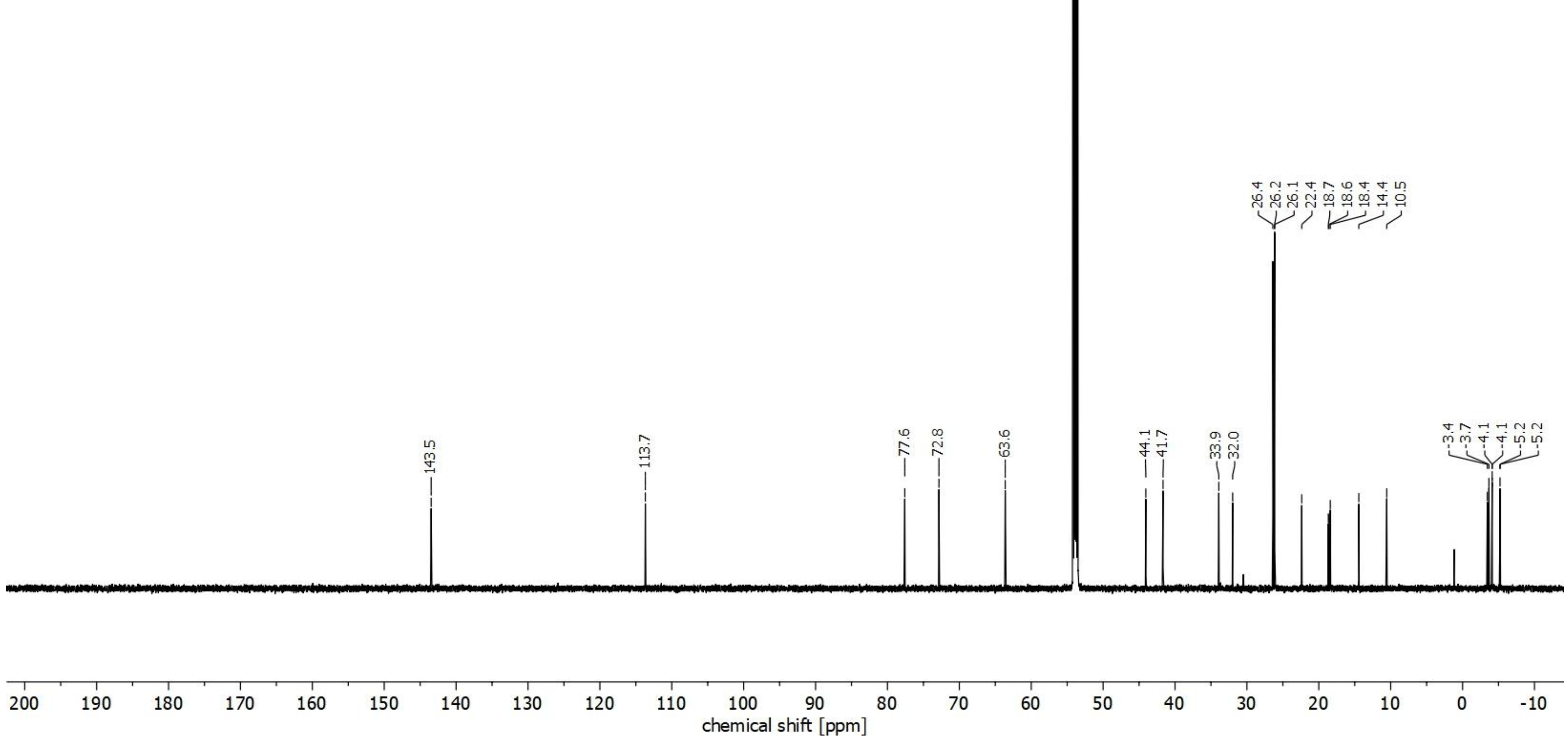

Nucleus: ^{1}H

Frequency: 700.41 MHz

Solvent: CD_2Cl_2

Temperature: 298.0 K

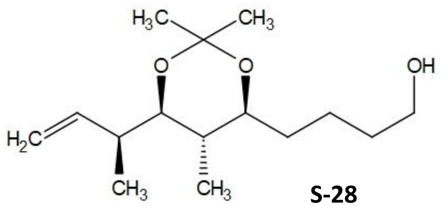

S-28

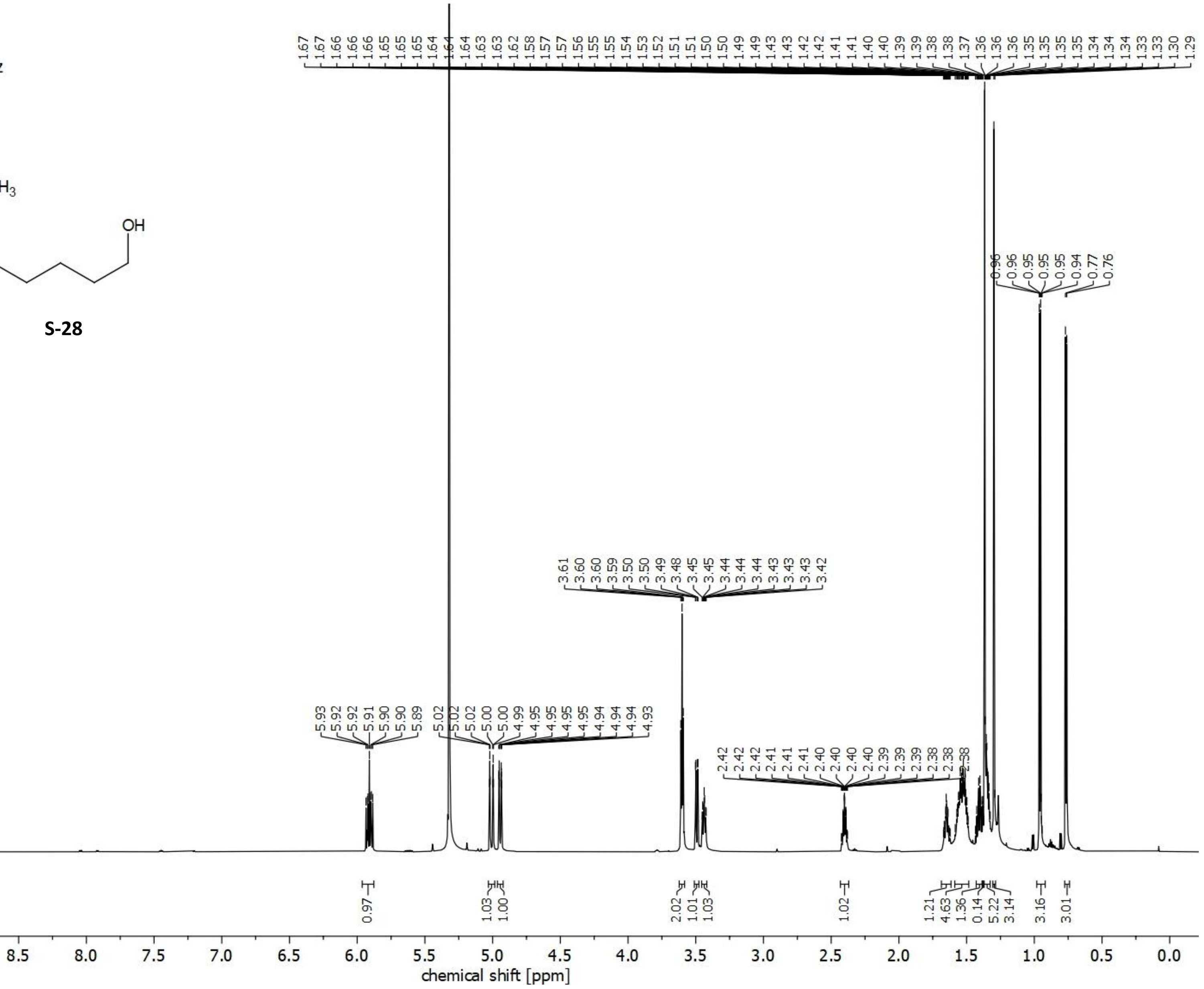

Nucleus: ^{13}C

Frequency: 176.14 MHz

Solvent: CD_2Cl_2

Temperature: 298.0 K

S-28

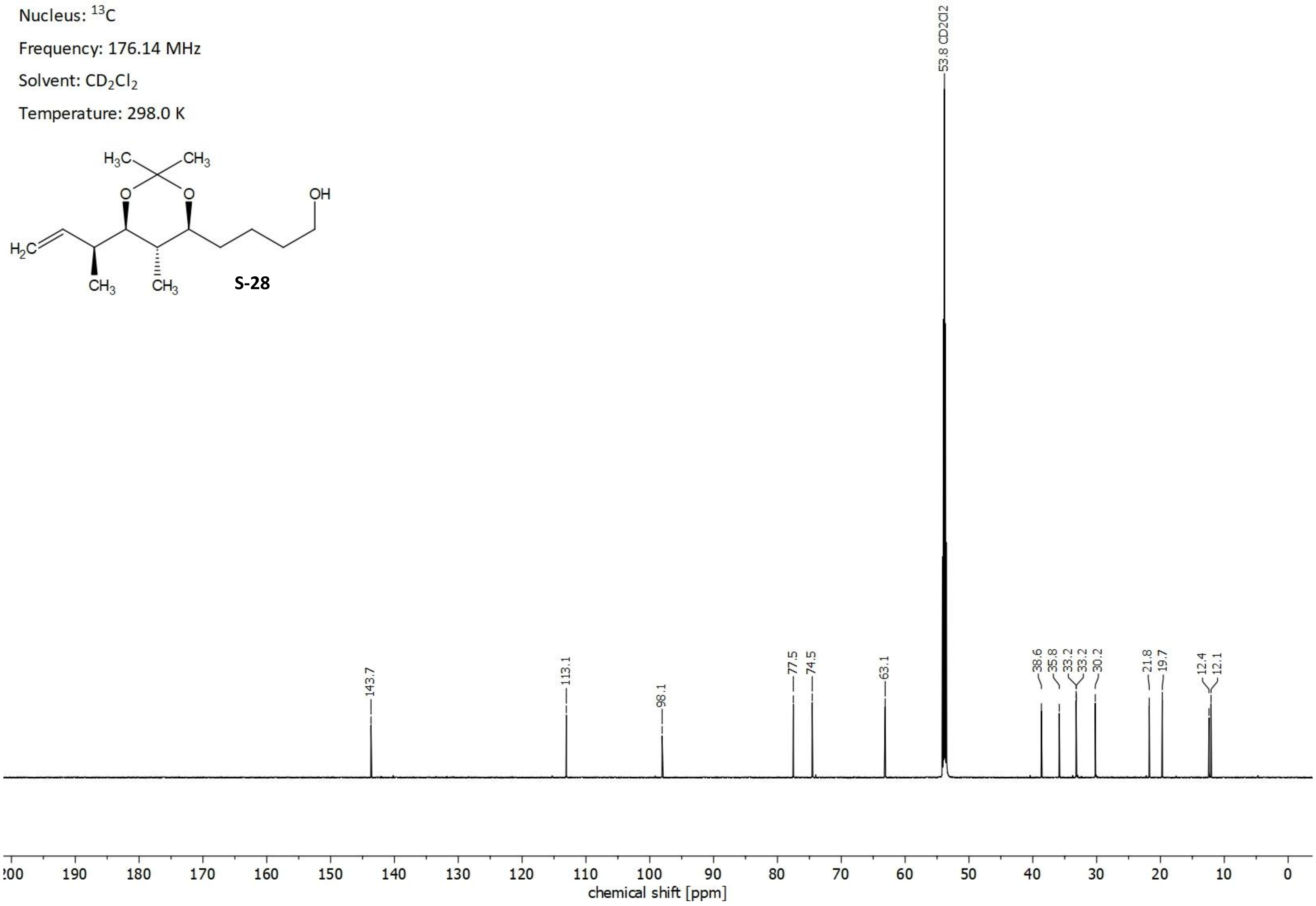

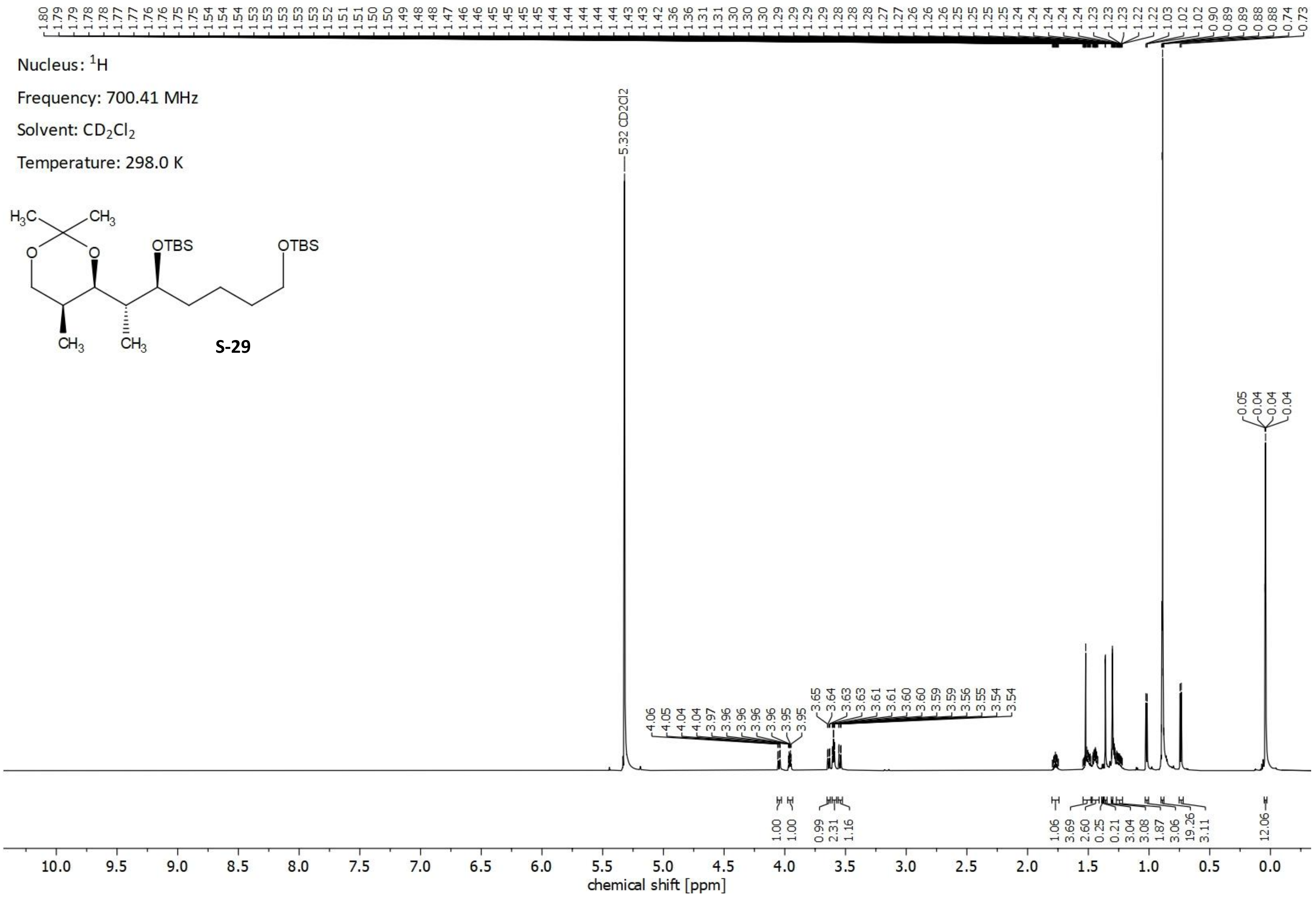
Nucleus: ^{1}H
Frequency: 700.41 MHz
Solvent: CD_2Cl_2
Temperature: 298.0 K
S-29
5.32 CD2Cl2
chemical shift [ppm]

Nucleus: ^{13}C

Frequency: 176.14 MHz

Solvent: CD_2Cl_2

Temperature: 298.0 K

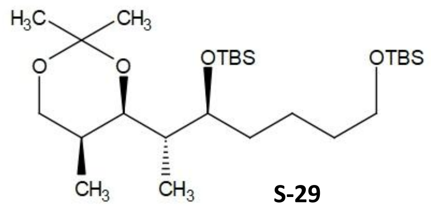

S-29

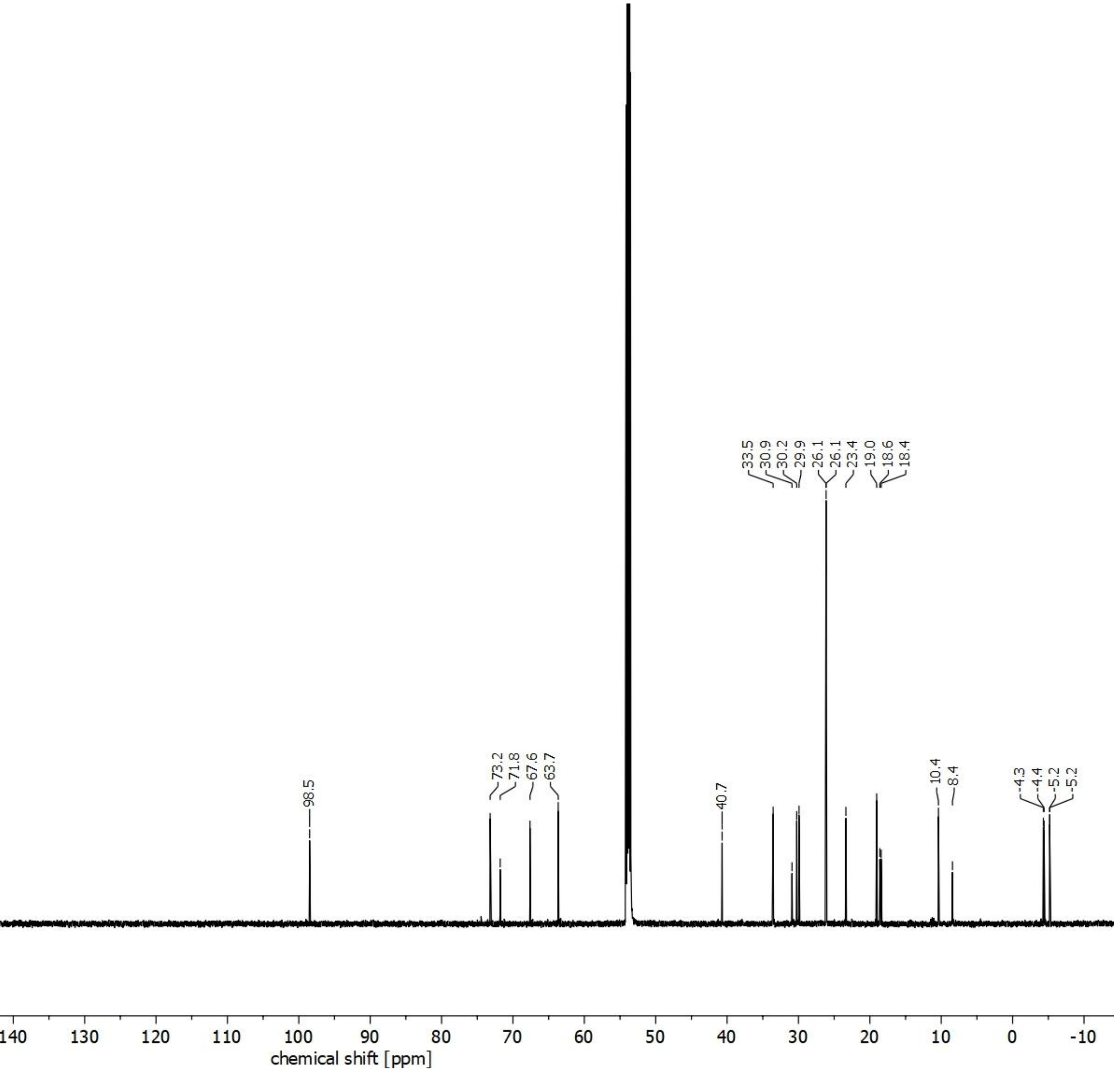

Nucleus: ^{1}H

Frequency: 700.41 MHz

Solvent: CD_2Cl_2

Temperature: 298.0 K

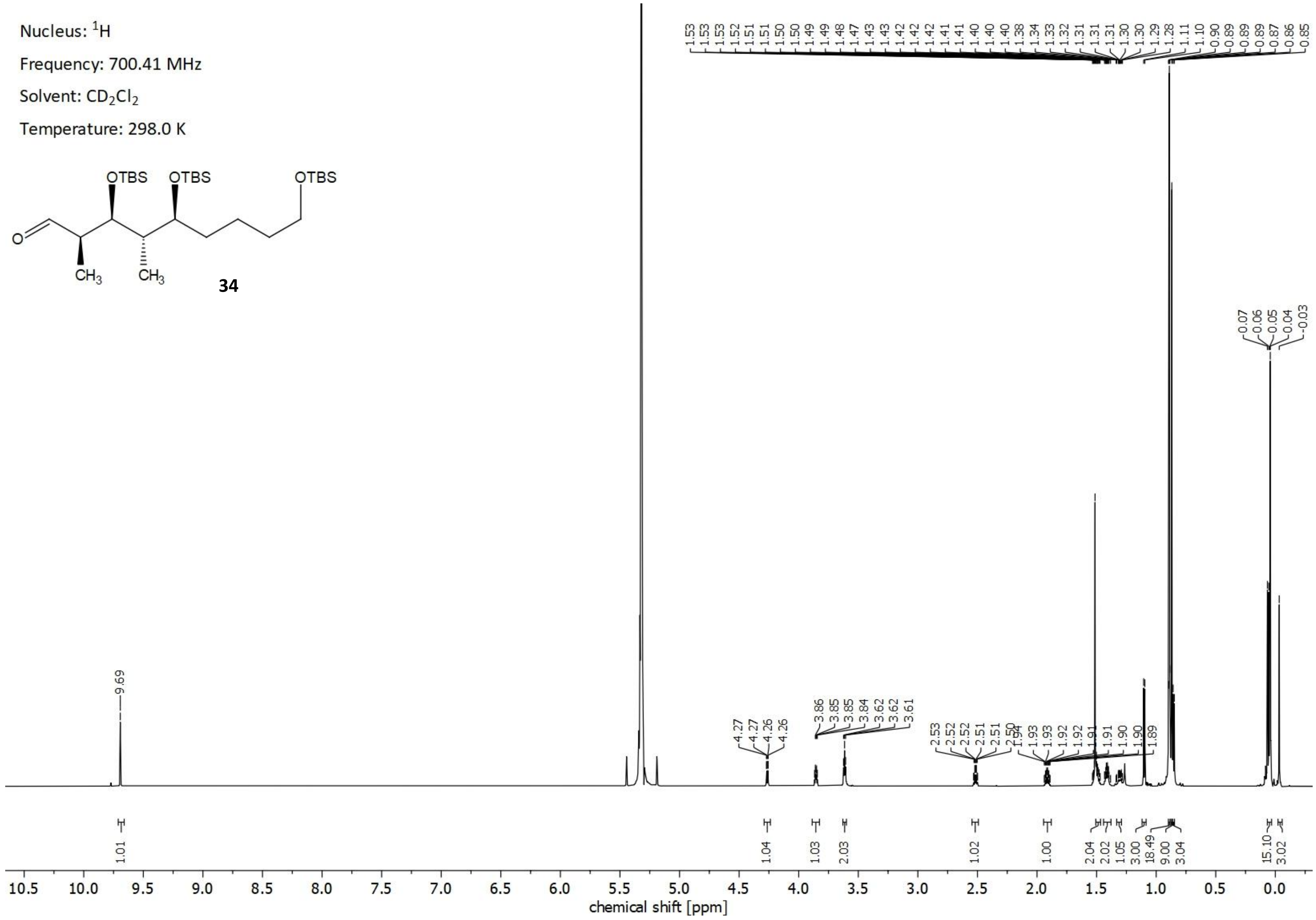

Nucleus: ^{13}C

Frequency: 176.14 MHz

Solvent: CD_2Cl_2

Temperature: 298.0 K

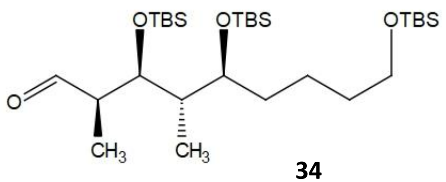

34

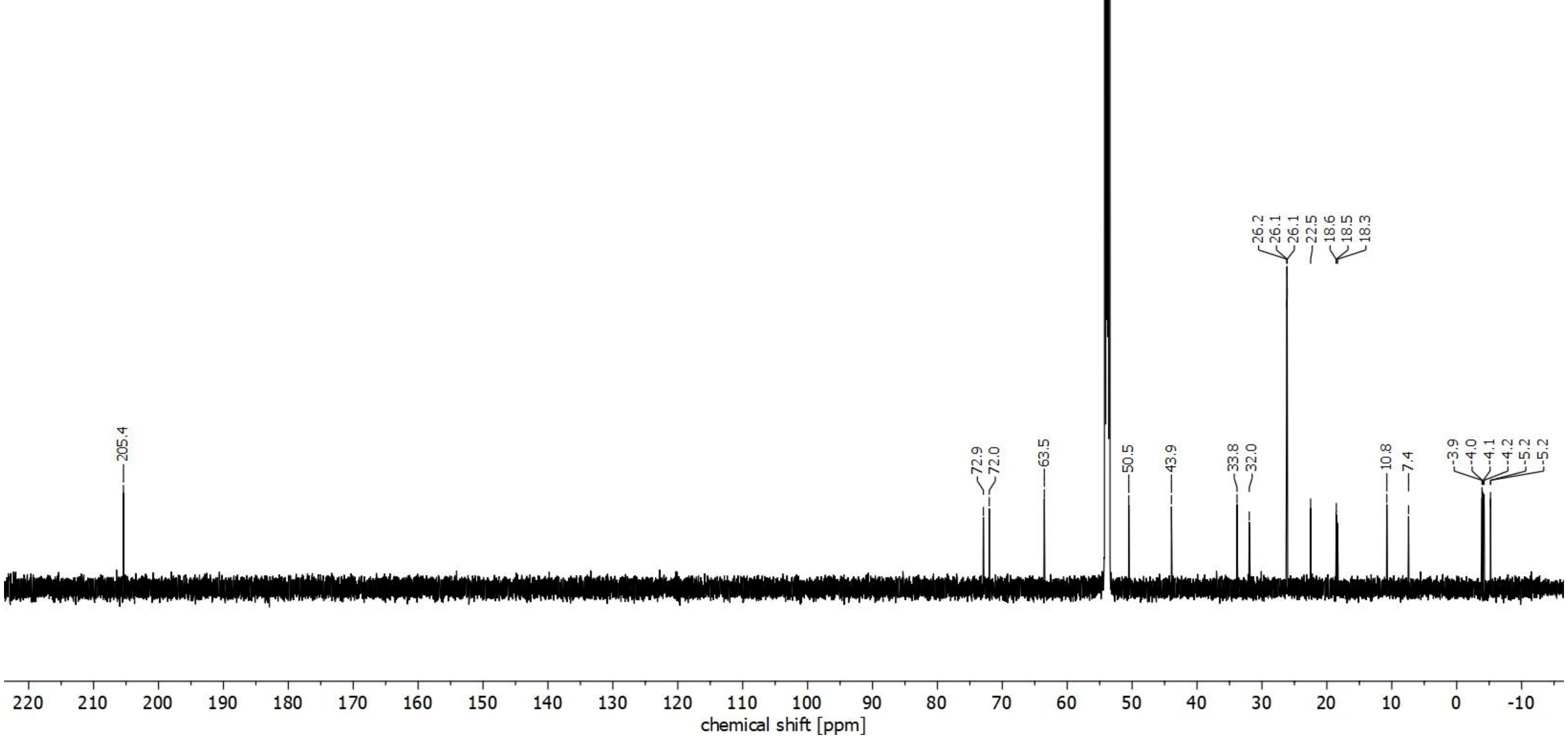

Nucleus: ^{1}H

Frequency: 500.04 MHz

Solvent: CD_2Cl_2

Temperature: 298.0 K

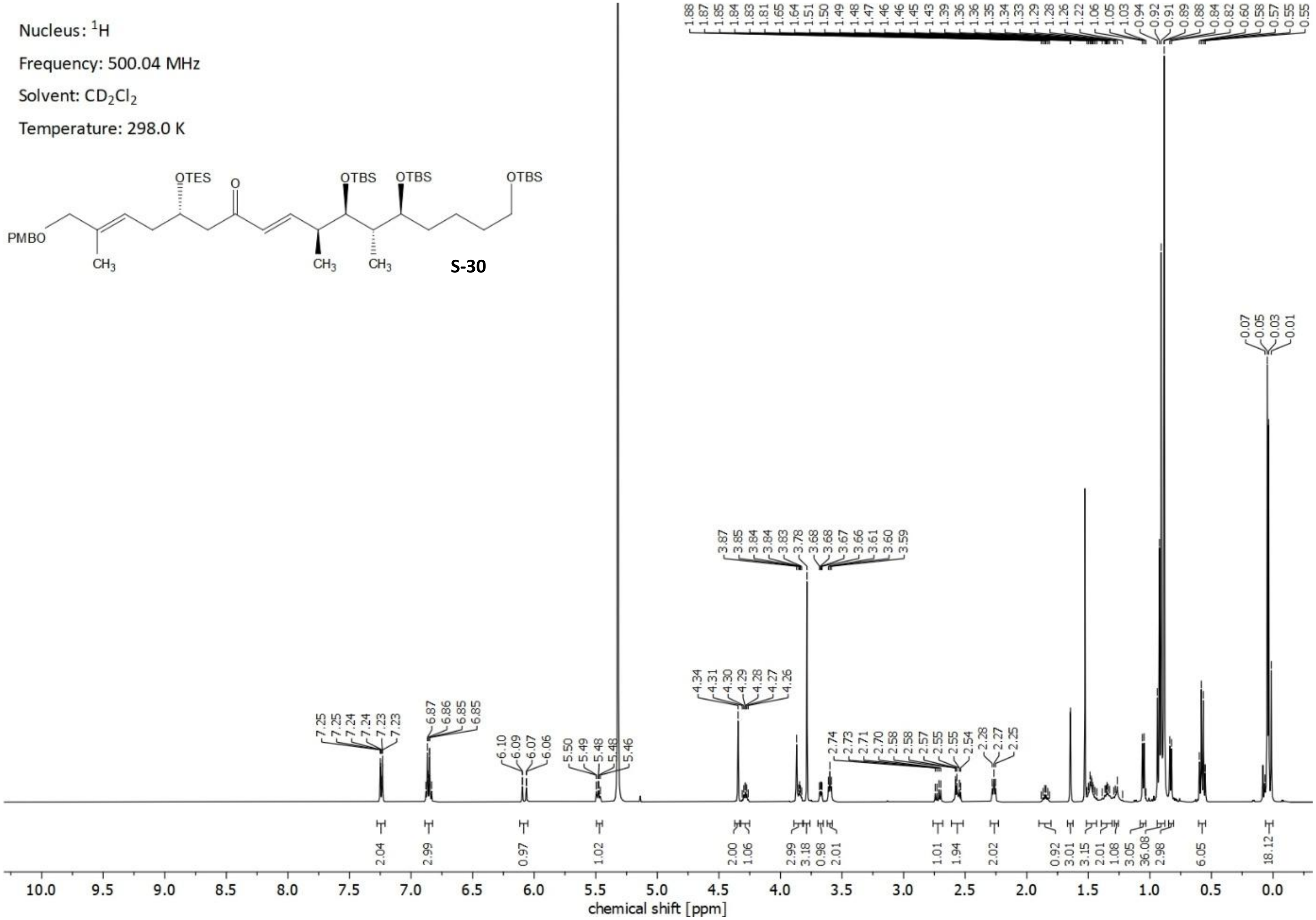

S-30

Nucleus: ^{13}C

Frequency: 125.75 MHz

Solvent: CD_2Cl_2

Temperature: 298.0 K

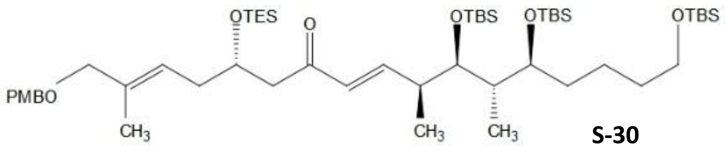

S-30

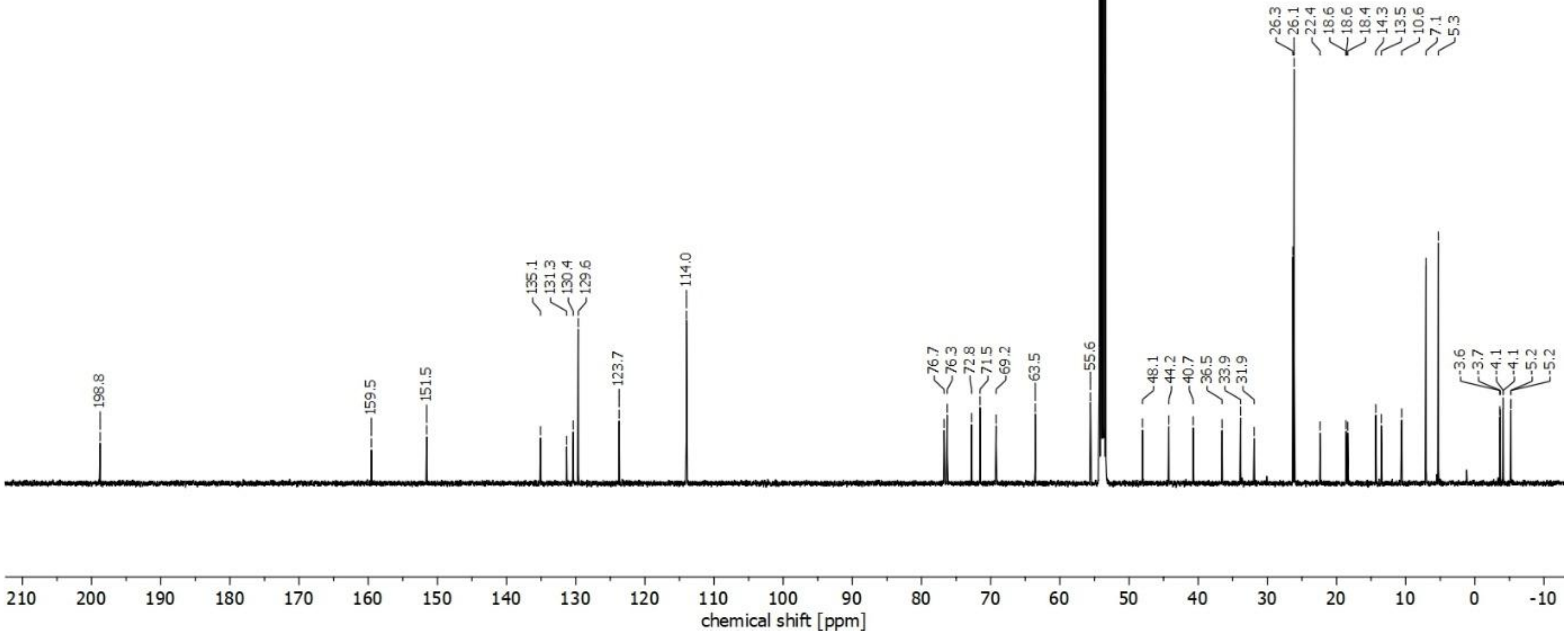

Nucleus: ^{1}H

Frequency: 500.04 MHz

Solvent: CD_2Cl_2

Temperature: 298.0 K

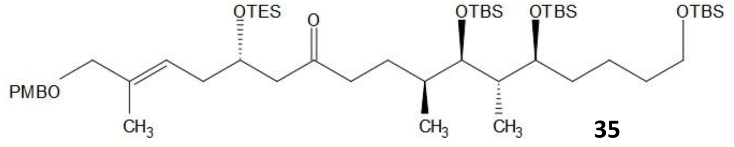

35

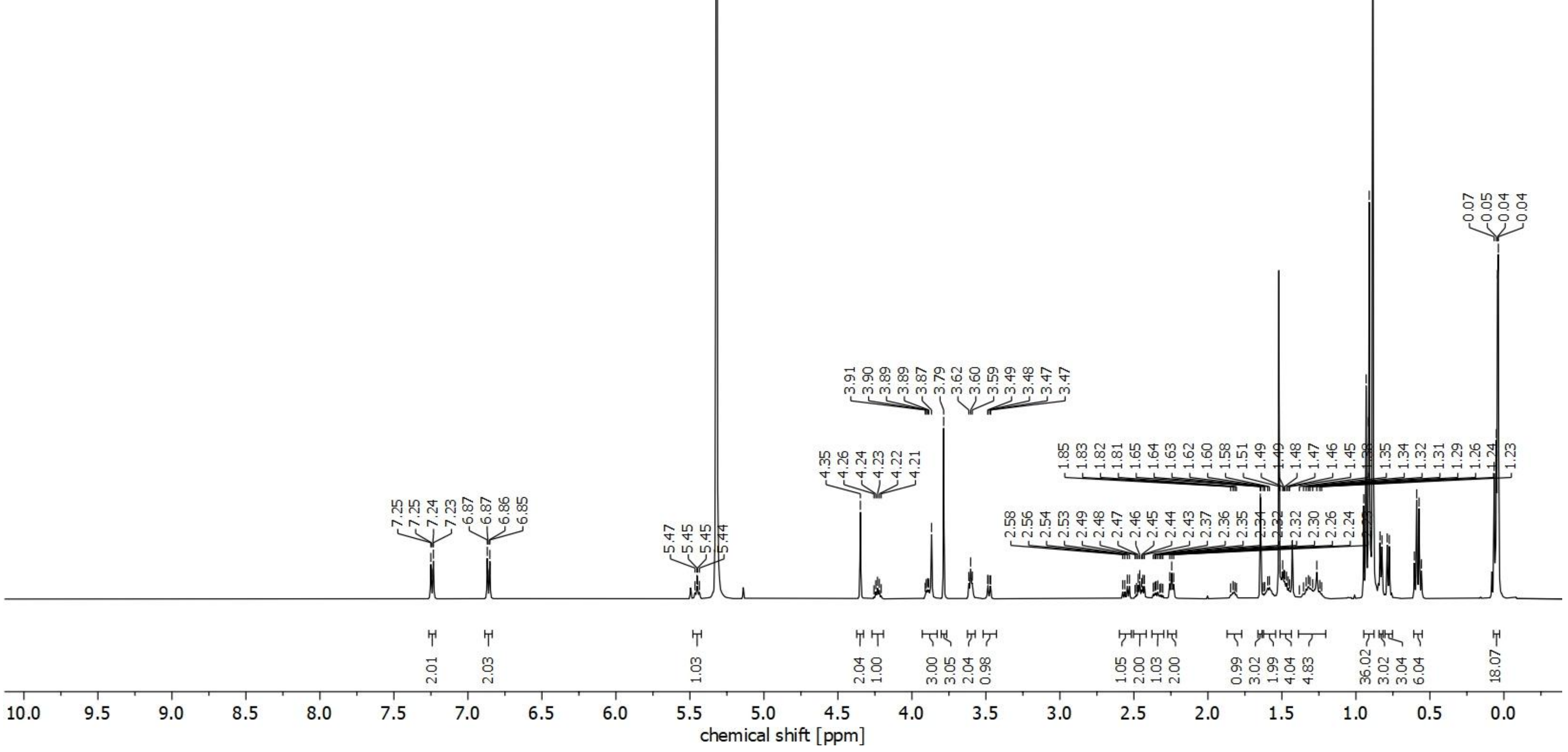

Nucleus: ^{13}C

Frequency: 125.75 MHz

Solvent: CD_2Cl_2

Temperature: 298.0 K

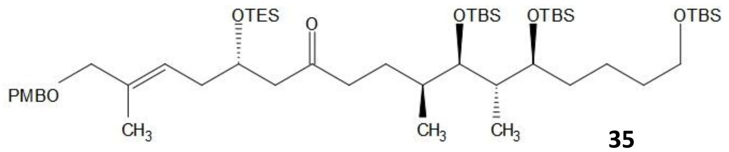

35

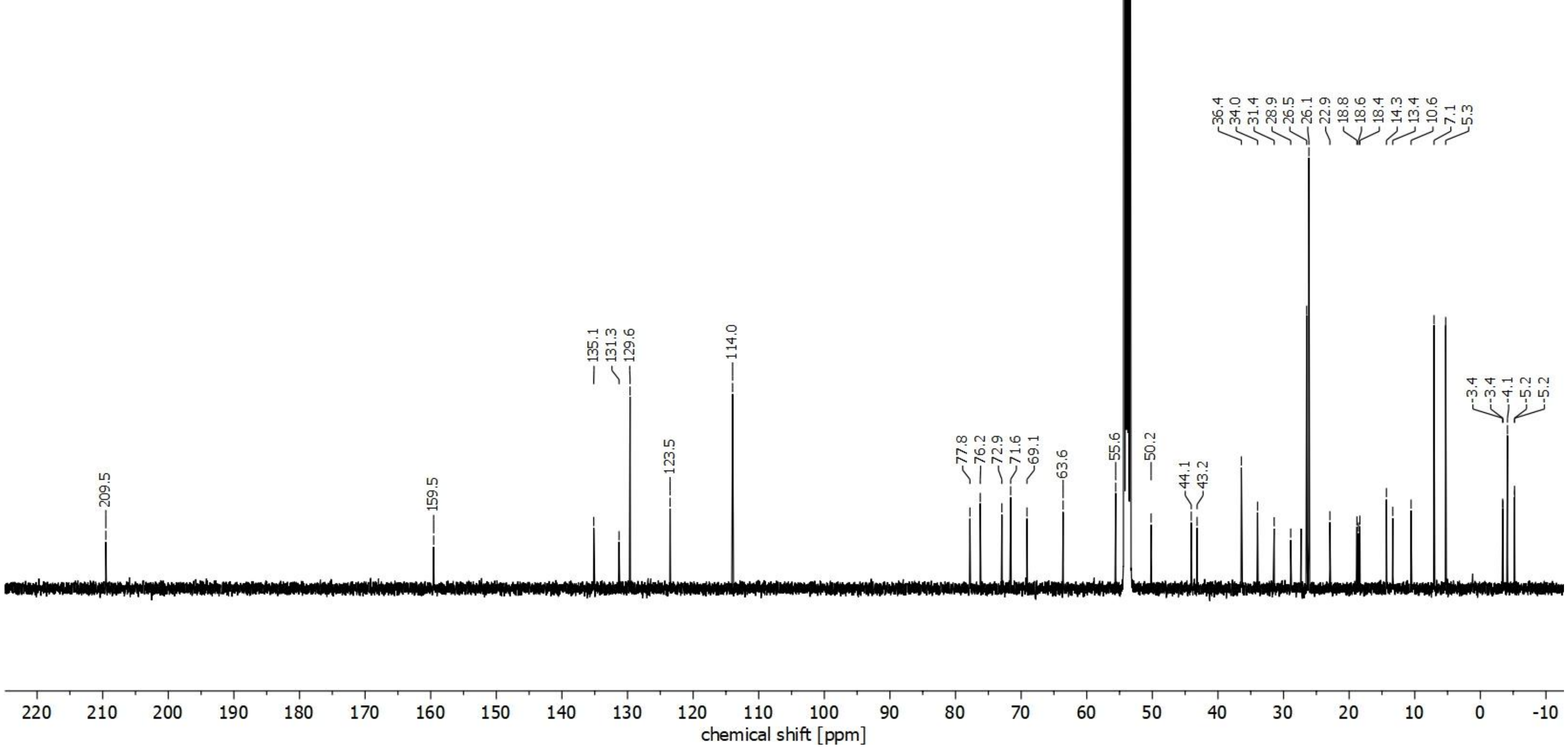

Nucleus: ^{1}H

Frequency: 700.41 MHz

Solvent: CD_2Cl_2

Temperature: 298.0 K

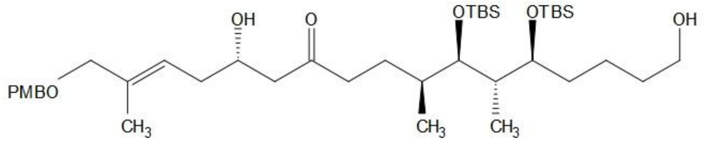

S-31

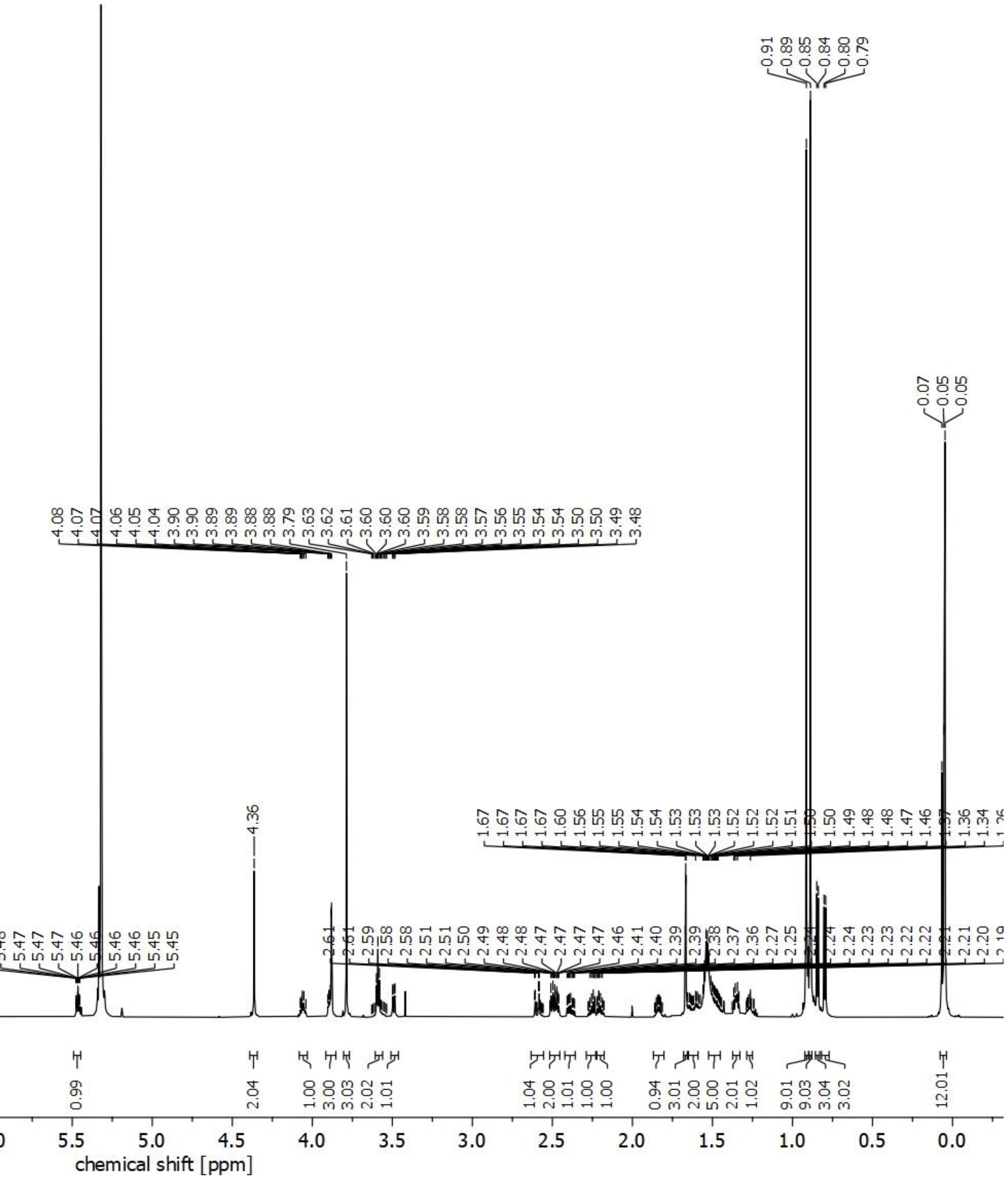

Nucleus: ^{13}C

Frequency: 176.14 MHz

Solvent: CD_2Cl_2

Temperature: 298.0 K

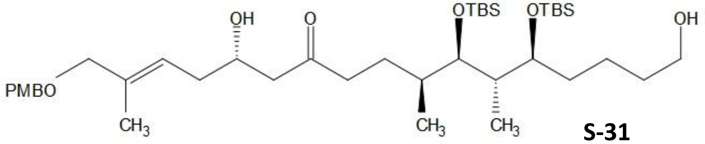

S-31

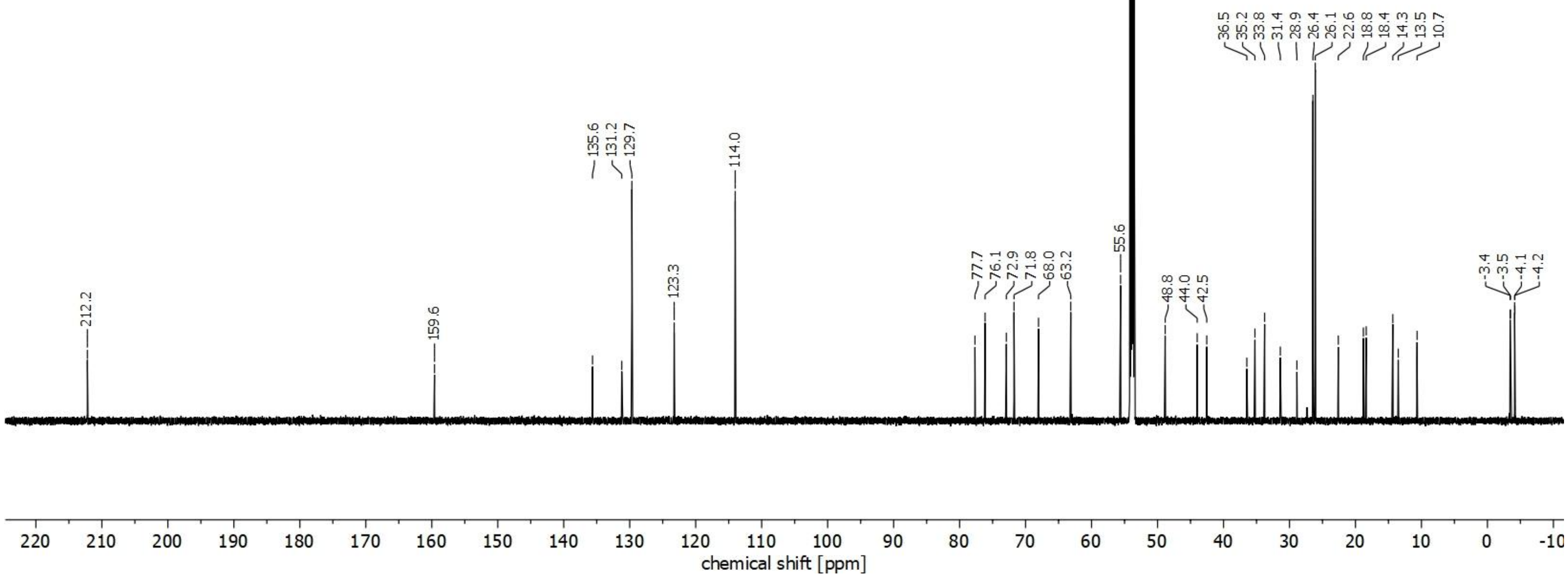

Nucleus: ^{1}H

Frequency: 700.41 MHz

Solvent: CD_2Cl_2

Temperature: 298.0 K

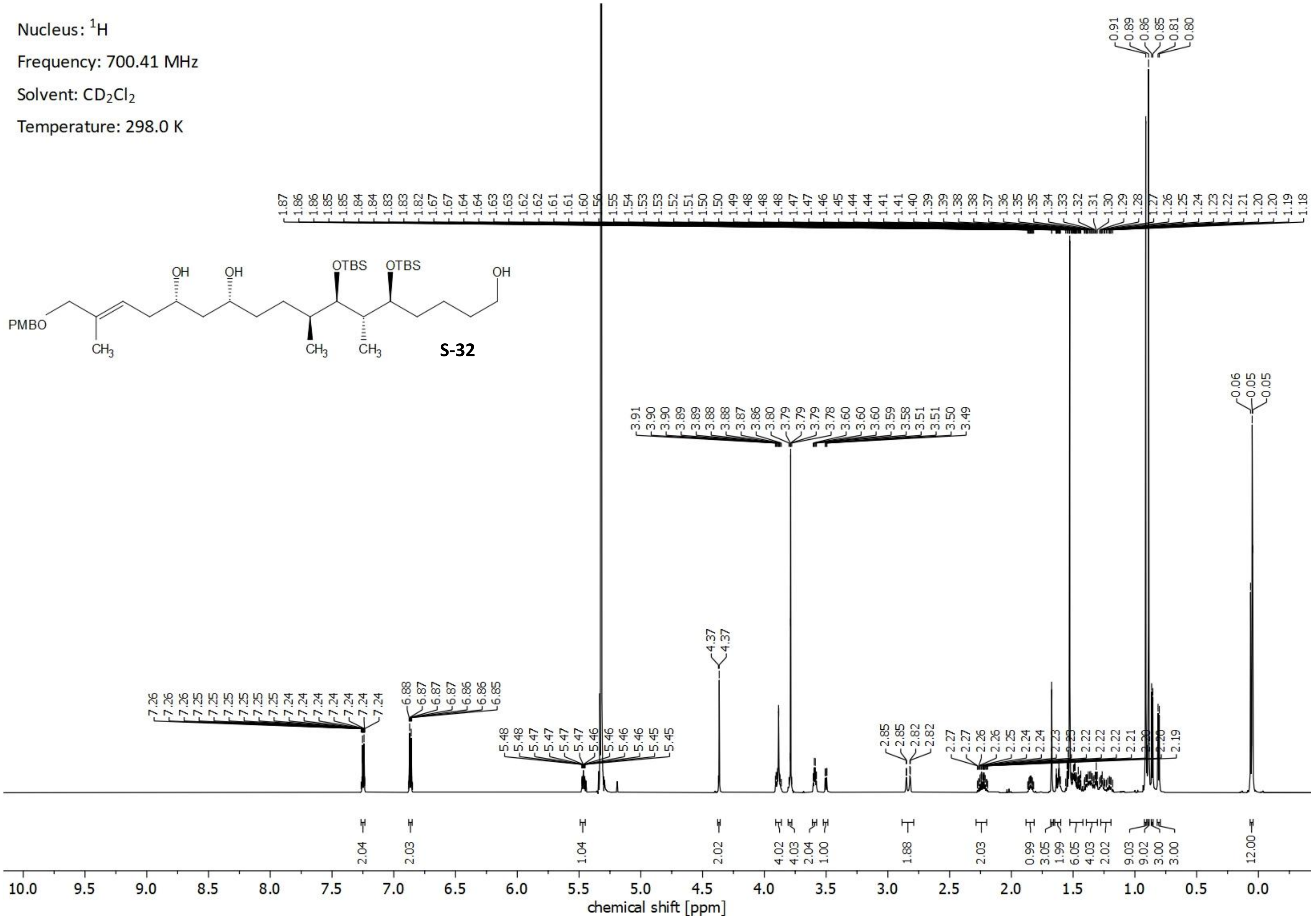

S-32

Nucleus: ^{13}C

Frequency: 176.14 MHz

Solvent: CD_2Cl_2

Temperature: 298.0 K

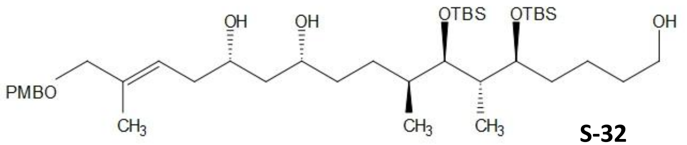

S-32

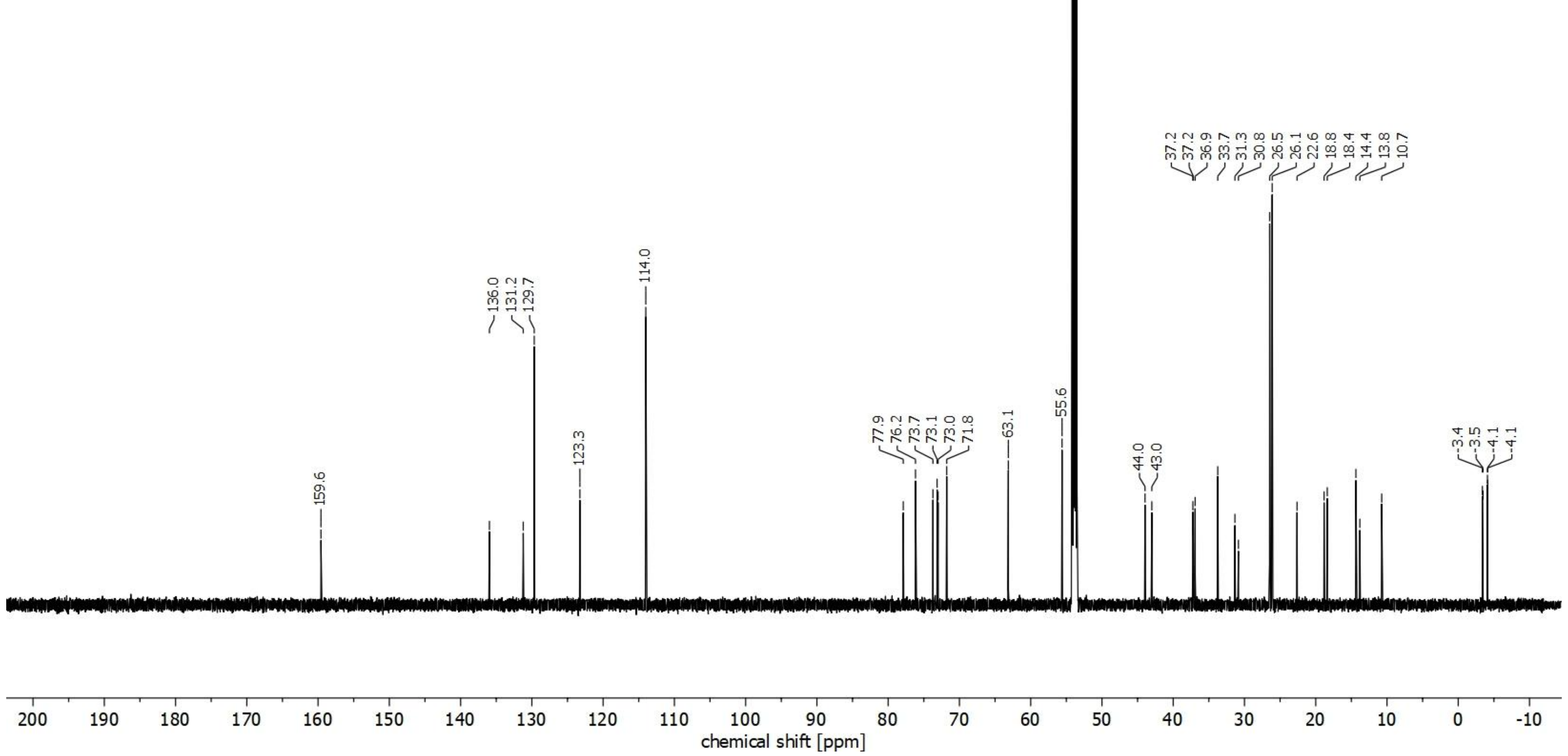

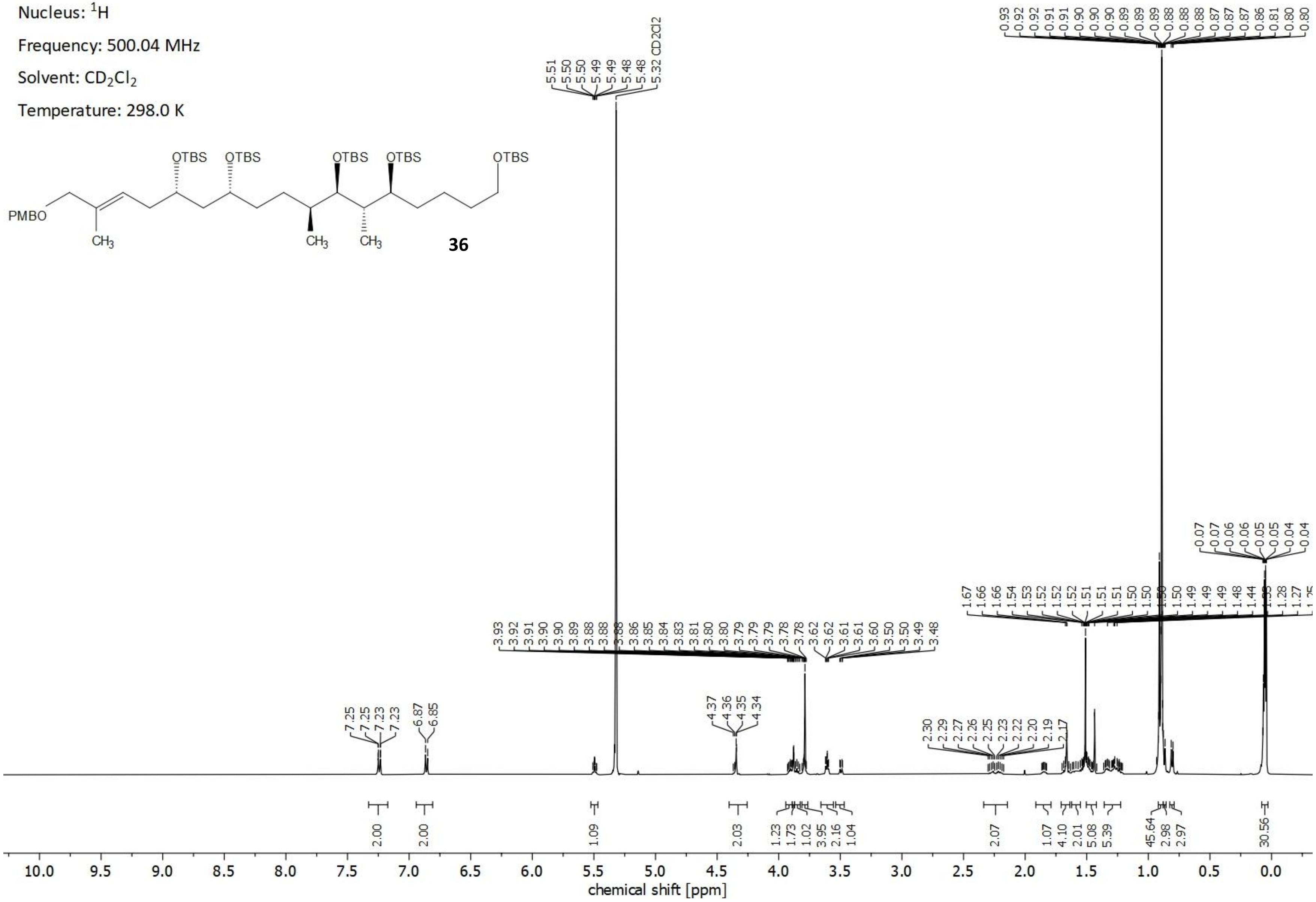
Nucleus: ^1H
Frequency: 500.04 MHz
Solvent: CD_2Cl_2
Temperature: 298.0 K
36
chemical shift [ppm]

Nucleus: ^{13}C

Frequency: 125.75 MHz

Solvent: CD_2Cl_2

Temperature: 298.0 K

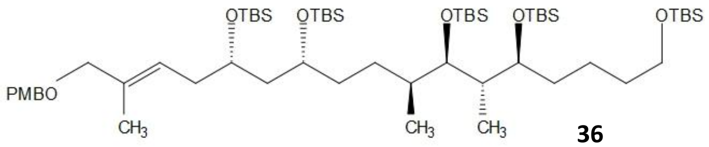

36

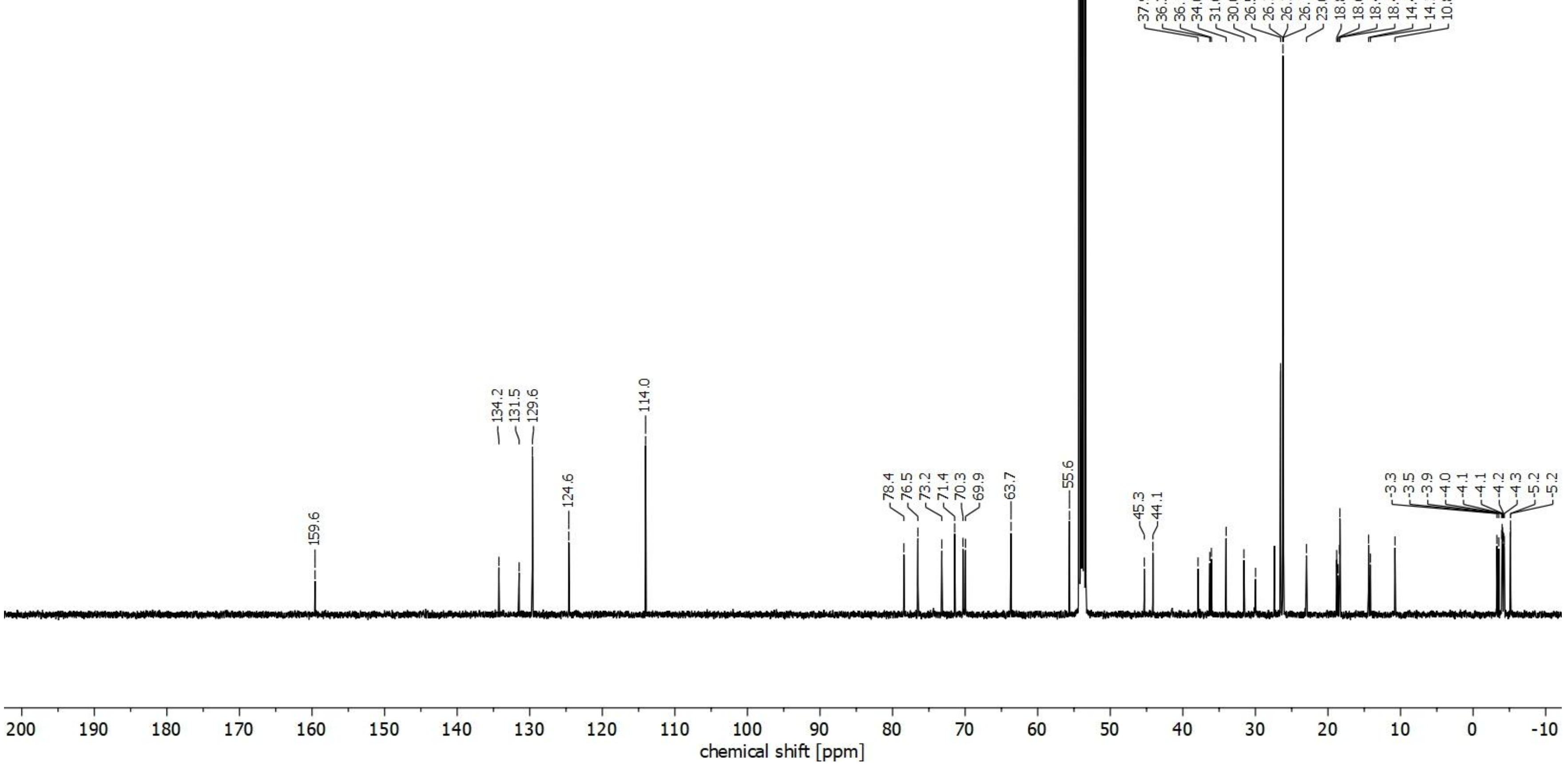

Nucleus: ^{1}H

Frequency: 500.04 MHz

Solvent: CD_2Cl_2

Temperature: 298.0 K

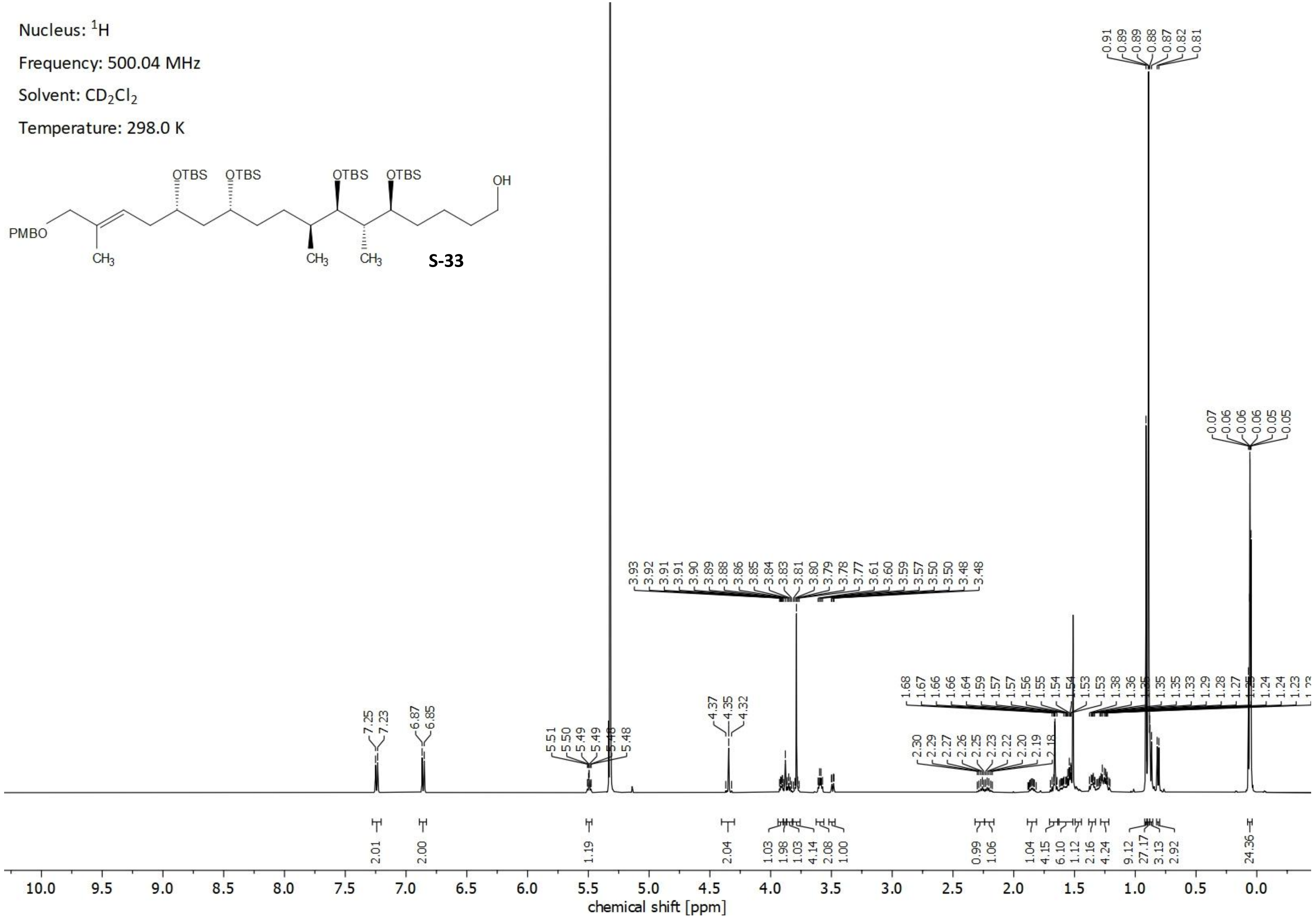

Nucleus: ^{13}C

Frequency: 125.75 MHz

Solvent: CD_2Cl_2

Temperature: 298.0 K

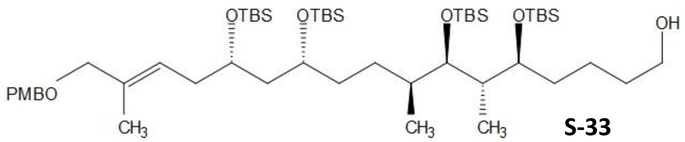

S-33

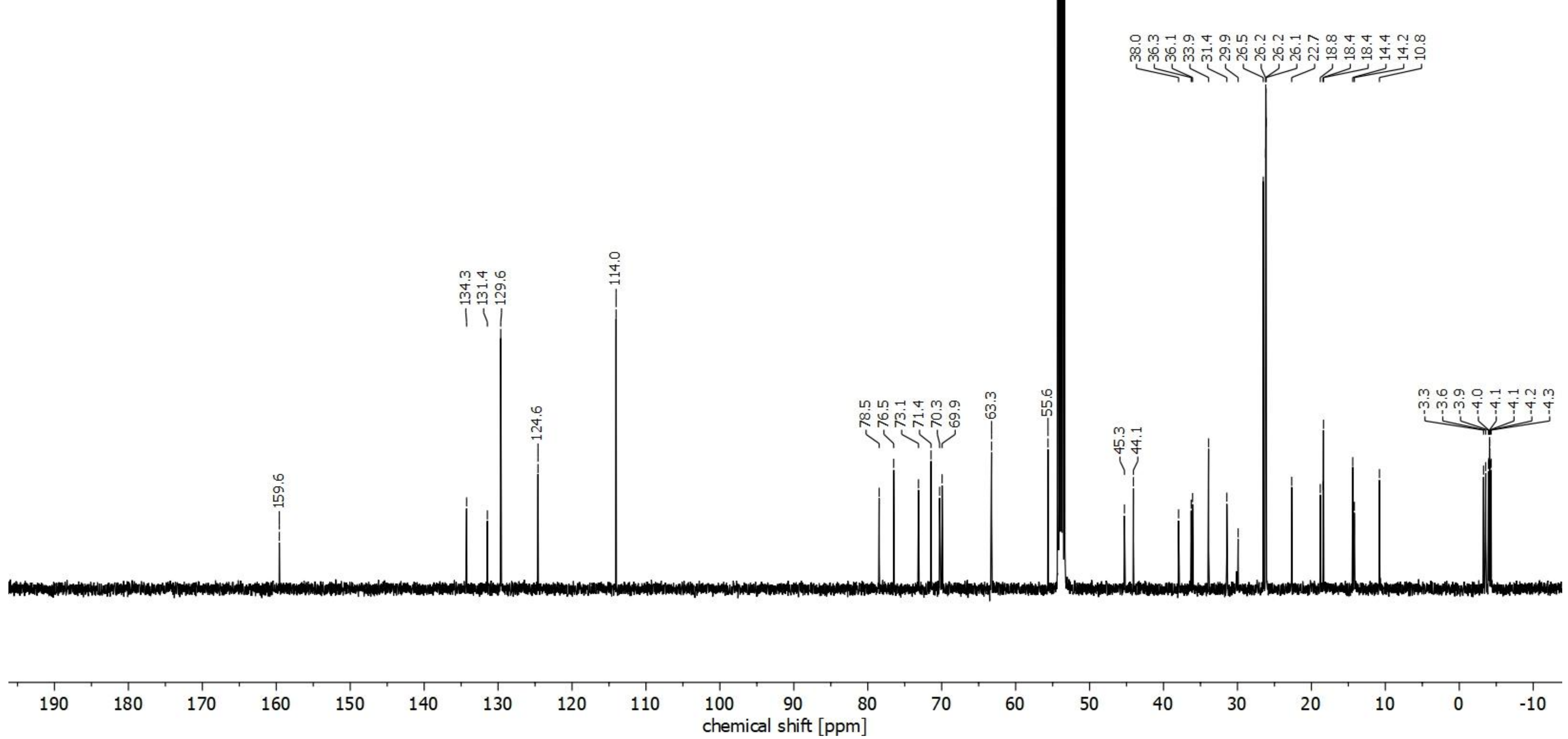

Nucleus: ^{1}H

Frequency: 500.04 MHz

Solvent: CD_2Cl_2

Temperature: 298.0 K

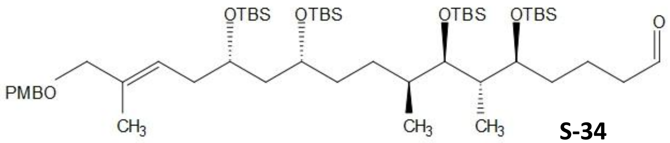

S-34

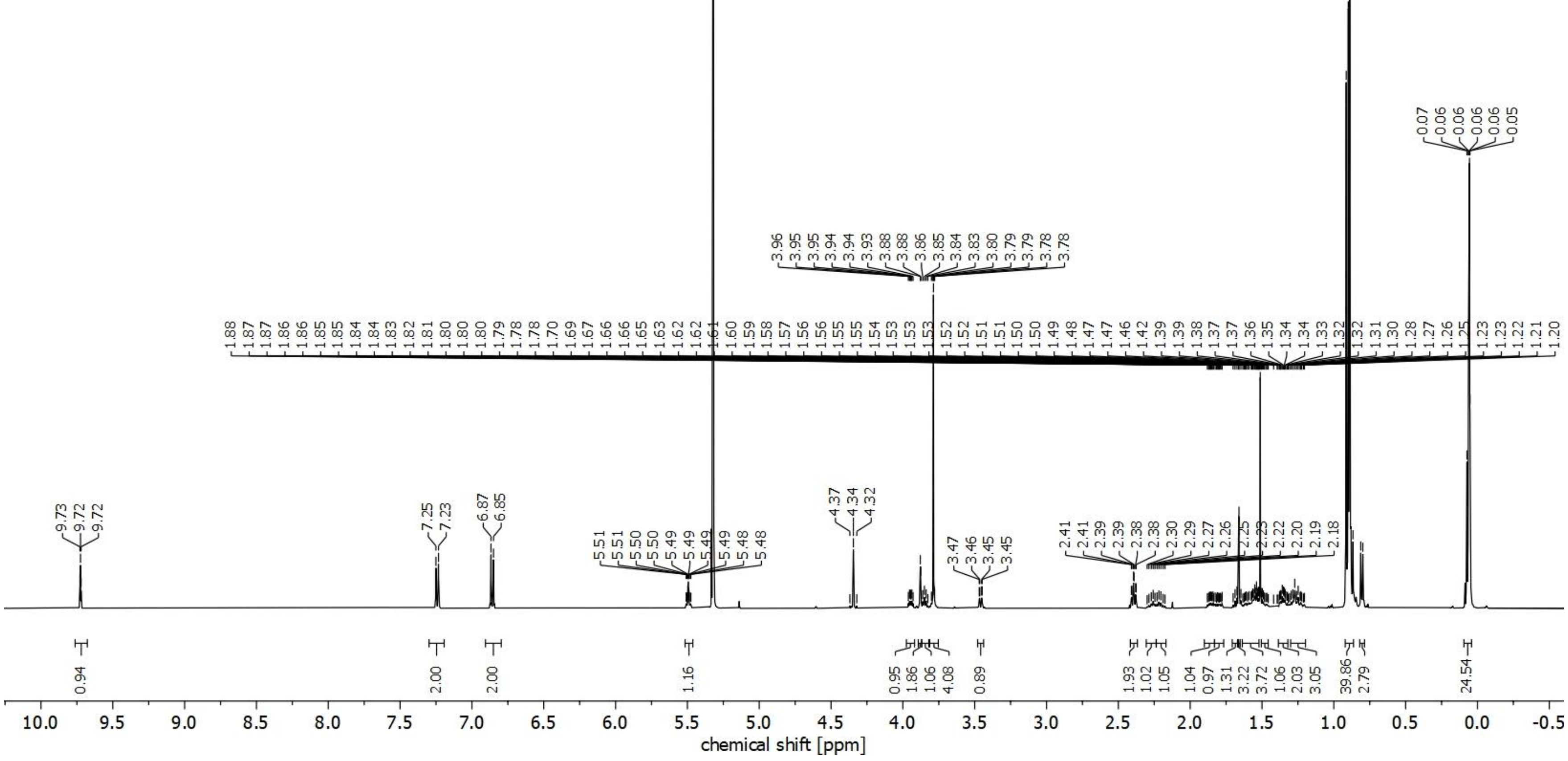

Nucleus: ^{13}C

Frequency: 125.75 MHz

Solvent: CD_2Cl_2

Temperature: 298.0 K

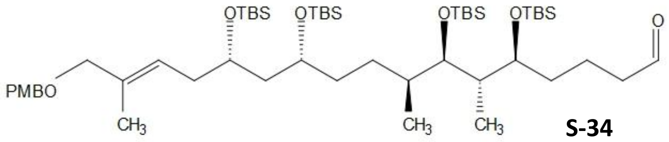

S-34

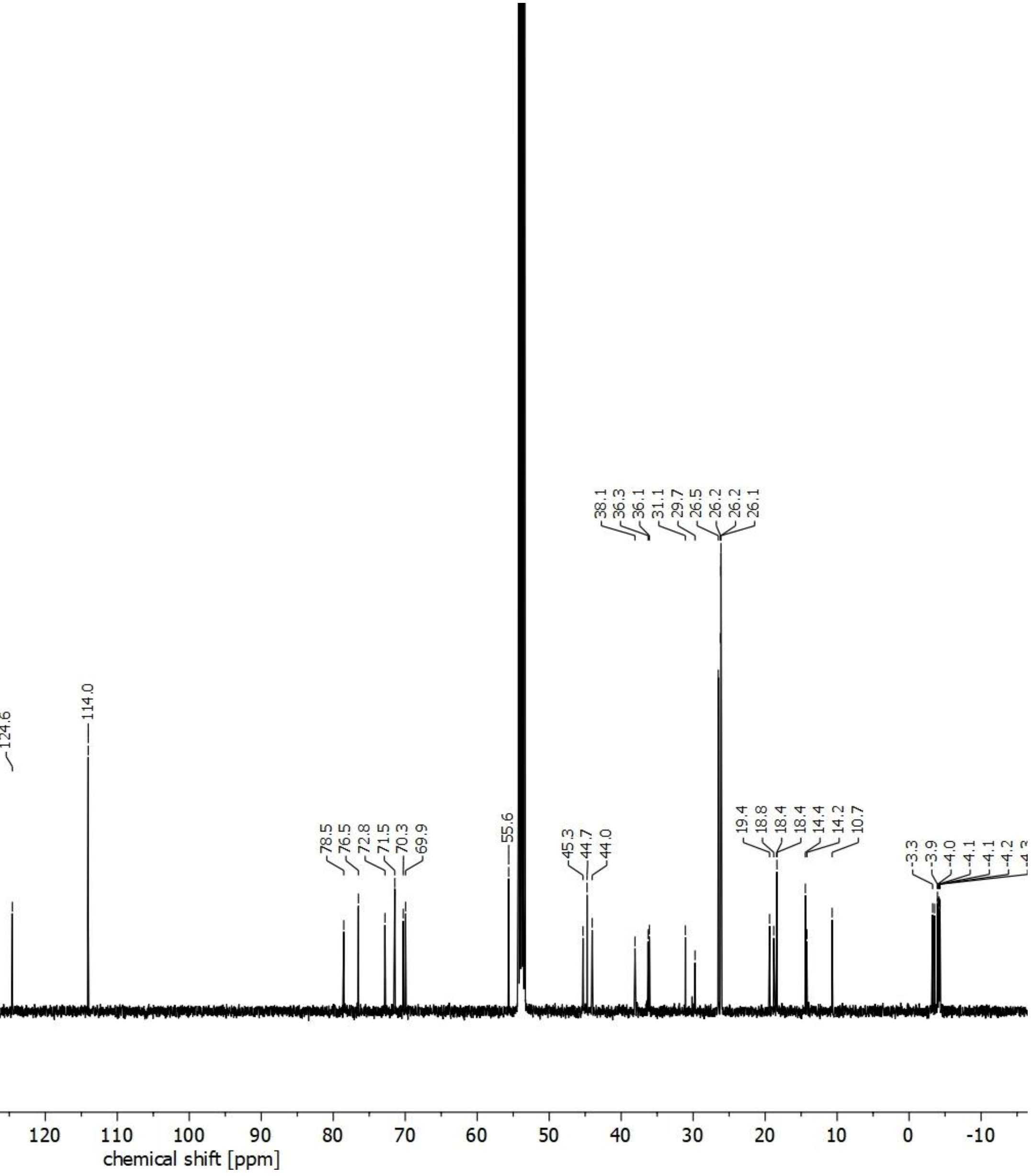

Nucleus: ^{1}H

Frequency: 700.41 MHz

Solvent: CD_2Cl_2

Temperature: 298.0 K

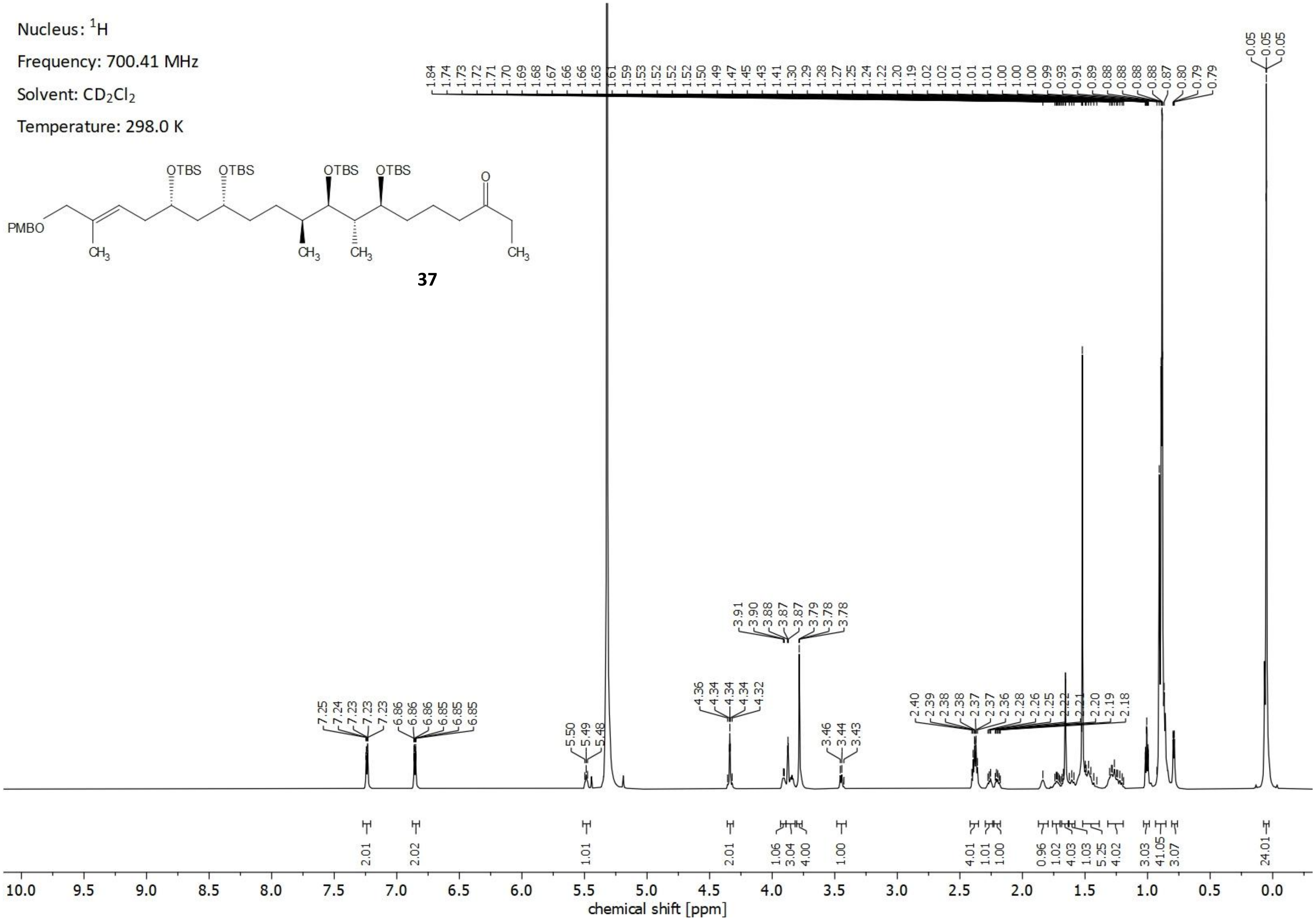

37

Nucleus: ^{13}C

Frequency: 176.14 MHz

Solvent: CD_2Cl_2

Temperature: 298.0 K

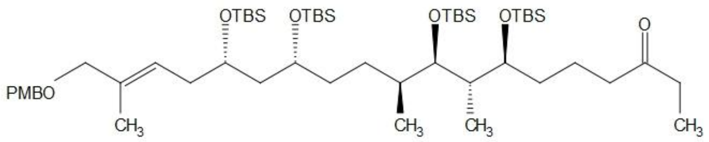

37

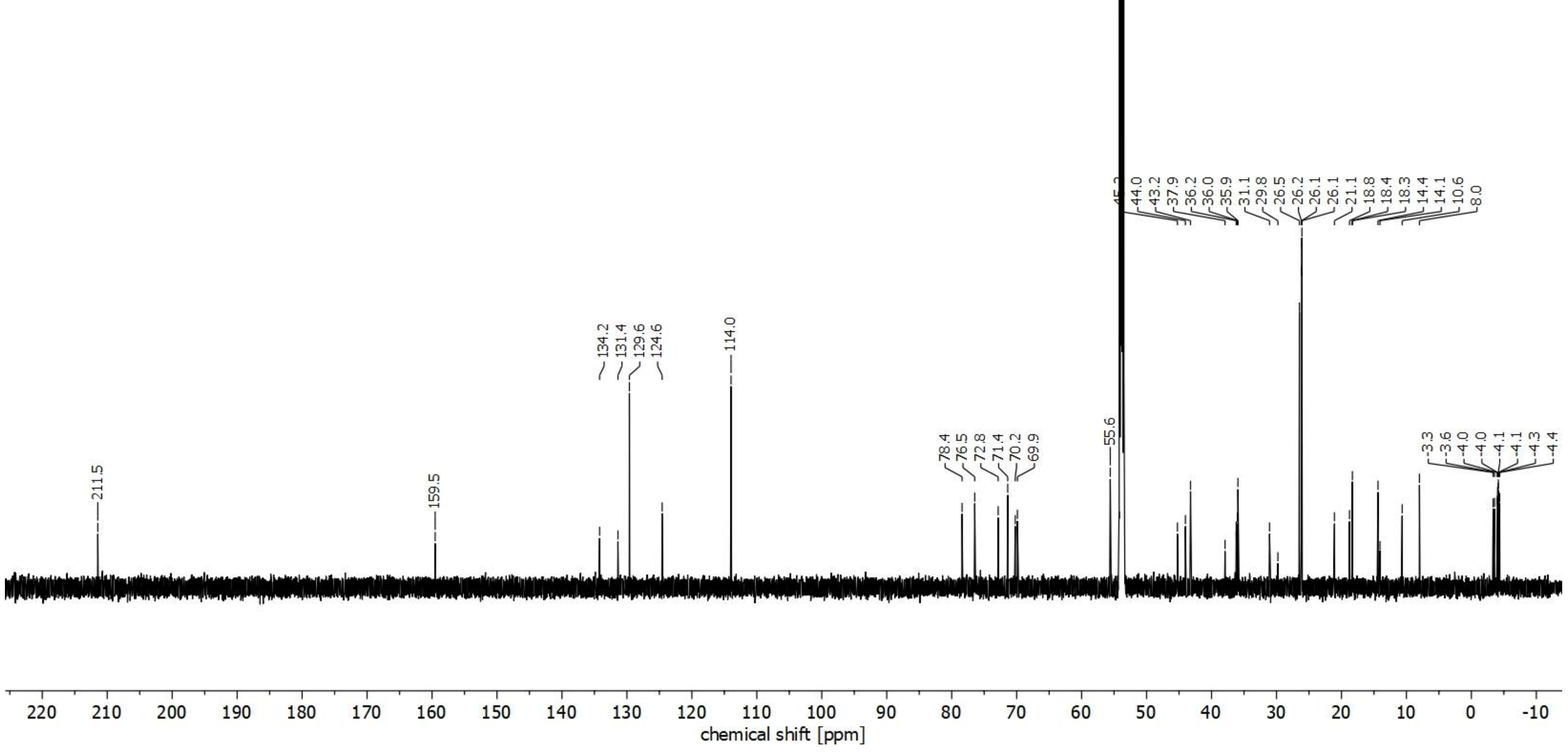

Nucleus: ^{1}H

Frequency: 700.41 MHz

Solvent: CD_2Cl_2

Temperature: 298.0 K

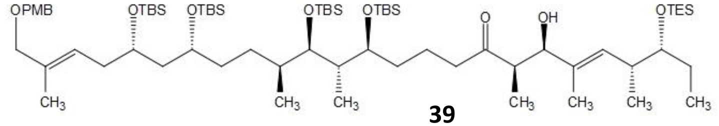

39

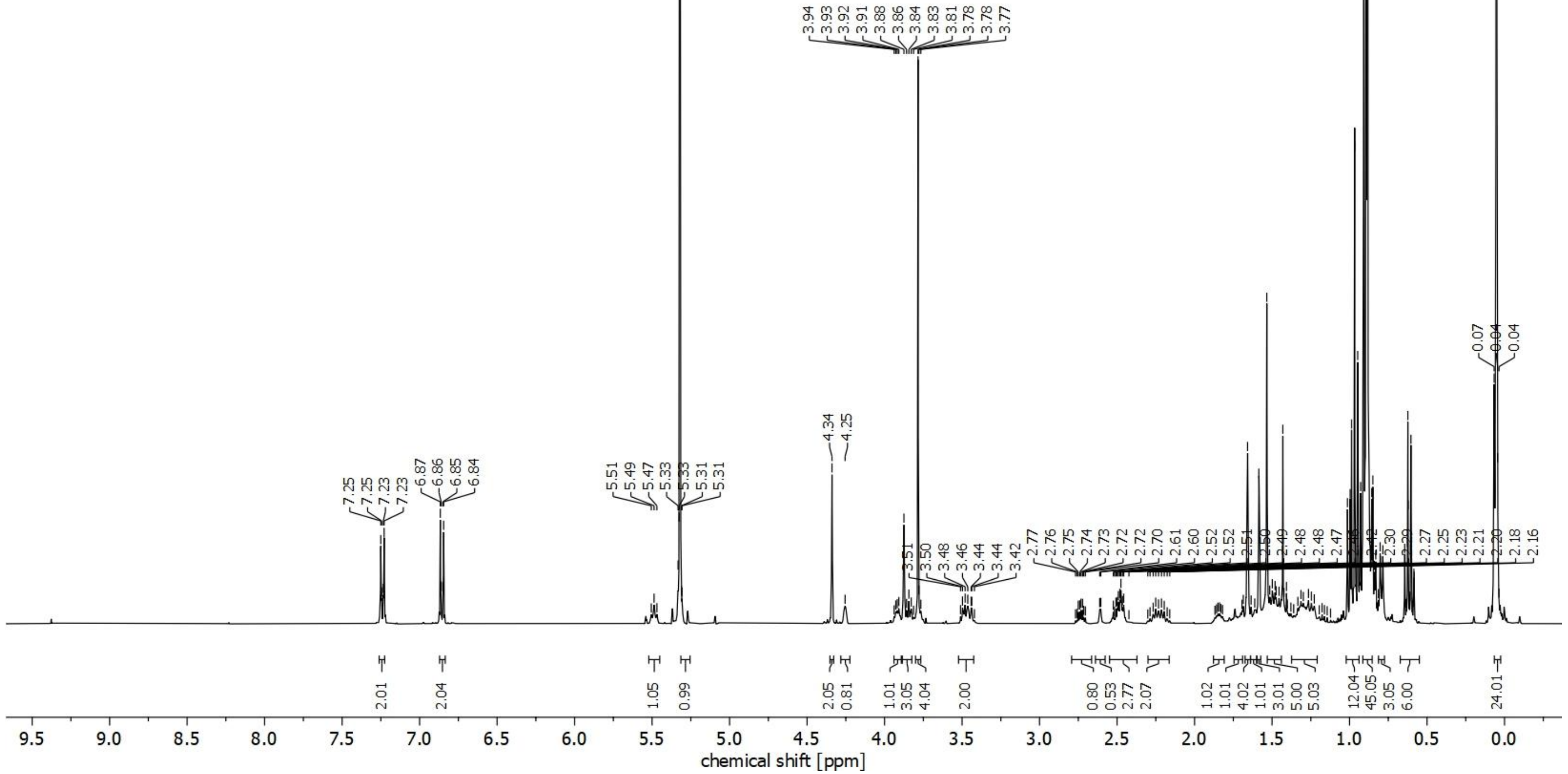

Nucleus: ^{13}C

Frequency: 176.14 MHz

Solvent: CD_2Cl_2

Temperature: 298.0 K

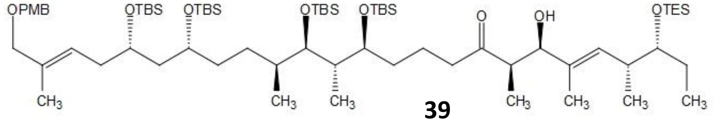

39

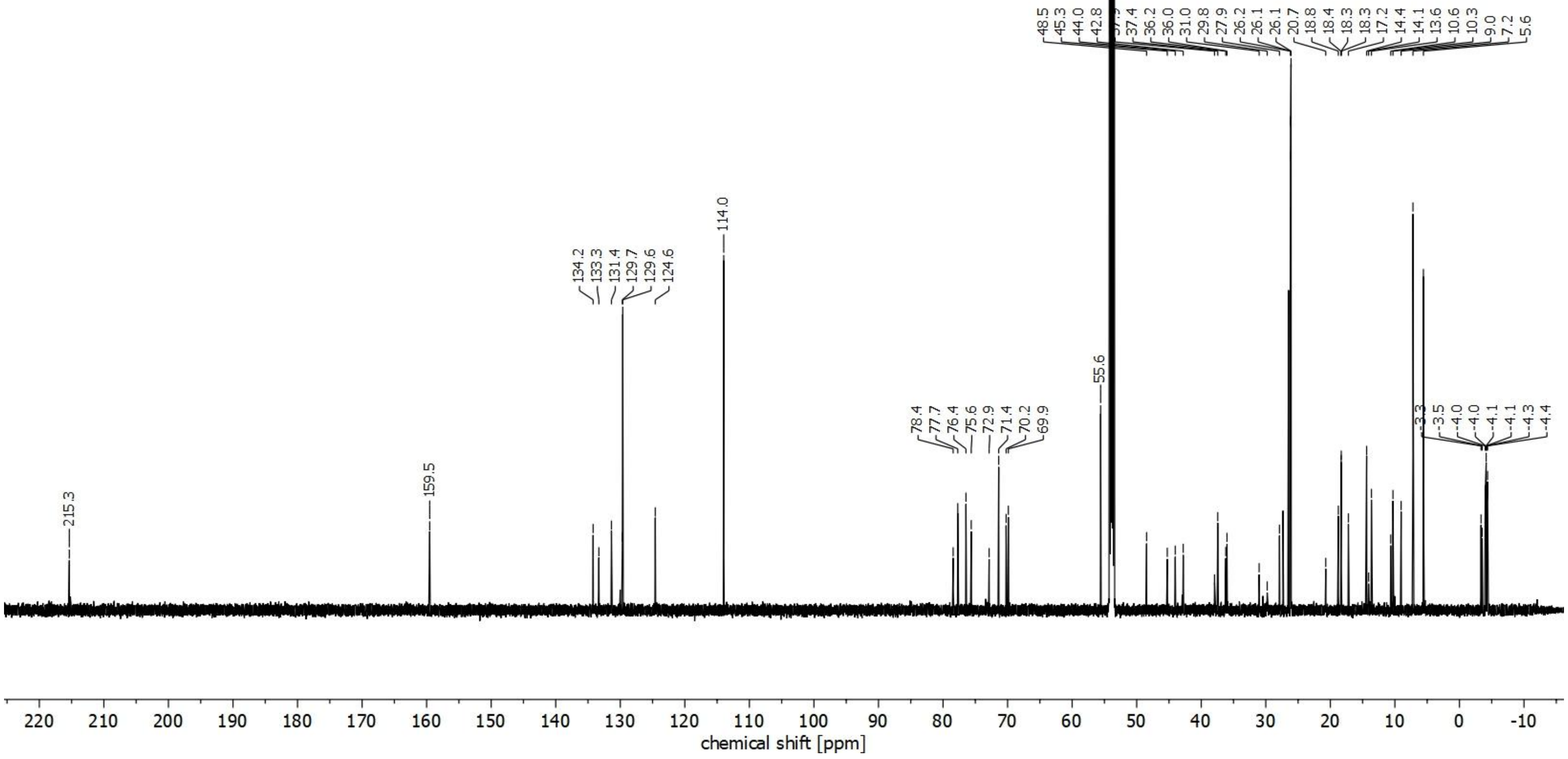

^{1}H-NMR, 700 MHz, CD_2Cl_2, 298 K

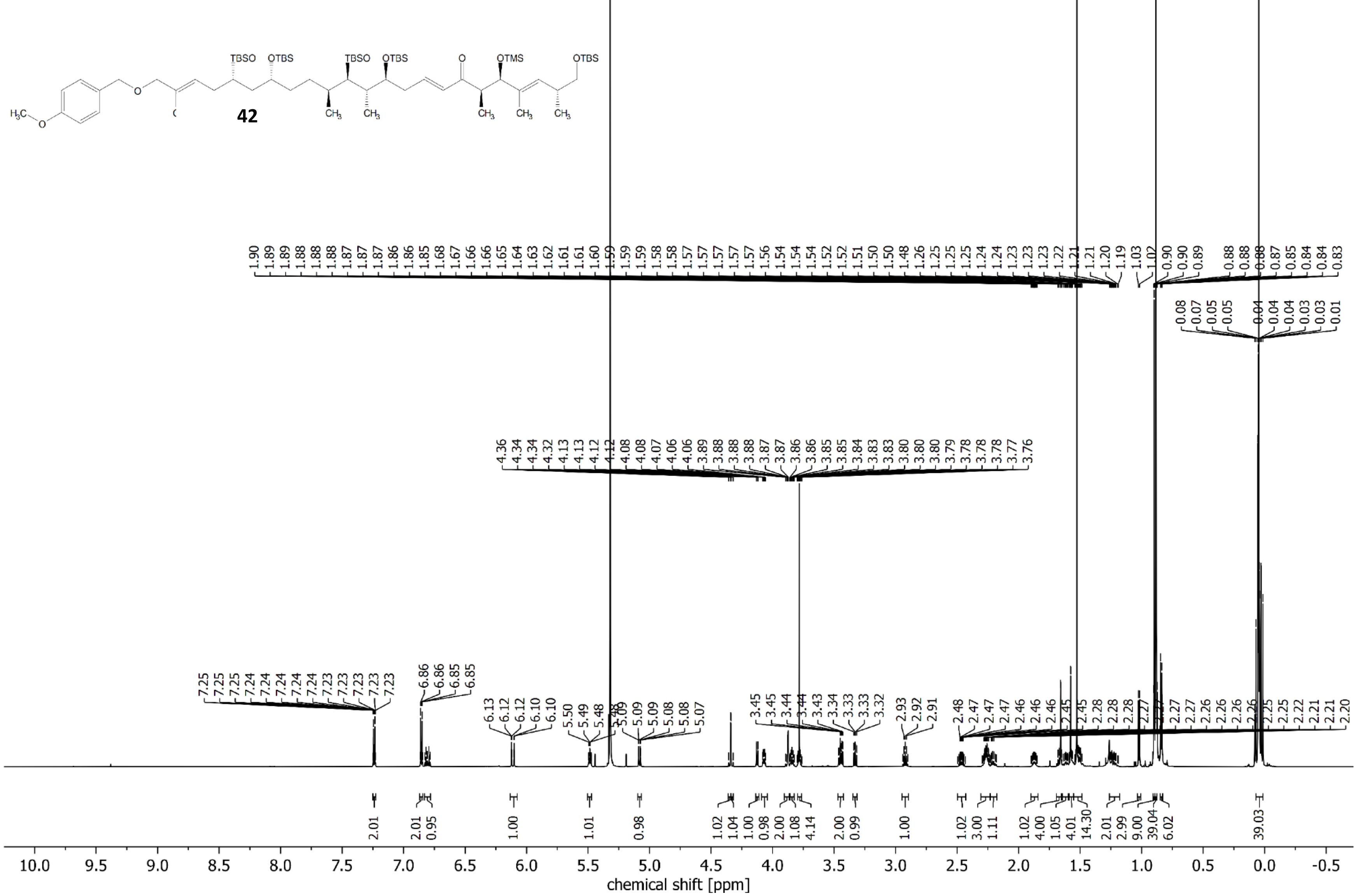

^{13}C-NMR, 176 MHz, CD_2Cl_2, 298 K

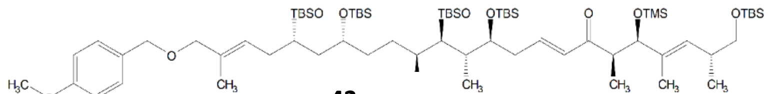

42

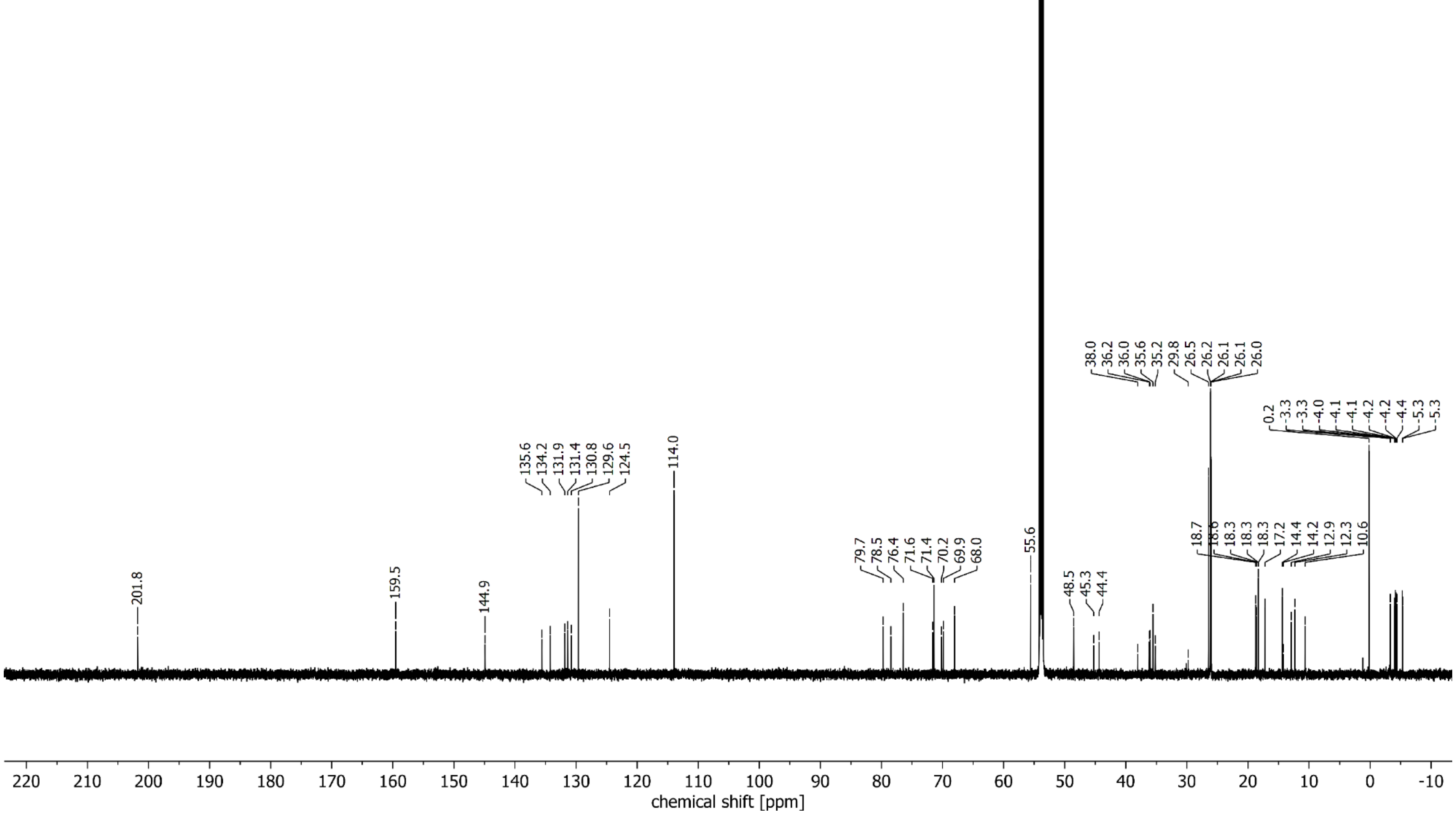

^{1}H-NMR, 500 MHz, CD_2Cl_2, 298 K

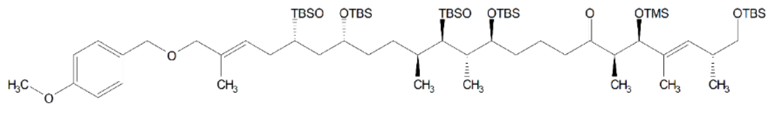

S-35

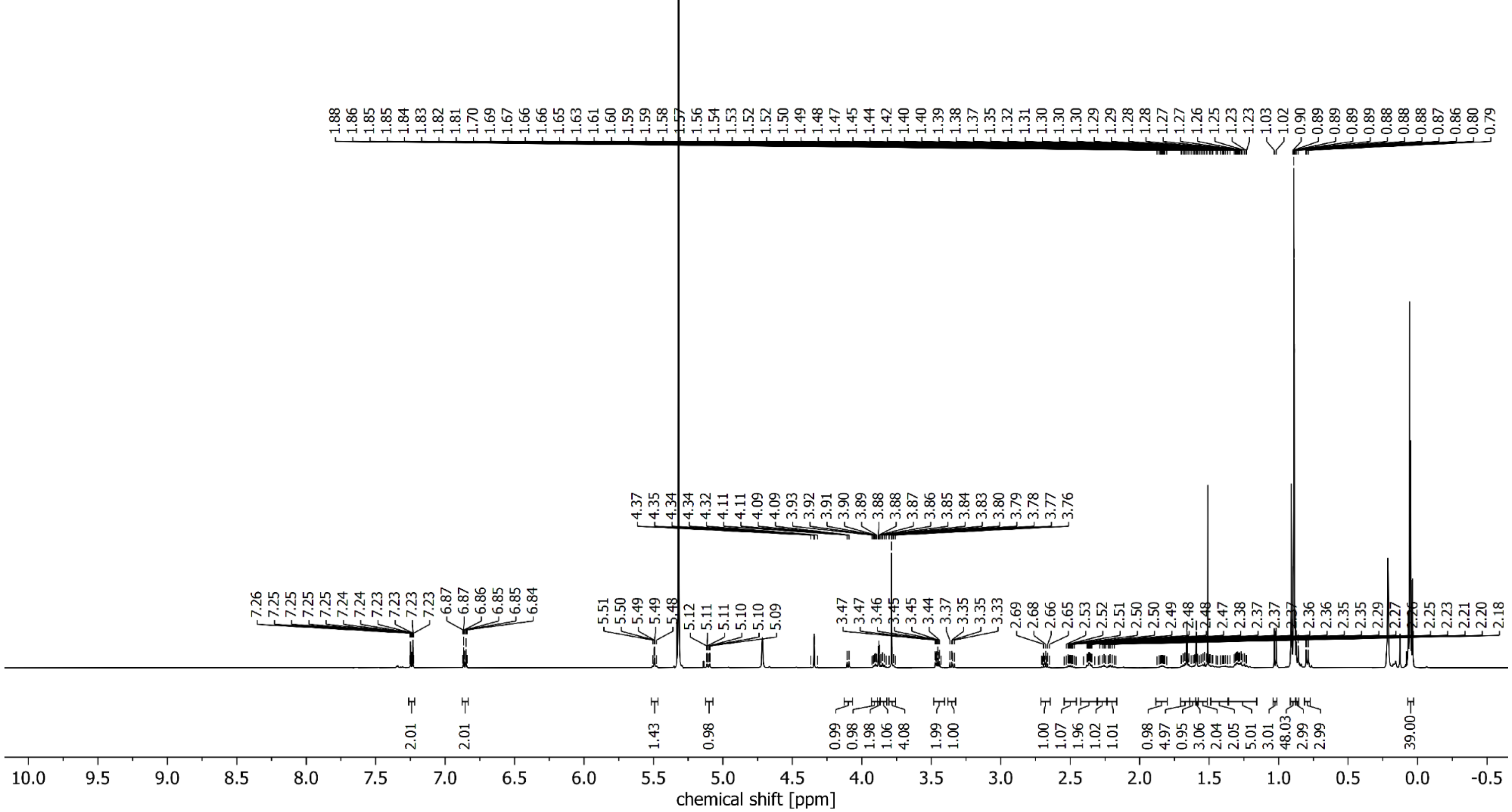

^{13}C-NMR, 126 MHz, CD_2Cl_2, 298 K

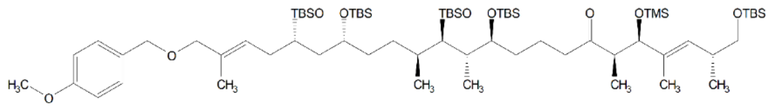

S-35

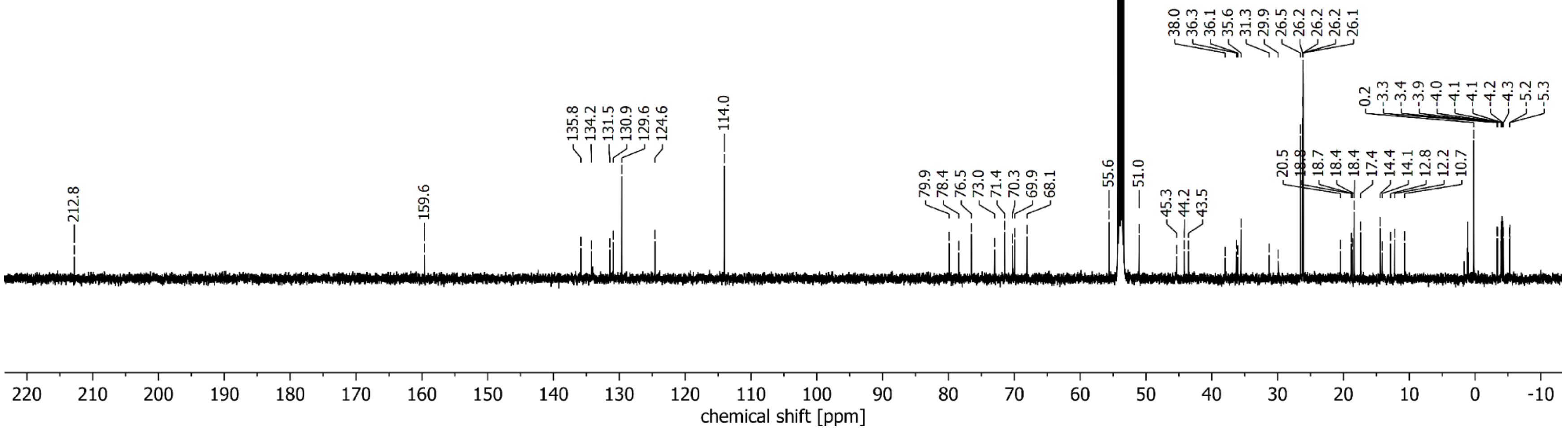

^{1}H-NMR, 500 MHz, CD_2Cl_2, 298 K

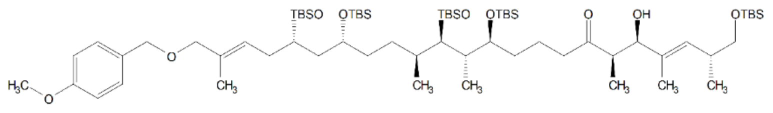

S-36

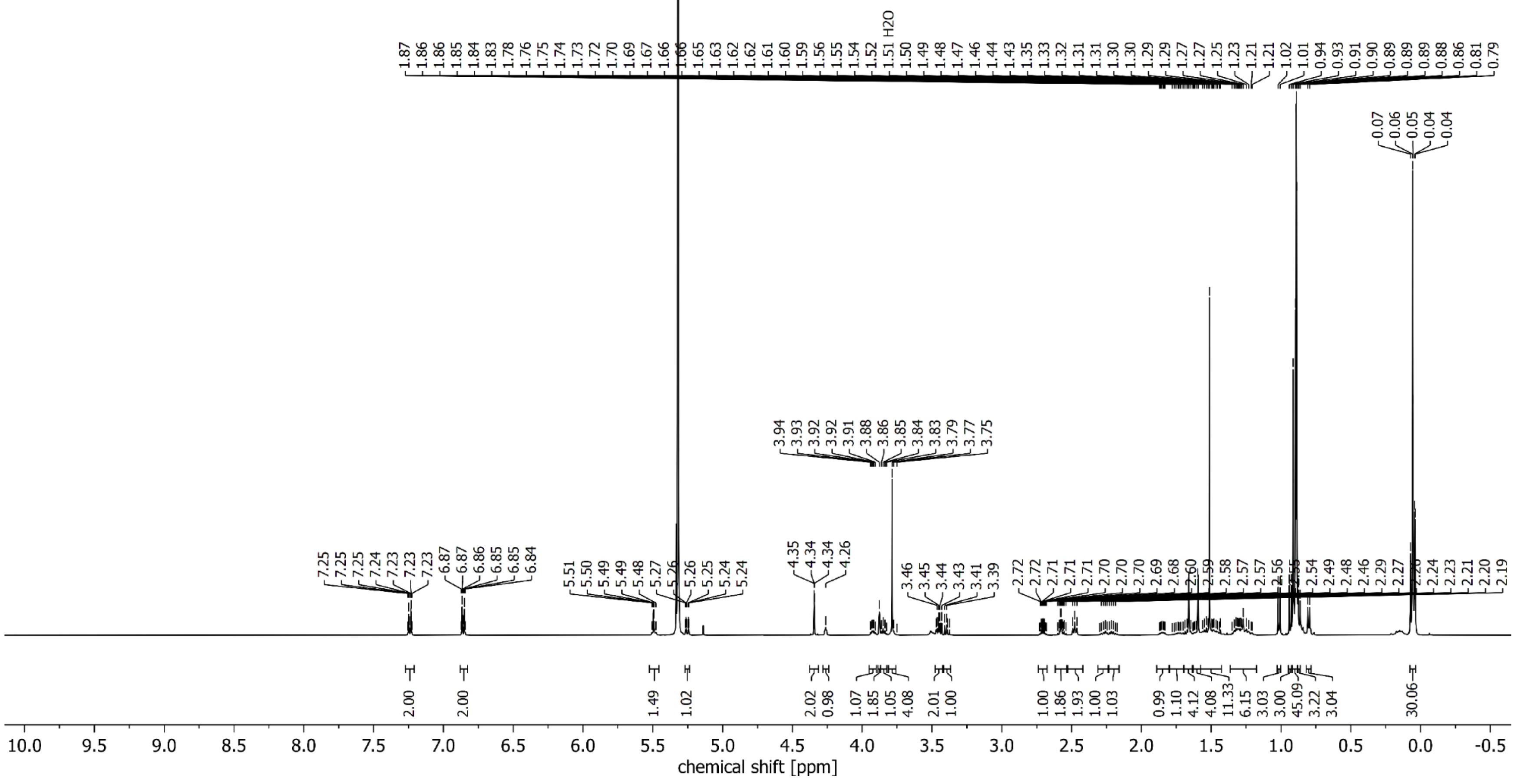

^{13}C-NMR, 126 MHz, CD_2Cl_2, 298 K

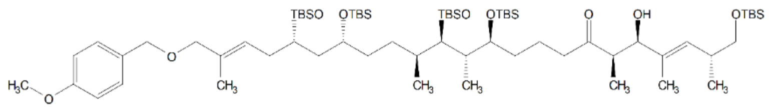

S-36

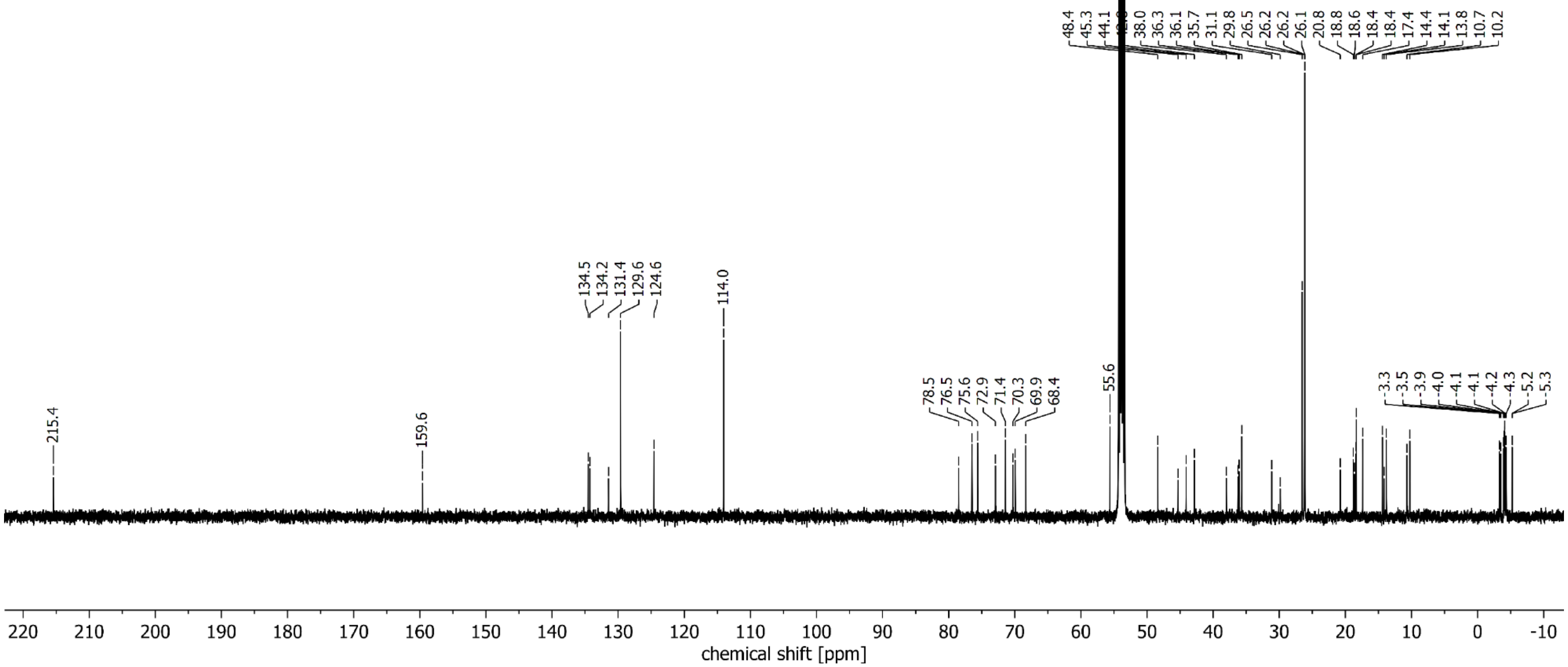

^{1}H-NMR, 500 MHz, CD_2Cl_2, 298 K

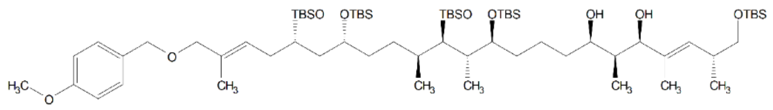

43

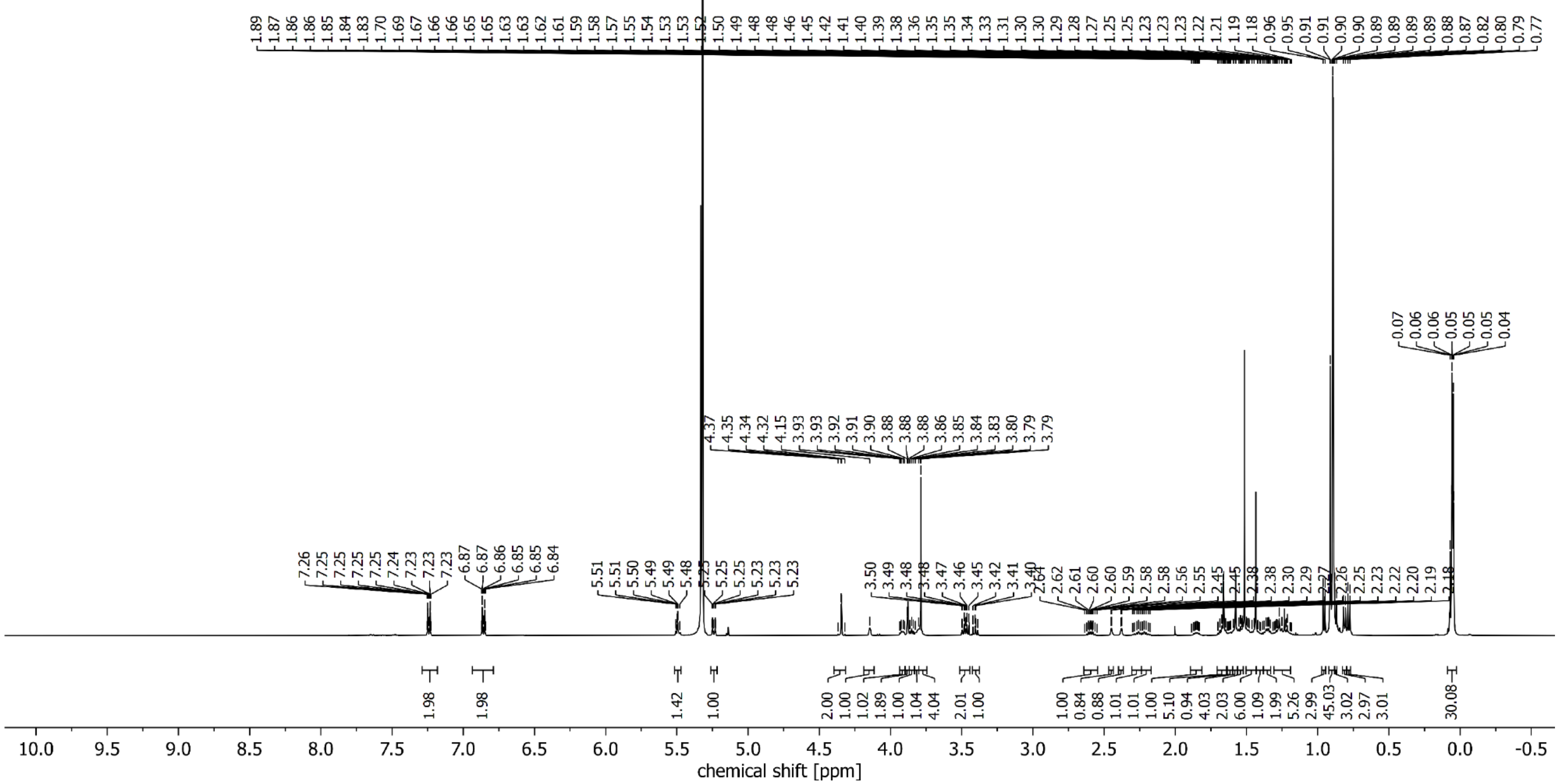

^{13}C-NMR, 126 MHz, CD_2Cl_2, 298 K

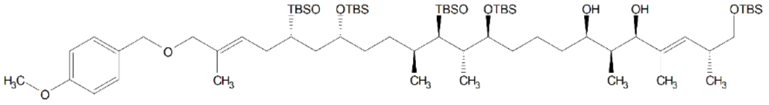

43

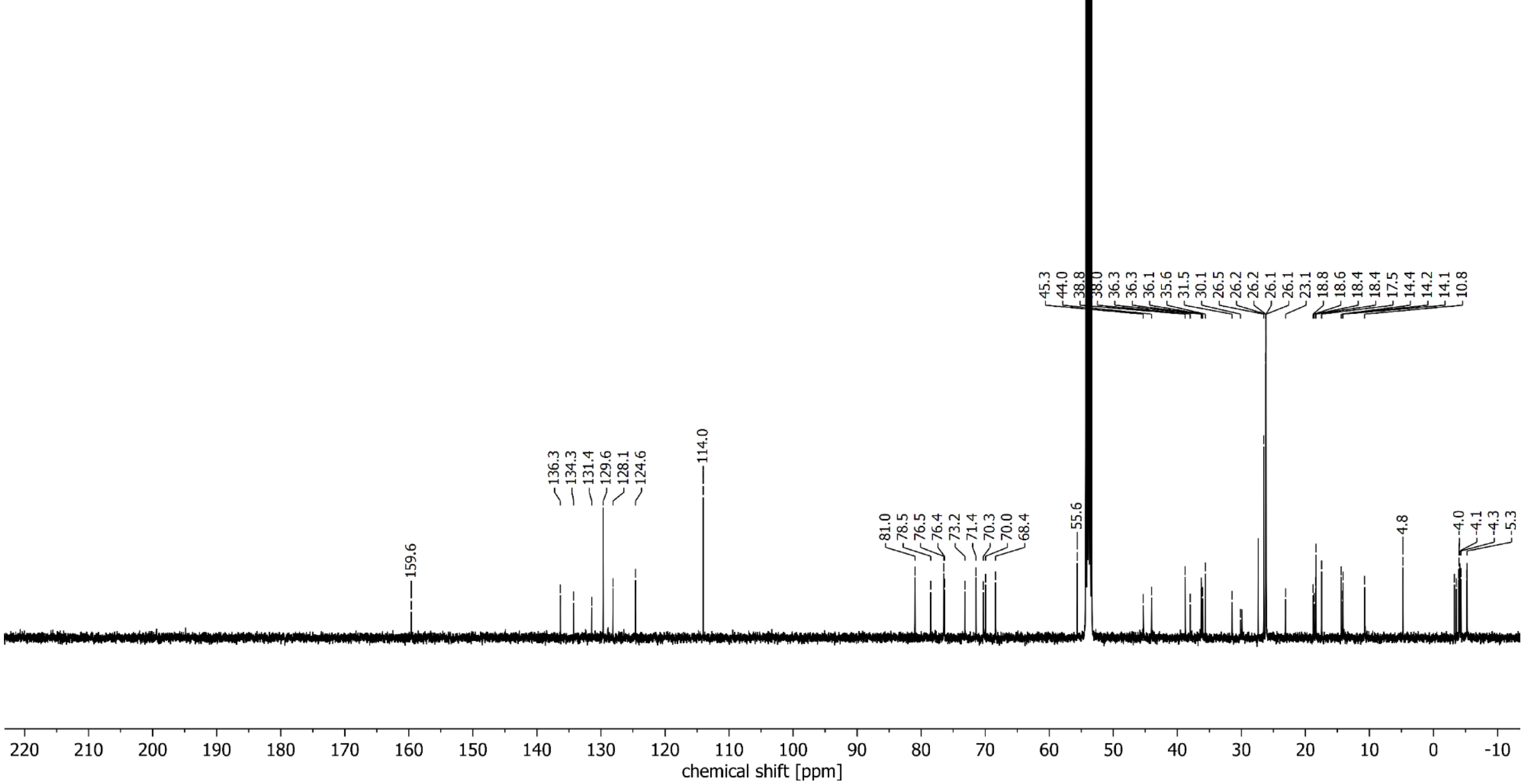

^{1}H-NMR, 700 MHz, CD_3OD, 298 K

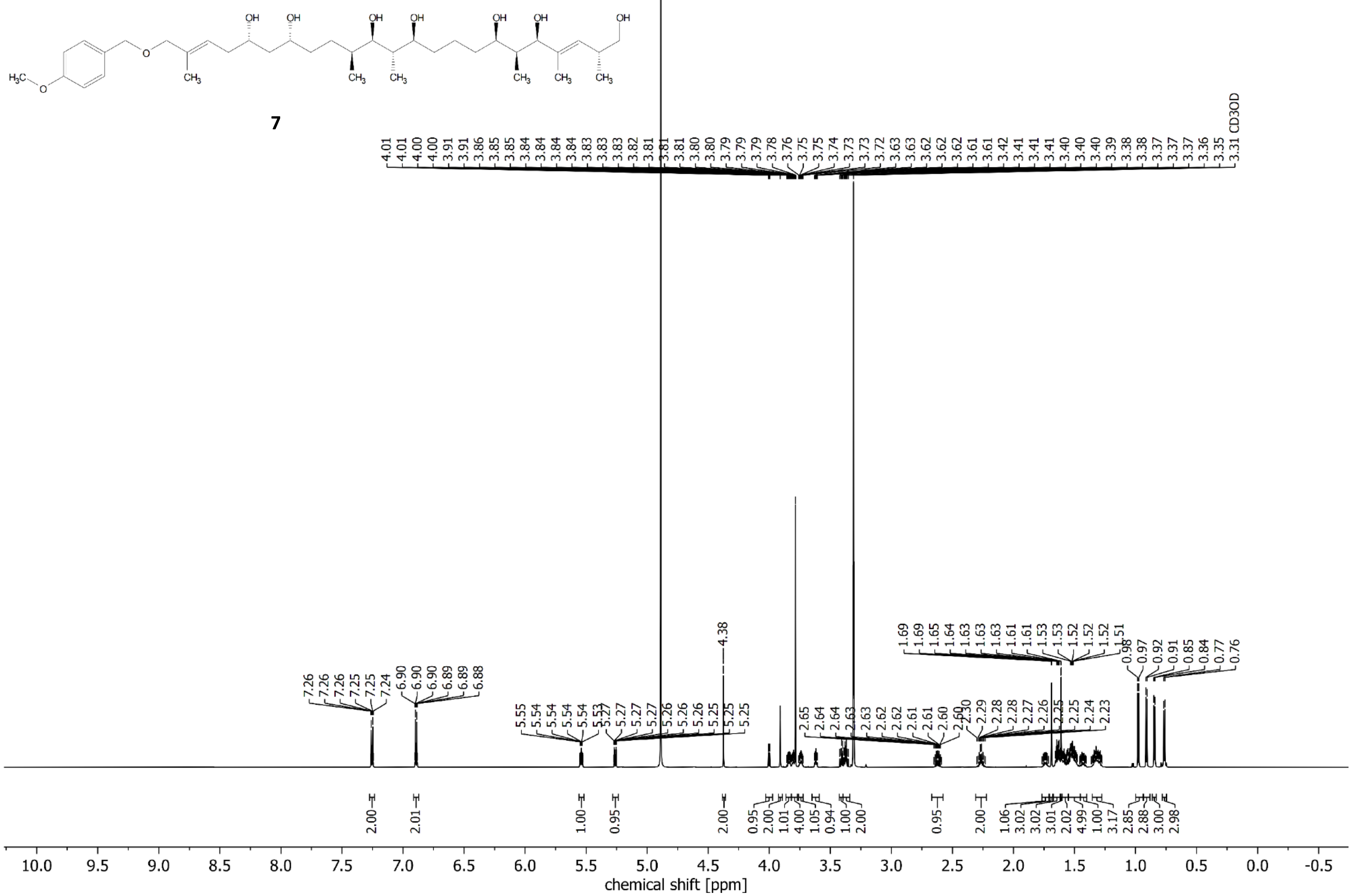

7

^{13}C-NMR, 176 MHz, CD_3OD, 298 K

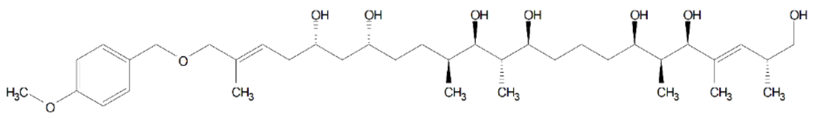

7

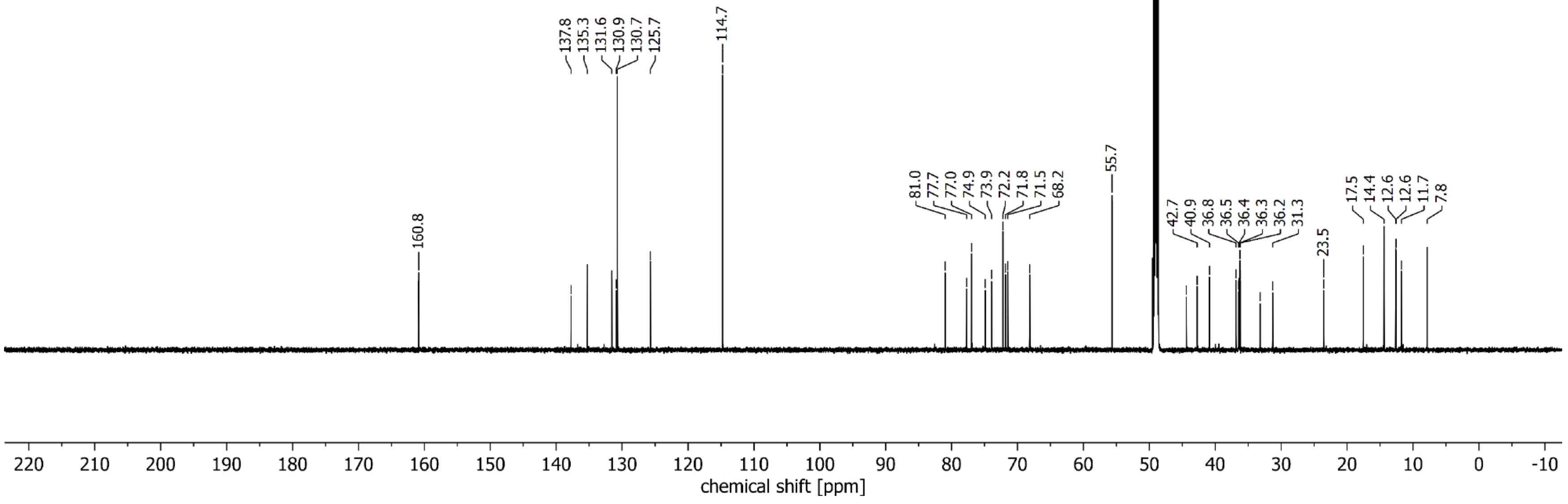

Synthesis of Mycosamine 10

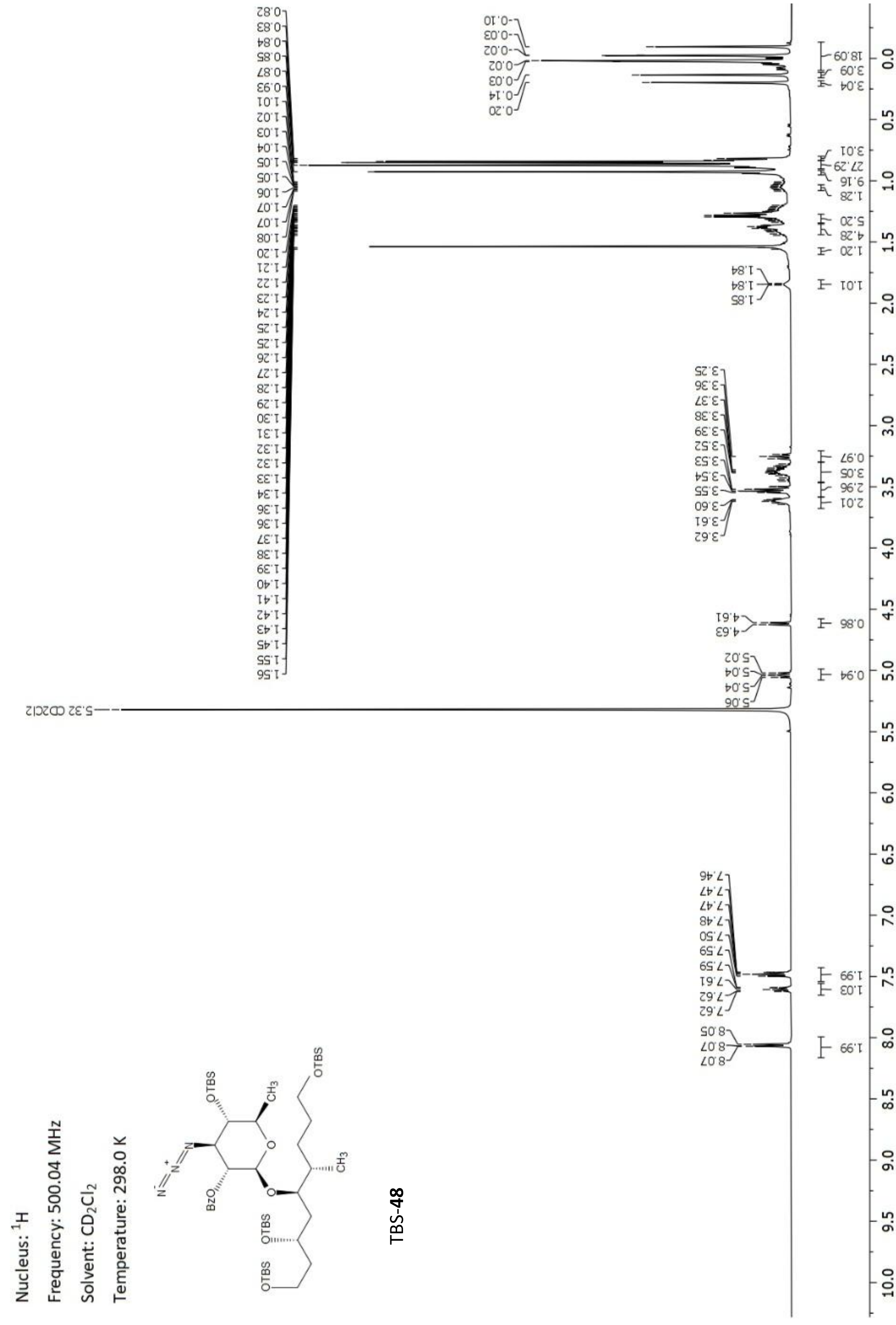

Nucleus: ^{13}C

Frequency: 125.75 MHz

Solvent: CD_2Cl_2

Temperature: 298.0 K

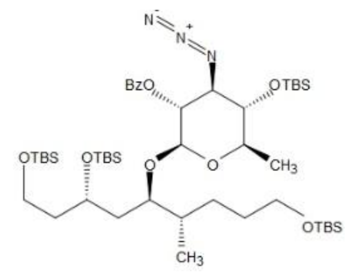

TBS-**48**

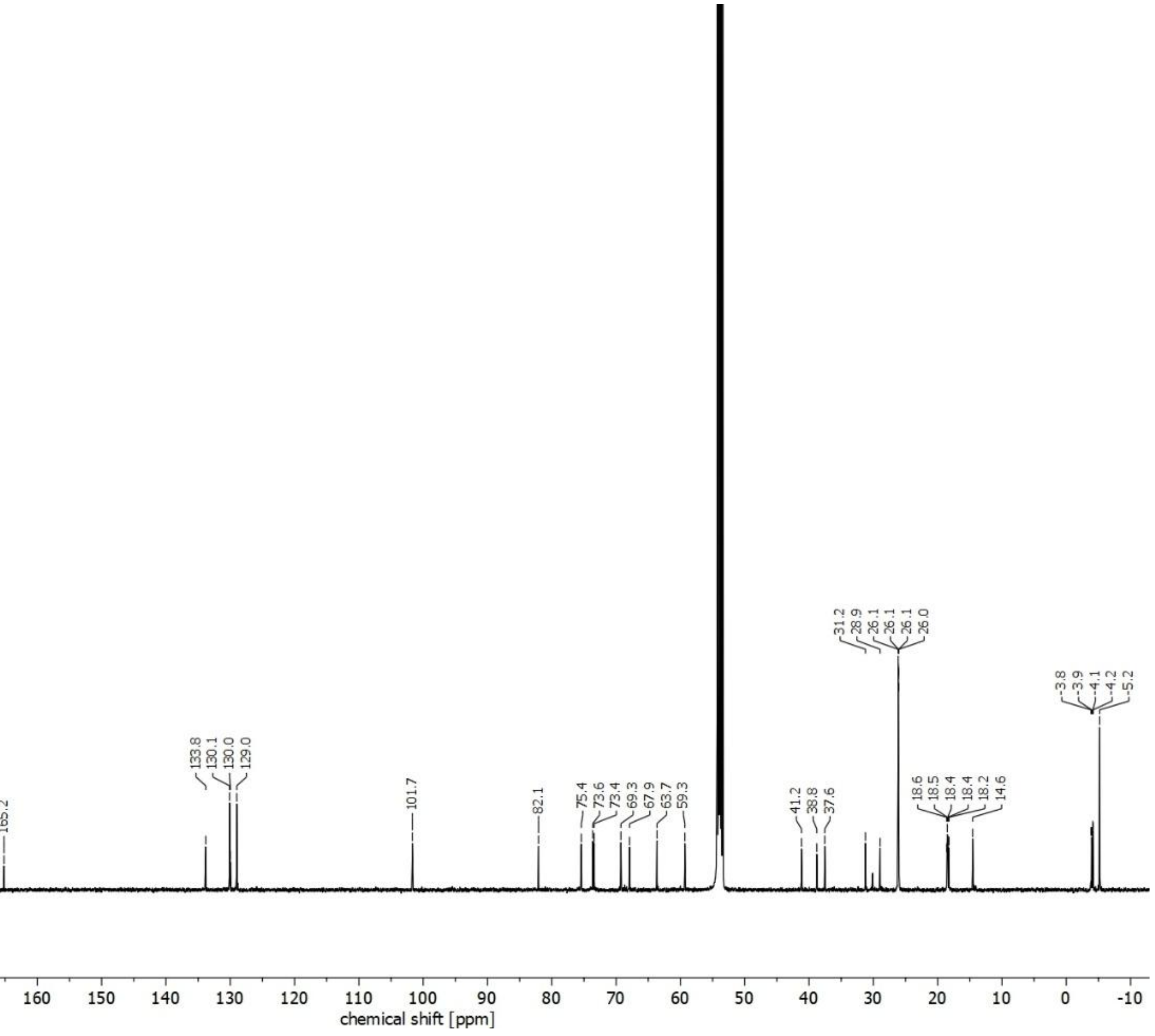

Nucleus: ^{1}H

Frequency: 700.41 MHz

Solvent: CD_2Cl_2

Temperature: 298.0 K

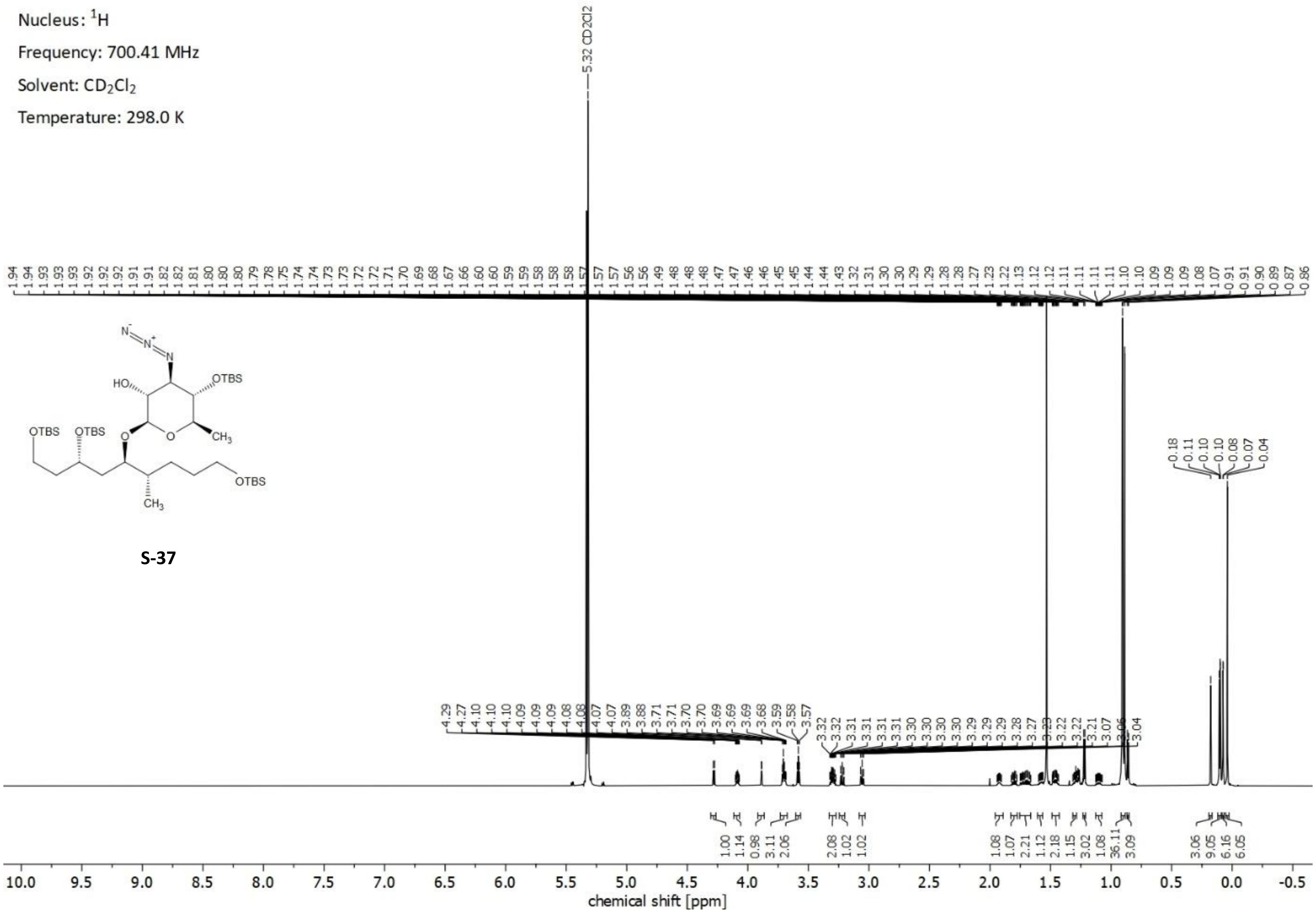

S-37

Nucleus: ^{13}C

Frequency: 176.14 MHz

Solvent: CD_2Cl_2

Temperature: 298.0 K

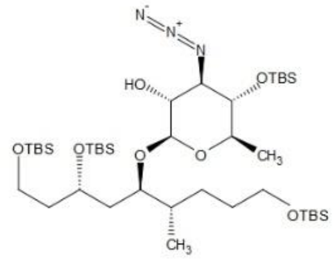

S-37

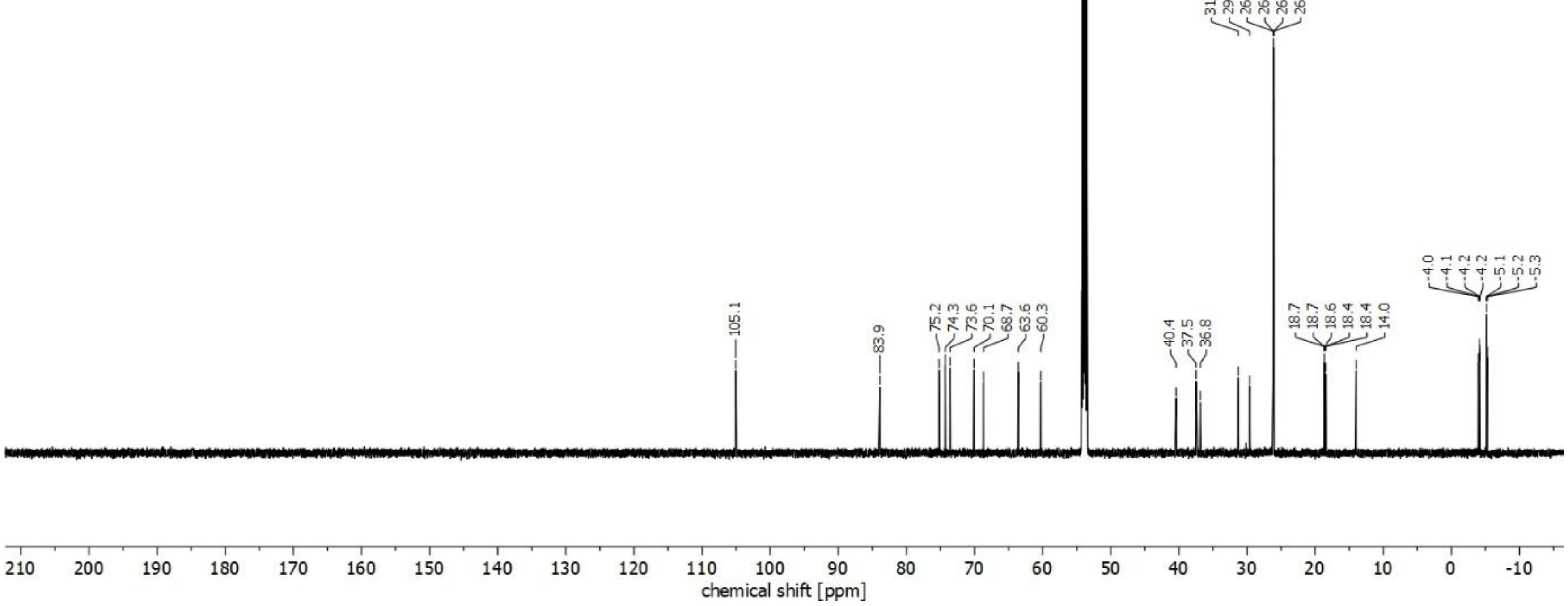

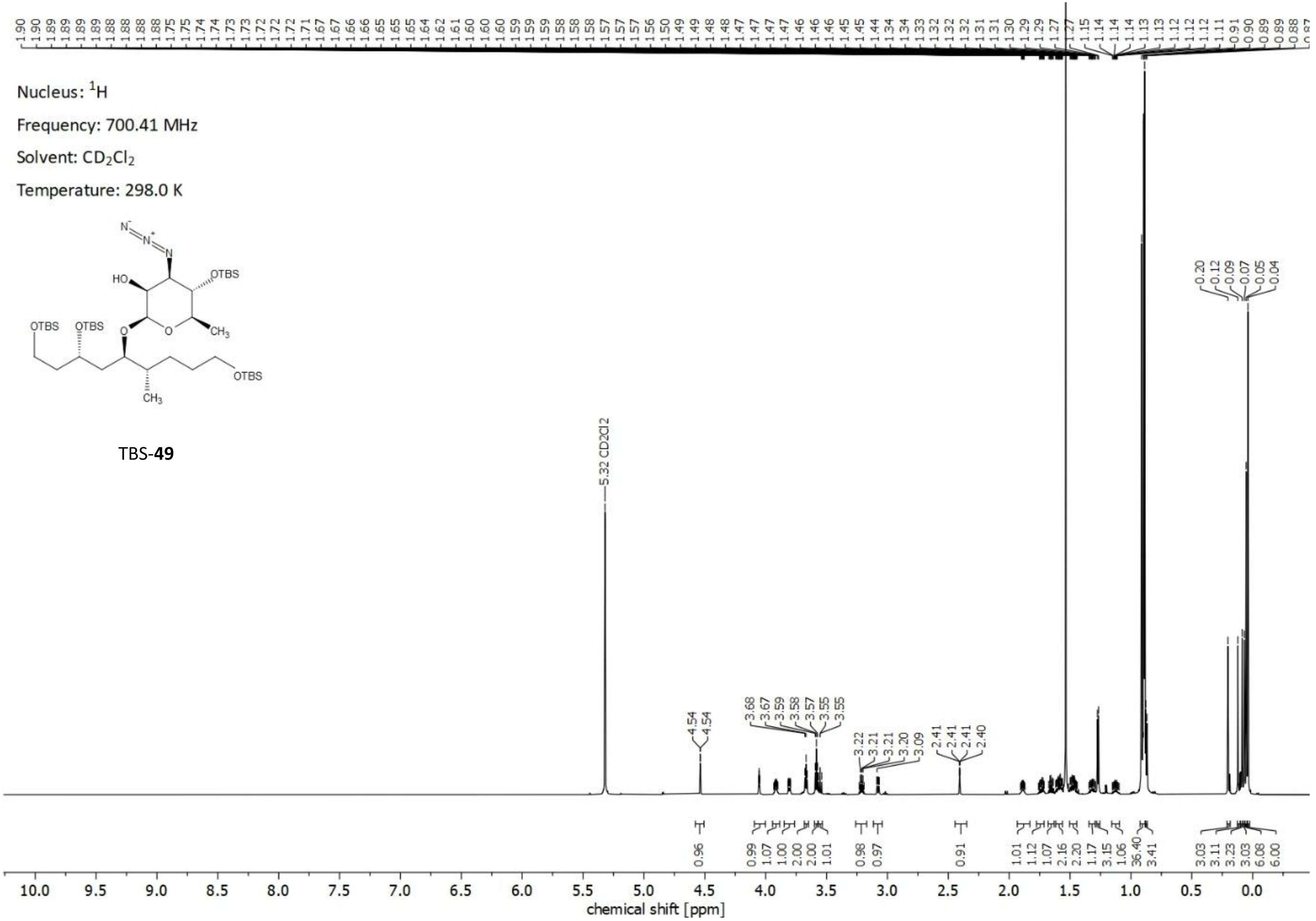
Nucleus: ^{1}H
Frequency: 700.41 MHz
Solvent: CD_2Cl_2
Temperature: 298.0 K
TBS-**49**
5.32 CD2Cl2
chemical shift [ppm]

Nucleus: ^{13}C

Frequency: 176.14 MHz

Solvent: CD_2Cl_2

Temperature: 298.0 K

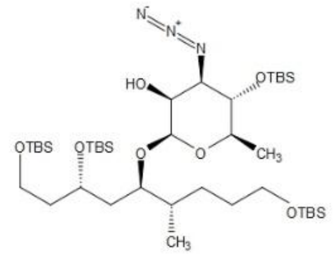

TBS-**49**

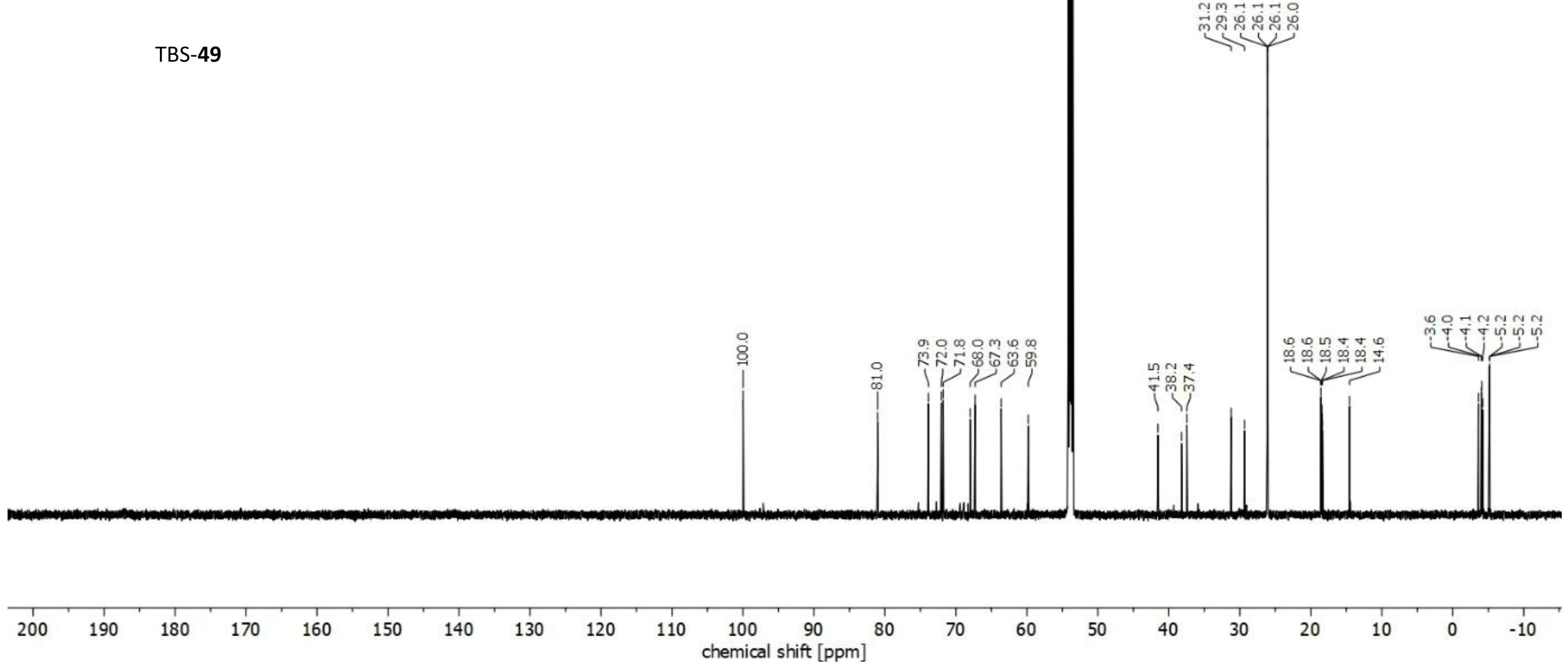

Nucleus: ^1H

Frequency: 700.41 MHz

Solvent: CD_2Cl_2

Temperature: 298.0 K

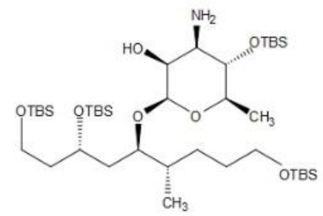

S-38

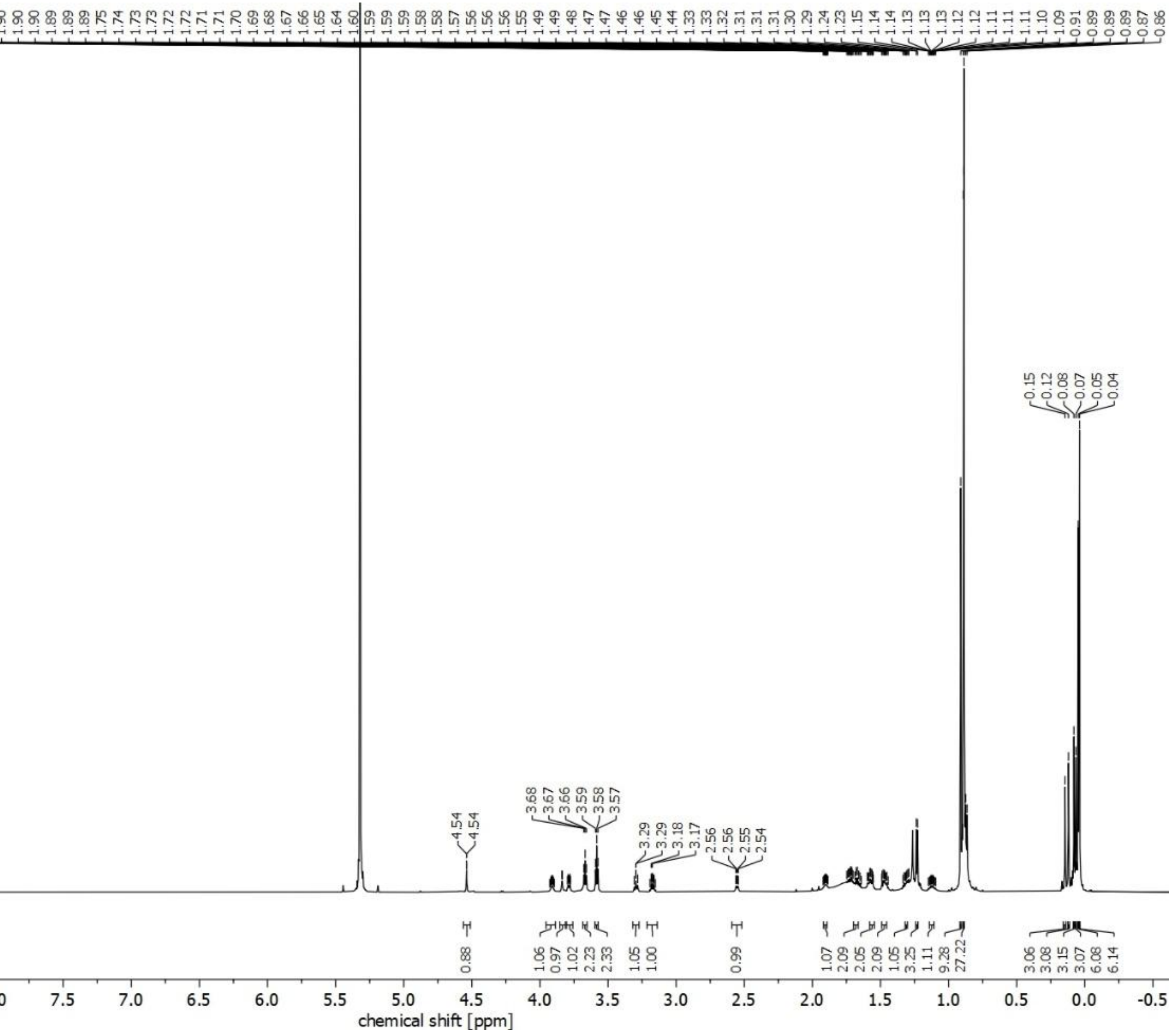

Nucleus: ^{13}C

Frequency: 176.14 MHz

Solvent: CD_2Cl_2

Temperature: 298.0 K

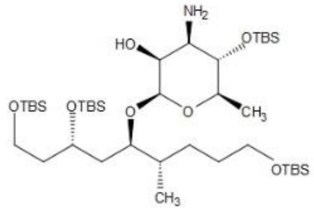

S-38

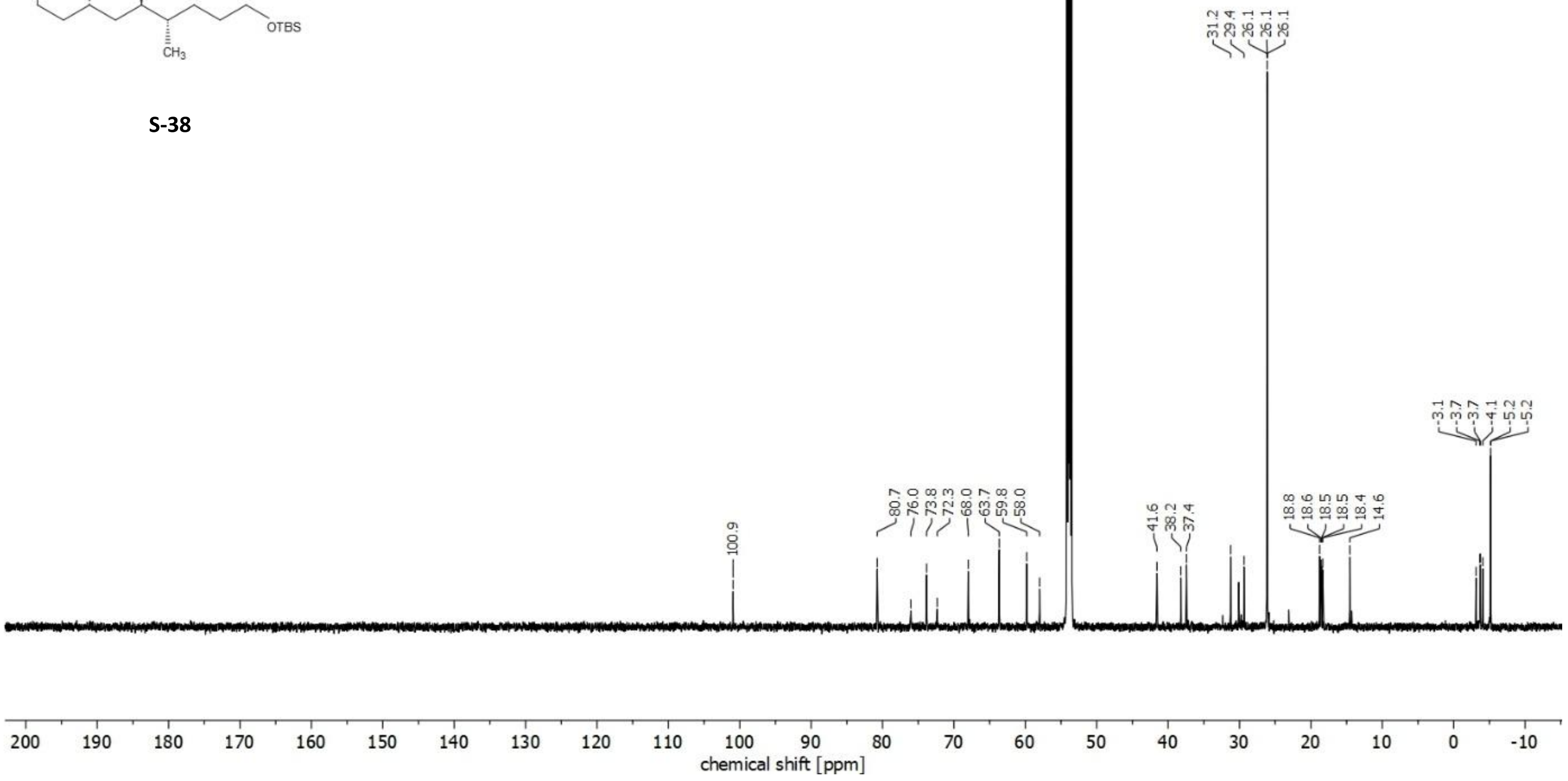

Nucleus: ^{1}H

Frequency: 499.13 MHz

Solvent: CD_2Cl_2

Temperature: 298.0 K

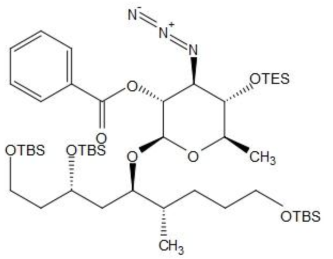

TES-**48**

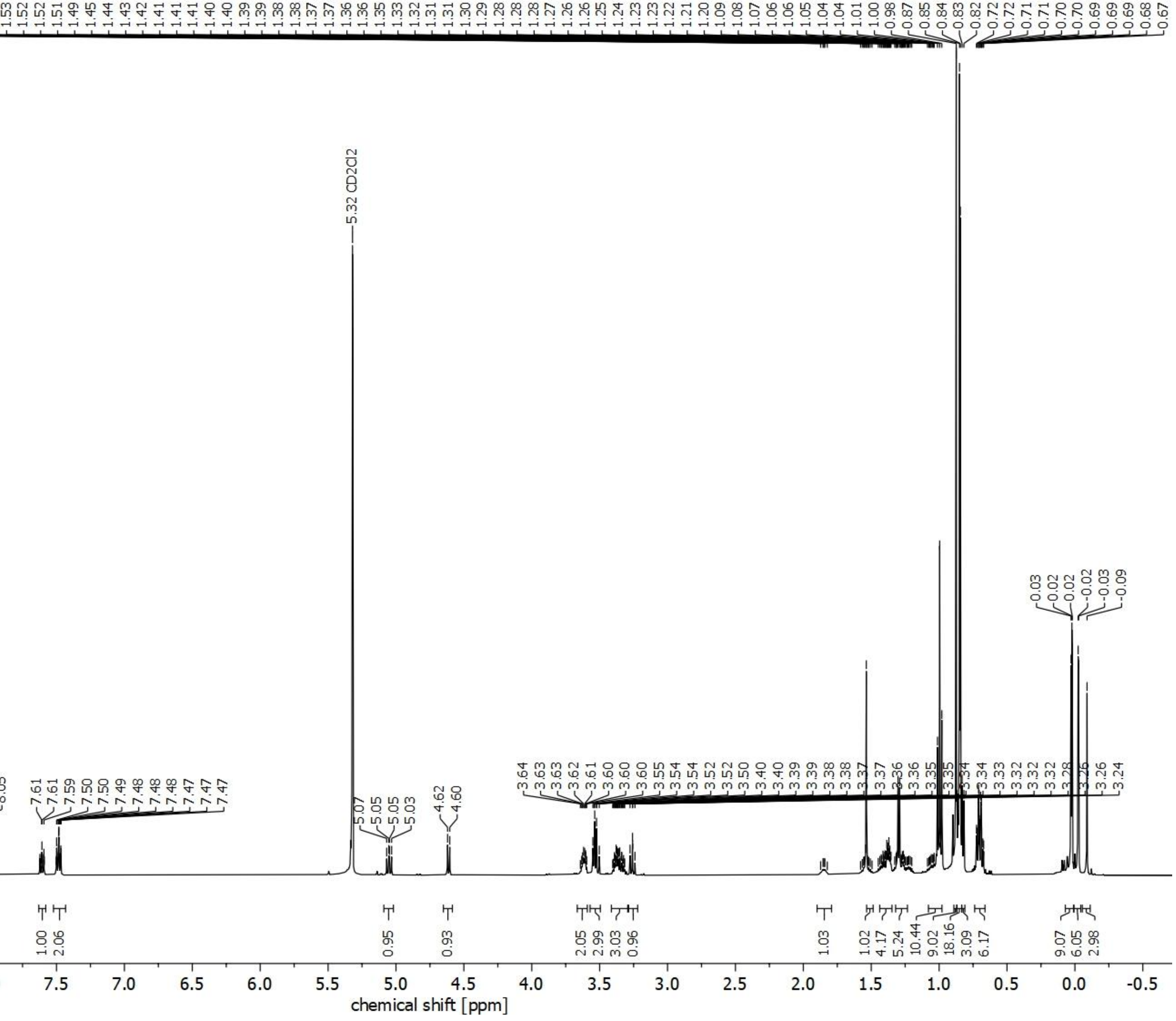

Nucleus: ^{13}C

Frequency: 125.52 MHz

Solvent: CD_2Cl_2

Temperature: 298.0 K

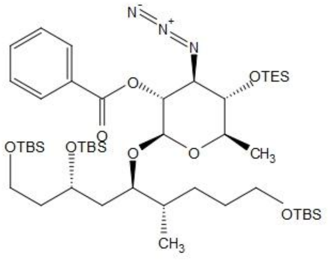

TES-**48**

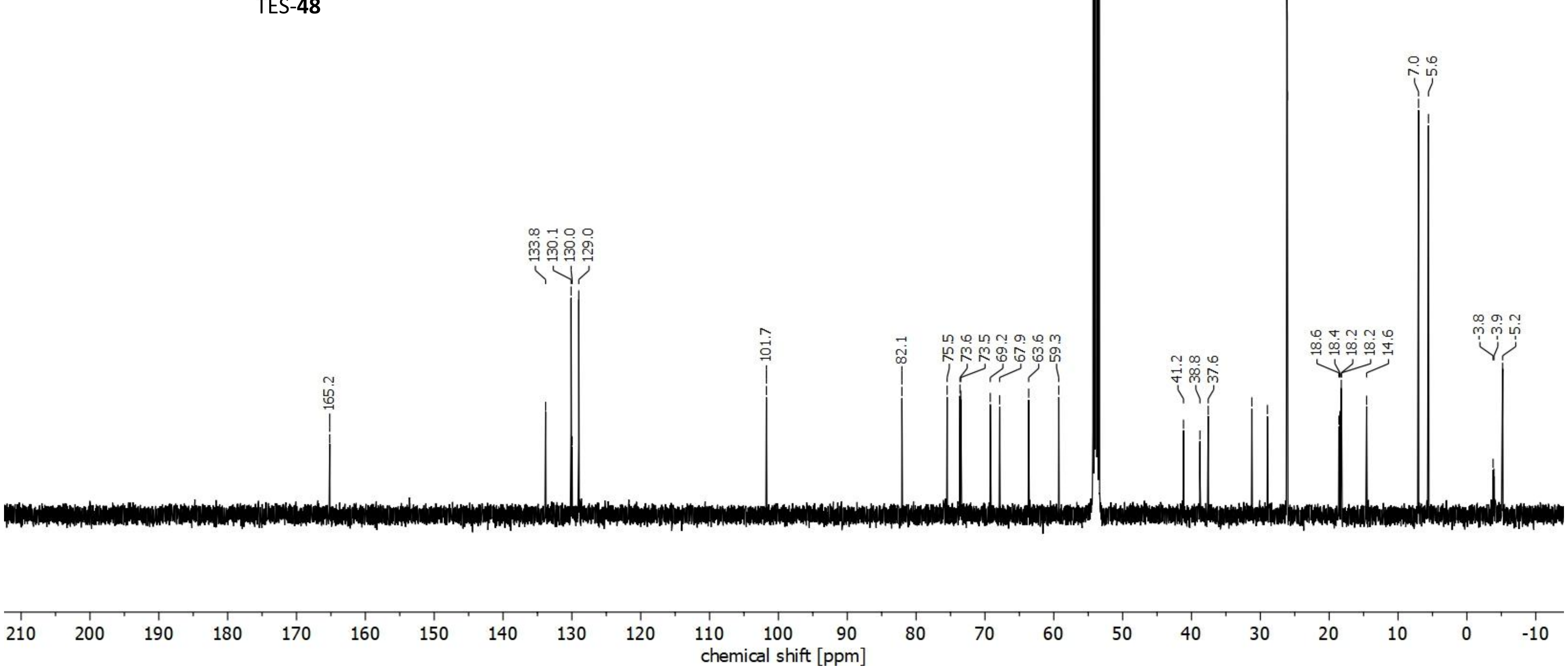

Nucleus: ^{1}H

Frequency: 500.04 MHz

Solvent: CD_2Cl_2

Temperature: 298.0 K

S-39

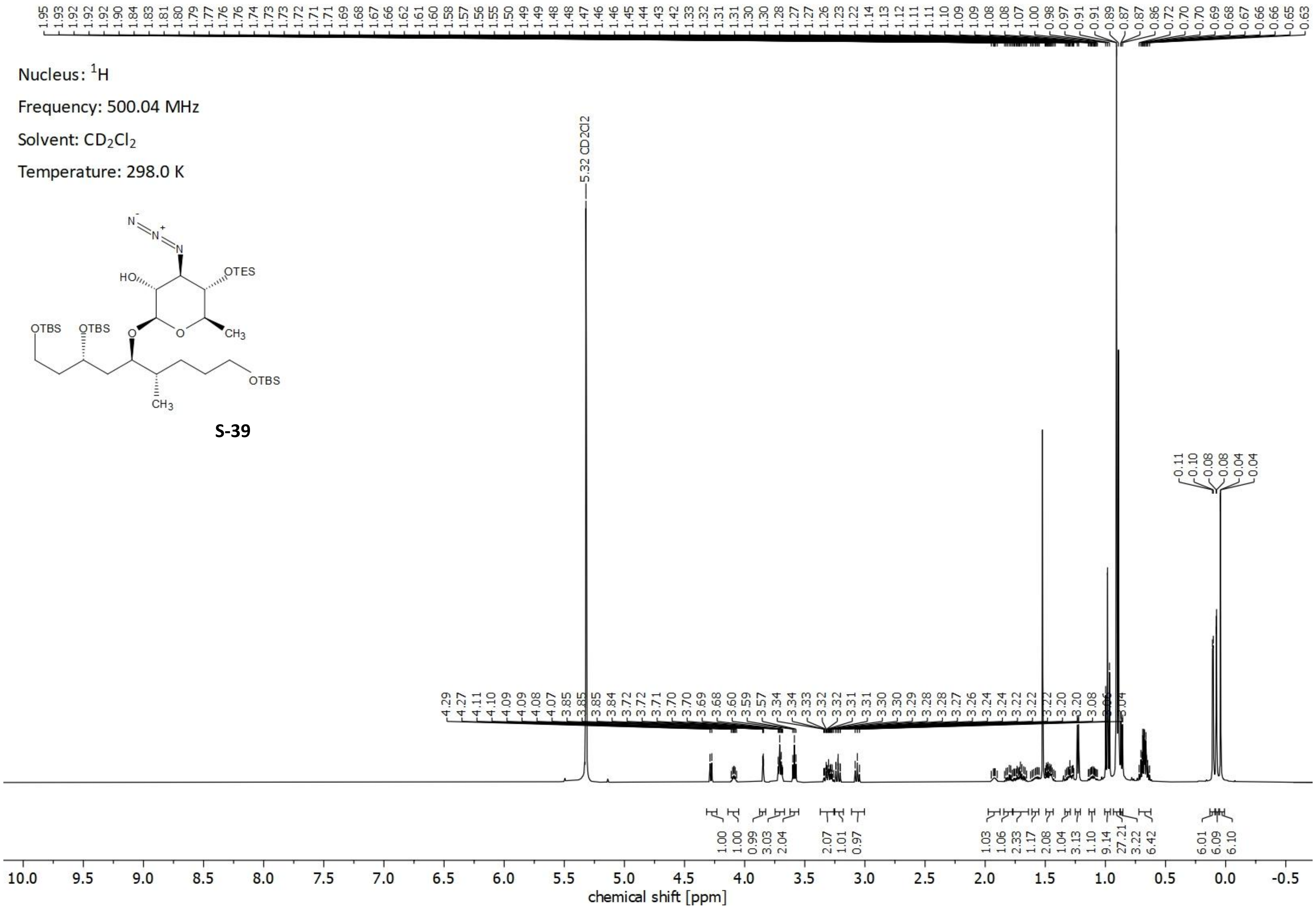

Nucleus: ^{13}C

Frequency: 125.75 MHz

Solvent: CD_2Cl_2

Temperature: 298.0 K

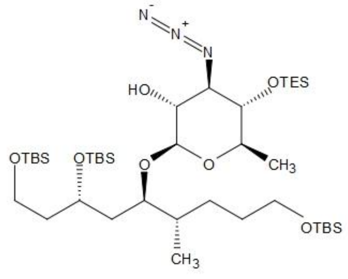

S-39

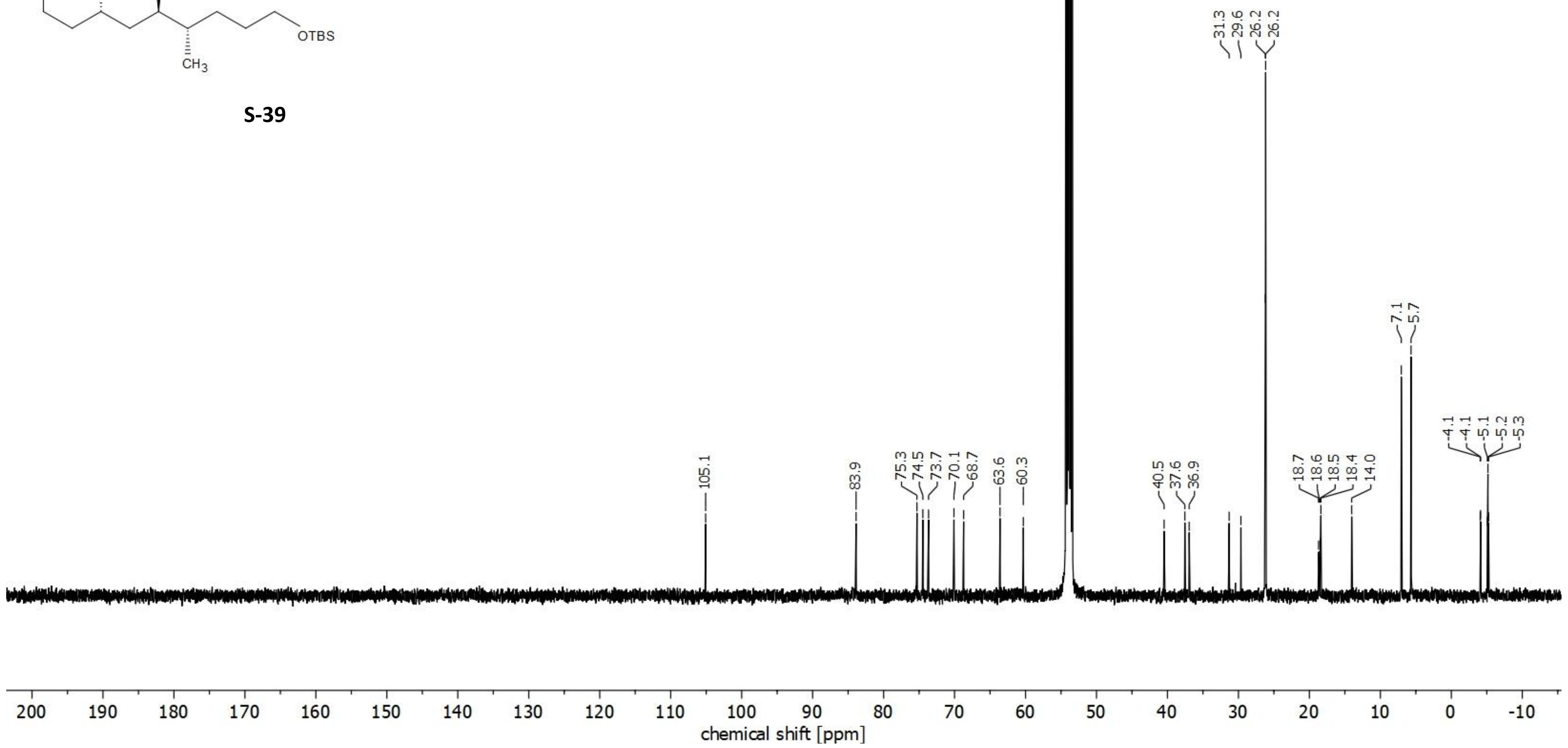

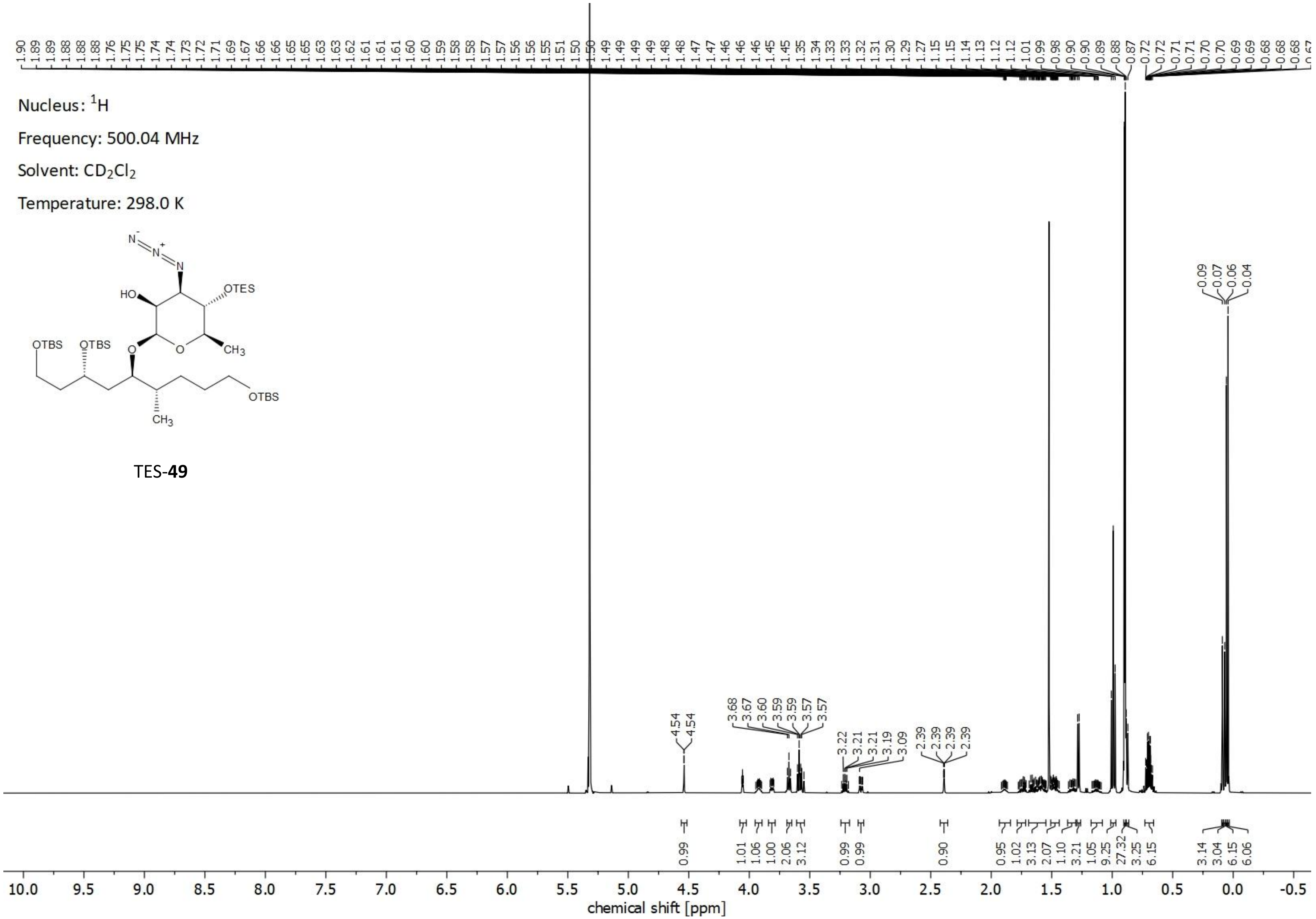
Nucleus: ^{1}H
Frequency: 500.04 MHz
Solvent: CD_2Cl_2
Temperature: 298.0 K
chemical shift [ppm]
OTES
HO
OTBS
OTBS
OTBS
O
CH_3
CH_3

TES-**49**

Nucleus: ^{13}C

Frequency: 125.75 MHz

Solvent: CD_2Cl_2

Temperature: 298.0 K

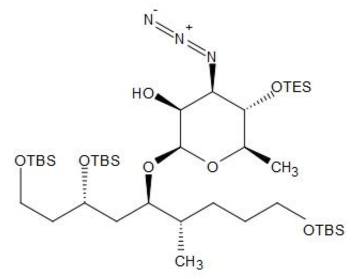

TES-**49**

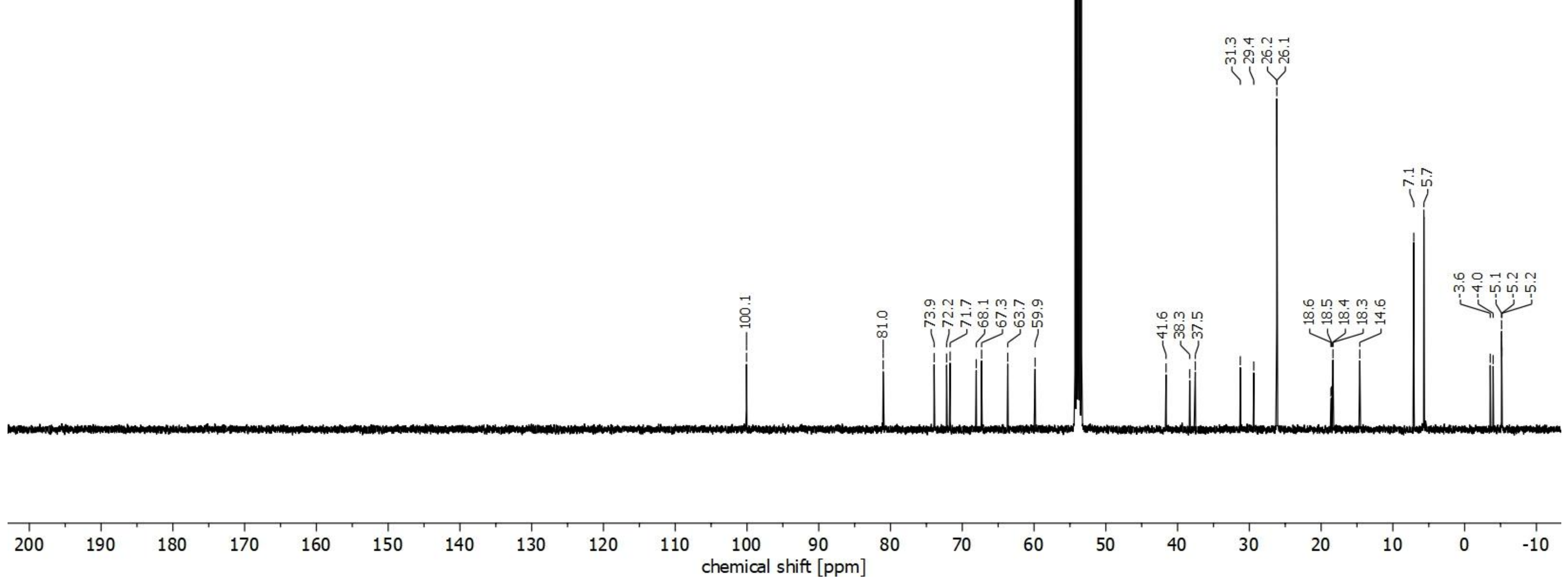

Nucleus: ^{1}H

Frequency: 500.04 MHz

Solvent: MeOD

Temperature: 298.0 K

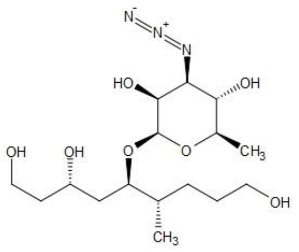

S-40

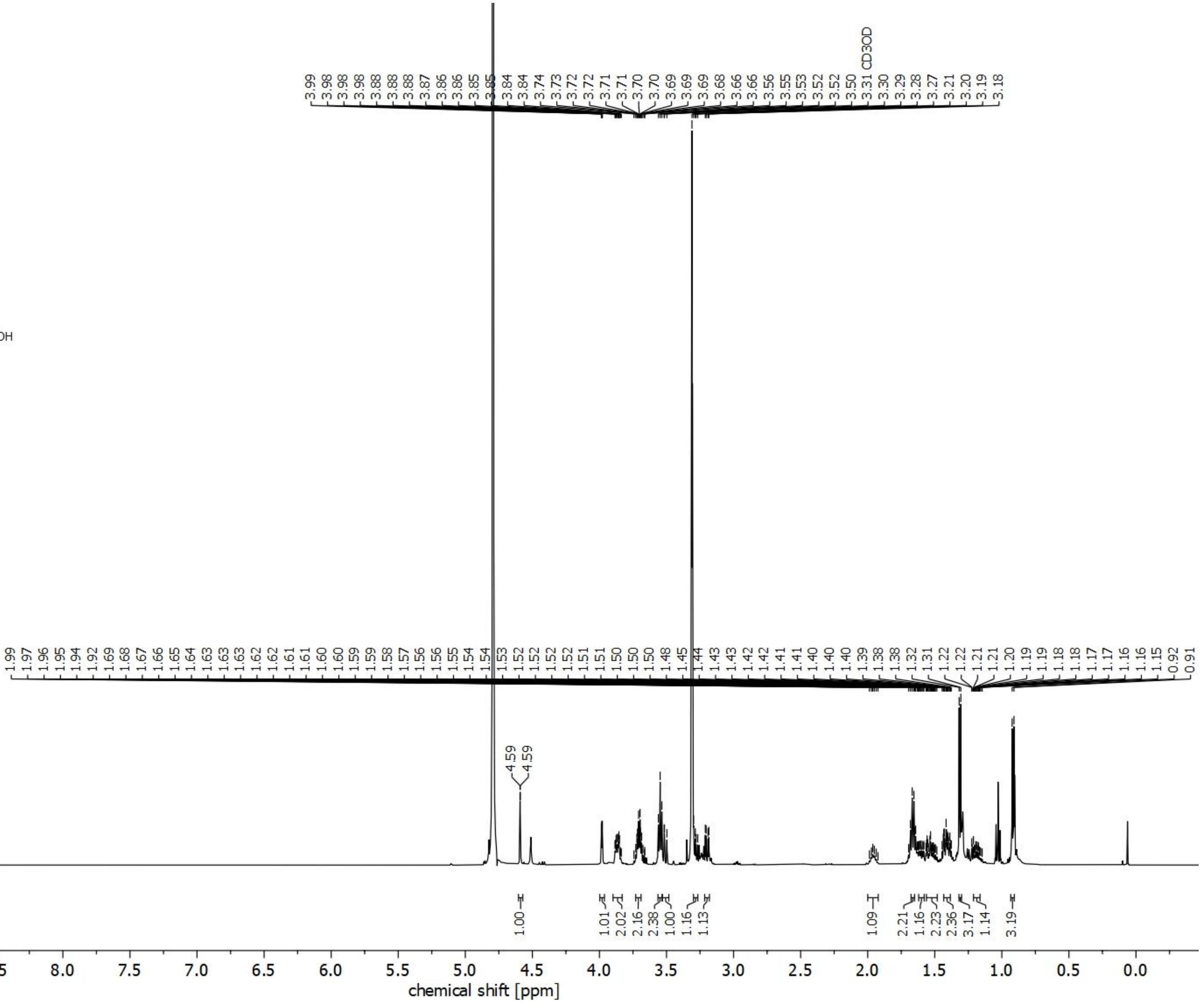

Nucleus: ^{13}C

Frequency: 125.75 MHz

Solvent: MeOD

Temperature: 298.0 K

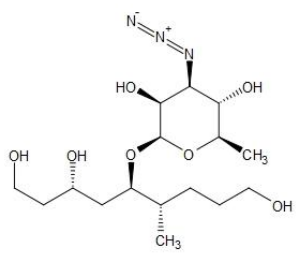

S-40

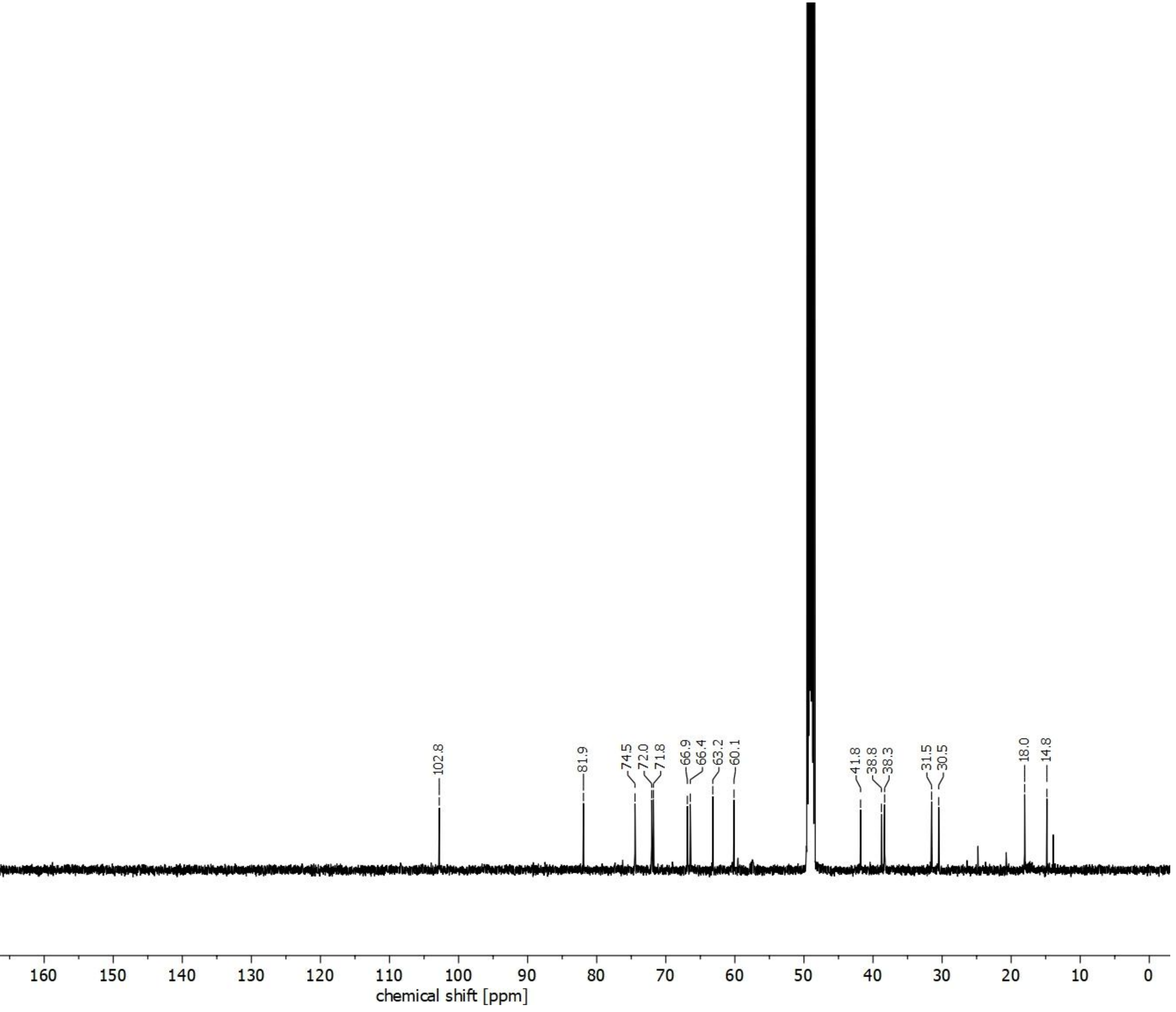

Nucleus: ^{1}H

Frequency: 500.04 MHz

Solvent: MeOD

Temperature: 298.0 K

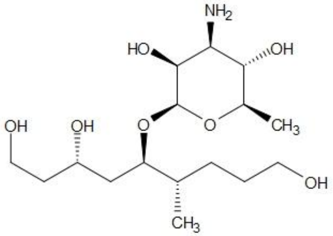

10

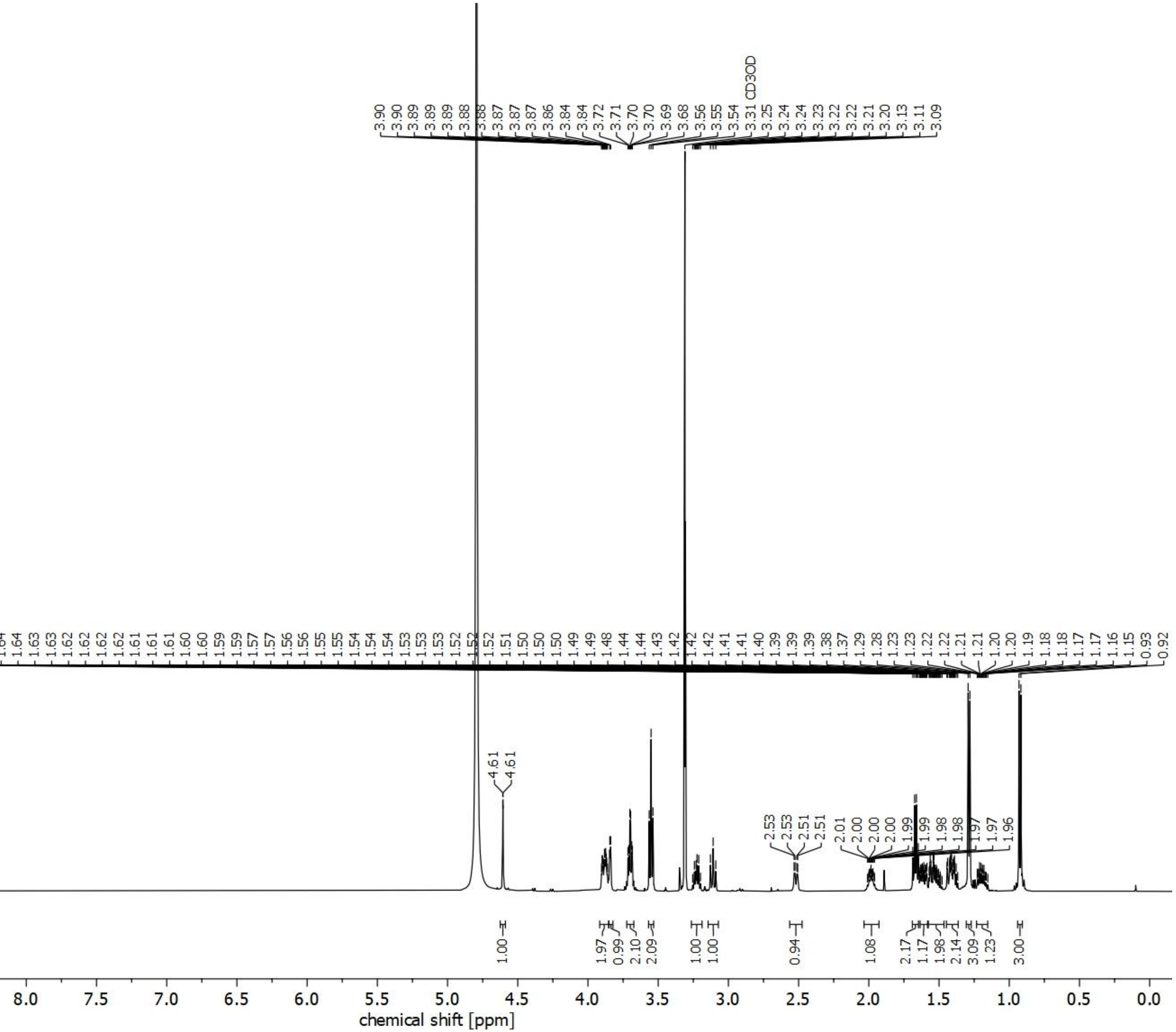

Nucleus: ^{13}C

Frequency: 125.75 MHz

Solvent: MeOD

Temperature: 298.0 K

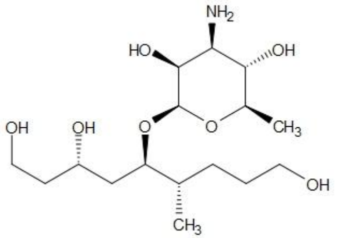

10

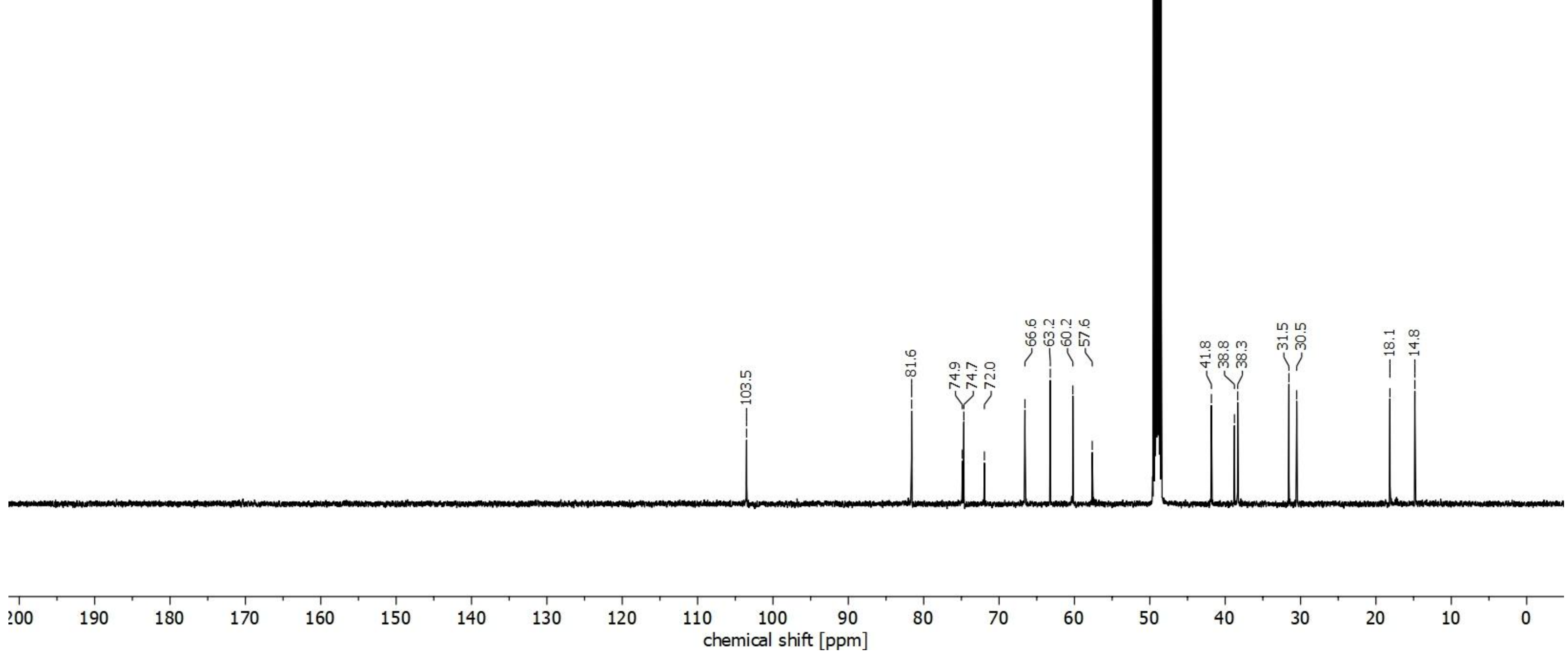

6. References

[1] S. Spindler, L. M. Wingen, M. Schönenbroicher, M. Seul, M. Adamek, S. Essig, M. Kurz, N. Ziemert, D. Menche, *Org. Lett.* **2021**, *23*, 1175–1180.

[2] M. Schönenbroicher, M. Seul, S. Morgenschweis, F. Heppner, D. Menche, *Org. Lett.* **2026**, *28*, 2194–2199.

[3] M. Schönenbroicher, M. Seul, S. Morgenschweis, F. Heppner, D. Menche, *Org. Lett.* **2026**, *28*, 3814–3819.

[4] B. A. Baker, Z. V. Bosković, B. H. Lipshutz, *Org. Lett.* **2008**, *10*, 289–292.

[5] J. M. Manthorpe, A. M. Szpilman, E. M. Carreira, *Synthesis* **2005**, 3380–3388.

[6] S. Herbert, A.-K. Ziebandt, K. Ohlsen, T. Schäfer, M. Hecker, D. Albrecht, R. Novick, F. Götz, *Infect. Immun.* **2010**, *78*, 2877–2889.

[7] U. Seybold, E. V. Kourbatova, J. G. Johnson, S. J. Halvosa, Y. F. Wang, M. D. King, S. M. Ray, H. M. Blumberg, *Clin. Infect. Dis.* **2006**, *42*, 647–656.

[8] M. Kuroda et al., *Lancet* **2001**, *357*, 1225–1240.

[9] A. Dietrich, U. Steffens, P. Sass, G. Bierbaum, *Int. J. Med. Microbiol.* **2021**, *311*, 151545.

[10] A. E. Jacob, S. J. Hobbs, *J. Bacteriol.* **1974**, *117*, 360–372.

[11] D. F. Sahm, J. Kissinger, M. S. Gilmore, P. R. Murray, R. Mulder, J. Solliday, B. Clarke, *Antimicrob. Agents Chemother.* **1989**, *33*, 1588–1591.

[12] F. Kunst et al., *Nature* **1997**, *390*, 249–256.

[13] H. Harms, A. Klöckner, J. Schrör, M. Josten, S. Kehraus, M. Crüsemann, W. Hanke, T. Schneider, T. Schäberle, G. König, *Planta Med.* **2018**, *84*, 1363–1371.

[14] J. Radeck, S. Gebhard, P. S. Orchard, M. Kirchner, S. Bauer, T. Mascher, G. Fritz, *Mol. Microbiol.* **2016**, *100*, 607–620.

[15] Clinical and Laboratory Standards Institute, *Performance standards for antimicrobial susceptibility testing*. *Twenty-forth informational supplement*, CLSI, **2014**.

[16] H. Strahl, L. W. Hamoen, *Proc. Natl. Acad. Sci. USA* **2010**, *107*, 12281–12286.

[17] J. Schindelin et al., *Nat. Methods* **2012**, *9*, 676–682.

[18] T. Schneider et al., *Science* **2010**, *328*, 1168–1172.

[19] P. Pracht et al., *J. Chem. Phys.* **2024**, *160*.

[20] C. Bannwarth, S. Ehlert, S. Grimme, *J. Chem. Theory Comput.* **2019**, *15*, 1652–1671.

[21] S. Ehlert, M. Stahn, S. Spicher, S. Grimme, *J. Chem. Theory Comput.* **2021**, *17*, 4250–4261.

[22] S. Grimme, F. Bohle, A. Hansen, P. Pracht, S. Spicher, M. Stahn, *J. Phys. Chem. A* **2021**, *125*, 4039–4054.

[23] S. Grimme, A. Hansen, S. Ehlert, J.-M. Mewes, *J. Chem. Phys.* **2021**, *154*, 64103.

[24] F. Neese, *WIREs Comput. Mol. Sci.* **2025**, *15*.

[25] F. Neese, F. Wennmohs, U. Becker, C. Riplinger, *J. Chem. Phys.* **2020**, *152*, 224108.

[26] V. Barone, M. Cossi, *J. Phys. Chem. A* **1998**, *102*, 1995–2001.
